# Supplementary material for: Metal-free photoinduced C(sp3)–H/C(sp3)–H cross-coupling to access α‑tertiary amino acid derivatives
Source: Nat Commun. 2023 Oct 6;14:6225. doi: 10.1038/s41467-023-41956-6 (PMC10558569; doi:10.1038/s41467-023-41956-6)
Supplement: Supplementary file 1 — Supplementary Information [file 41467_2023_41956_MOESM1_ESM.pdf]

Supplementary Information

Metal-free Photoinduced C(sp^3)–H/C(sp^3)–H Cross-Coupling to access α-Tertiary Amino Acid Derivatives

Yujun Li[1], Shaopeng Guo[2], Qing-Han Li[2] and Ke Zheng[1]*

[1] Key Laboratory of Green Chemistry & Technology, Ministry of Education, College of Chemistry, Sichuan University, Chengdu 610064, P. R. China.

[2] Key Laboratory of Pollution Control Chemistry and Environmental Functional Materials for Qinghai-Tibet Plateau of the National Ethnic Affairs Commission, College of Chemistry and Environment, Southwest Minzu University, Chengdu, 610041, P.R. China.

*Correspondence author: E-mail: kzheng@scu.edu.cn

Table of content

Supplementary Methods 3

Supplementary Discussion 6

Supplementry Notes 17

Mechanistic Studies 17

Substrate Synthesis 36

Analytical Data of Compounds 38

NMR Spectra 74

Supplementary References 178

Supplementary Methods

All required fine chemicals were used directly without purification unless stated otherwise. All air and moisture sensitive reactions were carried out under nitrogen atmosphere using standard Schlenk manifold technique. Pivalonitrile (*t*-BuCN) [98%, CAS: 630-18-2] was purchased from Adamas. Other solvents were bought from *J&K Scientific* as 99.9% purity under 4 Å molecular sieves. Other commercial reagents were purchased from Adamas, TCI, Aldrich, Bidepharm and Alfa. Reactions were monitored by thin layer chromatography (TLC) using silica gel 60 F-254 plates. Flash chromatography columns were packed with 200-300 mesh silica gel. ^{1}H, ^{13}C and ^{19}F NMR spectra were acquired at various field strengths, as indicated, using Bruker 400 or 600 MHz spectrometers. All spectral data was acquired at 295 K. Deuterated solvents were purchased from Adamas. ^{1}H and ^{13}C chemicals shifts (δ) are quoted in parts per million (ppm) against tetramethylsilane (TMS, δ = 0.00 ppm) and were internally referenced to residual $CHCl_3$ (7.26 ppm for ^{1}H, 77.16 ppm for ^{13}C) or DMSO (2.50 ppm for ^{1}H, 39.52 ppm for ^{13}C). ^{19}F chemicals shifts (δ) are quoted in parts per million (ppm) and were calibrated using absolute referencing to the ^{1}H NMR spectrum. Coupling constants (J) are reported in Hertz (Hz) to the nearest 0.1 Hz. The following abbreviations (or combinations thereof) were used to explain multiplicities: s = singlet, d = doublet, t = triplet, br = broad, m = multiplet. High-resolution mass spectra (HRMS) were recorded on a UPLC of Thermo Q Exactive Focus. UV-Vis absorption spectra were recorded using 1 cm quartz cuvettes on a Thermo NANODROP 2000C Spectrophotometer. Cyclic voltammograms were obtained on a CHI 600E potentiostat. GC- MS were carried out on a PerKinElmer−Clarus 690 and PerKinElmer Clarus SQ 8T.

Standard Reaction Setup:

The setup (shown below Figure 1) is employed to photochemical organic synthesis reaction, which is made up of separable base and reaction hole. The integrated light panel with certain wavelength can be embedded into the sliding groove of the base. Due to the hollow design, the reaction can be kept at an ideal temperature through cold or hot medium. In a typical reaction, Schlenk tube was inserted into the hole and the reaction mixture is irradiated under 10 W LEDs light with 1.0 cm distance.

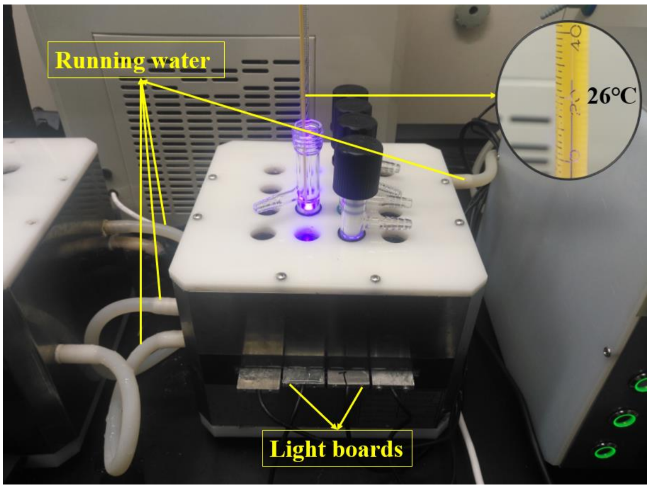

Supplementary Figure 1 16-hole parallel photoreactor (PhotoSyn 3.0)

General Procedure:

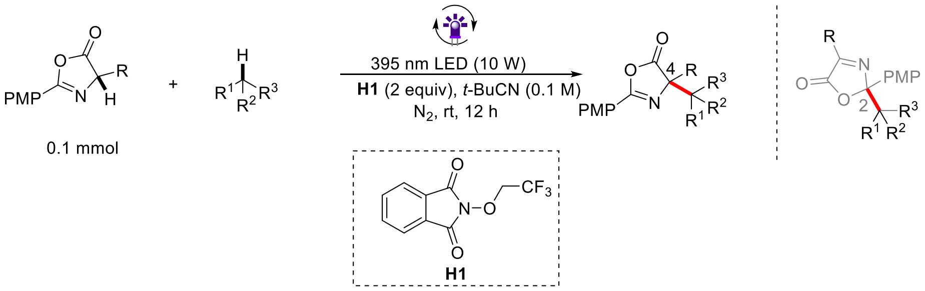

Procedure A: An oven-dried 10-mL Schlenk tube equipped with a stirrer was charged with the oxazolones (0.1 mmol, 1.0 equiv.) and the phthalimide **H1** (0.2 mmol, 2.0 equiv). Then, 1.0 mL *t*-BuCN (0.1 M) was added followed by the alkane (0.5 mmol, 5 equiv) in glove box. The tube was sealed with a screw cap and took out from glove box. The reaction mixture was inserted into the PhotoSyn 3.0 reactor and irradiated using a 10 W LED lamp (395 nm) for 12 h. The reaction mixture was concentrated in vacuo and purified by flash column chromatography (petroleum ether/EA = 20/1).

Procedure B: An oven-dried 10-mL Schlenk tube equipped with a stirrer was charged with the oxazolones (0.1 mmol, 1.0 equiv.) and the phthalimide **H1** (0.2 mmol, 2.0 equiv). Then, 1.0 mL *t*-BuCN (0.1 M) was added followed by the alkane (1.0 mmol, 10 equiv) in glove box. The tube was sealed with a screw cap and took out from glove box. The reaction mixture was inserted into the PhotoSyn 3.0 reactor and irradiated using a 10 W LED lamp (395 nm) for 12 h. The reaction mixture was concentrated in vacuo and purified by flash column chromatography (petroleum ether/EA = 30/1).

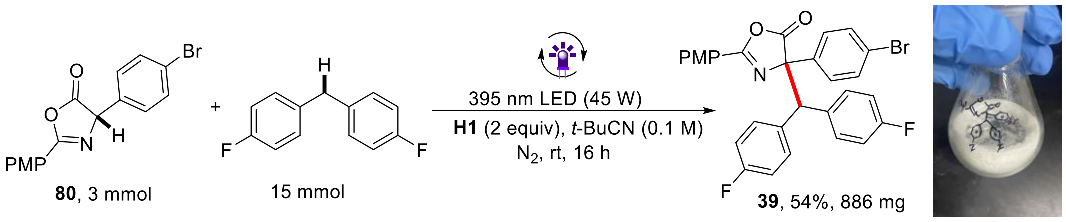

Procedure C: An oven-dried round bottom flask was charged with the oxazolone **80** (3.0 mmol, 1.03 g) and **H1** (6.0 mmol, 1.47 g, 2 equiv). Then, *t*-BuCN (30 mL) was added in glove box, followed by **4,4'-Difluorodiphenylmethane** (15 mmol, 3.06 g, 5 equiv). The round bottom flask was taken out of the glove box. The solution was irradiated with 395nm LED (45 W) at 22~28 °C for 16 h. After completed, the white precipitate was filtered off and the solution was concentrated in vacuo and purified by flash column chromatography (petroleum ether/EA = 20/1).

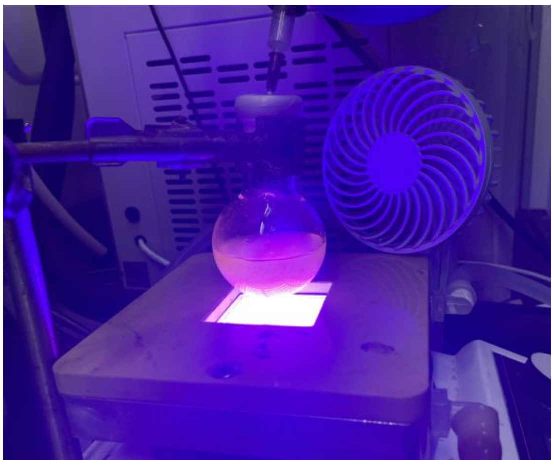

Supplementary Figure 2 Set-up diagram for reaction

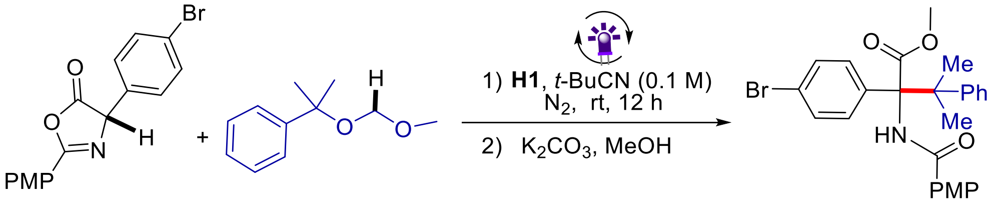

80 **81** **82**

Procedure D: An oven-dried 10-mL Schlenk tube equipped with a stirrer was charged with the oxazolone **80** (0.1 mmol, 1.0 equiv.) and the phthalimide **H1** (0.2 mmol, 2.0 equiv). Then, 1.0 mL *t*-BuCN (0.1 M) was added followed by the alkane **81** (0.5 mmol, 5 equiv) in glove box. The tube was sealed with a screw cap and took out from glove box. The reaction mixture was inserted into the PhotoSyn 3.0 reactor and irradiated using a 10 W LED lamp (395 nm) for 12 h. The reaction mixture was concentrated in vacuo. Then, methanol (1 mL) and anhydrous K_2CO_3 (14 mg, 0.1 mmol) were added. The mixture was stirred at room temperature., the crude mixture was purified by flash column chromatography (petroleum ether/ethyl acetate = 3 : 1) to give the title compound **82** (23.7 mg, 49% yield).

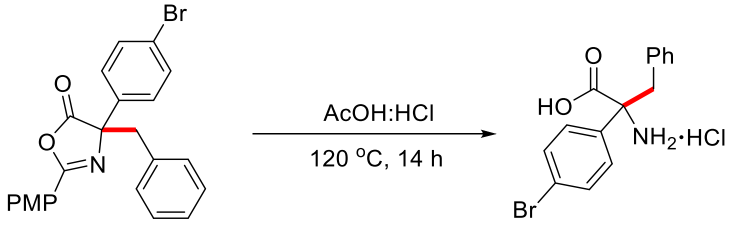

12, 0.2 mmol **83**, 80%

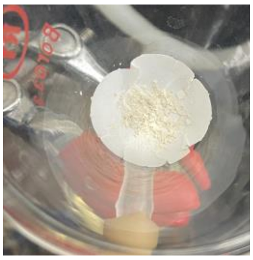

Procedure E: A 10 mL flask was charged with **12** (87 mg, 0.2 mmol) and AcOH : HCl (12 M) = 1:1 (1.0 mL, V/V). The mixture was heated at 120 °C for 14 h under N_2. After concentration under reduced pressure, the crude product was washed with diethyl ether and ethyl acetate to give 2-amino-2-(4-bromophenyl)-3-phenylpropanoic acid hydrochloride **83**(56.7 mg, 80% yield) as white solid.

^{1}H NMR (400 MHz, DMSO) δ 9.01 (br, 3H), 7.76 – 7.51 (m, 4H), 7.41 – 7.25 (m, 5H), 3.66

– 3.56 (m, 2H).

^{13}C NMR (101 MHz, DMSO) δ 170.72, 136.02, 133.77, 131.91, 131.37, 129.33, 128.91, 128.01, 122.85, 65.59, 41.57.

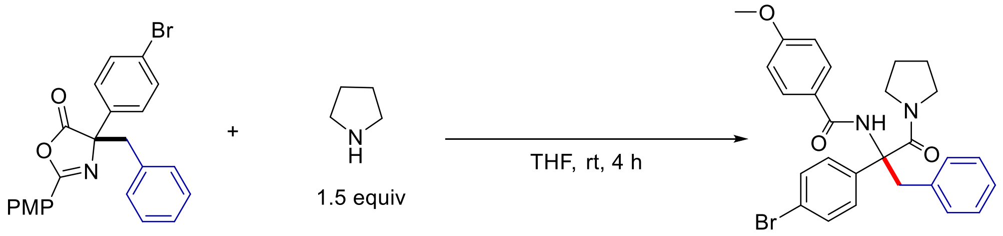

Procedure F: A 10 mL flask was charged with **12** (43.5 mg, 0.1 mmol), THF (1.0 mL) and Pyrrolidine (0.15 mmol) The mixture was stirred at room temperature.for 4 h, the crude mixture was purified by flash column chromatography (petroleum ether/ethyl acetate = 3 : 1) to give the title compound **84** (36.0 mg, 71% yield).

N-(2-(4-bromophenyl)-1-oxo-3-phenyl-1-(pyrrolidin-1-yl)propan-2-yl)-4-methoxybenzamide

^{1}H NMR (600 MHz, CDCl$_3$) δ 8.45 (s, 1H), 7.67 (d, *J* = 8.7 Hz, 2H), 7.50 (d, *J* = 8.6 Hz, 2H), 7.42 (d, *J* = 8.1 Hz, 2H), 7.20 (t, *J* = 6.2 Hz, 3H), 7.00 – 6.96 (m, 2H), 6.86 (d, *J* = 8.8 Hz, 2H), 4.53 (d, *J* = 13.3 Hz, 1H), 3.81 (s, 3H), 3.60 – 3.52 (m, 2H), 3.49 – 3.40 (m, 2H), 2.65 – 2.58 (m, 1H), 1.88 – 1.81 (m, 2H), 1.78 – 1.67 (m, 2H).

^{13}C NMR (151 MHz, CDCl$_3$) δ 167.79, 163.53, 161.08, 138.53, 134.70, 130.55, 128.82, 127.87, 127.73, 127.18, 126.22, 126.11, 120.76, 112.57, 64.48, 54.34, 47.30, 46.24, 33.59, 25.71, 22.01.

HRMS (ESI) calcd for $C_{27}H_{28}{}^{79}BrN_2O_3$ $(M+H)^+$: 507.1278, found: 507.1280; calcd for $C_{27}H_{28}{}^{81}BrN_2O_3$ $(M+H)^+$: 509.1257, found: 509.1260.

Supplementary Discussion

An oven-dried 10-mL Schlenk tube equipped with a stirrer was charged with the oxazolone **1** (0.1 mmol, 1.0 equiv) and the phthalimide (0.2 mmol, 2.0 equiv). Then, 1.0 mL solvent (0.1 M) was added followed by the alkane **2** (0.5 mmol, 5 equiv) in glove box. The tube was sealed with a screw cap and took out from glove box. The reaction mixture was inserted into the PhotoSyn 3.0 reactor and irradiated using a 10 W LED lamp (395 nm) for 12 h. The reaction mixture was concentrated in vacuo and purified by flash column chromatography (petroleum ether/EA = 20/1).

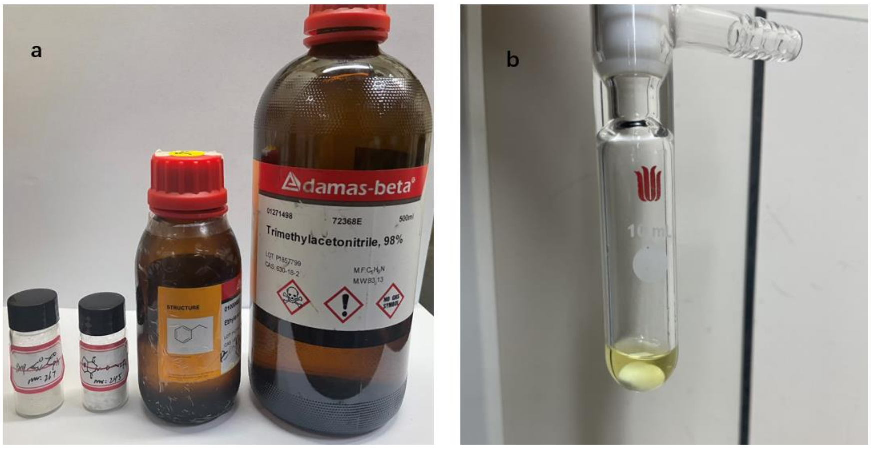

Supplementary Figure 3 a) Image of standard reagents; b) Image of reaction mixture before light

Supplementary Table 1: Evaluation of different solvents

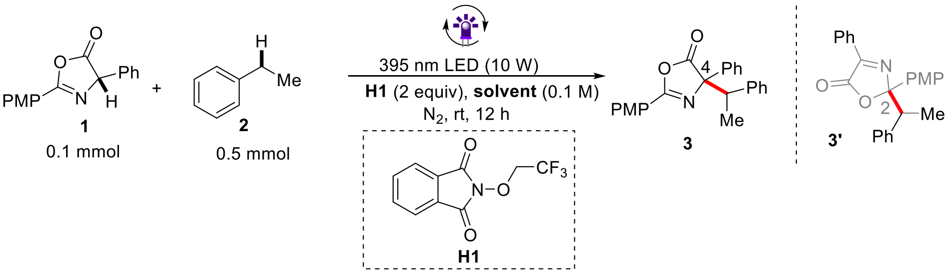

Entry	Solvents (0.1 M)	Yield (%)
1	$PhCF_3$	trace
2	CH_2Cl_2	trace
3	DCE	trace
4	DMSO	n.d
5	DMF	n.d
6	DMA	n.d
7	HFIP	trace
8	MeCN	49 (4:1)
9	*t*-$BuCO_2Me$	48 (10:1)
10	*t*-BuCN	70 (18:1)
11	*t*-BuCN:DMF = 1:1	n.d
12	CCl_3CN	trace

Isolated yield. The regioselectivity of **3**/**3'** in parentheses was determined by ^{1}H NMR. n.d = not detected by TLC.

Supplementary Table 2: Evaluation of different phthalimides

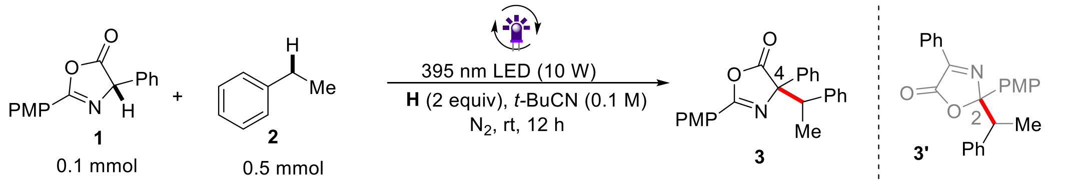

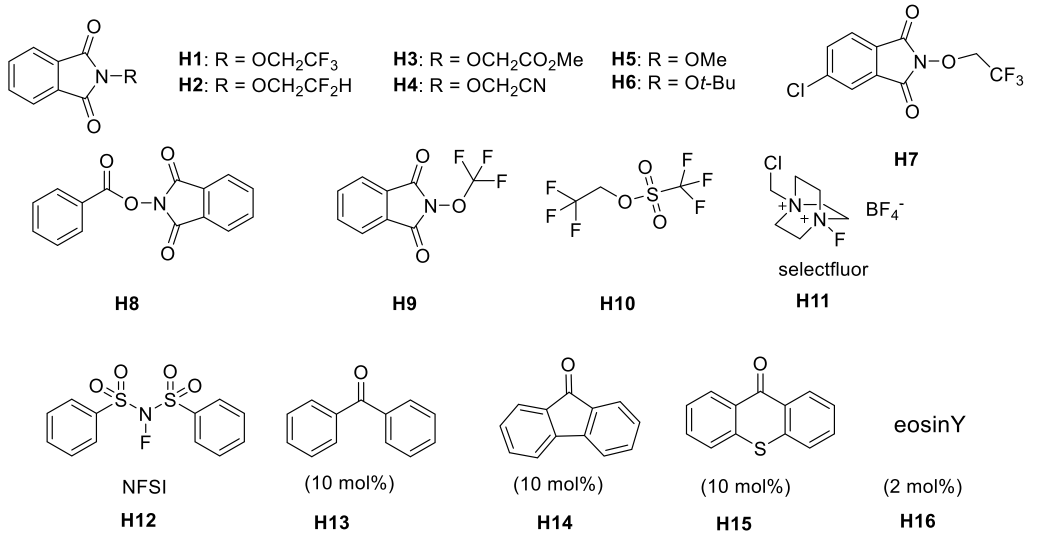

Entry	Phthalimides	Yield (%)
1	H1	70 (18:1)
2	H2	64 (11:1)
3	H3	55 (15:1)
4	H4	53 (15:1)
5	H5	trace
6	H6	trace
7	H7	49 (17:1)
8	H8	trace
9	H9	n.d
10	H10	n.d
11	H11	n.d
12	H12	n.d
13	H13	trace
14	H14	trace
15	H15	n.d
16	H16	trace

Isolated yield. The regioselectivity of **3/3'** in parentheses was determined by ^{1}H NMR. n.d = not detected by TLC.

Supplementary Table 3: Evaluation of different additivies

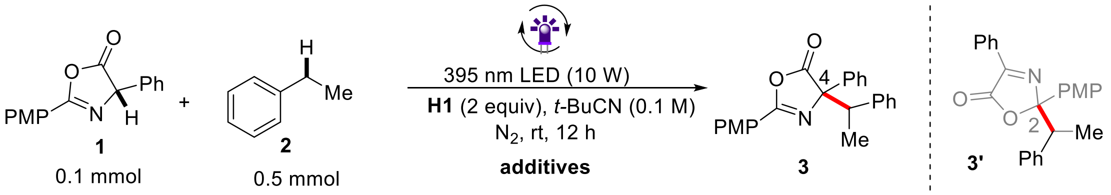

Entry	Additivies	Yield (%)
1	CF_3COOH (1 equiv)	62 (16:1)
2	BpinH (1 equiv)	54 (12:1)
3	Succinonitrile (5 equiv)	68 (18:1)
4	pivalic acid (1 equiv)	39
5	AgOTf (1 equiv)	31
6	Al_2O_3 (50 mg)	35
7	$FeCl_3$ (10% mol)	36
8	^{n}Bu_4NCl (10% mol)	trace
9	DMAP (1 equiv)	trace
10	Cs_2CO_3 (1 equiv)	trace

Isolated yield. The regioselectivity of **3/3'** in parentheses was determined by ^{1}H NMR. n.d = not detected by TLC.

Supplementary Table 4: Control experiment

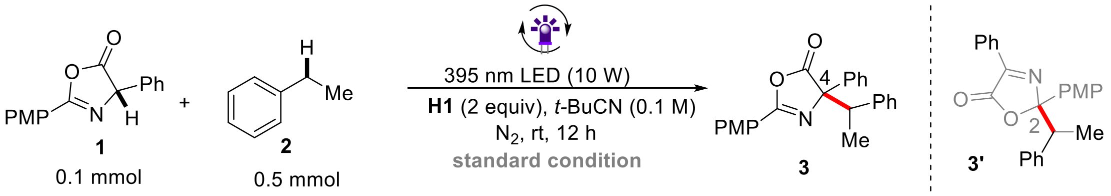

Entry	Deviation from standard conditions	Yield (%)
1	None	70 (18:1)
2	1 equiv **H1** instead of 2 equiv **H1**	44 (18:1)
3	40 °C instead of 25~30 °C	62 (17:1)
4	0.05 M instead of 0.1 M	62 (18:1)
5	0.2 M instead of 0.1 M	66 (18:1)
6	380 nm instead of 395 nm	58 (18:1)
7	420 nm instead of 395 nm	68 (4:1)
8	10 equiv **2** instead of 5 equiv **2**	71 (17:1)
9	4 equiv **2** instead of 5 equiv **2**	63(18:1)
10	In air	n.d
11	No **H1**	n.d
12	No Light	n.d
13	No Light, 120 °C	n.d

Isolated yield. The regioselectivity of **3/3'** in parentheses was determined by ^{1}H NMR. n.d = not detected by TLC.

Supplementary Table 5: Radical initiator initiates the reaction

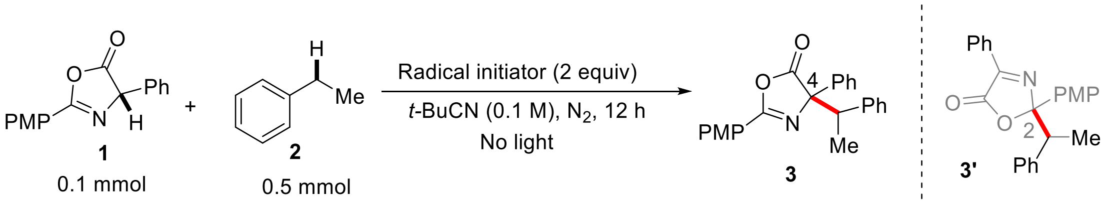

Entry	Additivies	Yield (%)
1	$(NH_4)_2S_2O_8$, 100 °C	n.d
2	AIBN, 100 °C	n.d
3	TBHP, 100 °C	trace
4	DTBP, 100 °C	trace
5	PBO, 100 °C	trace
6	*m*-CPBA, rt	trace
7	Selectfluor, rt	n.d
8	PIDA, rt	n.d

n.d = not detected by TLC.

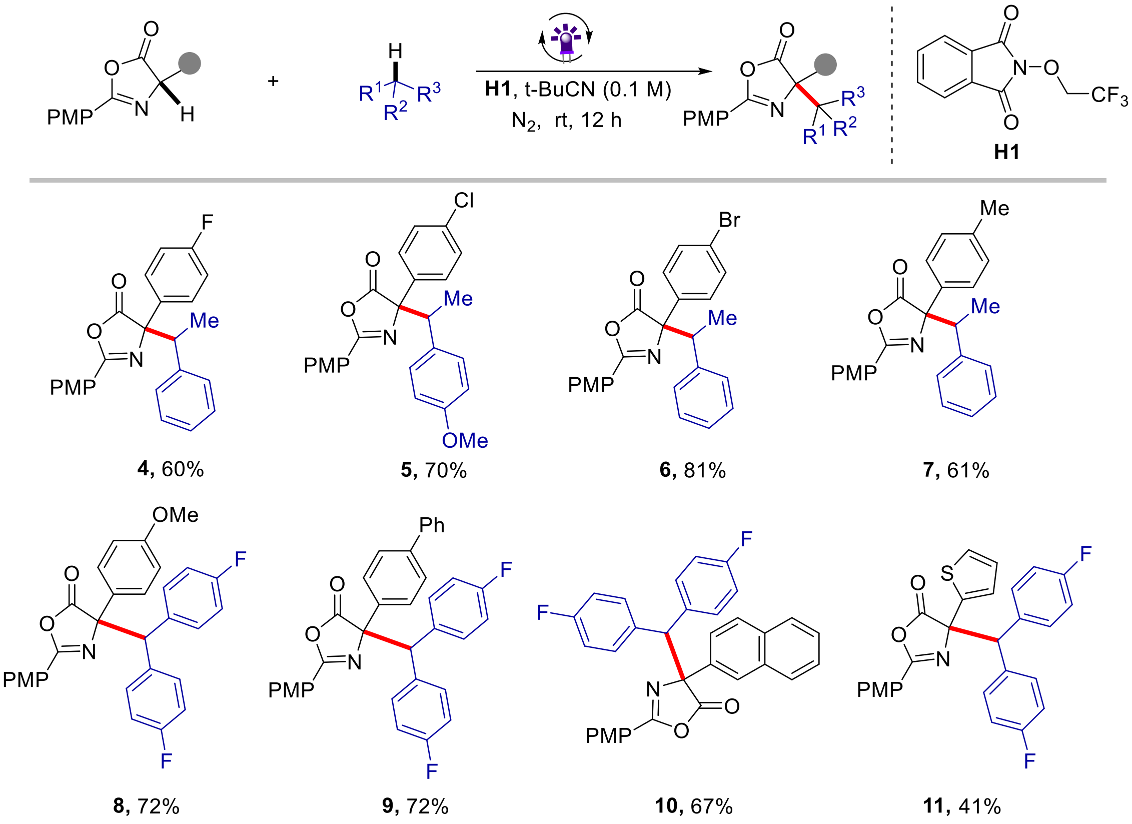

Supplementary Figure 4 Oxazolone substrates

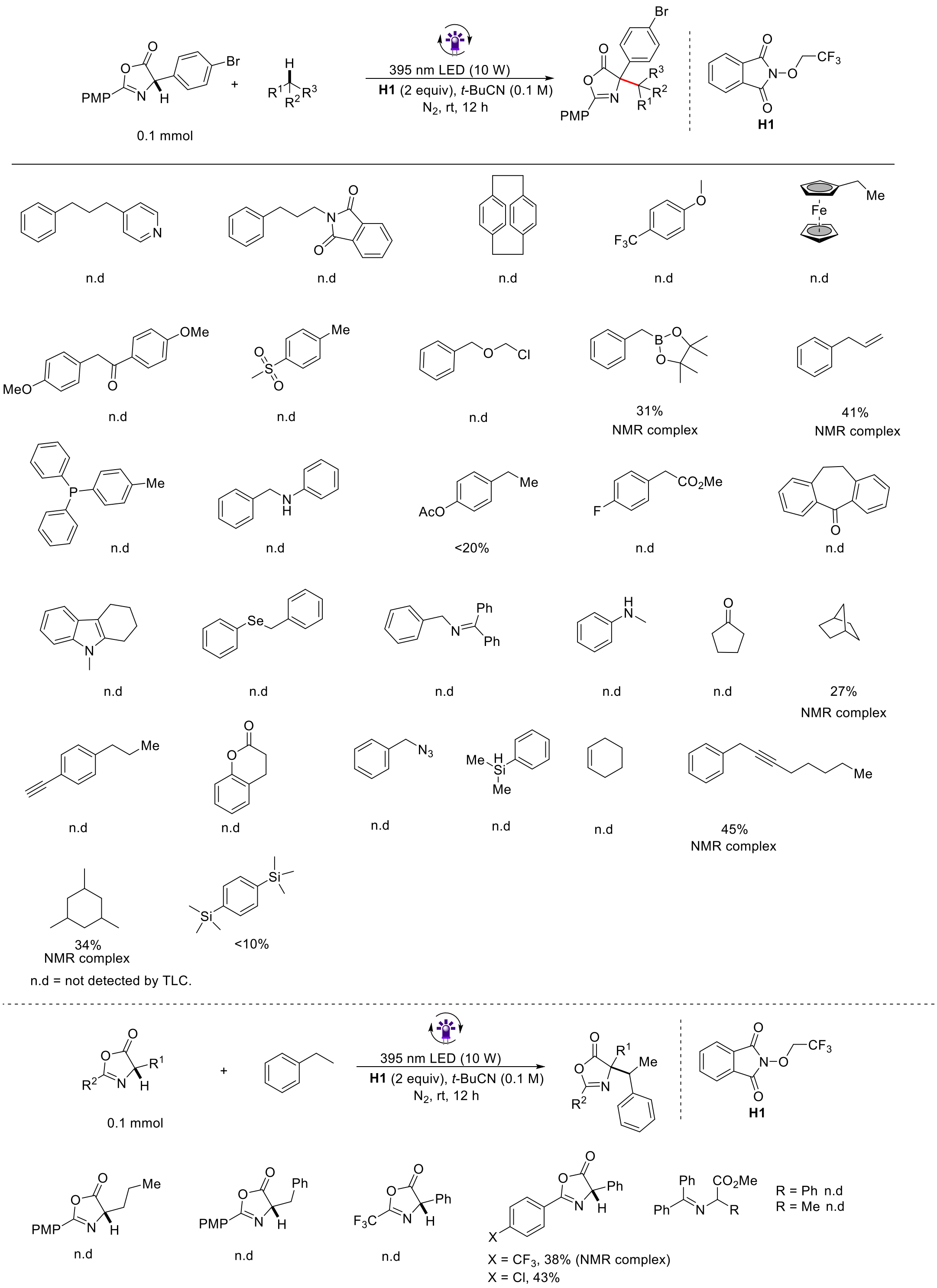

Supplementary Figure 5 Failed substrates

Examination of asymmetric version

We explored various chiral catalysts under standard reaction conditions. The C-H benzyl substrates **2a** with hydroxyl groups were employed as free radical precursors. The introduction of hydroxyl groups aimed to facilitate hydrogen bonding interactions with the chiral catalysts, potentially enhancing the enantioselectivity. Upon investigation, it was found that utilizing chiral phosphoric acid **L8** as the catalyst led to the formation of product

85 in 40% yield with 37% *ee*. chiral urea and chiral guanidine catalysts proved to be incompatible with this reaction (Figure 6).

4-(4-bromophenyl)-4-((4-((tert-butyldimethylsilyl)oxy)phenyl)(phenyl)methyl)-2-(4-methoxyphenyl)oxazol-5(4H)-one (85)

^{1}H NMR (600 MHz, $CDCl_3$) δ 7.94 –7.93 (m, 2H), 7.62 – 7.61 (m, 1H), 7.57 – 7.54 (m, 2H), 7.46 – 7.45 (m, 1H), 7.39 – 7.29 (m, 3H), 7.19 – 7.16 (m, 2H), 7.13 – 7.12 (m, 2H), 6.99 – 6.94 (m, 2H), 6.66 – 6.64 (m, 1H), 6.59 – 6.57 (m, 1H), 4.86 – 4.83 (m, 1H), 3.89 – 3.87 (m, 3H), 0.90 – 0.88 (m, 9H), 0.14 – 0.11 (m, 3H), 0.01 – -0.02 (m, 3H). **^{13}C NMR (151 MHz, $CDCl_3$)** δ 177.46, 177.38, 162.27, 159.24, 153.83, 153.28, 138.01, 137.20, 135.49, 135.39, 130.45, 130.34, 130.26, 129.82, 129.76, 129.63, 128.93, 128.88, 128.73, 128.64, 127.13, 127.06, 126.30, 125.61, 121.99, 121.08, 121.03, 118.70, 116.82, 113.15, 113.13, 77.04, 76.99, 59.46, 59.10, 54.49, 54.45, 25.27, 24.61, 24.57, 17.15, 17.10, -5.48, -5.62, -5.67.

HRMS (ESI) calcd for $C_{35}H_{37}{}^{79}BrNO_4Si$ $(M+H)^+$:642.1670, found: 642.1672; calcd for $C_{35}H_{37}{}^{81}BrNO_4Si$ $(M+H)^+$: 644.1649, found: 644.1648.

(CHIRALPAK IA column, hexane/*i*-PrOH, 80:20 v/v, flow rate 1.0 mL/min, λ = 254 nm, 37 °C), tR (major) = 4.31 min, tR (minor) = 9.26 min.

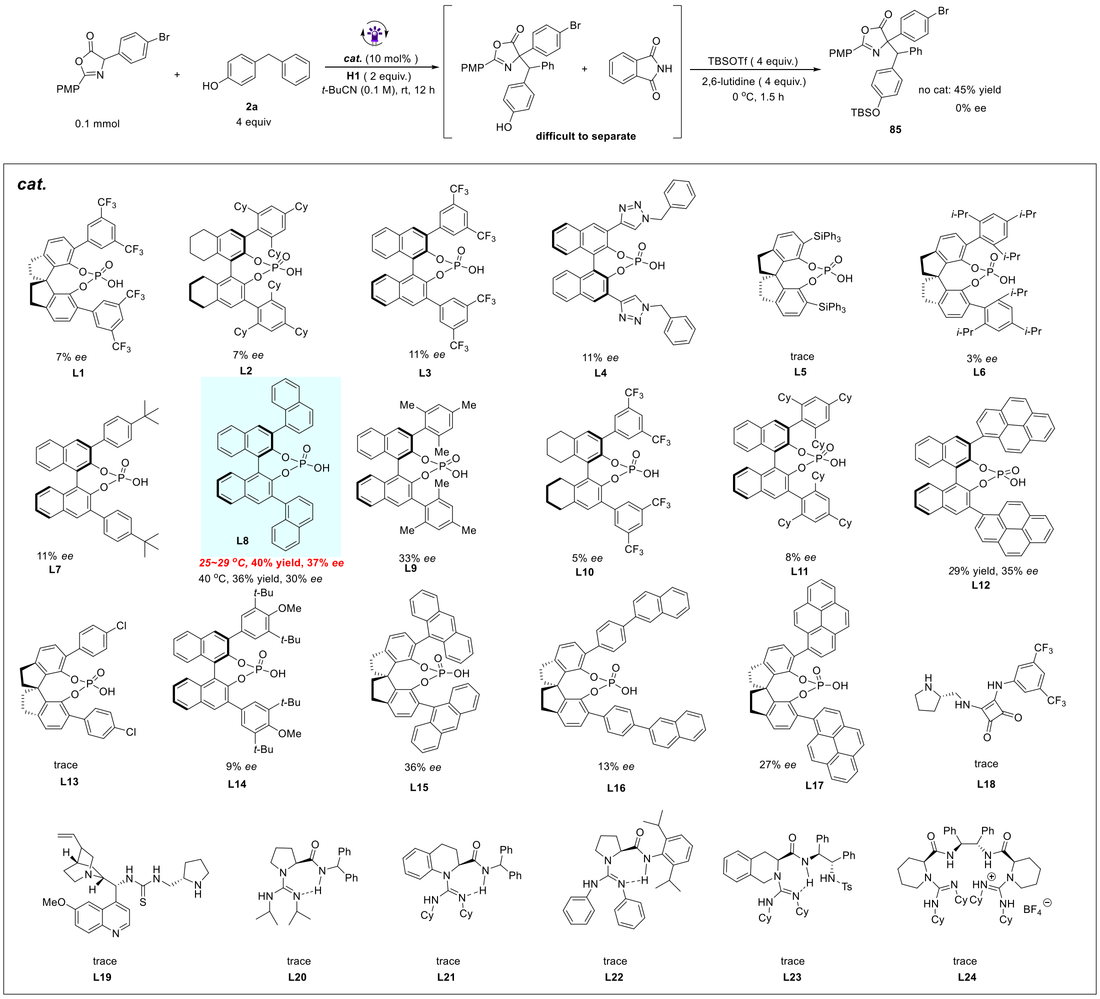

Supplementary Figure 6 Explore different chiral catalysts

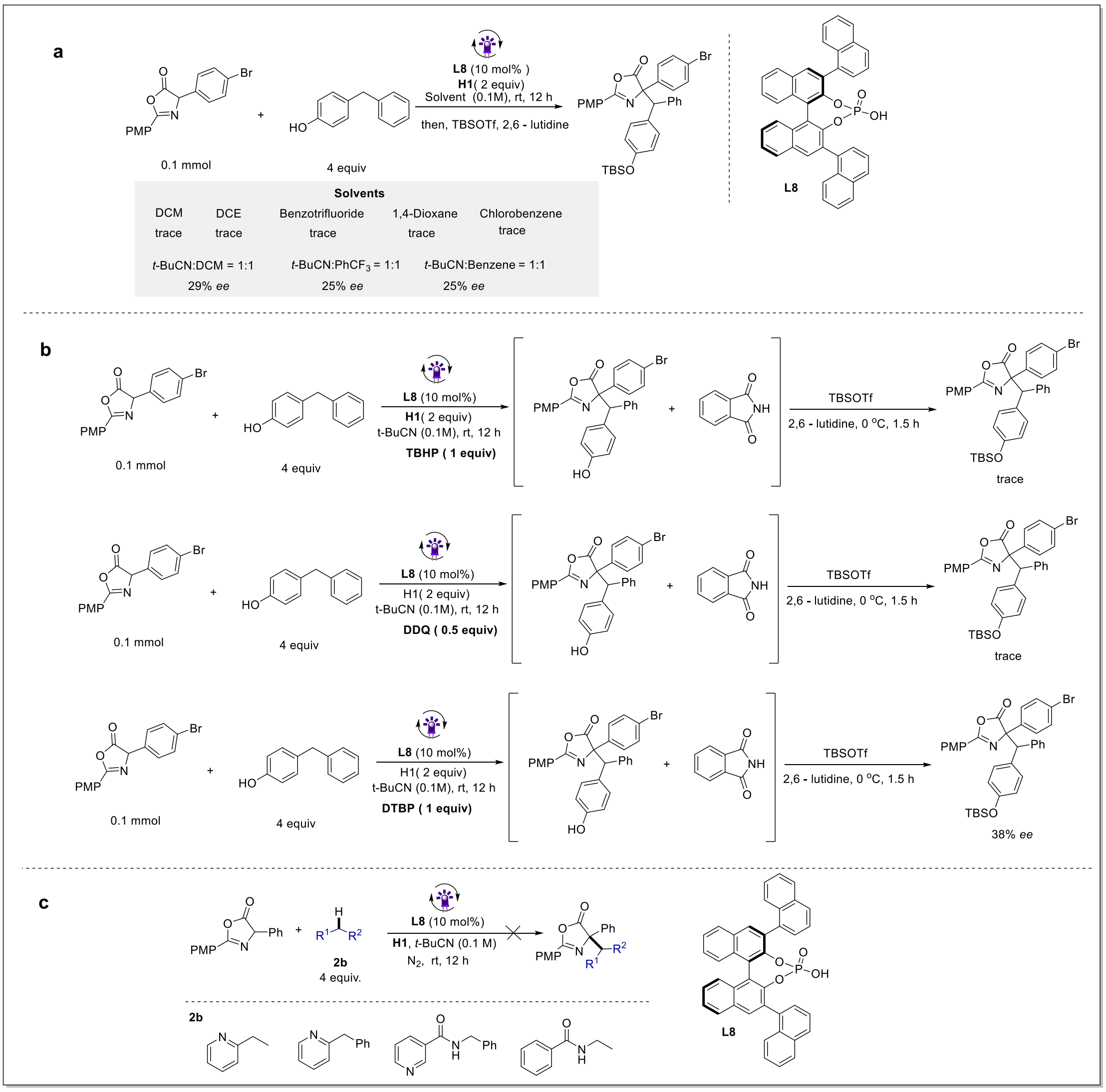

Supplementary Figure 7 Explore different reaction conditions

Despite exploring alternative reaction conditions, including different solvents, chemical oxidants, etc., no significant improvement in enantioselectivity was achieved (Figure 7a & 7b). The C-H benzyl substrates **2b** with pyridine or amide structures exhibited incompatibility with our system (Figure 7c).

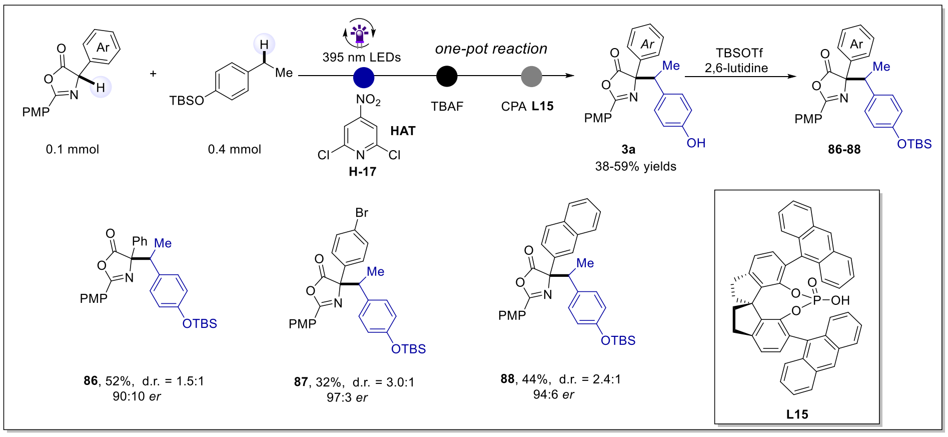

Supplementary Figure 8 multi-step synthesis strategy

Inspired by the relevant literature (*J. Am. Chem. Soc.***2023**, 145, 2794–2799; *Chem. Asian J.* **2018**, 13, 2440–2444), we pursued an alternative approach to attain asymmetric synthesis through multi-step reactions in a one-pot fashion. To our delight, employing chiral phosphoric acid **L15** as the catalyst under adjusted reaction conditions enabled the successful synthesis of the target CDC products (**86-88**) in moderate yields with excellent stereoselectivities (up to 97:3 er). Due to challenges in effectively separating the enantiomers of product **3a** *via* HPLC, we directly synthesized products **86-88** using a one-step method (Figure 8).

Step1: An oven-dried 10-mL Schlenk tube equipped with a stirrer was charged with the 2,6-dichloro-4-nitropyridine (0.2 mmol, 1.0 equiv.) and the C-H substrate (0.4 mmol, 2.0 equiv). Then, 2.0 mL (DCM:HFIP = 1:1) was added in glove box. The tube was sealed with a screw cap and took out from glove box. The reaction mixture was inserted into the PhotoSyn 3.0 reactor and irradiated using a 10 W LED lamp (395 nm) for 14 h. The reaction mixture was concentrated in vacuo and purified by flash column chromatography (petroleum ether/EA = 10/1).

Step2: benzyl alcohol was prepared from step1. Dissolve benzyl alcohol in 1.0 mL THF, Then, TBAF (1 M in THF, 1.2 equiv) was added in 0 °C for 30 min. The reaction mixture was concentrated in vacuo, the mixture was diluted with EA (15 mL), then extracted with saturated NH_4Cl aqueous solution for three times. The combined organic layers were dried with Na_2SO_4, filtered, and concentrated in vacuo, which was used in the next step without further purification (**3a**).

Step3 and **4**: An oven-dried 10-mL Schlenk tube equipped with a stirrer with Step 2 of the crude product, oxazolone (0.1 mmol), **L15** (10mol%) and 5Å MS (50 mg). Then, 4.0 mL DCE was added in glove box. The tube was sealed with a screw cap and took out from glove box. The reaction mixture was stirred at 0 °C for 12h. After consumption of starting materials, 2,6-lutidine (0.4 mmol, 4 equiv) and TBSOTf (0.4 mmol, 4 equiv.) were added subsequently. The mixture was stirred at 0 °C for 1.5 h. Next, the mixture was filtered through celite, and the filtrate was concentrated. The residue was purified by silica gel chromatography to give the desired product.

1-(4-((tert-butyldimethylsilyl)oxy)phenyl)ethan-1-ol

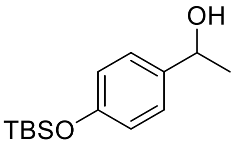

^{1}H NMR (400 MHz, CDCl$_3$) δ 7.25 (d, J = 8.5 Hz, 2H), 6.88 –6.79 (m, 2H), 4.86 (q, J = 6.4 Hz, 1H), 1.84 (s, 1H), 1.48 (d, J = 8.9 Hz, 3H), 1.02 (s, 9H), 0.22 (s, 6H).

^{13}C NMR (101 MHz, CDCl$_3$) δ 155.03, 138.53, 126.58, 119.99, 70.06, 25.69, 24.97, 18.21, -4.41.

4-(1-(4-((tert-butyldimethylsilyl)oxy)phenyl)ethyl)-2-(4-methoxyphenyl)-4-phenyloxazol-5(4H)-one (86)

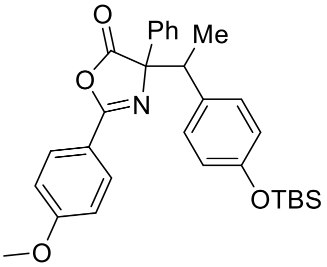

^{1}H NMR (400 MHz, CDCl$_3$) δ 8.03 – 8.01 (m, 1H), 7.91 – 7.83 (m, 2H), 7.61 – 7.58 (m, 1H), 7.45 – 7.34 (m, 2H), 7.21 – 7.16 (m, 1H), 7.16 – 7.12 (m, 1H), 7.03 – 6.96 (m, 3H), 6.67 – 6.61 (m, 2H), 3.90 – 3.89 (m, 3H), 3.53 – 3.69 (m, 1H), 1.33 – 1.27 (m, 3H), 0.97 – 0.94 (m, 9H), 0.11 – 0.08 (m, 6H).

^{13}C NMR (101 MHz, CDCl$_3$) δ 178.93, 178.73, 163.15, 163.06, 159.70, 159.39, 154.85, 154.43, 137.88, 137.72, 132.89, 132.33, 130.01, 129.96, 129.90, 129.82, 128.52, 128.08, 128.03, 127.65, 126.28, 126.18, 119.57, 119.28, 118.33, 114.15, 114.07, 78.35, 78.08, 55.51, 55.48, 49.64, 49.31, 25.70, 25.67, 18.22, 18.20, 16.16, 15.53, 1.04, -4.48, -4.51, -4.55.

HRMS (ESI) calcd for $C_{30}H_{36}NO_4Si$ $(M+H)^+$: 502.2408, found:502.2409.

(major) er = 90:10 (CHIRALPAK AD-H column; hexane/*i*-PrOH, 98:2 v/v, flow rate 0.9 mL/min, λ = 254 nm, 37 °C), tR (major) = 6.03 min, tR (minor) = 21.04 min.

4-(4-bromophenyl)-4-(1-(4-((tert-butyldimethylsilyl)oxy)phenyl)ethyl)-2-(4-methoxyphenyl)oxazol-5(4H)-one (87)

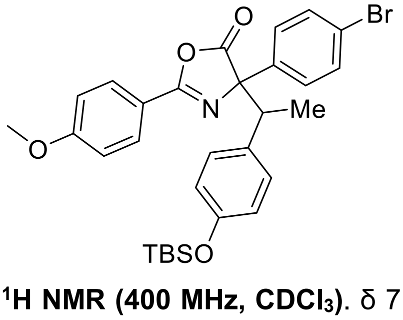

^{1}H NMR (400 MHz, CDCl$_3$). δ 7.89 – 7.87 (m, 2H), 7.73 – 7.71 (m, 2H), 7.54 – 7.56 (m, 2H) , 7.12 – 7.09 (m, 2H), 6.97 – 6.95 (m, 2H), 6.65 – 6.63 (m, 2H), 3.89 (s, 3H), 3.62 – 3.56 (m, 1H), 1.26 (d, J = 8.0 Hz, 3H), 0.91 (s, 9H), 0.08 – 0.76 (m, 6H).

^{13}C NMR (101 MHz, CDCl$_3$) δ 178.36, 163.18, 159.95, 154.96, 136.96, 131.95, 131.61, 131.14, 129.96, 129.85, 128.10, 127.94, 122.29, 119.61, 119.41, 118.10, 114.22, 114.11, 77.98, 55.49, 49.40, 25.70, 25.66, 18.19, 16.25, 15.45, 1.03, -4.53, -4.56.

HRMS (ESI) calcd for $C_{30}H_{35}{}^{79}BrNO_4Si$ $(M+H)^+$: 580.1513, found: 580.1512; calcd for $C_{30}H_{35}{}^{81}BrNO_4Si$ $(M+H)^+$: 582.1493, found: 582.1490.

(major) er = 97:3 (CHIRALPAK AD-H column; hexane/*i*-PrOH, 97:3 v/v, flow rate 0.9 mL/min, λ = 254 nm, 37 °C), tR (major) = 5.89 min, tR (minor) = 22.60 min.

4-(1-(4-((tert-butyldimethylsilyl)oxy)phenyl)ethyl)-2-(4-methoxyphenyl)-4-(naphthalen-2-yl)oxazol-5(4H)-one (88)

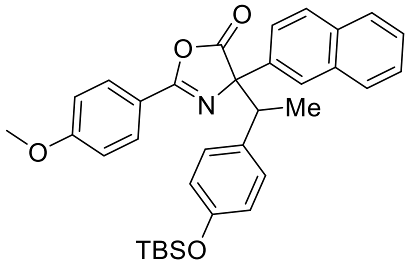

^{1}H NMR (400 MHz, CDCl$_3$) δ 8.37– 8.24 (m, 1H), 8.08 – 7.77 (m, 6H), 7.58 – 7.40 (m, 2H), 7.24 – 6.92 (m, 4H), 6.74 – 6.53 (m, 2H), 3.90 – 3.87 (m, 3H), 3.82 – 3.65 (m, 1H), 1.37– 1.28 (m, 3H), 0.93 – 0.90 (m, 9H), 0.11 – 0.05 (m, 6H).

^{13}C NMR (101 MHz, CDCl$_3$) δ 178.67, 163.09, 159.83, 154.88, 135.23, 133.11, 132.95, 132.30, 130.03, 129.93, 129.87, 128.33, 127.82, 127.60, 127.43, 126.30, 126.25, 125.72, 124.06, 123.72, 119.59, 119.31, 118.36, 114.15, 114.09, 78.51, 55.49, 49.45, 49.18, 25.67, 25.44, 18.19, 16.17, 15.59, 1.03, -4.52, -4.55.

HRMS (ESI) calcd for $C_{34}H_{38}NO_4Si$ $(M+H)^+$: 552.2565, found: 552.2561.

(major) er = 94:6 (CHIRALPAK AD-H column; hexane/*i*-PrOH, 97:3 v/v, flow rate 0.9 mL/min, λ = 254 nm, 37 °C), tR (major) = 8.20 min, tR (minor) = 25.43 min.

Supplementry Notes

Mechanistic Studies

Rradical trapping experiment

Reaction in the presence of TEMPO and BHT as the radical scavengers

To verify radical mechanism of this transformation, the radical trapping experiment was carried out as shown in below Figure 9. When 3 equiv TEMPO or BHT were added to this system, no cross-coupling product **3** was detected. Meanwhile, the radical trap product **89** was also detected by high-resolution mass spectrometry (HRMS).

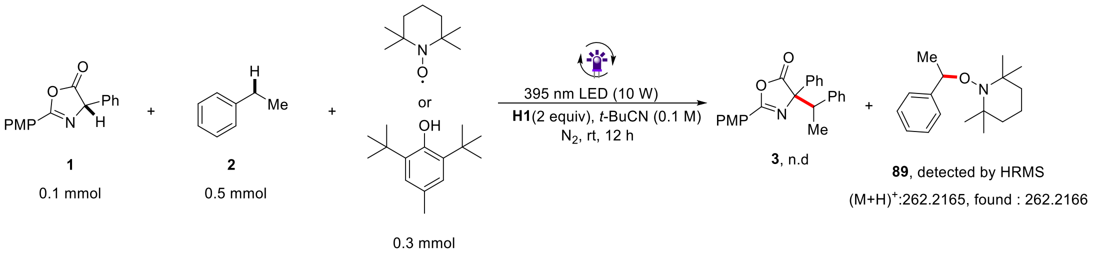

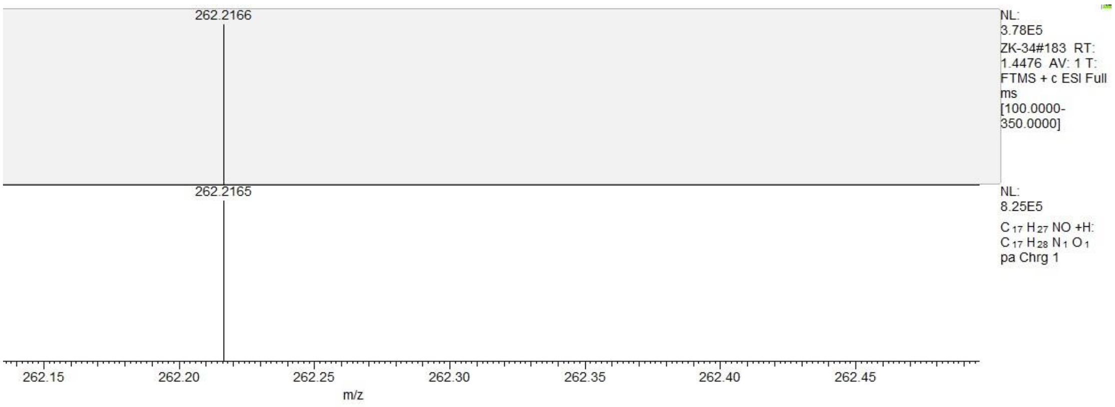

Supplementary Figure 9 High resolution mass spectrometry of intermediate **89**

Radical-trap experiment using air

An oven-dried 10-mL Schlenk tube equipped with a stirrer was charged with the oxazolone**1** (0.1 mmol, 1.0 equiv.) and the phthalimide **H1** (0.2 mmol, 2.0 equiv). Then, 1.0 mL *t*-BuCN (0.1 M) was added followed by the alkane **2** (0.5 mmol, 5 equiv) in air. The mixture was stirred at room temperature under an air atmosphere irradiated using a 10 W LED lamp (395 nm) for 12 h. TLC analysis revealed that no product **3**. Acetophenone and 1-phenylethan-1-ol were detected in this reaction by GC-MS.

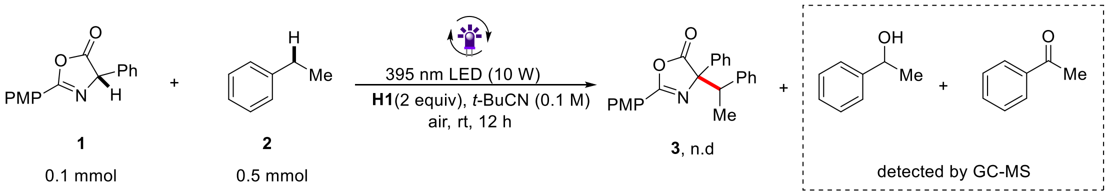

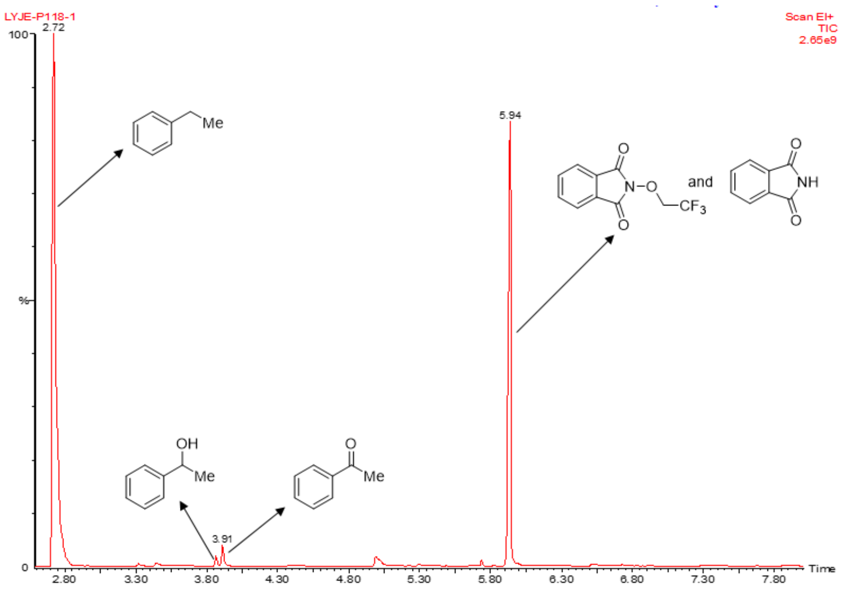

Supplementary Figure 10 GC-MS for the mixture in air

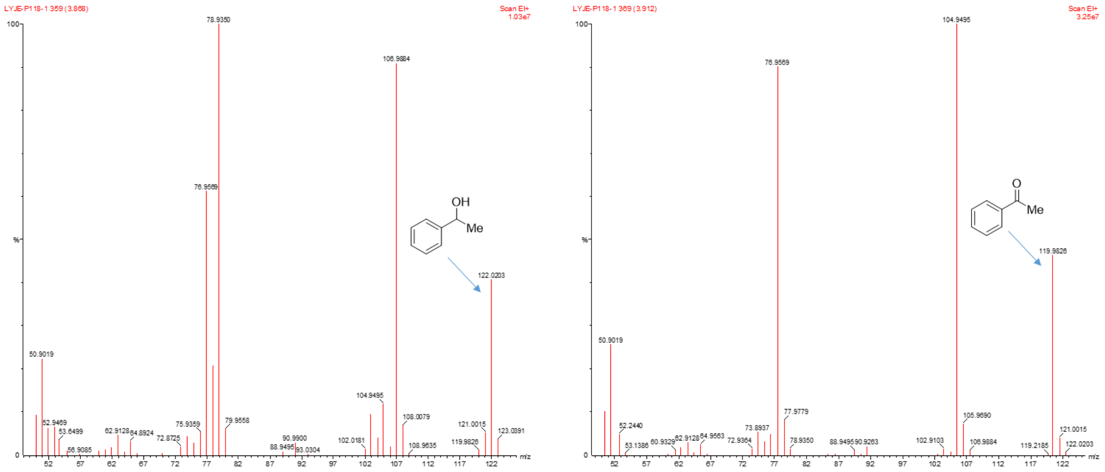

Supplementary Figure 11 GC-MS for acetophenone and 1-phenylethan-1-ol

Homocoupling dimer trapping experiment

An oven-dried 10-mL Schlenk tube equipped with a stirrer was charged with the oxazolone**1** (0.1 mmol, 1.0 equiv.) and the phthalimide **H1** (0.2 mmol, 2.0 equiv). Then, 1.0 mL *t*-BuCN (0.1 M) was added in glove box. The tube was sealed with a screw cap and took out from glove box. The reaction mixture was inserted into the PhotoSyn 3.0 reactor and irradiated using a 10 W LED lamp (395 nm) for 12 h. The reaction mixture was concentrated in vacuo. The crude product was analyzed by crude ^{1}H NMR based on a CH_2Br_2 standard. The self-coupling product in 48% NMR yield, detected in this reaction by LC-MS.

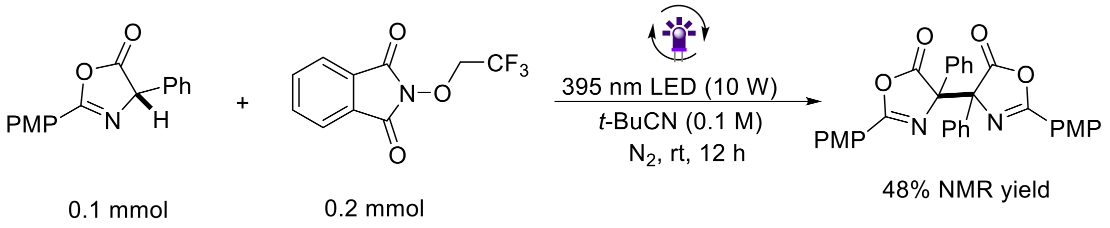

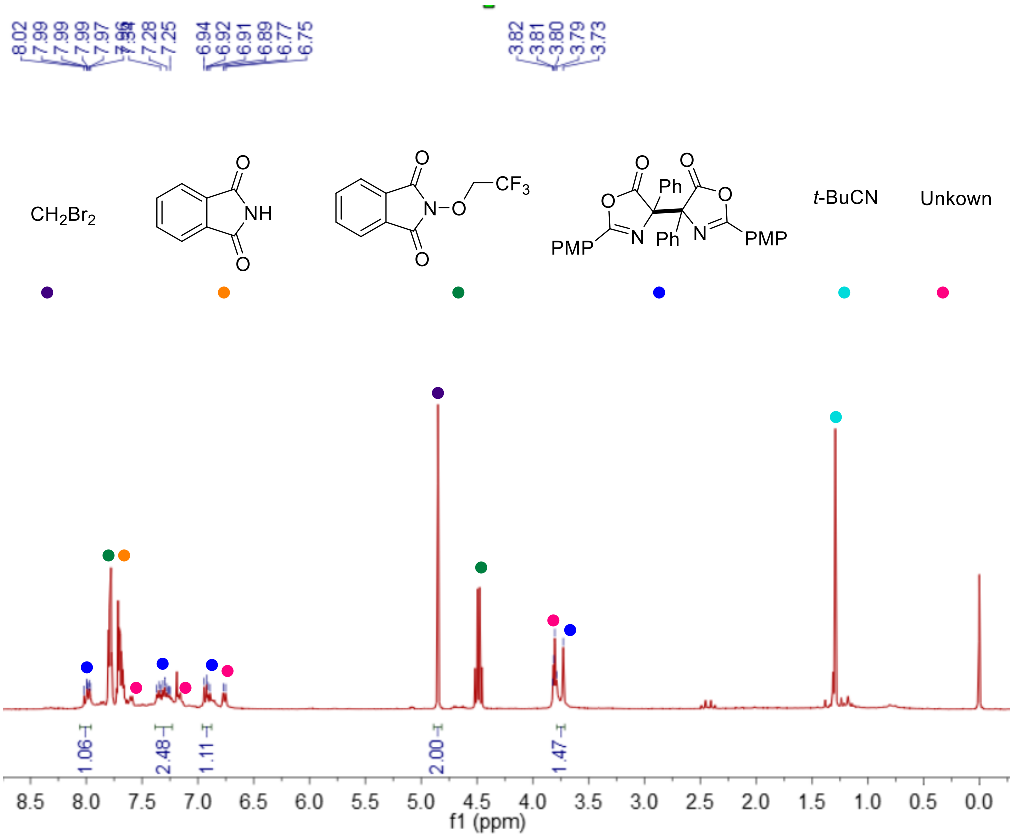

Supplementary Figure 12 The ^{1}H NMR spectrum of crude product

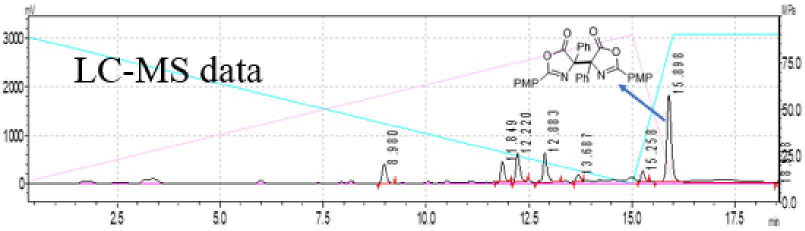

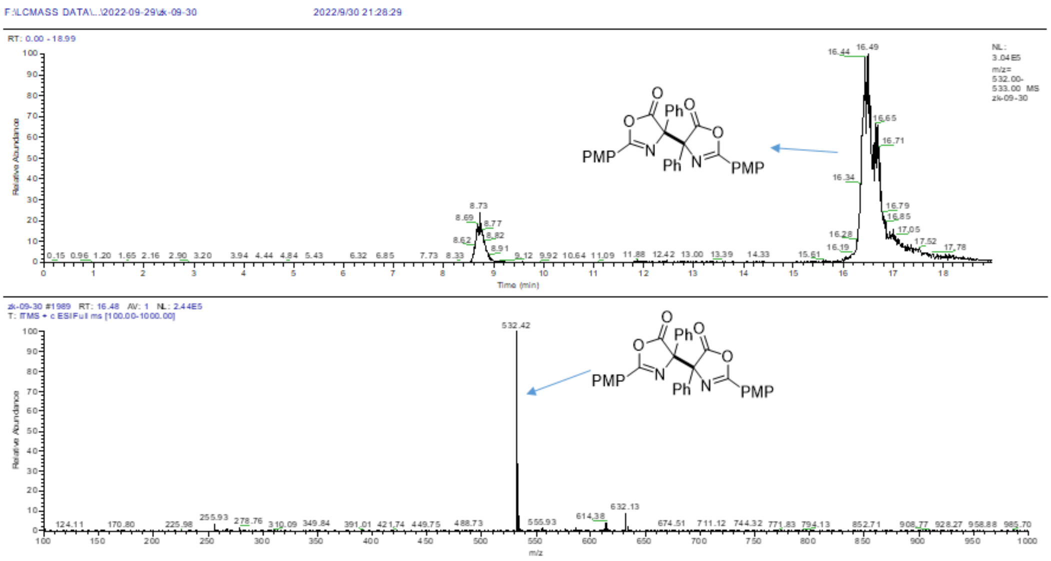

Supplementary Figure 13 The LC-MS data of self-coupling product

^{19}F NMR Studies

A: An oven-dried 10-mL Schlenk tube equipped with a stirrer was charged with the **H1** (0.05 mmol), Then, the CD_3CN (1.0 mL) was added followed by the 2,2,2-trifluoroethanol (0.1 mmol) in glove box. When the solid in the tube was completely dissolved (about 30s) by stirring. The reaction mixture (0.5 mL) was removed for ^{19}FNMR analysis.

B: An oven-dried 10-mL Schlenk tube equipped with a stirrer was charged with the **1** (0.1 mmol) and the **H1** (0.2 mmol). Then, the CD_3CN (1.0 mL) was added followed **2** (0.5 mmol) in glove box. When the solid in the tube was completely dissolved (about 30s) by stirring. The reaction mixture (0.5 mL) was removed for ^{19}FNMR analysis.

C: An oven-dried 10-mL Schlenk tube equipped with a stirrer was charged with the **1** (0.1 mmol) and the **H1** (0.2 mmol). Then, the CD_3CN (1.0 mL) was added followed **2** (0.5 mmol) in glove box. The tube was sealed with a screw cap and took out from glove box. The reaction mixture was inserted into the PhotoSyn 3.0 reactor and irradiated using a 10 W LED lamp (395 nm) for 12 h. The reaction mixture (0.5 mL) was removed for ^{19}FNMR analysis.

D: An oven-dried 10-mL Schlenk tube equipped with a stirrer was charged with the **1** (0.1 mmol) and the **H1** (0.2 mmol). Then, the *t*-BuCN (1.0 mL) was added followed **2** (0.5 mmol) in glove box. The tube was sealed with a screw cap and took out from glove box. The reaction mixture was inserted into the PhotoSyn 3.0 reactor and irradiated using a 10 W LED lamp (395 nm) for 12 h. The reaction mixture (0.1 mL) was removed in CD_3CN for ^{19}FNMR analysis.

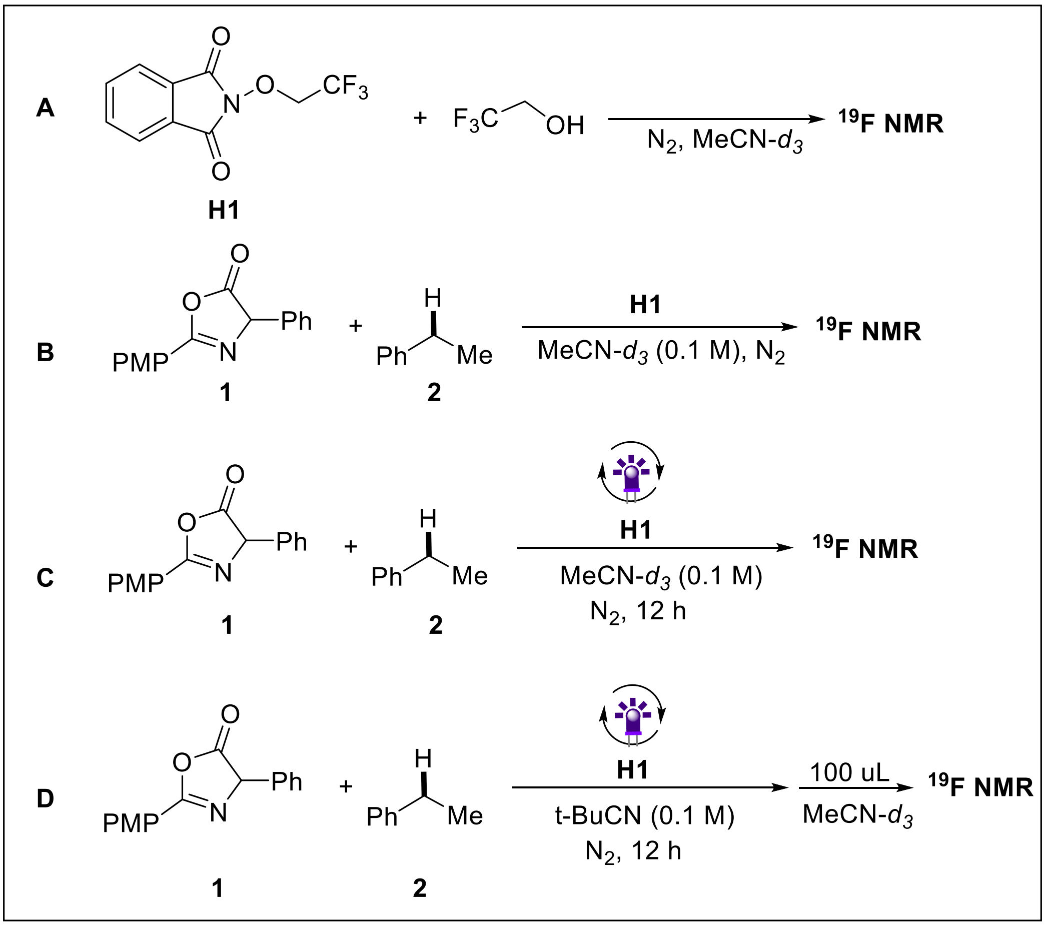

Supplementary Figure 14 ^{19}F NMR Studies

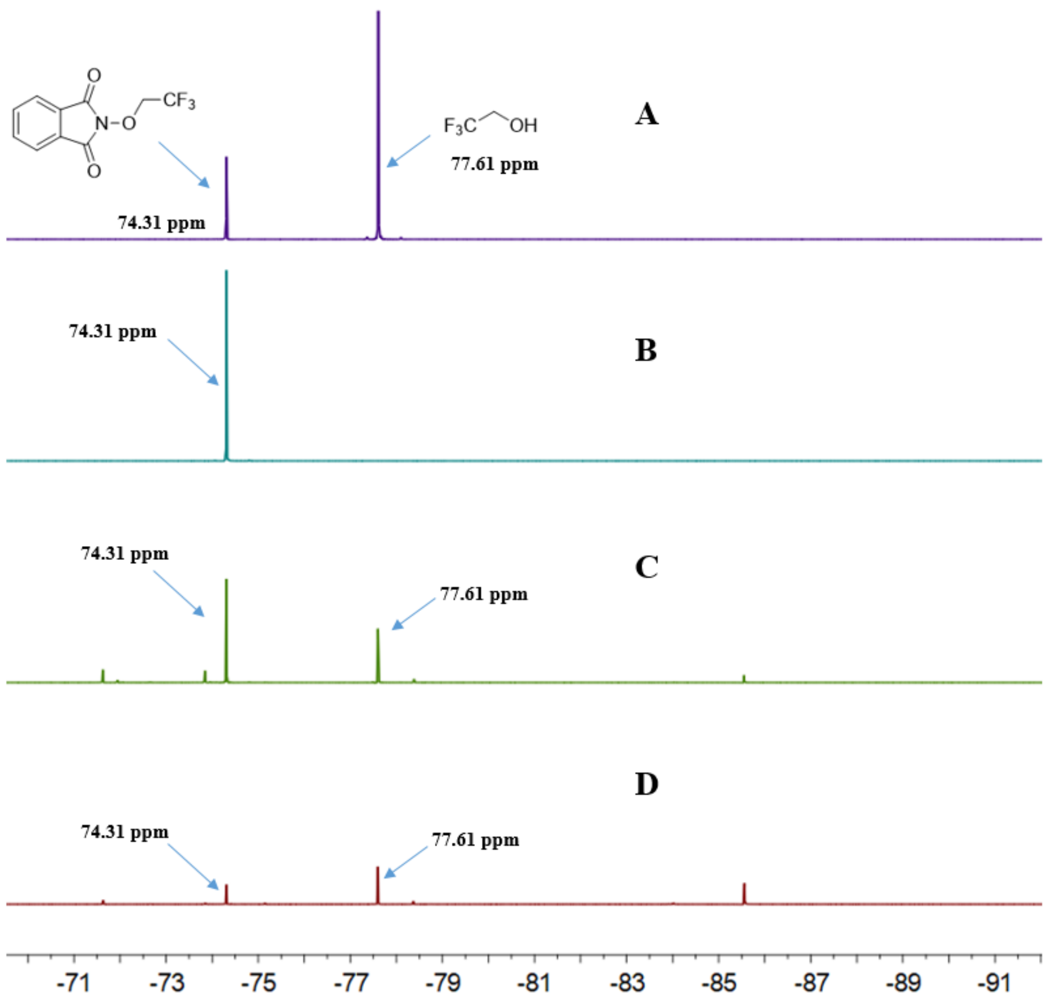

Supplementary Figure 15 ^{19}F NMR spectrum

For those process, the substrate**1** and phthalimide **H1** don't form complexes in CD_3CN, because ^{19}F chemical shift changes are not seen in the mixed fluorine spectrum (Figure 15A and B**)**. *In situ* NMR studies revealed the evolution of 2,2,2-trifluoroethanol as the reaction proceeded, indicating that trifluoroethoxy radical indeed facilitates HAT from the substrate (Figure 15C and D**)**.

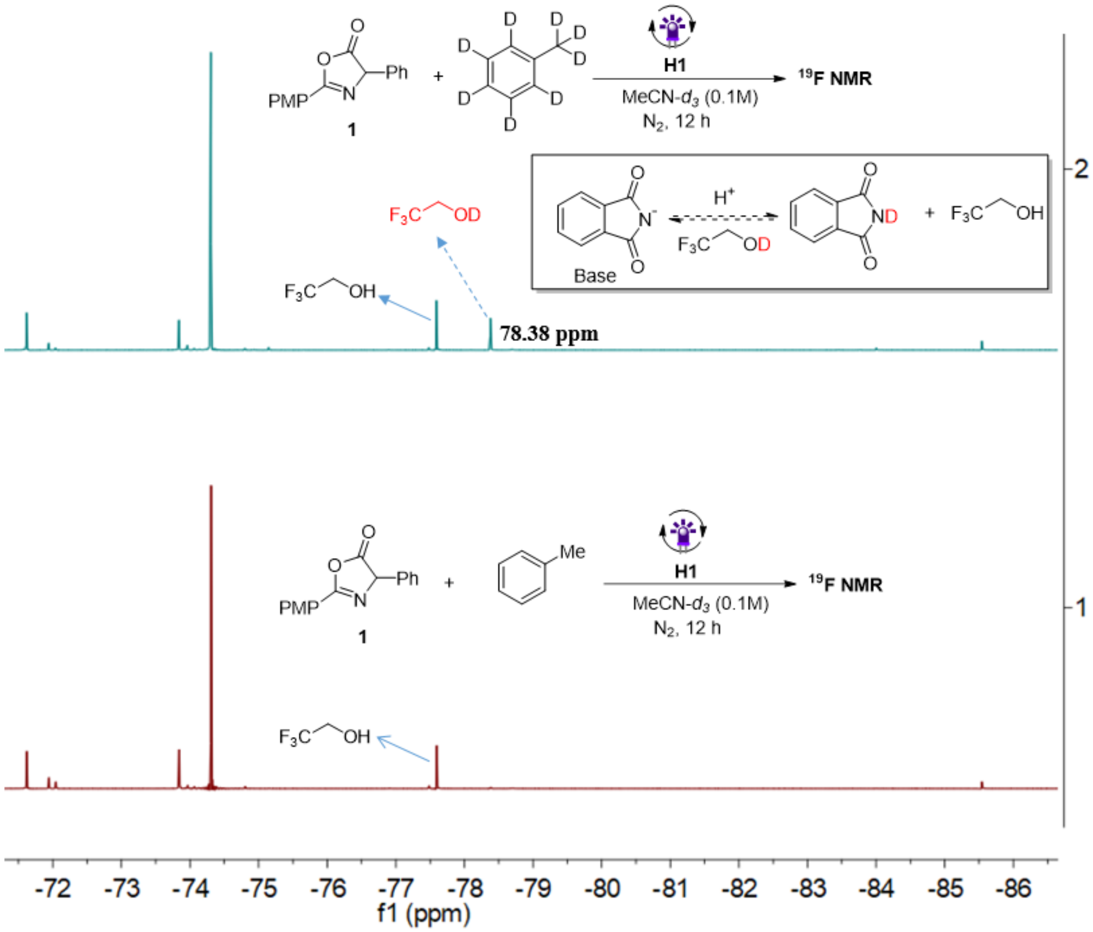

Supplementary Figure 16 ^{19}F NMR spectrum

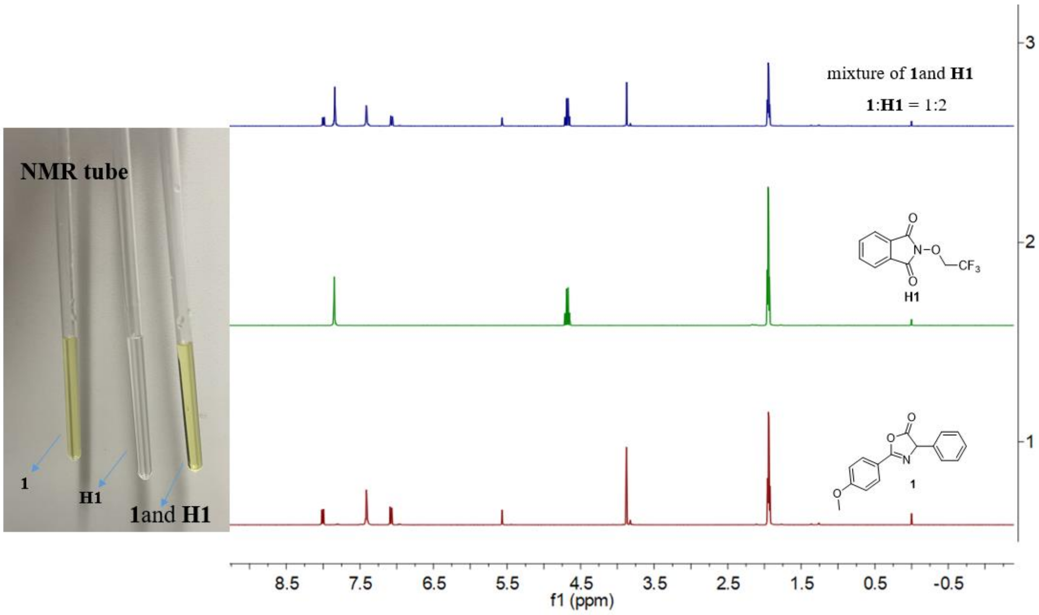

Supplementary Figure 17 ^{1}H NMR spectrum of **1** and **H1**

Trifluoroethoxy radical trapping experiment

An oven-dried 10-mL Schlenk tube equipped with a stirrer was charged with the oxazolone **1** (0.1 mmol, 1.0 equiv.) and the phthalimide **H1** (0.2 mmol, 2.0 equiv). Then, 1.0 mL *t*-BuCN (0.1 M) was added followed by the prop-1-en-2-ylbenzene **90** (0.2 mmol, 2 equiv) in glove box. The tube was sealed with a screw cap and took out from glove box. The reaction mixture was inserted into the PhotoSyn 3.0 reactor and irradiated using a 10 W LED lamp (395 nm) for 12 h. The reaction mixture was concentrated in vacuo. Purification of the crude mixture by flash column chromatography on silica gel (petroleum ether/ethyl acetate 20:1, 31.3 mg, 65%) provided adduct **91**.

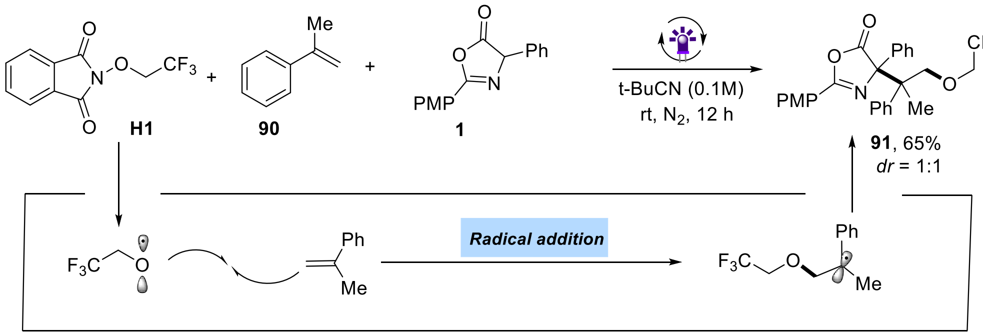

UV/Vis Spectroscopy

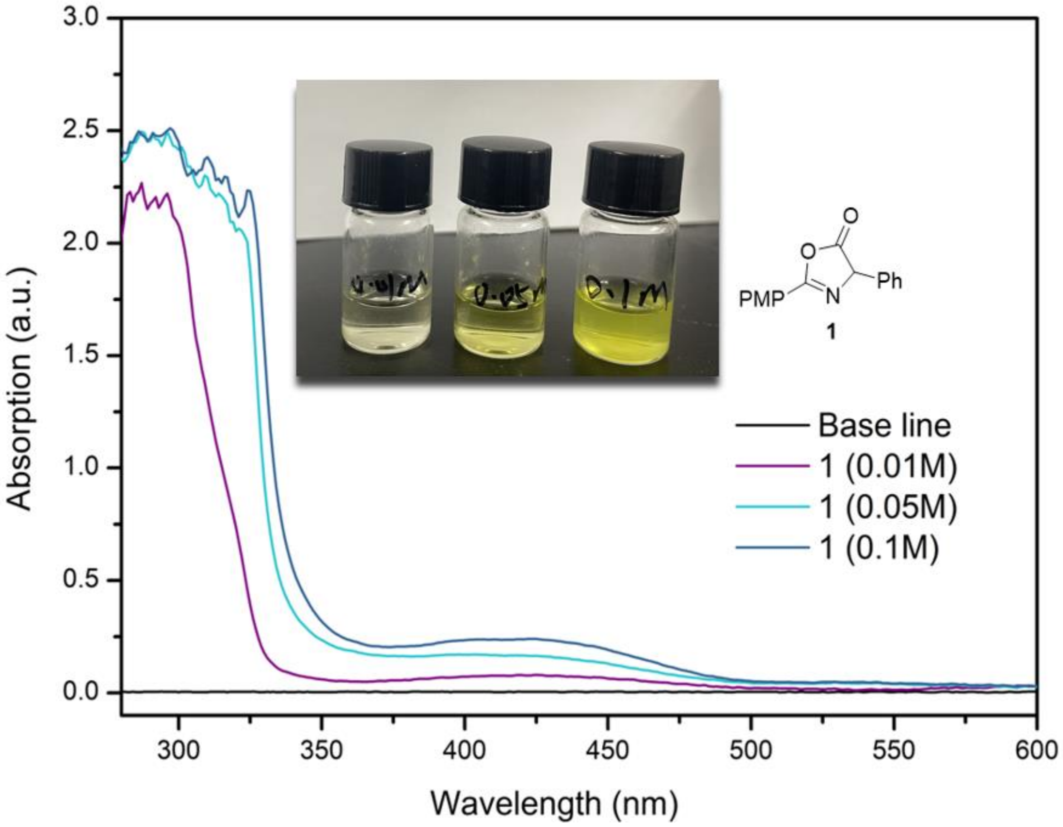

Supplementary Figure 18 UV-Vis absorption spectra of **1**was measured in different concentrations in *t*-BuCN

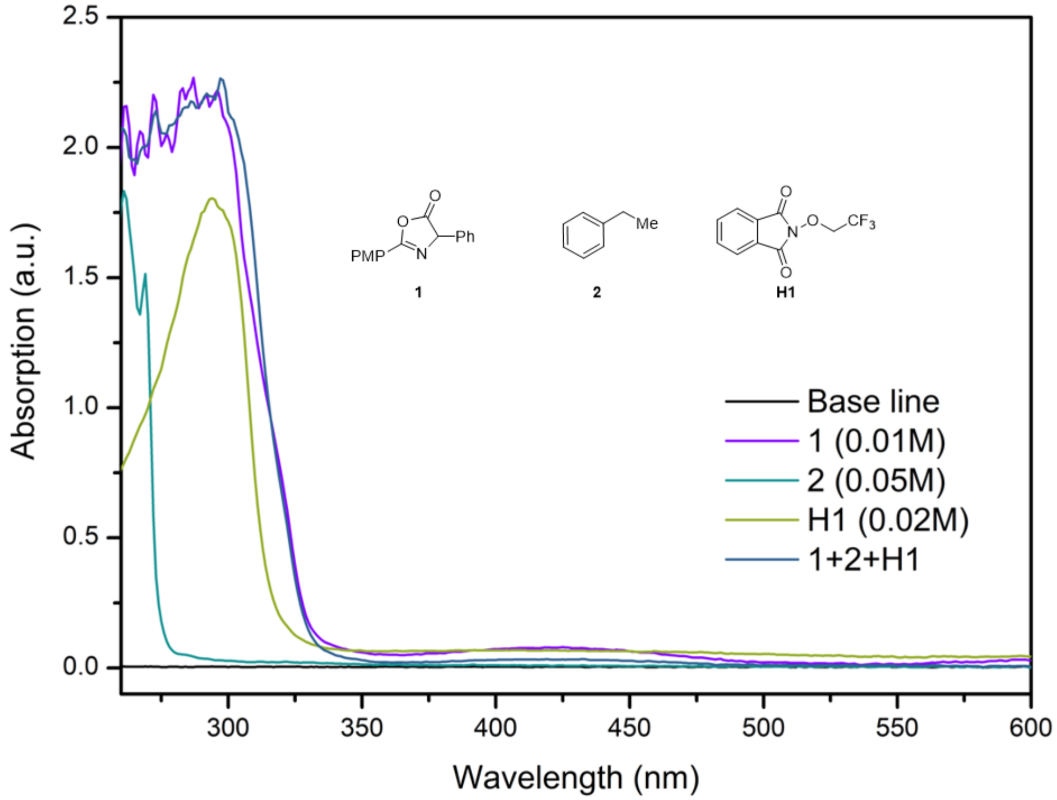

Supplementary Figure 19 UV-Vis absorption spectra of **1, 2** and **H1**were measured in *t*-BuCN

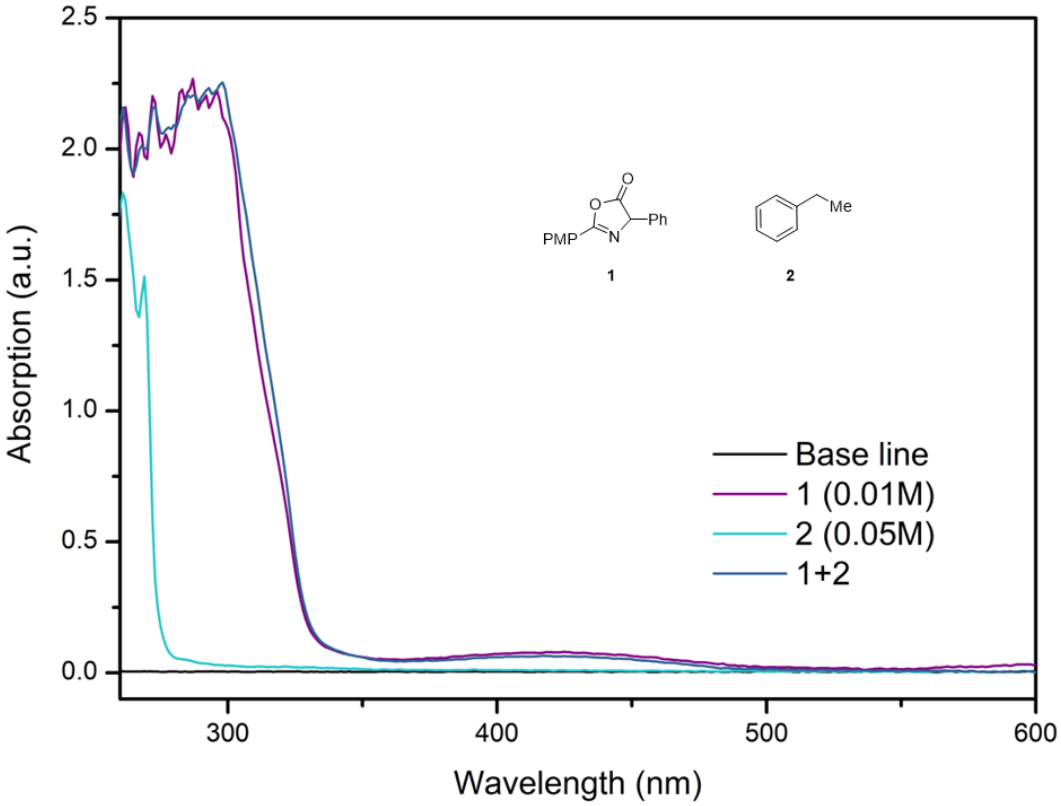

Supplementary Figure 20 UV-Vis absorption spectra of **1** and **2** were measured in *t*-BuCN

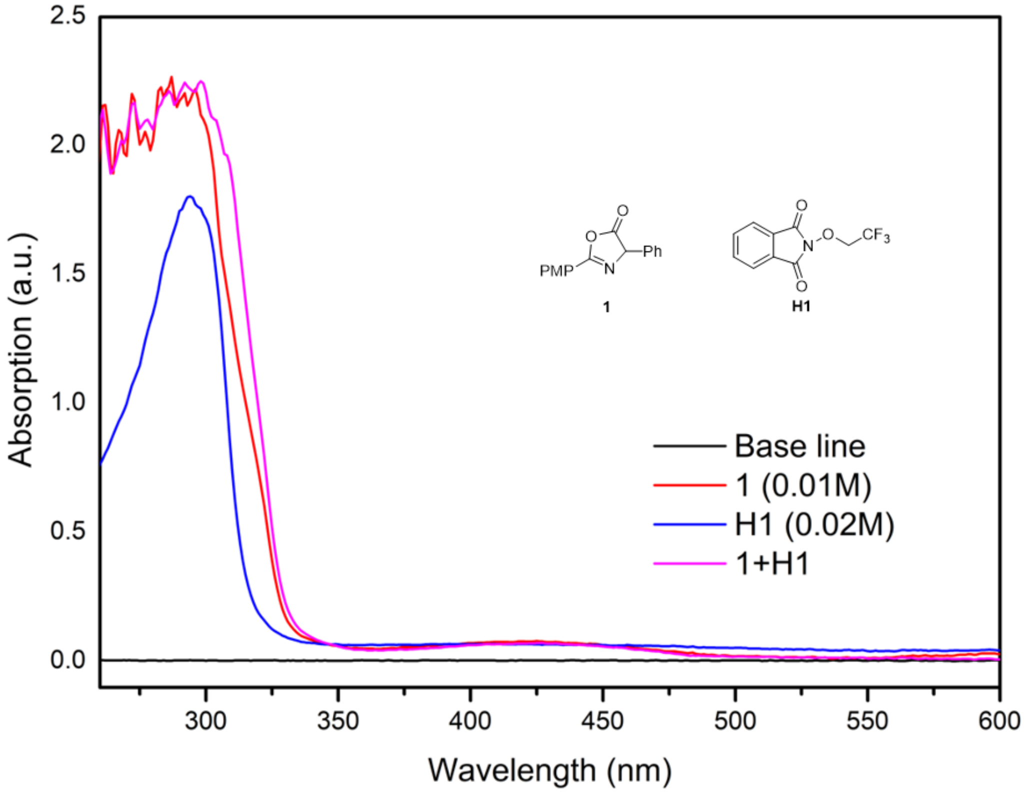

Supplementary Figure 21 UV-Vis absorption spectra of **1** and **H1** were measured in *t*-BuCN

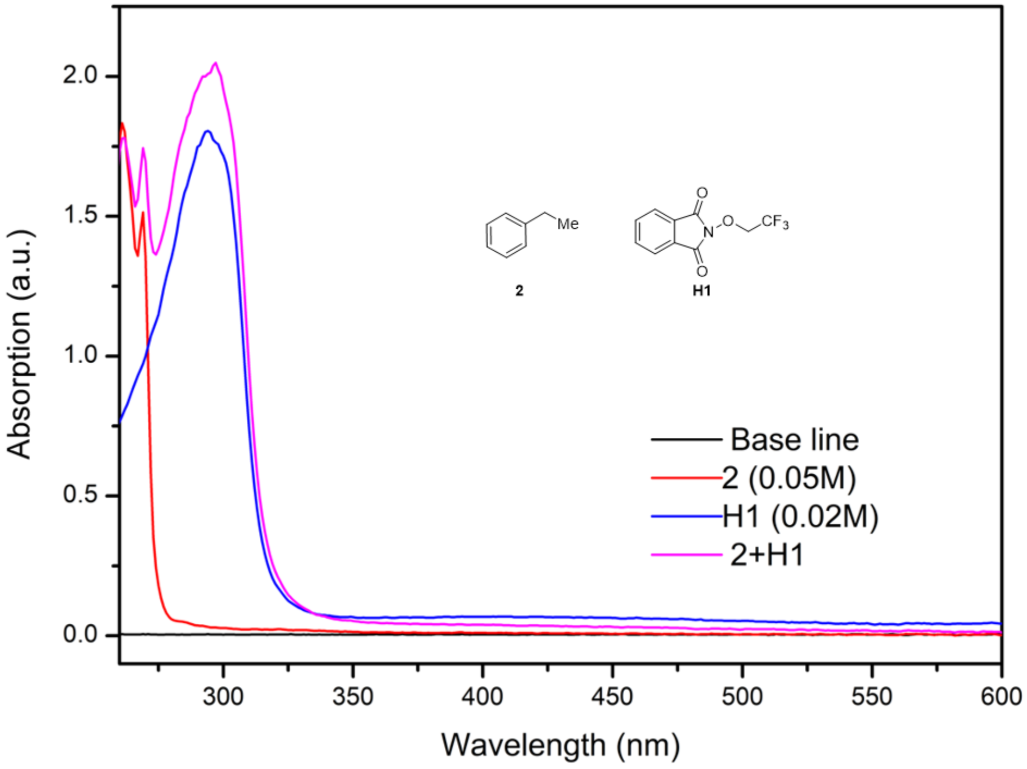

Supplementary Figure 22 UV-Vis absorption spectra of **2** and **H1** were measured in *t*-BuCN

Stern-Volmer fluorescence quenching experiments

Stern-Volmer fluorescence quenching experiments were run with freshly prepared solutions of 1×10^{-4} M **1**and varying concentrations of quencher (**H1** or **2**) in *t*-BuCN at room temperature. The solutions were irradiated at 365 nm and fluorescence was measured from 365 nm to 500 nm. Control experiments showed that excited state of **1** was quenched by **H1**.

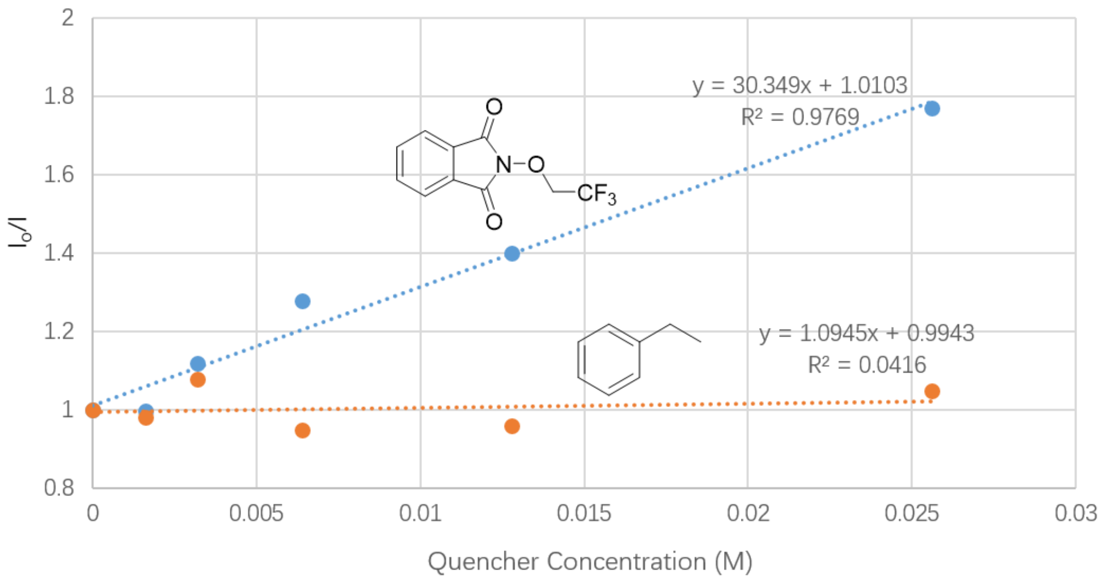

Supplementary Figure 23 Stern-Volmer plots of quenching of **H1**and **2** with **1** in *t*-BuCN.

Cyclic voltammetry studies

Cyclic voltammetry was performed in a three-electrode cell connected to a schlenk line at room temperature. The working electrode was a platinum disk electrode, the counter electrode a platinum wire. The reference was an Ag/AgCl electrode submerged in saturated aqueous KCl solution, and separated from reaction by a salt bridge. 5 mL of *t*-BuCN containing 0.1 M $^{n}Bu_4NPF_6$ were poured into the electrochemical cell in all experiments. The scan rate is 0.1 V/s, ranging from 0 V to ±3.0 V. The peak potentials vs. Ag/AgCl for used.

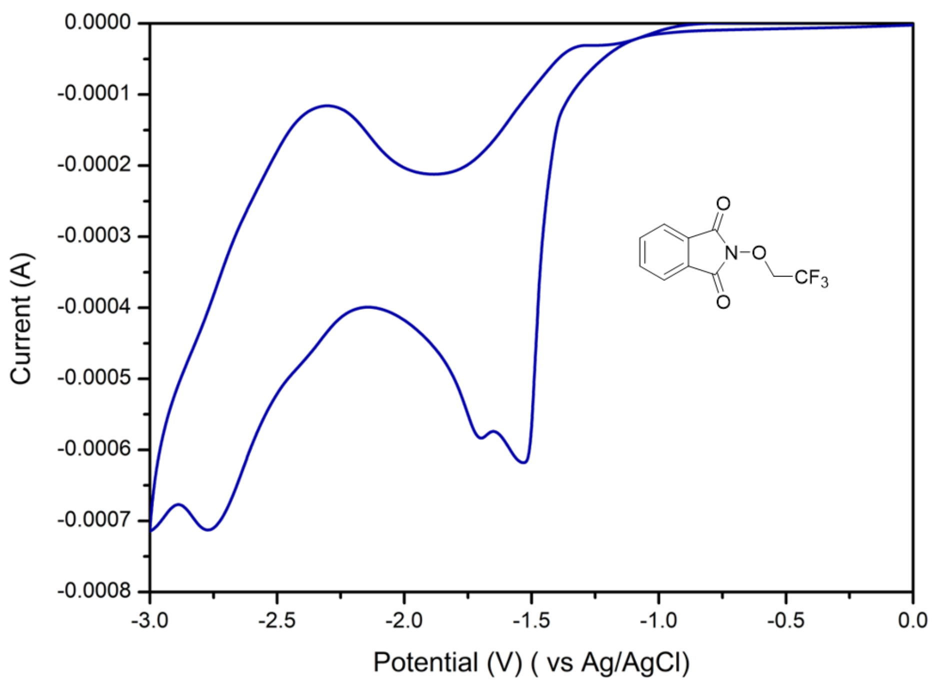

Supplementary Figure 24 N-Alkoxyphthalimide **H1** in *t*-BuCN (0.05 M)

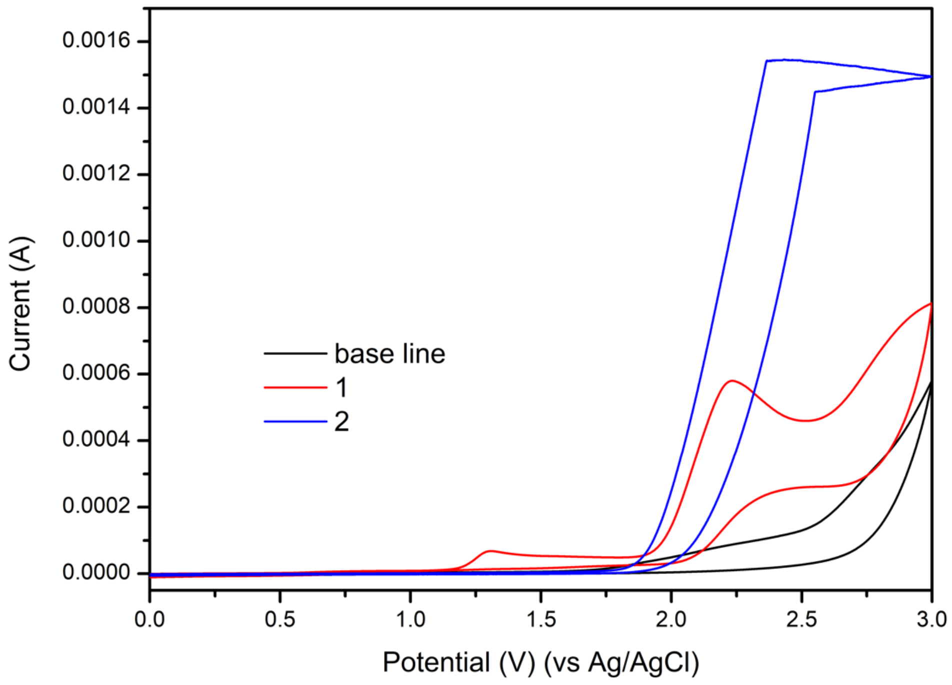

Supplementary Figure 25 1(0.05 M) and **2** (0.1 M) in *t*-BuCN

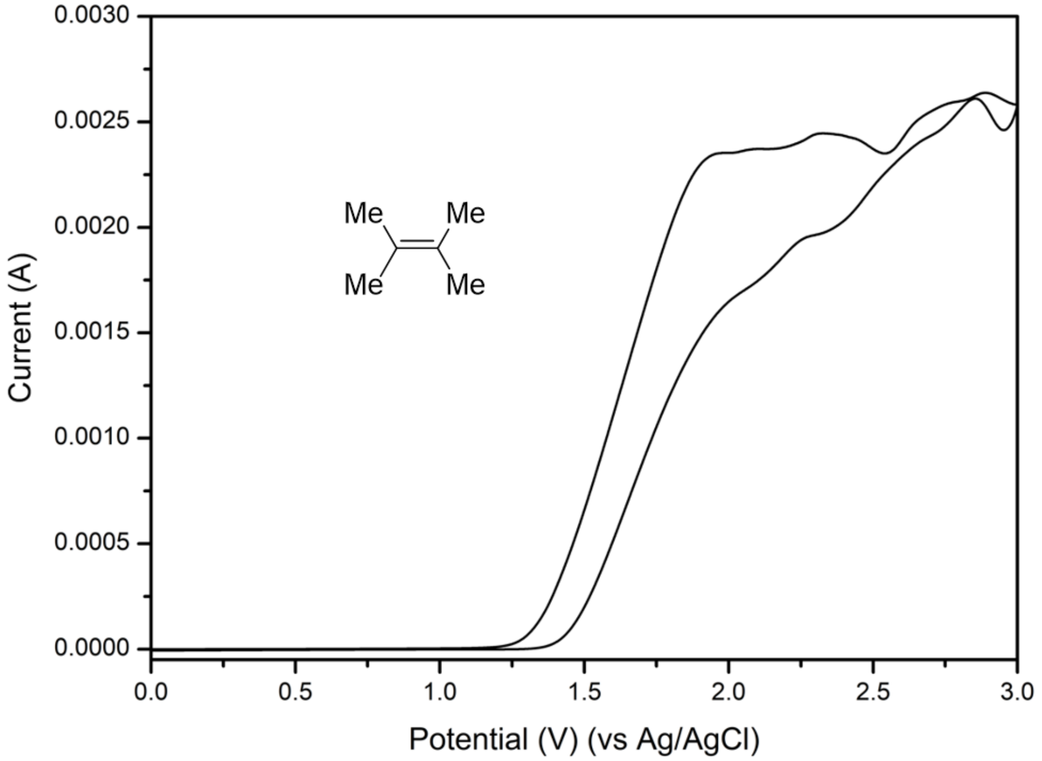

Supplementary Figure 26 2,3-Dimethyl-2-butene (0.1 M) in *t*-BuCN

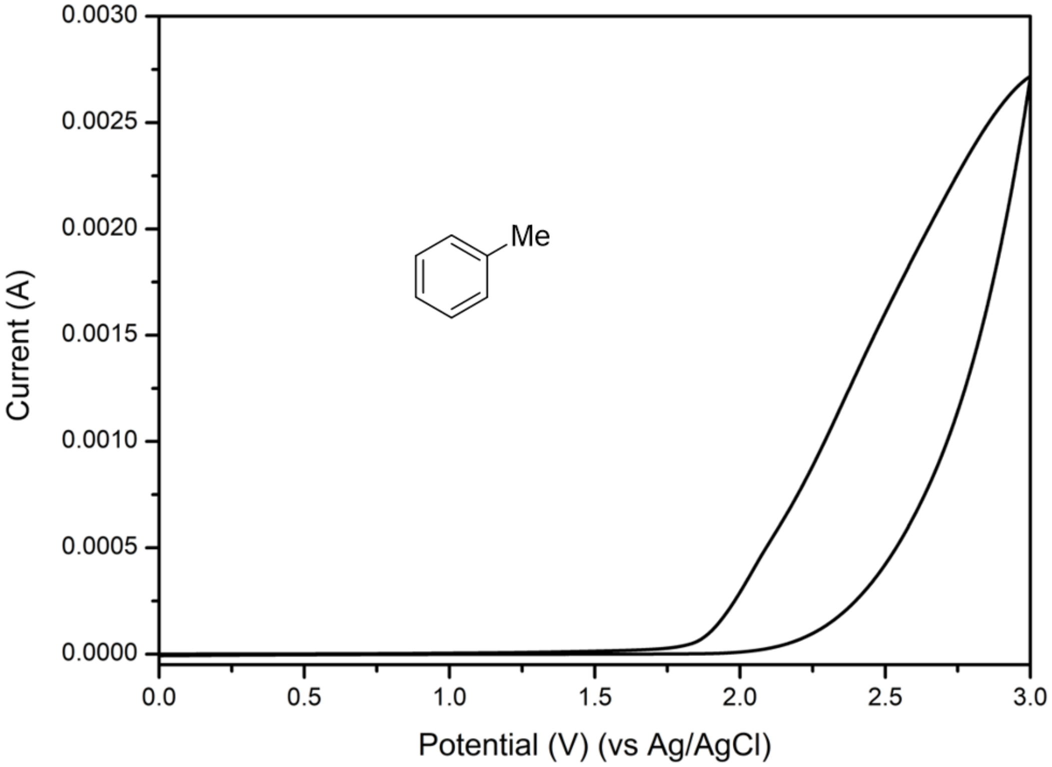

Supplementary Figure 27 Toluene (0.1 M) in *t*-BuCN

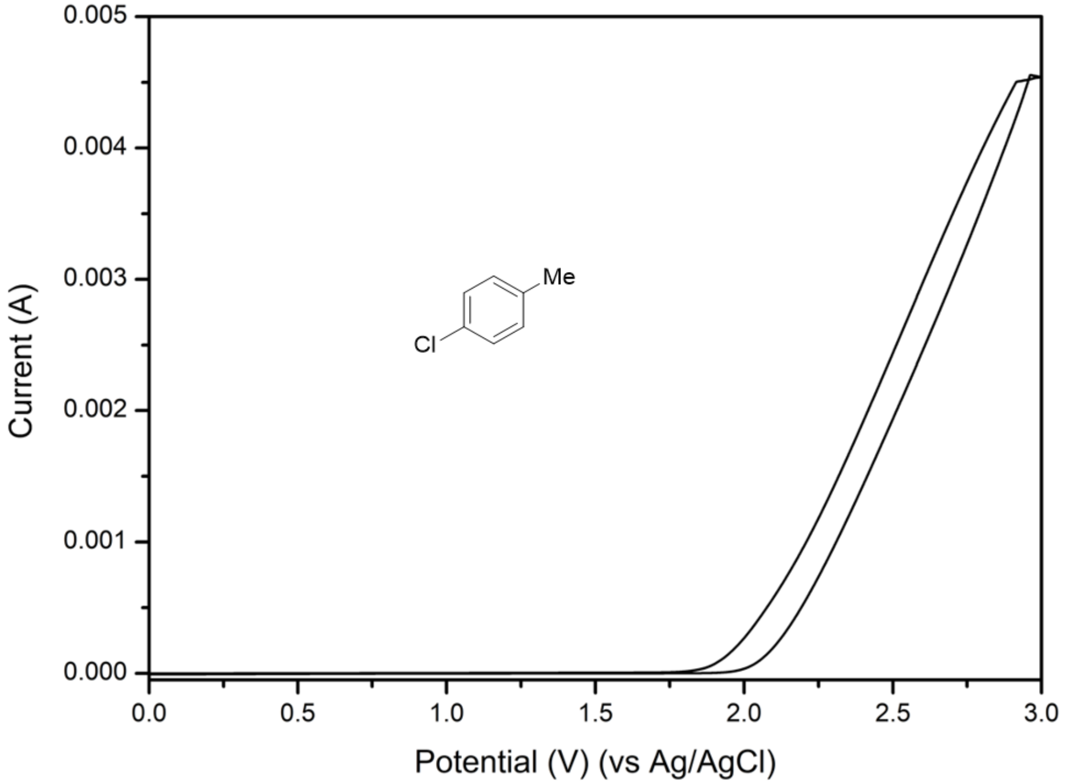

Supplementary Figure 28 4-Chlorotoluene (0.1 M) in *t*-BuCN

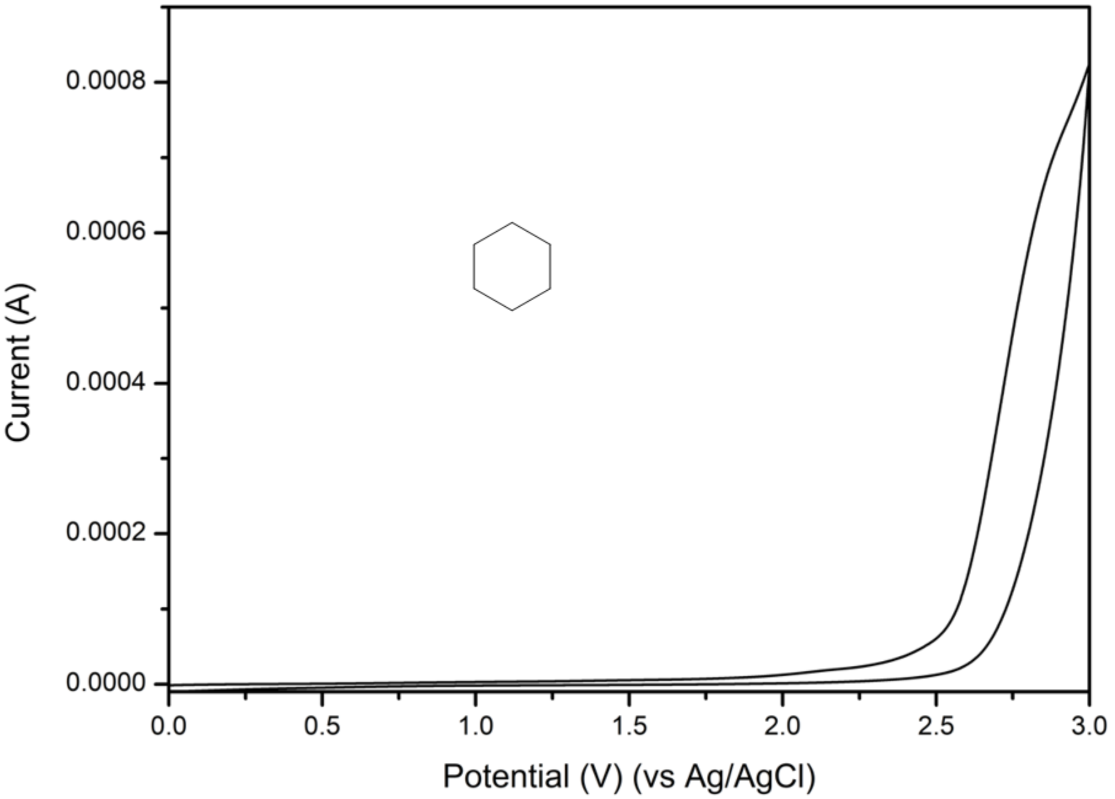

Supplementary Figure 29 Cyclohexane (0.1 M) in *t*-BuCN

Excluding arene radical cations

An oven-dried 10-mL Schlenk tube equipped with a stirrer was charged with the oxazolone **80** (0.1 mmol, 1.0 equiv.) and the phthalimide **H1** (0.2 mmol, 2.0 equiv). Then, 1.0 mL *t*-BuCN (0.1 M) was added followed by the trimethyl(phenylmethyl)-silan **92** (0.5 mmol, 5 equiv) in glove box. The tube was sealed with a screw cap and took out from glove box. The reaction mixture was inserted into the PhotoSyn 3.0 reactor and irradiated using a 10 W LED lamp (395 nm) for 12 h. The reaction mixture was concentrated in vacuo and purified by flash column chromatography (petroleum ether/EA = 20/1) provided **49** in 64% yield. No product **12** was detected in this process, it showed that the corresponding arene radical cations were not generated in this reaction.

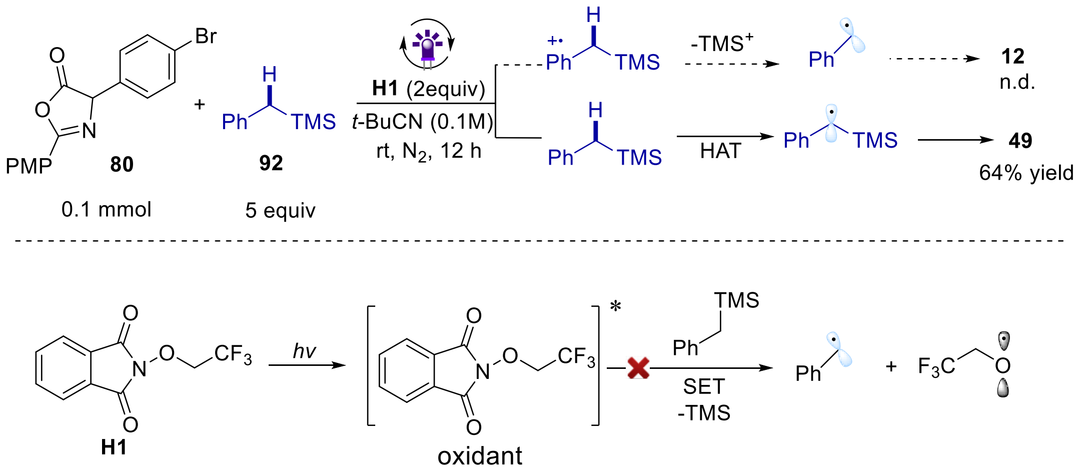

Supplementary Figure 30 Excluding arene radical cations

Proving photosensitive intermediates

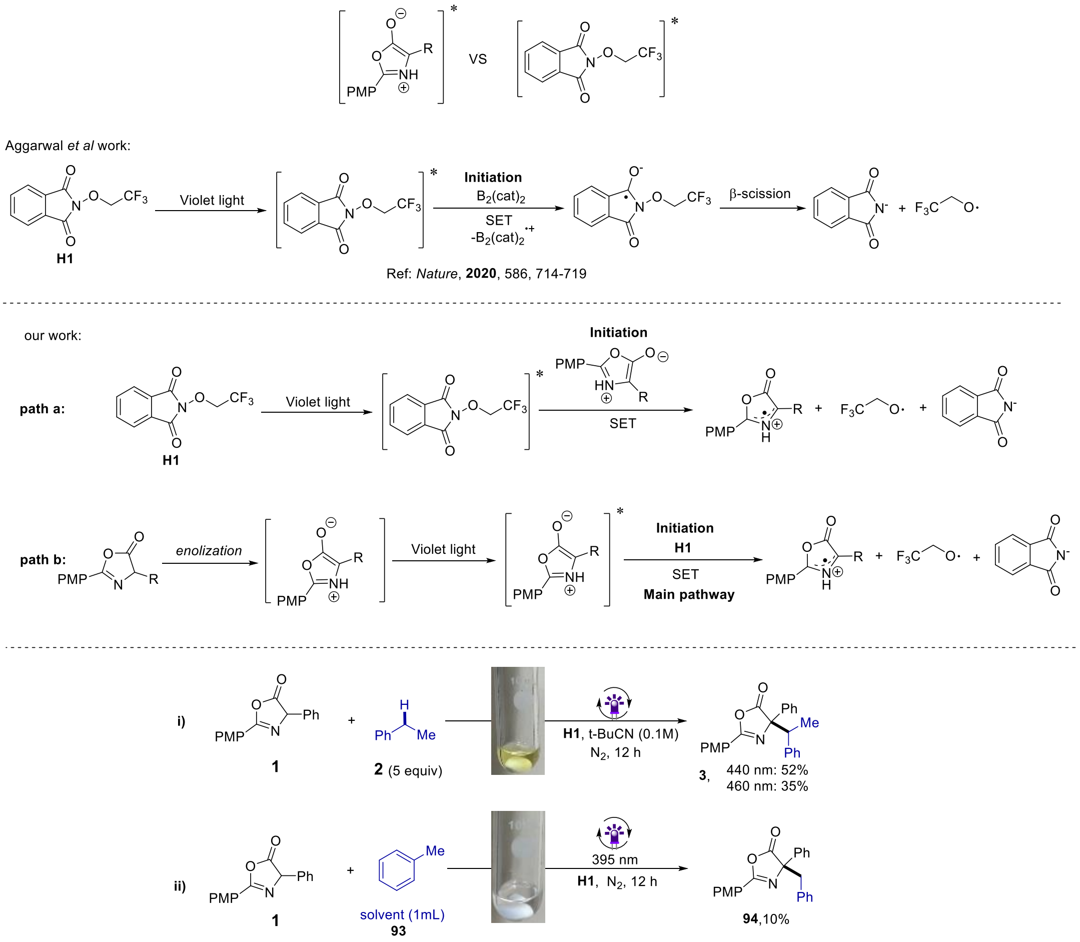

Supplementary Figure 31 Comparison of photosensitizer intermediates in the reaction

According to the previous report and Figure S31, Enolate intermediate of oxazolone and *N*-(2,2,2-trifluoroethoxy)phthalimide **H1** can be excited by violet light (about 400 nm) to initiate the reaction as photosensitive intermediates. In 2020, the Aggarwal and coworkers demonstrated a chlorine radical–boron 'ate' complex-mediated HAT process for the C(sp)^3–H borylation using N-(Trifluoroethoxy) phthalimide **H1**/B-chlorocatecholborane (ClB(cat)) system, After photoexcitation of **H1** to excited state **H1***, reductive quenching by B_2(cat)$_2$ and subsequent β-scission of radical anion **H1** gives the trifluoroethoxy radical. However, the light source of 450 nm is used, almost no C-H Boride product can be obtained (<10%yield), **H1** mainly can't excited. whether SET between excited state **H1*** and enolate intermediate of oxazolone is a plausible mechanism (Figure 31 path a), It is noteworthy that our reaction can proceed smoothly under blue light (>440 nm), which showed that enolate intermediate of oxazolone is more likely to be a photosensitive intermediate than **H1** (Figure 31i). In addition, the solvent amount of toluene was used as C-H feedstock, and the reaction solution was almost colorless, and only 10% of the target product was irradiated by violet light (Figure 31ii), which further indicated that enolate intermediate of oxazolone was photoinitiator. it is likely that trifluoroethoxy radical is generated by the mechanisms shown in Figure 31 (path b). However, it can not be completely ruled out that direct photoinitiation of **H1** may also have played a role (path a).

Intermolecular competition experiment

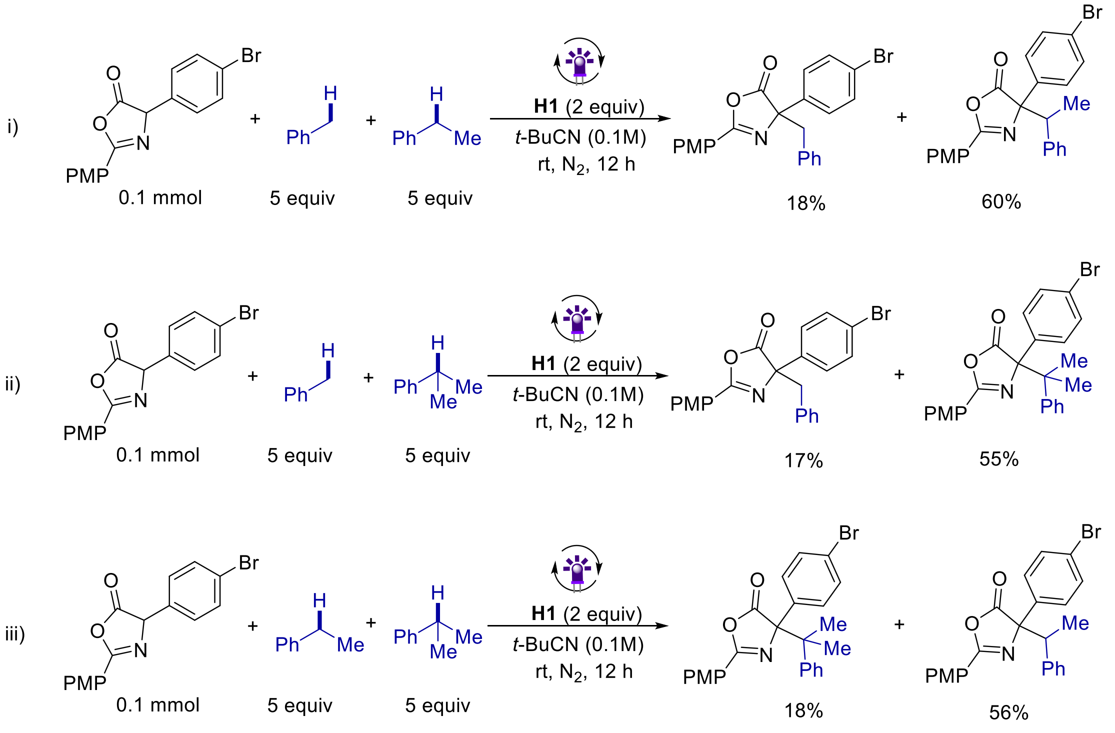

Supplementary Figure 32 Intermolecular competition experiment

Intermolecular competition KIE experiment

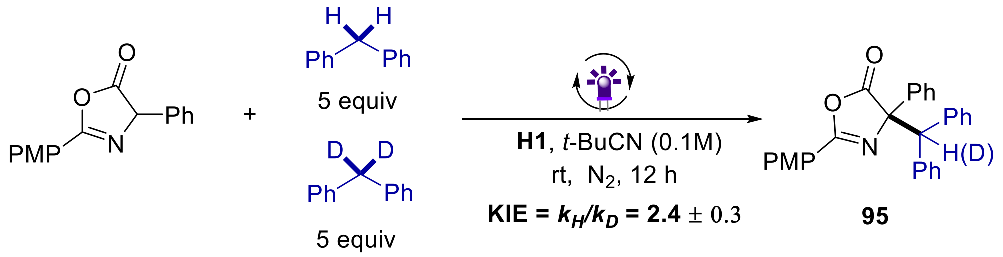

An oven-dried 10-mL Schlenk tube equipped with a stirrer was charged with the oxazolones **1** (0.1 mmol, 1.0 equiv.) and the phthalimide **H1** (0.2 mmol, 2.0 equiv). Then, 1.0 mL *t*-BuCN (0.1 M) was added followed by the diphenylmethane (0.5 mmol, 5 equiv) and ***d*₂**-diphenylmethane (0.5 mmol, 5 equiv) in glove box. The tube was sealed with a screw cap and took out from glove box. The reaction mixture was inserted into the PhotoSyn 3.0 reactor and irradiated using a 10 W LED lamp (395 nm) for 12 h. The reaction mixture was concentrated in vacuo and purified by flash column chromatography (petroleum ether/EA = 20/1). Average of two runs: *KIE*= 2.43

Run	**75**	***d*₁-75**	k_H/k_D	**Yield** (%)
1	0.713	0.287	2.48	72
2	0.704	0.296	2.38	66

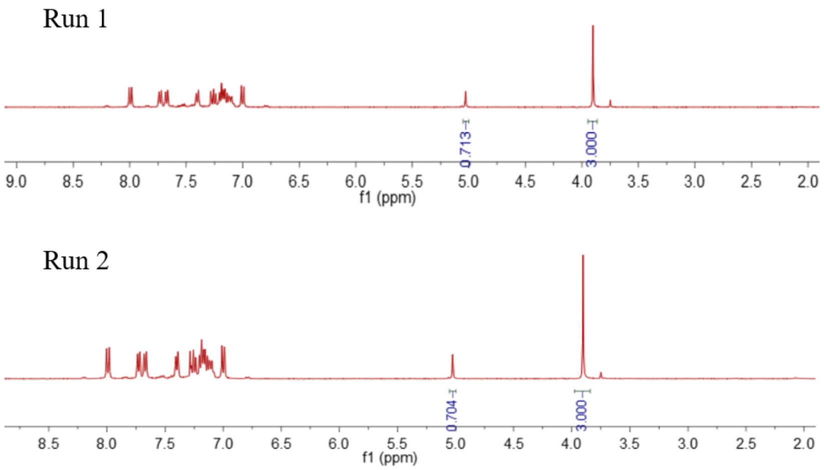

Supplementary Figure 33 Intermolecular competition KIE experiment

Determination of the light intensity at 395 nm:

According to the procedure of Yoon[1] and our previous work[2], the photon flux of the LED was determined by standard ferrioxalate actinometry. A 0.15 M solution of ferrioxalate was prepared by dissolving potassium ferrioxalate hydrate (2.21 g) in 30 mL of a 0.05 M H_2SO_4 solution. A buffered solution of 1,10-phenanthroline was prepared by dissolving 1,10-phenanthroline (50 mg) and sodium acetate (11.25 g) in 50 mL of a 0.5 M solution H_2SO_4. Both solutions were stored in the dark. To determine the photon flux of the LEDs, the ferrioxalate solution (2.0 mL) was placed in a cuvette and irradiated for 90 s at λmax = 395 nm. After irradiation, the phenanthroline solution (0.35 mL) was added to the cuvette and the mixture and stired in the dark for 1 h to allow the ferrous ions to completely coordinate to the phenanthroline. The absorbance of the solution was measured at 510 nm. A nonirradiated sample was also prepared and the absorbance at 510 nm was measured. Conversion was calculated using eq. 1.

$$\text{mol Fe}^{2+} = \frac{\text{V}\cdot\Delta\text{A}}{\text{l}\cdot\varepsilon} \quad (1)$$

where V is the total volume (0.00235 L) of the solution after addition of phenanthroline, ΔA is the difference in absorbance at 510 nm between the irradiated and non-irradiated solutions, l is the path length (1.00 cm), and ε is the molar absorptivity of the ferrioxalate actinometer at 510 nm (11,100 L mol-1 cm-1).

$$\text{photo flux} = \frac{\text{mol Fe}^{2+}}{\Phi\cdot\text{t}\cdot\text{f}} \quad (2)$$

where Φ is the quantum yield for the ferrioxalate actinometer (1.13 at λex = 395 nm)[3], t is the irradiation time (90 s), and f is the fraction of light absorbed at λex = 395nm by the ferrioxalate actinometer. This value is calculated using the following equation where A (395 nm) is the absorption of the ferrioxalate solution at 395 nm. An absorption spectrum gave an A (395 nm) value of > 3, indicating that the fraction of absorbed light (f) is >0.999.

$$\text{f} = 1 - 10^{-\text{A(395nm)}} \quad (3)$$

The average photon flux was thus calculated to be 4.4×10^{-9} einsteins s^{-1}.

Determination of the reaction quantum yield:

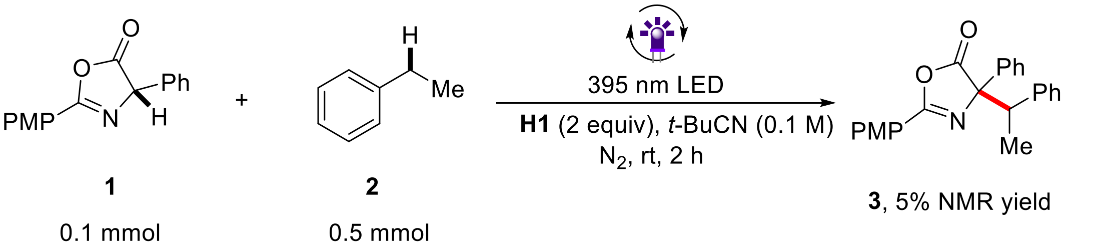

An oven-dried cuvette equipped with a stirrer was charged with the oxazolone **1** (0.1 mmol, 1.0 equiv.) and **H1** (0.2 mmol, 2.0 equiv). Then, *t*-BuCN (0.05M) and **2** (0.5 mmol, 5.0 equiv) were added in glove box. The tube was sealed with a screw cap and took out from glove box. Then at room temperate under LEDs (λmax = 395nm) for 2 h. The crude yield of the product **3** was determined by ^{1}H NMR based on a 4-Chlorobenzaldehyde standard and the final yield was 5%. The reaction quantum yield (Φ) was determined using eq. 4 where the photon flux is 4.4×10^{-9} einsteins s^{-1} (determined by actinometry as described above), t is the reaction time (7200 s) and f_R is the fraction of incident light absorbed by the reaction mixture, determined using eq 4. An absorption spectrum of the reaction mixture gave an absorbance value of > 3 at 395 nm (A >3 indicating that the fraction of light absorbed is >0.999).

$$\Phi = \frac{\text{Mol product}}{\text{flux}\cdot\text{t}\cdot\text{f}} \quad (4)$$

The reaction quantum yield (Φ) was thus determined to be Φ = 0.15

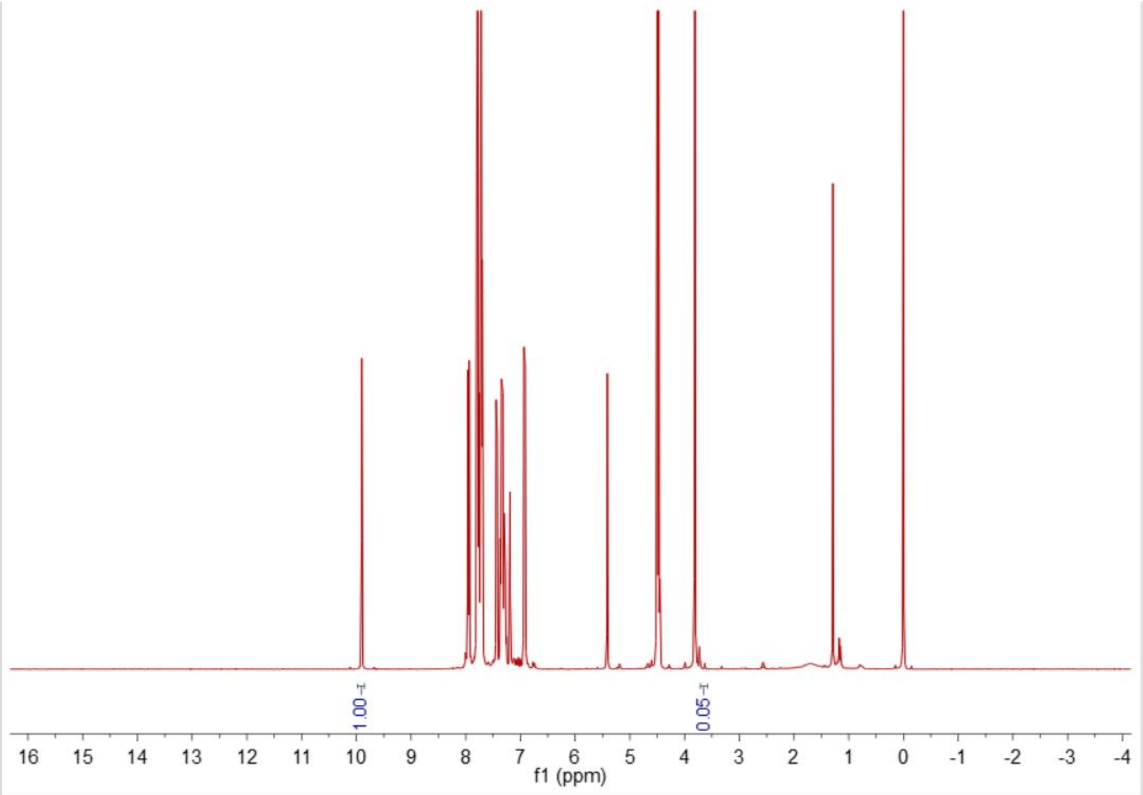

Supplementary Figure 34 ^{1}H NMR yield of **3**

Substrate Synthesis

Synthesis of azlactone according to literature[2,4], **H5** and **H8** were synthesized according to the literature[5,6].

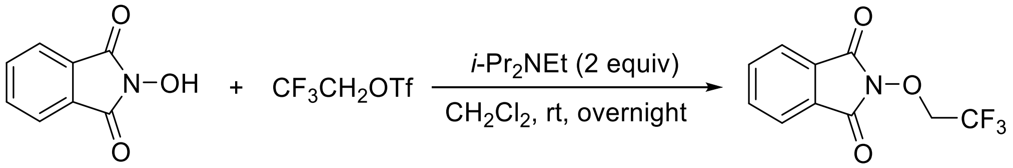

Under a nitrogen atmosphere, to a solution of -hydroxyphthalimide (1.63 g, 10.0 mmol, 1.0 equiv.) in CH_2Cl_2 (30 mL) at r.t. was added *i*-Pr_2NEt (2.58 g, 20.0 mmol, 2.0 equiv.) followed by 2,2,2-trifluoroethyl trifluoromethanesulfonate (2.55 g, 11.0 mmol, 1.10 equiv.) and the solution stirred overnight. The reaction was quenched with water (30 mL) and extracted into CH_2Cl_2 (3 × 40 mL). The organic extracts were combined and washed with brine (30 mL), dried over Na_2SO_4, filtered, and concentrated in vacuo. The residue was purified by flash column chromatography (petroleum ether/EA = 10/1) to give 2-(2,2,2-Trifluoroethoxy)isoindoline-1,3-dione.

^{1}H NMR (600 MHz, $CDCl_3$) δ 7.88 – 7.86 (m, 2H), 7.82 – 7.76 (m, 2H), 4.56 (q, J = 8.0 Hz, 2H).

^{13}C NMR (151 MHz, $CDCl_3$) δ 162.53, 134.96, 128.58, 123.97, 122.38 (q, *J* = 279.3 Hz), 73.09 (q, *J* = 35.2 Hz).

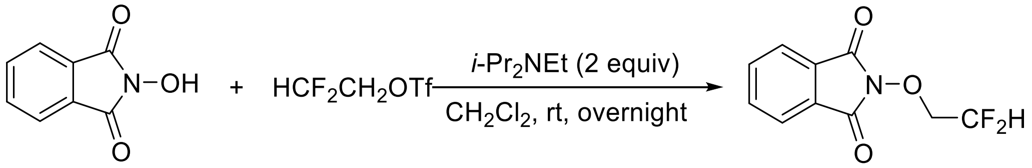

Under a nitrogen atmosphere, to a solution of -hydroxyphthalimide (1.63 g, 10.0 mmol, 1.0 equiv.) in CH_2Cl_2 (30 mL) at r.t. was added i-Pr_2NEt (2.58 g, 20.0 mmol, 2.0 equiv.) followed by 2,2-difluoroethyl trifluoromethanesulfonate (11.0 mmol, 1.10 equiv.) and the solution stirred overnight. The reaction was quenched with water (30 mL) and extracted into CH_2Cl_2 (3 × 40 mL). The organic extracts were combined and washed with brine (30 mL), dried over Na_2SO_4, filtered, and concentrated in vacuo. The residue was purified by flash column chromatography (petroleum ether/EA = 10/1) to give 2-(2,2-difluoroethoxy)isoindoline-1,3-dione.

^{1}H NMR (400 MHz, $CDCl_3$) δ 7.84 – 7.77 (m, 2H), 7.76 – 7.68 (m, 2H), 6.30 – 6.01 (m, 1H), 4.35 – 4.27 (m, 2H).

^{13}C NMR (101 MHz, $CDCl_3$) δ 163.01, 134.88, 128.62, 123.89, 113.21 (t, *J* = 242.4 Hz), 76.06 (t, *J* = 29.4 Hz).

^{19}F NMR (377 MHz, $CDCl_3$) δ -124.46.

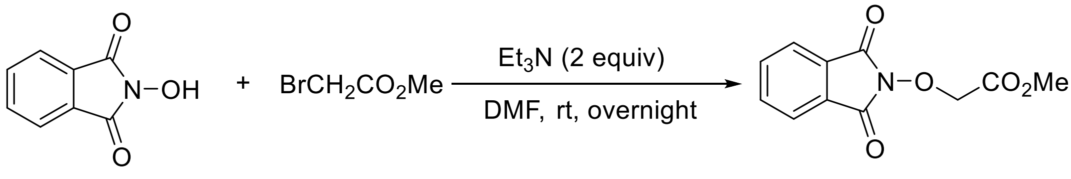

To a solution of N-hydroxyphthalimide (0.81 g, 5.0 mmol, 1.0 equiv.) in DMF (20 mL) at r.t. was added Et_3N (1.02 g, 10.0 mmol, 2.0 equiv.) followed by bromoacetonitrile (7.5 mmol, 1.5 equiv.) and the solution stirred overnight. The reaction was quenched with water (30 mL) and extracted into CH_2Cl_2 (3 × 40 mL). The organic extracts were combined and washed with brine (30 mL), dried over Na_2SO_4, filtered, and concentrated in vacuo. The residue was purified by flash column chromatography (petroleum ether/EA = 5/1) to give methyl 2-((1,3-dioxoisoindolin-2-yl)oxy)acetate.

^{1}H NMR (600 MHz, $CDCl_3$) δ 7.89 – 7.80 (m, 2H), 7.77 – 7.75 (m, 2H), 4.82 (s, 2H), 3.80 (s, 3H).

^{13}C NMR (151 MHz, $CDCl_3$) δ 167.32, 162.97, 134.75, 128.76, 123.79, 73.07, 52.46.

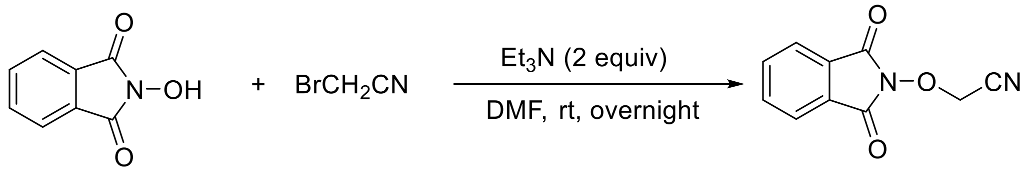

To a solution of N-hydroxyphthalimide (0.81 g, 5.0 mmol, 1.0 equiv.) in DMF (20 mL) at r.t. was added Et_3N (1.02 g, 10.0 mmol, 2.0 equiv.) followed by bromoacetonitrile (0.9 g, 7.5 mmol, 1.5 equiv.) and the solution stirred overnight. The reaction was quenched with water (30 mL) and extracted into CH_2Cl_2 (3 × 40 mL). The organic extracts were combined and washed with brine (30 mL), dried over Na_2SO_4, filtered, and concentrated in vacuo. The residue was purified by flash column chromatography (petroleum ether/EA = 5/1) to give 2-((1,3-dioxoisoindolin-2-yl)oxy)acetonitrile.

^{1}H NMR (400 MHz, $CDCl_3$) δ 7.93 – 7.86 (m, 2H), 7.85 – 7.78 (m, 2H), 4.96 (s, 2H).

^{13}C NMR (101 MHz, $CDCl_3$) δ 162.72, 135.17, 128.53, 124.17, 113.70, 61.93.

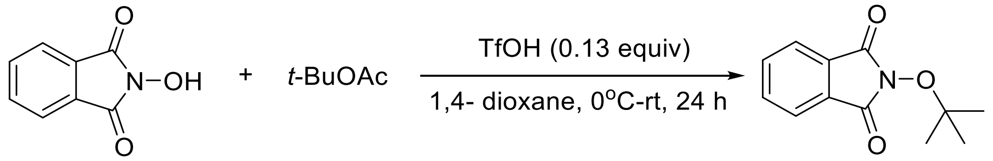

To a solution of N-hydroxyphthalimide (1.38 g, 8.45 mmol, 1.00 equiv.) in 1,4-dioxane (40 mL) at 0 °C was added tert-butyl acetate (13.5 mL, 16.9 mmol, 2.0 equiv.) and triflic acid (0.1 mL, 1.13 mmol, 0.133 equiv.) and the solution stirred for 24 h at r.t. The reaction mixture was poured into saturated aq. $NaHCO_3$ (60 mL), and further diluted with saturated aq. $NaHCO_3$ until the aqueous layer was basic (pH >10). The organic layer was separated, and the aqueous layer was extracted with CH_2Cl_2 (3 × 50 mL). The combined organic extracts were dried over Na_2SO_4, filtered, and concentrated in vacuo. The residue was purified by flash column chromatography (petroleum ether/EA = 10/1) to give 2-(tert-butoxy)isoindoline-1,3-dione.

^{1}H NMR (400 MHz, $CDCl_3$) δ 7.85 – 7.81 (m, 2H), 7.79 – 7.71 (m, 2H), 1.42 (s, 9H).

^{13}C NMR (101 MHz, $CDCl_3$) δ165.70, 134.43, 129.33, 123.47, 86.64, 27.33.

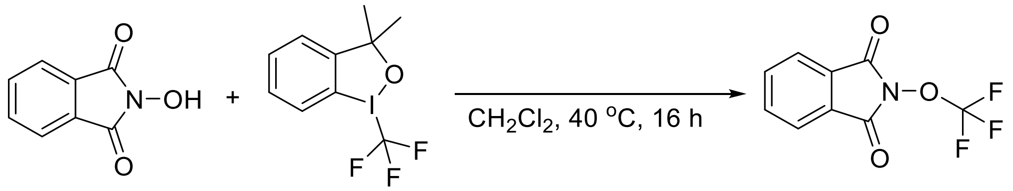

In a glovebox, to an oven-dried 20 mL screw cap vial was added 2-hydroxyisoindoline-1,3-dione (0.163 g, 1.00 mmol, 1.0 equiv), Tongi reagent I (0.330 g, 1.0 mmol, 1.0 eq). Then 10 mL DCM (0.1 M) was added with a magnetic stir bar. The vial was capped and taken out of the glovebox. The reaction mixture was then stirred at 40 °C for 16 h. Then the reaction mixture was concentrated in vacuo and purified by flash column chromatography (petroleum ether/EA = 20/1). The solid was further purified by recrystallization using DCM and hexanes.

^{1}H NMR (400 MHz, CDCl$_3$) δ 7.96 – 7.92 (m, 2H), 7.90 – 7.83 (m, 2H).

^{13}C NMR (101 MHz, CDCl$_3$) δ 161.54, 135.52, 128.56, 124.53, 122.07 (q, J = 268.6 Hz).

^{19}F NMR (377 MHz, CDCl$_3$) δ -65.24.

Analytical Data of Compounds

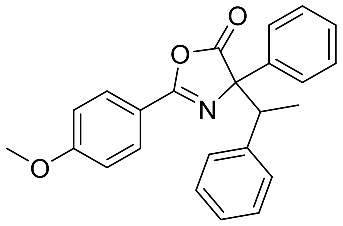

2-(4-methoxyphenyl)-4-phenyl-4-(1-phenylethyl)oxazol-5(4H)-one (3)

Following **procedure A** on 0.1 mmol scale. Purification via silica gel column chromatography (Petroleum ether/Ethyl acetate, 20:1) afforded 25.6 mg (70%) of the title compound.

^{1}H NMR (400 MHz, CDCl$_3$) δ 8.05 – 8.00 (m, 1H), 7.92 – 7.86 (m, 1H), 7.86 – 7.81 (m, 1H), 7.60 – 7.55 (m, 1H), 7.45 – 7.39 (m, 1H), 7.38 – 7.28 (m, 1H), 7.25 – 7.08 (m, 6H), 7.02 – 6.92 (m, 2H), 3.88 (d, J = 8.0 Hz, 3H), 3.73 – 3.58 (m, 1H), 1.35 – 1.28 (m, 3H).

^{13}C NMR (101 MHz, CDCl$_3$) δ 178.04, 177.58, 162.14, 162.01, 158.70, 158.51, 139.16, 138.62, 136.83, 136.62, 128.88, 128.74, 128.05, 128.01, 127.49, 127.05, 126.91, 126.70, 126.56, 126.36, 125.75, 125.25, 125.09, 117.23, 117.20, 113.13, 113.05, 112.40, 77.08, 76.84, 76.19, 54.47, 54.42, 49.07, 48.93, 15.39, 14.46.

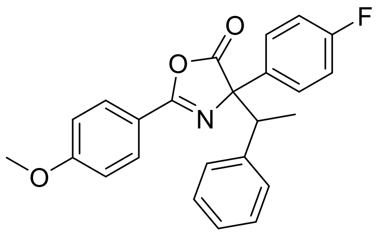

4-(4-fluorophenyl)-2-(4-methoxyphenyl)-4-(1-phenylethyl)oxazol-5(4H)-one (4)

Following **procedure A** on 0.1 mmol scale. Purification via silica gel column chromatography (Petroleum ether/Ethyl acetate, 20:1) afforded 23.3 mg (60%) of the title compound.

^{1}H NMR (400 MHz, CDCl$_3$) δ 8.13 – 7.98 (m, 1H), 7.91 – 7.84 (m, 1H), 7.83 – 7.78 (m, 1H),

7.55 – 7.49 (m, 1H), 7.26 – 7.22 (m, 1H), 7.19 – 7.06 (m, 5H), 7.00 (m, 1H), 6.97 – 6.93 (m, 1H), 6.89 (m, 1H), 3.88 (d, *J* = 9.7 Hz, 3H), 3.65 – 3.52 (m, 1H), 1.29 (dd, *J* = 10.7, 7.1 Hz, 3H).
^{13}C NMR (101 MHz, CDCl$_3$) δ 178.04, 177.48, 162.27, 162.12, 158.89, 158.74, 138.96, 138.38, 132.56 (d, *J* = 3.2 Hz), 132.44 (d, *J* = 2.9 Hz), 128.94, 128.78, 128.03, 127.98, 127.08 (d, *J* = 8.1 Hz), 126.95, 126.87 (d, *J* = 8.0 Hz), 126.66, 126.44, 125.86, 117.06, 117.03, 114.37 (d, *J* = 21.3 Hz), 113.89 (d, *J* = 21.7 Hz), 113.19, 113.10, 76.61, 76.19, 54.50, 54.44, 49.30, 49.09, 15.42, 14.35.
^{19}F NMR (377 MHz, CDCl$_3$) δ -114.15, -114.46.
HRMS (ESI) calcd for $C_{24}H_{21}FNO_3$ $(M+H)^+$: 390.1500, found: 390.1502.

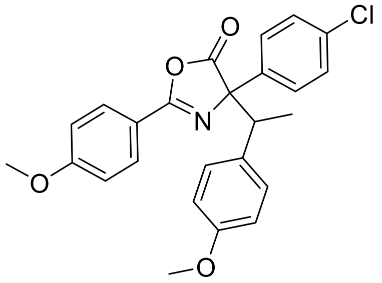

4-(4-chlorophenyl)-2-(4-methoxyphenyl)-4-(1-(4-methoxyphenyl)ethyl)oxazol-5(4H)-one (5)
Following **procedure A** on 0.1 mmol scale. Purification via silica gel column chromatography (Petroleum ether/Ethyl acetate, 20:1) afforded 30.5 mg (70%) of the title compound.
^{1}H NMR (400 MHz, CDCl$_3$) δ 8.07 – 7.98 (m, 1H), 7.93 – 7.84 (m, 1H), 7.81 – 7.72 (m, 1H), 7.54 – 7.46 (m, 1H), 7.41 – 7.35 (m, 1H), 7.17 (dd, *J* = 10.8, 8.7 Hz, 2H), 7.09 – 6.91 (m, 3H), 6.75 – 6.62 (m, 2H), 3.88 (d, *J* = 7.9 Hz, 3H), 3.72 (d, *J* = 8.9 Hz, 3H), 3.63 – 3.45 (m, 1H), 1.31 – 1.21 (m, 3H).
^{13}C NMR (101 MHz, CDCl$_3$) δ 178.90, 178.42, 163.33, 163.19, 160.00, 159.83, 158.82, 158.41, 136.47, 136.35, 134.10, 133.68, 131.95, 131.38, 130.03, 130.01, 129.99, 129.87, 128.67, 128.24, 127.75, 127.59, 118.08, 118.04, 114.24, 114.16, 113.36, 113.09, 77.59, 77.24, 55.55, 55.50, 55.13, 49.49, 49.38, 16.62, 15.62.
HRMS (ESI) calcd for $C_{25}H_{23}ClNO_4$ $(M+H)^+$: 436.1310, found: 436.1316.

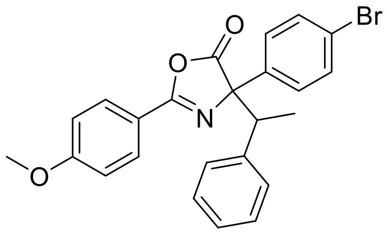

4-(4-bromophenyl)-2-(4-methoxyphenyl)-4-(1-phenylethyl)oxazol-5(4H)-one (6)
Following **procedure A** on 0.1 mmol scale. Purification via silica gel column chromatography (Petroleum ether/Ethyl acetate, 20:1) afforded 36.4 mg (81%) of the title compound.
^{1}H NMR (400 MHz, CDCl$_3$) δ 7.99 – 7.92 (m, 1H), 7.84 – 7.76 (m, 1H), 7.68 – 7.60 (m, 1H), 7.51 – 7.42 (m, 1H), 7.39 – 7.32 (m, 1H), 7.29 – 7.23 (m, 1H), 7.18 – 7.14 (m, 1H), 7.13 – 7.02 (m, 4H), 6.96 – 6.83 (m, 2H), 3.80 (d, *J* = 9.8 Hz, 3H), 3.59 – 3.44 (m, 1H), 1.25 – 1.19 (m, 3H).
^{13}C NMR (101 MHz, CDCl$_3$) δ 177.73, 177.18, 162.31, 162.16, 158.99, 158.84, 138.81, 138.25, 135.89, 135.73, 130.60, 130.14, 128.95, 128.80, 128.01, 127.97, 127.08, 126.96, 126.87, 126.72, 126.49, 125.94, 121.34, 120.96, 116.97, 116.94, 113.20, 113.11, 76.71, 76.42, 54.49,

54.44, 49.15, 49.01, 25.88, 15.44, 14.38.

HRMS (ESI) calcd for $C_{24}H_{21}{}^{79}BrNO_3$ $(M+H)^+$: 450.0699, found: 450.0707; calcd for $C_{24}H_{21}{}^{81}Br$ NO_3 $(M+H)^+$: 452.0679, found: 452.0686.

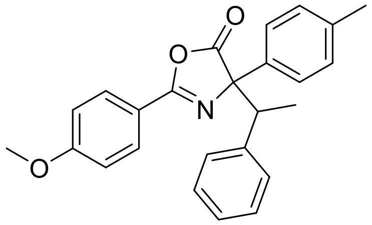

2-(4-methoxyphenyl)-4-(1-phenylethyl)-4-(p-tolyl)oxazol-5(4H)-one (7)

Following **procedure A** on 0.1 mmol scale. Purification via silica gel column chromatography (Petroleum ether/Ethyl acetate, 20:1) afforded 23.5 mg (61%) of the title compound.

^{1}H NMR (400 MHz, CDCl$_3$) δ 7.99 – 7.89 (m, 1H), 7.84 – 7.77 (m, 1H), 7.62 (d, *J* = 8.2 Hz, 1H), 7.37 (d, *J* = 8.3 Hz, 1H), 7.19 – 7.03 (m, 6H), 7.01 – 6.80 (m, 3H), 3.80 (d, *J* = 6.8 Hz, 3H), 3.64 – 3.49 (m, 1H), 2.32 – 2.16 (m, 3H), 1.23 (t, *J* = 6.9 Hz, 3H).

^{13}C NMR (101 MHz, CDCl$_3$) δ 177.72, 161.98, 139.33, 138.73, 136.83, 133.83, 133.58, 128.85, 128.73, 128.19, 128.08, 128.01, 127.77, 126.89, 126.58, 126.30, 125.70, 125.11, 124.97, 117.31, 113.10, 113.04, 76.98, 76.19, 54.42, 48.79, 48.74, 20.09, 20.00, 15.40, 14.45.

HRMS (ESI) calcd for $C_{25}H_{24}NO_3$ $(M+H)^+$: 386.1751, found: 386.1755.

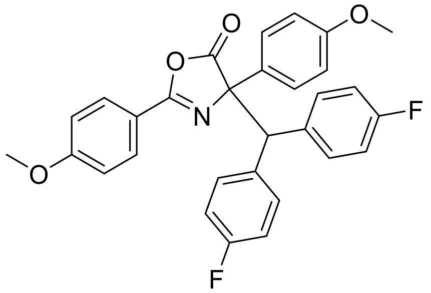

4-(bis(4-fluorophenyl)methyl)-2,4-bis(4-methoxyphenyl)oxazol-5(4H)-one (8)

Following **procedure A** on 0.1 mmol scale. Purification via silica gel column chromatography (Petroleum ether/Ethyl acetate, 20:1) afforded 35.9 mg (72%) of the title compound.

^{1}H NMR (400 MHz, CDCl$_3$) δ 7.93 – 7.84 (m, 2H), 7.57 – 7.44 (m, 4H), 7.25 – 7.19 (m, 2H), 6.95 – 6.88 (m, 2H), 6.84 – 6.65 (m, 6H), 4.86 (s, 1H), 3.81 (s, 3H), 3.65 (s, 3H).

^{13}C NMR (101 MHz, CDCl$_3$) δ 162.35, 160.92 (d, *J* = 246.4 Hz), 160.48 (d, *J* = 245.4 Hz), 159.31, 158.20, 133.91 (d, *J* = 3.3 Hz), 133.06 (d, *J* = 2.8 Hz), 130.32 (d, *J* = 8.0 Hz), 130.28 (d, *J* = 7.8 Hz), 128.87, 127.92, 126.44, 116.76, 114.00 (d, *J* = 21.2 Hz), 113.24, 112.75, 76.58, 76.19, 57.72, 54.49, 54.12.

^{19}F NMR (377 MHz, CDCl$_3$) δ -115.04, -116.00.

HRMS (ESI) calcd for $C_{30}H_{24}F_2NO_4$ $(M+H)^+$: 500.1668, found: 500.1667.

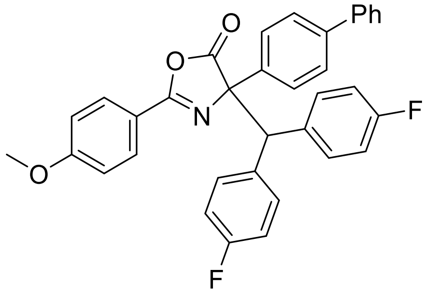

4-([1,1'-biphenyl]-4-yl)-4-(bis(4-fluorophenyl)methyl)-2-(4-methoxyphenyl)oxazol-5(4H)-one (9)

Following **procedure A** on 0.1 mmol scale. Purification via silica gel column chromatography (Petroleum ether/Ethyl acetate, 20:1) afforded 39.2 mg (72%) of the title compound.

^{1}H NMR (400 MHz, CDCl$_3$) δ 7.96 – 7.87 (m, 2H), 7.70 – 7.53 (m, 4H), 7.47 – 7.21 (m, 9H), 6.98 – 6.89 (m, 2H), 6.87 – 6.73 (m, 4H), 4.94 (s, 1H), 3.82 (s, 3H).

^{13}C NMR (101 MHz, CDCl$_3$) δ 177.44, 162.43, 160.97 (d, *J* = 246.9 Hz), 160.53 (d, *J* = 245.6 Hz), 159.49, 139.80, 139.20, 134.89, 133.76 (d, *J* = 3.1 Hz), 132.96 (d, *J* = 3.4 Hz), 130.39, 130.32, 130.24, 128.93, 127.71, 126.45, 126.05, 125.97, 125.67, 116.69, 114.07 (d, *J* = 21.1 Hz), 114.05 (d, *J* = 21.2 Hz), 113.28, 76.93, 76.19, 57.77, 54.50.

^{19}F NMR (377 MHz, CDCl$_3$) δ -114.90, -115.84.

HRMS (ESI) calcd for $C_{35}H_{26}F_2NO_3$ $(M+H)^+$: 546.1875, found: 546.1872.

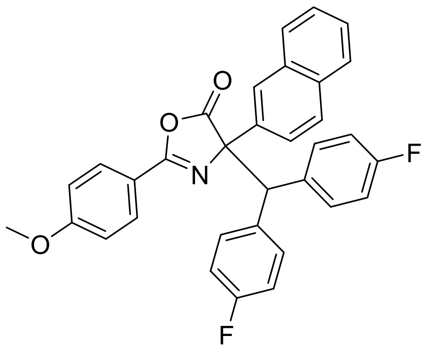

4-(bis(4-fluorophenyl)methyl)-2-(4-methoxyphenyl)-4-(naphthalen-2-yl)oxazol-5(4H)-one (10)

Following **procedure A** on 0.1 mmol scale. Purification via silica gel column chromatography (Petroleum ether/Ethyl acetate, 20:1) afforded 34.8 mg (67%) of the title compound.

^{1}H NMR (400 MHz, CDCl$_3$) δ 8.07 – 7.88 (m, 3H), 7.80 – 7.52 (m, 6H), 7.43 – 7.22 (m, 4H), 7.00 – 6.88 (m, 2H), 6.84 – 6.68 (m, 4H), 5.04 (s, 1H), 3.81 (s, 3H).

^{13}C NMR (101 MHz, CDCl$_3$) δ 177.38, 162.44, 160.98 (d, *J* = 247.0 Hz), 160.46 (d, *J* = 245.9 Hz), 159.55, 133.69 (d, *J* = 3.3 Hz), 133.28, 132.98 (d, *J* = 2.5 Hz), 131.87, 131.75, 130.38 (d, *J* = 8.1 Hz), 130.22 (d, *J* = 7.8 Hz), 128.97, 127.33, 127.20, 126.49, 125.37, 125.20, 124.86, 122.53, 116.70, 114.05 (d, *J* = 21.3 Hz), 114.03 (d, *J* = 21.1 Hz), 113.29, 77.13, 57.72, 54.50.

^{19}F NMR (377 MHz, CDCl$_3$) δ -114.87, -115.83.

HRMS (ESI) calcd for $C_{27}H_{20}F_2NO_3S$ $(M+H)^+$: 520.1719, found: 520.1720.

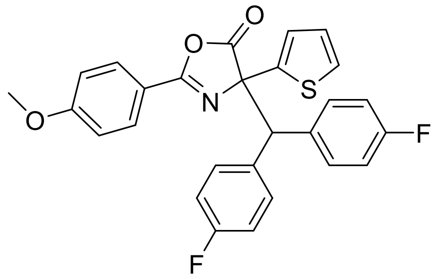

4-(bis(4-fluorophenyl)methyl)-2-(4-methoxyphenyl)-4-(thiophen-2-yl)oxazol-5(4H)-one (11)

Following **procedure A** on 0.1 mmol scale. Purification via silica gel column chromatography (Petroleum ether/Ethyl acetate, 10:1) afforded 19.5 mg (41%) of the title compound.

^{1}H NMR (400 MHz, CDCl$_3$) δ 7.92 – 7.85 (m, 2H), 7.58 – 7.52 (m, 2H), 7.29 – 7.26 (m, 1H), 7.24 – 7.20 (m, 2H), 7.18 – 7.17 (m, 1H), 7.13 – 7.10 (m, 1H), 6.93 – 6.89 (m, 2H), 6.84 – 6.73 (m, 4H), 4.81 (s, 1H), 3.82 (s, 3H).

^{13}C NMR (101 MHz, CDCl$_3$) δ 176.84, 162.42, 160.97 (d, *J* = 246.8 Hz), 160.59 (d, *J* = 245.7 Hz), 159.86, 136.31, 133.84 (d, *J* = 3.2 Hz), 132.77 (d, *J* = 2.8 Hz), 130.21 (d, *J* = 8.1 Hz), 130.09 (d, *J* = 8.0 Hz), 128.92, 125.15, 124.39, 122.01, 116.63, 114.07 (d, *J* = 21.4 Hz), 114.03 (d, *J* = 21.1 Hz), 113.26, 76.19, 76.09, 57.60, 54.49.

^{19}F NMR (377 MHz, CDCl$_3$) δ -114.87, -115.77.

HRMS (ESI) calcd for $C_{27}H_{20}F_2NO_3S$ $(M+H)^+$: 476.1126, found: 476.1127.

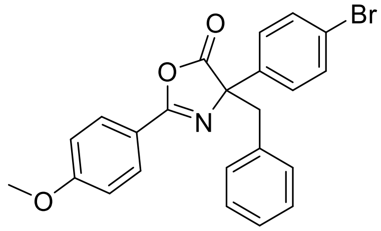

4-benzyl-4-(4-bromophenyl)-2-(4-methoxyphenyl)oxazol-5(4H)-one (12)

Following **procedure A** on 0.1 mmol scale. Purification via silica gel column chromatography (Petroleum ether/Ethyl acetate, 20:1) afforded 23.9 mg (55%) of the title compound.

^{1}H NMR (400 MHz, CDCl$_3$) δ 7.84 – 7.71 (m, 2H), 7.65 – 7.55 (m, 2H), 7.51 – 7.38 (m, 2H), 7.16 – 7.02 (m, 5H), 6.91 – 6.80 (m, 2H), 3.78 (s, 3H), 3.34 (q, *J* = 13.3 Hz, 2H).

^{13}C NMR (101 MHz, CDCl$_3$) δ 176.92, 162.20, 158.77, 136.28, 132.89, 130.70, 129.41, 128.80, 127.05, 126.64, 126.34, 121.44, 116.77, 113.11, 73.94, 54.44, 46.41.

HRMS (ESI) calcd for $C_{23}H_{19}{}^{79}BrNO_3$ $(M+H)^+$: 436.0543, found: 436.0549; calcd for $C_{23}H_{19}{}^{81}Br$ NO_3 $(M+H)^+$: 438.0522, found: 438.0528.

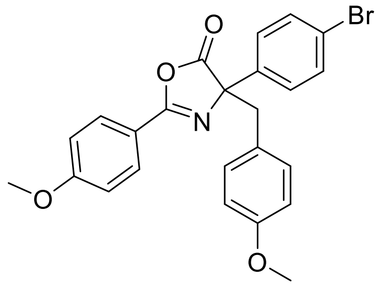

4-(4-bromophenyl)-4-(4-methoxybenzyl)-2-(4-methoxyphenyl)oxazol-5(4H)-one (13)

Following **procedure A** on 0.1 mmol scale. Purification via silica gel column chromatography (Petroleum ether/Ethyl acetate, 20:1) afforded 21.4 mg (46%) of the title compound.

^{1}H NMR (400 MHz, CDCl$_3$) δ 7.87 – 7.72 (m, 2H), 7.65 – 7.54 (m, 2H), 7.50 – 7.39 (m, 2H), 7.02 (d, *J* = 8.7 Hz, 2H), 6.94 – 6.81 (m, 2H), 6.70 – 6.54 (m, 2H), 3.79 (s, 3H), 3.63 (s, 3H), 3.28 (q, *J* = 13.5 Hz, 2H).

^{13}C NMR (101 MHz, CDCl$_3$) δ 176.98, 162.17, 158.74, 157.74, 136.31, 130.67, 130.46, 128.81, 126.62, 124.91, 121.37, 116.80, 113.11, 112.43, 74.15, 54.45, 54.07, 45.65.

HRMS (ESI) calcd for $C_{24}H_{21}{}^{79}BrNO_4$ $(M+H)^+$: 466.0648, found: 466.0655; calcd for $C_{24}H_{21}{}^{81}Br$ NO_4 $(M+H)^+$: 468.0628, found: 468.0635.

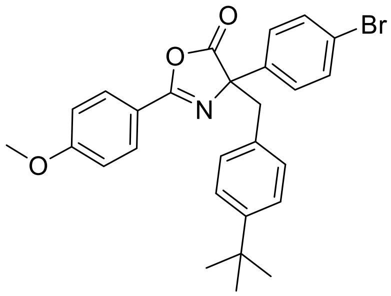

4-(4-bromophenyl)-4-(4-(tert-butyl)benzyl)-2-(4-methoxyphenyl)oxazol-5(4H)-one (14)

Following **procedure A** on 0.1 mmol scale. Purification via silica gel column chromatography (Petroleum ether/Ethyl acetate, 20:1) afforded 33.4 mg (68%) of the title compound.

^{1}H NMR (400 MHz, CDCl$_3$) δ 7.84 – 7.71 (m, 2H), 7.63 – 7.55 (m, 2H), 7.47 – 7.41 (m, 2H), 7.15 – 7.08 (m, 2H), 7.04 – 6.99 (m, 2H), 6.88 – 6.80 (m, 2H), 3.78 (s, 3H), 3.31 (q, *J* = 13.4 Hz, 2H), 1.12 (s, 9H).

^{13}C NMR (101 MHz, CDCl$_3$) δ 178.06, 163.19, 159.81, 150.21, 137.38, 131.72, 130.84, 130.12, 129.83, 127.72, 125.00, 122.44, 117.95, 114.10, 75.13, 55.50, 47.07, 34.40, 31.24.

HRMS (ESI) calcd for $C_{27}H_{27}{}^{79}BrNO_3$ $(M+H)^+$: 492.1169, found: 492.1174; calcd for $C_{27}H_{27}{}^{81}Br$ NO_3 $(M+H)^+$: 494.1148, found: 494.1158.

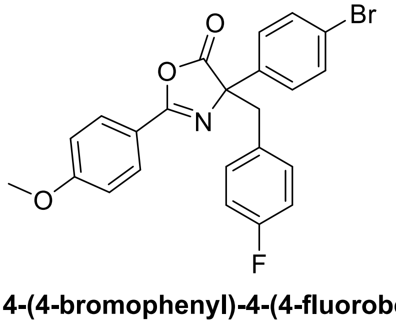

4-(4-bromophenyl)-4-(4-fluorobenzyl)-2-(4-methoxyphenyl)oxazol-5(4H)-one (15)

Following **procedure A** on 0.1 mmol scale. Purification via silica gel column chromatography (Petroleum ether/Ethyl acetate, 20:1) afforded 27.2 mg (60%) of the title compound.

^{1}H NMR (400 MHz, CDCl$_3$) δ 7.90 – 7.72 (m, 2H), 7.57 (d, *J* = 8.7 Hz, 2H), 7.44 (d, *J* = 8.7 Hz, 2H), 7.13 – 7.01 (m, 2H), 6.92 – 6.84 (m, 2H), 6.83 – 6.72 (m, 2H), 3.79 (s, 3H), 3.30 (q, *J* = 13.5 Hz, 2H).

^{13}C NMR (101 MHz, CDCl$_3$) δ 176.81, 162.33, 161.13 (d, *J* = 245.9 Hz), 158.91, 136.04, 130.99 (d, *J* = 8.1 Hz), 130.75, 128.82, 128.66 (d, *J* = 3.3 Hz), 126.59, 121.53, 116.59, 113.97 (d, *J* = 21.3 Hz), 113.20, 76.19, 73.84, 54.47, 45.50.

^{19}F NMR (377 MHz, CDCl$_3$) δ -115.01.

HRMS (ESI) calcd for $C_{23}H_{18}{}^{79}BrFNO_3$ $(M+H)^+$: 454.0449, found: 454.0454; calcd for $C_{23}H_{18}{}^{81}BrFNO_3$ $(M+H)^+$: 456.0428, found: 456.0435.

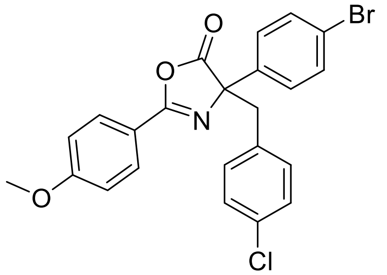

4-(4-bromophenyl)-4-(4-chlorobenzyl)-2-(4-methoxyphenyl)oxazol-5(4H)-one (16)

Following **procedure A** on 0.1 mmol scale. Purification via silica gel column chromatography (Petroleum ether/Ethyl acetate, 20:1) afforded 25.3 mg (54%) of the title compouand.

^{1}H NMR (600 MHz, CDCl$_3$) δ 7.88 (d, *J* = 8.9 Hz, 2H), 7.63 (d, *J* = 8.6 Hz, 2H), 7.52 (d, *J* = 8.6 Hz, 2H), 7.12 (dd, *J* = 29.1, 8.4 Hz, 4H), 6.95 (d, *J* = 8.9 Hz, 2H), 3.87 (s, 3H), 3.37 (q, *J* = 13.5 Hz, 2H).

^{13}C NMR (151 MHz, CDCl$_3$) δ 176.71, 162.39, 159.01, 135.98, 132.37, 131.44, 130.77, 130.74, 128.88, 127.26, 126.58, 121.59, 116.55, 113.23, 73.65, 54.48, 45.58.

HRMS (ESI) calcd for $C_{23}H_{18}{}^{79}BrClNO_3$ $(M+H)^+$: 470.0153, found: 470.0158; calcd for $C_{23}H_{18}{}^{81}BrClNO_3$ $(M+H)^+$: 472.0133, found: 472.0136.

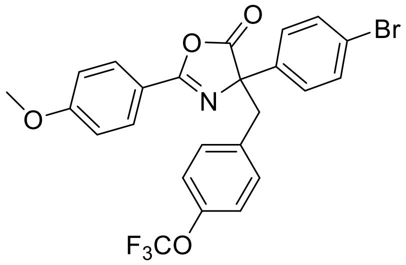

4-(4-bromophenyl)-2-(4-methoxyphenyl)-4-(4-(trifluoromethoxy)benzyl)oxazol-5(4H)-one (17)

Following **procedure A** on 0.1 mmol scale. Purification via silica gel column chromatography (Petroleum ether/Ethyl acetate, 20:1) afforded 26.5 mg (51%) of the title compouand.

^{1}H NMR (400 MHz, CDCl$_3$) δ 7.84 – 7.74 (m, 2H), 7.59 – 7.54 (m, 2H), 7.47 – 7.42 (m, 2H), 7.15 – 7.08 (m, 2H), 7.00 – 6.81 (m, 6H), 3.79 (s, 3H), 3.33 (q, *J* = 13.4 Hz, 2H).

^{13}C NMR (101 MHz, CDCl$_3$) δ 176.76, 162.36, 159.01, 135.86, 131.66, 130.79, 130.19, 129.23 (q, *J* = 245.4 Hz), 128.81, 127.76, 126.66, 126.58, 121.61, 119.48, 116.48, 113.19, 112.86, 73.66, 54.46, 45.51.

^{19}F NMR (377 MHz, CDCl$_3$) δ -57.90.

HRMS (ESI) calcd for $C_{24}H_{18}{}^{79}BrF_3NO_4$ $(M+H)^+$: 520.0366, found: 520.0368; calcd for $C_{24}H_{18}{}^{81}BrF_3NO_4$ $(M+H)^+$: 522.0345, found: 522.0347.

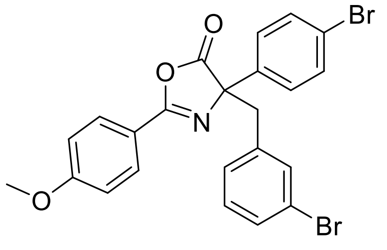

4-(3-bromobenzyl)-4-(4-bromophenyl)-2-(4-methoxyphenyl)oxazol-5(4H)-one (18)

Following **procedure A** on 0.1 mmol scale. Purification via silica gel column chromatography (Petroleum ether/Ethyl acetate, 20:1) afforded 28.2 mg (55%) of the title compouand.

^{1}H NMR (400 MHz, CDCl$_3$) δ 7.88 – 7.76 (m, 2H), 7.61 – 7.53 (m, 2H), 7.49 – 7.40 (m, 2H), 7.28 (d, *J* = 1.6 Hz, 1H), 7.24 – 7.20 (m, 1H), 7.06 – 6.93 (m, 2H), 6.92 – 6.83 (m, 2H), 3.80 (s, 3H), 3.29 (q, *J* = 13.4 Hz, 2H).

^{13}C NMR (101 MHz, CDCl$_3$) δ 176.68, 162.34, 159.04, 135.87, 135.14, 132.57, 130.77, 129.48, 128.88, 128.60, 127.88, 126.56, 121.61, 120.94, 116.51, 113.18, 73.55, 54.47, 45.80.

HRMS (ESI) calcd for $C_{23}H_{18}{}^{79}Br_2NO_3$ (M+H)$^+$: 513.9648, found: 513.9656; calcd for $C_{23}H_{18}{}^{81}Br_2NO_3$ (M+H)$^+$: 517.9607, found: 517.9609.

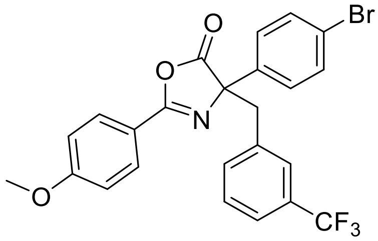

4-(4-bromophenyl)-2-(4-methoxyphenyl)-4-(3-(trifluoromethyl)benzyl)oxazol-5(4H)-one (19)

Following **procedure A** on 0.1 mmol scale. Purification via silica gel column chromatography (Petroleum ether/Ethyl acetate, 20:1) afforded 17.1 mg (34%) of the title compouand.

^{1}H NMR (400 MHz, CDCl$_3$) δ 7.89 – 7.82 (m, 2H), 7.68 – 7.62 (m, 2H), 7.53 (d, *J* = 8.7 Hz, 2H), 7.47 – 7.41 (m, 2H), 7.36 – 7.29 (m, 2H), 6.96 – 6.90 (m, 2H), 3.86 (s, 3H), 3.45 (q, *J* = 13.4 Hz, 2H).

^{13}C NMR (101 MHz, CDCl$_3$) δ 176.64, 162.39, 159.09, 135.74, 133.85, 132.55, 130.81, 128.84, 127.59, 126.56, 123.21, 123.17, 121.68, 116.35, 113.16, 73.48, 54.47, 45.92.

^{19}F NMR (377 MHz, CDCl$_3$) δ -62.76.

HRMS (ESI) calcd for $C_{24}H_{17}{}^{79}BrF_3NO_3$ (M+Na)$^+$: 526.0236, found: 526.0236; calcd for $C_{24}H_{17}{}^{81}BrF_3NO_3$ (M+Na)$^+$: 528.0216, found: 528.0226.

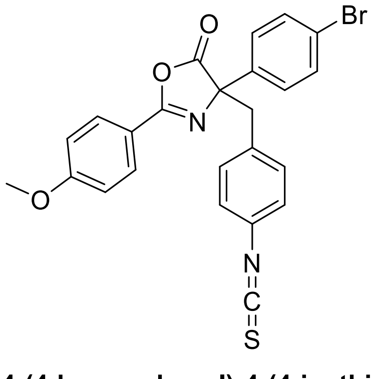

4-(4-bromophenyl)-4-(4-isothiocyanatobenzyl)-2-(4-methoxyphenyl)oxazol-5(4H)-one (20)

Following **procedure A** on 0.1 mmol scale. Purification via silica gel column chromatography (Petroleum ether/Ethyl acetate, 20:1) afforded 17.2 mg (35%) of the title compouand.

^{1}H NMR (400 MHz, CDCl$_3$) δ 7.86 – 7.76 (m, 2H), 7.61 – 7.52 (m, 2H), 7.50 – 7.40 (m, 2H), 7.13 – 7.04 (m, 2H), 7.01 – 6.93 (m, 2H), 6.92 – 6.84 (m, 2H), 3.80 (s, 3H), 3.32 (q, *J* = 13.4 Hz, 2H).

^{13}C NMR (101 MHz, CDCl$_3$) δ 176.67, 162.44, 159.02, 135.83, 132.42, 130.80, 130.60, 129.33, 128.86, 126.55, 124.40, 121.65, 116.44, 113.27, 73.61, 54.49, 45.79.

HRMS (ESI) calcd for $C_{24}H_{18}{}^{79}BrN_2O_3S$ $(M+H)^+$: 493.0216, found: 493.0221; calcd for $C_{24}H_{18}{}^{81}BrN_2O_3S$ $(M+H)^+$: 495.0196, found: 496.0201.

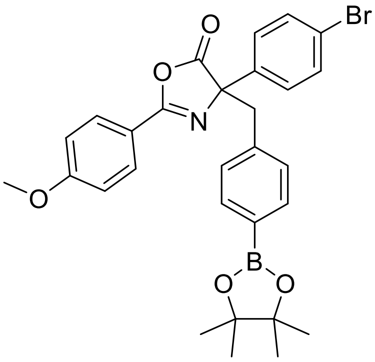

4-(4-bromophenyl)-2-(4-methoxyphenyl)-4-(4-(4,4,5,5-tetramethyl-1,3,2-dioxaborolan-2-yl)benzyl)oxazol-5(4H)-one (21)

Following **procedure A** on 0.1 mmol scale. Purification via silica gel column chromatography (Petroleum ether/Ethyl acetate, 20:1) afforded 23 mg (41%) of the title compouand.

^{1}H NMR (400 MHz, CDCl$_3$) δ 7.86 – 7.74 (m, 2H), 7.63 – 7.50 (m, 4H), 7.48 – 7.39 (m, 2H), 7.15 – 7.07 (m, 2H), 6.91 – 6.83 (m, 2H), 3.80 (s, 3H), 3.35 (d, *J* = 2.9 Hz, 2H), 1.23 (s, 12H).

^{13}C NMR (101 MHz, CDCl$_3$) δ 176.79, 162.23, 158.85, 136.11, 133.91, 133.76, 133.50, 130.69, 128.88, 128.82, 127.49, 126.62, 122.93, 113.14, 82.72, 73.74, 54.46, 46.44, 23.91, 23.82, 23.71.

HRMS (ESI) calcd for $C_{29}H_{29}B^{79}BrNO_5$ $(M+Na)^+$: 584.1214, found: 584.1221; calcd for $C_{29}H_{29}B^{81}BrNO_5$ $(M+K)^+$: 602.0933, found: 602.0931.

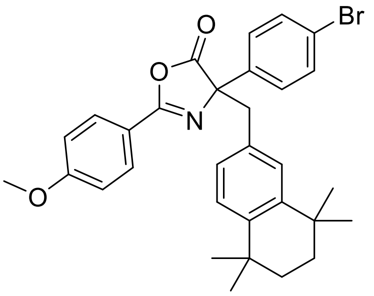

4-(4-bromophenyl)-2-(4-methoxyphenyl)-4-((5,5,8,8-tetramethyl-5,6,7,8-tetrahydronaphthalen-2-yl)methyl)oxazol-5(4H)-one (22)

Following **procedure A** on 0.1 mmol scale. Purification via silica gel column chromatography (Petroleum ether/Ethyl acetate, 20:1) afforded 34.9 mg (64%) of the title compouand.

^{1}H NMR (400 MHz, CDCl$_3$) δ 7.80 – 7.74 (m, 2H), 7.62 – 7.58 (m, 2H), 7.47 – 7.42 (m, 2H), 7.07 – 7.00 (m, 1H), 7.00 – 6.94 (m, 1H), 6.88 – 6.82 (m, 3H), 3.78 (s, 3H), 3.28 (q, *J* = 13.3 Hz, 2H), 1.50 – 1.44 (m, 4H), 1.12 – 1.09 (m, 6H), 1.07 (s, 3H), 0.83 (s, 3H).

^{13}C NMR (101 MHz, CDCl$_3$) δ 176.99, 162.13, 158.67, 143.30, 142.83, 136.41, 130.64, 129.67, 128.79, 127.58, 126.68, 126.55, 125.26, 121.35, 116.93, 112.98, 74.13, 54.45, 46.39, 33.94, 32.95, 32.90, 30.71, 30.67, 30.53, 30.46.

HRMS (ESI) calcd for $C_{31}H_{33}{}^{79}BrNO_3$ $(M+H)^+$: 546.1638, found: 546.1639.; calcd for $C_{31}H_{33}{}^{81}BrNO_3$ $(M+H)^+$: 547.1672, found: 547.1671.

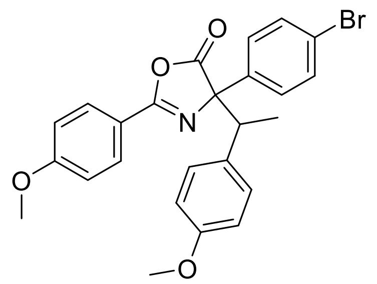

4-(4-bromophenyl)-2-(4-methoxyphenyl)-4-(1-(4-methoxyphenyl)ethyl)oxazol-5(4H)-one (23)

Following **procedure A** on 0.1 mmol scale. Purification via silica gel column chromatography (Petroleum ether/Ethyl acetate, 20:1) afforded 32.1 mg (67%) of the title compouand.

^{1}H NMR (400 MHz, CDCl$_3$) δ 8.07 – 7.98 (m, 1H), 7.93 – 7.84 (m, 1H), 7.81 – 7.72 (m, 1H), 7.54 – 7.46 (m, 1H), 7.41 – 7.35 (m, 1H), 7.17 (dd, *J* = 10.8, 8.7 Hz, 2H), 7.09 – 6.91 (m, 3H), 6.75 – 6.62 (m, 2H), 3.88 (d, *J* = 7.9 Hz, 3H), 3.72 (d, *J* = 8.9 Hz, 3H), 3.63 – 3.45 (m, 1H), 1.31 – 1.21 (m, 3H).

^{13}C NMR (101 MHz, CDCl$_3$) δ 178.90, 178.42, 163.33, 163.19, 160.00, 159.83, 158.82, 158.41, 136.47, 136.35, 134.10, 133.68, 131.95, 131.38, 130.03, 130.01, 129.99, 129.87, 128.67, 128.24, 127.75, 127.59, 118.08, 118.04, 114.24, 114.16, 113.36, 113.09, 77.59, 77.24, 55.55, 55.50, 55.13, 49.49, 49.38, 16.62, 15.62.

HRMS (ESI) calcd for $C_{25}H_{23}{}^{79}BrNO_4$ (M+H)$^+$:480.0805, found: 480.0814; calcd for $C_{25}H_{23}{}^{81}Br$ NO_4 (M+H)$^+$: 482.0785, found: 482.0794.

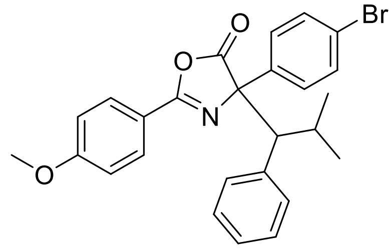

4-(4-bromophenyl)-2-(4-methoxyphenyl)-4-(2-methyl-1-phenylpropyl)oxazol-5(4H)-one (24)

Following **procedure A** on 0.1 mmol scale. Purification via silica gel column chromatography (Petroleum ether/Ethyl acetate, 20:1) afforded 33.4 mg (70%) of the title compouand.

^{1}H NMR (400 MHz, CDCl$_3$) δ 8.10 – 7.99 (m, 1H), 7.92 – 7.85 (m, 1H), 7.68 – 7.61 (m, 1H), 7.49 – 7.42 (m, 1H), 7.30 – 7.22 (m, 1H), 7.17 – 6.90 (m, 7H), 3.83 (d, *J* = 7.9 Hz, 3H), 3.36 – 3.26 (m, 1H), 2.14 – 1.85 (m, 1H), 0.96 – 0.84 (m, 3H), 0.65 – 0.45 (m, 3H).

^{13}C NMR (101 MHz, CDCl$_3$) δ 178.99, 177.28, 162.24, 159.15, 136.99, 136.70, 135.83, 130.61, 129.92, 129.02, 128.89, 127.13, 126.88, 126.86, 126.46, 126.40, 125.43, 121.28, 120.64, 117.14, 113.32, 113.22, 76.91, 76.19, 60.22, 60.13, 54.54, 54.48, 30.05, 27.97, 22.05, 21.61, 20.20, 18.93.

HRMS (ESI) calcd for $C_{26}H_{25}{}^{79}BrNO_3$ (M+H)$^+$: 478.1012, found: 478.1022; calcd for $C_{26}H_{25}{}^{81}Br$ NO_3 (M+H)$^+$: 480.0992, found: 480.1002.

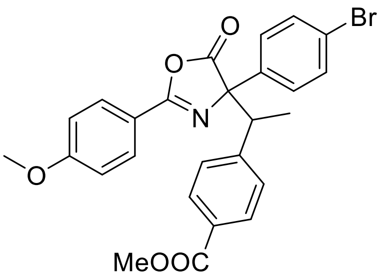

methyl 4-(1-(4-(4-bromophenyl)-2-(4-methoxyphenyl)-5-oxo-4,5-dihydrooxazol-4-yl)ethyl)benzoate (25)

Following **procedure A** on 0.1 mmol scale. Purification via silica gel column chromatography (Petroleum ether/Ethyl acetate, 10:1) afforded 29.4 mg (58%) of the title compouand.

^{1}H NMR (400 MHz, CDCl$_3$) δ 8.11 – 8.02 (m, 1H), 7.94 – 7.80 (m, 3H), 7.76 – 7.67 (m, 1H), 7.61 – 7.53 (m, 1H), 7.46 – 7.40 (m, 1H), 7.38 – 7.31 (m, 2H), 7.27 – 7.22 (m, 1H), 7.09 – 6.92 (m, 2H), 3.90 (t, *J* = 9.6 Hz, 6H), 3.72 – 3.60 (m, 1H), 1.32 (t, *J* = 6.6 Hz, 3H).

^{13}C NMR (101 MHz, CDCl$_3$) δ 178.49, 177.89, 166.96, 166.86, 163.51, 163.39, 160.26, 145.33, 144.72, 136.60, 136.45, 131.73, 131.33, 130.06, 129.88, 129.29, 129.09, 128.79, 128.06, 127.77, 122.58, 122.24, 117.77, 117.69, 114.33, 114.26, 77.23, 55.56, 55.51, 52.03, 50.04, 49.80, 16.50, 15.34.

HRMS (ESI) calcd for $C_{26}H_{22}{}^{79}BrNO_5$ $(M+Na)^+$: 530.0574, found: 530.0577; calcd for $C_{26}H_{22}{}^{81}BrNO_5$ $(M+Na)^+$: 532.0553, found: 532.0557.

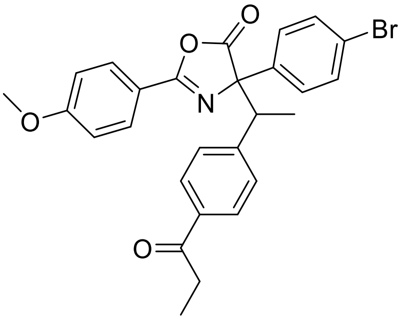

4-(4-bromophenyl)-2-(4-methoxyphenyl)-4-(1-(4-propionylphenyl)ethyl)oxazol-5(4H)-one (26)

Following **procedure A** on 0.1 mmol scale. Purification via silica gel column chromatography (Petroleum ether/Ethyl acetate, 10:1) afforded 30.8 mg (61%) of the title compouand.

^{1}H NMR (400 MHz, CDCl$_3$) δ 8.03 – 7.92 (m, 1H), 7.84 – 7.76 (m, 1H), 7.73 – 7.59 (m, 3H), 7.52 – 7.43 (m, 1H), 7.37 – 7.23 (m, 3H), 7.19 – 7.14 (m, 1H), 7.02 – 6.82 (m, 2H), 3.82 (d, *J* = 11.1 Hz, 3H), 3.63 – 3.52 (m, 1H), 2.92 – 2.80 (m, 2H), 1.26 – 1.19 (m, 3H), 1.14 – 1.05 (m, 3H).

^{13}C NMR (101 MHz, CDCl$_3$) δ 199.53, 177.50, 162.46, 159.20, 144.28, 143.64, 135.52, 135.40, 134.53, 130.70, 130.30, 129.03, 128.85, 128.22, 127.02, 126.73, 126.70, 126.50, 121.56, 121.20, 116.69, 116.59, 113.28, 113.22, 76.19, 54.54, 54.48, 48.93, 48.76, 30.66, 15.49, 14.28, 7.14, 7.10.

HRMS (ESI) calcd for $C_{27}H_{25}{}^{79}BrNO_4$$(M+H)^+$: 506.0961, found: 506.0967; calcd for $C_{27}H_{25}{}^{81}BrNO_4$ $(M+H)^+$: 508.0941, found: 508.0950.

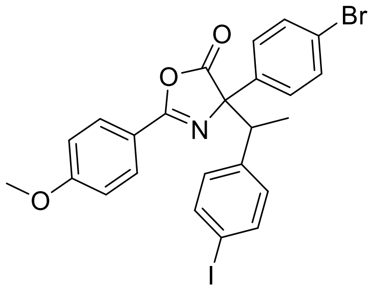

4-(4-bromophenyl)-4-(1-(4-iodophenyl)ethyl)-2-(4-methoxyphenyl)oxazol-5(4H)-one (27)

Following **procedure A** on 0.1 mmol scale. Purification via silica gel column chromatography (Petroleum ether/Ethyl acetate, 20:1) afforded 32.2 mg (56%) of the title compouand.

^{1}H NMR (400 MHz, CDCl$_3$) δ 8.08 – 7.98 (m, 1H), 7.94 – 7.85 (m, 1H), 7.73 – 7.65 (m, 1H), 7.57 – 7.45 (m, 3H), 7.43 – 7.32 (m, 2H), 7.04 – 6.94 (m, 3H), 6.92 – 6.84 (m, 1H), 3.89 (d, *J* = 6.4 Hz, 3H), 3.58 – 3.46 (m, 1H), 1.28 – 1.23 (m, 3H).

^{13}C NMR (101 MHz, CDCl$_3$) δ 177.54, 176.97, 162.43, 162.32, 159.21, 159.15, 138.60, 136.07, 135.82, 135.59, 135.47, 130.66, 130.31, 129.99, 129.91, 129.01, 128.87, 126.99, 126.75, 121.49, 116.72, 116.68, 113.26, 113.22, 92.33, 76.19, 54.53, 48.55, 48.40, 15.52, 14.34.

HRMS (ESI) calcd for $C_{24}H_{20}{}^{79}BrINO_3$ (M+H)$^+$: 575.9666, found: 575.9671; calcd for $C_{24}H_{20}{}^{81}BrINO_3$ (M+H)$^+$: 577.9645, found: 577.9646.

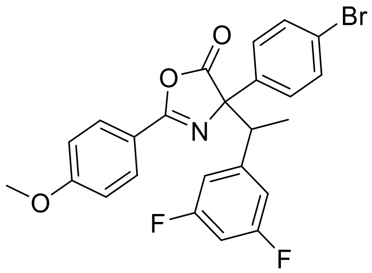

4-(4-bromophenyl)-4-(1-(3,5-difluorophenyl)ethyl)-2-(4-methoxyphenyl)oxazol-5(4H)-one (28)

Following **procedure A** on 0.1 mmol scale. Purification via silica gel column chromatography (Petroleum ether/Ethyl acetate, 20:1) afforded 23.3 mg (48%) of the title compouand.

^{1}H NMR (400 MHz, CDCl$_3$) δ 8.05 – 7.92 (m, 1H), 7.88 – 7.76 (m, 1H), 7.64 – 7.54 (m, 1H), 7.51 – 7.42 (m, 1H), 7.39 – 7.25 (m, 2H), 7.01 – 6.85 (m, 2H), 6.77 – 6.63 (m, 2H), 6.59 – 6.46 (m, 1H), 3.82 (d, *J* = 11.1 Hz, 3H), 3.56 – 3.40 (m, 1H), 1.20 – 1.15 (m, 3H).

^{13}C NMR (101 MHz, CDCl$_3$) δ 177.34, 176.78, 162.55, 162.42, 161.39 (dd, *J* = 248.0, 5.0 Hz), 161.27 (dd, *J* = 248.2, 5.5 Hz), 159.44, 143.04 (t, *J* = 9.0 Hz), 142.34 (t, *J* = 8.7 Hz), 135.28, 135.19, 130.74, 130.41, 129.09, 128.87, 126.96, 126.62, 121.65, 121.35, 116.55, 116.48, 113.31, 113.25 110.90 (dd, *J* = 25.1, 4.5 Hz), 102.00 (t, *J* = 25.2 Hz), 101.44 (t, *J* = 25.2 Hz), 76.18, 76.09, 75.77, 54.54, 54.47, 48.66, 48.28, 15.80, 14.36.

^{19}F NMR (377 MHz, CDCl$_3$) δ -109.91, -110.42.

HRMS (ESI) calcd for $C_{24}H_{19}{}^{79}BrF_2NO_3$ (M+H)$^+$: 486.0511, found: 486.0518; calcd for $C_{24}H_{19}{}^{81}BrF_2NO_3$ (M+H)$^+$: 488.0490, found: 488.0499.

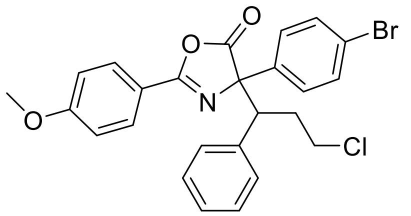

4-(4-bromophenyl)-4-(3-chloro-1-phenylpropyl)-2-(4-methoxyphenyl)oxazol-5(4H)-one (29)

Following **procedure A** on 0.1 mmol scale. Purification via silica gel column chromatography (Petroleum ether/Ethyl acetate, 20:1) afforded 35.8 mg (72%) of the title compouand.

^{1}H NMR (400 MHz, CDCl$_3$) δ 8.03 (d, *J* = 8.9 Hz, 1H), 7.89 (d, *J* = 8.9 Hz, 1H), 7.71 (d, *J* = 8.6 Hz, 1H), 7.56 (d, *J* = 8.6 Hz, 1H), 7.42 (d, *J* = 8.7 Hz, 1H), 7.32 (d, *J* = 8.7 Hz, 1H), 7.25 – 7.12 (m, 5H), 7.05 – 6.92 (m, 2H), 3.89 (d, *J* = 6.4 Hz, 3H), 3.80 – 3.63 (m, 1H), 3.40 – 3.28 (m, 1H), 3.18 – 2.99 (m, 1H), 2.52 – 2.24 (m, 1H), 2.03 – 1.91 (m, 1H).

^{13}C NMR (101 MHz, CDCl$_3$) δ 177.37, 176.47, 162.48, 162.36, 159.51, 159.12, 135.32, 135.25, 135.18, 134.59, 130.86, 130.25, 129.03, 128.89, 128.59, 127.38, 127.11, 127.08, 126.86, 126.50, 121.75, 116.79, 116.74, 113.27, 113.22, 76.07, 54.52, 54.48, 51.86, 51.39, 41.68, 41.31, 32.39, 30.87, 28.67.

HRMS (ESI) calcd for $C_{25}H_{22}{}^{79}BrClNO_3$ (M+H)$^+$: 498.0466, found: 498.0473; calcd for $C_{25}H_{22}{}^{81}BrClNO_3$ (M+H)$^+$: 500.0446, found: 500.0452.

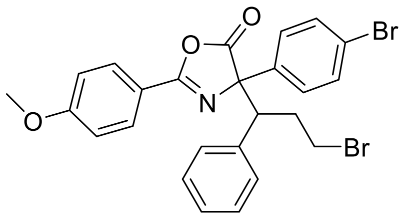

4-(3-bromo-1-phenylpropyl)-4-(4-bromophenyl)-2-(4-methoxyphenyl)oxazol-5(4H)-one (30)

Following **procedure A** on 0.1 mmol scale. Purification via silica gel column chromatography (Petroleum ether/Ethyl acetate, 20:1) afforded 41.1 mg (76%) of the title compouand.

^{1}H NMR (400 MHz, CDCl$_3$) δ 8.02 – 7.89 (m, 1H), 7.87 – 7.76 (m, 1H), 7.70 – 7.59 (m, 1H), 7.54 – 7.45 (m, 1H), 7.39 – 7.31 (m, 1H), 7.30 – 7.21 (m, 1H), 7.18 – 7.01 (m, 5H), 6.98 – 6.85 (m, 2H), 3.81 (d, *J* = 6.4 Hz, 3H), 3.74 – 3.53 (m, 1H), 3.22 – 3.08 (m, 1H), 2.92 – 2.73 (m, 1H), 2.52 – 2.23 (m, 1H), 2.01 – 1.86 (m, 1H).

^{13}C NMR (101 MHz, CDCl$_3$) δ 177.35, 176.45, 162.46, 162.34, 159.52, 159.11, 135.23, 135.20, 135.03, 134.42, 130.86, 130.25, 129.03, 128.88, 128.60, 127.40, 127.13, 127.10, 126.84, 126.52, 121.76, 121.17, 116.75, 116.69, 113.26, 113.21, 76.19, 54.52, 54.48, 52.97, 52.47, 32.44, 30.89, 30.55, 29.82.

HRMS (ESI) calcd for $C_{25}H_{22}{}^{79}Br_2NO_3$ (M+H)$^+$: 541.9961, found: 541.9962; calcd for $C_{25}H_{22}{}^{81}Br_2NO_3$ (M+H)$^+$: 545.9920, found: 545.9929.

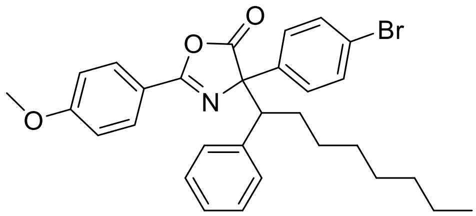

4-(4-bromophenyl)-2-(4-methoxyphenyl)-4-(1-phenyloctyl)oxazol-5(4H)-one (31)

Following **procedure A** on 0.1 mmol scale. Purification via silica gel column chromatography (Petroleum ether/Ethyl acetate, 20:1) afforded 22.4 mg (42%) of the title compouand.

^{1}H NMR (400 MHz, CDCl$_3$) δ 8.08 – 7.92 (m, 1H), 7.86 – 7.74 (m, 1H), 7.67 – 7.57 (m, 1H), 7.52 – 7.41 (m, 1H), 7.35 – 7.19 (m, 2H), 7.17 – 6.99 (m, 5H), 6.97 – 6.85 (m, 2H), 3.81 (d, *J* = 9.4 Hz, 3H), 3.44 – 3.16 (m, 1H), 1.47 – 0.79 (m, 12H), 0.78 – 0.65 (m, 3H).

^{13}C NMR (101 MHz, CDCl$_3$) δ 178.14, 177.04, 162.30, 162.16, 159.03, 158.66, 136.99, 136.34, 136.01, 135.85, 130.61, 130.04, 128.97, 128.82, 128.64, 128.57, 127.13, 126.99, 126.79, 126.68, 126.48, 125.83, 117.03, 113.21, 113.12, 76.90, 76.66, 76.19, 55.07, 54.71, 54.52, 54.45, 30.66, 29.50, 28.23, 28.07, 27.97, 27.90, 27.61, 26.30, 21.52, 13.01.

HRMS (ESI) calcd for $C_{30}H_{33}{}^{79}BrNO_3$ $(M+H)^+$: 534.1638, found: 534.1644; calcd for $C_{30}H_{33}{}^{81}Br$ NO_3 $(M+H)^+$: 536.1618, found: 536.1628.

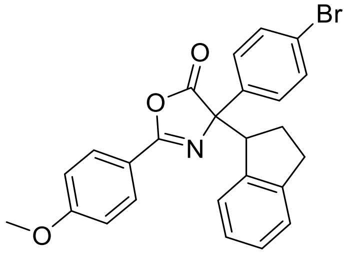

4-(4-bromophenyl)-4-(2,3-dihydro-1H-inden-1-yl)-2-(4-methoxyphenyl)oxazol-5(4H)-one (32)

Following **procedure A** on 0.1 mmol scale. Purification via silica gel column chromatography (Petroleum ether/Ethyl acetate, 20:1) afforded 36.4 mg (79%) of the title compouand.

^{1}H NMR (400 MHz, CDCl$_3$) δ 7.97 – 7.81 (m, 1H), 7.73 – 7.64 (m, 1H), 7.59 (d, *J* = 8.6 Hz, 1H), 7.54 – 7.35 (m, 3H), 7.17 – 6.75 (m, 6H), 6.28 (d, *J* = 7.6 Hz, 1H), 4.00 – 3.92 (m, 1H), 3.78 (d, *J* = 20.7 Hz, 3H), 3.08 – 2.80 (m, 1H), 2.74 – 2.61 (m, 1H), 2.23 – 1.75 (m, 2H).

^{13}C NMR (101 MHz, CDCl$_3$) δ 177.83, 177.45, 162.33, 162.04, 159.12, 158.66, 144.88, 144.41, 139.34, 138.85, 136.13, 135.51, 130.59, 130.51, 129.06, 128.61, 127.43, 127.14, 126.82, 126.61, 125.10, 124.73, 124.53, 123.49, 123.23, 121.49, 121.38, 116.88, 116.77, 113.21, 112.99, 76.69, 76.66, 76.19, 54.50, 54.40, 53.94, 53.33, 30.77, 30.71, 27.00, 26.65.

HRMS (ESI) calcd for $C_{25}H_{21}{}^{79}BrNO_3$ $(M+H)^+$: 462.0699, found: 462.0702; calcd for $C_{25}H_{21}{}^{81}Br$ NO_3 $(M+H)^+$: 464.0679, found: 464.0687.

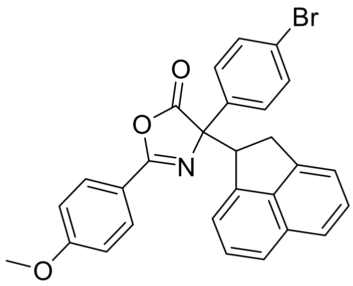

4-(4-bromophenyl)-4-(1,2-dihydroacenaphthylen-1-yl)-2-(4-methoxyphenyl)oxazol-5(4H)-one (33)

Following **procedure A** on 0.1 mmol scale. Purification via silica gel column chromatography (Petroleum ether/Ethyl acetate, 20:1) afforded 39.8 mg (80%) of the title compouand.

^{1}H NMR (400 MHz, CDCl$_3$) δ 7.79 (d, *J* = 8.9 Hz, 1H), 7.66 – 7.58 (m, 2H), 7.57 – 7.42 (m, 5H), 7.39 – 7.08 (m, 5H), 6.90 – 6.48 (m, 3H), 4.62 – 4.45 (m, 1H), 3.74 (d, *J* = 29.8 Hz, 3H), 3.46 – 3.33 (m, 1H), 3.26 – 3.12 (m, 1H).

^{13}C NMR (101 MHz, CDCl$_3$) δ 177.73, 176.47, 162.37, 162.09, 159.54, 159.16, 142.01, 141.44, 141.02, 138.27, 135.84, 135.40, 130.80, 130.31, 130.27, 129.12, 128.76, 127.58, 127.12, 126.81, 126.46, 126.34, 123.14, 121.76, 121.71, 121.54, 121.38, 119.90, 118.14, 118.09, 118.00, 116.63, 116.47, 113.17, 112.89, 76.19, 54.47, 54.36, 51.67, 50.97, 33.01, 32.36.

HRMS (ESI) calcd for $C_{28}H_{20}{}^{79}BrNO_3$ (M+Na)$^+$: 520.0519, found: 520.0518; calcd for $C_{28}H_{20}{}^{81}BrNO_3$ (M+Na)$^+$: 522.0498, found: 522.0499.

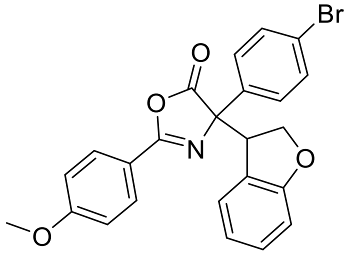

4-(4-bromophenyl)-4-(2,3-dihydrobenzofuran-3-yl)-2-(4-methoxyphenyl)oxazol-5(4H)-one (34)

Following **procedure A** on 0.1 mmol scale. Purification via silica gel column chromatography (Petroleum ether/Ethyl acetate, 20:1) afforded 23.6 mg (51%) of the title compouand.

^{1}H NMR (400 MHz, CDCl$_3$) δ 7.95 – 7.83 (m, 1H), 7.77 – 7.67 (m, 1H), 7.63 – 7.38 (m, 4H), 7.13 – 6.78 (m, 4H), 6.72 – 6.59 (m, 2H), 4.60 – 4.08 (m, 3H), 3.79 (d, *J* = 19.7 Hz, 3H).

^{13}C NMR (101 MHz, CDCl$_3$) δ 176.84, 162.53, 162.32, 159.99, 134.54, 133.97, 130.94, 130.79, 129.17, 128.90, 128.77, 127.34, 127.08, 124.93, 123.20, 122.92, 122.46, 122.01, 121.97, 119.38, 119.10, 113.27, 113.04, 108.87, 108.74, 76.19, 74.78, 74.74, 71.09, 70.75, 54.51, 54.43, 51.04, 50.44.

HRMS (ESI) calcd for $C_{24}H_{19}{}^{79}BrNO_4$ (M+H)$^+$: 464.0492, found: 464.0498; calcd for $C_{24}H_{19}{}^{81}BrNO_4$ (M+H)$^+$: 466.0472, found: 466.0478.

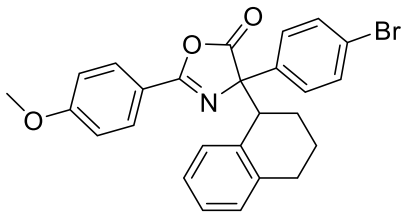

4-(4-bromophenyl)-2-(4-methoxyphenyl)-4-(1,2,3,4-tetrahydronaphthalen-1-yl)oxazol-5(4H)-one (35)

Following **procedure A** on 0.1 mmol scale. Purification via silica gel column chromatography (Petroleum ether/Ethyl acetate, 20:1) afforded 30.9 mg (65%) of the title compouand.

^{1}H NMR (400 MHz, CDCl$_3$) δ 7.94 – 7.81 (m, 1H), 7.68 – 7.56 (m, 2H), 7.53 – 7.31 (m, 3H), 7.16 – 6.48 (m, 6H), 3.83 – 3.65 (m, 4H), 2.90 – 2.50 (m, 2H), 1.94 – 1.63 (m, 2H), 1.63 – 1.52 (m, 1H), 1.48 – 1.35 (m, 1H).

^{13}C NMR (101 MHz, CDCl$_3$) δ 178.99, 177.23, 162.26, 161.95, 158.75, 157.86, 139.28, 138.65, 136.07, 135.88, 132.52, 132.34, 130.67, 130.48, 129.21, 128.95, 128.51, 127.82, 127.72, 127.60, 127.46, 127.14, 125.99, 125.65, 124.38, 123.60, 121.34, 121.30, 116.97, 116.81, 113.20, 112.96, 78.10, 78.03, 76.19, 54.48, 54.38, 46.52, 45.78, 28.49, 27.53, 24.33, 23.49, 20.08, 18.49.

HRMS (ESI) calcd for $C_{26}H_{23}{}^{79}BrNO_3$ (M+H)$^+$: 476.0856, found: 476.0862; calcd for $C_{26}H_{23}{}^{81}Br$ NO_3 (M+H)$^+$: 478.0835, found: 478.0841.

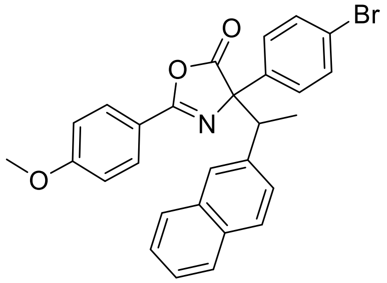

4-(4-bromophenyl)-2-(4-methoxyphenyl)-4-(1-(naphthalen-2-yl)ethyl)oxazol-5(4H)-one (36)

Following **procedure A** on 0.1 mmol scale. Purification via silica gel column chromatography (Petroleum ether/Ethyl acetate, 20:1) afforded 37.4 mg (75%) of the title compouand.

^{1}H NMR (400 MHz, CDCl$_3$) δ 8.03 – 7.95 (m, 1H), 7.81 – 7.75 (m, 1H), 7.72 – 7.45 (m, 6H), 7.40 – 7.20 (m, 5H), 6.97 – 6.78 (m, 2H), 3.80 (d, J = 18.2 Hz, 3H), 3.76 – 3.61 (m, 1H), 1.30 (t, J = 7.3 Hz, 3H).

^{13}C NMR (101 MHz, CDCl$_3$) δ 177.80, 177.13, 162.33, 162.14, 159.15, 158.98, 136.56, 135.95, 135.69, 132.02, 131.93, 131.66, 131.39, 130.65, 130.19, 129.00, 128.83, 127.30, 127.09, 126.92, 126.90, 126.85, 126.83, 126.42, 126.20, 125.56, 124.82, 124.75, 124.70, 124.56, 121.40, 121.01, 116.88, 113.22, 113.07, 76.81, 76.50, 76.19, 54.51, 54.43, 49.08, 15.81, 14.66.

HRMS (ESI) calcd for $C_{28}H_{23}{}^{79}BrNO_3$ (M+H)$^+$: 500.0856, found: 500.0862; calcd for $C_{28}H_{23}{}^{81}Br$ NO_3 (M+Na)$^+$: 524.0655, found: 524.0663.

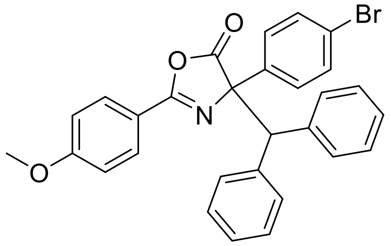

4-benzhydryl-4-(4-bromophenyl)-2-(4-methoxyphenyl)oxazol-5(4H)-one (37)

Following **procedure A** on 0.1 mmol scale. Purification via silica gel column chromatography (Petroleum ether/Ethyl acetate, 20:1) afforded 38.3 mg (75%) of the title compouand.

^{1}H NMR (400 MHz, CDCl$_3$) δ 7.93 – 7.83 (m, 2H), 7.63 – 7.47 (m, 4H), 7.32 – 7.24 (m, 4H), 7.14 – 6.99 (m, 6H), 6.90 (d, *J* = 8.9 Hz, 2H), 4.84 (s, 1H), 3.80 (s, 3H).

^{13}C NMR (101 MHz, CDCl$_3$) δ 177.30, 162.32, 159.38, 137.73, 136.93, 135.39, 130.38, 128.92, 128.75, 128.73, 127.17, 127.14, 127.11, 126.39, 125.73, 121.18, 116.78, 113.18, 76.80, 59.76, 54.47.

HRMS (ESI) calcd for $C_{29}H_{23}{}^{79}BrNO_3$ $(M+H)^+$: 512.0856, found: 512.0853; calcd for $C_{29}H_{23}{}^{81}Br$ NO_3 $(M+H)^+$: 514.0835, found: 514.0842.

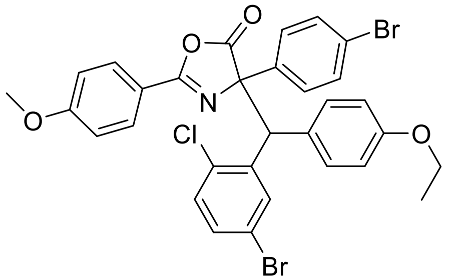

4-((5-bromo-2-chlorophenyl)(4-ethoxyphenyl)methyl)-4-(4-bromophenyl)-2-(4-methoxyphenyl)oxazol-5(4H)-one (38)

Following **procedure A** on 0.1 mmol scale. Purification via silica gel column chromatography (Petroleum ether/Ethyl acetate, 20:1) afforded 46.7 mg (70%) of the title compouand.

^{1}H NMR (400 MHz, CDCl$_3$) δ 8.70 (d, *J* = 2.4 Hz, 1H), 8.00 (dd, *J* = 33.9, 8.9 Hz, 2H), 7.75 (d, *J* = 2.3 Hz, 1H), 7.64 – 7.52 (m, 2H), 7.50 – 7.34 (m, 3H), 7.24 – 7.14 (m, 2H), 7.06 – 6.97 (m, 3H), 6.76 – 6.63 (m, 2H), 5.55 (d, *J* = 64.2 Hz, 1H), 3.96 – 3.84 (m, 5H), 1.38 – 1.29 (m, 3H).

^{13}C NMR (101 MHz, CDCl$_3$) δ 176.84, 176.23, 162.61, 162.50, 160.11, 159.81, 157.30, 156.93, 137.84, 137.57, 135.20, 134.88, 132.97, 132.52, 132.46, 131.78, 130.69, 130.49, 130.45, 130.28, 130.05, 129.92, 129.84, 129.56, 129.15, 129.07, 128.61, 127.15, 126.79, 125.70, 121.44, 119.15, 118.98, 116.45, 116.41, 113.32, 113.26, 113.00, 76.60, 76.19, 76.13, 62.18, 54.51, 53.53, 52.57, 13.77, 13.67.

HRMS (ESI) calcd for $C_{31}H_{25}{}^{79}Br_2ClNO_4(M+H)^+$: 667.9833, found: 667.9831; calcd for $C_{31}H_{25}{}^{81}Br_2ClNO_4(M+H)^+$: 671.9792, found: 671.9797.

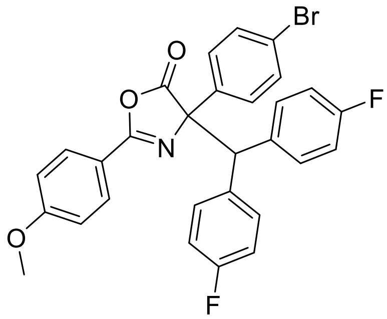

4-(bis(4-fluorophenyl)methyl)-4-(4-bromophenyl)-2-(4-methoxyphenyl)oxazol-5(4H)-one (39)

Following **procedure A** on 0.1 mmol scale. Purification via silica gel column chromatography (Petroleum ether/Ethyl acetate, 20:1) afforded 39.9 mg (73%) of the title compouand.

^{1}H NMR (400 MHz, $CDCl_3$) δ 7.95 – 7.82 (m, 2H), 7.58 – 7.45 (m, 4H), 7.34 – 7.26 (m, 2H), 7.24 – 7.18 (m, 2H), 6.94 – 6.88 (m, 2H), 6.84 – 6.71 (m, 4H), 4.82 (s, 1H), 3.81 (s, 3H).

^{13}C NMR (101 MHz, $CDCl_3$) δ 177.05, 162.52, 160.99 (d, *J* = 246.8 Hz), 160.58 (d, *J* = 245.9 Hz), 159.67, 135.03, 133.39 (d, *J* = 3.4 Hz), 132.56 (d, *J* = 3.3 Hz), 130.53, 130.26 (d, *J* = 9.1 Hz), 130.17 (d, *J* = 8.4 Hz), 128.95, 126.99, 121.40, 116.40, 114.18 (d, *J* = 21.1 Hz), 114.09 (d, *J* = 21.4 Hz), 113.30, 76.63, 76.19, 58.02, 54.50.

^{19}F NMR (377 MHz, $CDCl_3$) δ -114.65, -115.44.

HRMS (ESI) calcd for $C_{29}H_{21}{}^{79}BrF_2NO_3$ $(M+H)^+$: 548.0667, found: 548.0674; calcd for $C_{29}H_{21}{}^{81}BrF_2NO_3$ $(M+H)^+$: 550.0647, found: 550.0655.

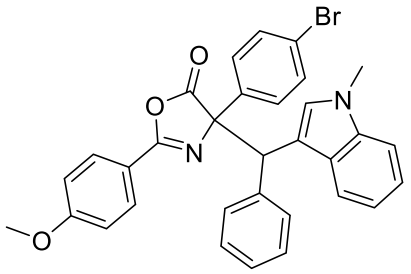

4-(4-bromophenyl)-2-(4-methoxyphenyl)-4-((1-methyl-1H-indol-3-yl)(phenyl)methyl)oxazol-5(4H)-one (40)

Following **procedure A** on 0.1 mmol scale. Purification via silica gel column chromatography (Petroleum ether/Ethyl acetate, 10:1) afforded 38.2 mg (70%) of the title compouand.

^{1}H NMR (600 MHz, $CDCl_3$) δ 7.98 – 7.84 (m, 2H), 7.73 – 7.49 (m, 3H), 7.48 – 7.39 (m, 1H), 7.35 – 7.26 (m, 4H), 7.16 – 6.83 (m, 9H), 5.17 (d, *J* = 62.1 Hz, 1H), 3.81 (d, *J* = 7.6 Hz, 3H), 3.65 (s, 2H), 3.46 (s, 1H).

^{13}C NMR (151 MHz, $CDCl_3$) δ 177.18, 162.25, 159.65, 159.54, 138.62, 136.97, 135.85, 135.69, 135.18, 135.02, 130.44, 130.25, 128.94, 128.88, 128.86, 128.51, 127.47, 127.15, 126.99, 126.87, 126.84, 126.73, 126.35, 126.24, 125.45, 121.12, 121.05, 120.71, 120.35, 118.29, 117.92, 117.83, 117.42, 117.14, 116.89, 113.20, 113.15, 110.93, 110.49, 107.94, 77.51, 76.96, 54.48, 54.47, 51.21, 50.53, 31.84, 31.68.

HRMS (ESI) calcd for $C_{32}H_{26}{}^{79}BrN_2O_3$ $(M+H)^+$: 565.1121, found: 565.1122; calcd for $C_{32}H_{26}{}^{81}BrN_2O_3$ $(M+H)^+$: 576.1101, found: 576.1107.

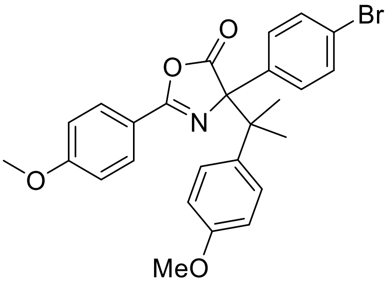

4-(4-bromophenyl)-2-(4-methoxyphenyl)-4-(2-(4-methoxyphenyl)propan-2-yl)oxazol-5(4H)-one (41)

Following **procedure A** on 0.1 mmol scale. Purification via silica gel column chromatography (Petroleum ether/Ethyl acetate, 20:1) afforded 30.1 mg (61%) of the title compouand.

^{1}H NMR (400 MHz, $CDCl_3$) δ 7.87 – 7.70 (m, 2H), 7.62 – 7.45 (m, 2H), 7.41 – 7.30 (m, 2H), 7.17 – 7.08 (m, 2H), 6.89 – 6.82 (m, 2H), 6.66 – 6.56 (m, 2H), 3.78 (s, 3H), 3.65 (s, 3H), 1.53 – 1.34 (m, 6H).

^{13}C NMR (101 MHz, $CDCl_3$) δ 176.37, 161.98, 157.82, 157.24, 133.50, 133.36, 129.51, 129.00, 128.66, 128.29, 121.26, 117.08, 113.03, 111.46, 78.11, 54.42, 54.09, 44.53, 22.94, 22.52.

HRMS (ESI) calcd for $C_{26}H_{25}{}^{79}BrNO_4$ (M+H)$^+$: 494.0961, found: 494.0963; calcd for $C_{26}H_{25}{}^{81}Br$ NO_4 (M+H)$^+$: 496.0941, found: 496.0951.

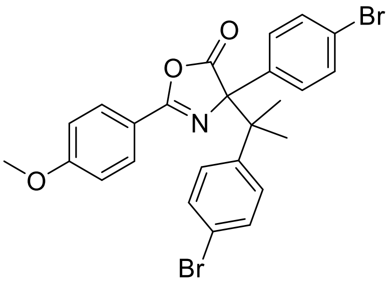

4-(4-bromophenyl)-4-(2-(4-bromophenyl)propan-2-yl)-2-(4-methoxyphenyl)oxazol-5(4H)-one (42)

Following **procedure A** on 0.1 mmol scale. Purification via silica gel column chromatography (Petroleum ether/Ethyl acetate, 20:1) afforded 30.1 mg (56%) of the title compouand.

^{1}H NMR (400 MHz, $CDCl_3$) δ 7.85 – 7.73 (m, 2H), 7.55 – 7.44 (m, 2H), 7.39 – 7.32 (m, 2H), 7.20 (s, 2H), 7.10 – 7.03 (m, 2H), 6.91 – 6.82 (m, 2H), 3.80 (s, 3H), 1.41 (d, J = 12.1 Hz, 6H).

^{13}C NMR (101 MHz, $CDCl_3$) δ 176.08, 162.16, 158.08, 140.47, 133.06, 129.62, 129.24, 129.05, 128.95, 128.71, 121.51, 120.16, 116.77, 113.14, 77.61, 76.19, 54.45, 44.73, 22.65, 22.39.

HRMS (ESI) calcd for $C_{25}H_{22}{}^{79}Br_2NO_3$ (M+H)$^+$: 541.9961, found: 541.9966; calcd for $C_{25}H_{22}{}^{81}$ Br_2NO_3 (M+H)$^+$: 545.9920, found: 545.9930.

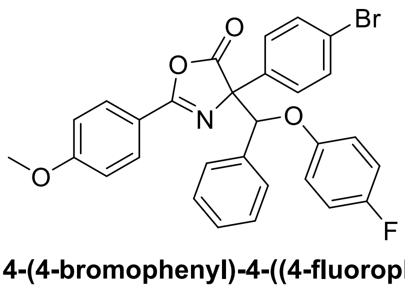

4-(4-bromophenyl)-4-((4-fluorophenoxy)(phenyl)methyl)-2-(4-methoxyphenyl)oxazol-5(4H)-one (43)

Following **procedure A** on 0.1 mmol scale. Purification via silica gel column chromatography

(Petroleum ether/Ethyl acetate, 20:1) afforded 38.7 mg (71%) of the title compouand.

^{1}H NMR (400 MHz, CDCl$_3$) δ 8.07 – 8.00 (m, 1H), 7.93 – 7.84 (m, 1H), 7.50 – 7.31 (m, 4H), 7.18 – 6.85 (m, 7H), 6.81 – 6.47 (m, 4H), 5.37 (d, *J* = 5.7 Hz, 1H), 3.82 (d, *J* = 13.3 Hz, 3H).

^{13}C NMR (101 MHz, CDCl$_3$) δ 176.67, 174.41, 162.45, 162.40, 159.95, 156.91 (d, *J* = 240.8 Hz), 153.03 (d, *J* = 2.6 Hz), 152.32 (d, *J* = 2.1 Hz), 133.13, 133.10, 133.00, 132.80, 130.43, 130.33, 129.22, 129.05, 127.92, 127.66, 127.60, 127.51, 127.38, 127.25, 126.97, 126.71, 121.92, 121.81, 117.09 (d, *J* = 7.9 Hz), 116.76 (d, *J* = 8.1 Hz), 116.61, 114.73 (d, *J* = 23.2 Hz), 114.70 (d, *J* = 23.1 Hz), 113.23, 113.19, 85.87, 85.64, 76.85, 76.71, 76.19, 54.54, 54.48.

^{19}F NMR (377 MHz, CDCl$_3$) δ -121.89, -122.08.

HRMS (ESI) calcd for $C_{29}H_{22}{}^{79}BrFNO_4$ (M+H)$^+$: 546.0711, found: 546.0705; calcd for $C_{29}H_{22}{}^{81}BrFNO_4$ (M+H)$^+$: 548.0690, found: 548.0698.

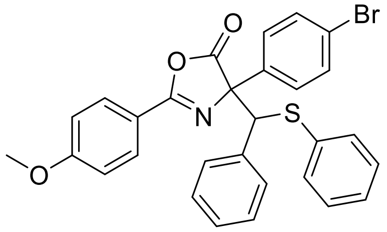

4-(4-bromophenyl)-2-(4-methoxyphenyl)-4-(phenyl(phenylthio)methyl)oxazol-5(4H)-one (44)

Following **procedure A** on 0.1 mmol scale. Purification via silica gel column chromatography (Petroleum ether/Ethyl acetate, 20:1) afforded 30.4 mg (56%) of the title compouand.

^{1}H NMR (400 MHz, CDCl$_3$) δ 8.25 – 8.09 (m, 1H), 7.97 – 7.88 (m, 1H), 7.75 – 7.68 (m, 1H), 7.51 – 7.47 (m, 1H), 7.42 – 7.33 (m, 2H), 7.29 – 7.26 (m, 1H), 7.26 – 7.21 (m, 2H), 7.20 – 6.89 (m, 9H), 4.70 (d, *J* = 79.5 Hz, 1H), 3.90 (d, *J* = 14.2 Hz, 3H).

^{13}C NMR (101 MHz, CDCl$_3$) δ 177.07, 175.42, 162.61, 162.46, 159.81, 136.82, 135.62, 135.02, 134.65, 134.02, 133.04, 132.07, 130.80, 130.47, 130.24, 129.41, 129.13, 128.56, 128.51, 127.95, 127.72, 127.27, 127.24, 127.02, 126.88, 126.77, 126.50, 126.18, 121.89, 116.46, 113.29, 113.19, 77.04, 77.01, 76.19, 62.93, 62.51, 54.55, 54.49.

HRMS (ESI) calcd for $C_{29}H_{23}{}^{79}BrNO_3S$ (M+H)$^+$: 544.0577, found: 544.0582; calcd for $C_{29}H_{23}{}^{81}BrNO_3S$ (M+H)$^+$: 546.0556, found: 546.0557.

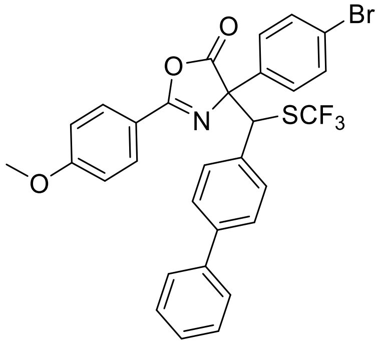

4-([1,1'-biphenyl]-4-yl((trifluoromethyl)thio)methyl)-4-(4-bromophenyl)-2-(4-methoxyphenyl)oxazol-5(4H)-one (45)

Following **procedure A** on 0.1 mmol scale. Purification via silica gel column chromatography (Petroleum ether/Ethyl acetate, 20:1) afforded 19.6 mg (32%) of the title compouand.

^{1}H NMR (400 MHz, CDCl$_3$) δ 8.11 – 8.02 (m, 1H), 7.92 – 7.80 (m, 1H), 7.75 – 7.65 (m, 1H),

7.54 – 7.42 (m, 3H), 7.40 – 7.21 (m, 9H), 7.01 – 6.96 (m, 1H), 6.93 – 6.84 (m, 1H), 4.86 (d, *J* = 11.9 Hz, 1H), 3.83 (d, *J* = 15.4 Hz, 3H).

^{13}C NMR (101 MHz, CDCl$_3$) δ 175.77, 174.42, 163.00, 162.82, 161.56, 160.62, 140.57, 139.84, 139.15, 139.04, 137.08 (q, J = 216.8 Hz), 134.63, 134.35, 133.69, 133.58, 130.88, 130.54, 129.55, 129.27, 128.56, 128.49, 127.99, 127.74, 127.73, 127.09, 126.92, 126.67, 126.57, 126.49, 126.35, 126.01, 125.95, 125.92, 125.72, 122.51, 122.04, 116.13, 115.93, 113.43, 113.39, 76.19, 54.60, 54.53, 28.41, 25.88.

^{19}F NMR (377 MHz, CDCl$_3$) δ -40.28, -40.96.

HRMS (ESI) calcd for $C_{30}H_{22}{}^{79}BrF_3NO_3S$ (M+H)$^+$: 612.0450, found: 612.0455; calcd for $C_{30}H_{22}$ $^{81}BrF_3NO_3S$ (M+H)$^+$: 614.0430, found: 614.0432.

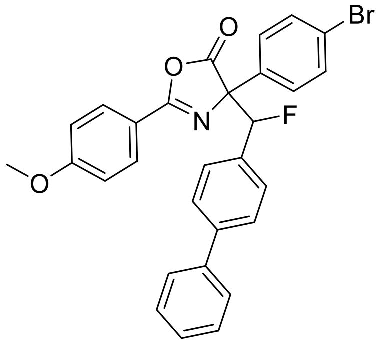

4-([1,1'-biphenyl]-4-ylfluoromethyl)-4-(4-bromophenyl)-2-(4-methoxyphenyl)oxazol-5(4H)-one (46)

Following **procedure A** on 0.1 mmol scale. Purification via silica gel column chromatography (Petroleum ether/Ethyl acetate, 20:1) afforded 21.2 mg (40%) of the title compouand.

^{1}H NMR (400 MHz, CDCl$_3$) δ 8.04 – 7.97 (m, 1H), 7.92 – 7.86 (m, 1H), 7.61 – 7.24 (m, 12H), 7.14 – 7.08 (m, 1H), 6.97 – 6.88 (m, 2H), 5.96 – 5.72 (m, 1H), 3.84 – 3.79 (m, 3H).

^{13}C NMR (101 MHz, CDCl$_3$) δ 175.61, 162.60, 162.55, 160.58, 160.40, 141.06, 140.86, 139.27, 139.16, 132.24 (d, *J* = 2.2 Hz), 131.96 (d, *J* = 4.9 Hz), 131.01 (d, *J* = 20.3 Hz), 130.68, 130.61, 129.27, 129.13, 127.76, 127.61, 127.29, 127.22, 126.88 (d, *J* = 6.9 Hz), 126.61, 126.56, 126.04, 125.44, 125.31, 116.76, 116.33, 113.26, 113.24, 95.62 (d, *J* = 186.1 Hz), 95.30 (d, *J* = 190.3 Hz), 76.05 (d, *J* = 27.7 Hz), 54.53, 54.49.

^{19}F NMR (377 MHz, CDCl$_3$) δ -176.02, -183.85.

HRMS (ESI) calcd for $C_{29}H_{22}{}^{79}BrFNO_3$ (M+H)$^+$: 530.0762, found: 530.0769; calcd for $C_{29}H_{22}{}^{81}$ $BrFNO_3$ (M+H)$^+$: 532.0741, found: 532.0732.

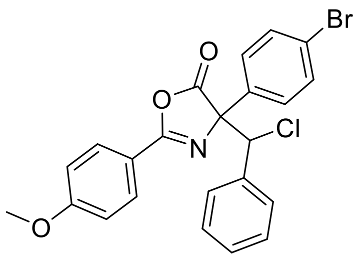

4-(4-bromophenyl)-4-(chloro(phenyl)methyl)-2-(4-methoxyphenyl)oxazol-5(4H)-one (47)

Following **procedure A** on 0.1 mmol scale. Purification via silica gel column chromatography (Petroleum ether/Ethyl acetate, 20:1) afforded 23.5 mg (50%) of the title compouand.

^{1}H NMR (400 MHz, CDCl$_3$) δ 8.10 – 8.01 (m, 1H), 7.93 – 7.83 (m, 1H), 7.61 – 7.53 (m, 1H), 7.48 – 7.21 (m, 5H), 7.18 – 7.10 (m, 3H), 7.00 – 6.94 (m, 1H), 6.93 – 6.87 (m, 1H), 5.33 (d, *J* = 7.6 Hz, 1H), 3.82 (d, *J* = 12.0 Hz, 3H).
^{13}C NMR (101 MHz, CDCl$_3$) δ 175.85, 174.26, 162.66, 162.60, 160.58, 160.23, 134.05, 133.95, 133.71, 133.29, 130.61, 130.51, 129.36, 129.17, 128.29, 128.26, 127.86, 127.32, 127.00, 126.96, 126.82, 122.23, 122.09, 116.25, 113.29, 113.25, 77.13, 77.10, 76.19, 66.45, 65.66, 54.55, 54.51.
HRMS (ESI) calcd for $C_{23}H_{18}{}^{79}BrClNO_3$ (M+H)$^+$: 470.0153, found: 470.0161; calcd for $C_{23}H_{18}{}^{81}BrClNO_3$ (M+H)$^+$: 472.0133, found: 472.0131.

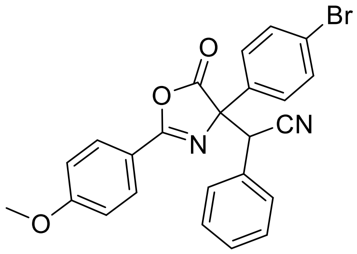

2-(4-(4-bromophenyl)-2-(4-methoxyphenyl)-5-oxo-4,5-dihydrooxazol-4-yl)-2-phenylacetonitrile (48)

Following **procedure A** on 0.1 mmol scale. Purification via silica gel column chromatography (Petroleum ether/Ethyl acetate, 20:1) afforded 24.8 mg (54%) of the title compouand.
^{1}H NMR (600 MHz, CDCl$_3$) δ 7.96 – 7.70 (m, 3H), 7.57 – 7.45 (m, 2H), 7.41 – 7.25 (m, 2H), 7.22 – 7.09 (m, 4H), 6.94 – 6.86 (m, 2H), 4.41 (d, *J* = 66.4 Hz, 1H), 3.81 (d, *J* = 8.2 Hz, 3H).
^{13}C NMR (151 MHz, CDCl$_3$) δ 174.50, 174.14, 162.85, 162.80, 160.75, 160.48, 133.11, 132.08, 131.18, 130.78, 129.38, 129.24, 128.62, 128.50, 128.17, 128.14, 127.72, 127.43, 127.18, 126.99, 123.01, 122.65, 115.97, 115.80, 115.69, 115.61, 113.30, 74.47, 73.95, 54.54, 54.52, 47.58, 46.77.
HRMS (ESI) calcd for $C_{24}H_{18}{}^{79}BrN_2O_3$ (M+H)$^+$: 461.0495, found: 461.0495; calcd for $C_{24}H_{18}{}^{81}BrN_2O_3$ (M+H)$^+$: 463.0475, found: 463.0476.

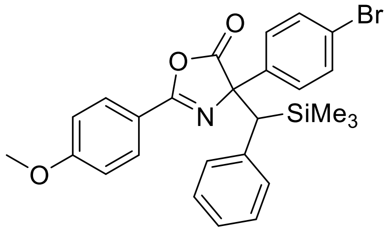

4-(4-bromophenyl)-2-(4-methoxyphenyl)-4-(phenyl(trimethylsilyl)methyl)oxazol-5(4H)-one (49)

Following **procedure A** on 0.1 mmol scale. Purification via silica gel column chromatography (Petroleum ether/Ethyl acetate, 20:1) afforded 32.5 mg (64%) of the title compouand.
^{1}H NMR (400 MHz, CDCl$_3$) δ 8.17 – 8.10 (m, 1H), 7.99 – 7.92 (m, 1H), 7.80 – 7.71 (m, 1H), 7.56 – 7.47 (m, 1H), 7.36 – 7.28 (m, 1H), 7.26 – 6.93 (m, 8H), 3.90 (d, *J* = 10.5 Hz, 3H), 3.10 (d, *J* = 61.5 Hz, 1H), 0.07 (s, 3H), -0.06 (s, 3H), -0.21 (s, 3H).
^{13}C NMR (101 MHz, CDCl$_3$) δ 181.36, 179.91, 164.98, 164.70, 161.31, 140.03, 139.67, 133.03, 132.48, 131.52, 131.31, 129.95, 129.49, 129.14, 127.89, 126.80, 124.01, 123.15, 119.44, 115.95, 115.73, 78.68, 77.09, 57.04, 56.98, 49.97, 48.33, 2.49, 1.10.

HRMS (ESI) calcd for $C_{26}H_{27}{}^{79}BrNO_3Si$ $(M+H)^+$: 508.0938, found: 508.0937; calcd for $C_{26}H_{27}{}^{81}BrNO_3Si$ $(M+H)^+$: 510.0918, found: 510.0921.

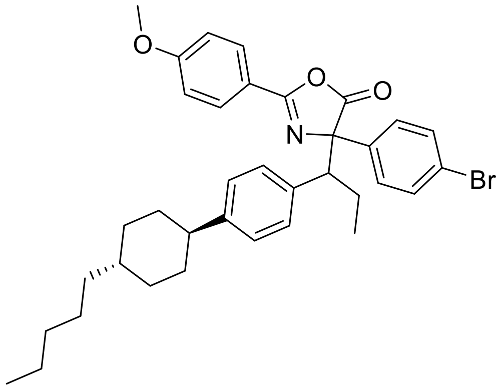

4-(4-bromophenyl)-2-(4-methoxyphenyl)-4-(1-(4-((1s,4r)-4-pentylcyclohexyl)phenyl)propyl)oxazol-5(4H)-one (50)

Following **procedure A** on 0.1 mmol scale. Purification via silica gel column chromatography (Petroleum ether/Ethyl acetate, 20:1) afforded 41.2 mg (67%) of the title compouand.

^{1}H NMR (400 MHz, CDCl$_3$) δ 7.99 – 7.91 (m, 1H), 7.82 – 7.75 (m, 1H), 7.69 – 7.57 (m, 1H), 7.52 – 7.41 (m, 1H), 7.35 – 7.22 (m, 2H), 7.04 – 6.85 (m, 6H), 3.81 (d, *J* = 4.8 Hz, 3H), 3.64 – 3.27 (m, 1H), 3.26 – 3.05 (m, 1H), 2.36 – 2.17 (m, 1H), 1.79 – 1.60 (m, 5H), 1.35 – 0.95 (m, 13H), 0.86 – 0.77 (m, 3H), 0.67 – 0.49 (m, 3H).

^{13}C NMR (101 MHz, CDCl$_3$) δ 178.17, 162.22, 162.08, 158.94, 146.00, 135.88, 133.66, 133.12, 130.56, 129.96, 128.91, 128.77, 128.48, 128.39, 127.15, 126.87, 125.38, 125.05, 121.23, 117.16, 117.08, 113.15, 113.07, 77.00, 76.83, 76.19, 56.61, 56.46, 54.50, 54.45, 43.05, 36.37, 36.35, 36.28, 36.24, 33.21, 33.16, 32.57, 31.19, 25.63, 22.49, 21.69, 20.93, 13.10, 11.28, 11.13.

HRMS (ESI) calcd for $C_{36}H_{43}{}^{79}BrNO_3$ $(M+H)^+$: 616.2421, found: 616.2424; calcd for $C_{36}H_{43}{}^{81}BrNO_3$ $(M+H)^+$: 618.2400, found: 618.2400.

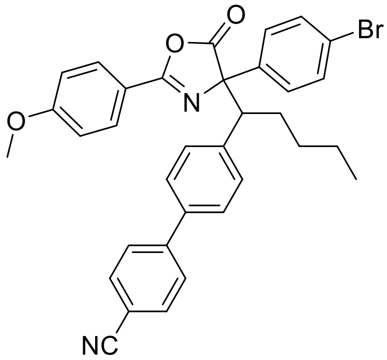

4'-(1-(4-(4-bromophenyl)-2-(4-methoxyphenyl)-5-oxo-4,5-dihydrooxazol-4-yl)pentyl)-[1,1'-biphenyl]-4-carbonitrile (51)

Following **procedure A** on 0.1 mmol scale. Purification via silica gel column chromatography (Petroleum ether/Ethyl acetate, 20:1) afforded 43.2 mg (73%) of the title compouand.

^{1}H NMR (400 MHz, CDCl$_3$) δ 8.04 – 7.95 (m, 1H), 7.88 – 7.79 (m, 1H), 7.65 – 7.47 (m, 6H), 7.36 – 7.29 (m, 3H), 7.26 – 7.16 (m, 3H), 6.99 – 6.92 (m, 1H), 6.92 – 6.83 (m, 1H), 3.81 (d, *J* = 13.9 Hz, 3H), 3.43 – 3.30 (m, 1H), 1.99 – 1.70 (m, 1H), 1.56 – 1.38 (m, 1H), 1.17 – 0.86 (m, 4H), 0.74 – 0.60 (m, 3H).

^{13}C NMR (101 MHz, CDCl$_3$) δ 177.88, 176.93, 162.44, 162.32, 159.23, 158.99, 144.15, 144.04,

137.95, 137.33, 137.06, 136.37, 135.78, 135.70, 131.48, 131.47, 130.71, 130.19, 129.39, 129.32, 129.04, 128.88, 127.09, 126.77, 126.50, 126.43, 125.79, 125.47, 121.49, 121.01, 116.87, 116.80, 113.28, 113.21, 109.74, 109.63, 76.76, 76.47, 54.57, 54.54, 54.48, 54.31, 29.26, 28.51, 28.43, 27.43, 21.31, 21.23, 12.80, 12.78.

HRMS (ESI) calcd for $C_{34}H_{30}{}^{79}BrN_2O_3$ $(M+H)^+$: 593.1434, found: 593.1439; calcd for $C_{34}H_{30}{}^{81}BrN_2O_3$ $(M+H)^+$: 595.1414, found: 595.1412.

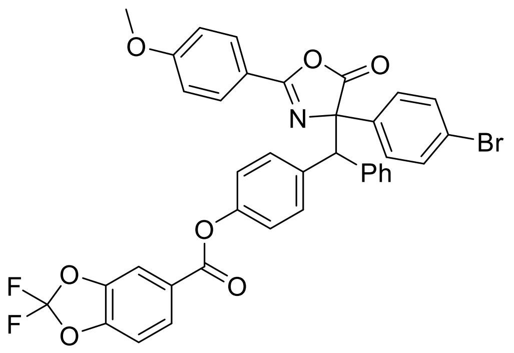

4-((4-(4-bromophenyl)-2-(4-methoxyphenyl)-5-oxo-4,5-dihydrooxazol-4-yl)(phenyl)methyl)phenyl 2,2-difluorobenzo[d][1,3]dioxole-5-carboxylate (52)

Following **procedure A** on 0.1 mmol scale. Purification via silica gel column chromatography (Petroleum ether/Ethyl acetate, 20:1) afforded 34.8 mg (49%) of the title compouand.

^{1}H NMR (400 MHz, CDCl$_3$) δ 7.96 – 7.84 (m, 3H), 7.78 – 7.71 (m, 1H), 7.66 – 7.60 (m, 1H), 7.59 – 7.48 (m, 3H), 7.36 – 7.25 (m, 4H), 7.15 – 7.03 (m, 4H), 7.00 – 6.88 (m, 4H), 4.88 (s, 1H), 3.81 (s, 3H).

^{13}C NMR (101 MHz, CDCl$_3$) δ 177.09, 162.43, 162.40, 159.60, 148.81, 148.29, 146.42, 142.74, 137.41, 136.56, 135.65, 135.23, 134.90, 130.54, 130.44, 129.95, 129.89, 129.01, 128.95, 128.72, 128.70, 127.29, 127.20, 127.09, 126.57, 126.18, 126.18, 125.91, 124.67, 121.39, 121.29, 120.13, 116.65, 113.26, 113.23, 110.19, 108.32, 108.28, 76.70, 76.69, 76.19, 59.08, 54.49.

^{19}F NMR (377 MHz, CDCl$_3$) δ -49.60, -49.61.

HRMS (ESI) calcd for $C_{37}H_{25}{}^{79}BrF_2NO_7$ $(M+H)^+$: 712.0777, found: 712.0662; calcd for $C_{37}H_{25}{}^{81}BrF_2NO_7$ $(M+H)^+$: 714.0757, found: 714.0964.

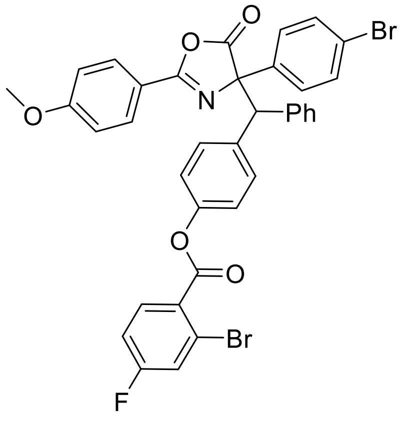

4-((4-(4-bromophenyl)-2-(4-methoxyphenyl)-5-oxo-4,5-dihydrooxazol-4-yl)(phenyl)methyl)phenyl 2-bromo-4-fluorobenzoate (53)

Following **procedure A** on 0.1 mmol scale. Purification via silica gel column chromatography (Petroleum ether/Ethyl acetate, 10:1) afforded 45.1 mg (62%) of the title compouand.

^{1}H NMR (400 MHz, CDCl$_3$) δ 7.98 – 7.84 (m, 3H), 7.65 – 7.60 (m, 1H), 7.57 – 7.48 (m, 3H), 7.39 – 7.23 (m, 5H), 7.14 – 6.87 (m, 8H), 4.88 (d, *J* = 4.6 Hz, 1H), 3.79 (s, 3H).

^{13}C NMR (101 MHz, CDCl$_3$) δ 177.19, 177.08, 163.15 (d, *J* = 258.2 Hz), 163.11 (d, *J* = 258.5 Hz), 162.42, 162.39, 162.17, 162.04, 159.62, 159.60, 148.70, 148.18, 137.43, 136.59, 135.74, 135.25, 135.22, 135.02, 132.96, 132.88, 132.79, 130.56, 130.44, 129.94, 129.87, 129.02, 128.94, 128.72, 128.68, 127.28, 127.18, 127.09, 126.55, 125.89, 122.87 (d, *J* = 7.1 Hz), 122.77 (d, *J* = 7.0 Hz), 121.40, 121.30, 121.28, 121.16 (d, *J* = 20.5 Hz), 121.01, 120.13, 116.63, 113.70 (d, *J* = 21.3 Hz), 113.65 (d, *J* = 21.3 Hz), 113.27, 113.23, 76.68, 59.01, 54.48.

^{19}F NMR (377 MHz, CDCl$_3$) δ -104.43, -104.45, -104.47, -104.49.

HRMS (ESI) calcd for $C_{36}H_{25}{}^{79}Br_2FNO_5$ $(M+H)^+$: 728.0078, found: 728.0087; calcd for $C_{36}H_{25}{}^{81}Br_2FNO_5$ $(M+H)^+$: 732.0037, found: 732.0044.

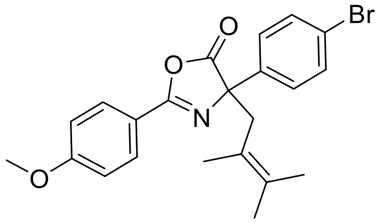

4-(4-bromophenyl)-4-(2,3-dimethylbut-2-en-1-yl)-2-(4-methoxyphenyl)oxazol-5(4H)-one (54)

Following **procedure A** on 0.1 mmol scale. Purification via silica gel column chromatography (Petroleum ether/Ethyl acetate, 30:1) afforded 17.9 mg (42%) of the title compouand.

^{1}H NMR (400 MHz, CDCl$_3$) δ 7.95 – 7.87 (m, 2H), 7.63 – 7.54 (m, 2H), 7.47 – 7.36 (m, 2H), 6.98 – 6.89 (m, 2H), 3.81 (s, 3H), 2.87 (q, *J* = 13.6 Hz, 2H), 1.56 (s, 6H), 1.49 – 1.45 (m, 3H).

^{13}C NMR (101 MHz, CDCl$_3$) δ 177.90, 162.14, 158.09, 136.93, 130.88, 130.47, 128.81, 126.63, 121.09, 120.19, 117.19, 113.19, 73.76, 54.47, 45.72, 20.20, 19.94, 19.23.

HRMS (ESI) calcd for $C_{22}H_{23}{}^{79}BrNO_3$ $(M+H)^+$: 428.0856, found: 428.0861; calcd for $C_{22}H_{23}{}^{81}BrNO_3$ $(M+H)^+$: 430.0835, found: 430.0835.

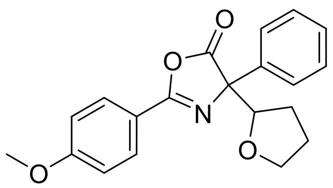

2-(4-methoxyphenyl)-4-phenyl-4-(tetrahydrofuran-2-yl)oxazol-5(4H)-one (55)

Following **procedure B** on 0.1 mmol scale. Purification via silica gel column chromatography (Petroleum ether/Ethyl acetate, 30:1) afforded 16.2 mg (48%) of the title compouand.

^{1}H NMR (400 MHz, CDCl$_3$) δ 8.18 – 7.97 (m, 2H), 7.85 – 7.63 (m, 2H), 7.50 – 7.30 (m, 3H), 7.11 – 6.88 (m, 2H), 4.79 – 4.64 (m, 1H), 3.89 (s, 3H), 3.80 – 3.71 (m, 2H), 2.18 – 2.08 (m, 1H), 2.04 – 1.94 (m, 1H), 1.84 – 1.69 (m, 2H).

^{13}C NMR (101 MHz, CDCl$_3$) δ 178.28, 163.22, 160.76, 135.84, 130.01, 128.62, 128.29, 126.08, 118.57, 114.18, 85.05, 70.25, 55.52, 26.62, 26.22.

HRMS (ESI) calcd for $C_{20}H_{20}NO_4$ $(M+H)^+$: 338.1387, found: 338.1387.

^{1}H NMR (400 MHz, CDCl$_3$) δ 8.06 – 8.00 (m, 2H), 7.82 – 7.76 (m, 2H), 7.43 – 7.29 (m, 3H),

7.09 – 6.88 (m, 2H), 4.47 (dd, 1H), 3.87 (s, 3H), 3.85 – 3.76 (m, 2H), 2.27 – 2.11 (m, 1H), 1.98 – 1.87 (m, 1H), 1.86 – 1.68 (m, 2H).

^{13}C NMR (101 MHz, CDCl$_3$) δ 176.87, 163.20, 160.34, 135.56, 130.09, 128.60, 128.32, 126.47, 118.23, 114.10, 84.33, 69.44, 55.49, 26.42, 26.00.

HRMS (ESI) calcd for $C_{20}H_{20}NO_4$ (M+H)$^+$: 338.1387, found: 338.1387.

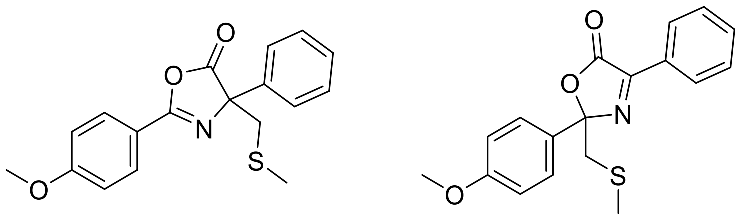

2-(4-methoxyphenyl)-4-((methylthio)methyl)-4-phenyloxazol-5(4H)-one (56)

Following **procedure B** on 0.1 mmol scale. Purification via silica gel column chromatography (Petroleum ether/Ethyl acetate, 30:1) afforded 17.0 mg (52%) of the title compouand. Regioisomers(C4/C2) = 3:1

^{1}H NMR (400 MHz, CDCl$_3$) δ 8.05 – 7.93 (m, 2H), 7.67 – 7.61 (m, 2H), 7.35 – 7.23 (m, 3H), 6.98 – 6.91 (m, 2H), 3.81 (s, 3H), 3.75 (s, 1H), 3.24 – 3.16 (m, 2H), 2.10 (s, 3H).

^{13}C NMR (101 MHz, CDCl$_3$) δ 177.03, 162.91, 162.37, 159.73, 159.11, 155.83, 136.62, 131.55, 129.09, 127.78, 127.76, 127.74, 127.53, 126.29, 124.65, 117.01, 113.26, 113.00, 104.20, 75.57, 54.50, 54.33, 45.56, 43.84, 17.61, 16.95.

HRMS (ESI) calcd for $C_{18}H_{18}NO_3S$ (M+H)$^+$: 328.1002, found: 328.1005.

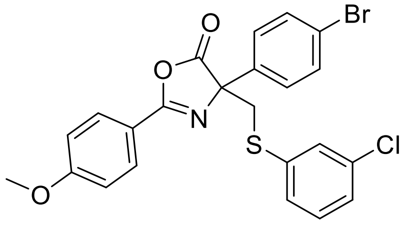

4-(4-bromophenyl)-4-(((3-chlorophenyl)thio)methyl)-2-(4-methoxyphenyl)oxazol-5(4H)-one (57)

Following **procedure B** on 0.1 mmol scale. Purification via silica gel column chromatography (Petroleum ether/Ethyl acetate, 30:1) afforded 19.0 mg (38%) of the title compouand.

^{1}H NMR (400 MHz, CDCl$_3$) δ 7.90 – 7.75 (m, 2H), 7.61 – 7.50 (m, 2H), 7.49 – 7.39 (m, 2H), 7.31 – 7.24 (m, 1H), 7.17 – 7.11 (m, 1H), 7.10 – 7.01 (m, 2H), 6.97 – 6.86 (m, 2H), 3.82 (s, 3H), 3.56 (dd, *J* = 31.5, 13.9 Hz, 2H).

^{13}C NMR (101 MHz, CDCl$_3$) δ 176.19, 162.58, 160.16, 136.15, 135.15, 133.46, 130.92, 129.49, 129.20, 128.78, 127.76, 126.53, 126.05, 122.06, 116.44, 113.23, 74.24, 54.53, 44.06.

HRMS (ESI) calcd for $C_{23}H_{18}{}^{79}BrClNO_3S$ (M+H)$^+$: 501.9874, found: 501.9873; calcd for $C_{23}H_{18}$ $^{81}BrClNO_3S$ (M+H)$^+$: 503.9853, found: 503.9853.

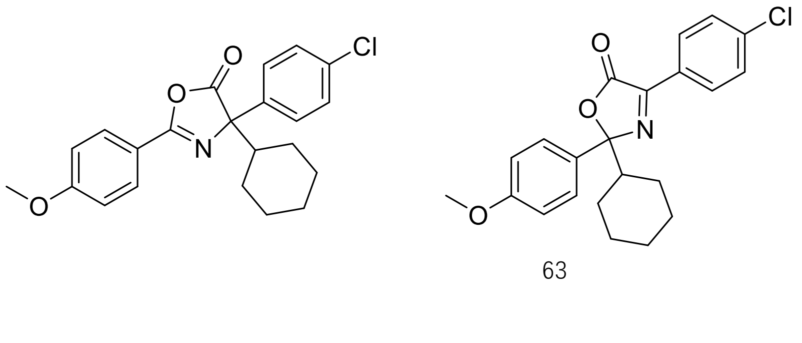

4-(4-chlorophenyl)-4-cyclohexyl-2-(4-methoxyphenyl)oxazol-5(4H)-one (58)

Following **procedure B** on 0.1 mmol scale. Purification via silica gel column chromatography (Petroleum ether/Ethyl acetate, 30:1) afforded 13.8 mg (36%) of the title compouand. regioisomers(C4/C2) = 3:1

^{1}H NMR (400 MHz, CDCl$_3$) δ 8.11 – 7.81 (m, 2H), 7.64 – 7.47 (m, 2H), 7.31 – 7.21 (m, 2H), 6.93 (d, *J* = 8.9 Hz, 2H), 3.82 (s, 3H), 2.08 – 1.99 (m, 1H), 1.70 – 1.59 (m, 3H), 1.25 – 1.13 (m, 3H), 1.08 – 0.93 (m, 4H).

^{13}C NMR (101 MHz, CDCl$_3$) δ 178.13, 162.22, 158.73, 135.45, 132.79, 128.94, 128.02, 127.49, 126.89, 126.61, 117.06, 113.17, 112.50, 76.25, 54.50, 54.27, 47.09, 46.80, 26.23, 25.87, 25.69, 25.52, 25.20, 25.01, 24.90, 24.80.

HRMS (ESI) calcd for $C_{22}H_{23}ClNO_3$ (M+H)$^+$: 384.1361, found: 384.1355.

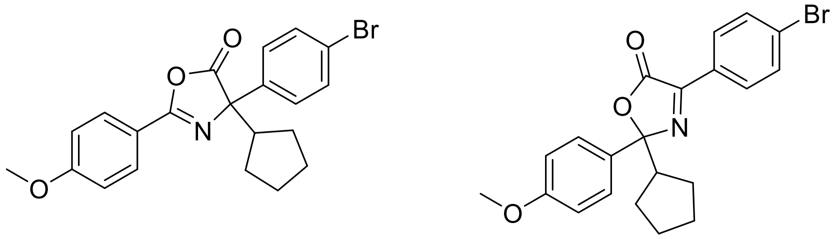

4-(4-bromophenyl)-4-cyclopentyl-2-(4-methoxyphenyl)oxazol-5(4H)-one (59)

Following **procedure B** on 0.1 mmol scale. Purification via silica gel column chromatography (Petroleum ether/Ethyl acetate, 30:1) afforded 13.2 mg (32%) of the title compouand. regioisomers(C4/C2) = 2.5:1

^{1}H NMR (400 MHz, CDCl$_3$) δ 8.02 – 7.87 (m, 2H), 7.58 – 7.51 (m, 2H), 7.44 – 7.37 (m, 2H), 6.98 – 6.87 (m, 2H), 3.82 (s, 3H), 2.80 – 2.60 (m, 1H), 1.53 – 1.34 (m, 8H).

^{13}C NMR (101 MHz, CDCl$_3$) δ 177.89, 162.27, 158.96, 158.65, 136.73, 131.01, 130.48, 129.09, 128.96, 126.68, 126.55, 121.02, 117.02, 113.20, 112.63, 107.45, 76.19, 74.51, 54.51, 54.28, 48.74, 48.42, 26.29, 26.07, 25.72, 24.64, 24.50, 24.37, 24.19.

HRMS (ESI) calcd for $C_{21}H_{21}{}^{79}BrNO_3$ (M+H)$^+$: 414.0699, found: 414.0704; calcd for $C_{21}H_{21}{}^{81}Br$ NO_3 (M+H)$^+$: 416.0679, found: 416.0683.

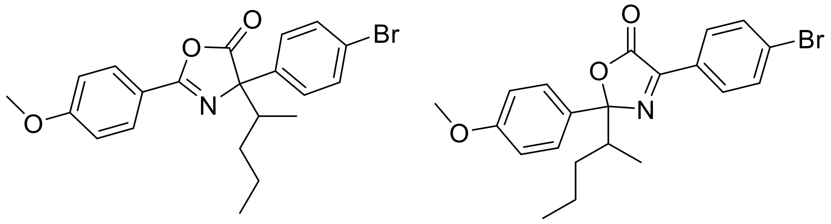

4-(4-bromophenyl)-2-(4-methoxyphenyl)-4-(pentan-2-yl)oxazol-5(4H)-one (60)

Following **procedure B** on 0.1 mmol scale. Purification via silica gel column chromatography (Petroleum ether/Ethyl acetate, 30:1) afforded 17.0 mg (41%) of the title compouand. regioisomers(C4/C2) = 5.7:1

^{1}H NMR (400 MHz, CDCl$_3$) δ 8.03 – 7.87 (m, 2H), 7.56 – 7.46 (m, 2H), 7.44 – 7.39 (m, 2H), 6.96 – 6.89 (m, 2H), 3.82 (s, 3H), 2.30 – 2.04 (m, 1H), 1.36 – 1.12 (m, 4H), 0.84 – 0.70 (m, 6H).

^{13}C NMR (101 MHz, CDCl$_3$) δ 178.60, 178.28, 178.14, 162.26, 162.23, 158.92, 158.78, 158.55, 136.66, 136.47, 136.27, 131.02, 130.48, 128.96, 128.92, 126.97, 126.93, 121.09, 113.19, 112.55, 76.98, 76.83, 76.75, 76.19, 54.51, 42.12, 41.79, 32.37, 31.30, 21.20, 20.25, 19.45, 19.32, 12.98, 12.90, 12.88, 12.37, 11.28, 10.87.

HRMS (ESI) calcd for $C_{21}H_{23}{}^{79}BrNO_3$ $(M+H)^+$: 416.0856, found: 416.0858; calcd for $C_{21}H_{23}{}^{81}Br$ NO_3 $(M+H)^+$: 418.0835, found: 418.0838.

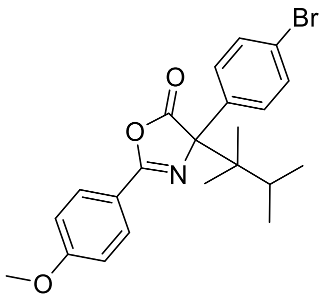

4-(4-bromophenyl)-4-(2,3-dimethylbutan-2-yl)-2-(4-methoxyphenyl)oxazol-5(4H)-one (61)
Following **procedure B** on 0.1 mmol scale. Purification via silica gel column chromatography (Petroleum ether/Ethyl acetate, 30:1) afforded 12.0 mg (28%) of the title compouand.
^{1}H NMR (400 MHz, CDCl$_3$) δ 8.01 – 7.96 (m, 2H), 7.73 – 7.66 (m, 2H), 7.48 – 7.41 (m, 2H), 6.99 – 6.95 (m, 2H), 3.88 (s, 3H), 1.97 – 1.88 (m, 1H), 1.03 (s, 3H), 0.97 (s, 3H), 0.92 (d, *J* = 6.8 Hz, 3H), 0.74 (d, *J* = 6.8 Hz, 3H).
^{13}C NMR (101 MHz, CDCl$_3$) δ 177.56, 162.09, 157.34, 134.90, 129.66, 129.06, 128.76, 121.17, 117.28, 113.16, 78.33, 76.19, 54.48, 43.99, 31.33, 20.21, 18.76, 18.65, 16.92.
HRMS (ESI) calcd for $C_{22}H_{25}{}^{79}BrNO_3$ $(M+H)^+$: 430.1012, found: 430.1017; calcd for $C_{22}H_{25}{}^{81}Br$ NO_3 $(M+H)^+$: 432.0992, found: 432.1001.

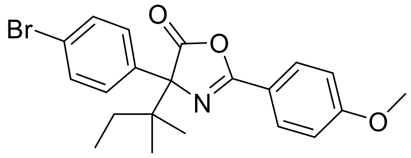

4-(4-bromophenyl)-2-(4-methoxyphenyl)-4-(tert-pentyl)oxazol-5(4H)-one (62)
Following **procedure B** on 0.1 mmol scale. Purification via silica gel column chromatography (Petroleum ether/Ethyl acetate, 30:1) afforded 19.9 mg (48%) of the title compouand.
^{1}H NMR (400 MHz, CDCl$_3$) δ 7.98 (d, *J* = 8.9 Hz, 2H), 7.70 (d, *J* = 8.7 Hz, 2H), 7.46 (d, *J* = 8.7 Hz, 2H), 6.97 (d, *J* = 8.9 Hz, 2H), 3.87 (s, 3H), 1.17 – 1.07 (m, 2H), 0.99 – 0.94 (m, 6H), 0.91 – 0.87 (m, 3H).
^{13}C NMR (101 MHz, CDCl$_3$) δ 177.03, 162.09, 157.87, 133.68, 130.54, 129.57, 129.04, 128.89, 128.81, 126.88, 121.11, 117.17, 113.12, 78.47, 54.47, 41.40, 27.17, 19.16, 19.02, 7.26.
HRMS (ESI) calcd for $C_{21}H_{23}{}^{79}BrNO_3$ $(M+H)^+$: 416.0856, found: 416.0858; calcd for $C_{21}H_{23}{}^{81}BrNO_3$ $(M+H)^+$: 418.0835, found: 418.0836.

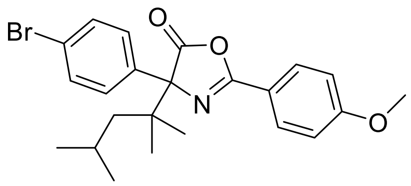

4-(4-bromophenyl)-4-(2,4-dimethylpentan-2-yl)-2-(4-methoxyphenyl)oxazol-5(4H)-one (63)
Following **procedure B** on 0.1 mmol scale. Purification via silica gel column chromatography (Petroleum ether/Ethyl acetate, 30:1) afforded 17.7mg (40%) of the title compouand.

¹H NMR (400 MHz, CDCl₃) δ 7.99 (d, *J* = 8.9 Hz, 2H), 7.69 (d, *J* = 8.7 Hz, 2H), 7.46 (d, *J* = 8.7 Hz, 2H), 6.98 (d, *J* = 8.9 Hz, 2H), 3.88 (s, 3H), 1.68 – 1.62 (m, 1H), 1.35 – 1.29 (m, 2H), 1.02 – 0.93 (m, 3H), 0.93 – 0.82 (m, 9H).

¹³C NMR (101 MHz, CDCl₃) δ 176.98, 162.10, 157.83, 133.63, 130.27, 129.56, 129.11, 128.83, 127.45, 121.12, 117.19, 113.26, 113.12, 78.61, 54.47, 43.31, 41.89, 28.67, 24.60, 24.53, 22.90, 20.22.

HRMS (ESI) calcd for $C_{23}H_{27}{}^{79}BrNO_3$ $(M+H)^+$: 444.1169, found: 444.1169; calcd for $C_{23}H_{27}{}^{81}BrNO_3$ $(M+H)^+$: 446.1148, found: 446.1147.

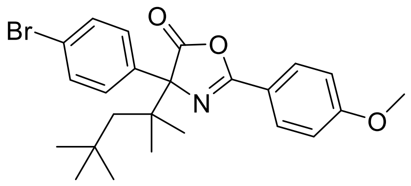

4-(4-bromophenyl)-2-(4-methoxyphenyl)-4-(2,4,4-trimethylpentan-2-yl)oxazol-5(4H)-one (64)

Following **procedure B** on 0.1 mmol scale. Purification via silica gel column chromatography (Petroleum ether/Ethyl acetate, 30:1) afforded 19.2 mg (42%) of the title compouand.

¹H NMR (400 MHz, CDCl₃) δ 8.08 – 7.95 (m, 2H), 7.71 – 7.40 (m, 4H), 7.04 – 6.96 (m, 2H), 3.88 (d, *J* = 6.4 Hz, 3H), 1.30 – 1.25 (m, 2H), 1.19 – 1.06 (m, 3H), 1.03 – 0.91 (m, 6H), 0.91 – 0.76 (m, 6H).

¹³C NMR (101 MHz, CDCl₃) δ 176.94, 162.28, 157.84, 133.50, 130.57, 130.29, 129.73, 129.49, 129.27, 128.81, 127.32, 126.51, 126.34, 121.13, 117.19, 113.30, 113.13, 79.48, 72.09, 54.50, 46.43, 42.89, 34.34, 31.07, 30.58, 29.34, 29.06, 28.89, 28.67, 28.42, 27.07, 24.50, 23.03, 21.59, 21.10.

HRMS (ESI) calcd for $C_{24}H_{29}{}^{79}BrNO_3$ $(M+H)^+$: 458.1325, found: 458.1323; calcd for $C_{24}H_{29}{}^{81}BrNO_3$ $(M+H)^+$: 460.1305, found: 460.1306.

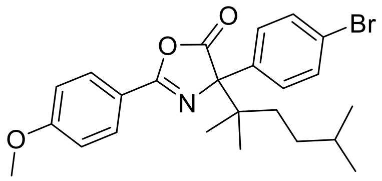

4-(4-bromophenyl)-4-(2,5-dimethylhexan-2-yl)-2-(4-methoxyphenyl)oxazol-5(4H)-one (65)

Following **procedure B** on 0.1 mmol scale. Purification via silica gel column chromatography (Petroleum ether/Ethyl acetate, 30:1) afforded 13.0 mg (28%) of the title compouand.

¹H NMR (400 MHz, CDCl₃) δ 7.99 (d, *J* = 8.9 Hz, 2H), 7.70 (d, *J* = 8.7 Hz, 2H), 7.46 (d, *J* = 8.7 Hz, 2H), 6.99 – 6.97 (m, 2H), 3.88 (s, 3H), 1.59 (s, 1H), 1.18 – 1.02 (m, 4H), 0.98 (d, *J* = 1.6 Hz, 6H), 0.83 – 0.80 (m, 6H).

¹³C NMR (101 MHz, CDCl₃) δ 177.03, 162.09, 157.87, 133.70, 129.57, 129.06, 128.82, 121.11, 117.19, 113.12, 78.53, 54.47, 41.14, 32.57, 31.90, 28.67, 27.68, 21.71, 21.66, 19.85, 19.74.

HRMS (ESI) calcd for $C_{24}H_{29}{}^{79}BrNO_3$ $(M+H)^+$: 458.1325, found: 458.1328; calcd for $C_{24}H_{29}{}^{81}BrNO_3$ $(M+H)^+$: 460.1305, found: 460.1307.

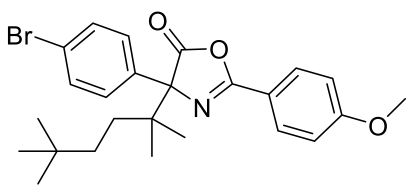

4-(4-bromophenyl)-2-(4-methoxyphenyl)-4-(2,5,5-trimethylhexan-2-yl)oxazol-5(4H)-one (66)

Following **procedure B** on 0.1 mmol scale. Purification via silica gel column chromatography (Petroleum ether/Ethyl acetate, 30:1) afforded 20.2 mg (43%) of the title compouand.

^{1}H NMR (400 MHz, CDCl$_3$) δ 8.05 – 7.96 (m, 2H), 7.74 – 7.54 (m, 2H), 7.53 – 7.41 (m, 2H), 7.03 – 6.95 (m, 2H), 3.87 (s, 3H), 1.36 – 1.23 (m, 4H), 1.00 – 0.86 (m, 6H), 0.86 – 0.76 (m, 9H).

^{13}C NMR (101 MHz, CDCl$_3$) δ 177.05, 162.08, 157.86, 133.74, 129.56, 129.05, 128.88, 128.80, 121.12, 117.20, 113.13, 78.59, 54.46, 41.02, 36.42, 29.27, 29.17, 28.67, 28.28, 21.68, 19.85, 19.67.

HRMS (ESI) calcd for $C_{25}H_{31}{}^{79}BrNO_3$ $(M+H)^+$: 472.1482, found: 472.1481. calcd for $C_{25}H_{31}{}^{81}BrNO_3$ $(M+H)^+$: 474.1461, found: 474.1461.

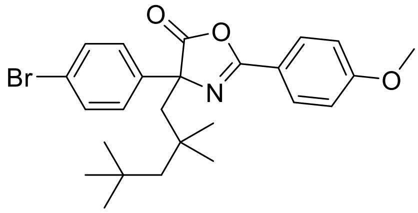

4-(4-bromophenyl)-2-(4-methoxyphenyl)-4-(2,2,4,4-tetramethylpentyl)oxazol-5(4H)-one (67)

Following **procedure B** on 0.1 mmol scale. Purification via silica gel column chromatography (Petroleum ether/Ethyl acetate, 30:1) afforded 18.0 mg (39%) of the title compouand.

^{1}H NMR (400 MHz, CDCl$_3$) δ 8.03 (d, J = 8.7 Hz, 2H), 7.55 (d, J = 8.5 Hz, 2H), 7.46 (d, J = 8.5 Hz, 2H), 7.02 (d, J = 8.7 Hz, 2H), 3.89 (s, 3H), 2.30 (d, J = 3.4 Hz, 2H), 1.32 – 1.26 (m, 2H), 1.05 – 0.91 (m, 15H).

^{13}C NMR (101 MHz, CDCl$_3$) δ 178.84, 162.33, 158.02, 139.02, 130.59, 130.09, 128.86, 126.37, 121.08, 117.29, 113.34, 72.17, 54.71, 54.50, 53.25, 35.83, 32.98, 31.68, 31.35, 31.12, 28.31, 28.25.

HRMS (ESI) calcd for $C_{25}H_{31}{}^{79}BrNO_3$ $(M+H)^+$: 472.1482, found: 472.1485; calcd for $C_{25}H_{31}{}^{81}BrNO_3$ $(M+H)^+$: 474.1461, found: 474.1463.

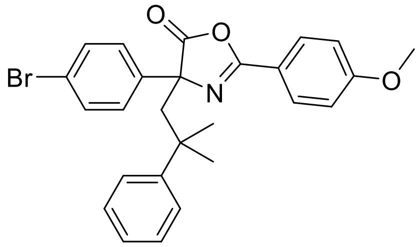

4-(4-bromophenyl)-2-(4-methoxyphenyl)-4-(2-methyl-2-phenylpropyl)oxazol-5(4H)-one (68)

Following **procedure B** on 0.1 mmol scale. Purification via silica gel column chromatography

(Petroleum ether/Ethyl acetate, 30:1) afforded 10.0 mg (21%) of the title compouand.

^{1}H NMR (600 MHz, $CDCl_3$) δ 7.78 – 7.74 (m, 2H), 7.47 – 7.44 (m, 2H), 7.42 – 7.40 (m, 2H), 7.21 – 7.18 (m, 2H), 7.15 – 7.12 (m, 3H), 6.97 – 6.95 (m, 2H), 3.90 (s, 3H), 2.73 (d, *J* = 14.7 Hz, 1H), 2.55 (d, *J* = 14.6 Hz, 1H), 1.43 (d, *J* = 4.1 Hz, 3H), 1.36 (d, 3H).

^{13}C NMR (151 MHz, $CDCl_3$) δ 178.11, 162.12, 158.10, 145.15, 138.70, 130.50, 129.02, 128.89, 126.86, 126.22, 126.05, 125.43, 124.94, 121.05, 117.11, 113.03, 71.74, 54.47, 53.14, 37.06, 29.70, 28.71.

HRMS (ESI) calcd for $C_{26}H_{25}{}^{79}BrNO_3$ $(M+H)^+$: 478.1012, found: 478.1014; calcd for $C_{26}H_{25}{}^{81}BrNO_3$ $(M+H)^+$: 480.0992, found: 480.0994.

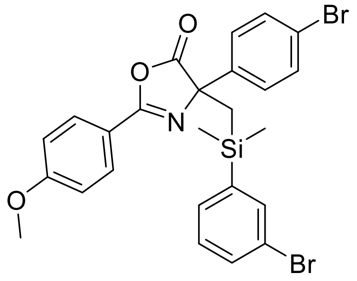

4-(4-bromophenyl)-4-(((3-bromophenyl)dimethylsilyl)methyl)-2-(4-methoxyphenyl)oxazol-5(4H)-one (69)

Following **procedure B** on 0.1 mmol scale. Purification via silica gel column chromatography (Petroleum ether/Ethyl acetate, 30:1) afforded 11.9 mg (21%) of the title compouand.

^{1}H NMR (600 MHz, $CDCl_3$) δ 8.14 – 7.84 (m, 2H), 7.77 – 7.36 (m, 6H), 7.25 (d, *J* = 9.0 Hz, 1H), 7.15 – 6.92 (m, 3H), 3.90 (s, 3H), 1.80 (dd, *J* = 162.5, 14.7 Hz, 2H), 0.27 – 0.20 (m, 4H), 0.07 (s, 2H).

^{13}C NMR (151 MHz, $CDCl_3$) δ 178.59, 162.33, 158.52, 140.03, 137.79, 135.27, 131.05, 130.88, 130.86, 130.46, 128.91, 128.41, 126.41, 121.74, 121.28, 116.89, 113.27, 70.55, 54.52, 30.58, 28.61, 28.46, -2.96, -3.12.

HRMS (ESI) calcd for $C_{25}H_{24}{}^{79}Br_2NO_3Si$ $(M+H)^+$: 571.9887, found: 571.9891; calcd for $C_{25}H_{24}{}^{81}Br_2NO_3Si$ $(M+H)^+$: 573.9866, found: 573.9863.

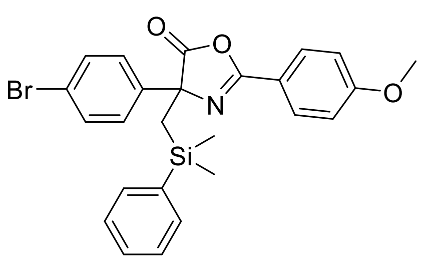

4-(4-bromophenyl)-4-((dimethyl(phenyl)silyl)methyl)-2-(4-methoxyphenyl)oxazol-5(4H)-one (70)

Following **procedure B** on 0.1 mmol scale. Purification via silica gel column chromatography (Petroleum ether/Ethyl acetate, 30:1) afforded 13.0 mg (26%) of the title compouand.

^{1}H NMR (400 MHz, $CDCl_3$) δ 7.73 (d, *J* = 8.9 Hz, 2H), 7.30 – 7.20 (m, 4H), 7.17 – 7.03 (m, 5H), 6.81 (d, *J* = 8.9 Hz, 2H), 3.72 (s, 3H), 1.78 – 1.73 (m, 1H), 1.52 (d, *J* = 14.7 Hz, 1H), 0.11 (s, 3H), -0.11 (s, 3H).

^{13}C NMR (101 MHz, $CDCl_3$) δ 181.63, 165.22, 161.41, 141.16, 139.73, 135.47, 133.39, 131.87, 130.92, 129.60, 129.39, 124.09, 120.04, 116.14, 73.71, 57.45, 33.54, 31.62, 31.57, 2.95.

HRMS (ESI) calcd for $C_{25}H_{25}{}^{79}BrNO_3Si$ $(M+H)^+$: 494.0782, found: 494.0782; calcd for $C_{25}H_{25}{}^{81}BrNO_3Si$ $(M+H)^+$: 496.0761, found: 496.0765.

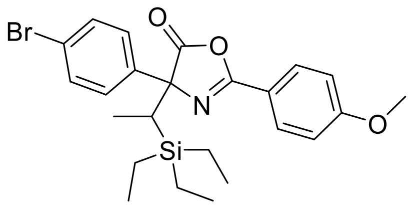

4-(4-bromophenyl)-2-(4-methoxyphenyl)-4-((triethylsilyl)methyl)oxazol-5(4H)-one (71)
Following **procedure B** on 0.1 mmol scale. Purification via silica gel column chromatography (Petroleum ether/Ethyl acetate, 30:1) afforded 16.0 mg (33%) of the title compouand.
^{1}H NMR (400 MHz, CDCl$_3$) δ 8.05 – 8.01 (m, 2H), 7.63 – 7.39 (m, 4H), 7.03– 7.00 (m, 2H), 3.89 (s, 3H), 2.05 – 1.77 (m, 1H), 1.02 – 0.79 (m, 12H), 0.57 – 0.26 (m, 6H).
^{13}C NMR (101 MHz, CDCl$_3$) δ 178.92, 178.66, 162.24, 158.39, 137.62, 137.26, 130.63, 130.41, 130.36, 128.98, 128.91, 127.15, 127.02, 126.50, 121.32, 120.97, 117.20, 117.10, 113.29, 113.26, 74.52, 54.51, 54.48, 35.63, 30.59, 28.61, 28.17, 27.73, 10.99, 10.44, 6.61, 6.50, 6.28, 4.27, 2.22, 1.96, 1.90.
HRMS (ESI) calcd for $C_{24}H_{31}{}^{79}BrNO_3Si$ $(M+H)^+$: 488.1250, found: 488.1255; calcd for $C_{24}H_{31}{}^{81}BrNO_3Si$ $(M+H)^+$: 490.1231, found: 490.1232.

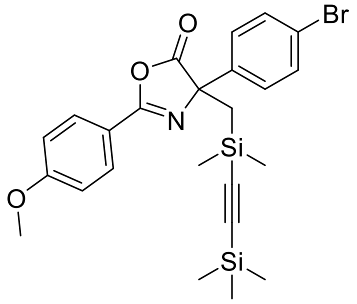

4-(4-bromophenyl)-4-((dimethyl((trimethylsilyl)ethynyl)silyl)methyl)-2-(4-methoxyphenyl)oxazol-5(4H)-one (72)
Following **procedure B** on 0.1 mmol scale. Purification via silica gel column chromatography (Petroleum ether/Ethyl acetate, 30:1) afforded 10.2 mg (20%) of the title compouand.
^{1}H NMR (600 MHz, CDCl$_3$) δ 8.05 (d, J = 7.8 Hz, 2H), 7.40 (dd, J = 34.3, 7.7 Hz, 4H), 7.00 (d, J = 7.7 Hz, 2H), 3.89 (s, 3H), 1.41 – 1.25 (m, 2H), 0.37 (s, 7H), 0.07 – 0.06 (m, 8H).
^{13}C NMR (151 MHz, CDCl$_3$) δ 176.19, 161.54, 159.45, 141.33, 137.11, 129.55, 128.30, 127.68, 121.10, 116.92, 112.52, 72.99, 65.91, 65.67, 53.87, 1.64, -0.01, -0.60.
HRMS (ESI) calcd for $C_{24}H_{29}{}^{79}BrNO_3Si_2$ $(M+H)^+$: 514.0864, found: 514.0867; calcd for $C_{24}H_{29}{}^{81}BrNO_3Si_2$ $(M+H)^+$: 516.0843, found: 516.0842.

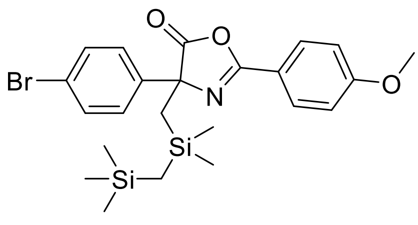

(4-bromophenyl)-4-((dimethyl((trimethylsilyl)methyl)silyl)methyl)-2-(4-methoxyphenyl)oxazol-5(4H)-one (73)

Following **procedure B** on 0.1 mmol scale. Purification via silica gel column chromatography (Petroleum ether/Ethyl acetate, 30:1) afforded 20.6 mg (40%) of the title compouand.

^{1}H NMR (400 MHz, CDCl$_3$) δ 8.00 (d, *J* = 8.9 Hz, 2H), 7.54 – 7.39 (m, 4H), 6.99 (d, *J* = 8.9 Hz, 2H), 3.87 (d, *J* = 1.1 Hz, 3H), 1.45 –1.26 (m, 2H), 0.04 (d, *J* = 7.6 Hz, 6H), -0.06 (d, *J* = 5.5 Hz, 6H), -0.12 (d, *J* = 9.4 Hz, 5H).

^{13}C NMR (101 MHz, CDCl$_3$) δ 179.28, 178.66, 161.98, 161.96, 158.01, 157.91, 139.30, 138.24, 130.18, 130.06, 128.53, 128.51, 127.11, 126.16, 120.96, 120.74, 116.93, 116.82, 113.03, 112.97, 75.35, 73.34, 70.73, 54.17, 30.26, 30.20, 28.94, 28.35, 24.78, 2.27, 1.06, 0.28, -0.01, -0.33, -0.79, -0.81.

HRMS (ESI) calcd for $C_{23}H_{31}{}^{79}BrNO_3Si_2$ $(M+H)^+$: 504.1020, found: 504.1022; calcd for $C_{23}H_{31}{}^{81}BrNO_3Si_2$ $(M+H)^+$: 506.1000, found: 506.1003.

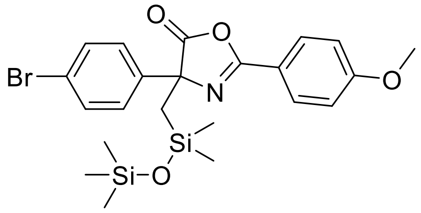

4-(4-bromophenyl)-2-(4-methoxyphenyl)-4-(2,2,4,4-tetramethylpentyl)oxazol-5(4H)-one (74)

Following **procedure B** on 0.1 mmol scale. Purification via silica gel column chromatography (Petroleum ether/Ethyl acetate, 30:1) afforded 18.0 mg (37%) of the title compouand.

^{1}H NMR (400 MHz, CDCl$_3$) δ 8.02 (d, *J* = 8.7 Hz, 2H), 7.49 (dd, *J* = 22.3, 8.6 Hz, 4H), 7.01 (d, *J* = 8.7 Hz, 2H), 3.89 (s, 3H), 1.63 (s, 2H), 0.09 (d, *J* = 5.7 Hz, 5H), -0.01 (d, *J* = 7.4 Hz, 10H).

^{13}C NMR (101 MHz, CDCl$_3$) δ 177.75, 161.56, 157.93, 138.34, 129.83, 128.20, 125.63, 120.30, 116.62, 112.55, 69.69, 53.76, 29.61, 0.32, 0.13, -0.01, -0.72.

HRMS (ESI) calcd for $C_{22}H_{29}{}^{79}BrNO_4Si_2$ $(M+H)^+$: 506.0813, found: 506.0820. calcd for $C_{22}H_{29}{}^{81}BrNO_4Si_2$ $(M+H)^+$: 508.0793, found: 508.0797.

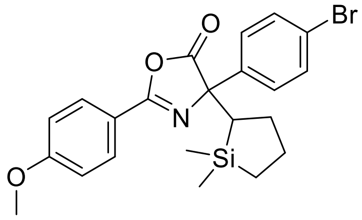

4-(4-bromophenyl)-4-(1,1-dimethylsilolan-2-yl)-2-(4-methoxyphenyl)oxazol-5(4H)-one (75)

Following **procedure B** on 0.1 mmol scale. Purification via silica gel column chromatography (Petroleum ether/Ethyl acetate, 30:1) afforded 18.7mg (41%) of the title compouand.

^{1}H NMR (600 MHz, CDCl$_3$) δ 8.04 – 7.97 (m, 2H), 7.64 – 7.54 (m, 2H), 7.53 – 7.45 (m, 2H), 7.01 –6.99 (m, 2H), 3.89 – 3.88 (m, 3H), 1.89 – 1.67 (m, 2H), 1.43 – 1.24 (m, 4H), 0.28 – 0.01 (m, 7H).

^{13}C NMR (151 MHz, CDCl$_3$) δ 178.02, 177.87, 162.23, 162.20, 162.16, 158.97, 158.74, 158.44, 137.38, 136.82, 136.68, 131.00, 130.97, 130.61, 130.54, 130.40, 130.36, 130.32, 129.08,

129.06, 128.96, 128.93, 128.85, 128.84, 126.75, 126.71, 126.67, 120.88, 120.86, 120.83, 117.34, 117.16, 117.09, 117.07, 113.23, 113.18, 113.16, 112.53, 112.49, 73.82, 73.47, 54.49, 54.47, 54.26, 50.29, 49.98, 39.43, 38.76, 30.58, 30.39, 29.26, 29.14, 28.93, 28.67, 28.60, 28.57, 26.49, 26.30, 23.10, 22.82, 13.07, 12.65, 12.52, 12.35, 11.22, 10.92, -2.07, -2.27, -2.30, -2.43, -2.53, -2.62, -2.63, -3.73.

HRMS (ESI) calcd for $C_{22}H_{25}{}^{79}BrNO_3Si$ $(M+H)^+$: 458.0782, found: 458.0785; calcd for $C_{22}H_{25}{}^{81}BrNO_3Si$ $(M+H)^+$: 460.0761, found: 460.0763.

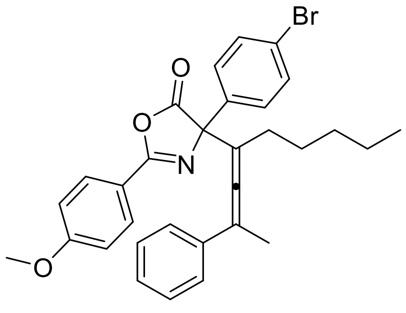

4-(4-bromophenyl)-2-(4-methoxyphenyl)-4-(2-phenylnona-2,3-dien-4-yl)oxazol-5(4H)-one (76)

Following **procedure A** on 0.1 mmol scale. Purification via silica gel column chromatography (Petroleum ether/Ethyl acetate, 20:1) afforded 33.1 mg (61%) of the title compouand.

^{1}H NMR (400 MHz, CDCl$_3$) δ 8.03 – 7.92 (m, 2H), 7.55 – 7.47 (m, 2H), 7.42 – 7.35 (m, 2H), 7.31 – 7.21 (m, 3H), 7.18 – 7.10 (m, 2H), 6.97 – 6.90 (m, 2H), 3.82 (s, 3H), 2.01 (s, 1H), 1.97 (s, 1H), 1.33 – 1.07 (m, 8H), 0.74 – 0.66 (m, 3H).

^{13}C NMR (101 MHz, CDCl$_3$) δ 200.98, 175.14, 162.37, 159.75, 135.67, 135.45, 135.38, 135.32, 130.38, 130.34, 129.04, 127.44, 127.30, 127.26, 126.06, 125.95, 124.78, 124.64, 121.42, 117.13, 113.22, 107.41, 107.22, 105.95, 105.90, 54.51, 30.38, 27.42, 27.35, 26.28, 21.44, 16.07, 15.97, 12.96.

HRMS (ESI) calcd for $C_{31}H_{31}{}^{79}BrNO_3$ $(M+H)^+$: 544.1482, found: 544.1484; calcd for $C_{31}H_{31}{}^{81}Br$ NO_3 $(M+H)^+$: 546.1461, found: 546.1470.

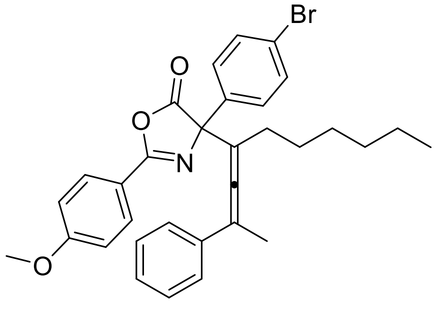

4-(4-bromophenyl)-2-(4-methoxyphenyl)-4-(2-phenyldeca-2,3-dien-4-yl)oxazol-5(4H)-one (77)

Following **procedure A** on 0.1 mmol scale. Purification via silica gel column chromatography (Petroleum ether/Ethyl acetate, 20:1) afforded 35.1 mg (63%) of the title compouand.

^{1}H NMR (400 MHz, CDCl$_3$) δ 8.01 – 7.92 (m, 2H), 7.55 – 7.47 (m, 2H), 7.42 – 7.36 (m, 2H), 7.30 – 7.22 (m, 3H), 7.17 – 7.07 (m, 2H), 6.97 – 6.90 (m, 2H), 3.82 (s, 3H), 1.99 (d, J = 16.4 Hz, 3H), 1.29 – 1.04 (m, 10H), 0.76 – 0.68 (m, 3H).

^{13}C NMR (101 MHz, CD$_3$CN) δ 200.98, 175.15, 162.38, 159.56, 135.33, 130.39, 130.35, 129.05,

127.45, 127.30, 127.27, 126.07, 125.96, 124.79, 124.65, 121.43, 117.14, 117.11, 113.23, 107.40, 107.22, 105.94, 105.90, 76.20, 54.51, 30.64, 27.88, 27.48, 27.40, 26.60, 21.55, 16.08, 15.98, 12.98.

HRMS (ESI) calcd for $C_{32}H_{33}{}^{79}BrNO_3$ $(M+H)^+$: 558.1638, found: 558.1640; calcd for $C_{32}H_{33}{}^{81}Br$ NO_3 $(M+H)^+$: 560.0618, found: 560.0619.

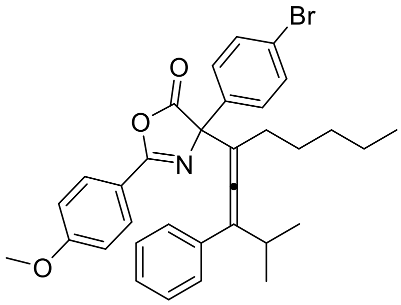

4-(4-bromophenyl)-2-(4-methoxyphenyl)-4-(2-methyl-3-phenyldeca-3,4-dien-5-yl)oxazol-5(4H)-one (78)

Following **procedure A** on 0.1 mmol scale. Purification via silica gel column chromatography (Petroleum ether/Ethyl acetate, 20:1) afforded 36.0 mg (63%) of the title compouand.

^{1}H NMR (600 MHz, $CDCl_3$) δ 8.00 – 7.89 (m, 2H), 7.57 – 7.45 (m, 2H), 7.43 – 7.37 (m, 2H), 7.35 – 7.22 (m, 3H), 7.18 – 7.07 (m, 2H), 6.98 – 6.87 (m, 2H), 3.82 (s, 3H), 2.81 – 2.61 (m, 1H), 2.09 – 1.79 (m, 2H), 1.38 – 1.21 (m, 2H), 1.14 – 0.95 (m, 7H), 0.92 – 0.86 (m, 3H), 0.74 – 0.61 (m, 3H).

^{13}C NMR (151 MHz, $CDCl_3$) δ 199.59, 198.97, 175.26, 174.82, 162.31, 159.51, 135.59, 135.24, 135.18, 135.13, 130.50, 130.41, 129.02, 128.97, 127.69, 127.62, 127.34, 126.01, 125.85, 125.62, 125.48, 121.45, 121.41, 118.16, 117.98, 117.15, 117.09, 113.22, 113.20, 109.69, 109.32, 75.88, 54.50, 30.51, 30.47, 28.08, 27.68, 27.43, 27.39, 26.50, 26.37, 21.45, 21.41, 21.10, 21.06, 21.03, 20.84, 12.94, 12.92.

HRMS (ESI) calcd for $C_{33}H_{35}{}^{79}BrNO_3$ $(M+H)^+$: 572.1795, found: 572.1797; calcd for $C_{33}H_{35}{}^{81}Br$ NO_3 $(M+H)^+$: 574.1774, found: 574.1770.

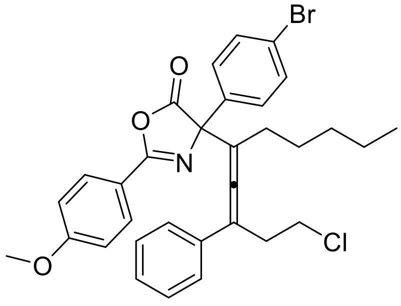

4-(4-bromophenyl)-4-(1-chloro-3-phenyldeca-3,4-dien-5-yl)-2-(4-methoxyphenyl)oxazol-5(4H)-one (79)

Following **procedure A** on 0.1 mmol scale. Purification via silica gel column chromatography (Petroleum ether/Ethyl acetate, 20:1) afforded 31.9 mg (54%) of the title compouand.

^{1}H NMR (600 MHz, $CDCl_3$) δ 8.05 – 8.00 (m, 2H), 7.58 – 7.53 (m, 2H), 7.49 – 7.44 (m, 2H), 7.39 – 7.33 (m, 2H), 7.29 – 7.26 (m, 1H), 7.26 – 7.23 (m, 1H), 7.21 – 7.18 (m, 1H), 7.03 – 6.99 (m, 2H), 3.90 (d, *J* = 2.2 Hz, 3H), 3.65 – 3.50 (m, 2H), 2.95 – 2.78 (m, 2H), 2.11 – 1.95 (m, 2H), 1.43 – 1.32 (m, 2H), 1.23 – 1.13 (m, 4H), 0.80 – 0.74 (m, 3H).

^{13}C NMR (151 MHz, CDCl$_3$) δ 200.77, 200.24, 175.18, 174.80, 162.45, 159.90, 159.77, 135.13, 134.86, 134.42, 133.97, 130.72, 130.54, 129.08, 129.06, 127.59, 127.57, 127.42, 127.40, 127.20, 127.01, 126.54, 126.42, 126.20, 124.93, 124.69, 121.66, 121.61, 116.91, 116.87, 113.27, 110.17, 109.70, 108.01, 107.84, 75.39, 54.52, 41.13, 32.92, 32.68, 30.46, 30.45, 28.68, 27.50, 27.48, 26.40, 26.32, 21.41, 21.37, 12.94.
HRMS (ESI) calcd for $C_{32}H_{32}{}^{79}BrClNO_3$ $(M+H)^+$: 592.1249, found: 592.1249; calcd for $C_{32}H_{32}{}^{81}BrClNO_3$ $(M+H)^+$: 594.1228, found: 594.1230.

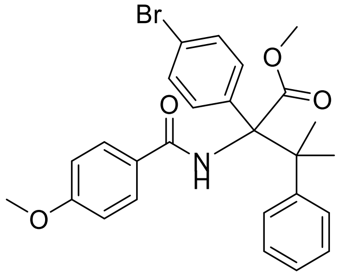

methyl 2-(4-bromophenyl)-2-(4-methoxybenzamido)-3-methyl-3-phenylbutanoate (82)
Following **procedure A** on 0.1 mmol scale. Purification via silica gel column chromatography (Petroleum ether/Ethyl acetate, 3:1) afforded 24.3 mg (49%) of the title compouand.
^{1}H NMR (400 MHz, CDCl$_3$) δ 7.58 – 7.49 (m, 3H), 7.35 – 7.23 (m, 5H), 7.16 – 7.09 (m, 2H), 6.88 – 6.81 (m, 2H), 6.68 (s, 1H), 3.78 (s, 3H), 3.51 (s, 3H), 1.49 (s, 3H), 1.35 (s, 3H).
^{13}C NMR (101 MHz, CDCl$_3$) δ 169.75, 165.37, 161.43, 141.98, 134.57, 129.67, 128.95, 127.68, 127.26, 126.70, 126.50, 125.55, 120.61, 112.87, 68.31, 54.44, 51.09, 44.40, 24.39, 23.94.
HRMS (ESI) calcd for $C_{26}H_{27}{}^{79}BrNO_4$ $(M+H)^+$: 496.1118, found: 496.1120; calcd for $C_{26}H_{27}{}^{81}BrNO_4$ $(M+H)^+$: 498.1098, found: 498.1101.

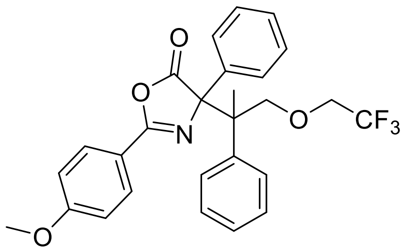

2-(4-methoxyphenyl)-4-phenyl-4-(2-phenyl-1-(2,2,2-trifluoroethoxy)propan-2-yl)oxazol-5 (4H)-one (91)
^{1}H NMR (400 MHz, CDCl$_3$) δ 7.97 – 7.85 (m, 1H), 7.83 – 7.73 (m, 1H), 7.68 – 7.58 (m, 1H), 7.46 – 7.35 (m, 1H), 7.31 – 7.19 (m, 4H), 7.15 – 7.02 (m, 5H), 6.95 – 6.80 (m, 2H), 4.42 (d, *J* = 9.1 Hz, 1H), 4.08 (d, *J* = 8.9 Hz, 1H), 3.86 (d, *J* = 8.9 Hz, 1H), 3.79 (d, *J* = 7.2 Hz, 3H), 3.76 – 3.57 (m, 2H), 1.64 (d, *J* = 44.4 Hz, 3H).
^{13}C NMR (101 MHz, CDCl$_3$) δ 177.18, 176.56, 163.11, 163.07, 159.49, 138.98, 135.37, 129.76, 129.71, 128.70, 128.38, 128.32, 128.26, 127.89, 127.77, 127.70, 127.42, 127.33, 127.32, 127.25, 118.39, 118.15, 114.17, 114.10, 77.24, 76.89, 76.45, 68.86 (dd, *J* = 34.1, 8.2 Hz), 55.48, 51.10, 50.22, 18.92, 17.79.
^{19}F NMR (377 MHz, CDCl$_3$) δ -73.59, -73.64.
HRMS (ESI) calcd for $C_{27}H_{25}F_3NO_4$ $(M+H)^+$: 484.1730, found: 484.1737.

NMR Spectra

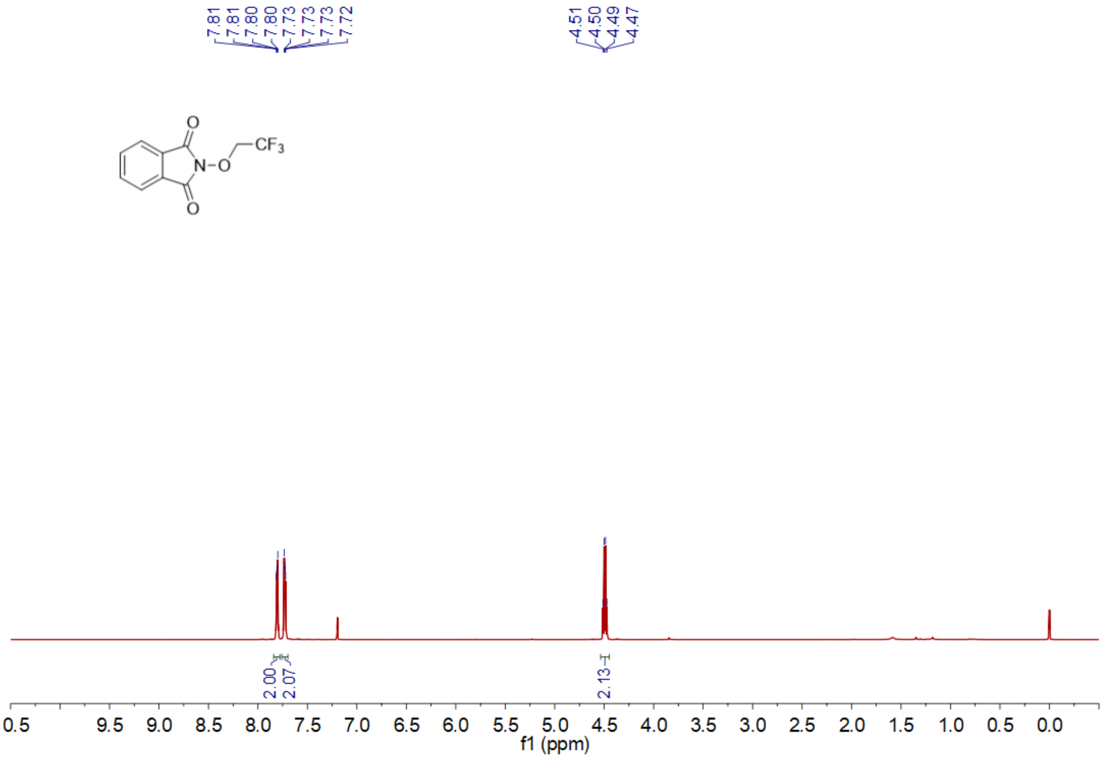

Supplementary Figure 35 ^{1}H NMR (600 MHz, $CDCl_3$, 25 °C) of compound **H1**

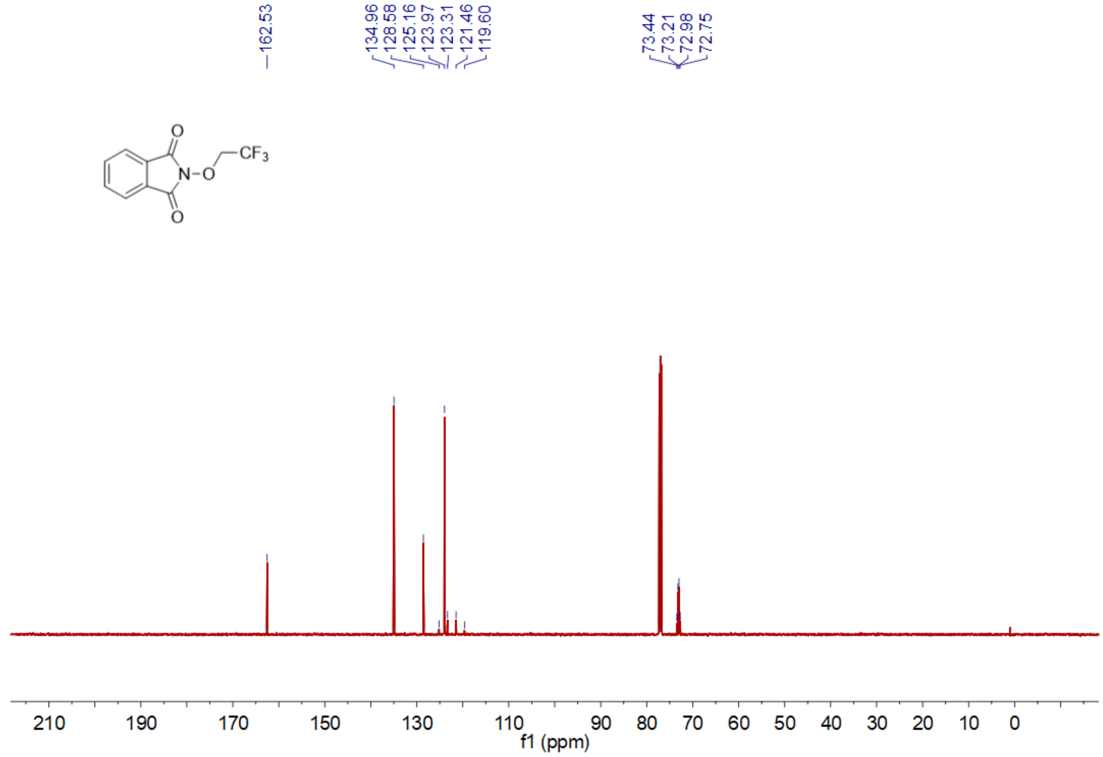

Supplementary Figure 36 ^{13}C NMR (151 MHz, $CDCl_3$, 25 °C) of compound **H1**

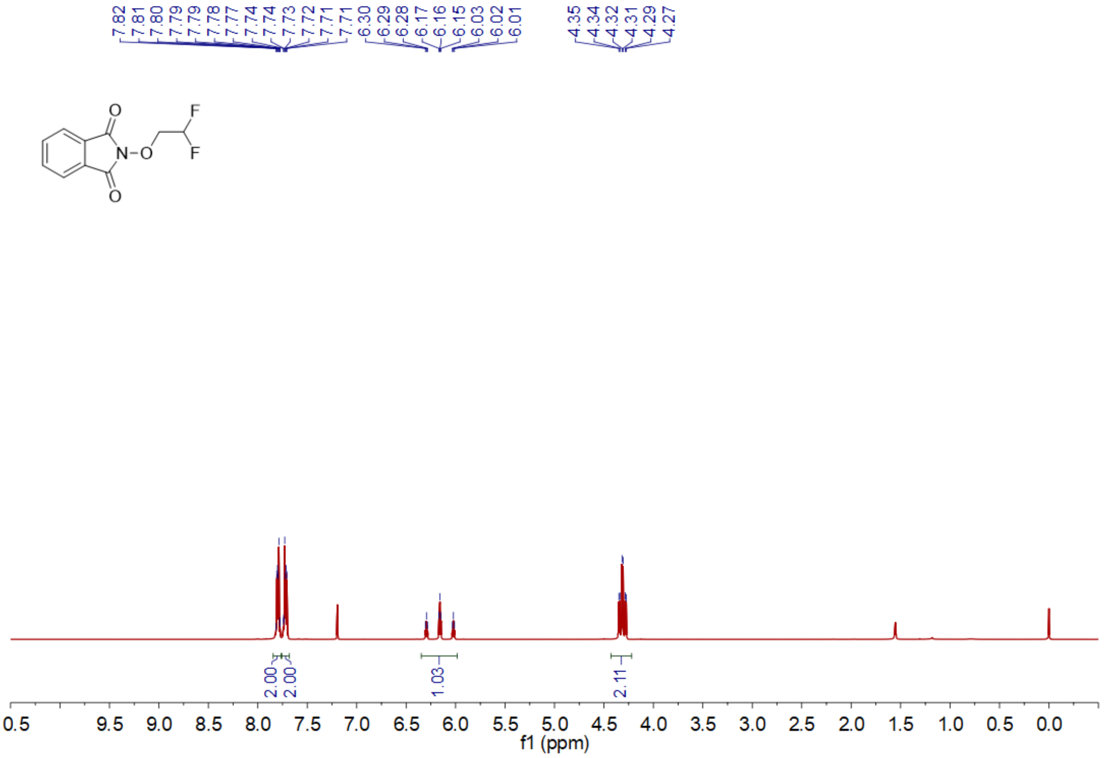

Supplementary Figure 37 ^{1}H NMR (400 MHz, $CDCl_3$, 25 °C) of compound **H2**

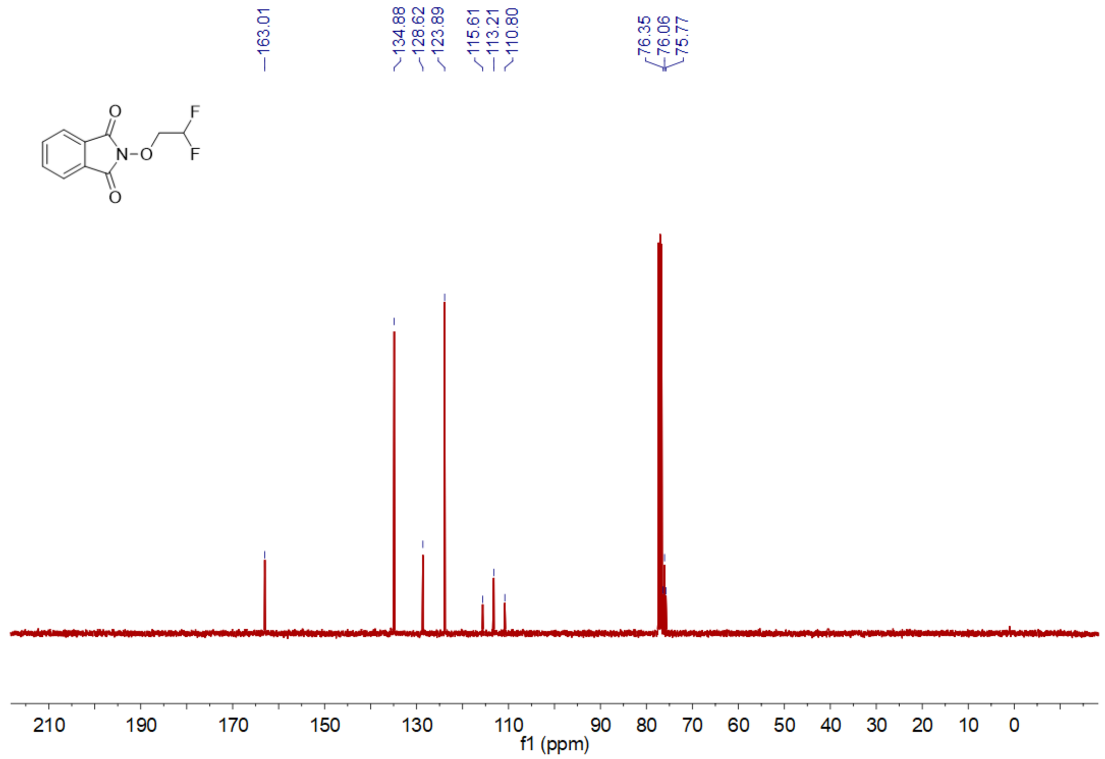

Supplementary Figure 38 ^{13}C NMR (151 MHz, $CDCl_3$, 25 °C) of compound **H2**

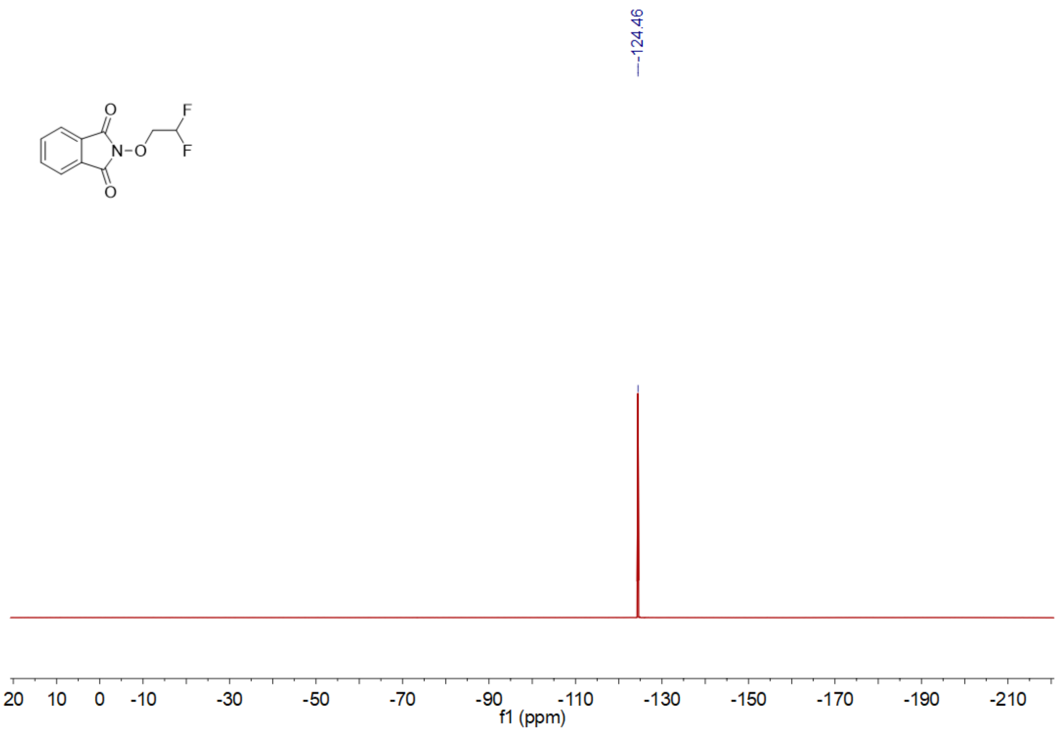

Supplementary Figure 39 ^{19}F NMR (377 MHz, $CDCl_3$, 25 °C) of compound **H2**

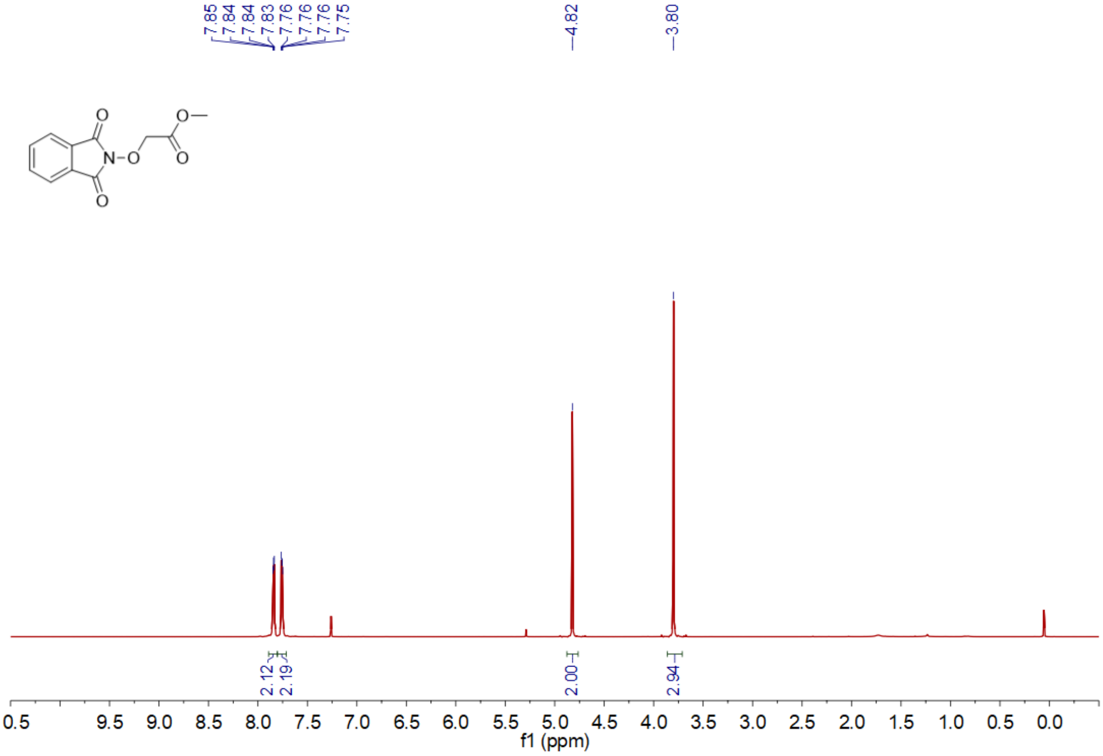

Supplementary Figure 40 ^{1}H NMR (600 MHz, $CDCl_3$, 25 °C) of compound **H3**

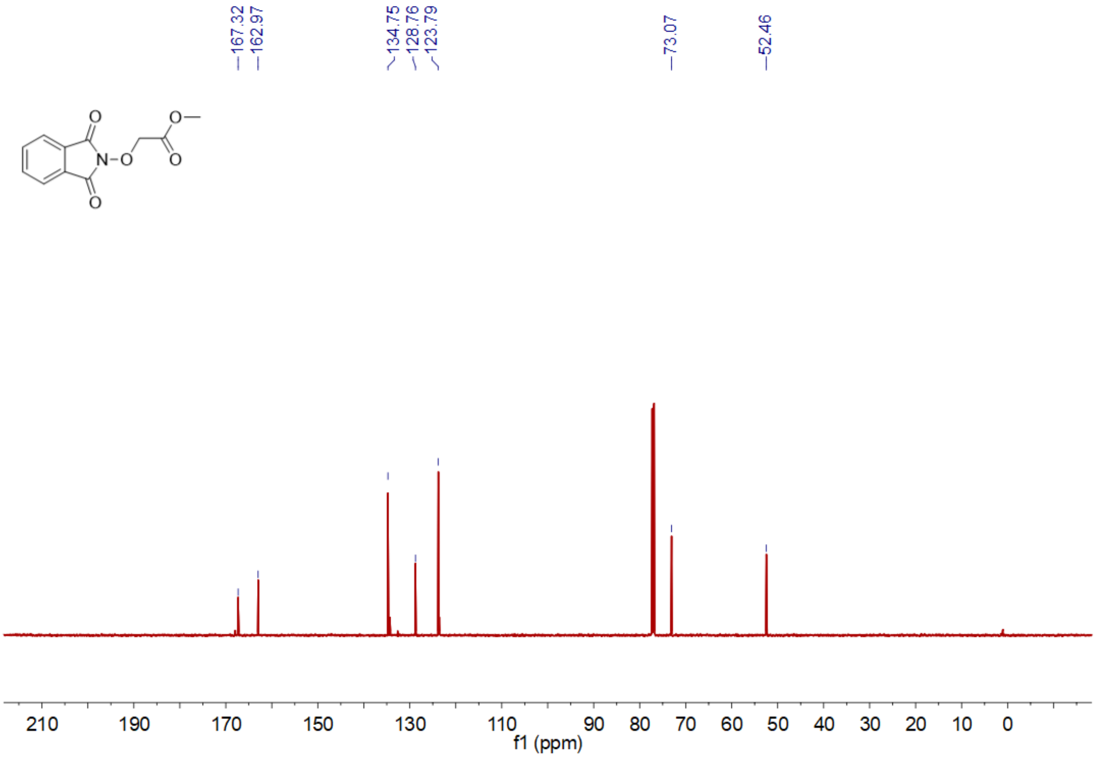

Supplementary Figure 41 ^{13}C NMR (151 MHz, $CDCl_3$, 25 °C) of compound **H3**

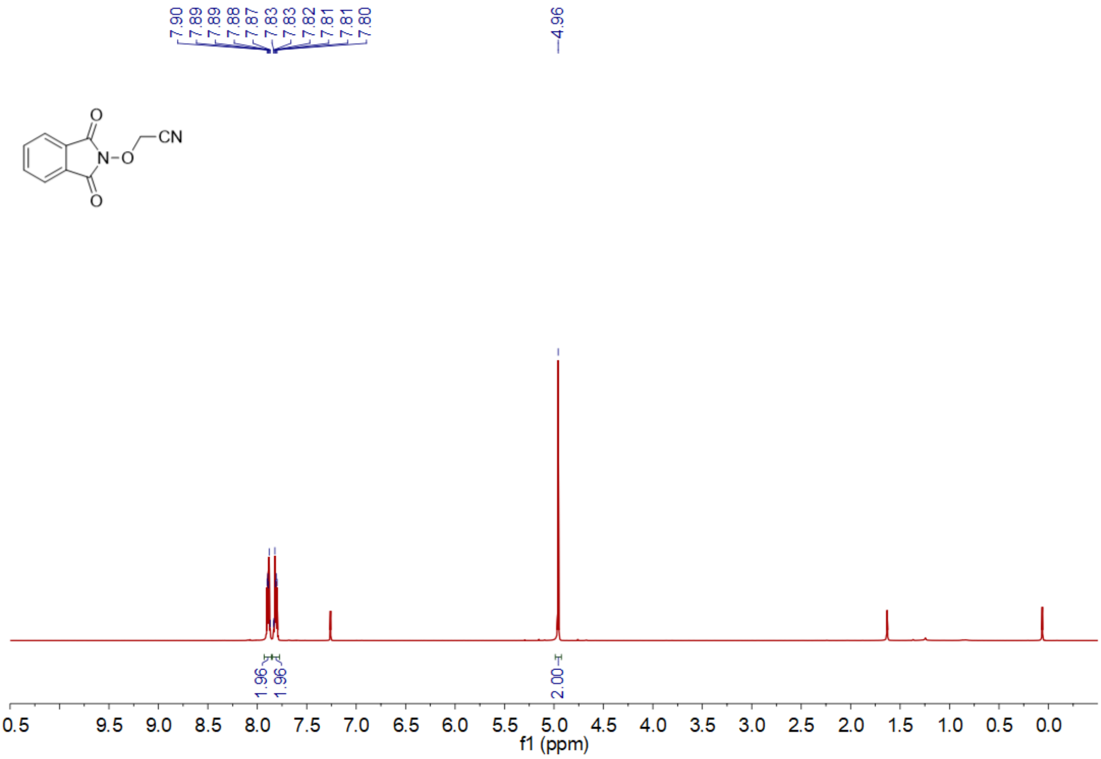

Supplementary Figure 42 ^{1}H NMR (400 MHz, $CDCl_3$, 25 °C) of compound **H4**

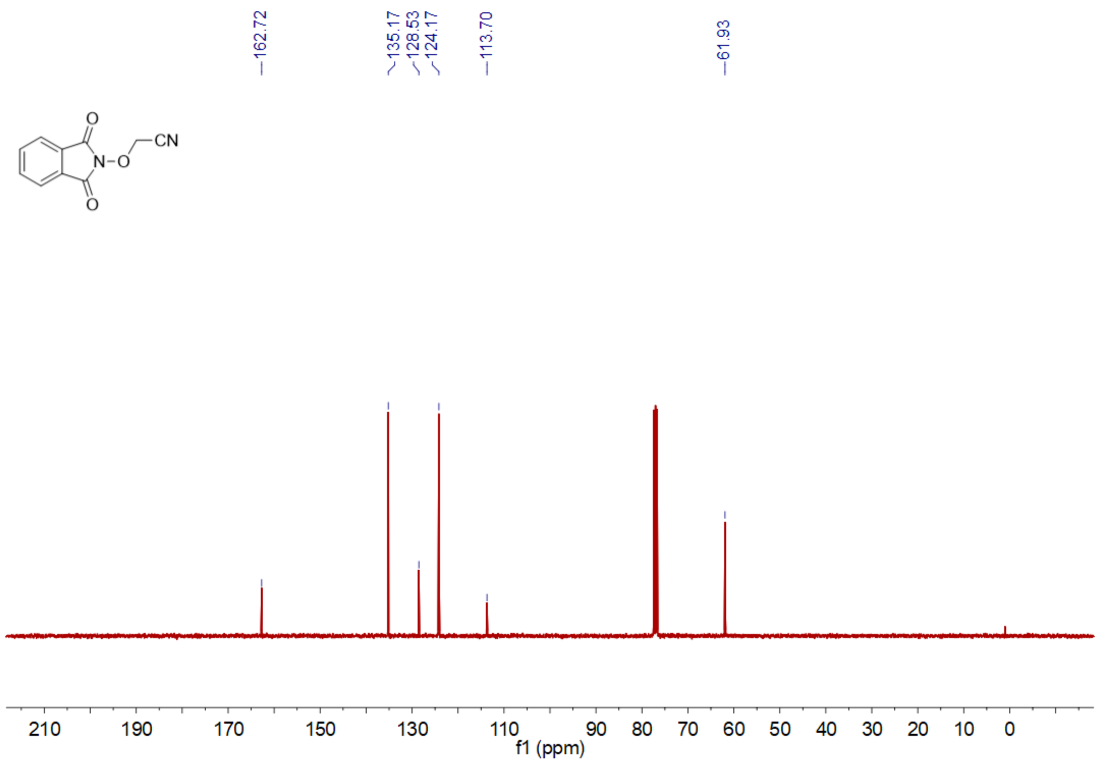

Supplementary Figure 43 ^{13}C NMR (101 MHz, $CDCl_3$, 25 °C) of compound **H4**

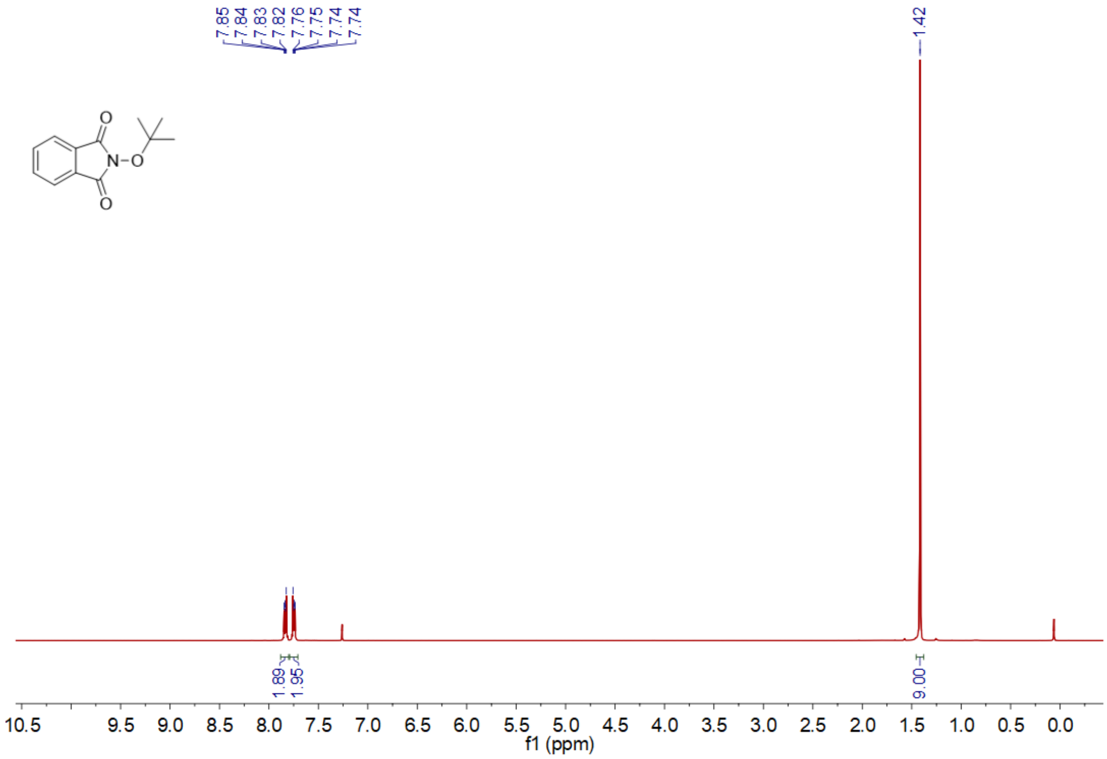

Supplementary Figure 44 ^{1}H NMR (400 MHz, $CDCl_3$, 25 °C) of compound **H6**

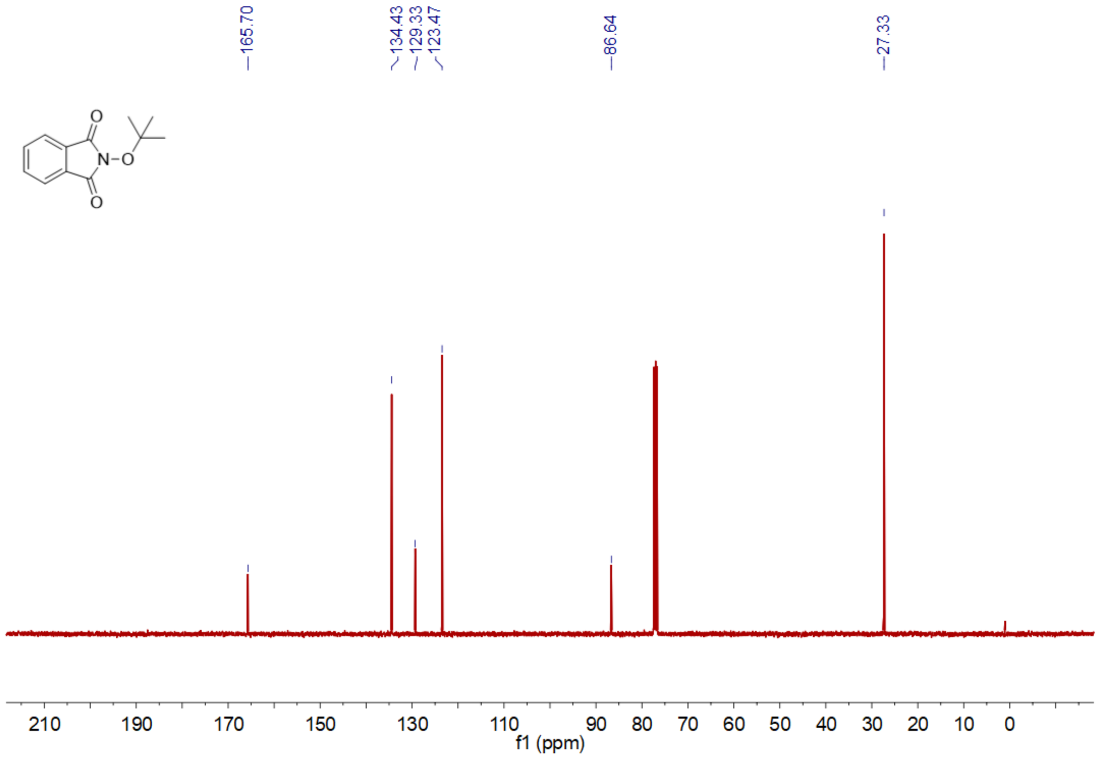

Supplementary Figure 45 ^{13}C NMR (101 MHz, $CDCl_3$, 25 °C) of compound **H6**

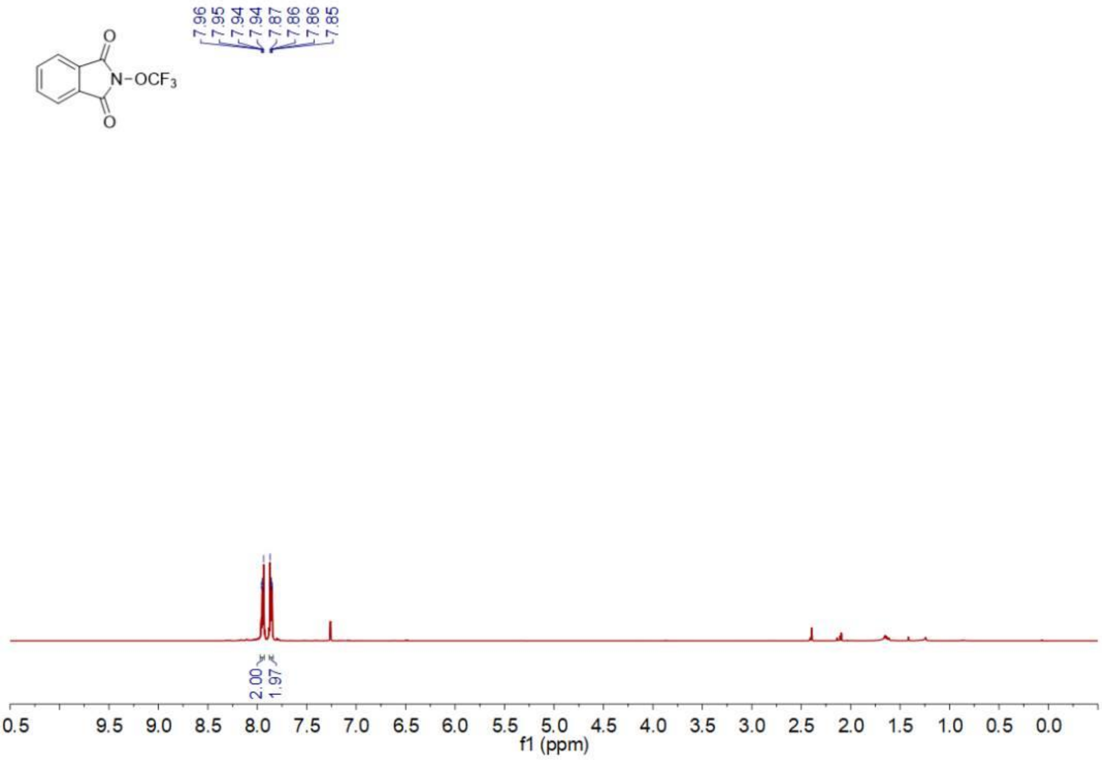

Supplementary Figure 46 ^{1}H NMR (400 MHz, $CDCl_3$, 25 °C) of compound **H9**

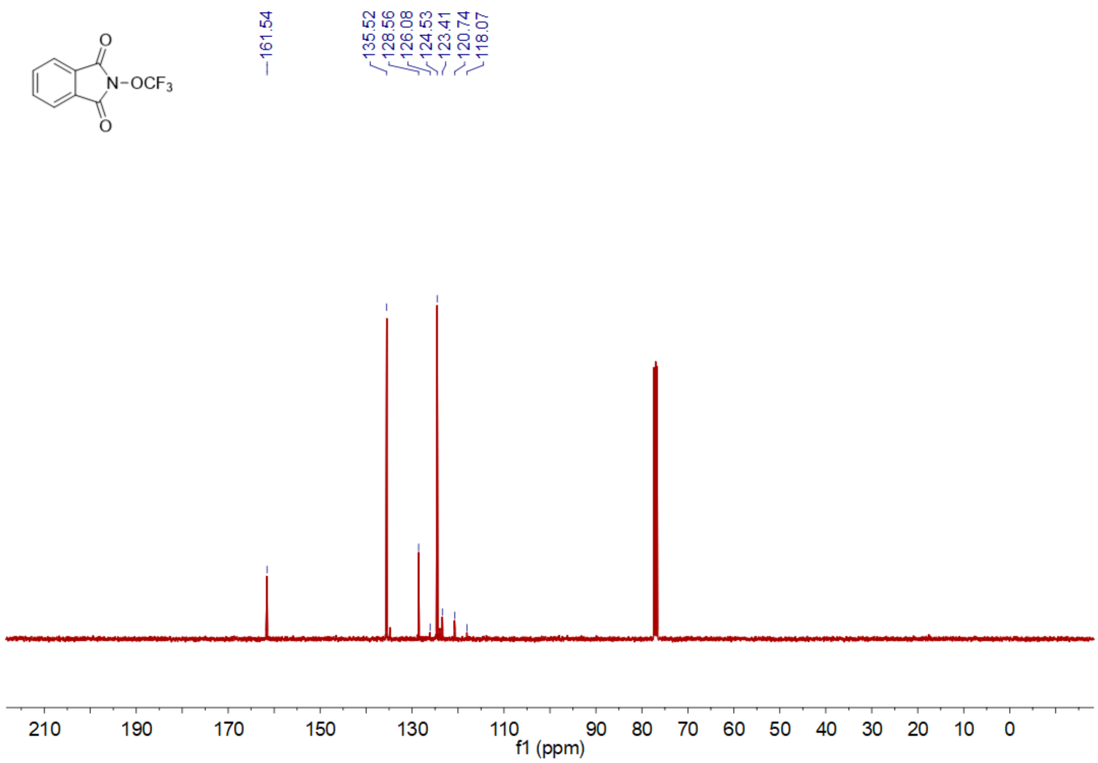

Supplementary Figure 47 ^{13}C NMR (101 MHz, $CDCl_3$, 25 °C) of compound **H9**

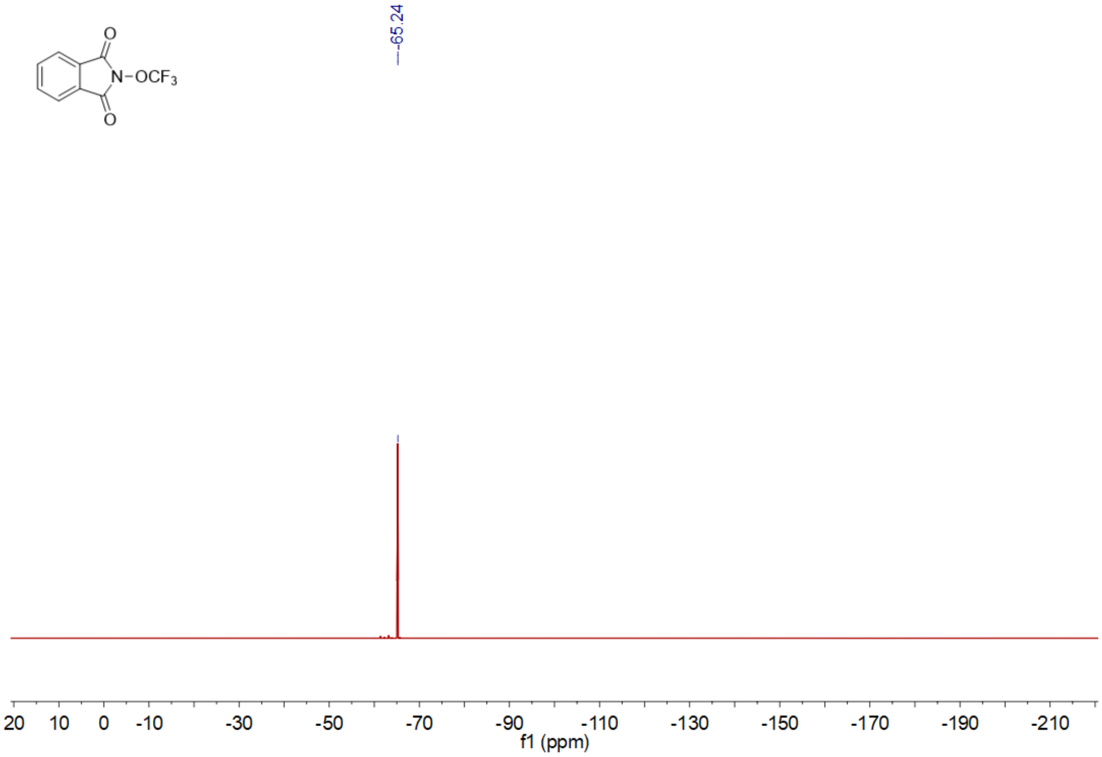

Supplementary Figure 48 ^{19}F NMR (377 MHz, $CDCl_3$, 25 °C) of compound **H9**

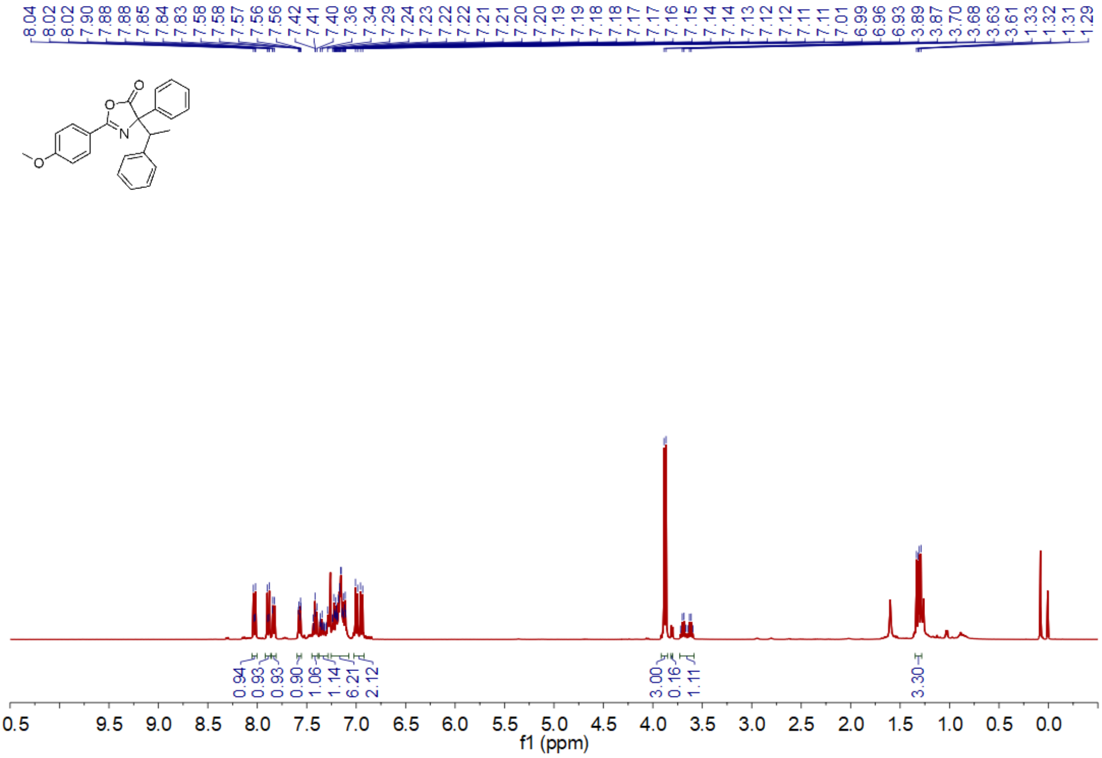

Supplementary Figure 49 ^{1}H NMR (400 MHz, $CDCl_3$, 25 °C) of compound **3**

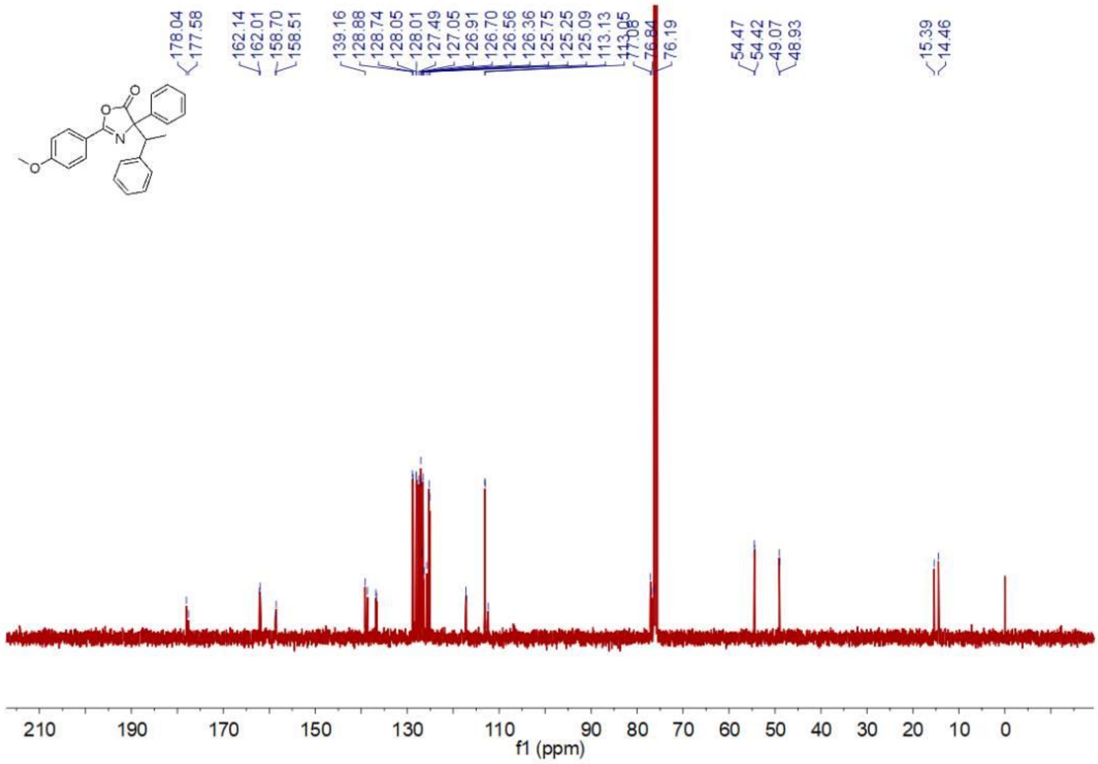

Supplementary Figure 50 ^{13}C NMR (101 MHz, $CDCl_3$, 25 °C) of compound **3**

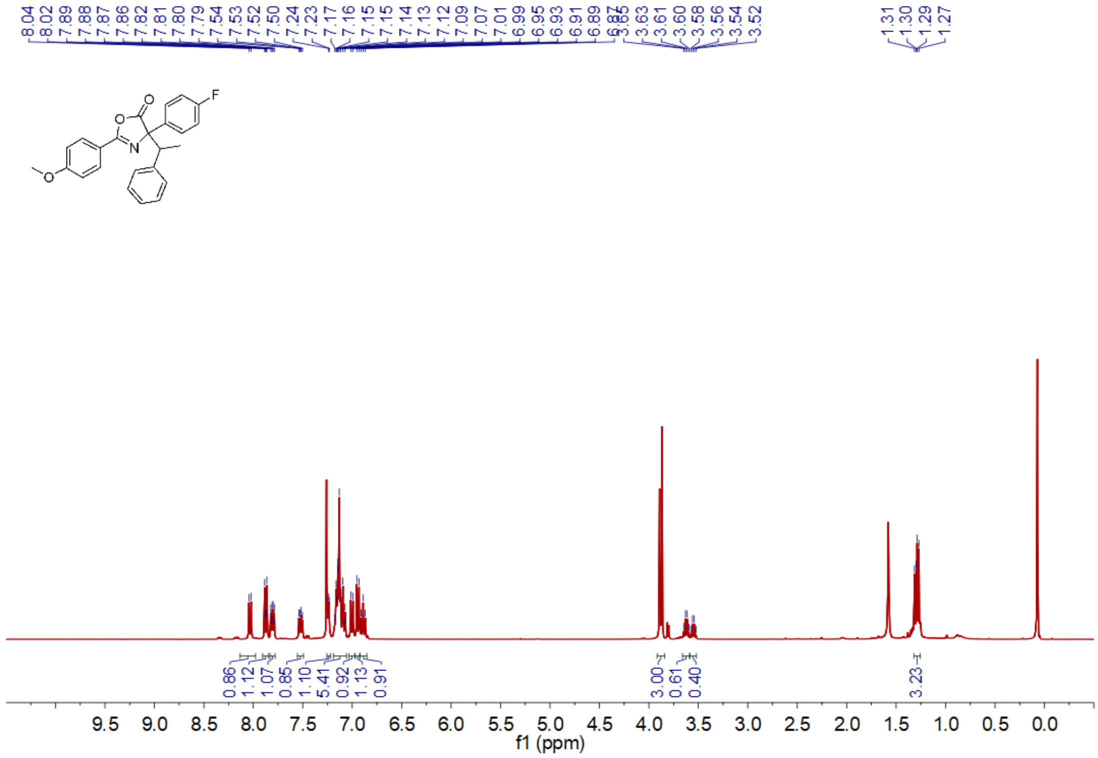

Supplementary Figure 51 ^{1}H NMR (400 MHz, $CDCl_3$, 25 °C) of compound **4**

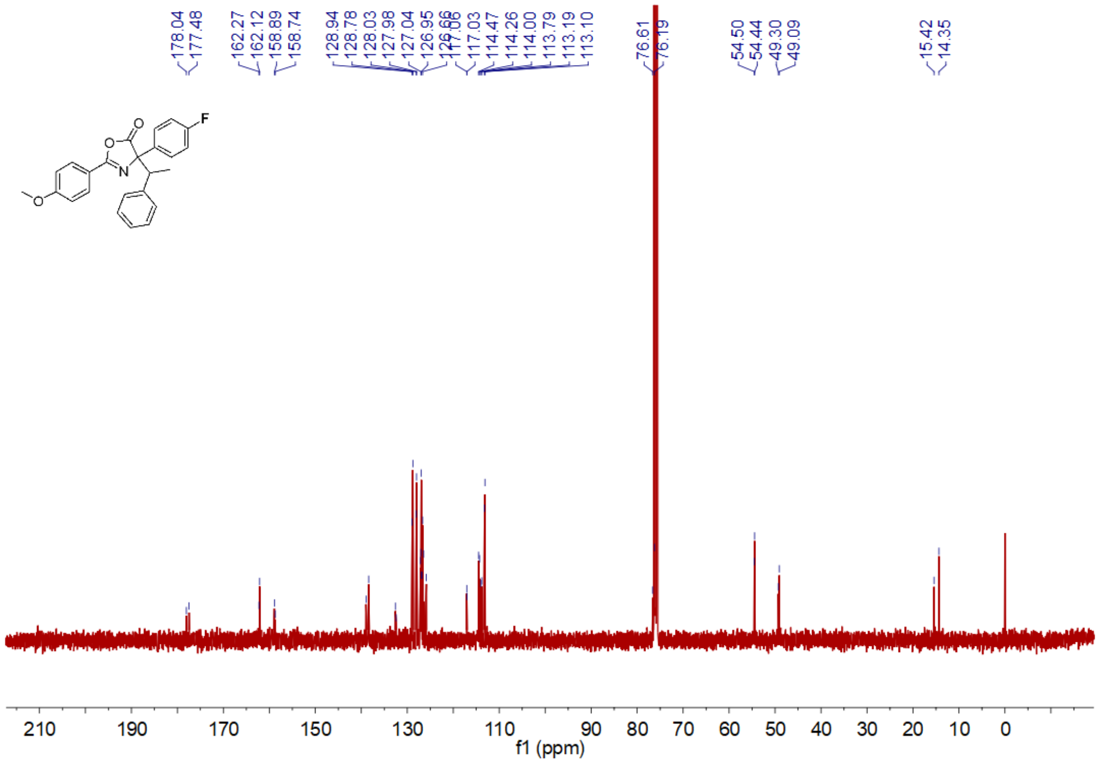

Supplementary Figure 52 ^{13}C NMR (101 MHz, $CDCl_3$, 25 °C) of compound **4**

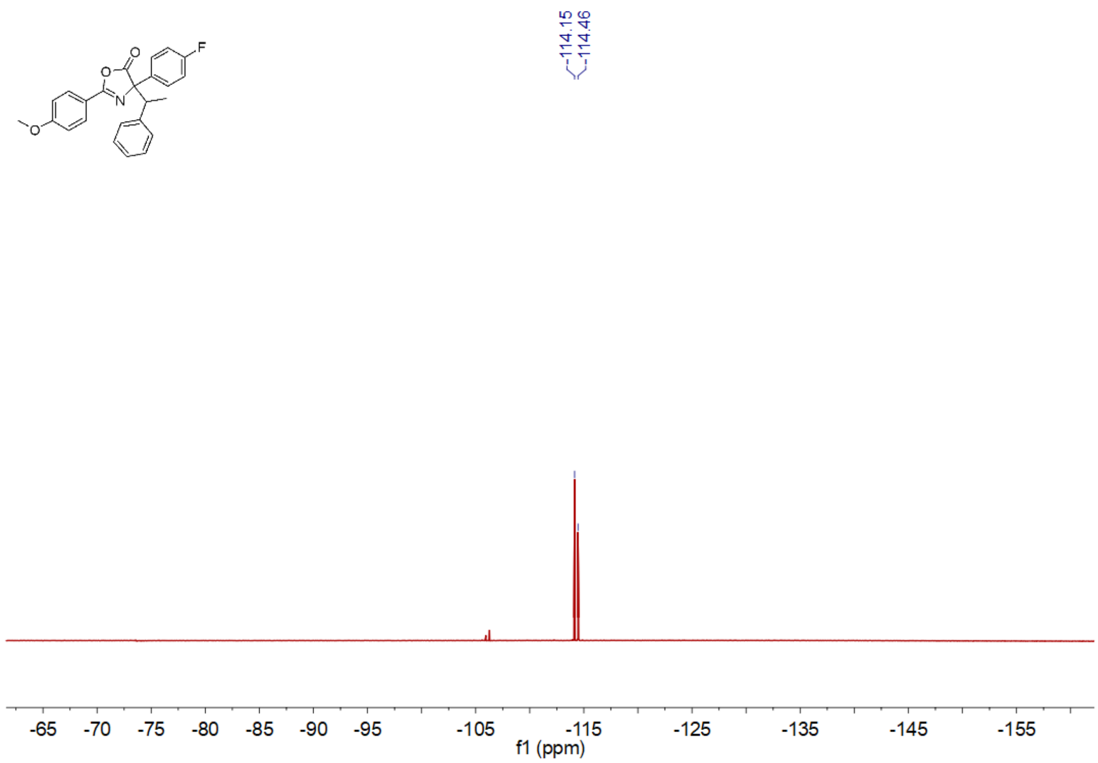

Supplementary Figure 53 ^{19}F NMR (377 MHz, $CDCl_3$, 25 °C) of compound **4**

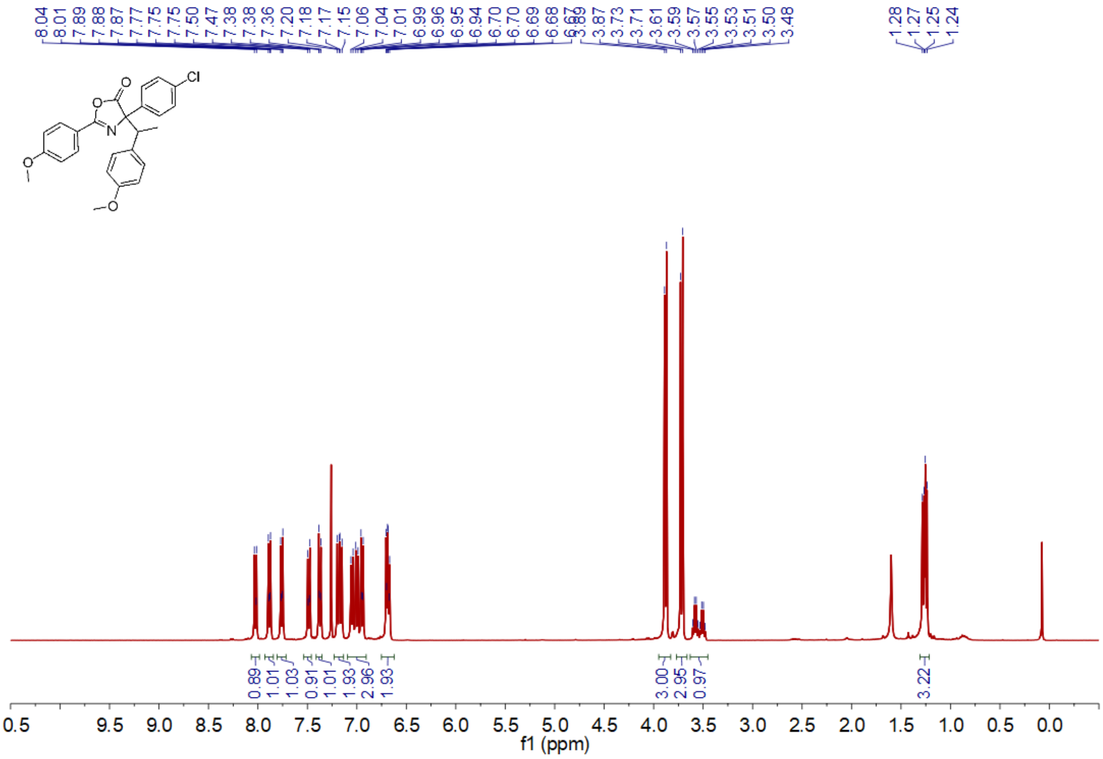

Supplementary Figure 54 ^{1}H NMR (400 MHz, $CDCl_3$, 25 °C) of compound **5**

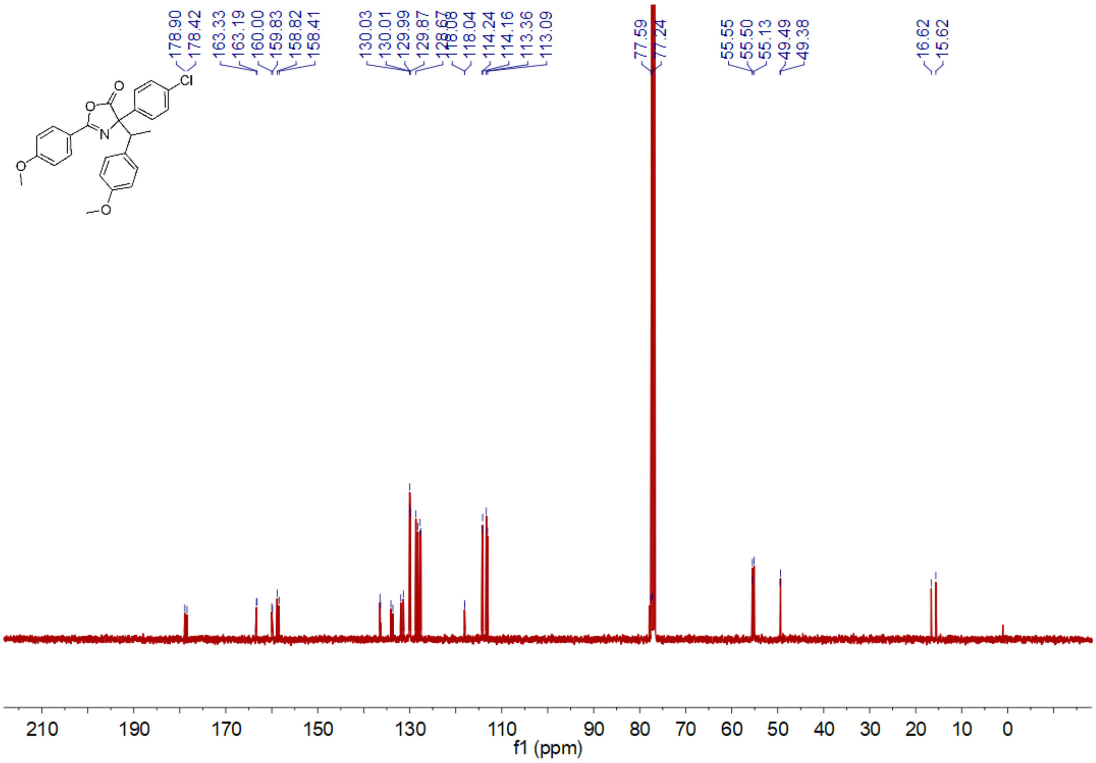

Supplementary Figure 55 ^{13}C NMR (101 MHz, $CDCl_3$, 25 °C) of compound **5**

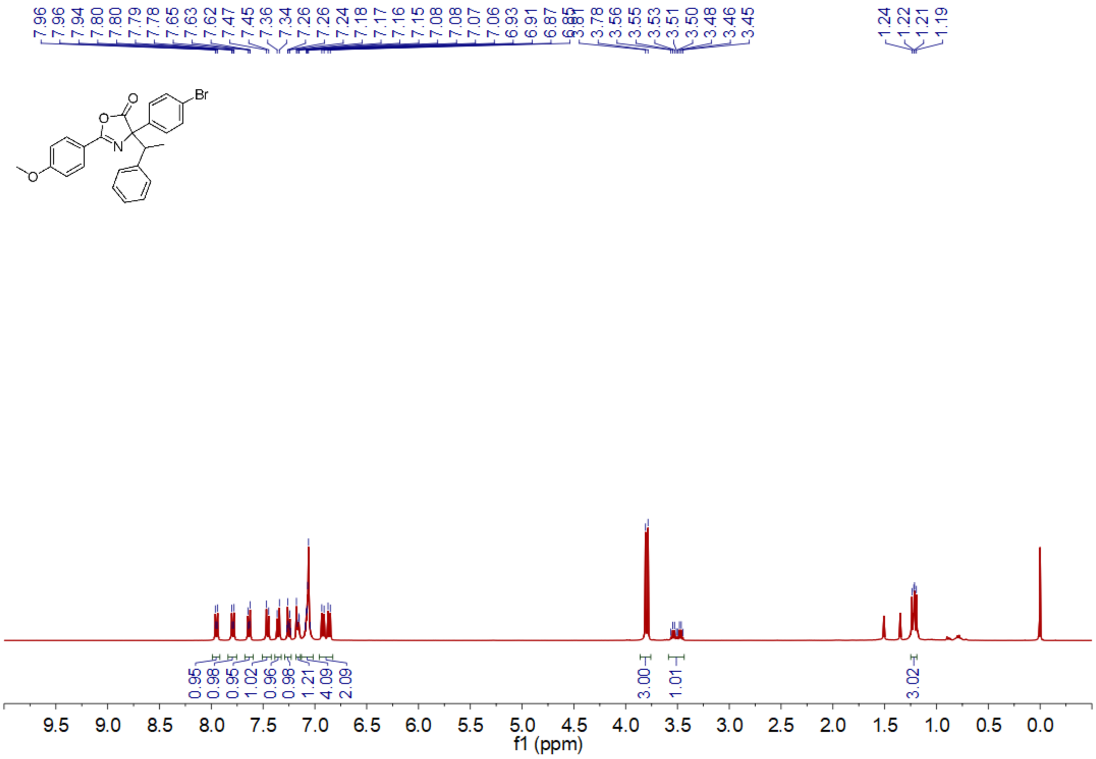

Supplementary Figure 56 ^{1}H NMR (400 MHz, $CDCl_3$, 25 °C) of compound **6**

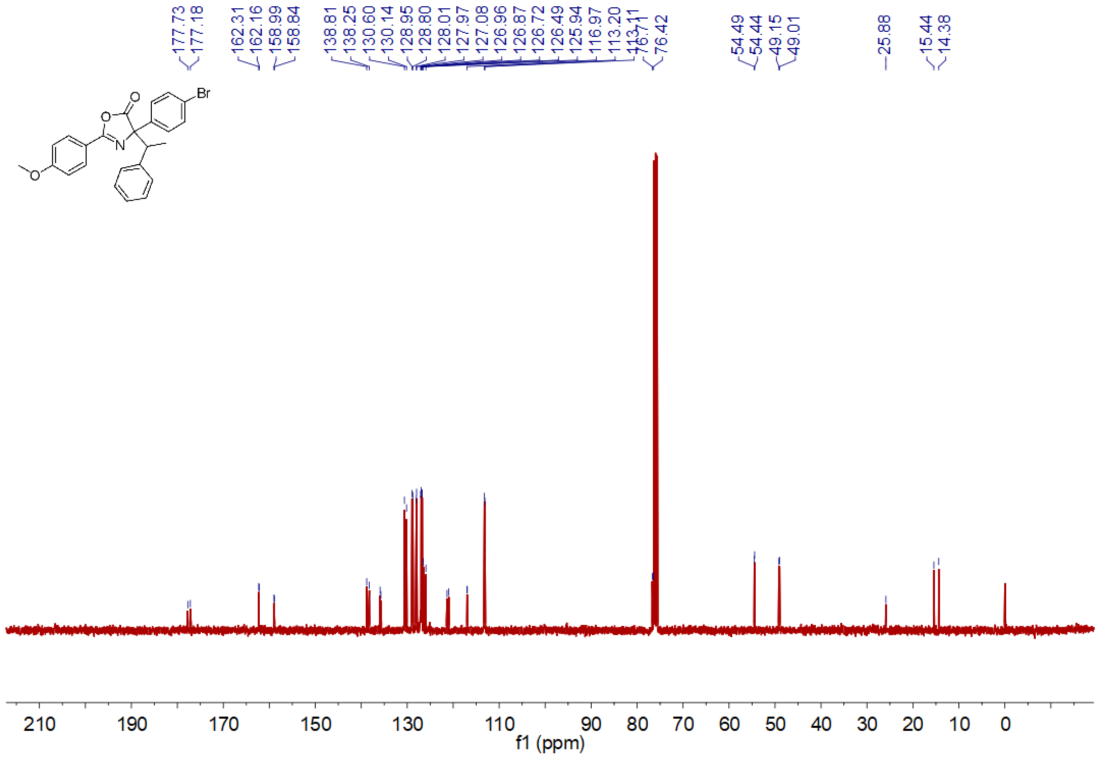

Supplementary Figure 57 ^{13}C NMR (101 MHz, $CDCl_3$, 25 °C) of compound **6**

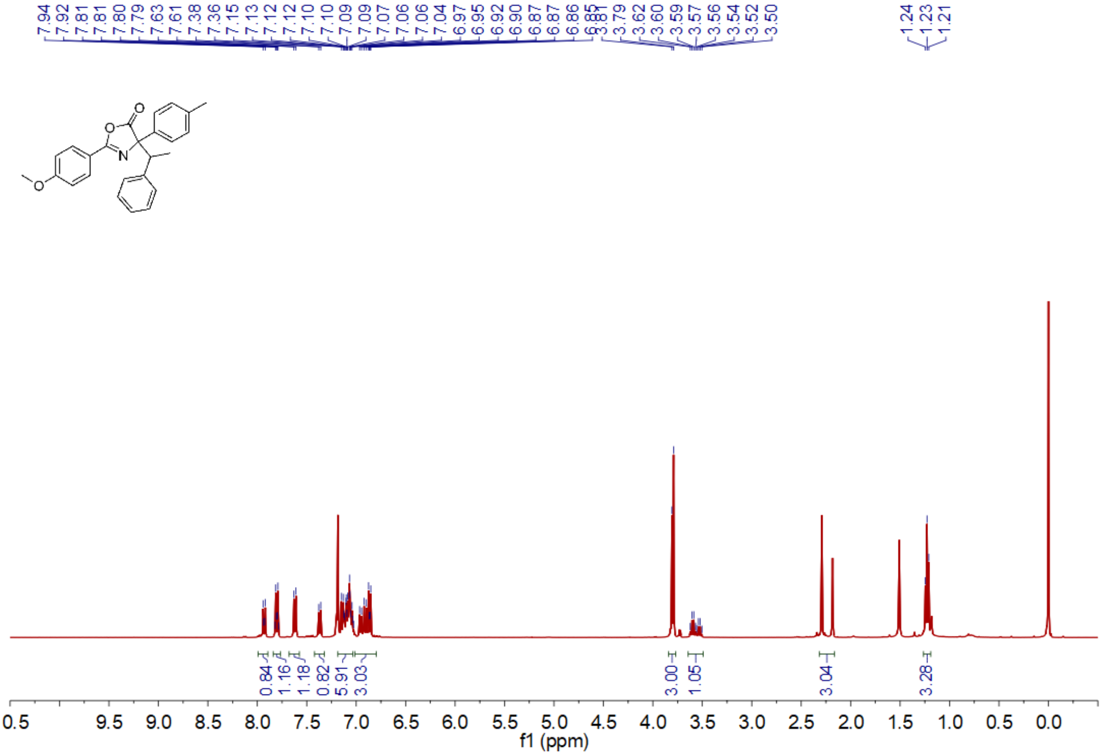

Supplementary Figure 58 ^{1}H NMR (400 MHz, $CDCl_3$, 25 °C) of compound **7**

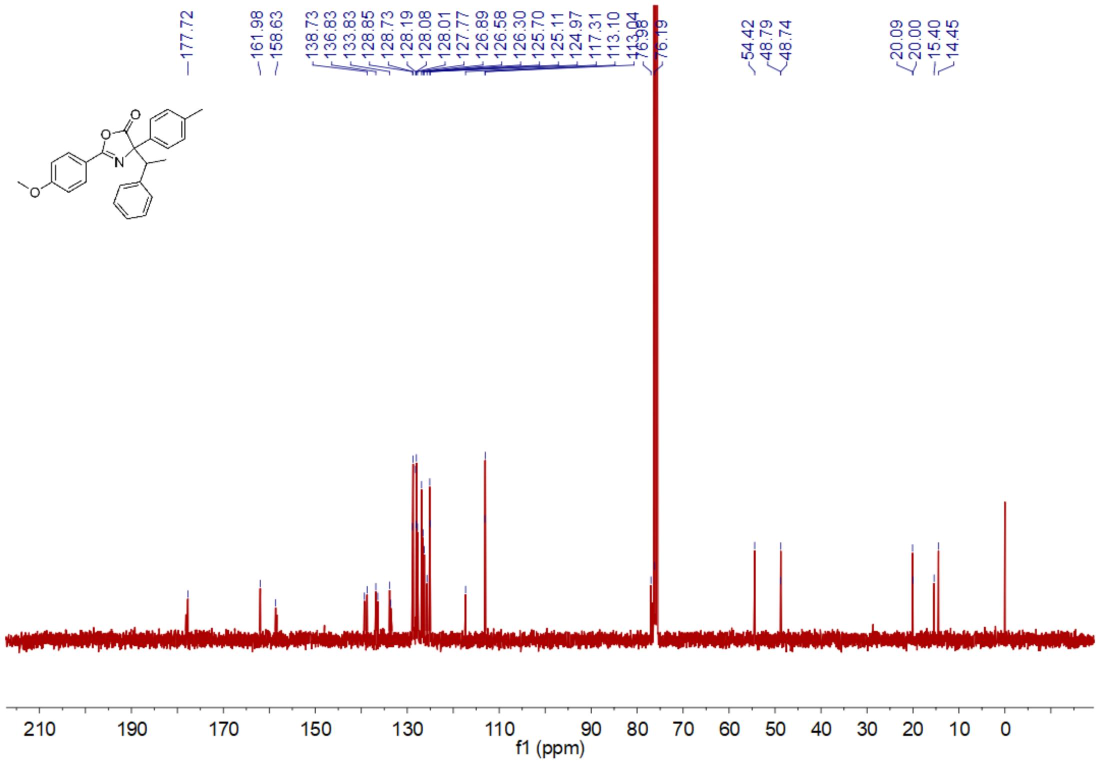

Supplementary Figure 59 ^{13}C NMR (101 MHz, $CDCl_3$, 25 °C) of compound **7**

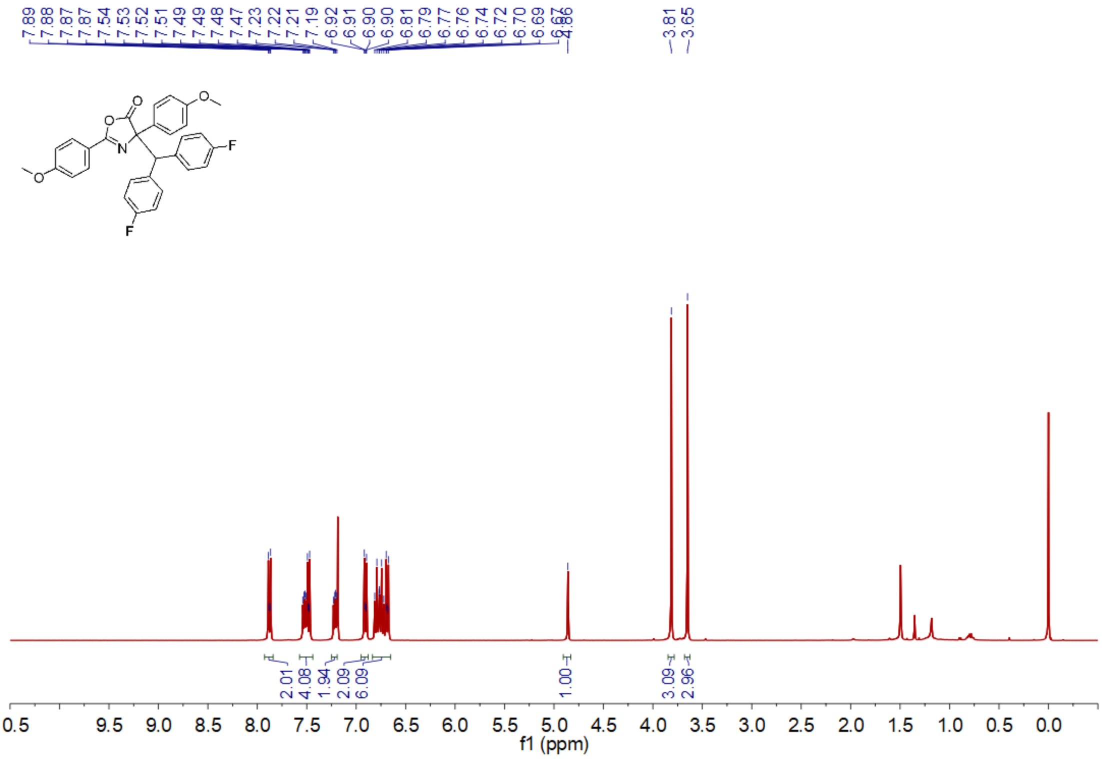

Supplementary Figure 60 ^{1}H NMR (400 MHz, $CDCl_3$, 25 °C) of compound **8**

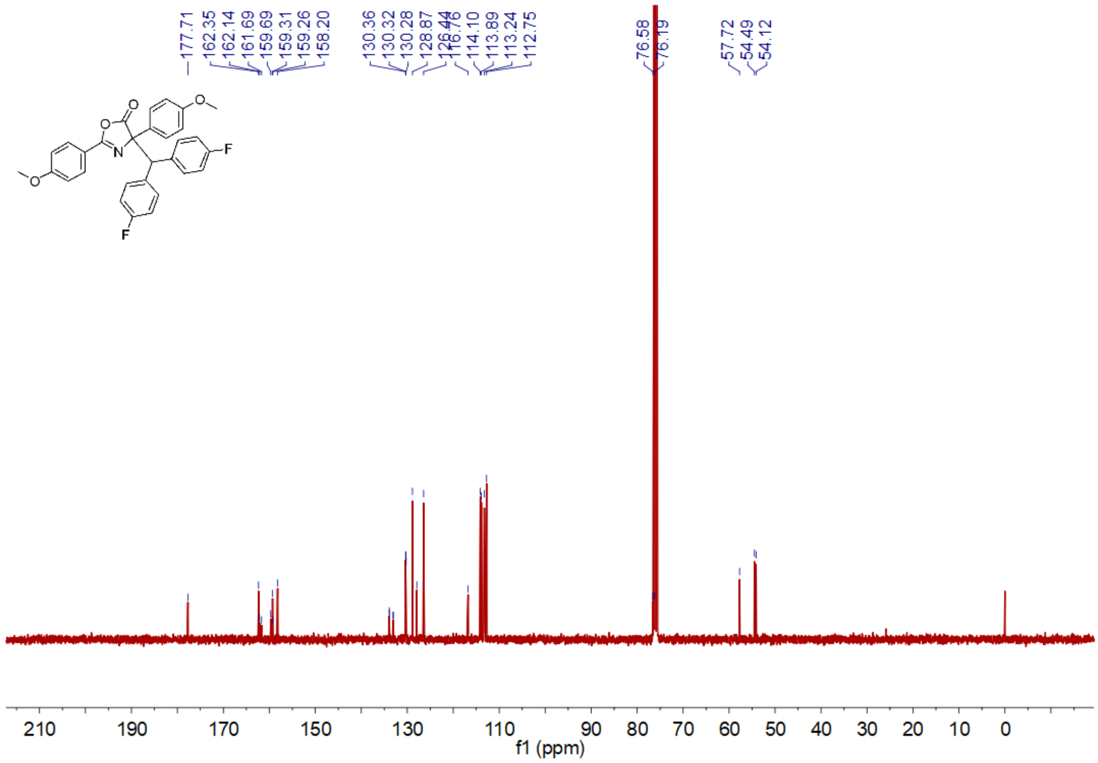

Supplementary Figure 61 ^{13}C NMR (101 MHz, $CDCl_3$, 25 °C) of compound **8**

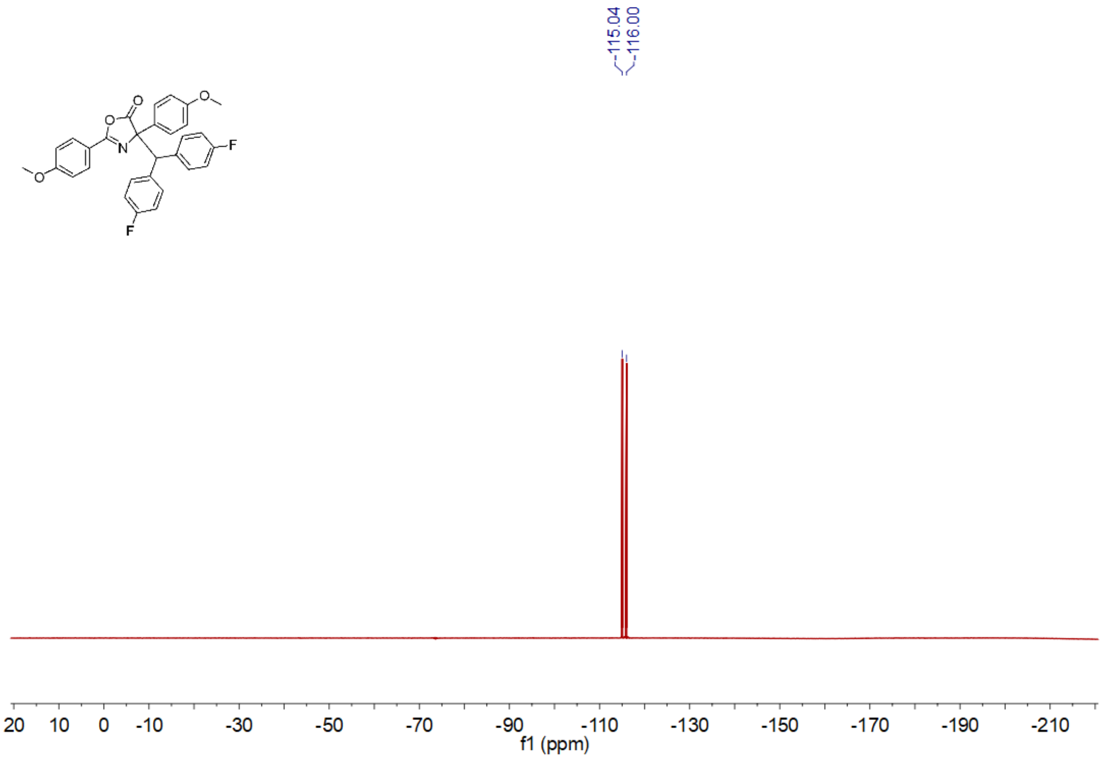

Supplementary Figure 62 ^{19}F NMR (377 MHz, $CDCl_3$, 25 °C) of compound **8**

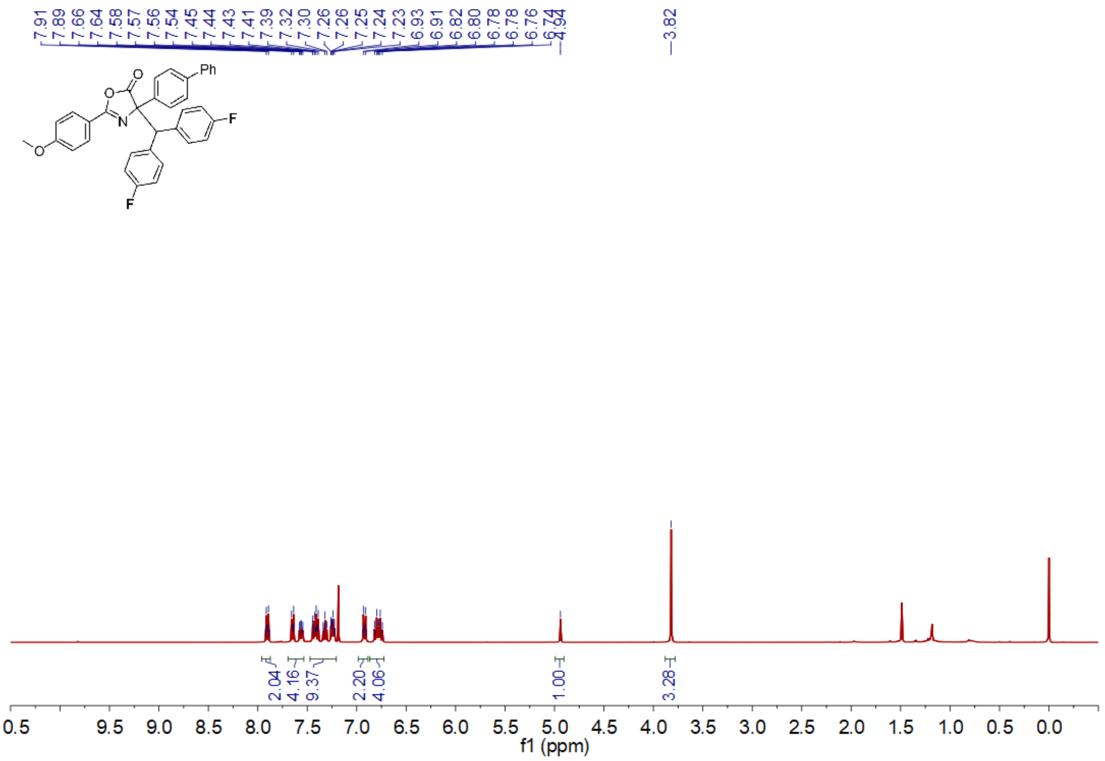

Supplementary Figure 63 ^{1}H NMR (400 MHz, $CDCl_3$, 25 °C) of compound **9**

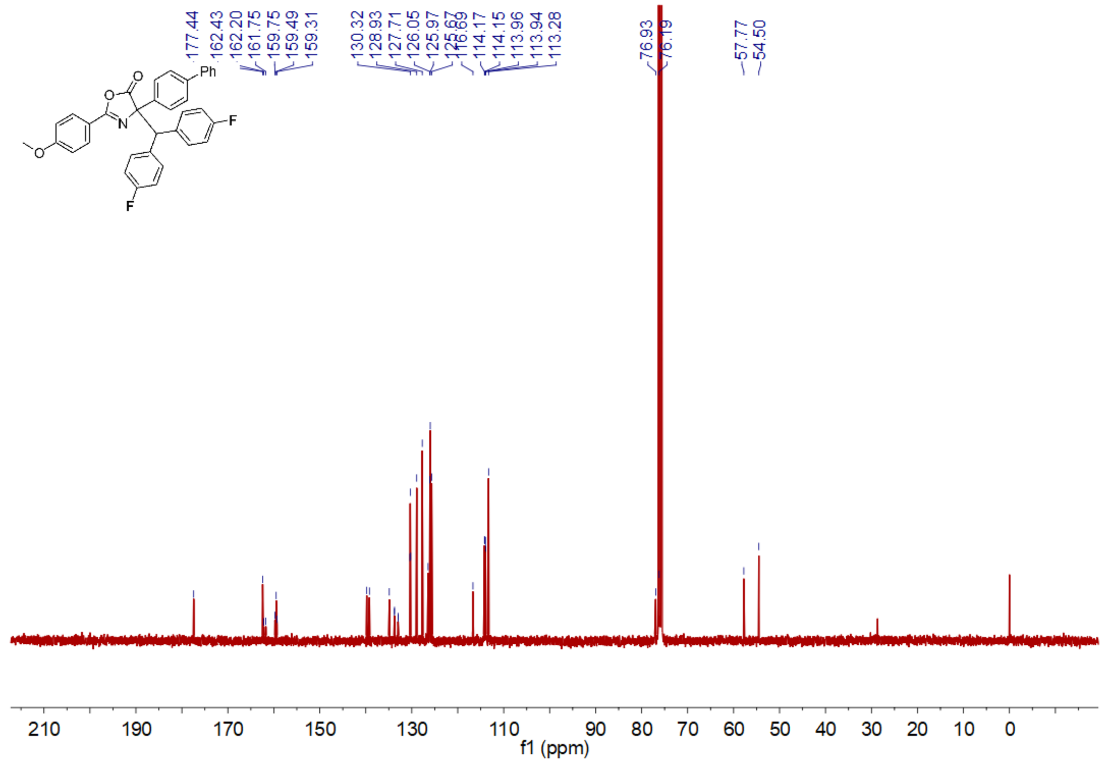

Supplementary Figure 64 ^{13}C NMR (101 MHz, $CDCl_3$, 25 °C) of compound **9**

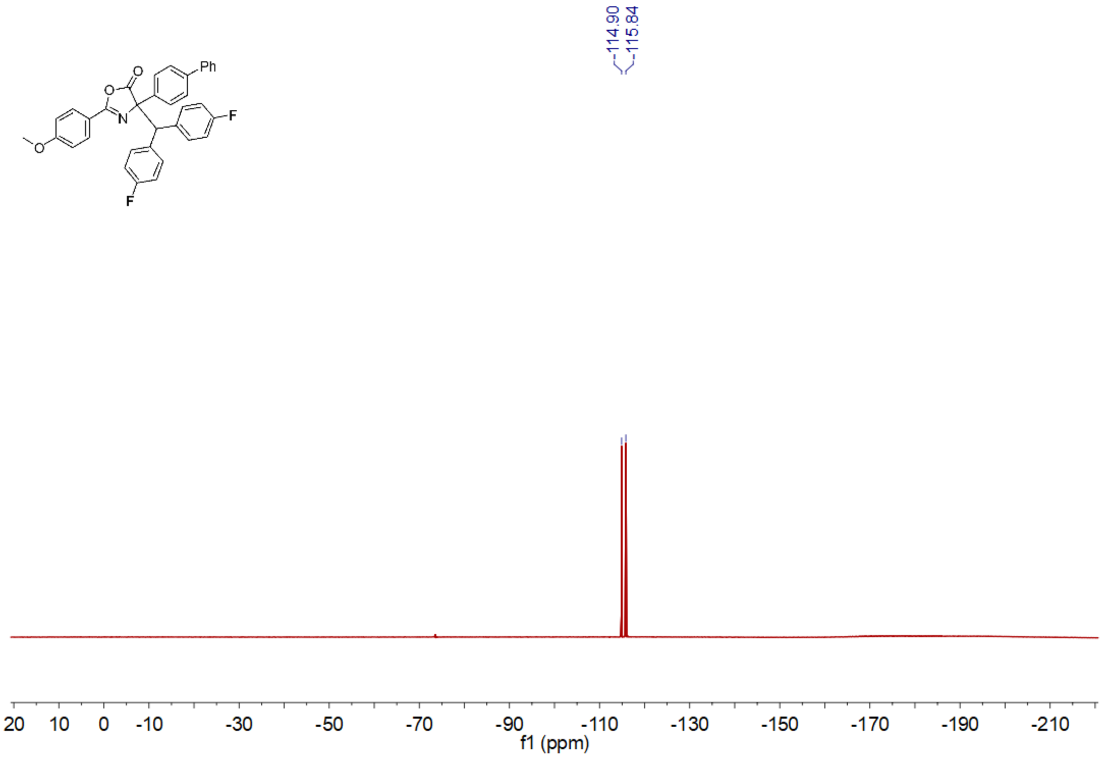

Supplementary Figure 65 ^{19}F NMR (377 MHz, $CDCl_3$, 25 °C) of compound **9**

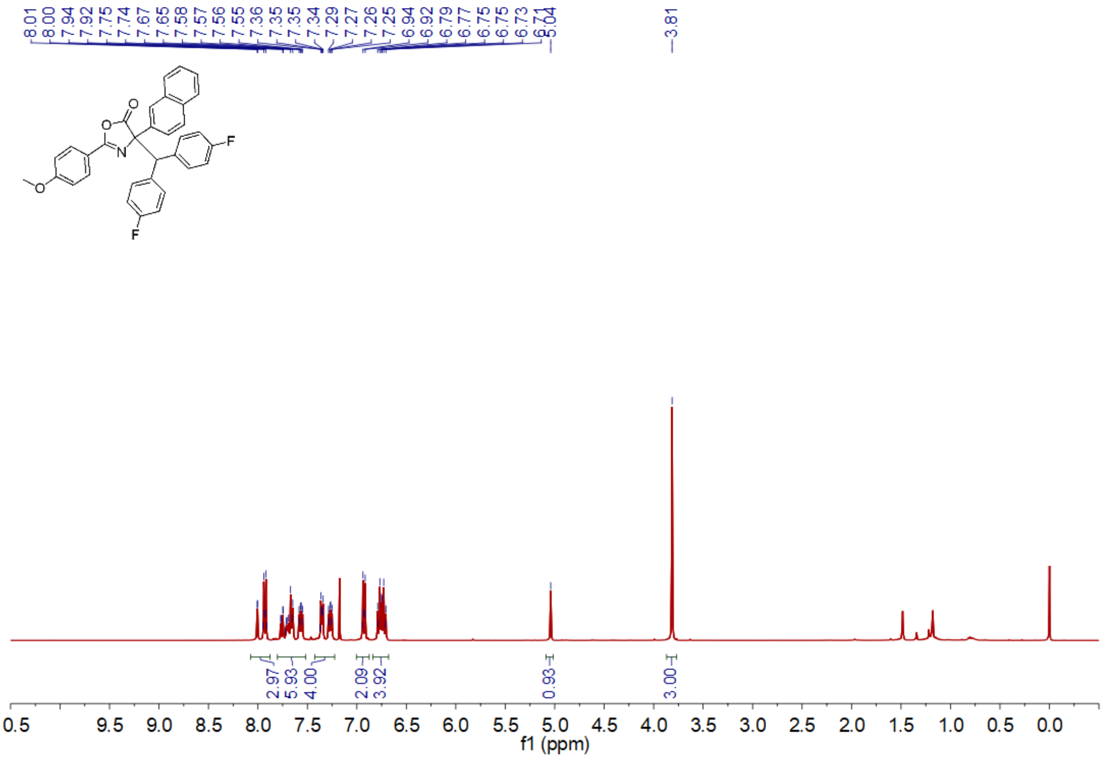

Supplementary Figure 66 ^{1}H NMR (400 MHz, $CDCl_3$, 25 °C) of compound **10**

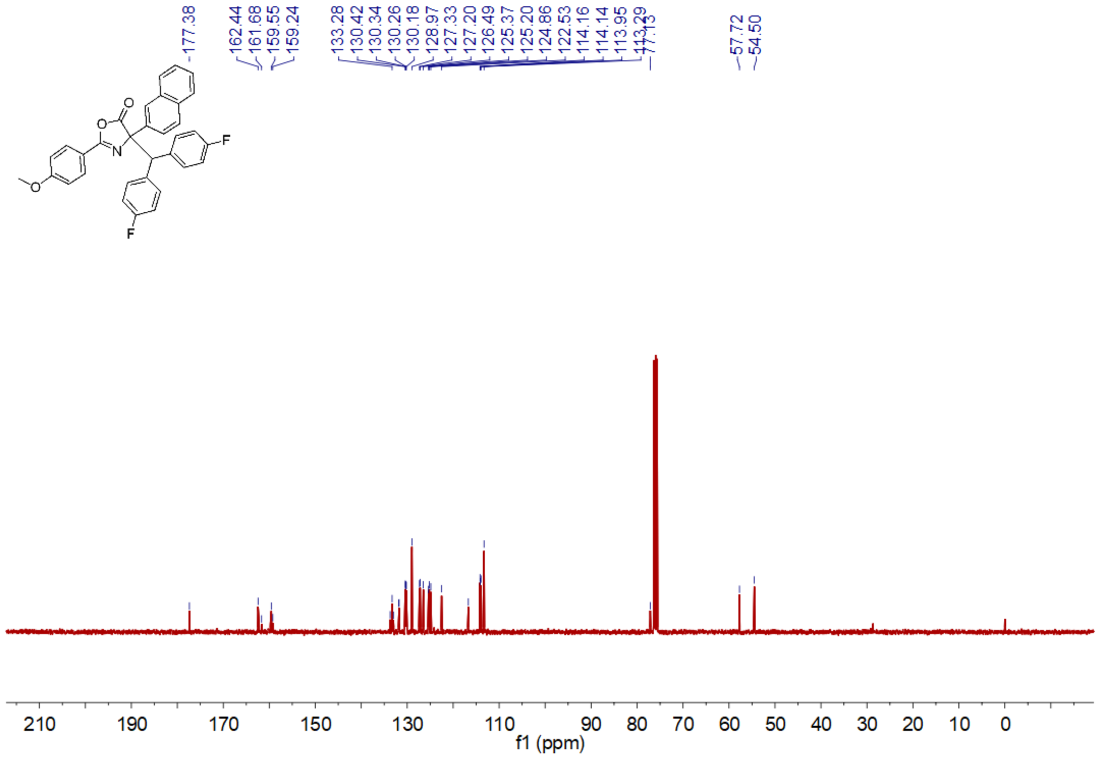

Supplementary Figure 67 ^{13}C NMR (101 MHz, $CDCl_3$, 25 °C) of compound **10**

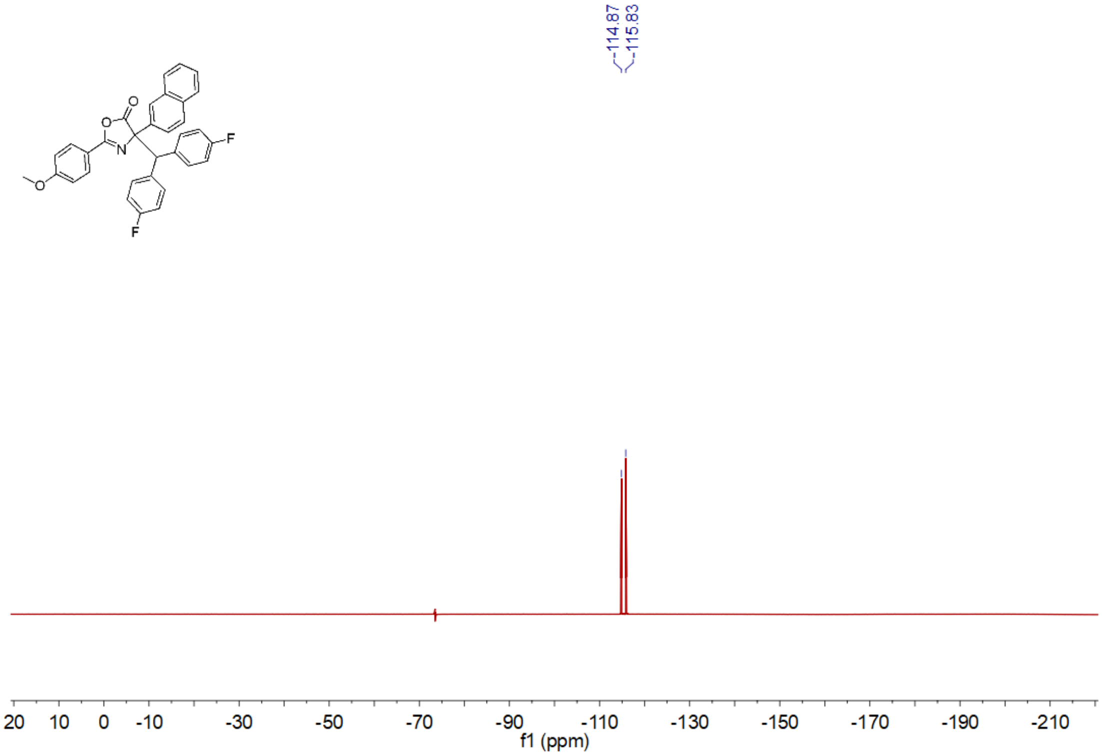

Supplementary Figure 68 ^{19}F NMR (377 MHz, $CDCl_3$, 25 °C) of compound **10**

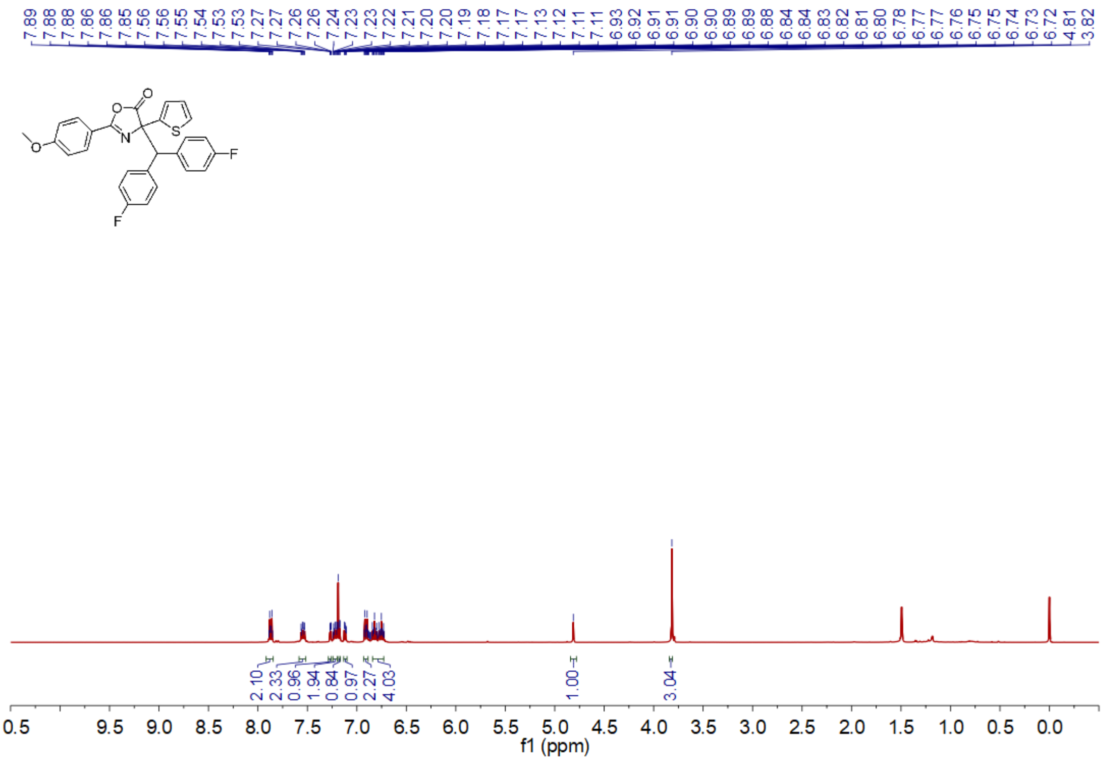

Supplementary Figure 69 ^{1}H NMR (400 MHz, $CDCl_3$, 25 °C) of compound **11**

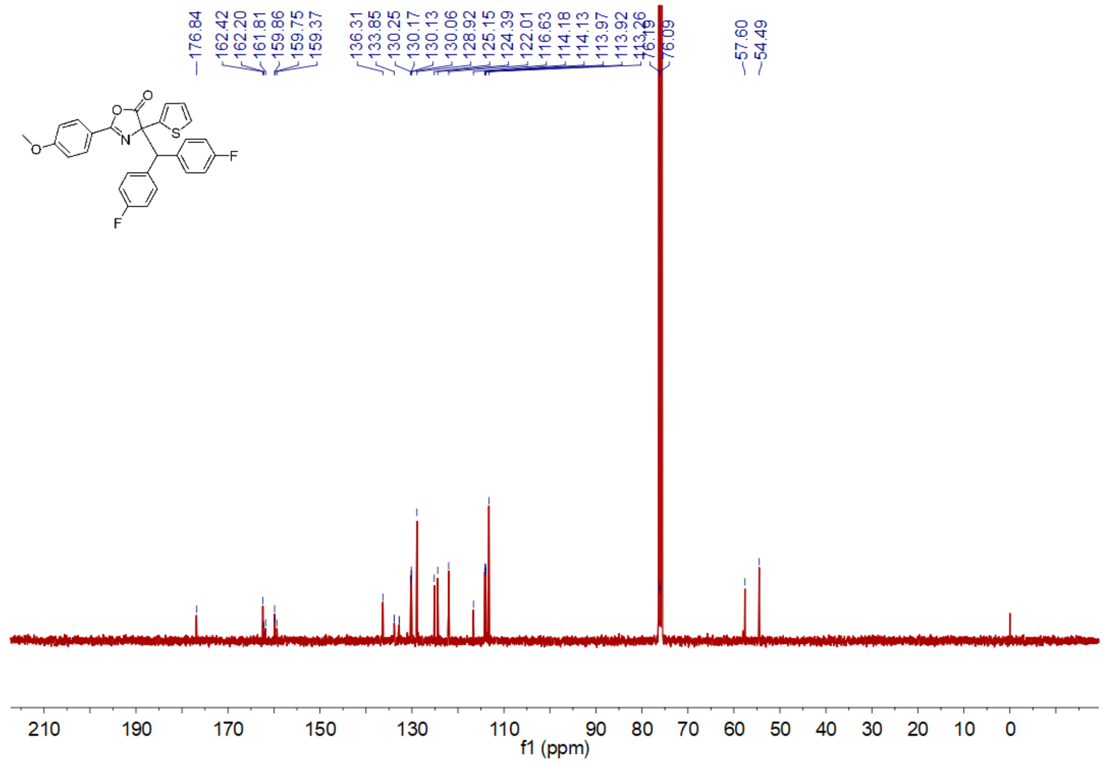

Supplementary Figure 70 ^{13}C NMR (101 MHz, $CDCl_3$, 25 °C) of compound **11**

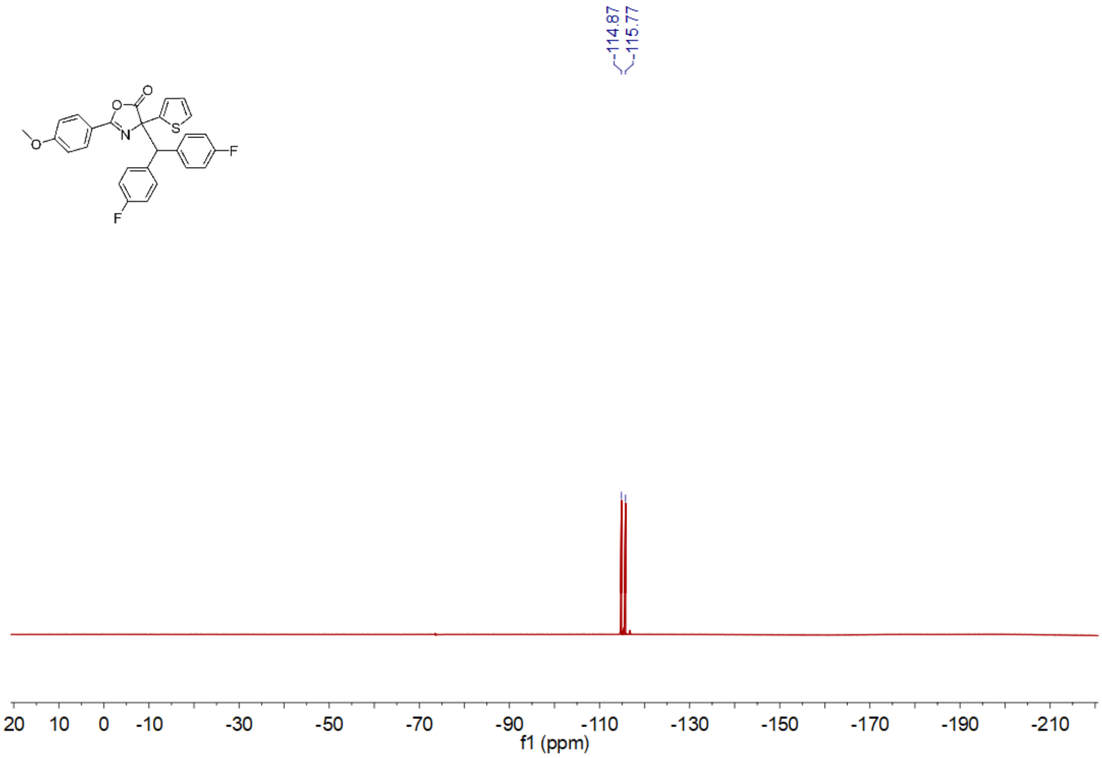

Supplementary Figure 71 ^{19}F NMR (377 MHz, $CDCl_3$, 25 °C) of compound **11**

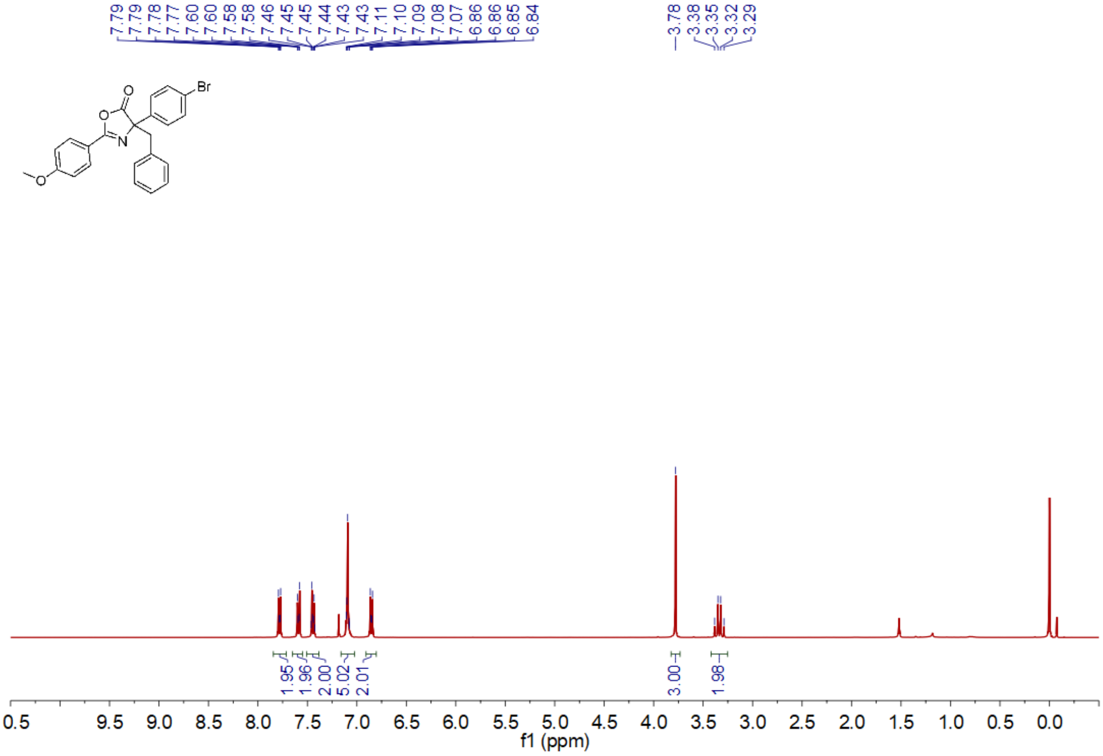

Supplementary Figure 72 ^{1}H NMR (400 MHz, $CDCl_3$, 25 °C) of compound **12**

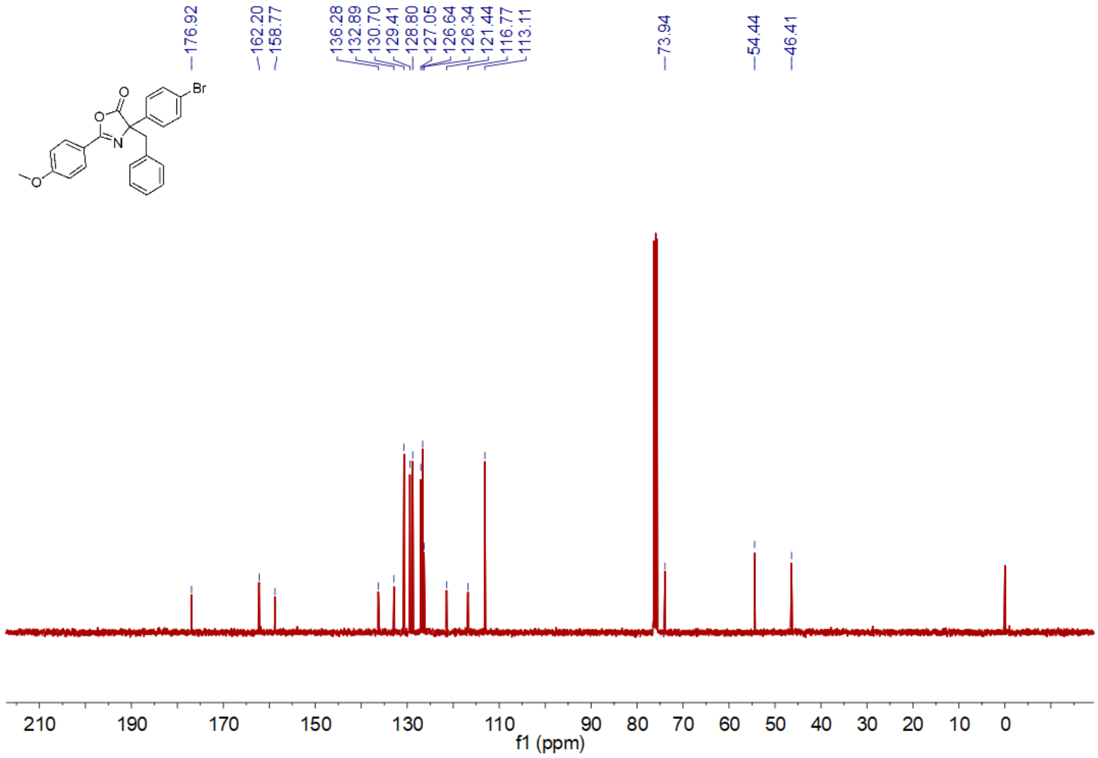

Supplementary Figure 73 ^{13}C NMR (101 MHz, $CDCl_3$, 25 °C) of compound **12**

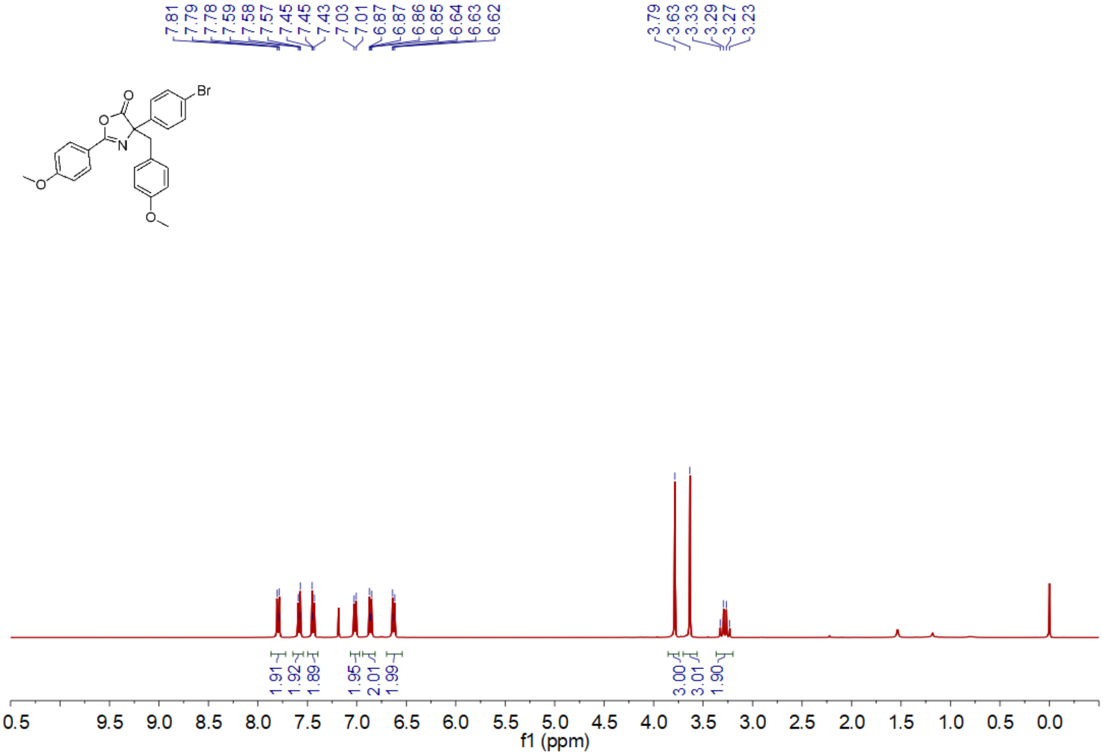

Supplementary Figure 74 ^{1}H NMR (400 MHz, $CDCl_3$, 25 °C) of compound **13**

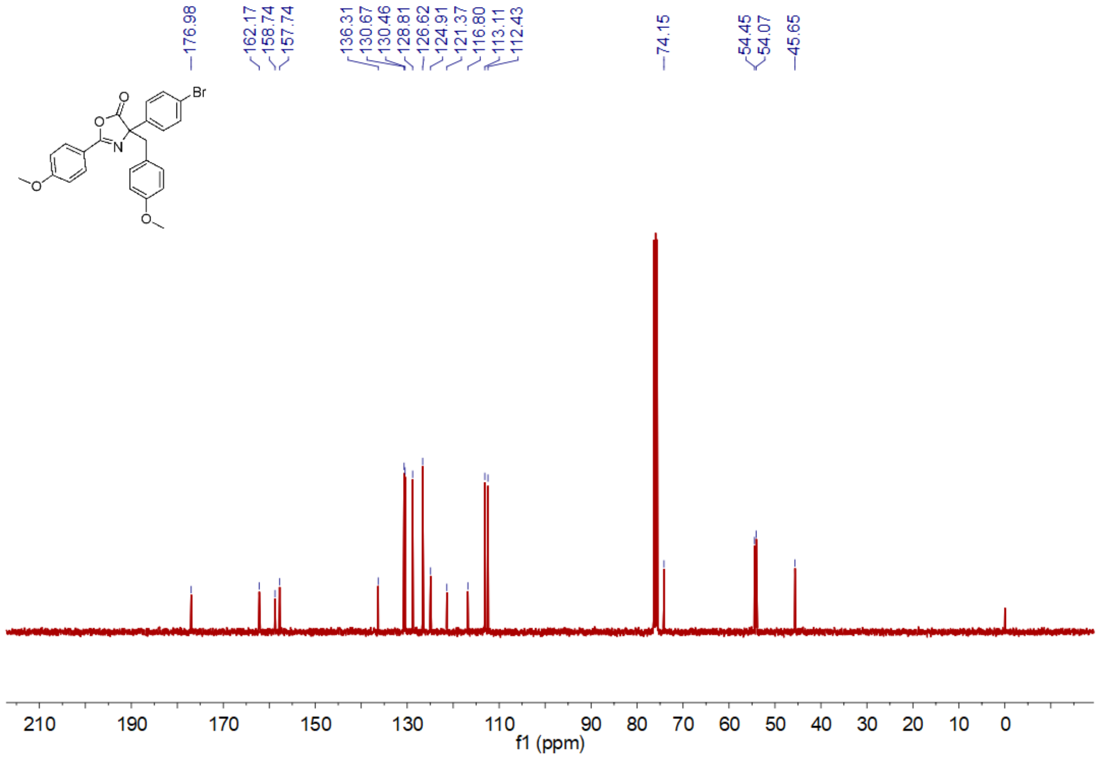

Supplementary Figure 75 ^{13}C NMR (101 MHz, $CDCl_3$, 25 °C) of compound **13**

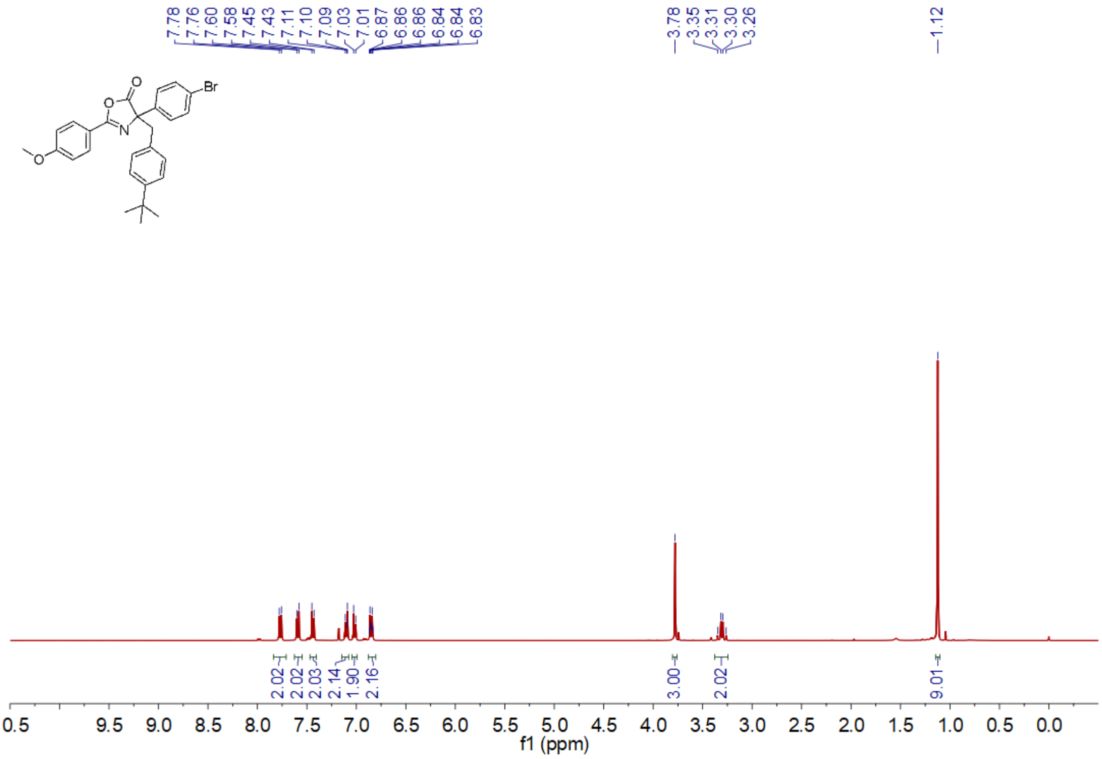

Supplementary Figure 76 ^{1}H NMR (400 MHz, $CDCl_3$, 25 °C) of compound **14**

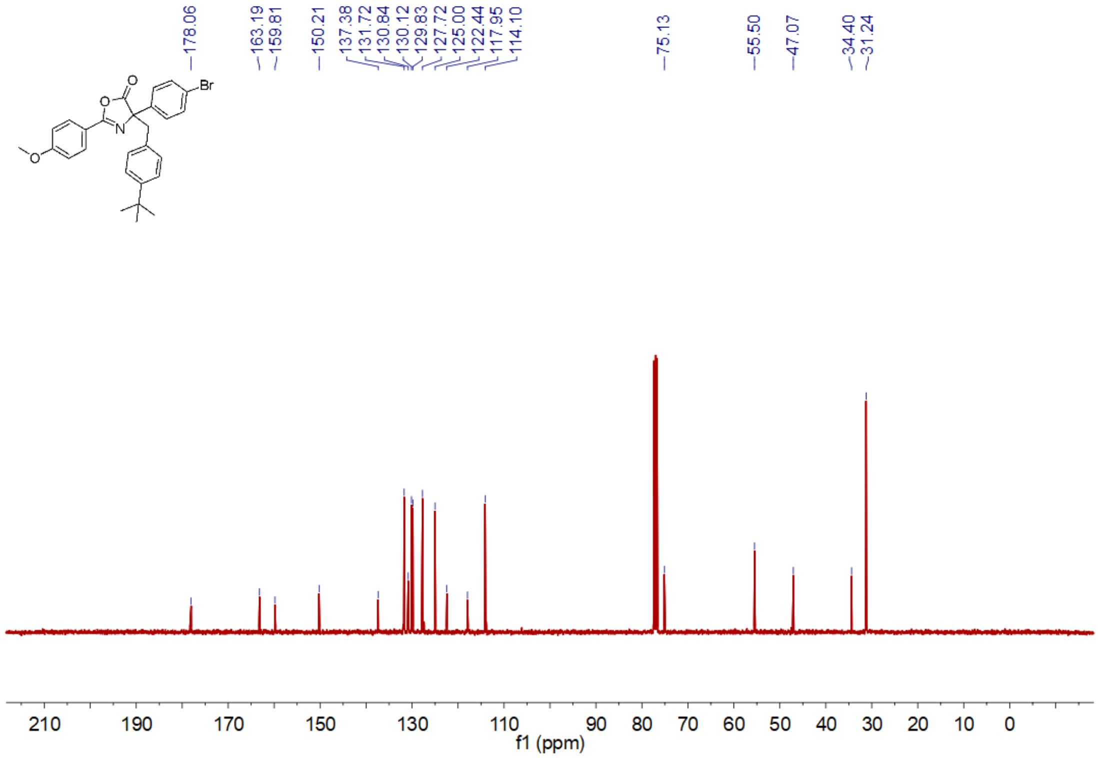

Supplementary Figure 77 ^{13}C NMR (101 MHz, $CDCl_3$, 25 °C) of compound **14**

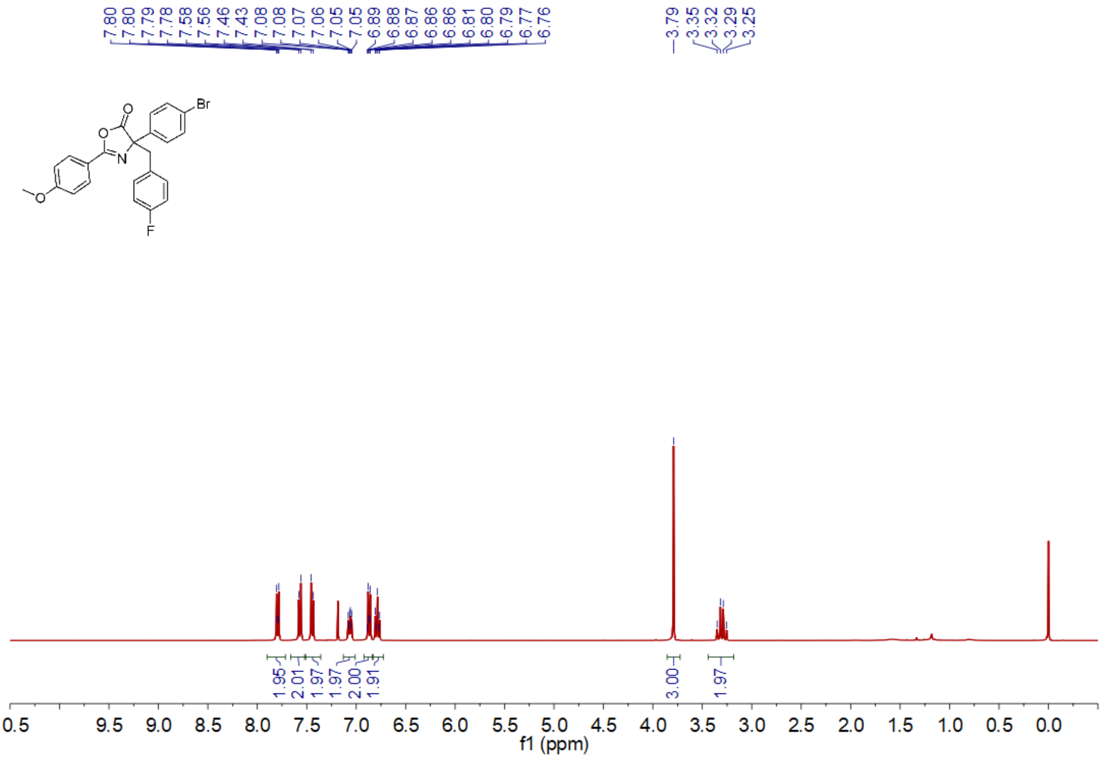

Supplementary Figure 78 ^{1}H NMR (400 MHz, $CDCl_3$, 25 °C) of compound **15**

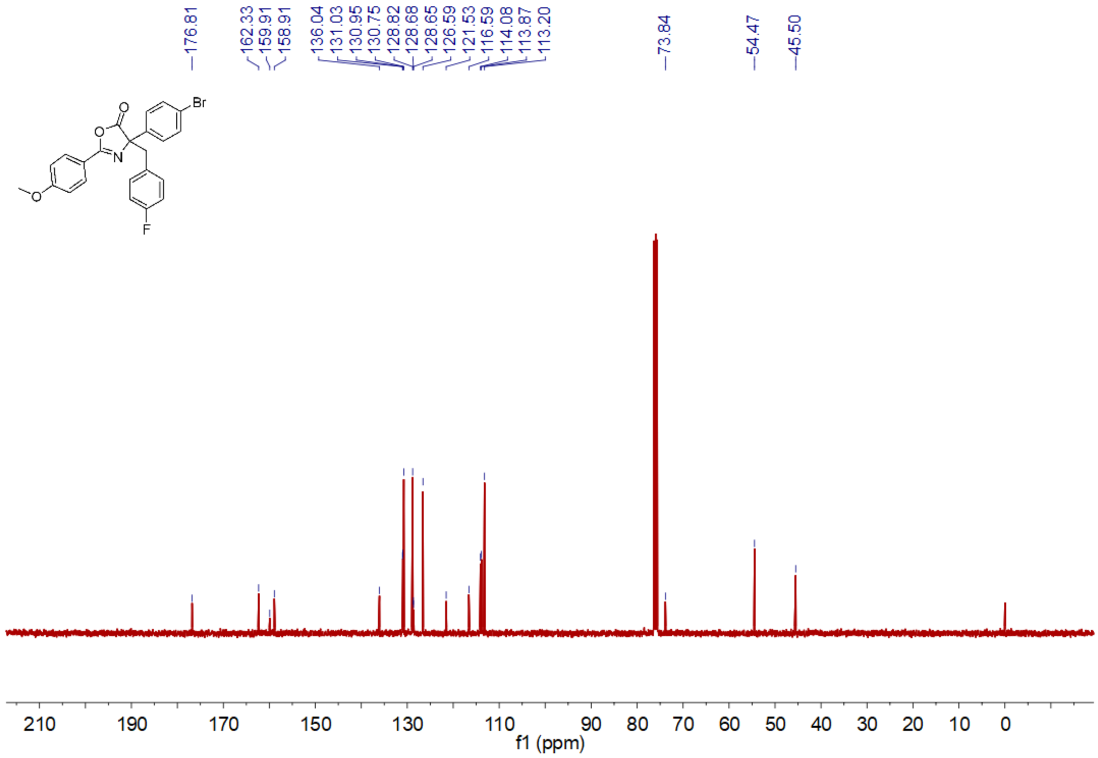

Supplementary Figure 79 ^{13}C NMR (101 MHz, $CDCl_3$, 25 °C) of compound **15**

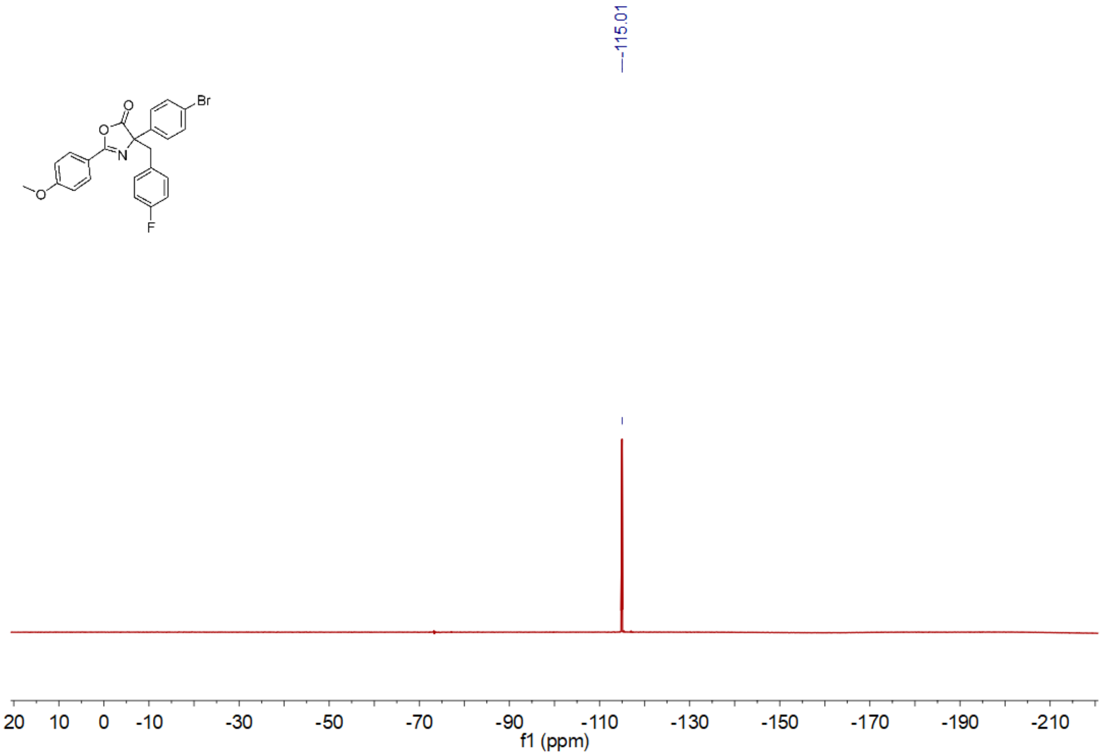

Supplementary Figure 80 ^{19}F NMR (377 MHz, $CDCl_3$, 25 °C) of compound **15**

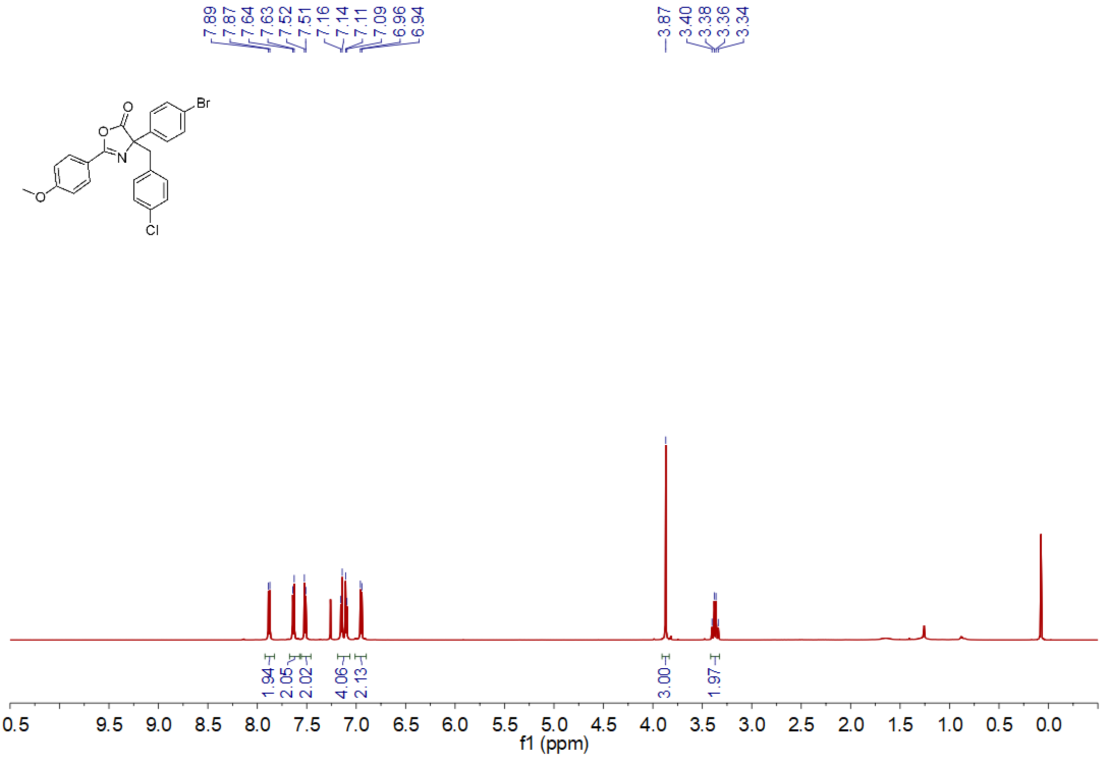

Supplementary Figure 81 ^{1}H NMR (600 MHz, $CDCl_3$, 25 °C) of compound **16**

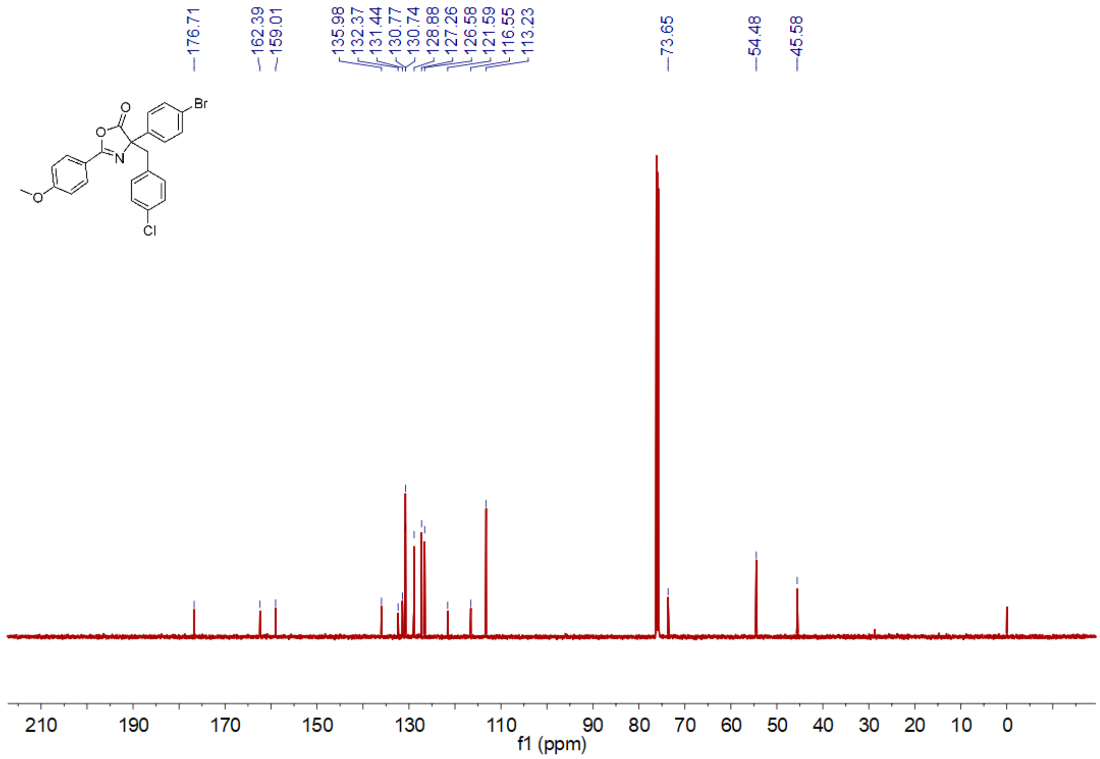

Supplementary Figure 82 ^{13}C NMR (151 MHz, $CDCl_3$, 25 °C) of compound **16**

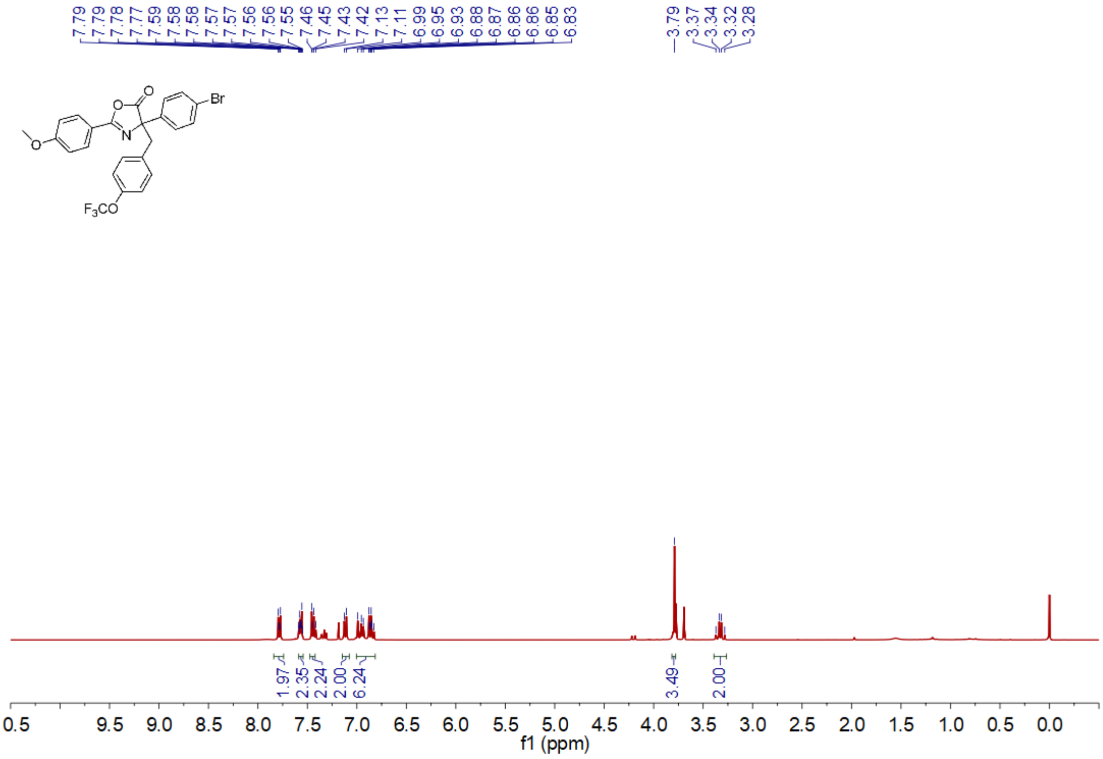

Supplementary Figure 83 ^{1}H NMR (400 MHz, $CDCl_3$, 25 °C) of compound **17**

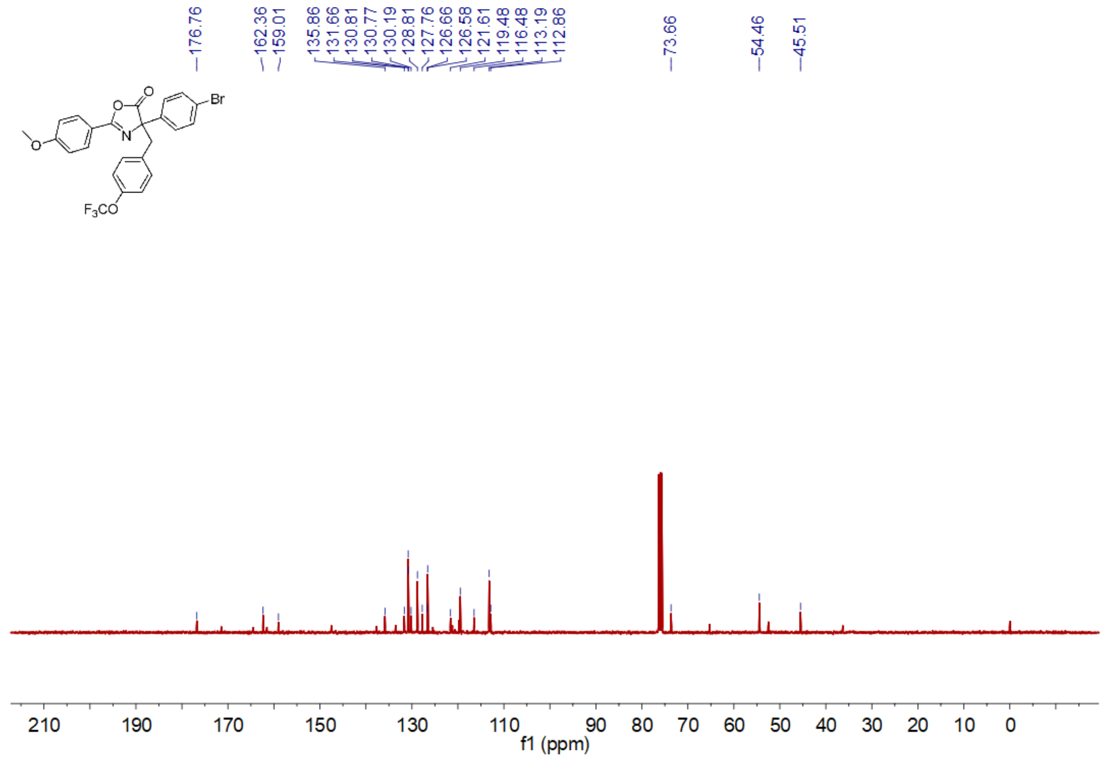

Supplementary Figure 84 ^{13}C NMR (101 MHz, $CDCl_3$, 25 °C) of compound **17**

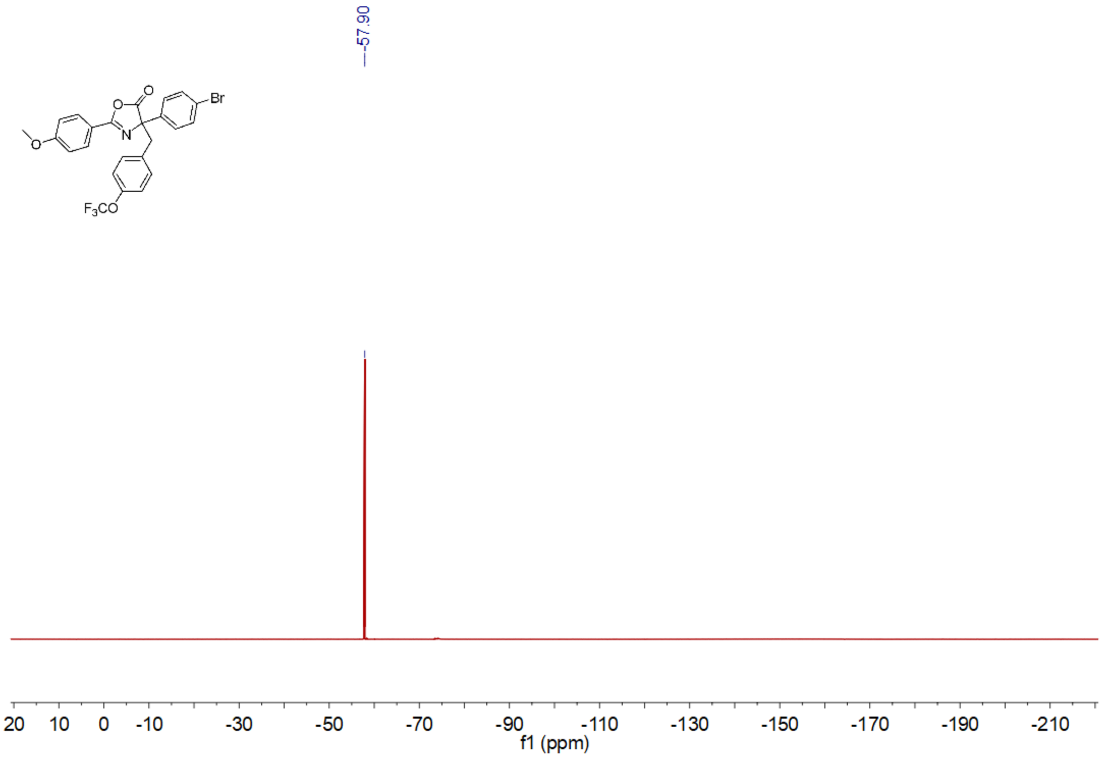

Supplementary Figure 85 ^{19}F NMR (377 MHz, $CDCl_3$, 25 °C) of compound **17**

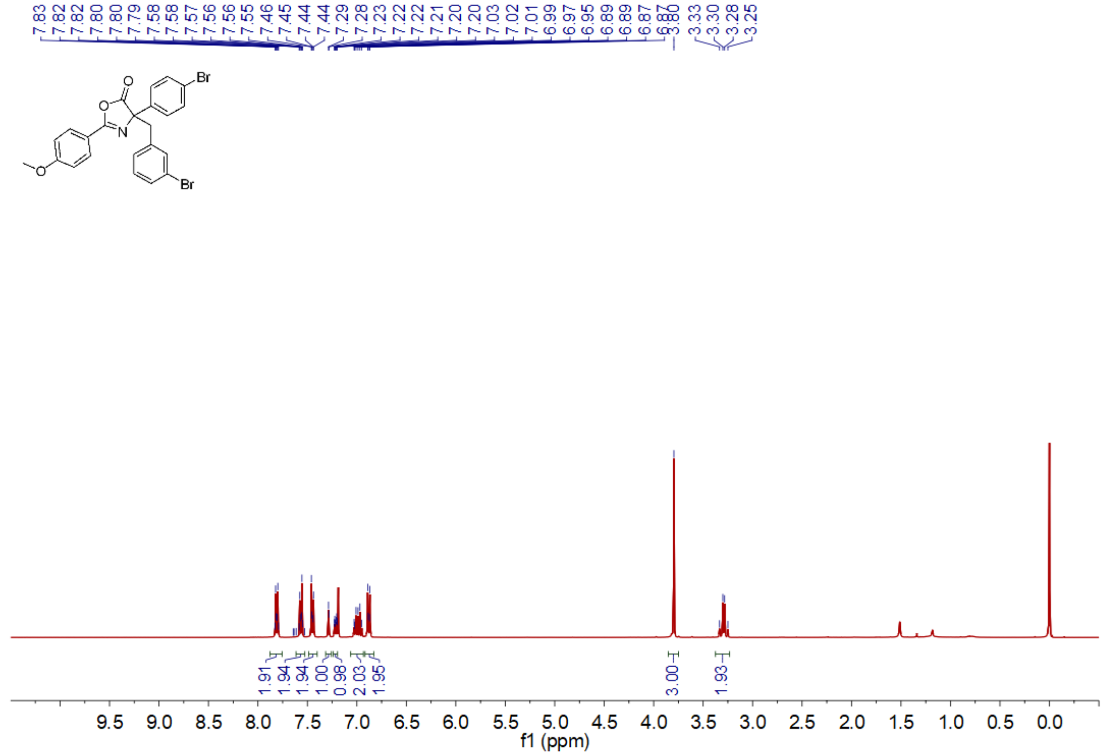

Supplementary Figure 86 ^{1}H NMR (400 MHz, $CDCl_3$, 25 °C) of compound **18**

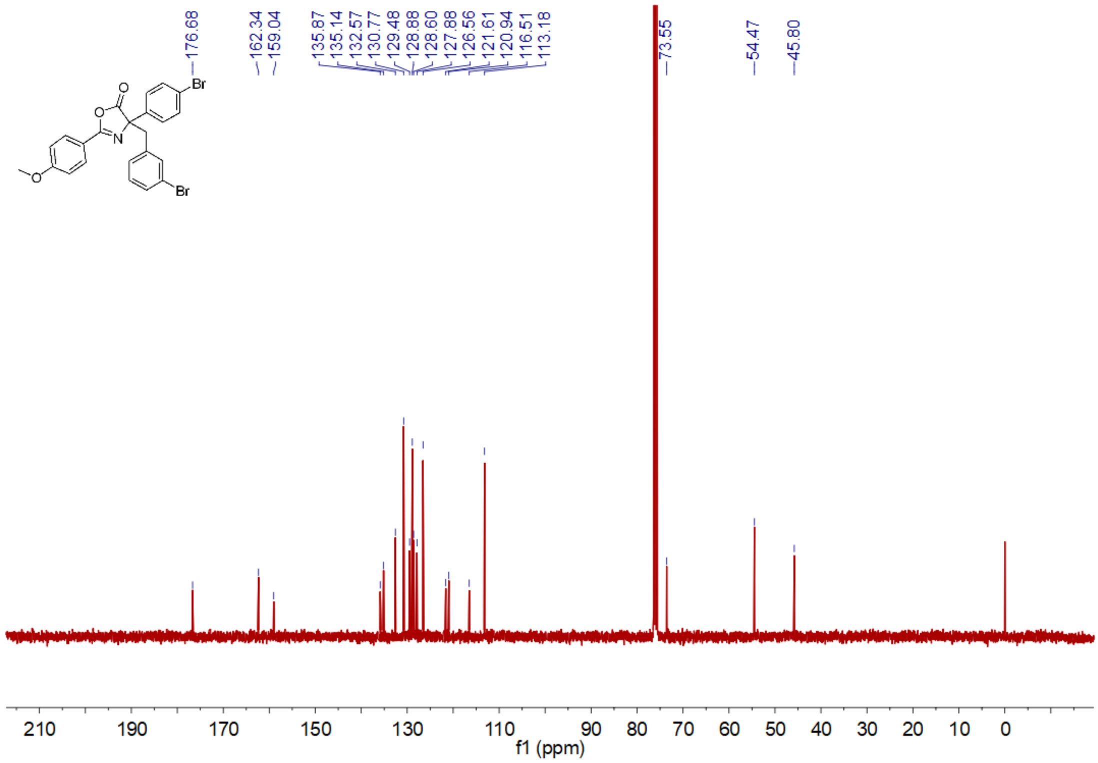

Supplementary Figure 87 ^{13}C NMR (101 MHz, $CDCl_3$, 25 °C) of compound **18**

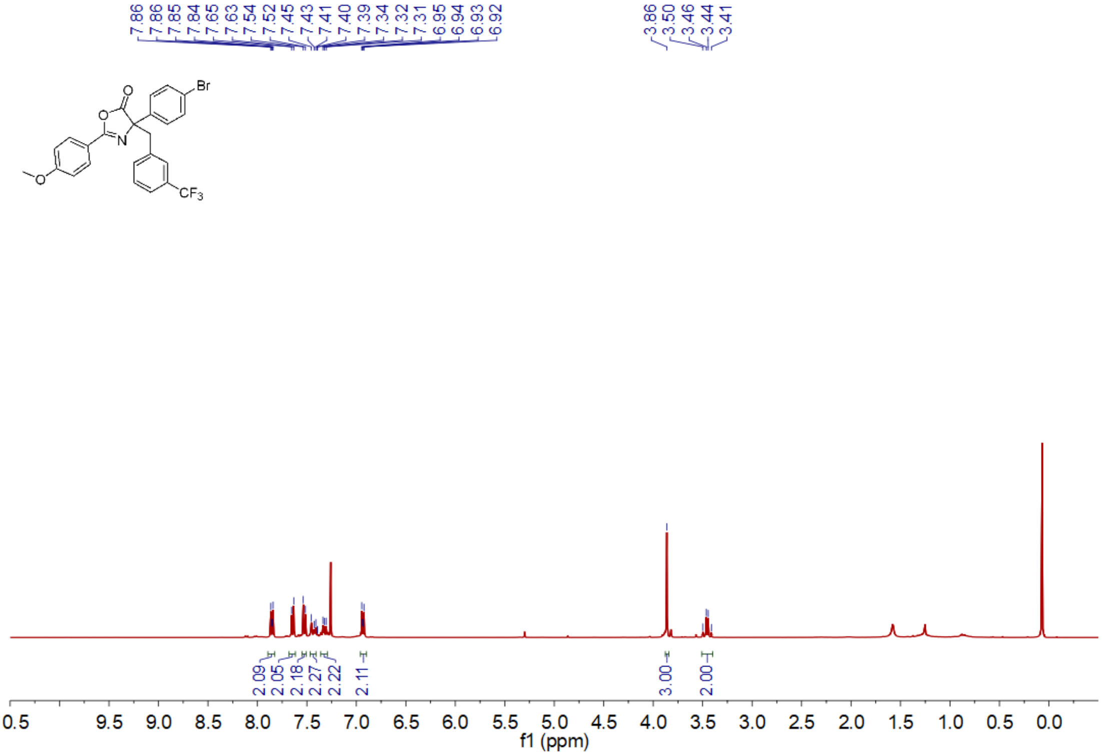

Supplementary Figure 88 ^{1}H NMR (400 MHz, $CDCl_3$, 25 °C) of compound **19**

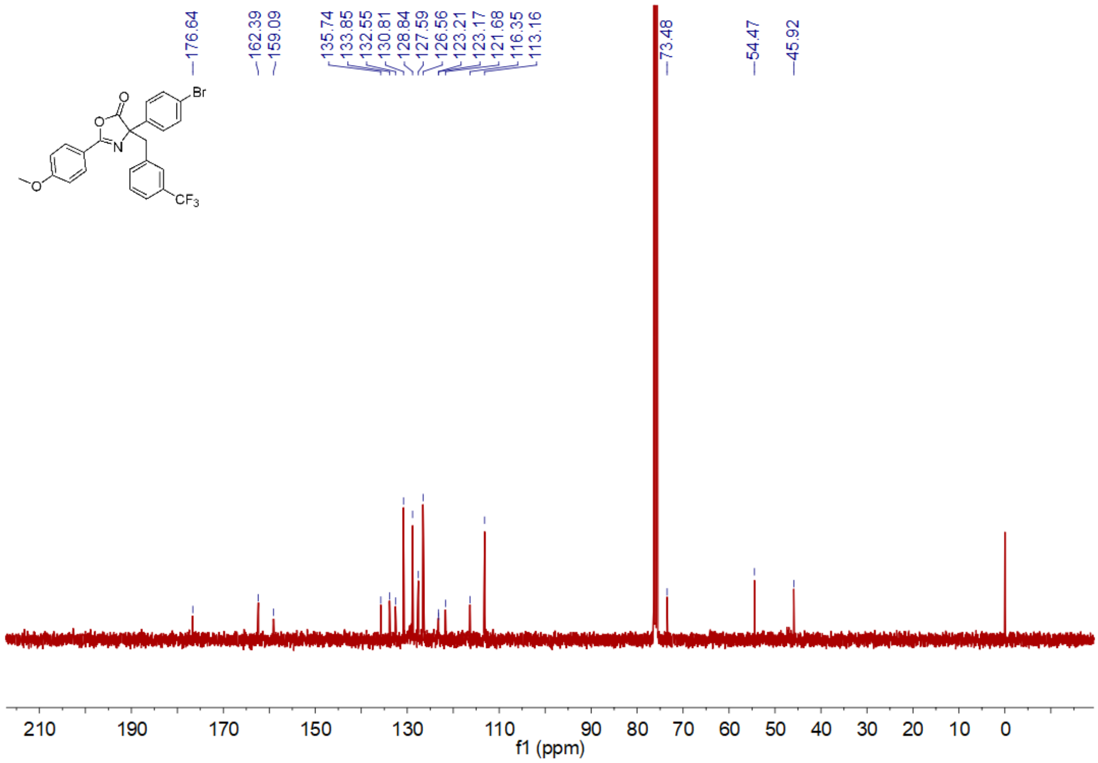

Supplementary Figure 89 ^{13}C NMR (101 MHz, $CDCl_3$, 25 °C) of compound **19**

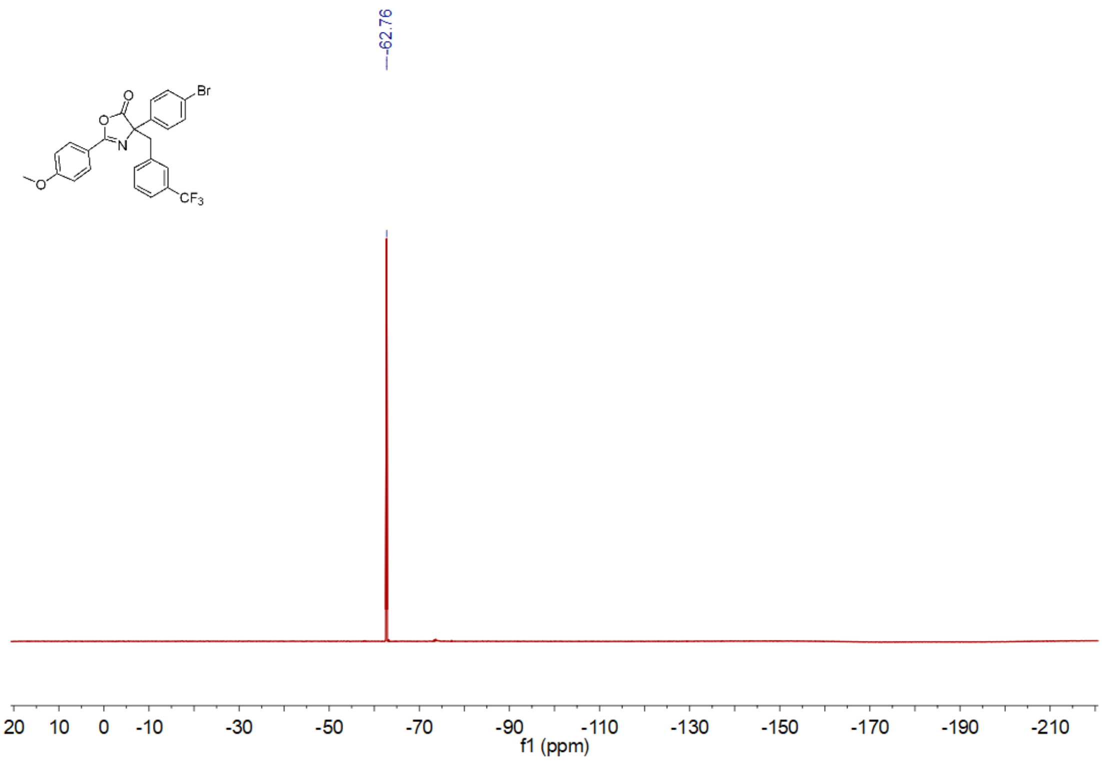

Supplementary Figure 90 ^{19}F NMR (377 MHz, $CDCl_3$, 25 °C) of compound **19**

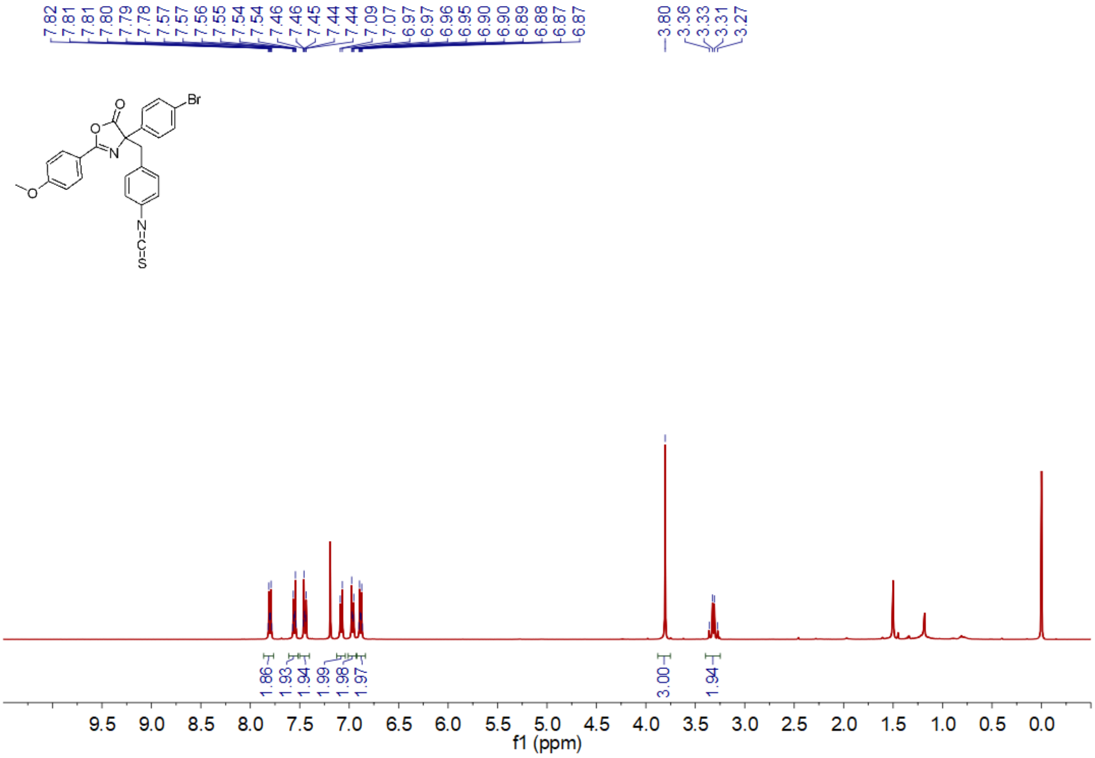

Supplementary Figure 91 ^{1}H NMR (400 MHz, $CDCl_3$, 25 °C) of compound **20**

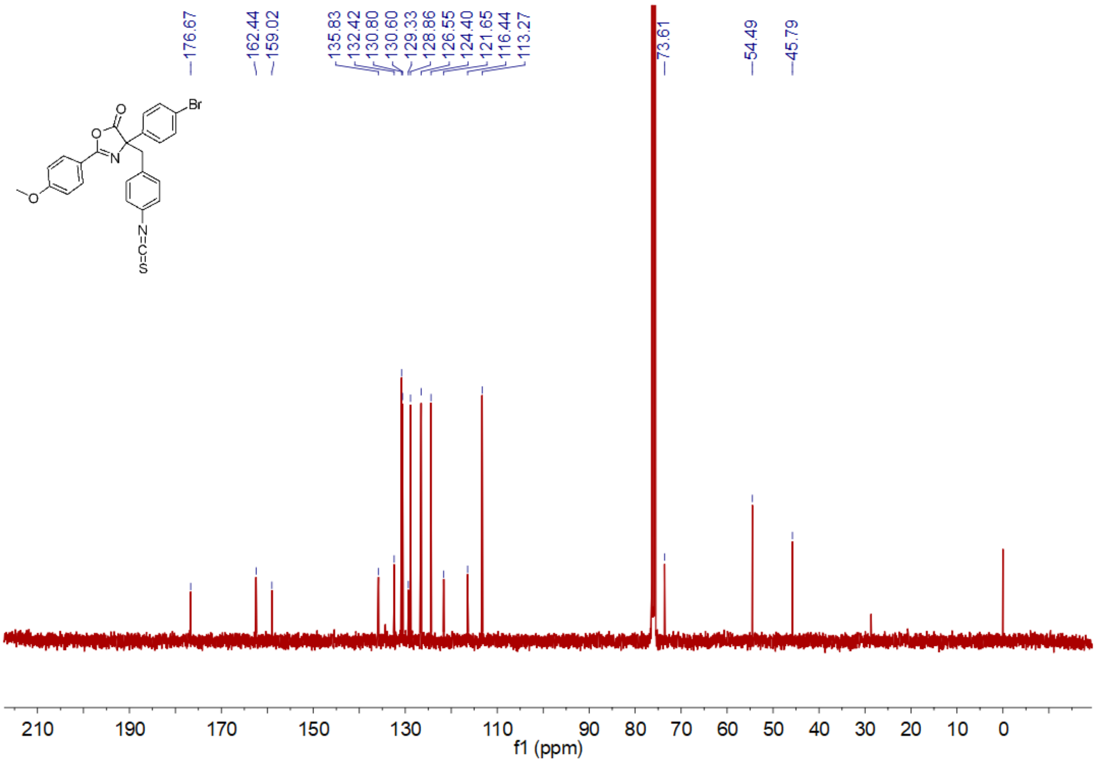

Supplementary Figure 92 ^{13}C NMR (101 MHz, $CDCl_3$, 25 °C) of compound **20**

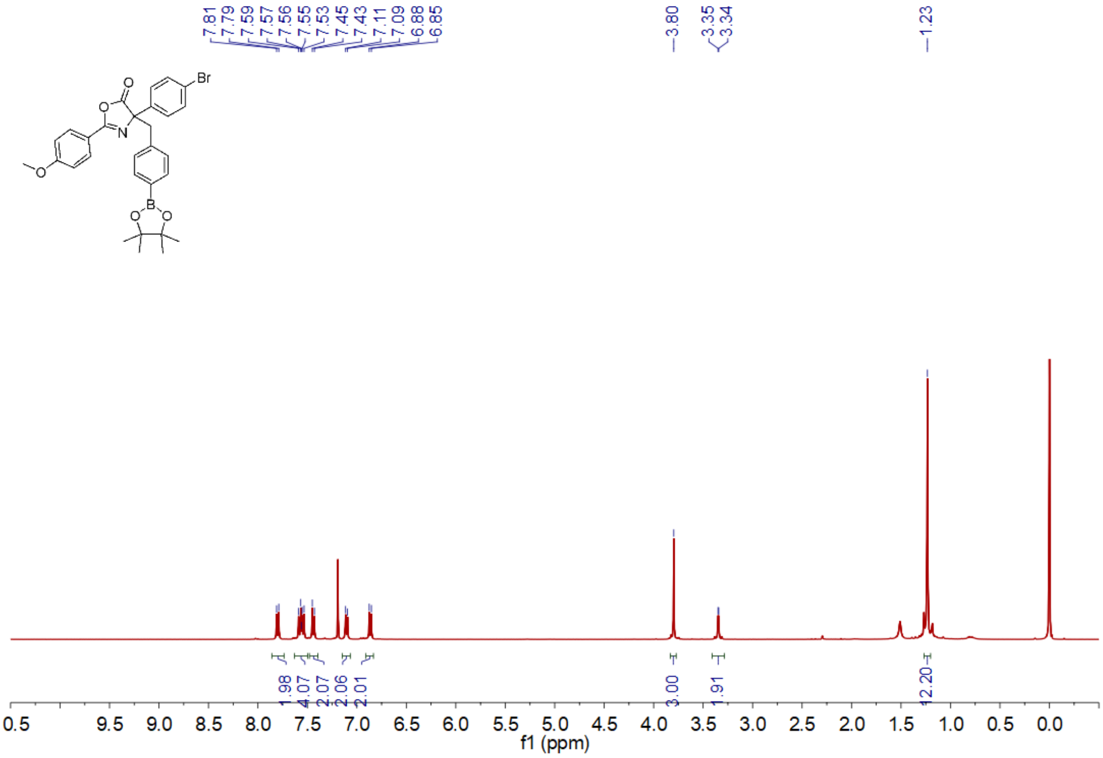

Supplementary Figure 93 ^{1}H NMR (400 MHz, $CDCl_3$, 25 °C) of compound **21**

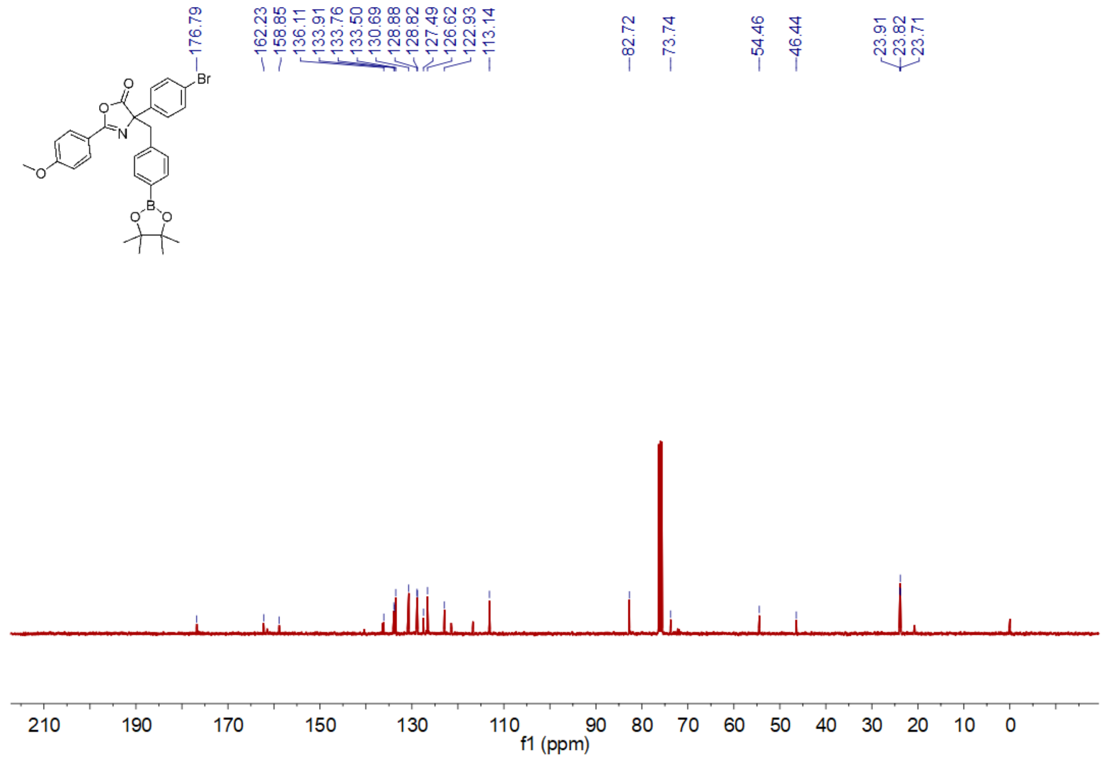

Supplementary Figure 94 ^{13}C NMR (101 MHz, $CDCl_3$, 25 °C) of compound **21**

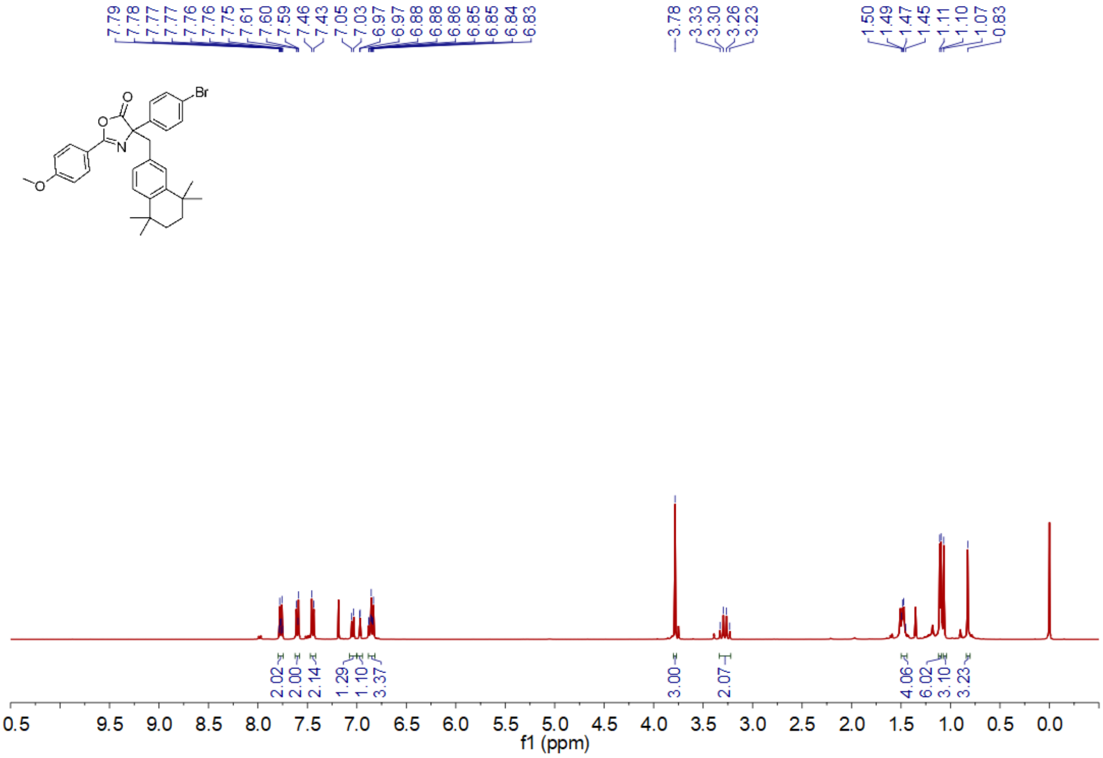

Supplementary Figure 95 ^{1}H NMR (400 MHz, $CDCl_3$, 25 °C) of compound **22**

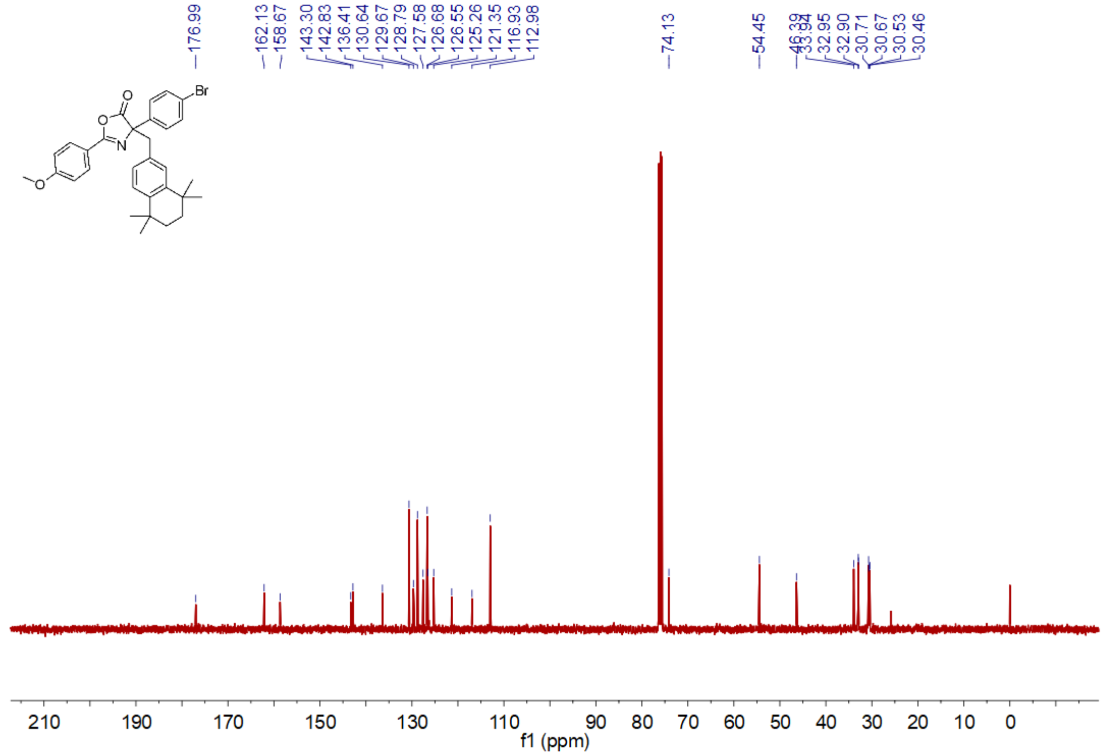

Supplementary Figure 96 ^{13}C NMR (101 MHz, $CDCl_3$, 25 °C) of compound **22**

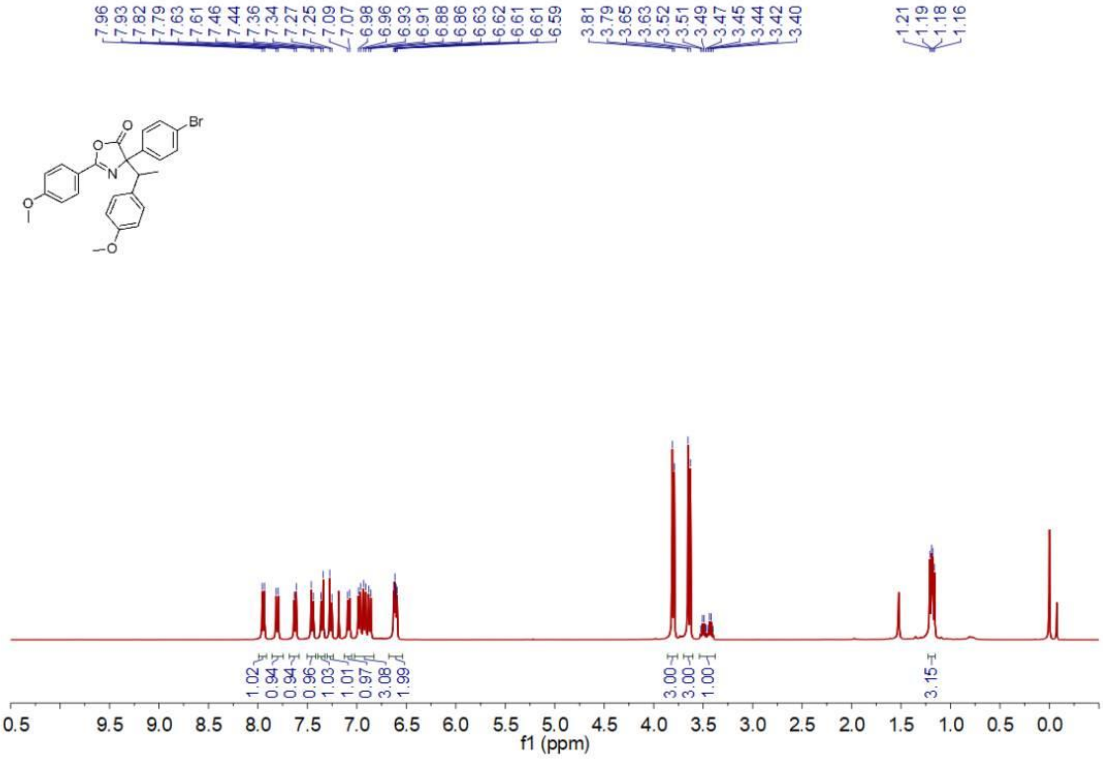

Supplementary Figure 97 ^{1}H NMR (400 MHz, $CDCl_3$, 25 °C) of compound **23**

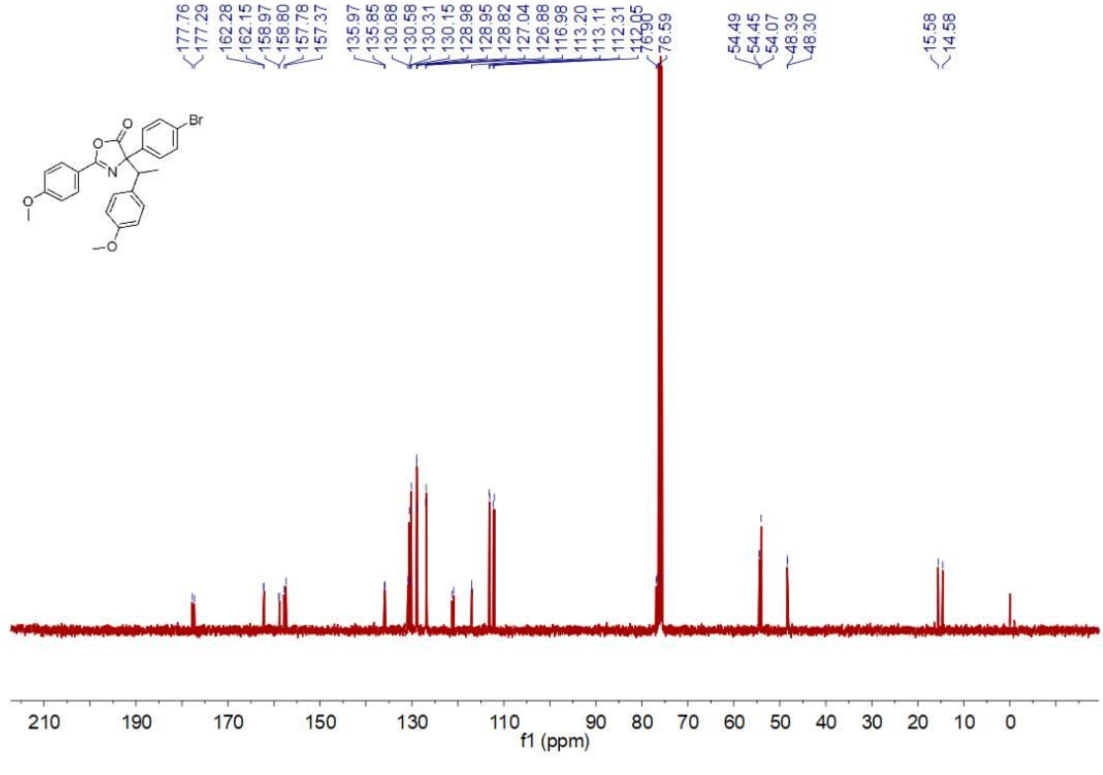

Supplementary Figure 98 ^{13}C NMR (101 MHz, $CDCl_3$, 25 °C) of compound **23**

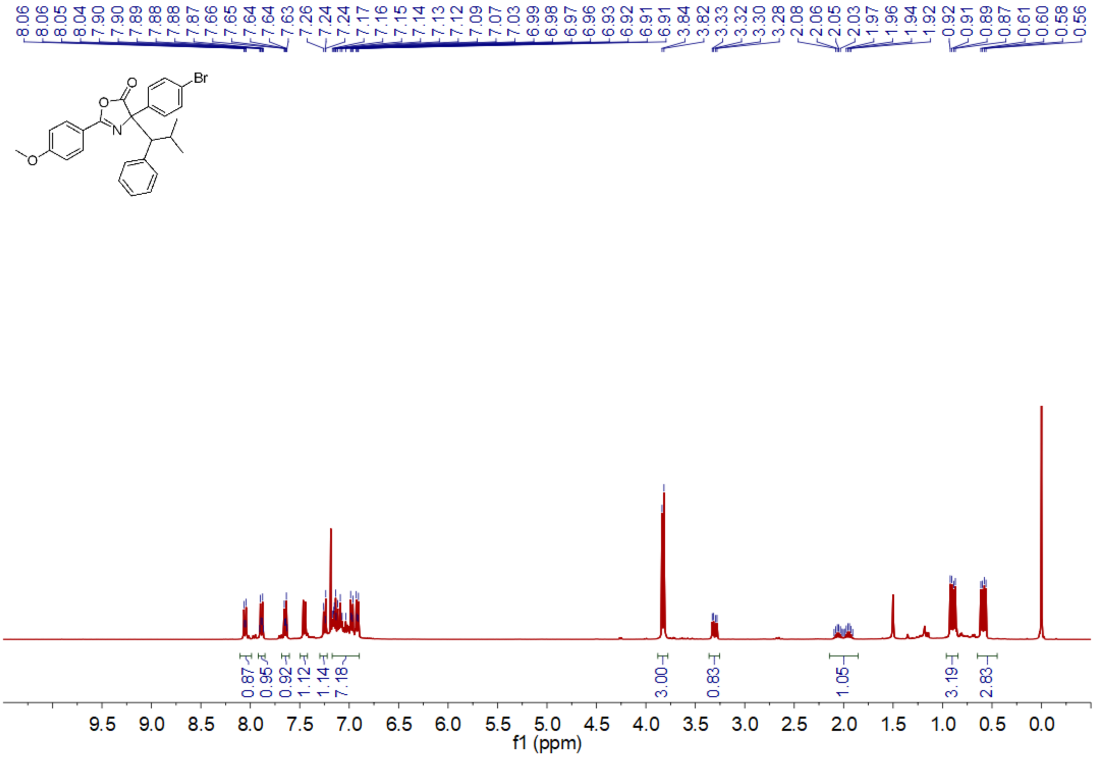

Supplementary Figure 99 ^{1}H NMR (400 MHz, $CDCl_3$, 25 °C) of compound **24**

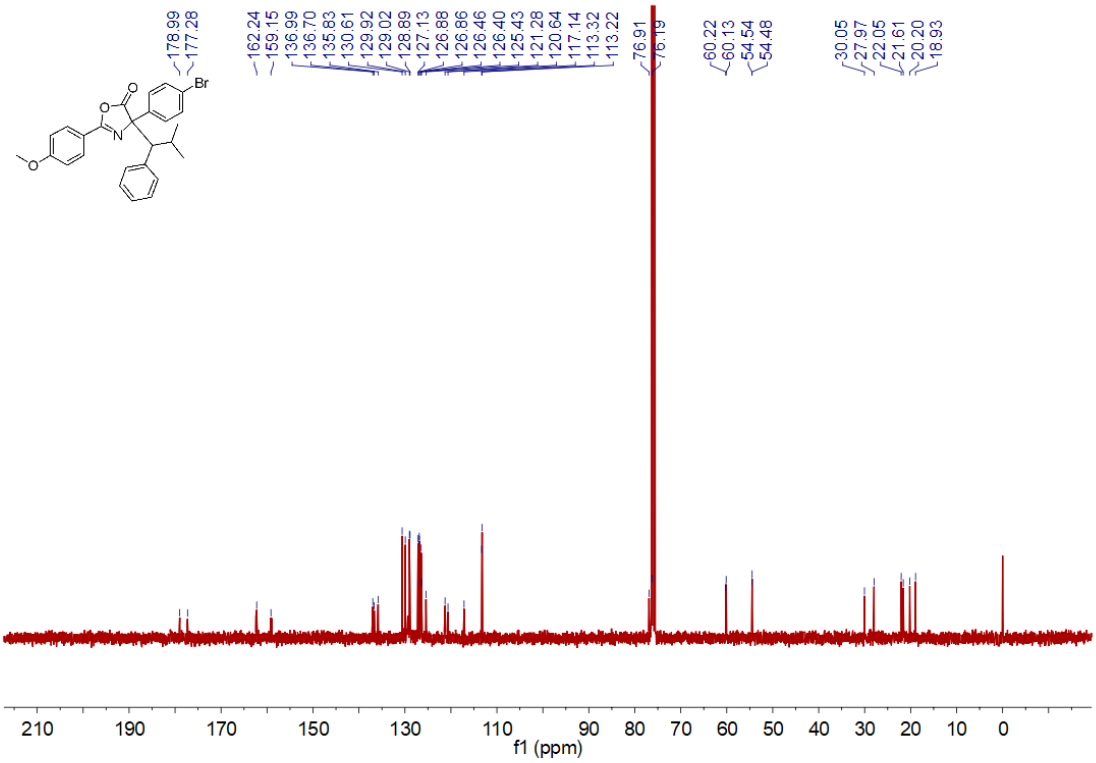

Supplementary Figure 100 ^{13}C NMR (101 MHz, $CDCl_3$, 25 °C) of compound **24**

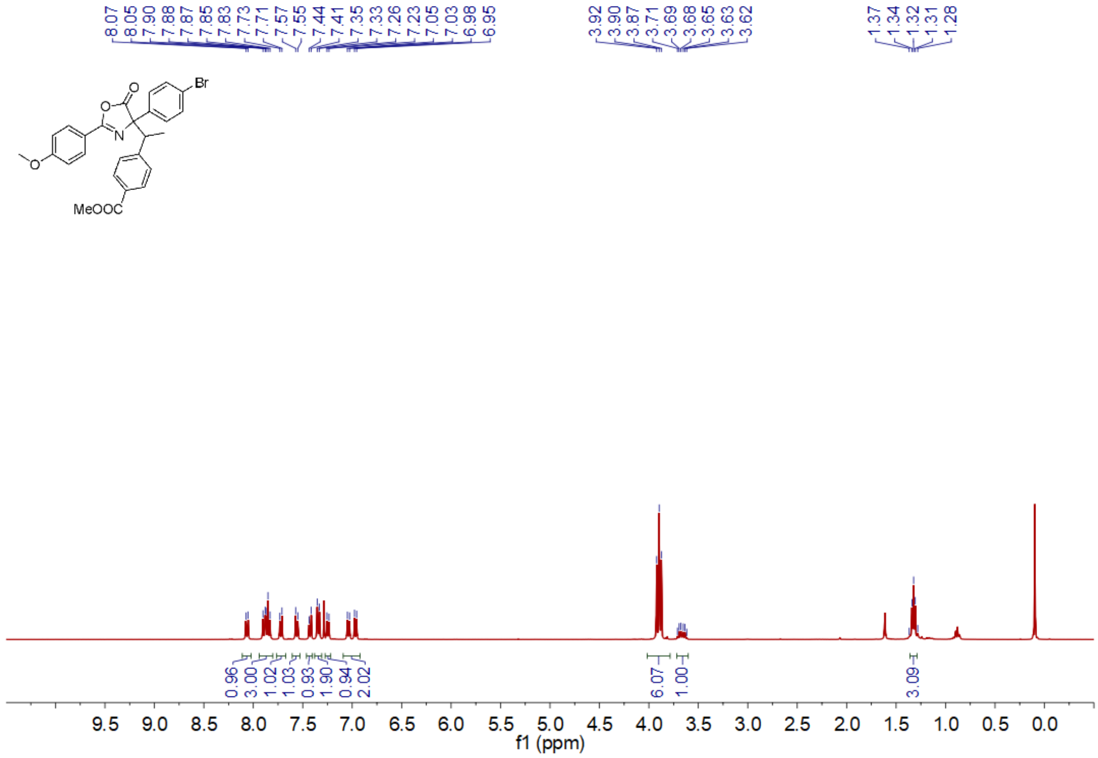

Supplementary Figure 101 ^{1}H NMR (400 MHz, $CDCl_3$, 25 °C) of compound **25**

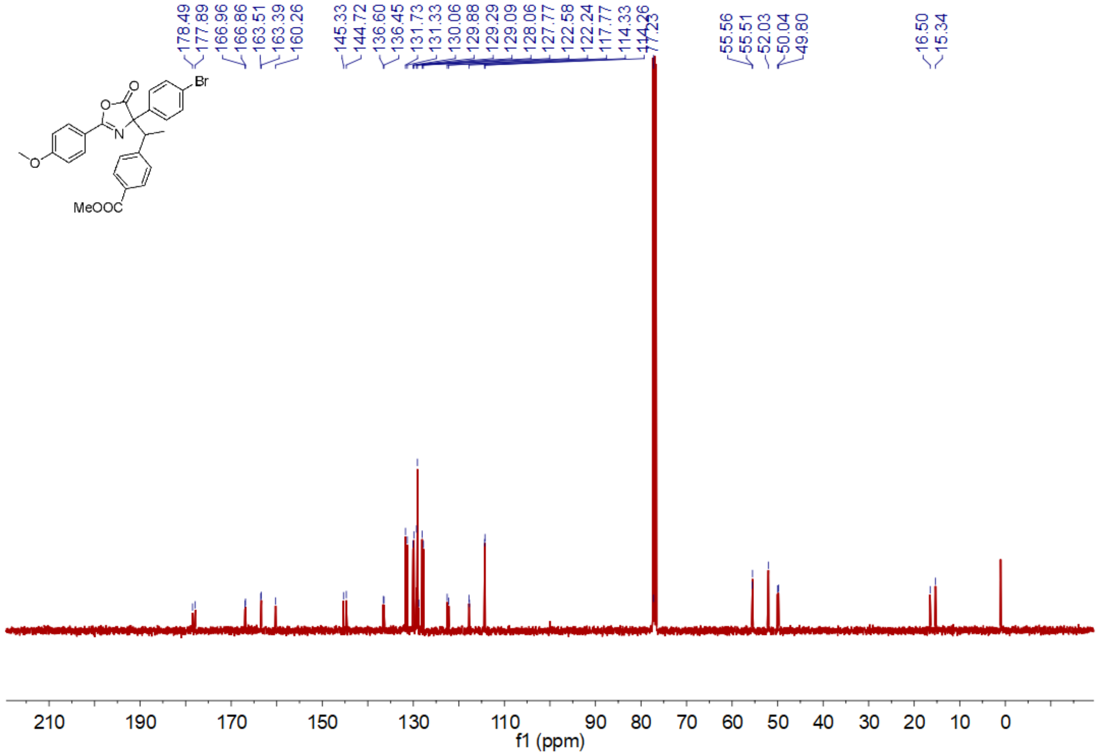

Supplementary Figure 102 ^{13}C NMR (101 MHz, $CDCl_3$, 25 °C) of compound **25**

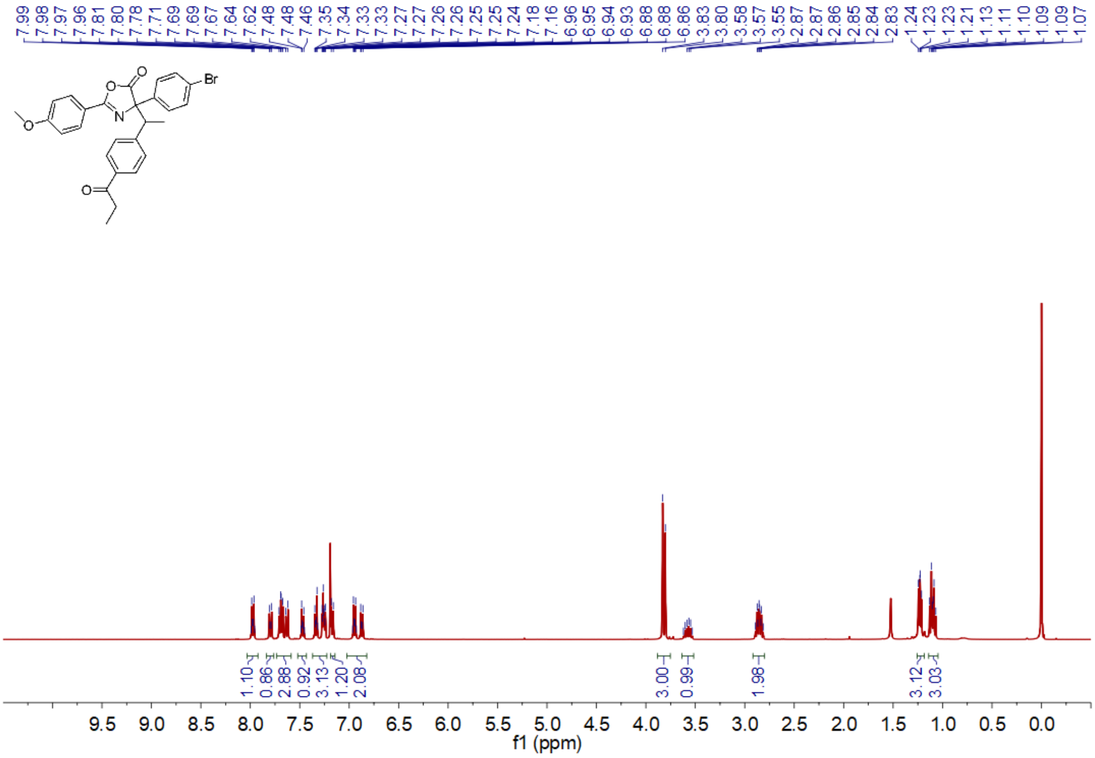

Supplementary Figure 103 ^{1}H NMR (400 MHz, $CDCl_3$, 25 °C) of compound **26**

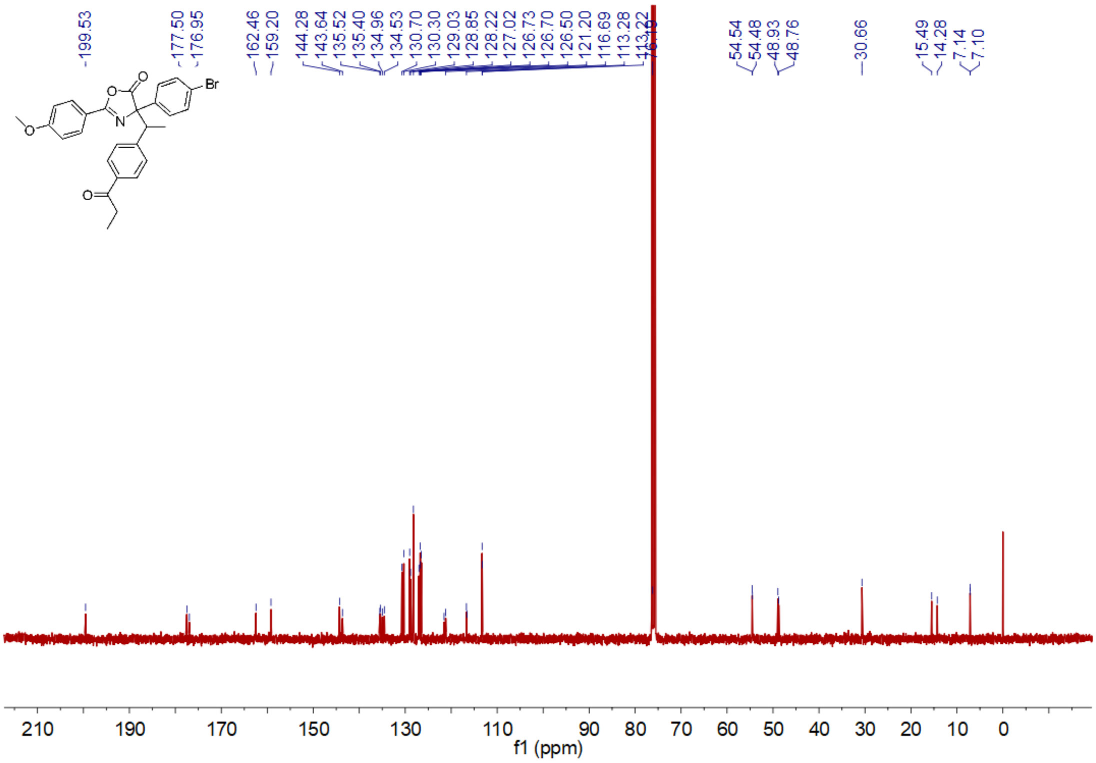

Supplementary Figure 104 ^{13}C NMR (101 MHz, $CDCl_3$, 25 °C) of compound **26**

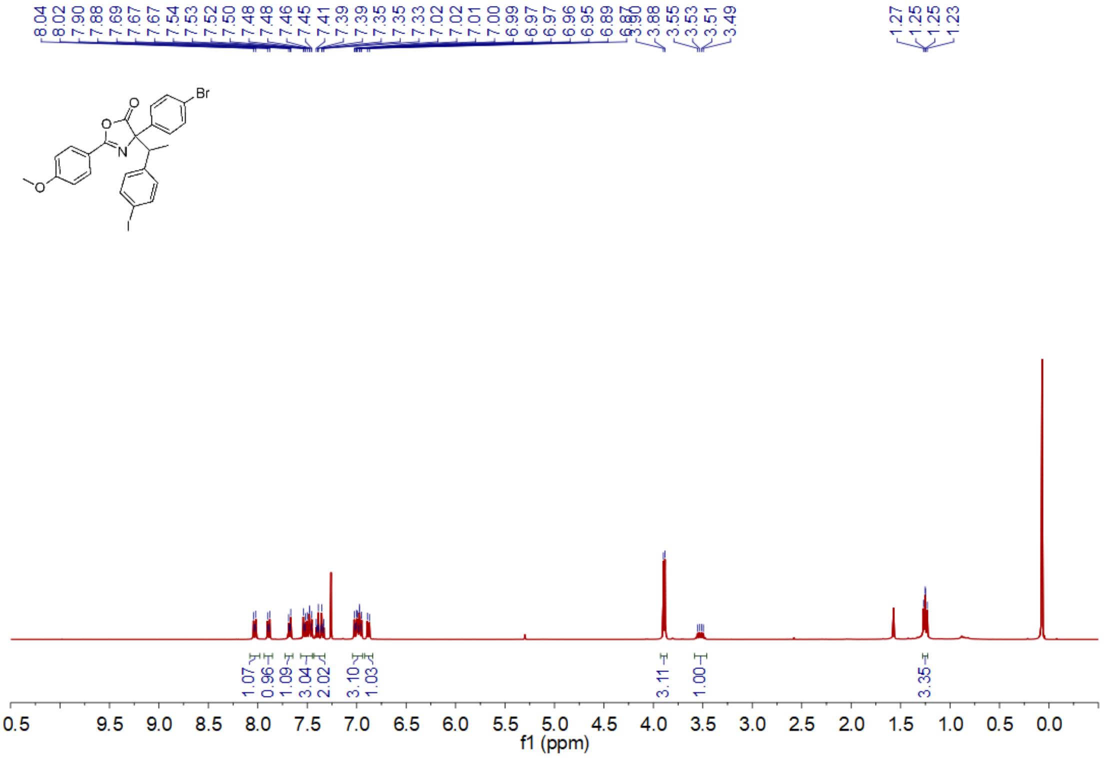

Supplementary Figure 105 ^{1}H NMR (400 MHz, $CDCl_3$, 25 °C) of compound **27**

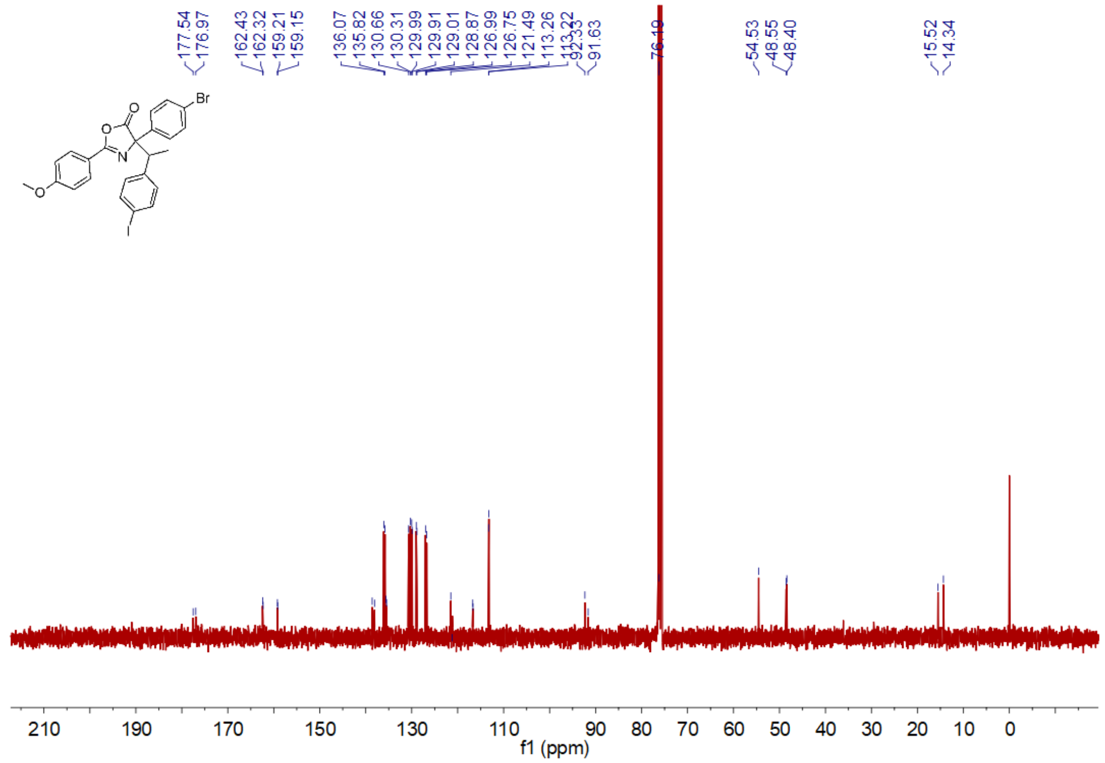

Supplementary Figure 106 ^{13}C NMR (101 MHz, $CDCl_3$, 25 °C) of compound **27**

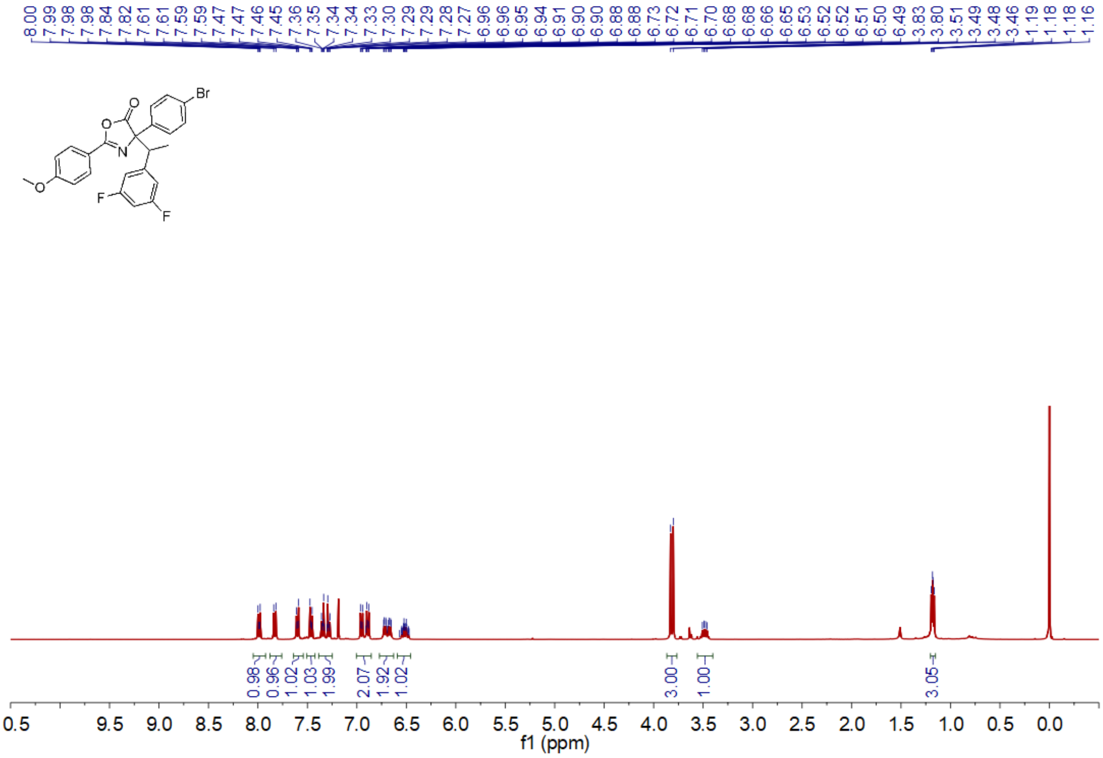

Supplementary Figure 107 ^{1}H NMR (400 MHz, $CDCl_3$, 25 °C) of compound **28**

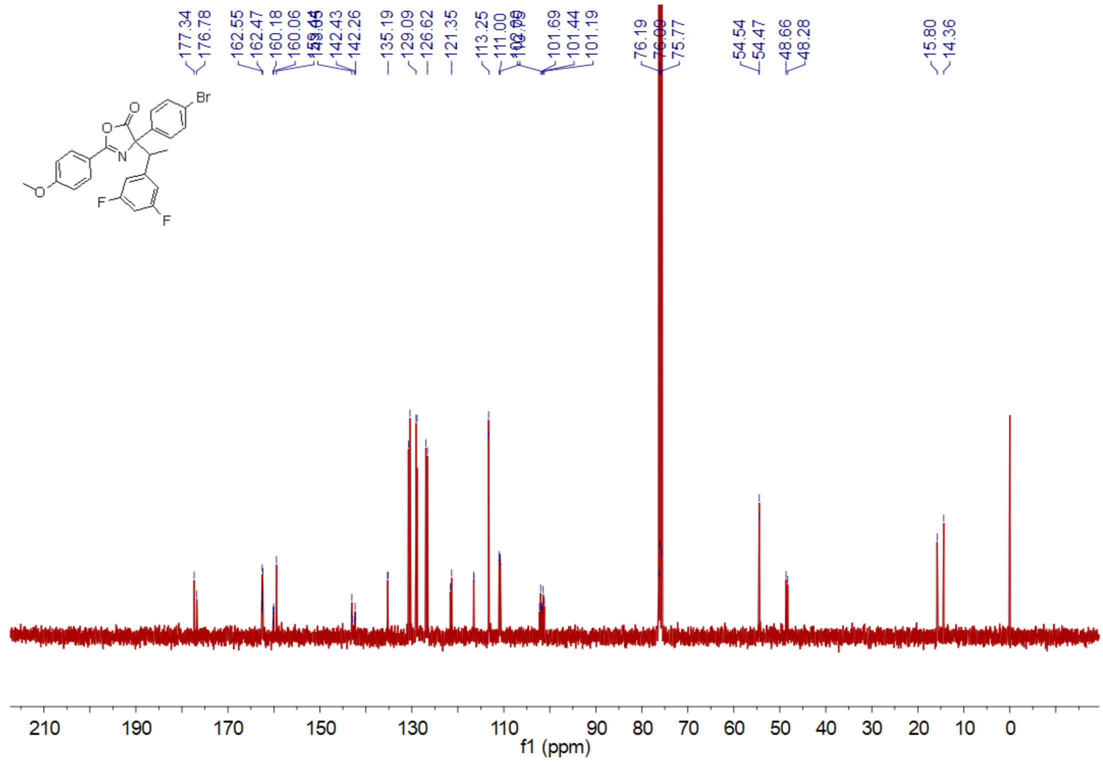

Supplementary Figure 108 ^{13}C NMR (101 MHz, $CDCl_3$, 25 °C) of compound **28**

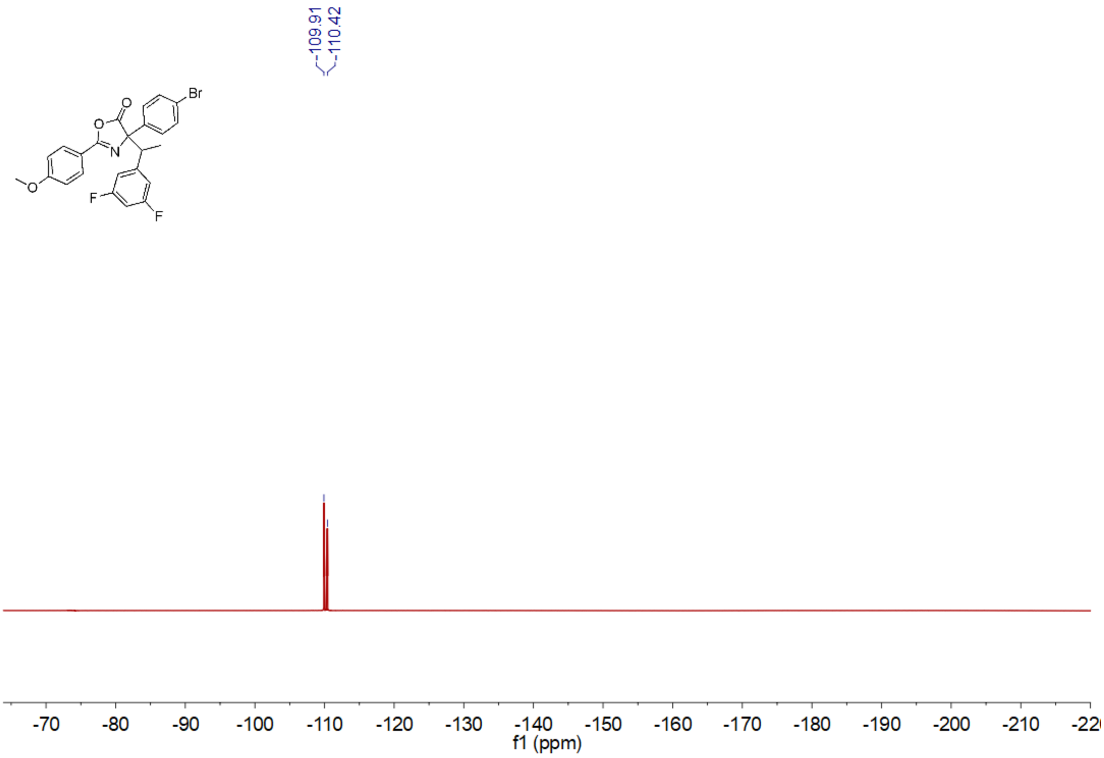

Supplementary Figure 109 ^{19}F NMR (377 MHz, $CDCl_3$, 25 °C) of compound **28**

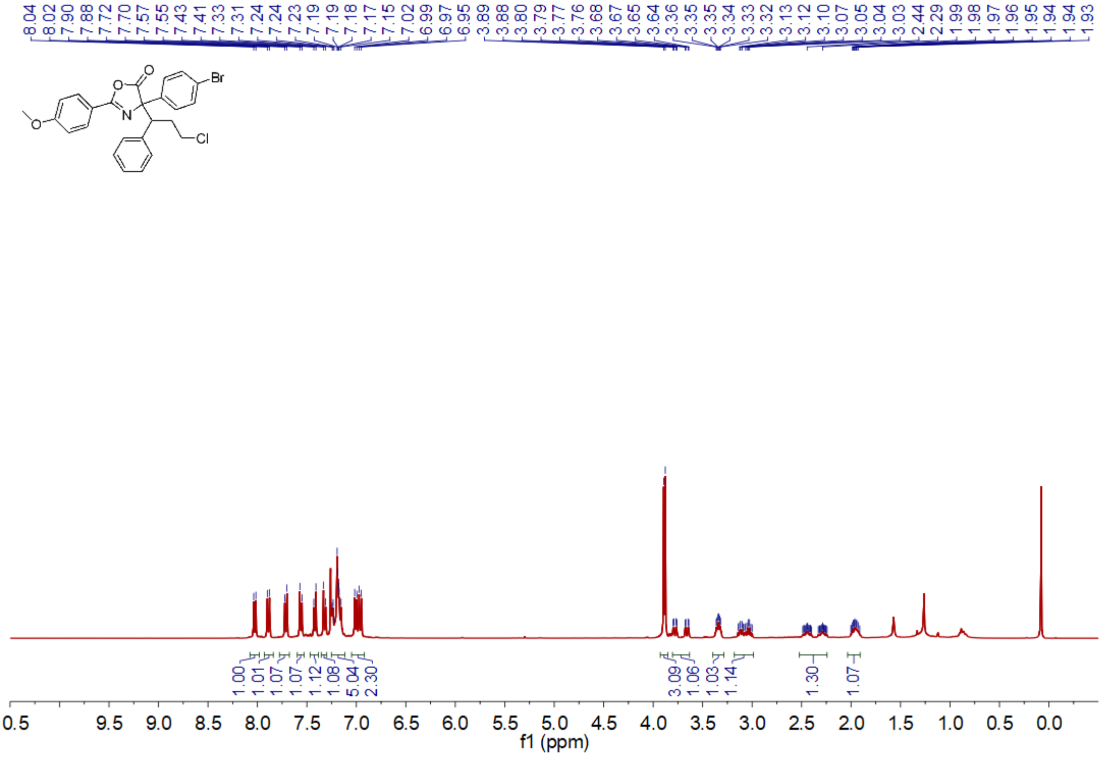

Supplementary Figure 110 ^{1}H NMR (400 MHz, $CDCl_3$, 25 °C) of compound **29**

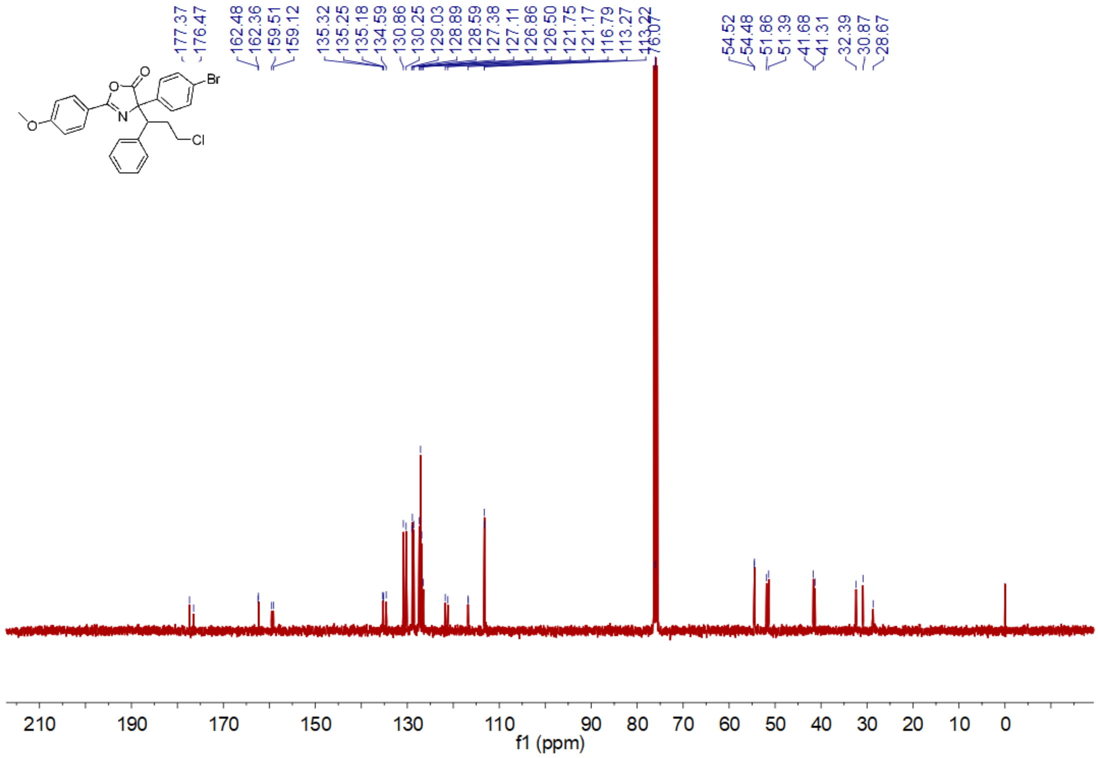

Supplementary Figure 111 ^{13}C NMR (101 MHz, $CDCl_3$, 25 °C) of compound **29**

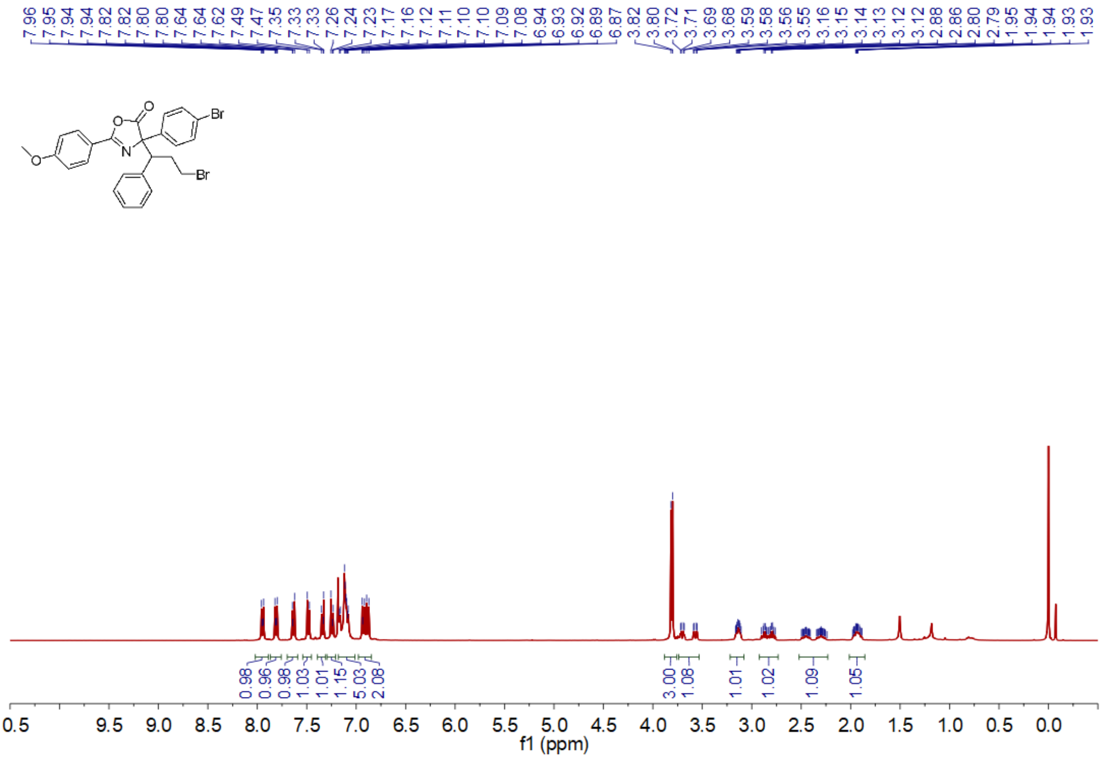

Supplementary Figure 112 ^{1}H NMR (400 MHz, $CDCl_3$, 25 °C) of compound **30**

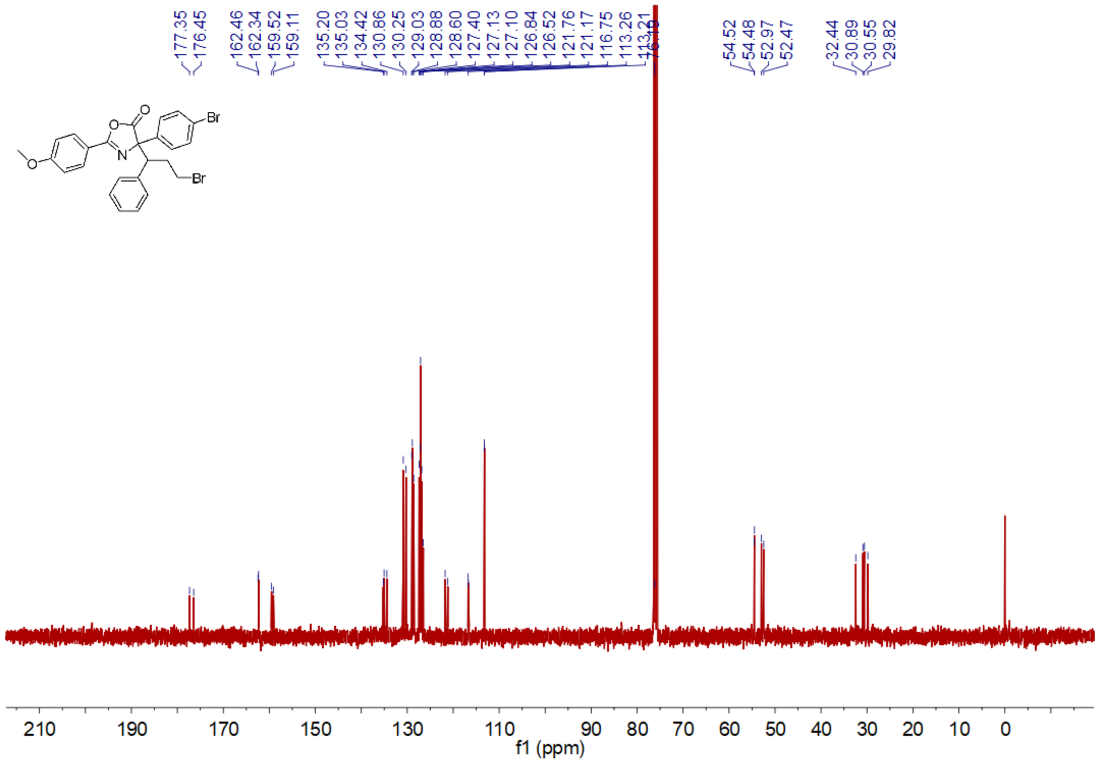

Supplementary Figure 113 ^{13}C NMR (101 MHz, $CDCl_3$, 25 °C) of compound **30**

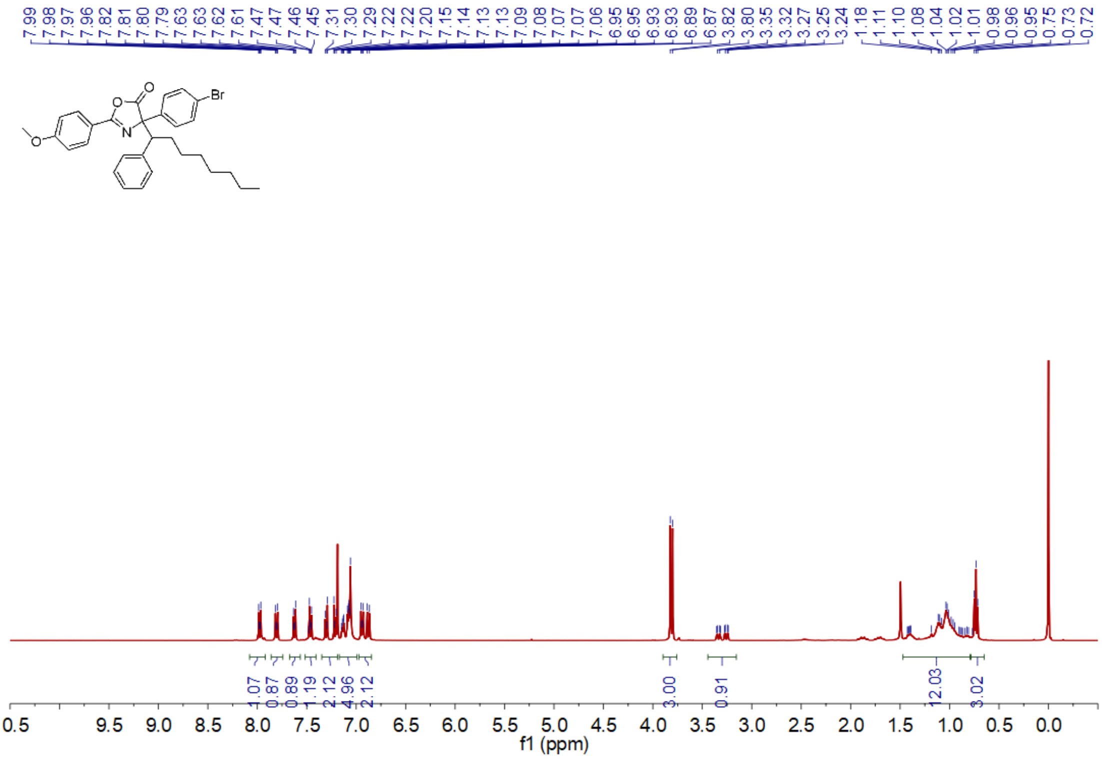

Supplementary Figure 114 ^{1}H NMR (400 MHz, $CDCl_3$, 25 °C) of compound **31**

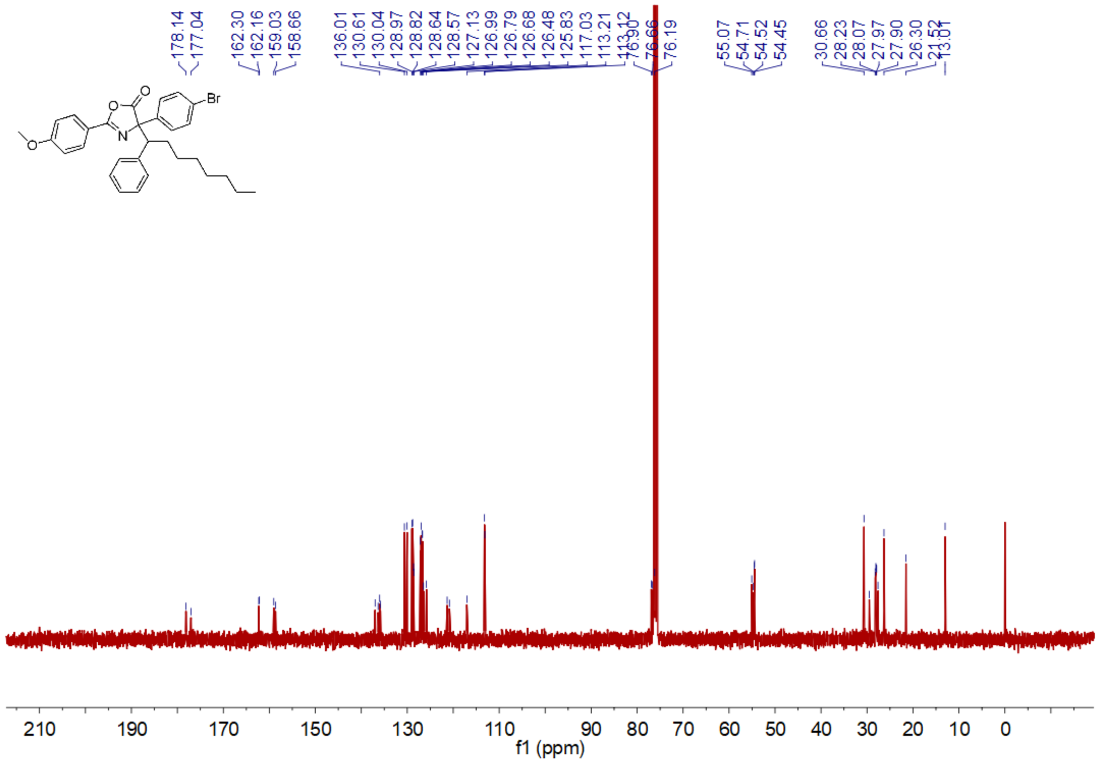

Supplementary Figure 115 ^{13}C NMR (101 MHz, $CDCl_3$, 25 °C) of compound **31**

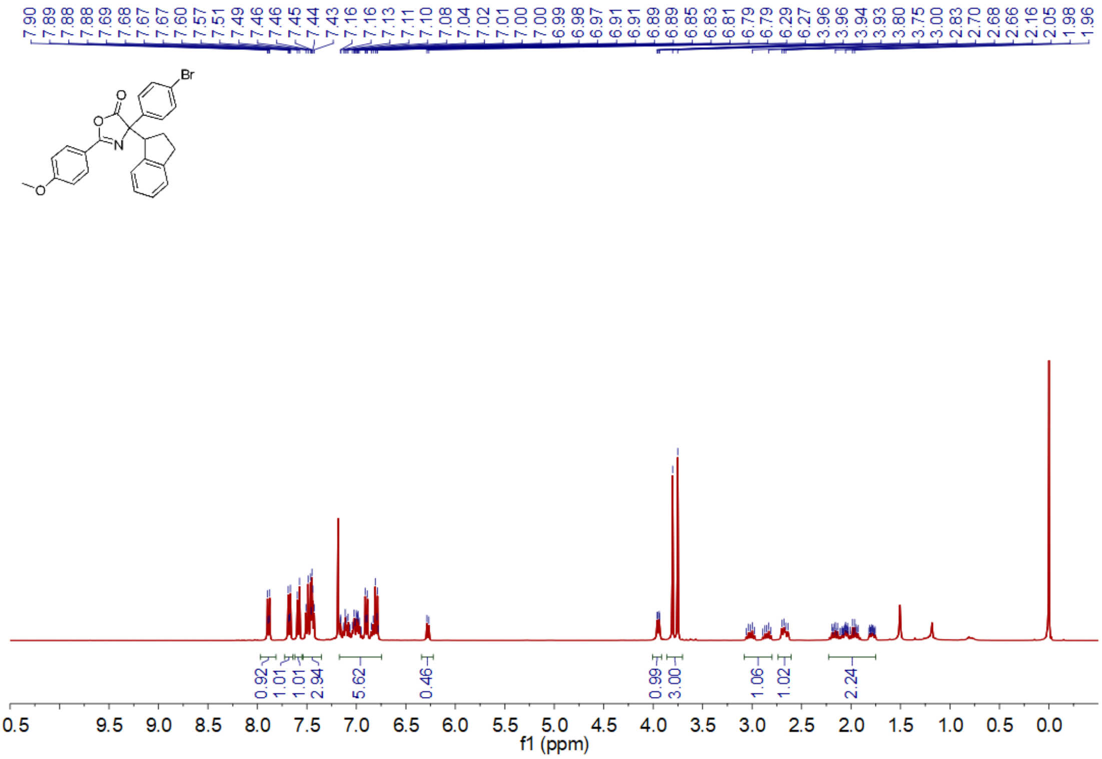

Supplementary Figure 116 ^{1}H NMR (400 MHz, $CDCl_3$, 25 °C) of compound **32**

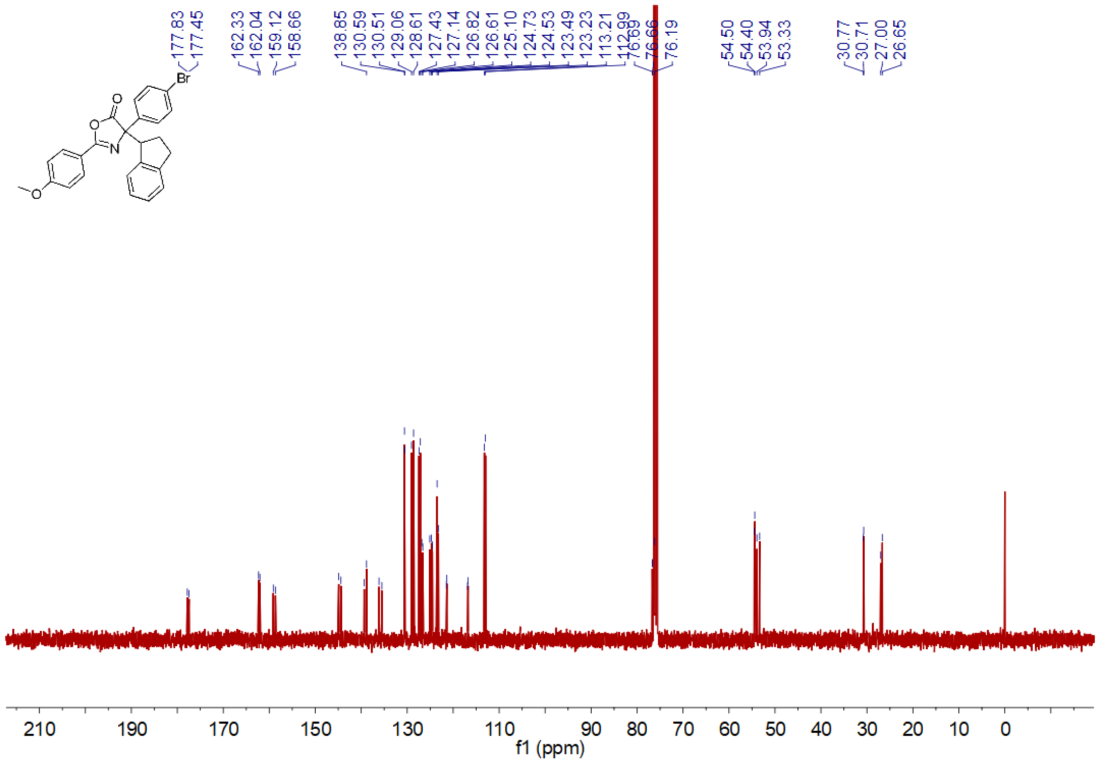

Supplementary Figure 117 ^{13}C NMR (101 MHz, $CDCl_3$, 25 °C) of compound **32**

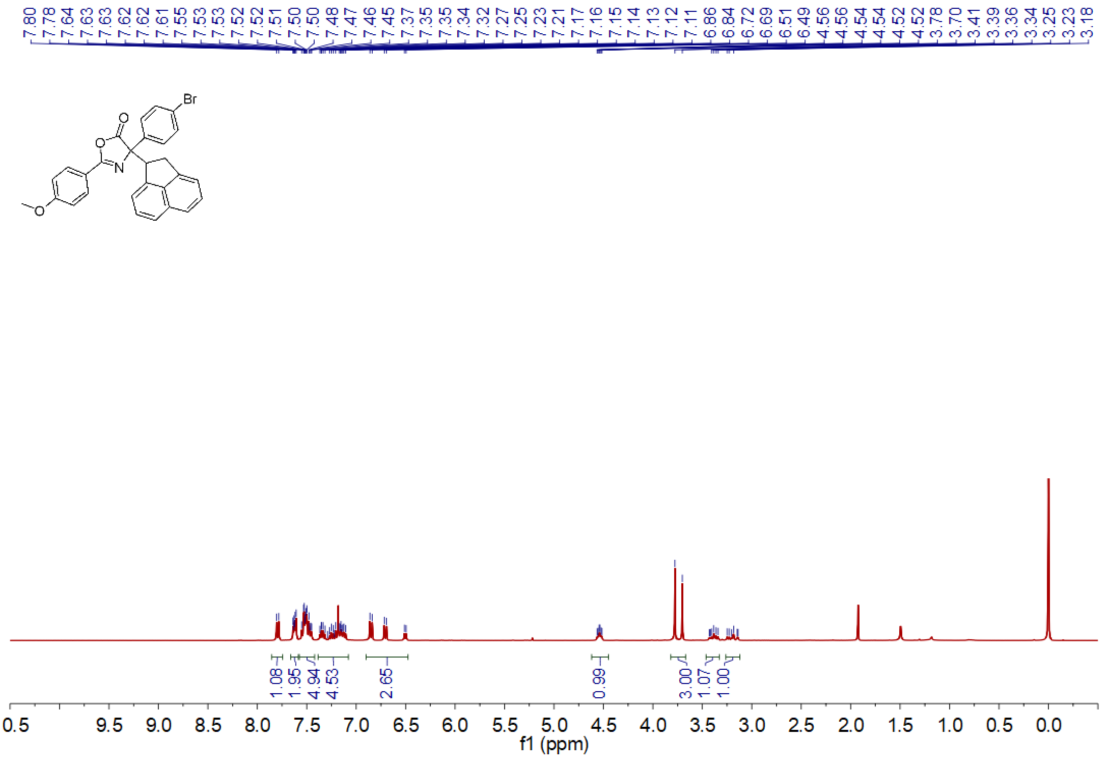

Supplementary Figure 118 ^{1}H NMR (400 MHz, $CDCl_3$, 25 °C) of compound **33**

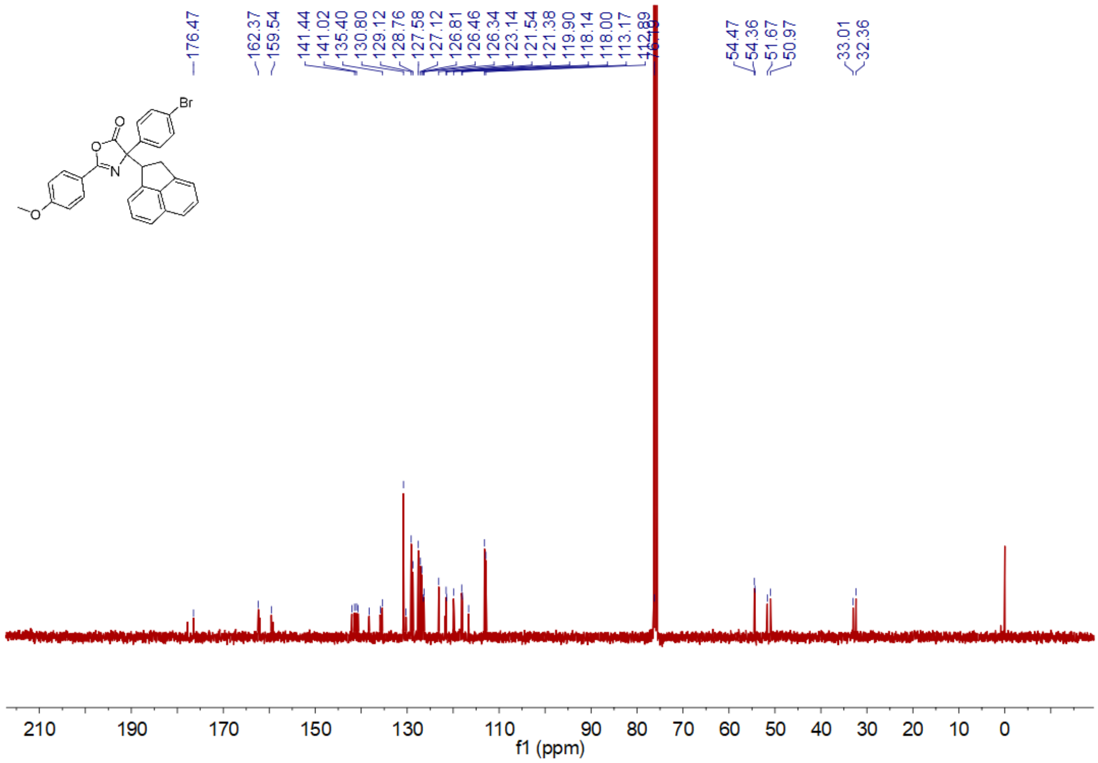

Supplementary Figure 119 ^{13}C NMR (101 MHz, $CDCl_3$, 25 °C) of compound **33**

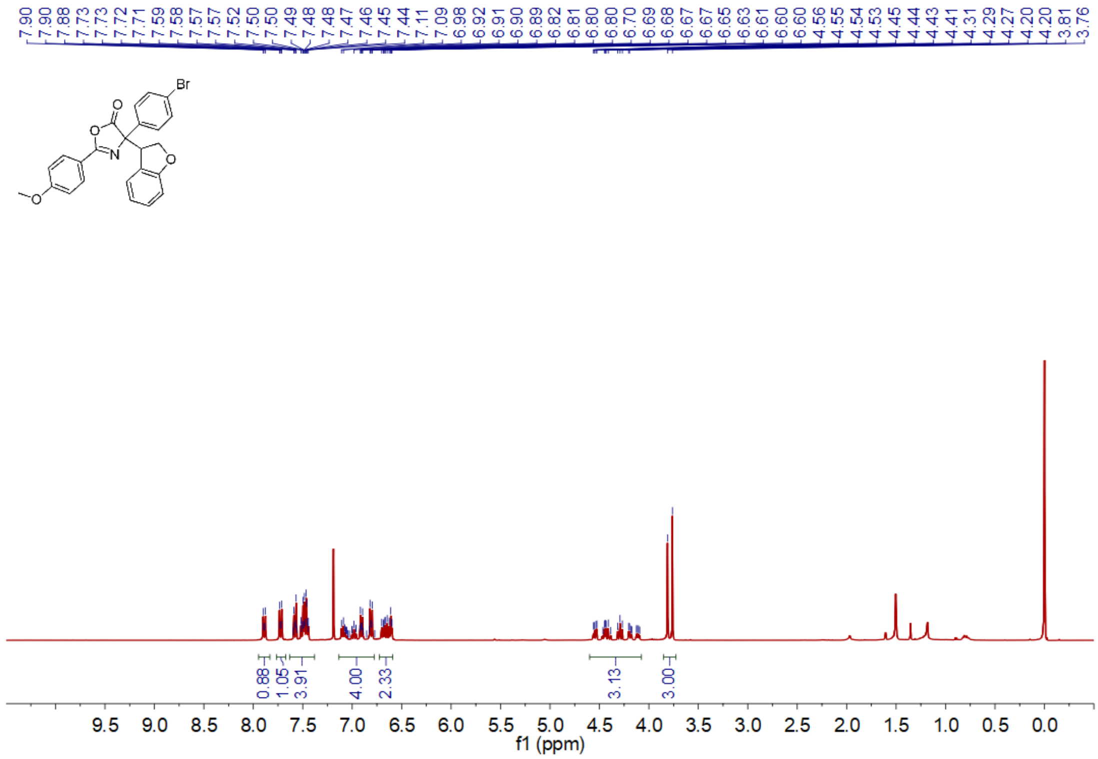

Supplementary Figure 120 ^{1}H NMR (400 MHz, $CDCl_3$, 25 °C) of compound **34**

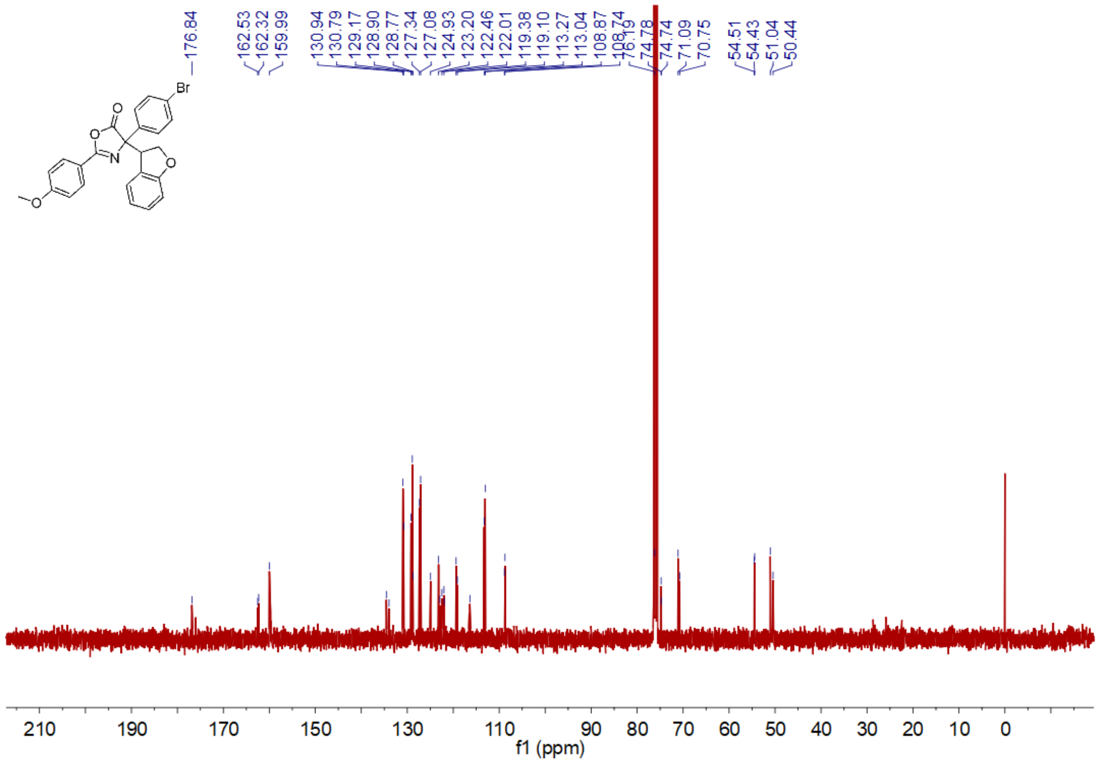

Supplementary Figure 121 ^{13}C NMR (101 MHz, $CDCl_3$, 25 °C) of compound **34**

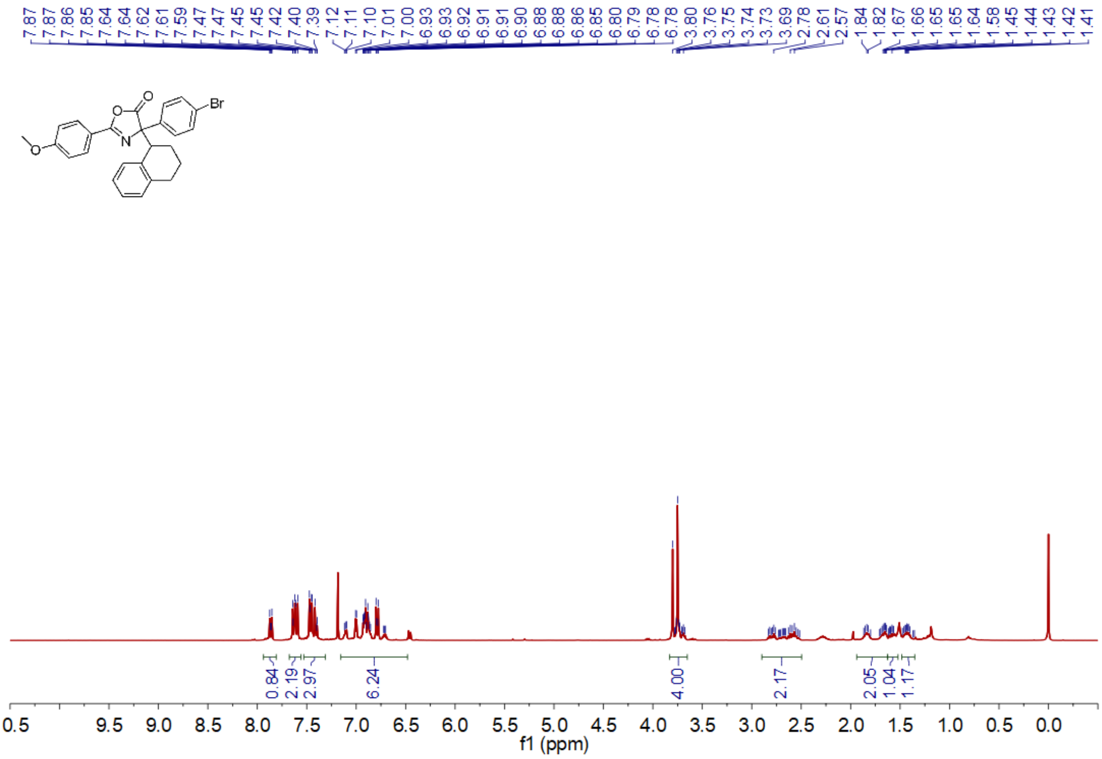

Supplementary Figure 122 ^{1}H NMR (400 MHz, $CDCl_3$, 25 °C) of compound **35**

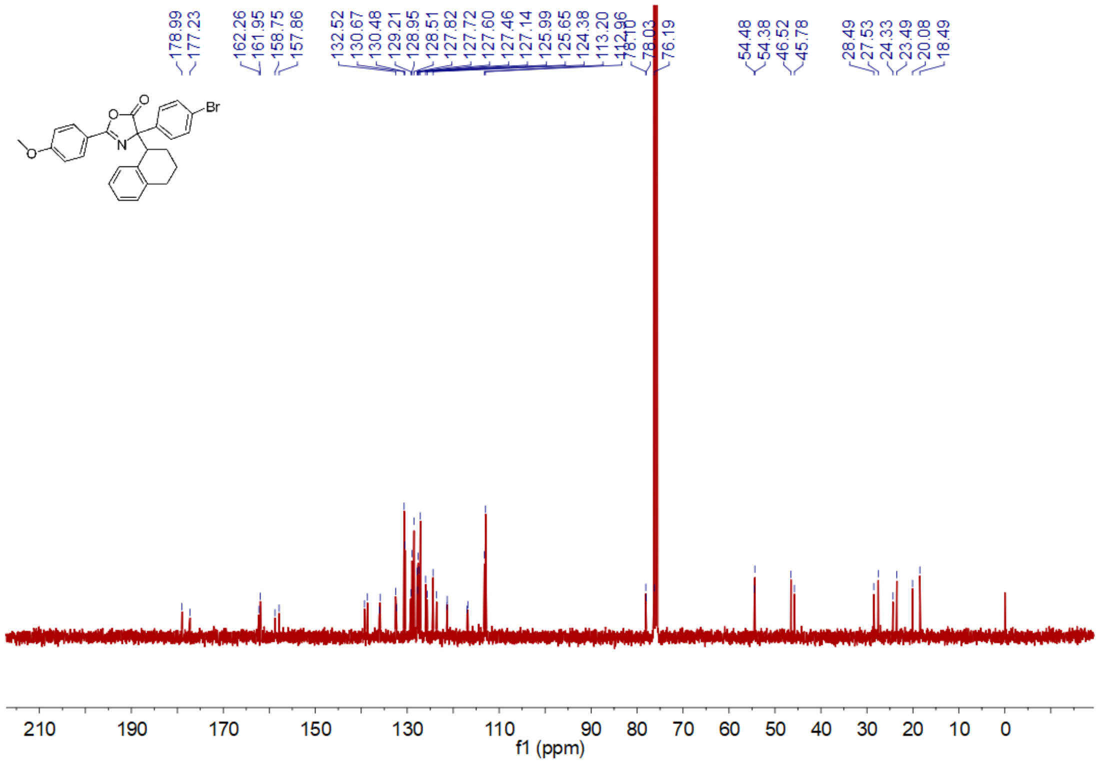

Supplementary Figure 123 ^{13}C NMR (101 MHz, $CDCl_3$, 25 °C) of compound **35**

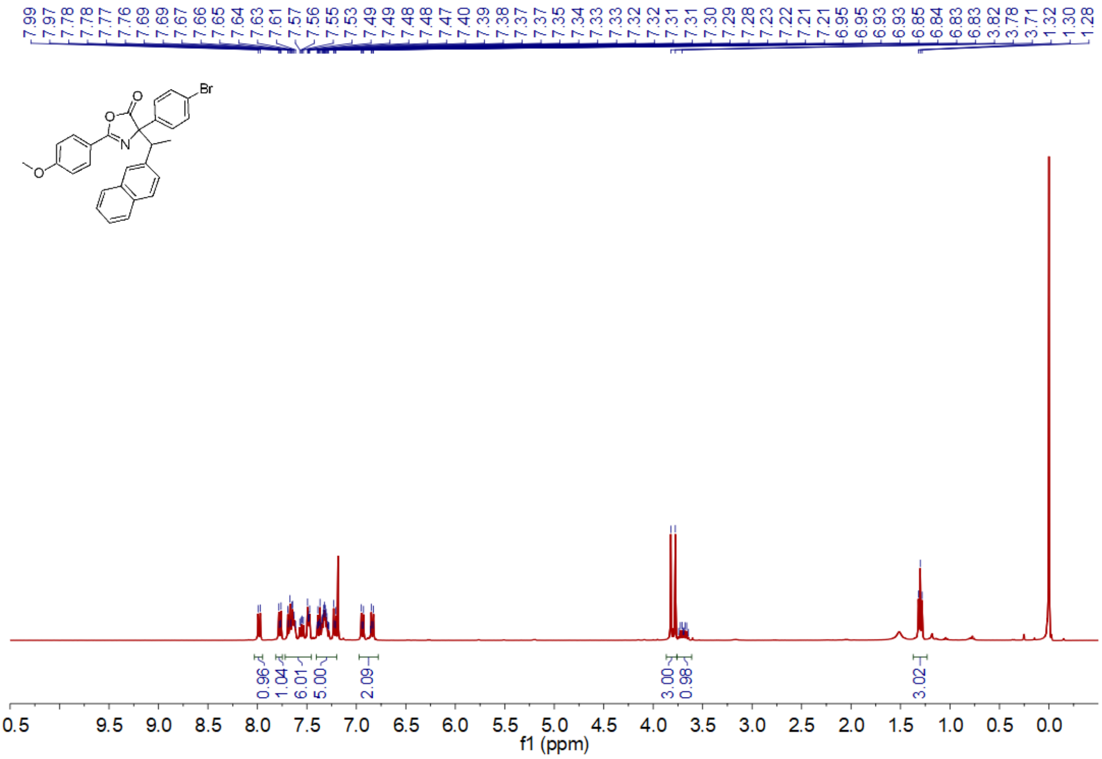

Supplementary Figure 124 ^{1}H NMR (400 MHz, $CDCl_3$, 25 °C) of compound **36**

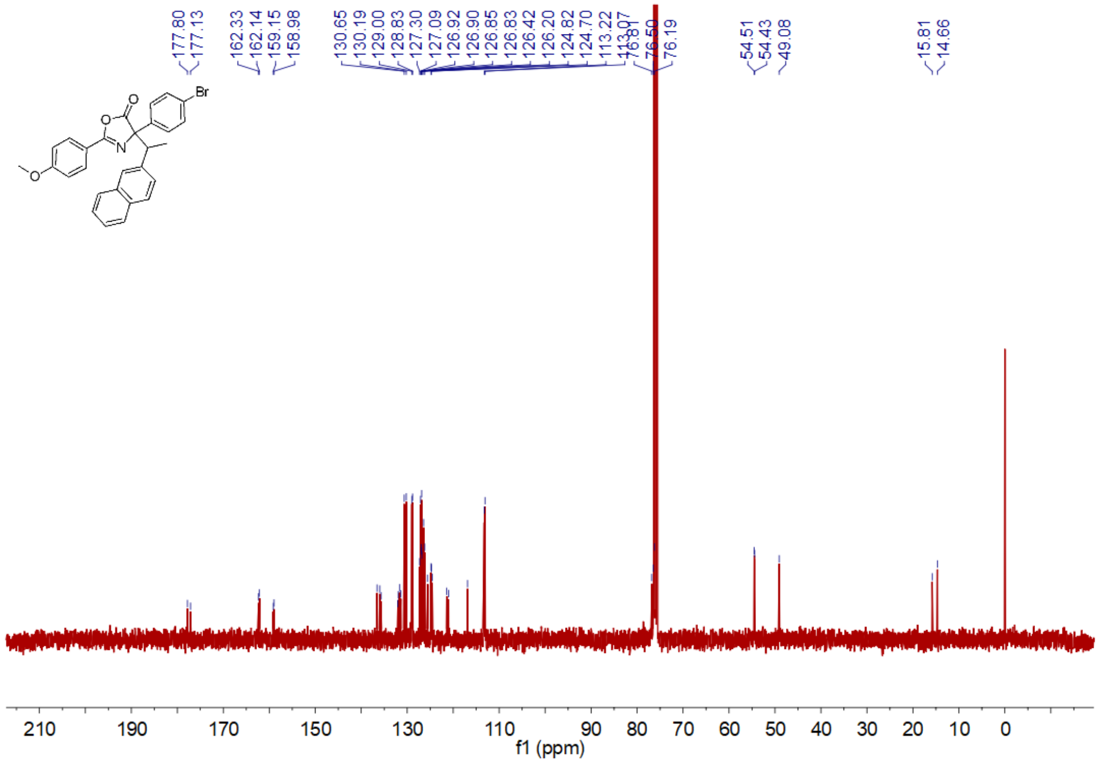

Supplementary Figure 125 ^{13}C NMR (101 MHz, $CDCl_3$, 25 °C) of compound **36**

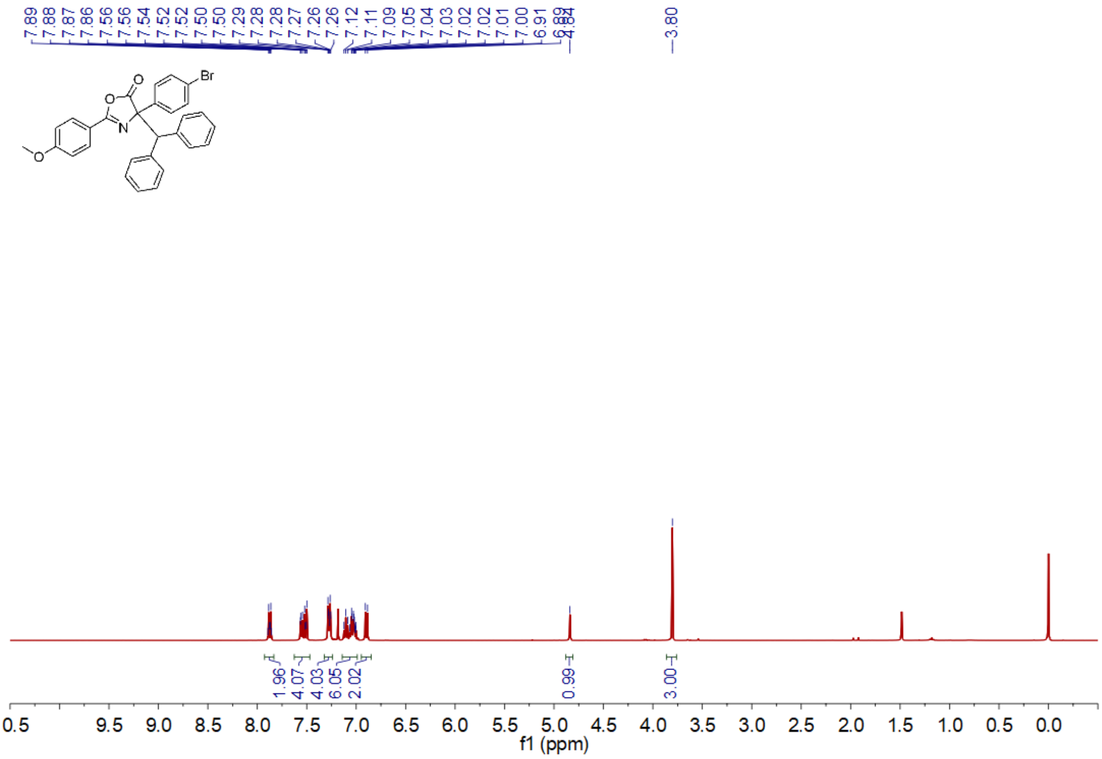

Supplementary Figure 126 ^{1}H NMR (400 MHz, $CDCl_3$, 25 °C) of compound **37**

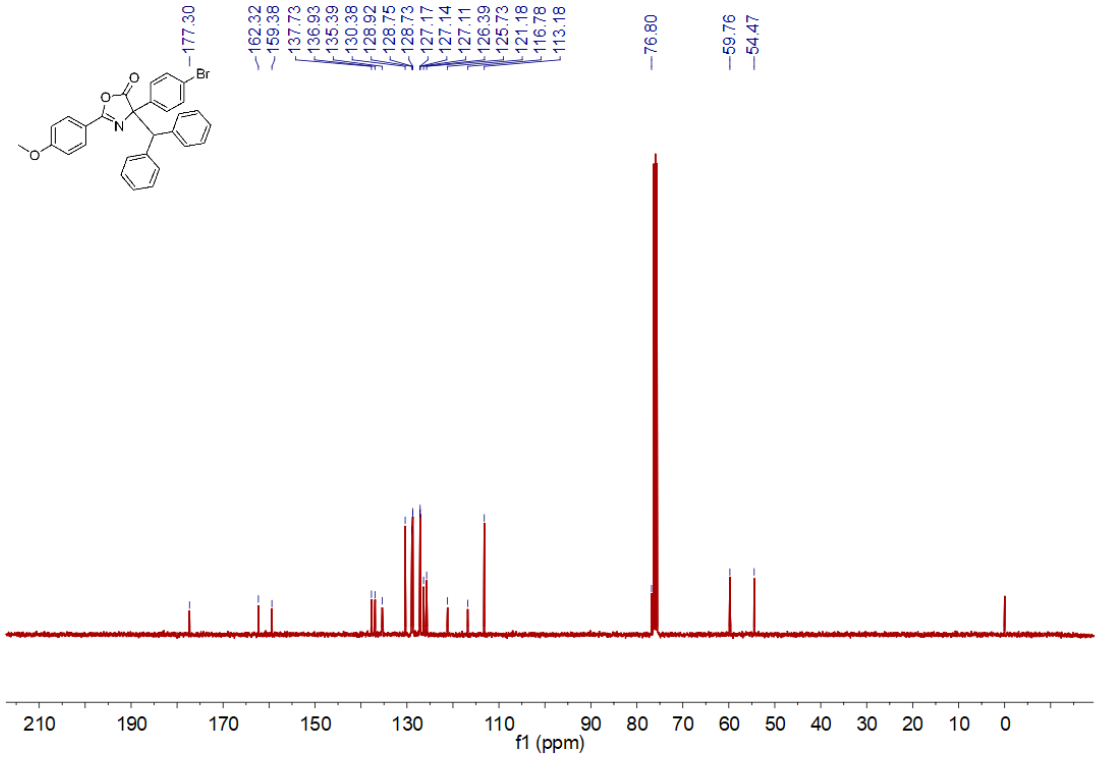

Supplementary Figure 127 ^{13}C NMR (101 MHz, $CDCl_3$, 25 °C) of compound **37**

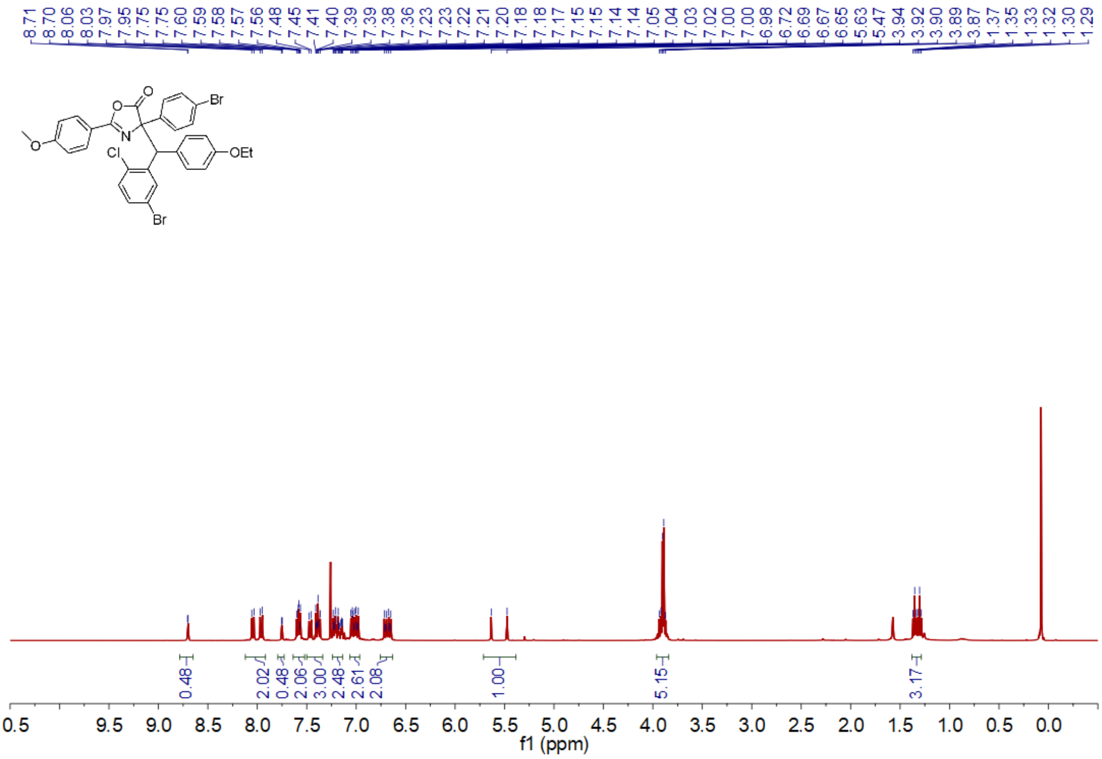

Supplementary Figure 128 ^{1}H NMR (400 MHz, $CDCl_3$, 25 °C) of compound **38**

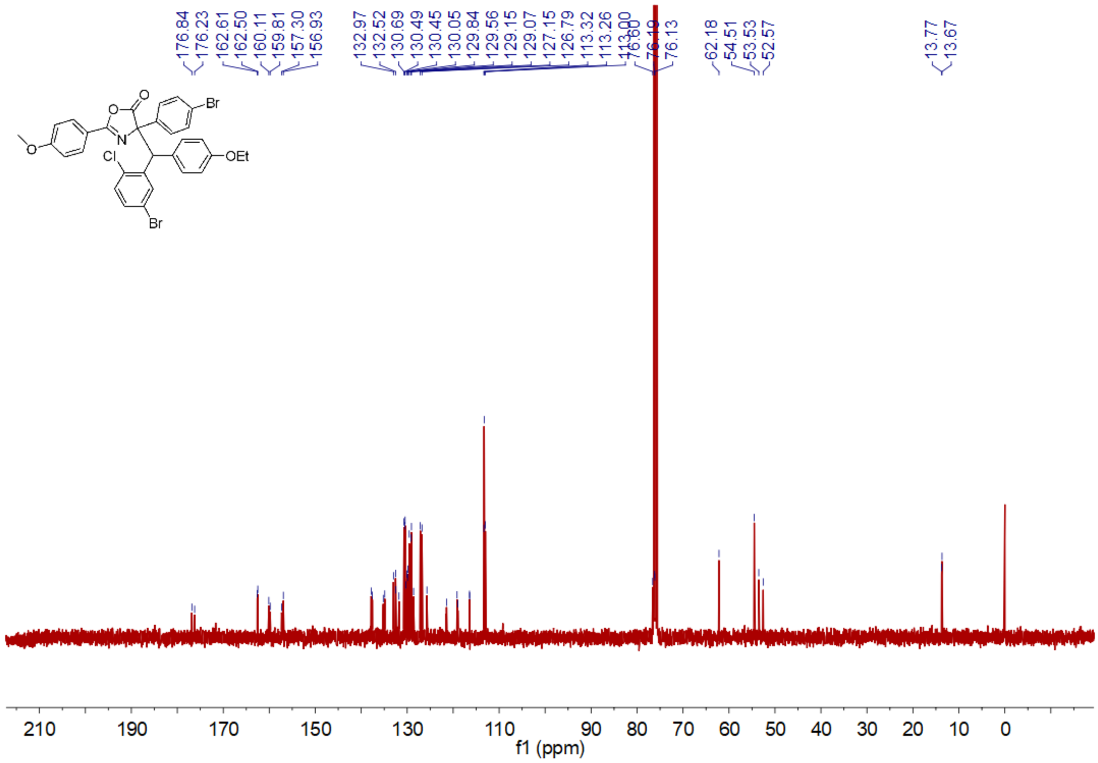

Supplementary Figure 129 ^{13}C NMR (101 MHz, $CDCl_3$, 25 °C) of compound **38**

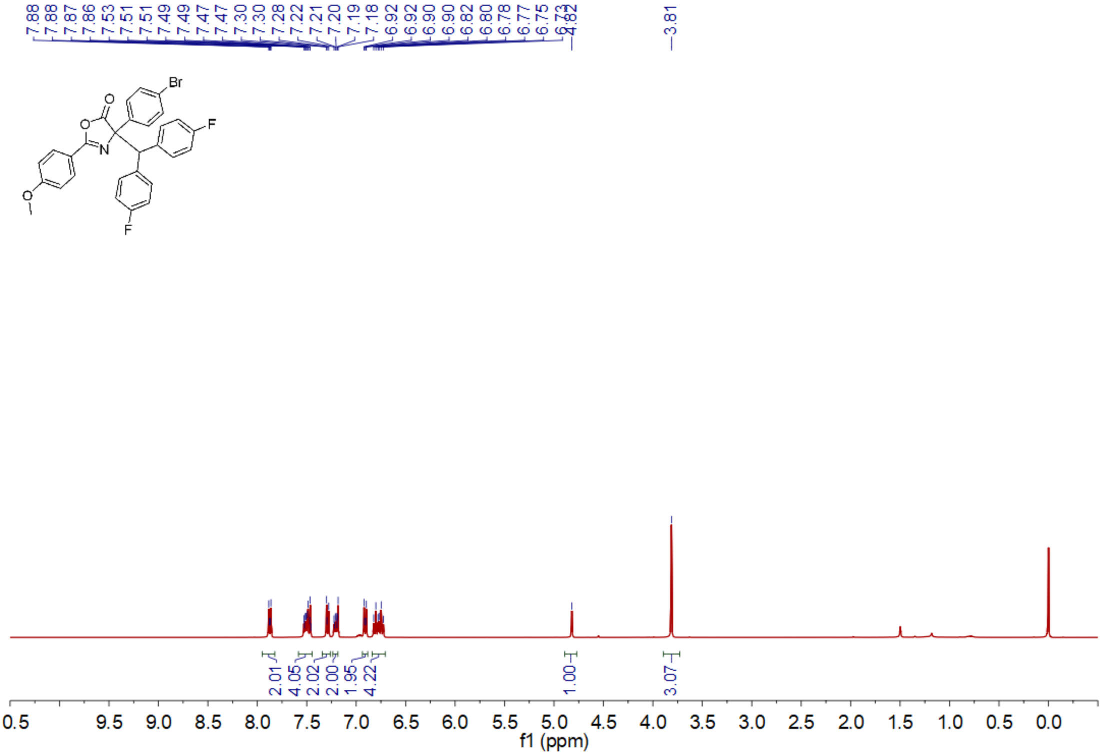

Supplementary Figure 130 ^{1}H NMR (400 MHz, $CDCl_3$, 25 °C) of compound **39**

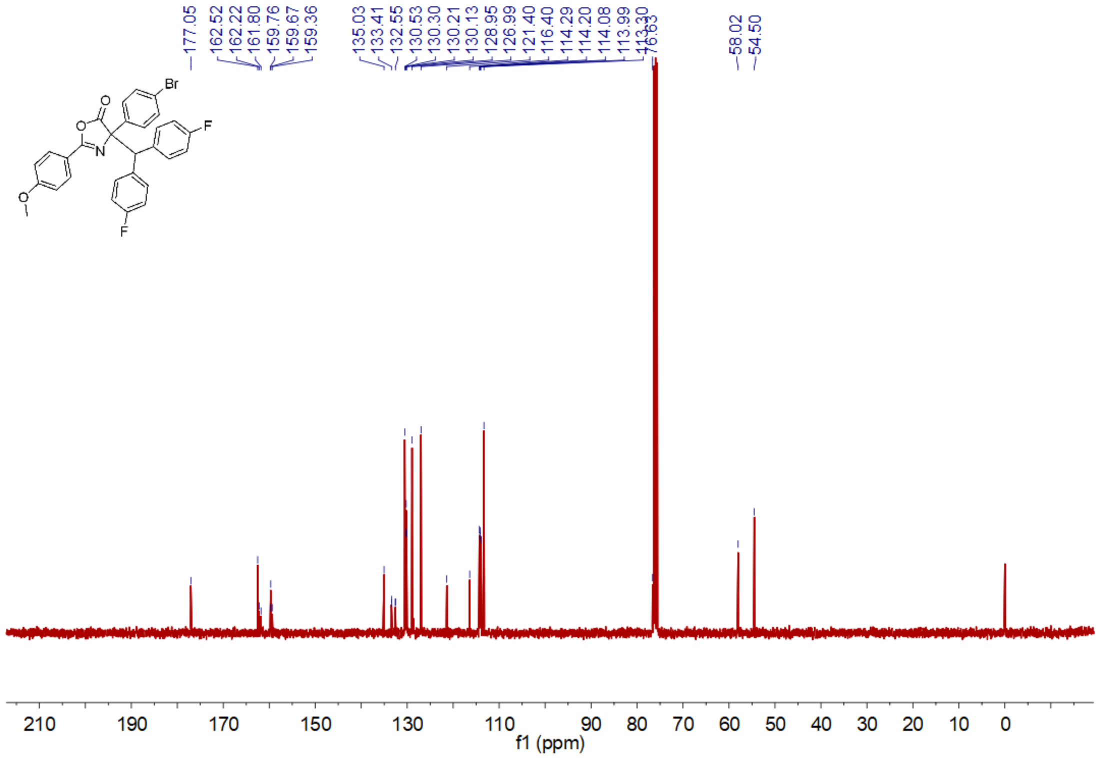

Supplementary Figure 131 ^{13}C NMR (101 MHz, $CDCl_3$, 25 °C) of compound **39**

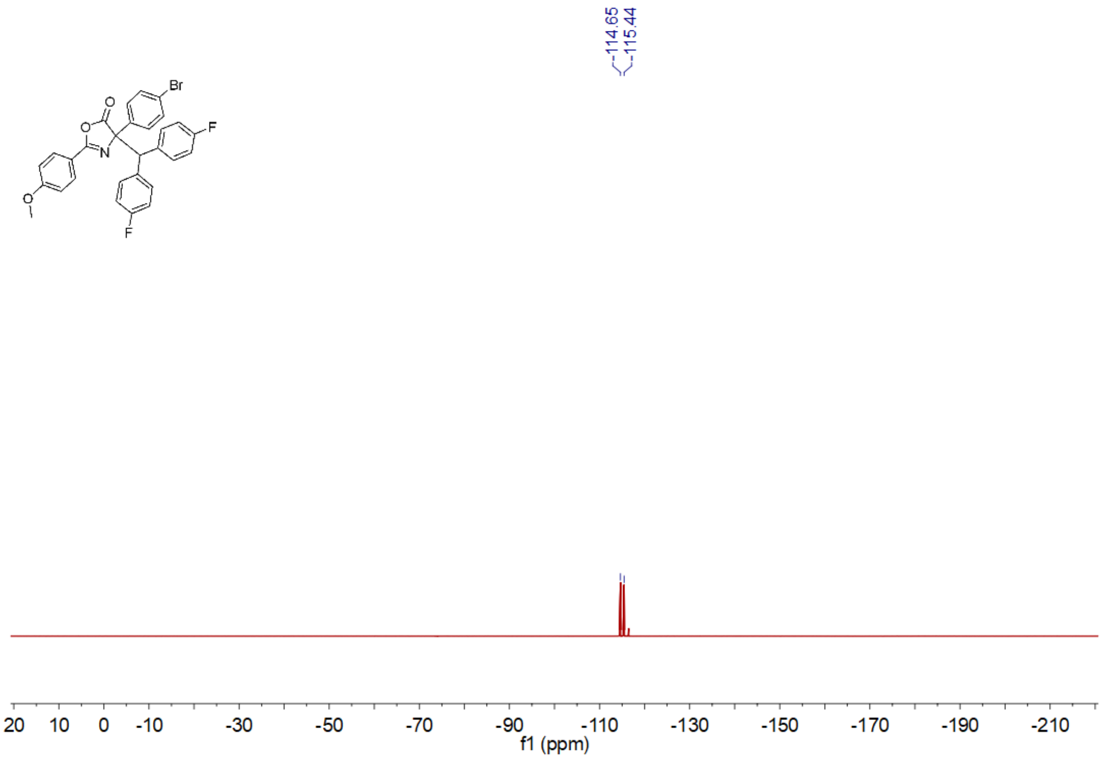

Supplementary Figure 132 ^{19}F NMR (377 MHz, $CDCl_3$, 25 °C) of compound **39**

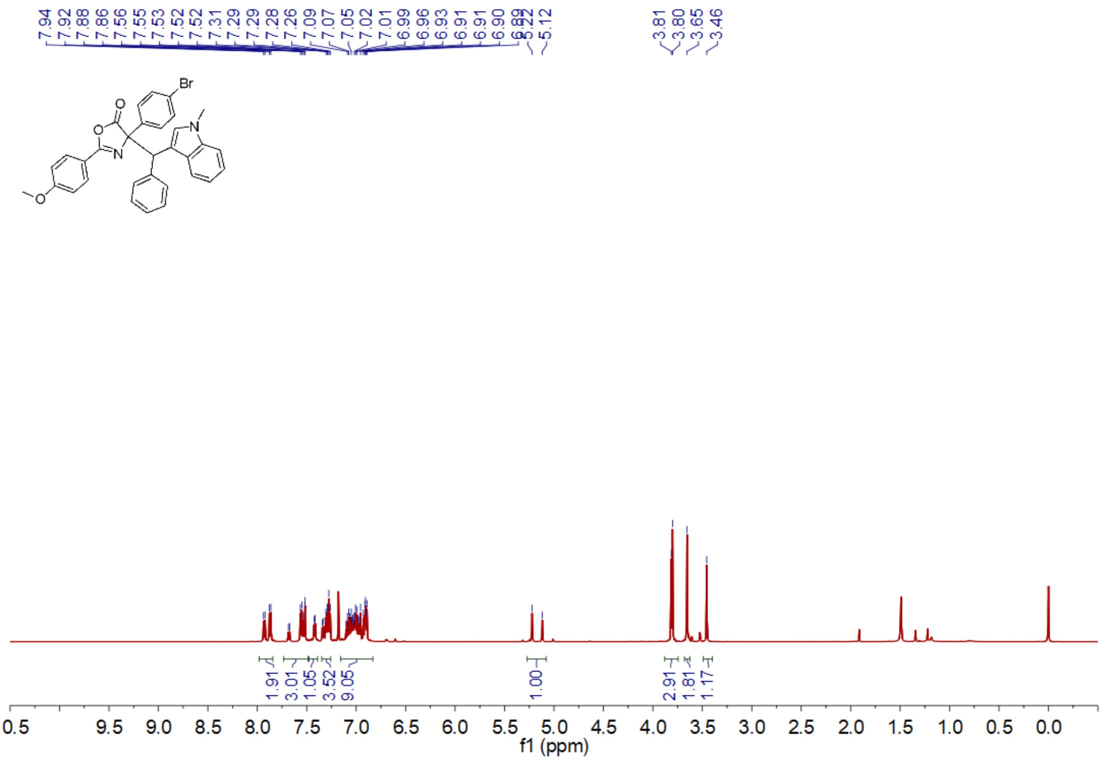

Supplementary Figure 133 ^{1}H NMR (600 MHz, $CDCl_3$, 25 °C) of compound **40**

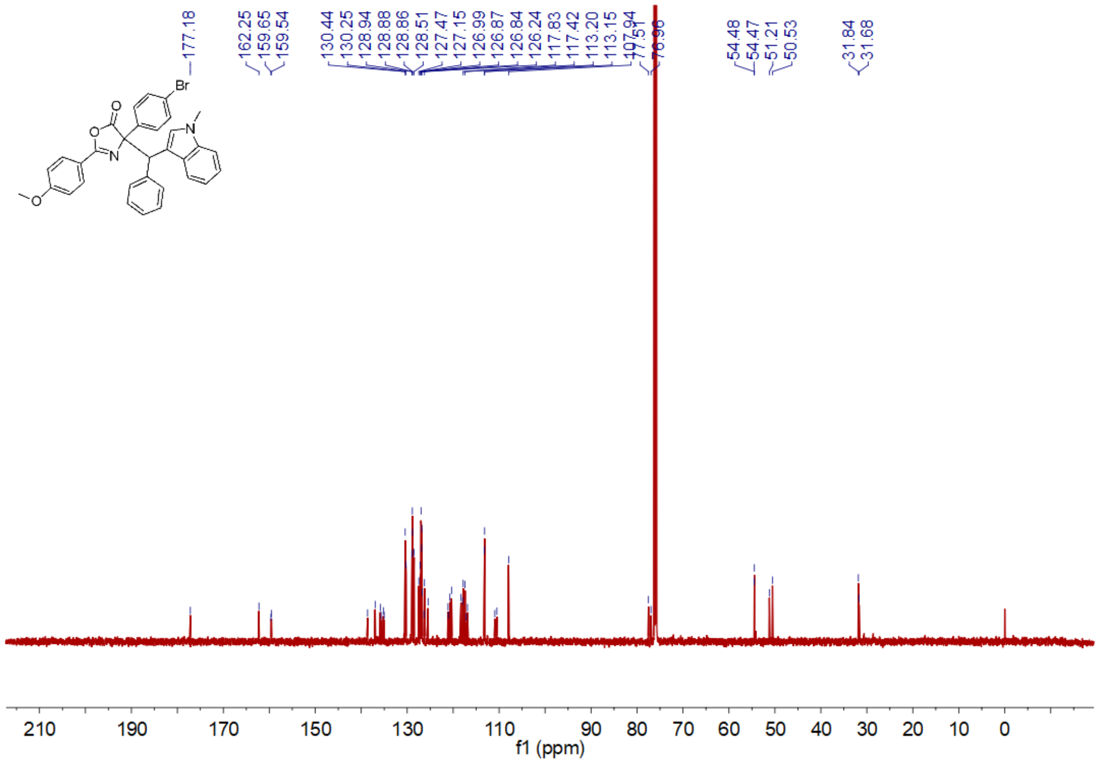

Supplementary Figure 134 ^{13}C NMR (151 MHz, $CDCl_3$, 25 °C) of compound **40**

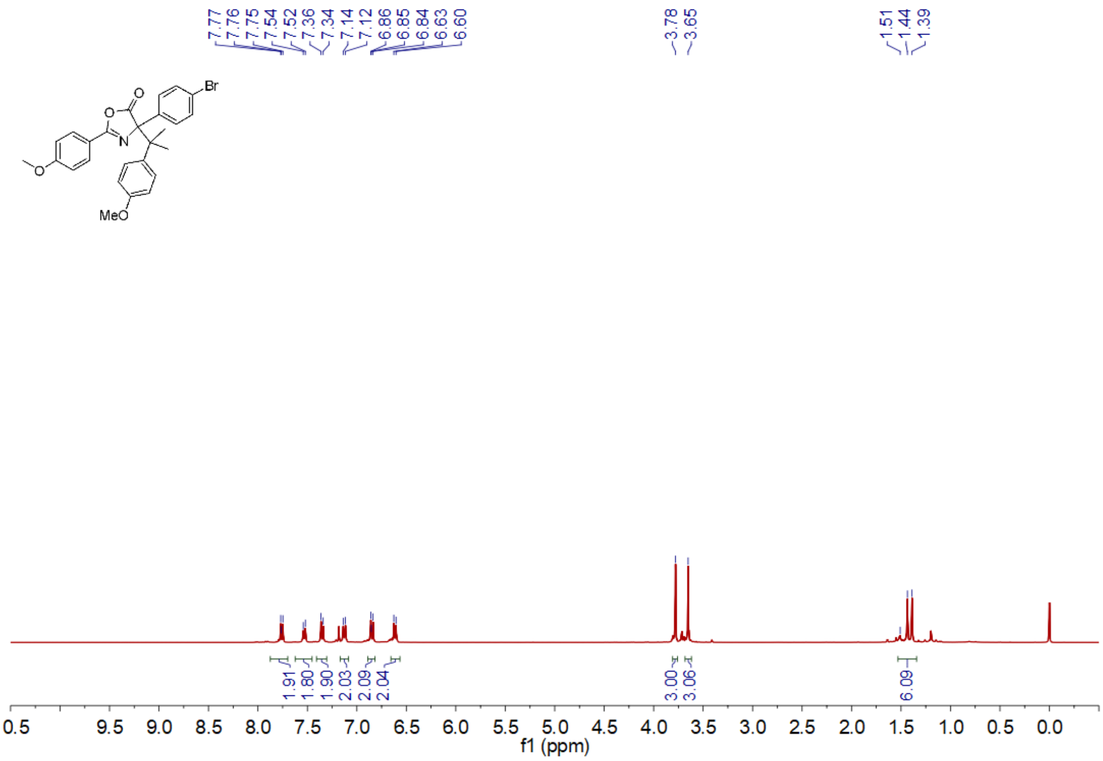

Supplementary Figure 135 ^{1}H NMR (400 MHz, $CDCl_3$, 25 °C) of compound **41**

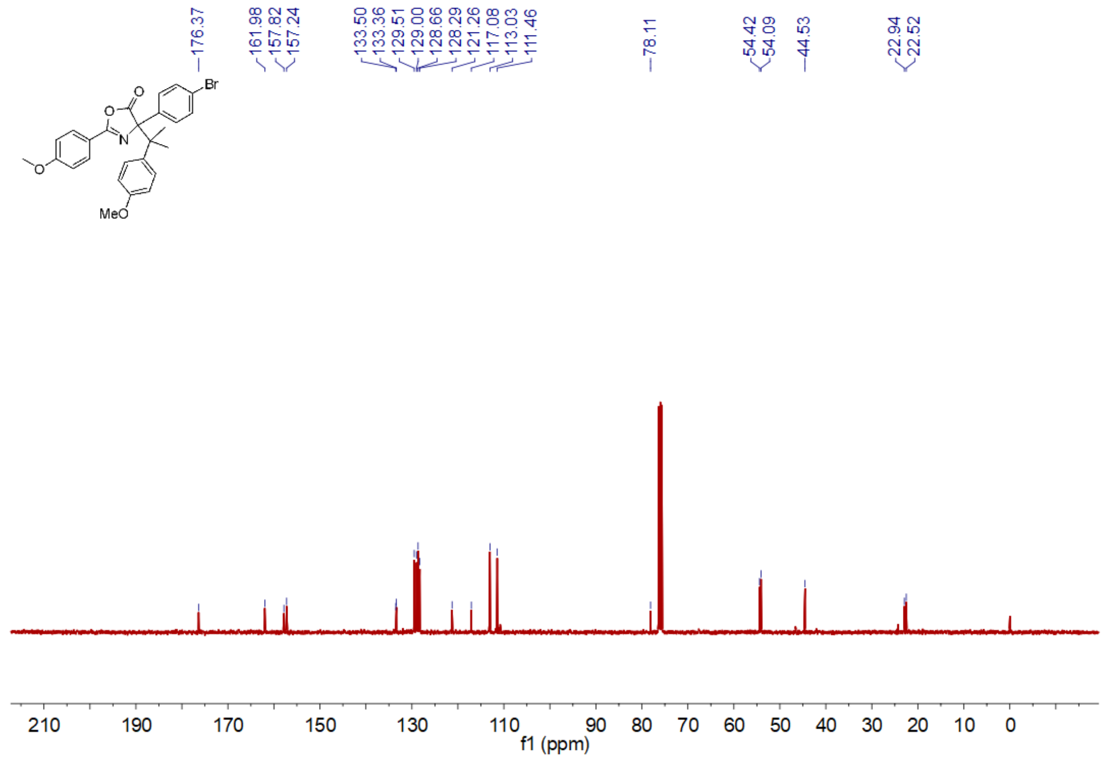

Supplementary Figure 136 ^{13}C NMR (101 MHz, $CDCl_3$, 25 °C) of compound **41**

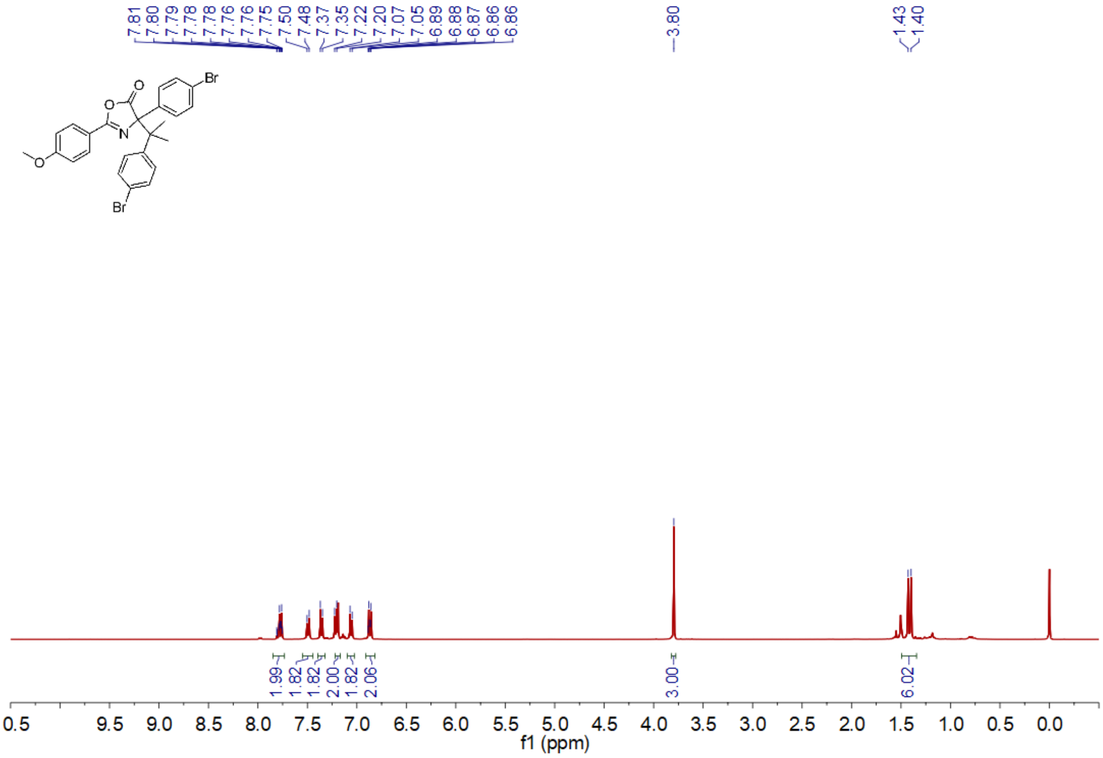

Supplementary Figure 137 ^{1}H NMR (400 MHz, $CDCl_3$, 25 °C) of compound **42**

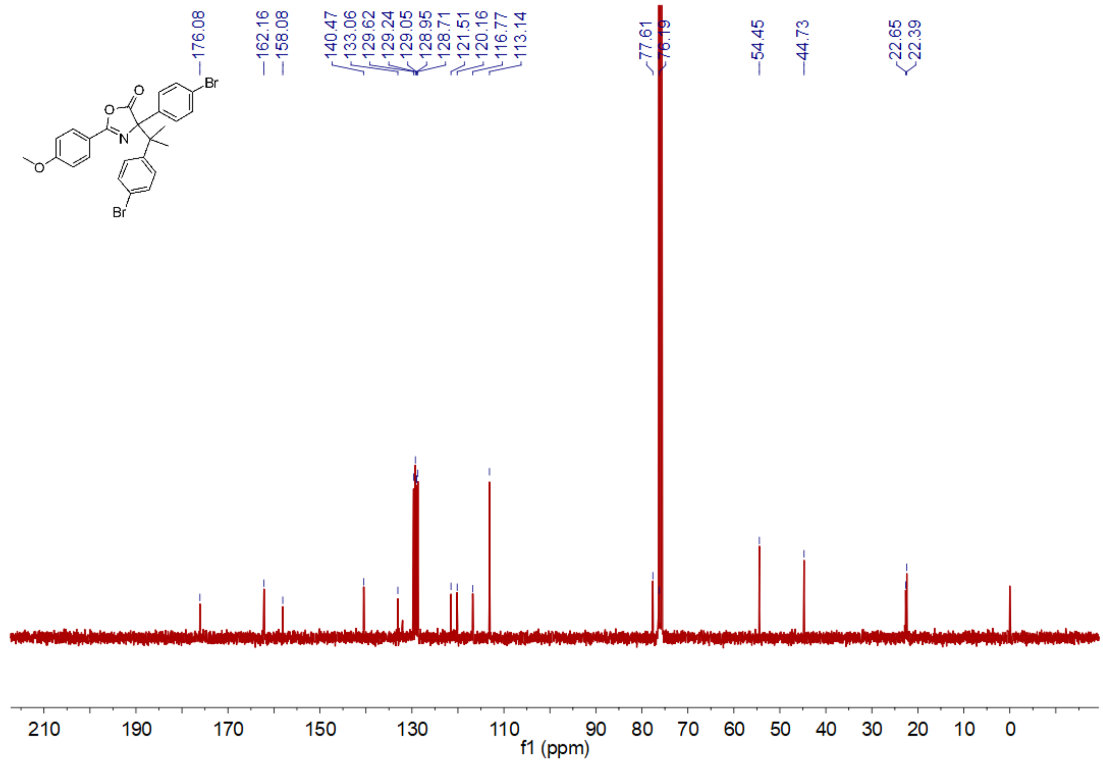

Supplementary Figure 138 ^{13}C NMR (101 MHz, $CDCl_3$, 25 °C) of compound **42**

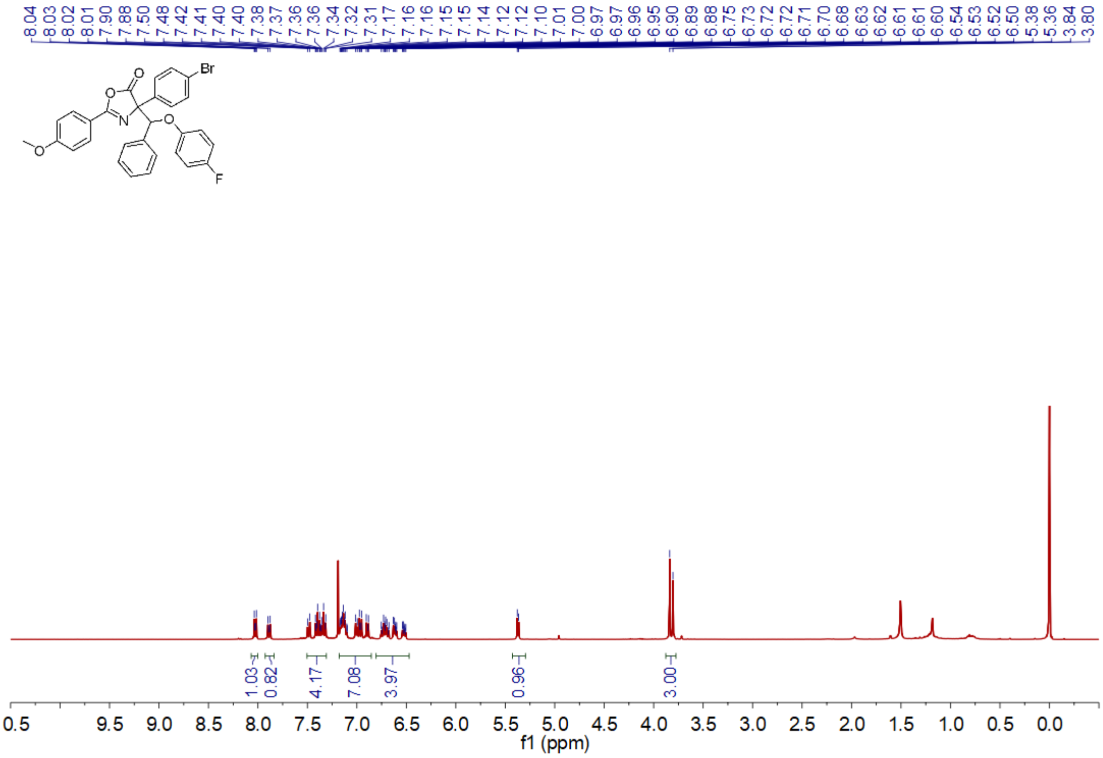

Supplementary Figure 139 ^{1}H NMR (400 MHz, $CDCl_3$, 25 °C) of compound **43**

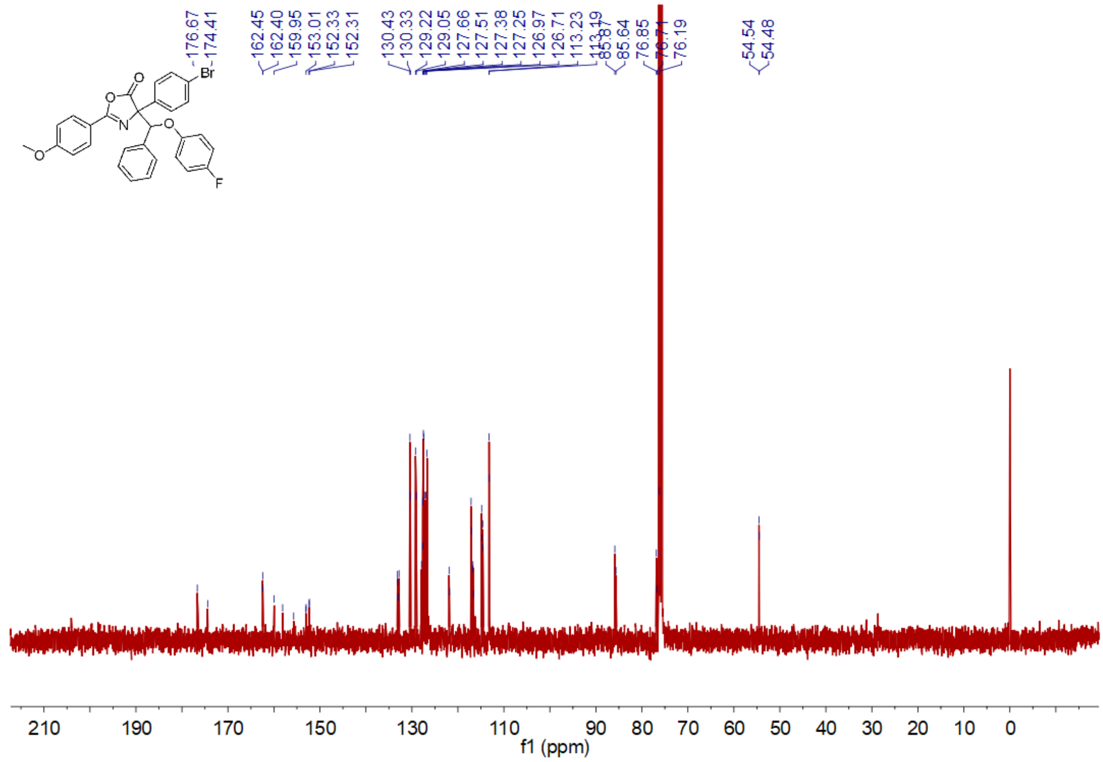

Supplementary Figure 140 ^{13}C NMR (101 MHz, $CDCl_3$, 25 °C) of compound **43**

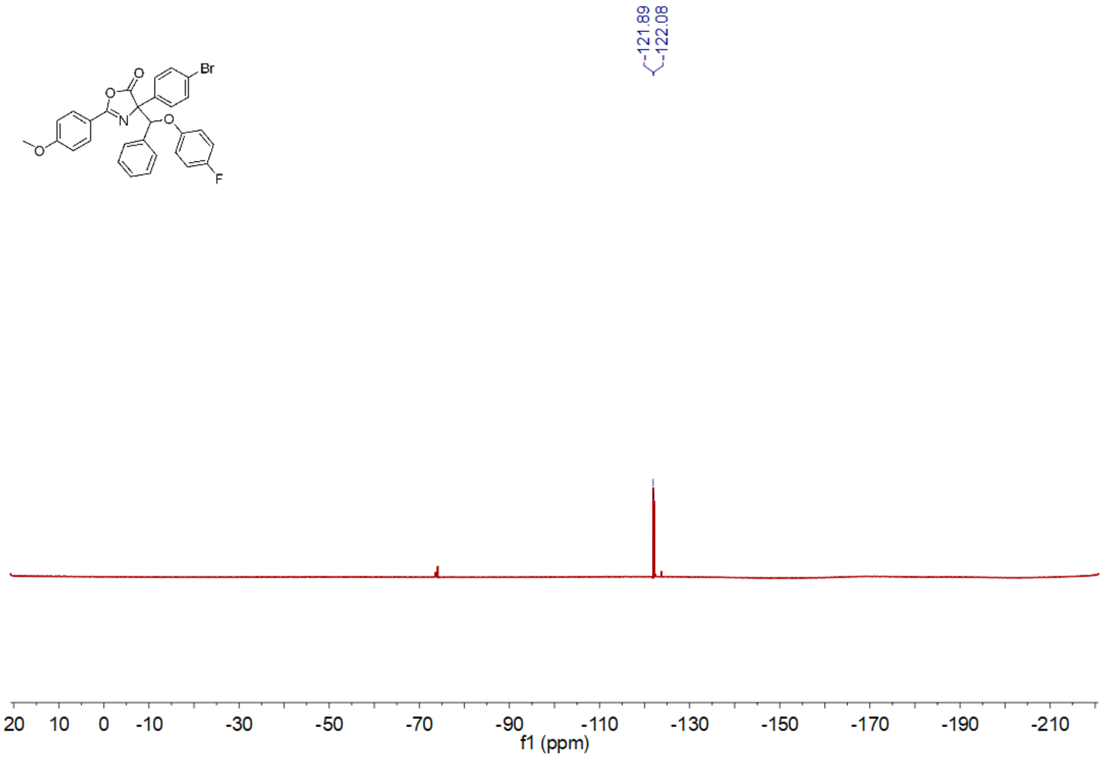

Supplementary Figure 141 ^{19}F NMR (377 MHz, CDCl$_3$, 25 °C) of compound **43**

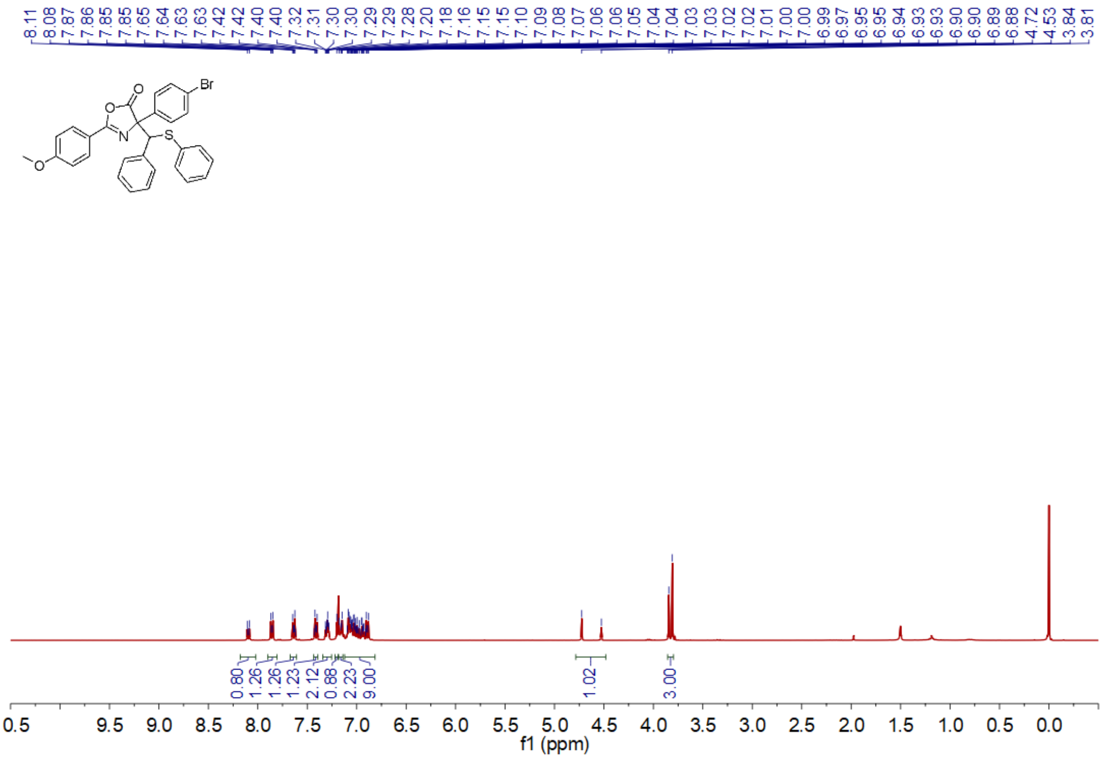

Supplementary Figure 142 ^{1}H NMR (400 MHz, CDCl$_3$, 25 °C) of compound **44**

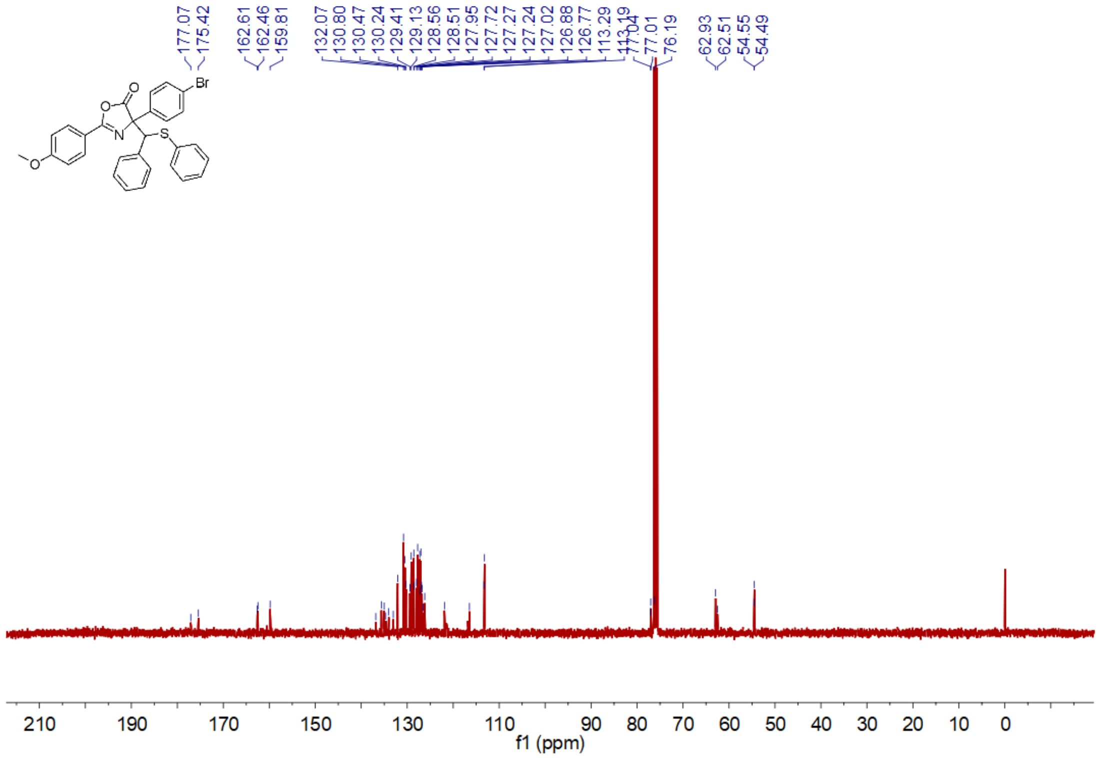

Supplementary Figure 143 ^{13}C NMR (101 MHz, $CDCl_3$, 25 °C) of compound **44**

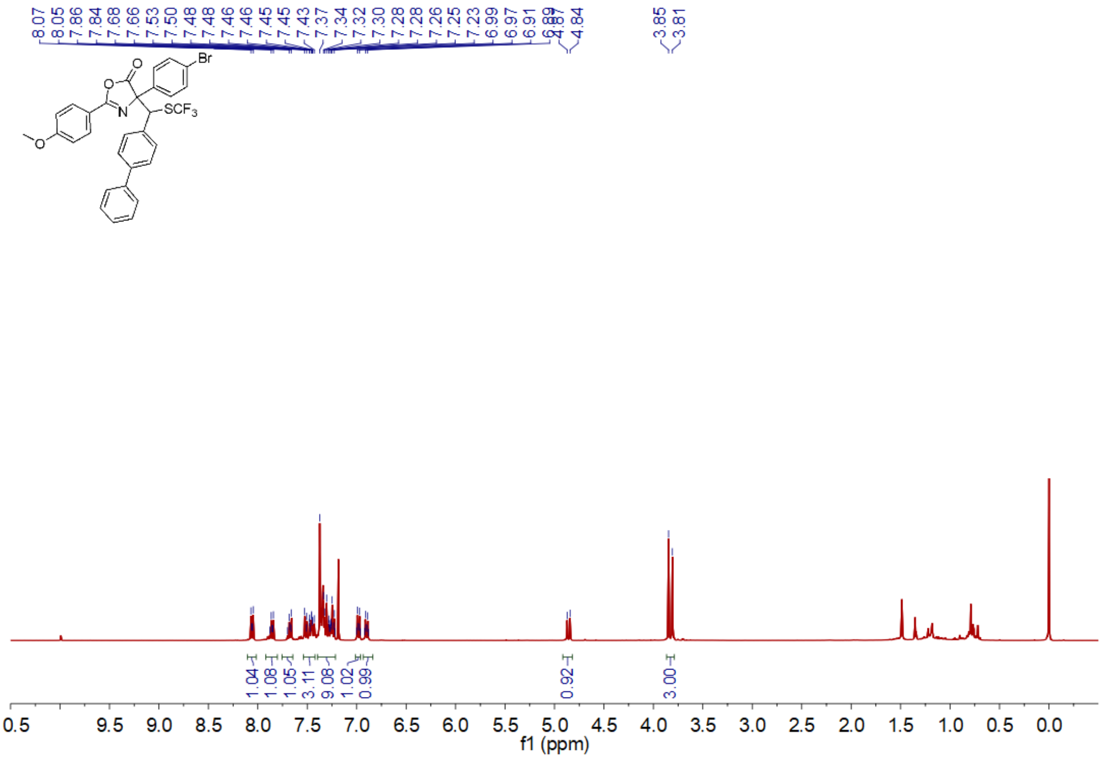

Supplementary Figure 144 ^{1}H NMR (400 MHz, $CDCl_3$, 25 °C) of compound **45**

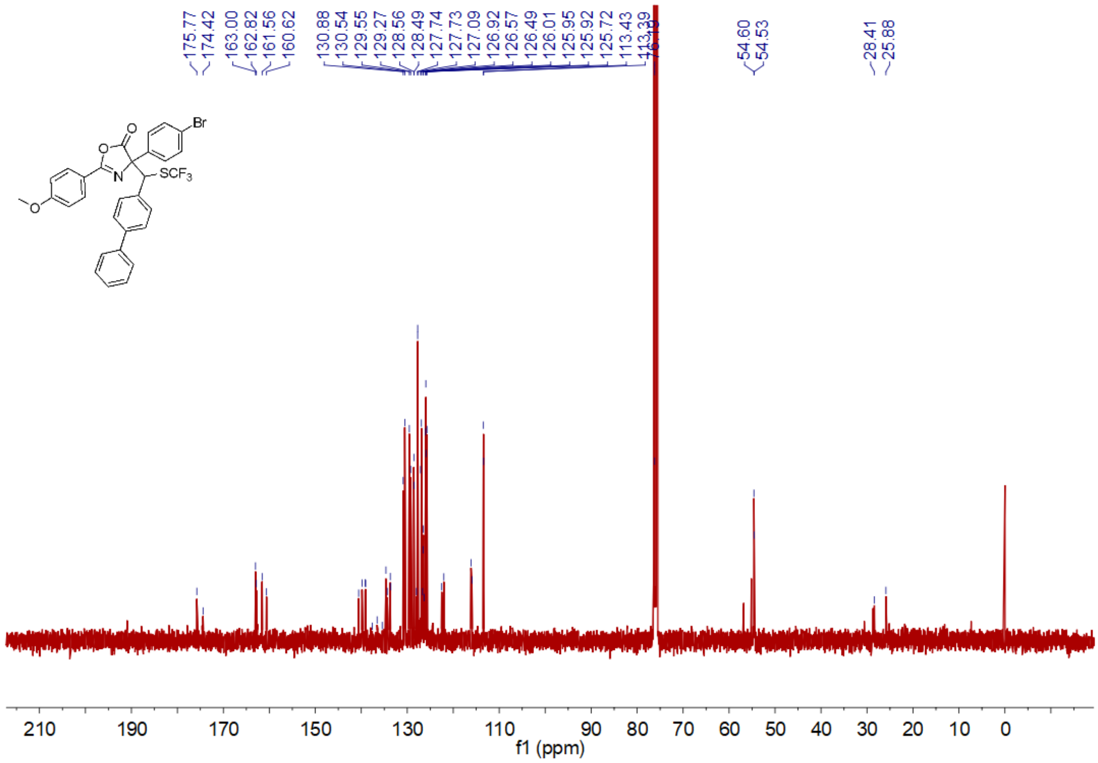

Supplementary Figure 145 ^{13}C NMR (101 MHz, $CDCl_3$, 25 °C) of compound **45**

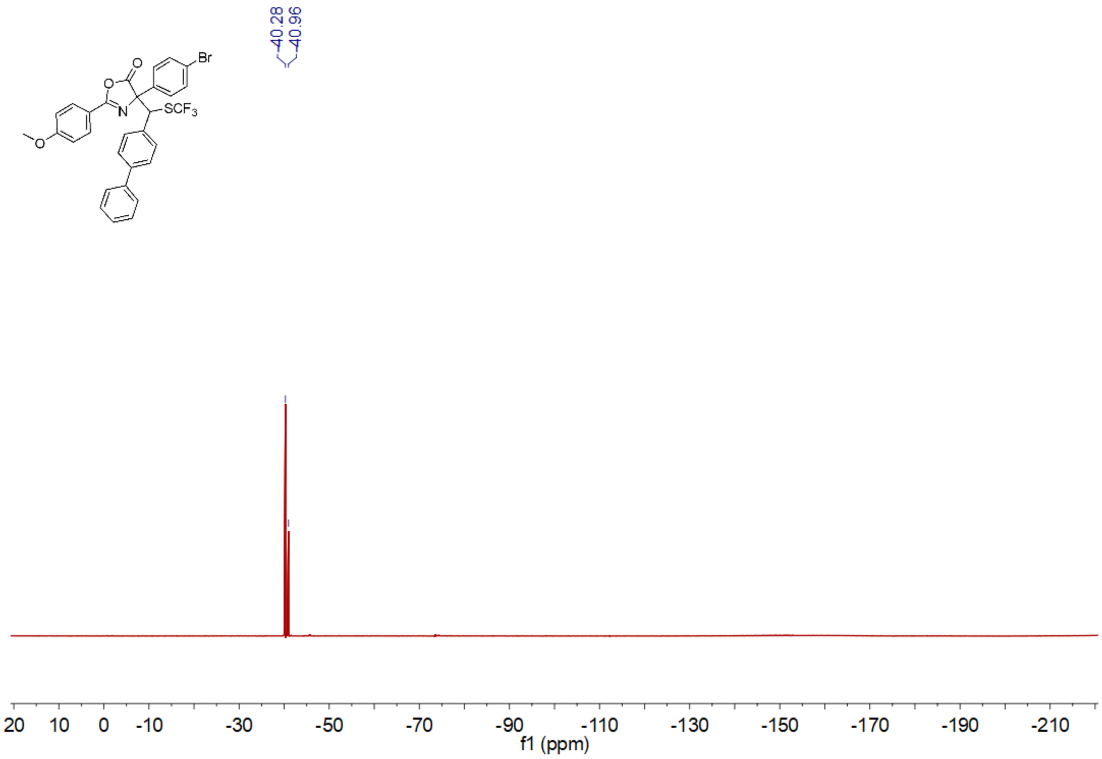

Supplementary Figure 146 ^{19}F NMR (377 MHz, $CDCl_3$, 25 °C) of compound **45**

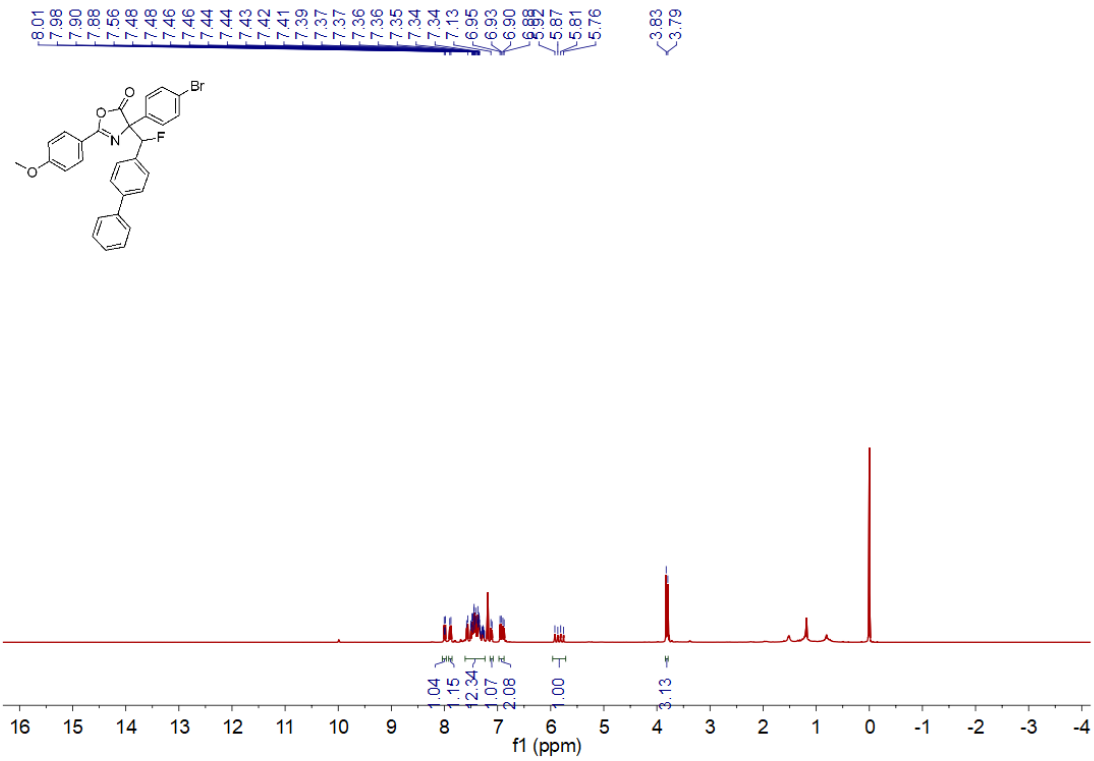

Supplementary Figure 147 ^{1}H NMR (400 MHz, $CDCl_3$, 25 °C) of compound **46**

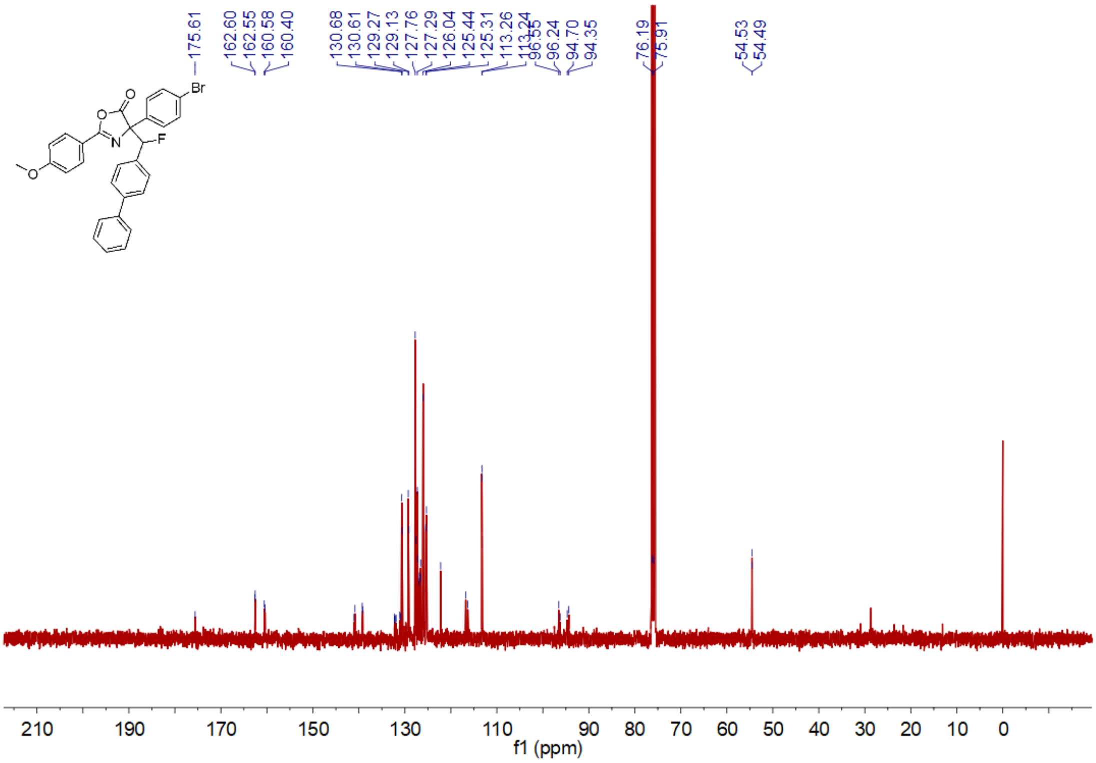

Supplementary Figure 148 ^{13}C NMR (101 MHz, $CDCl_3$, 25 °C) of compound **46**

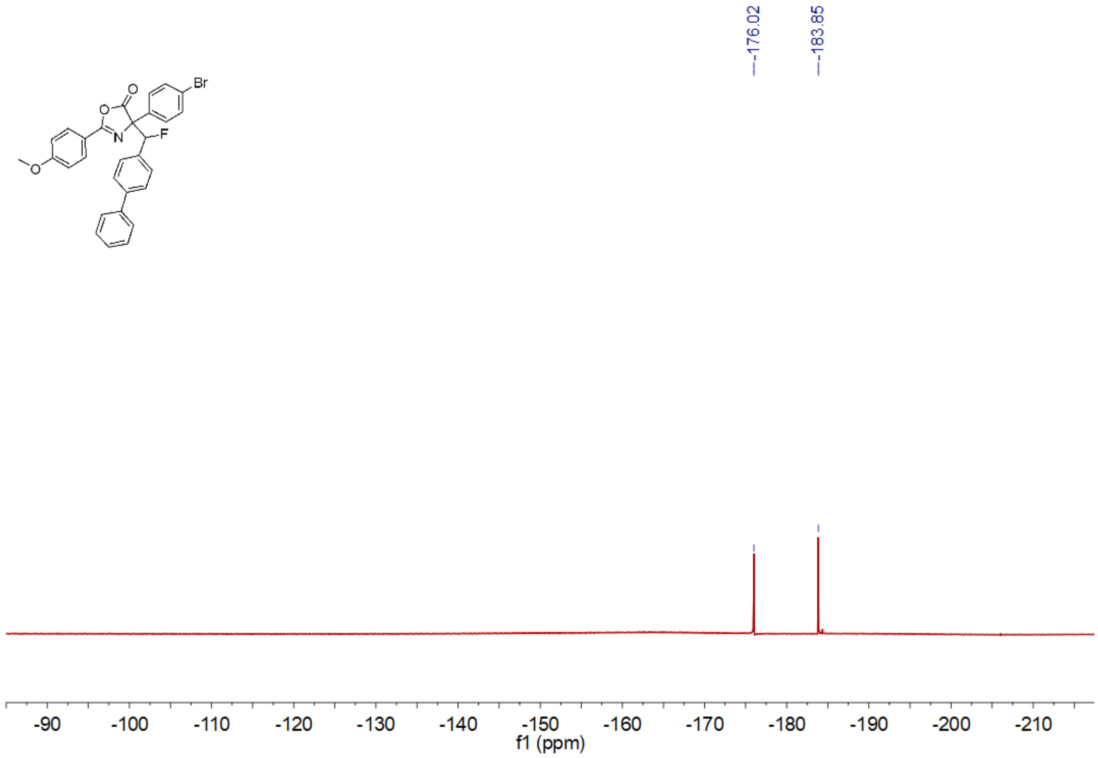

Supplementary Figure 149 ^{19}F NMR (377 MHz, $CDCl_3$, 25 °C) of compound **46**

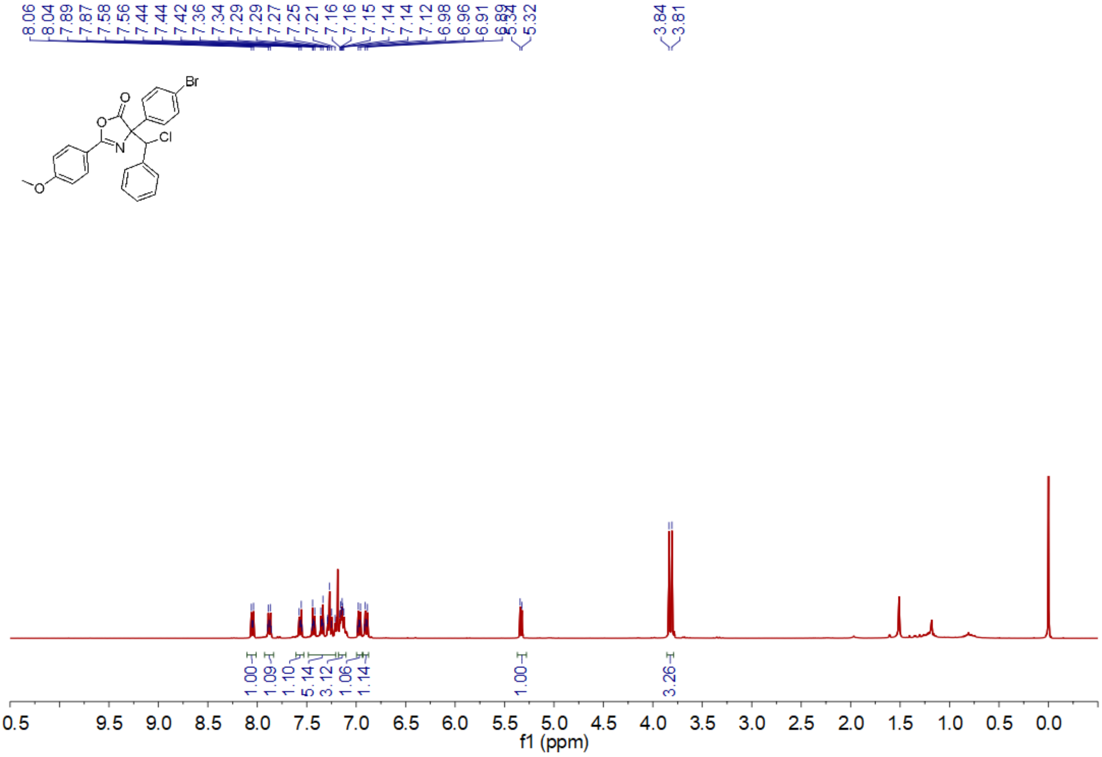

Supplementary Figure 150 ^{1}H NMR (400 MHz, $CDCl_3$, 25 °C) of compound **47**

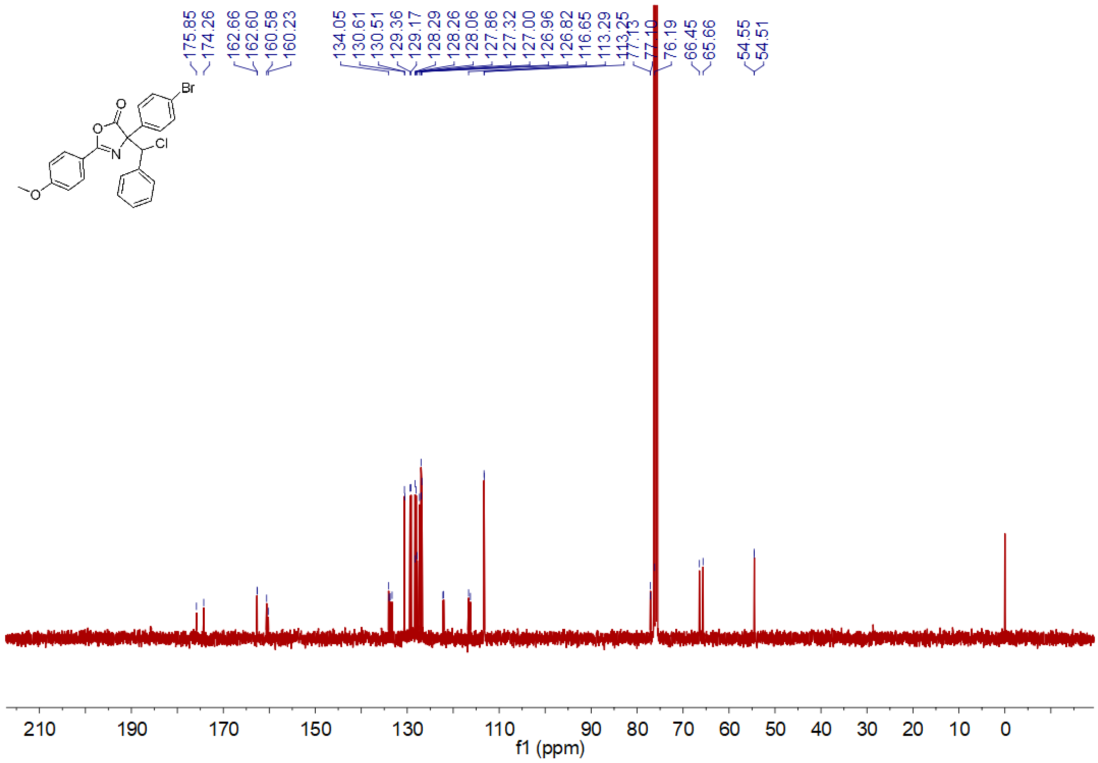

Supplementary Figure 151 ^{13}C NMR (101 MHz, $CDCl_3$, 25 °C) of compound **47**

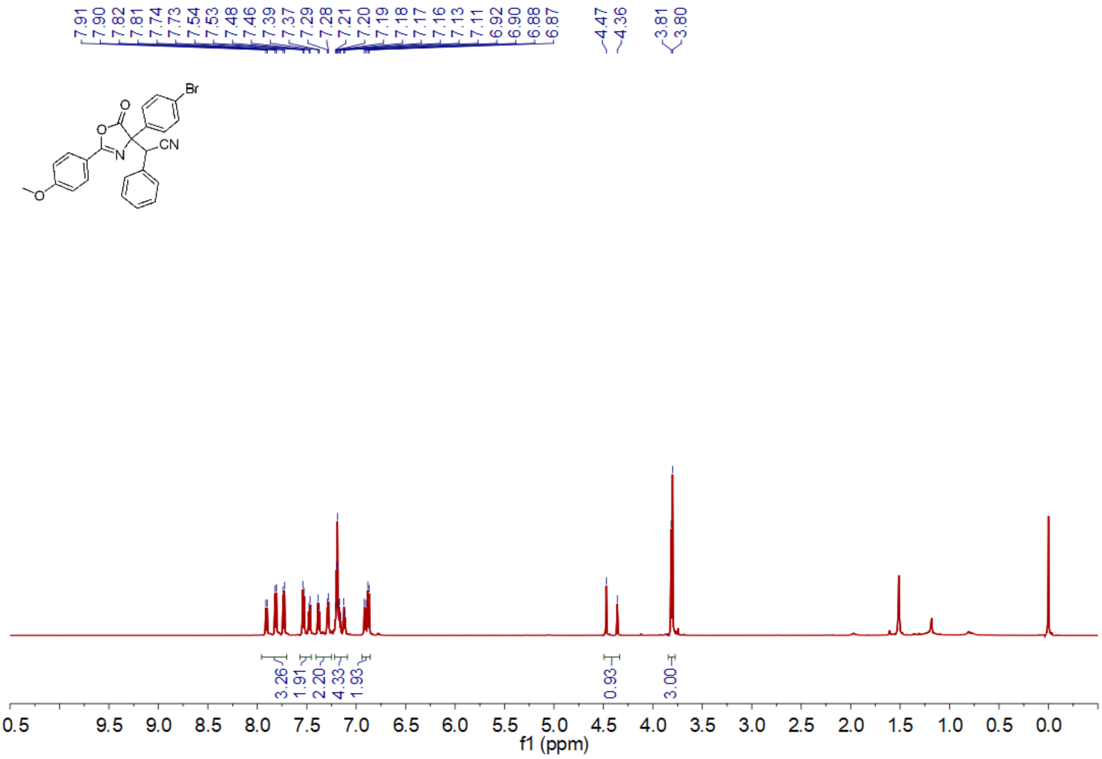

Supplementary Figure 152 ^{1}H NMR (600 MHz, $CDCl_3$, 25 °C) of compound **48**

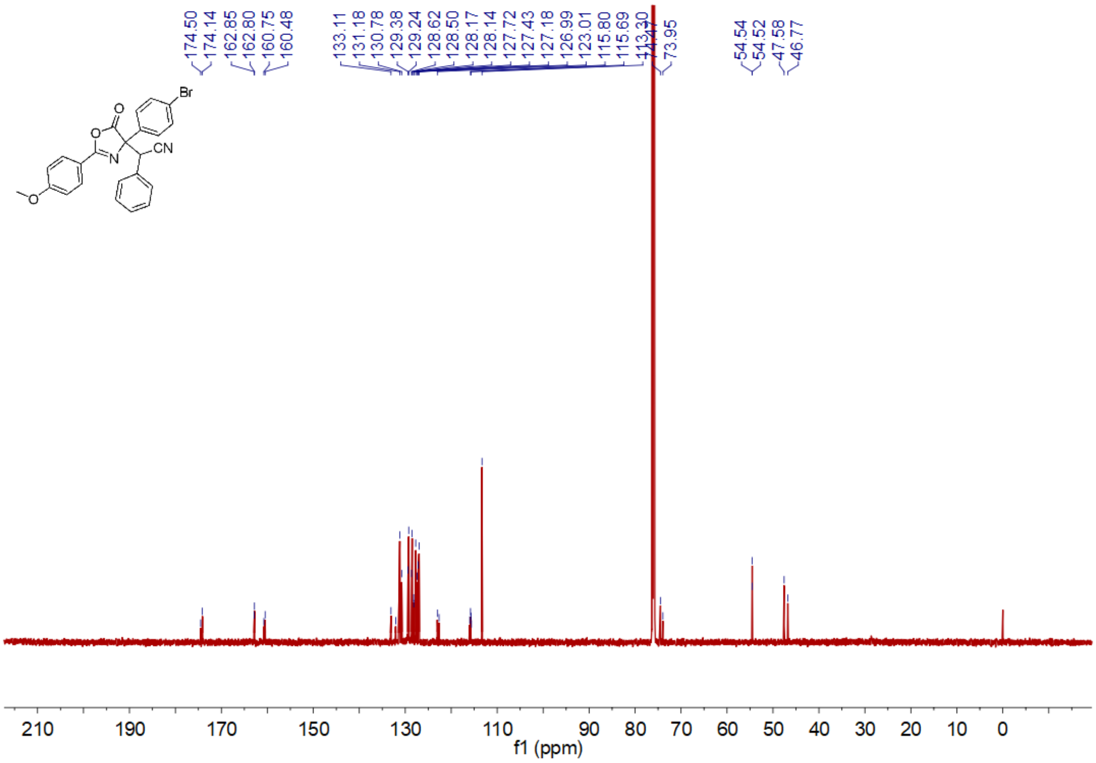

Supplementary Figure 153 ^{13}C NMR (151 MHz, $CDCl_3$, 25 °C) of compound **48**

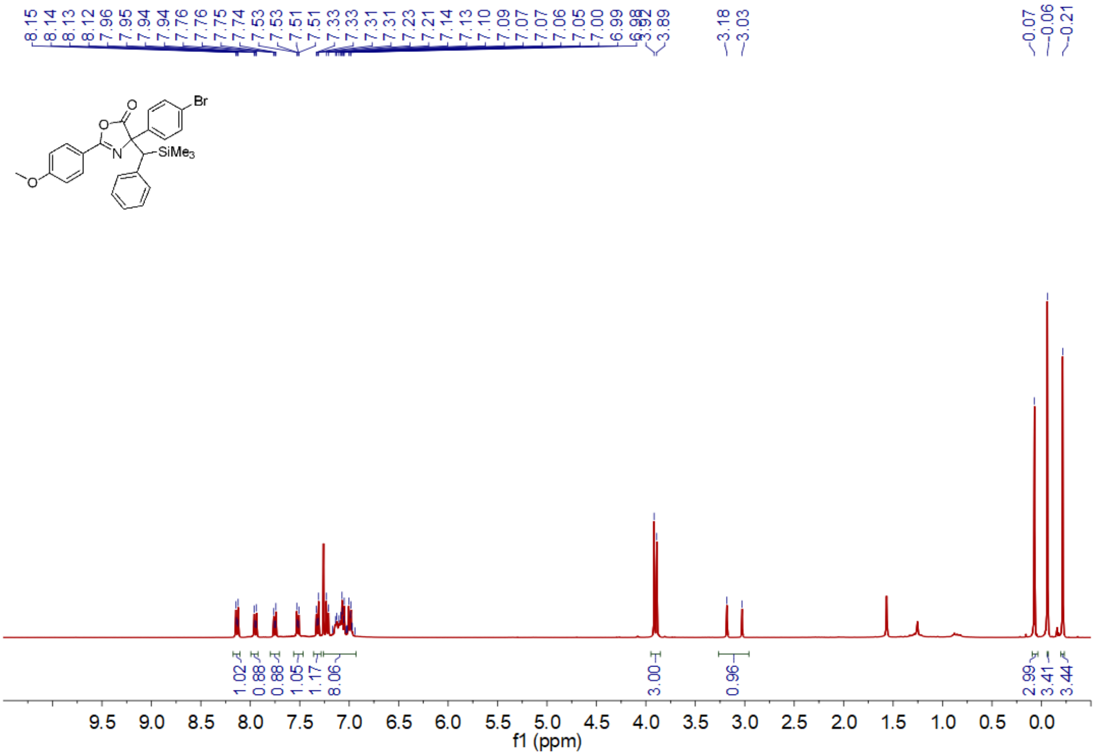

Supplementary Figure 154 ^{1}H NMR (400 MHz, $CDCl_3$, 25 °C) of compound **49**

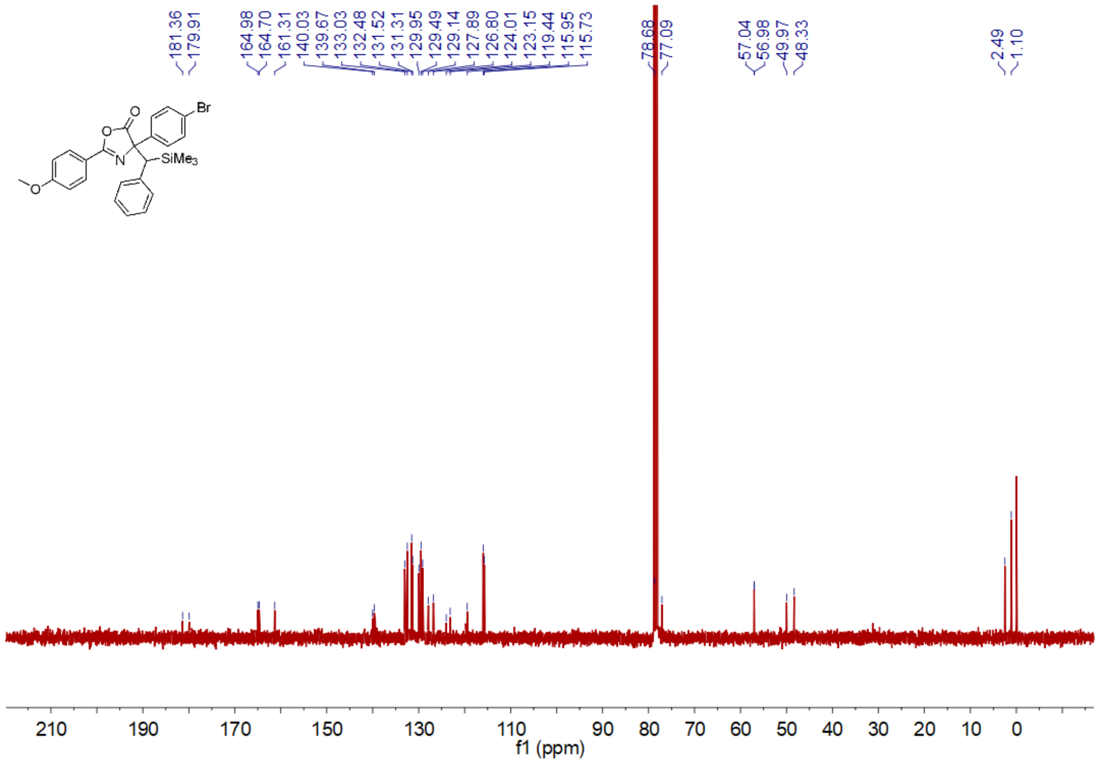

Supplementary Figure 155 ^{13}C NMR (101 MHz, $CDCl_3$, 25 °C) of compound **49**

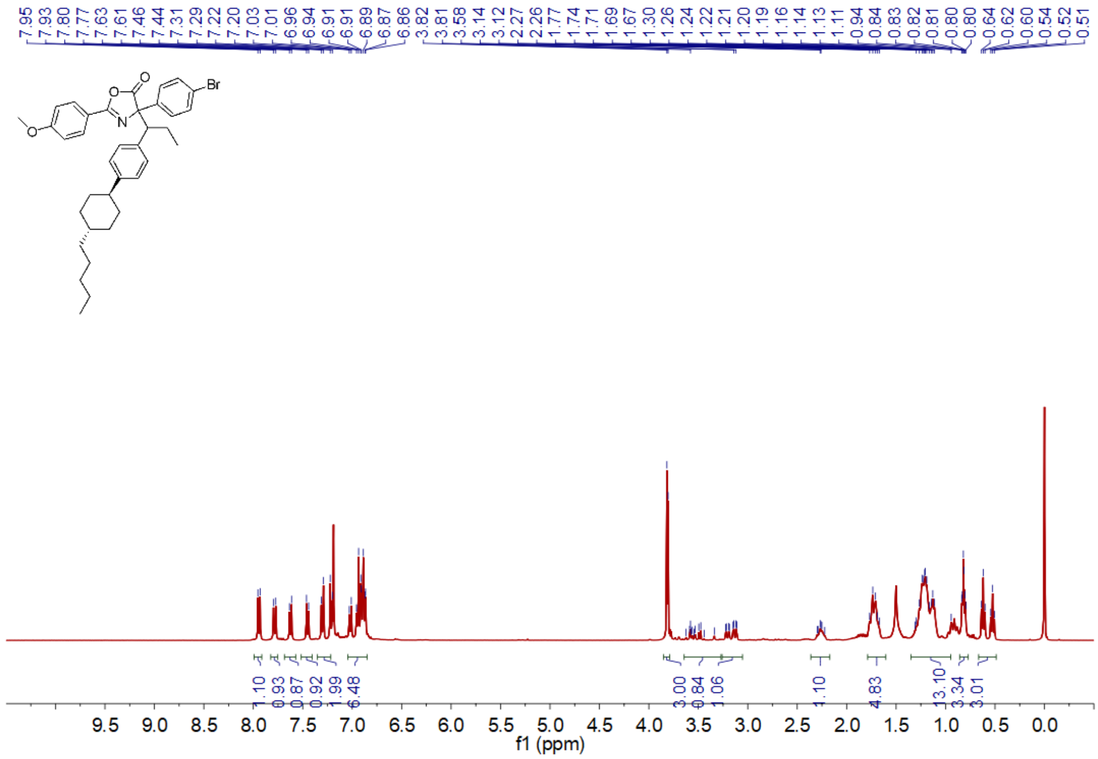

Supplementary Figure 156 ^{1}H NMR (400 MHz, $CDCl_3$, 25 °C) of compound **50**

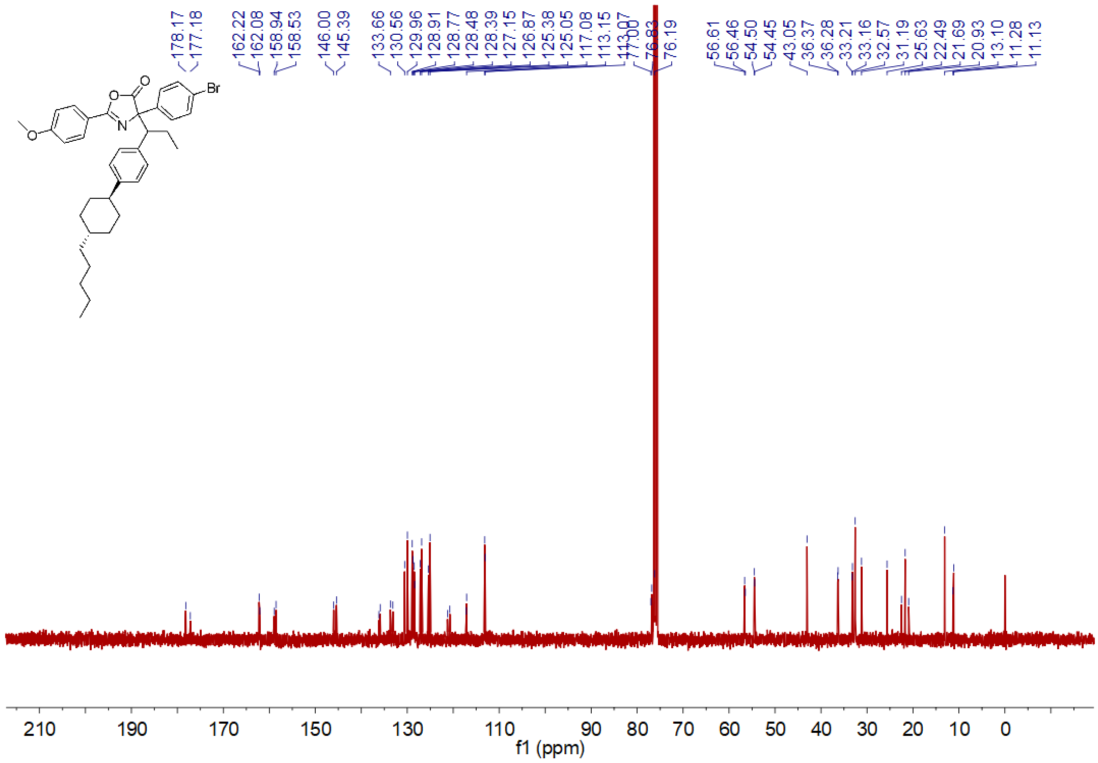

Supplementary Figure 157 ^{13}C NMR (101 MHz, $CDCl_3$, 25 °C) of compound **50**

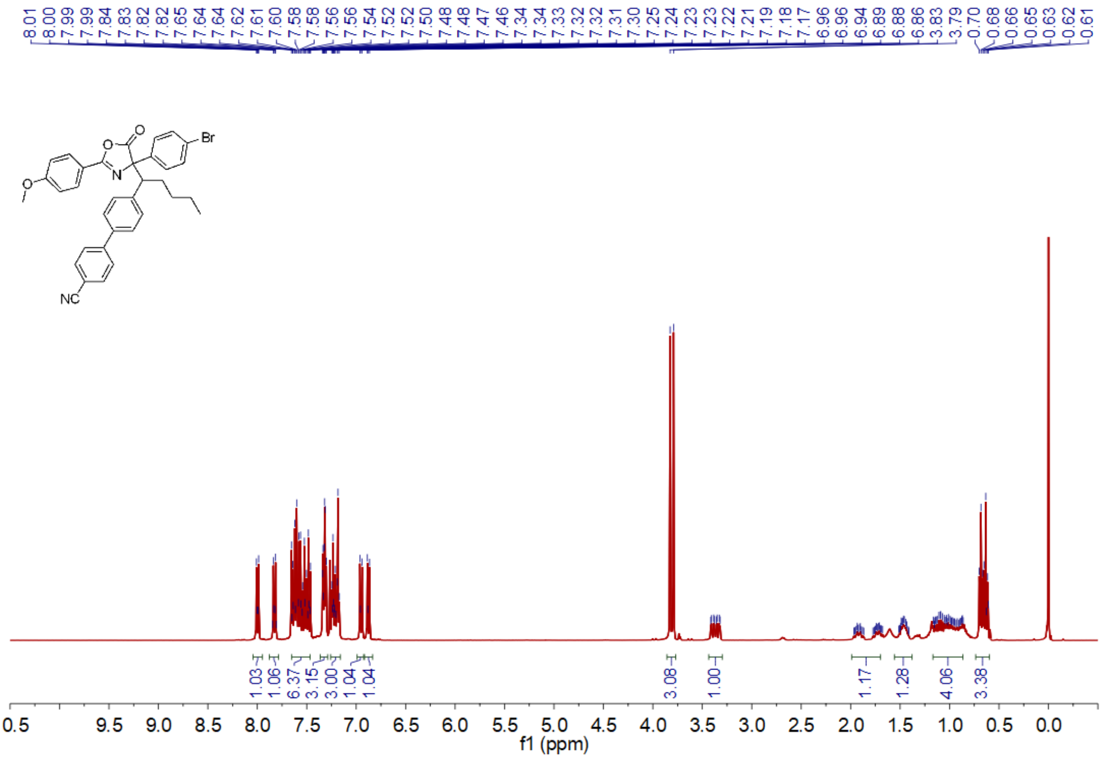

Supplementary Figure 158 ^{1}H NMR (400 MHz, $CDCl_3$, 25 °C) of compound **51**

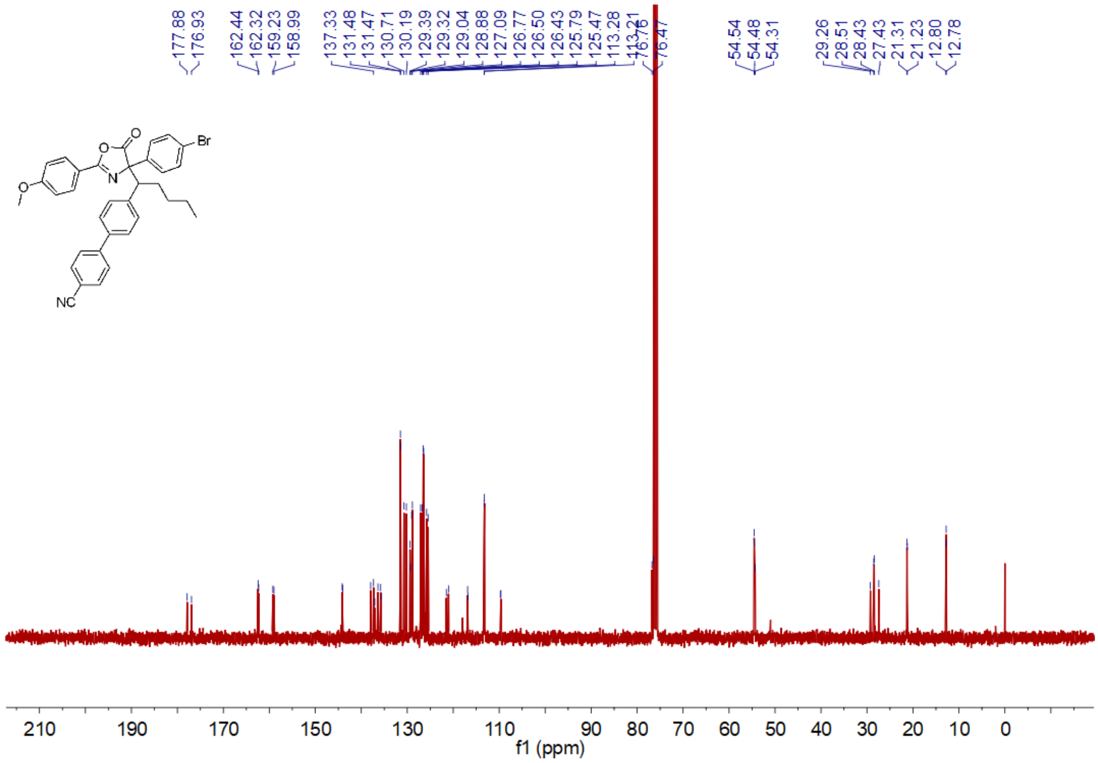

Supplementary Figure 159 ^{13}C NMR (101 MHz, $CDCl_3$, 25 °C) of compound **51**

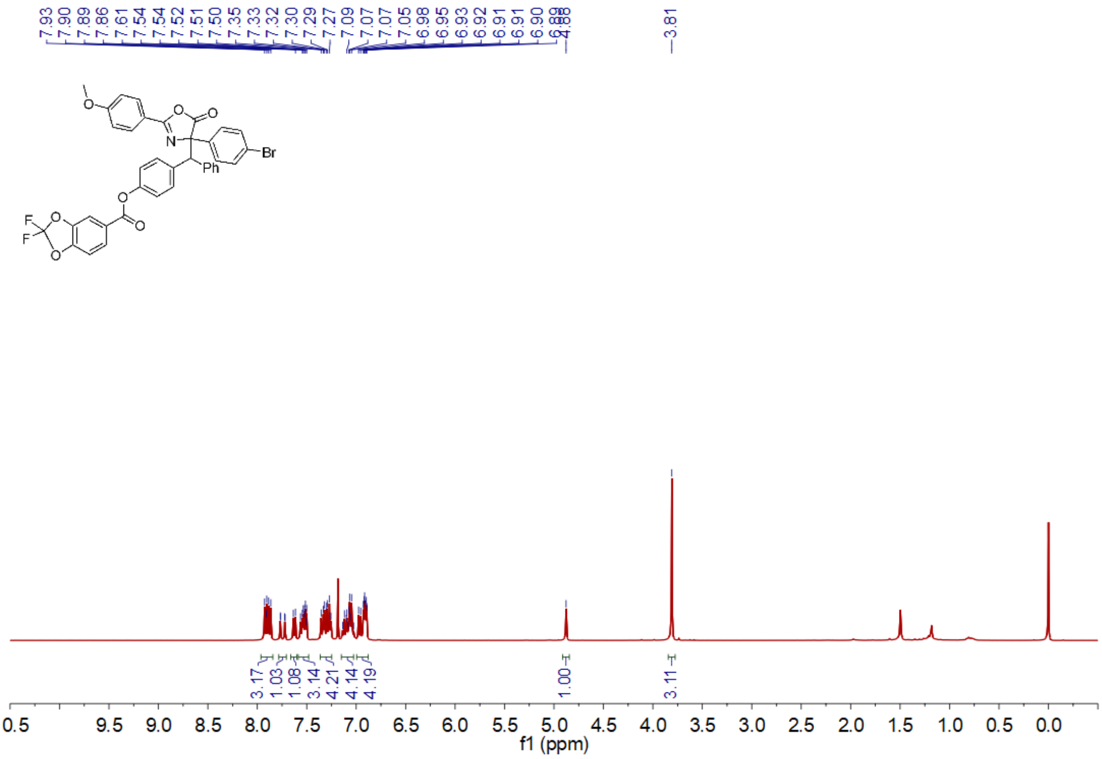

Supplementary Figure 160 ^{1}H NMR (400 MHz, $CDCl_3$, 25 °C) of compound **52**

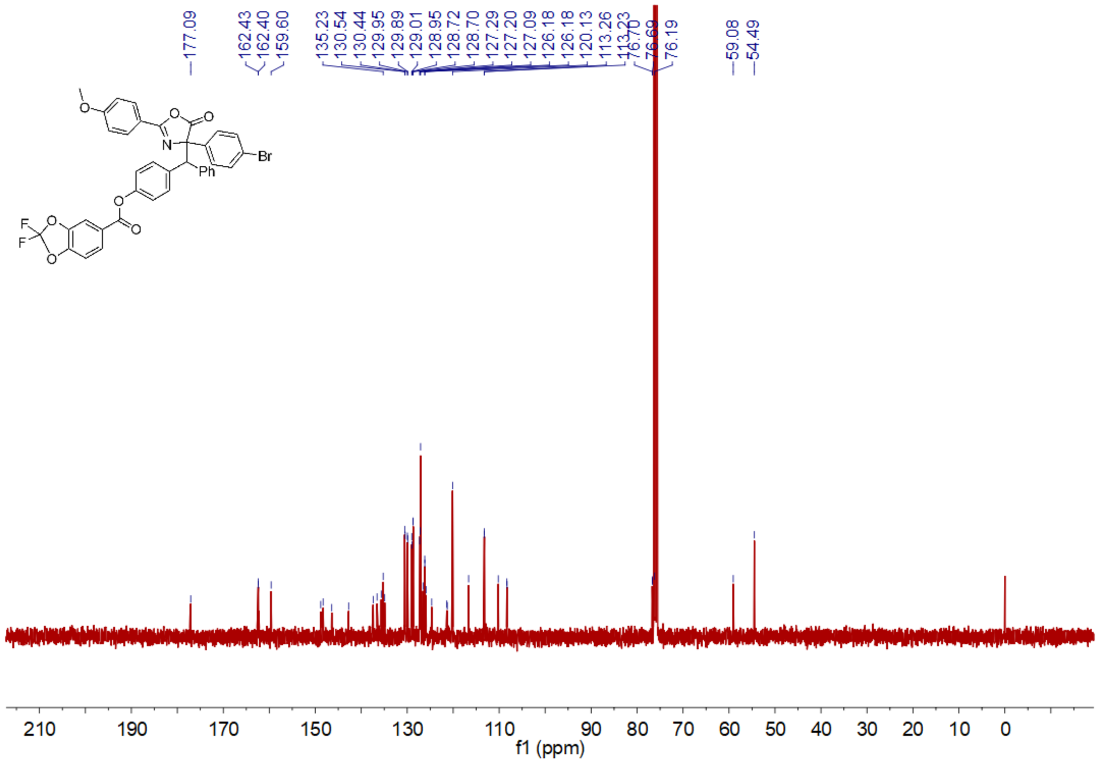

Supplementary Figure 161 ^{13}C NMR (101 MHz, $CDCl_3$, 25 °C) of compound **52**

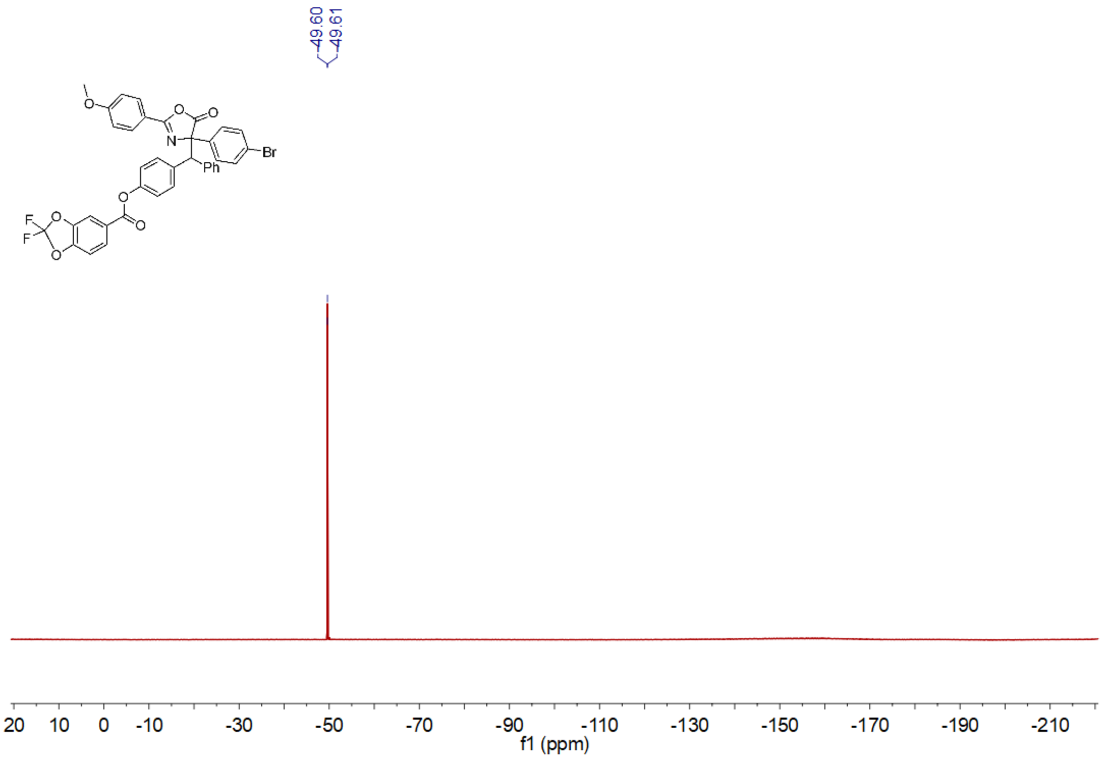

Supplementary Figure 162 ^{19}F NMR (377 MHz, $CDCl_3$, 25 °C) of compound **52**

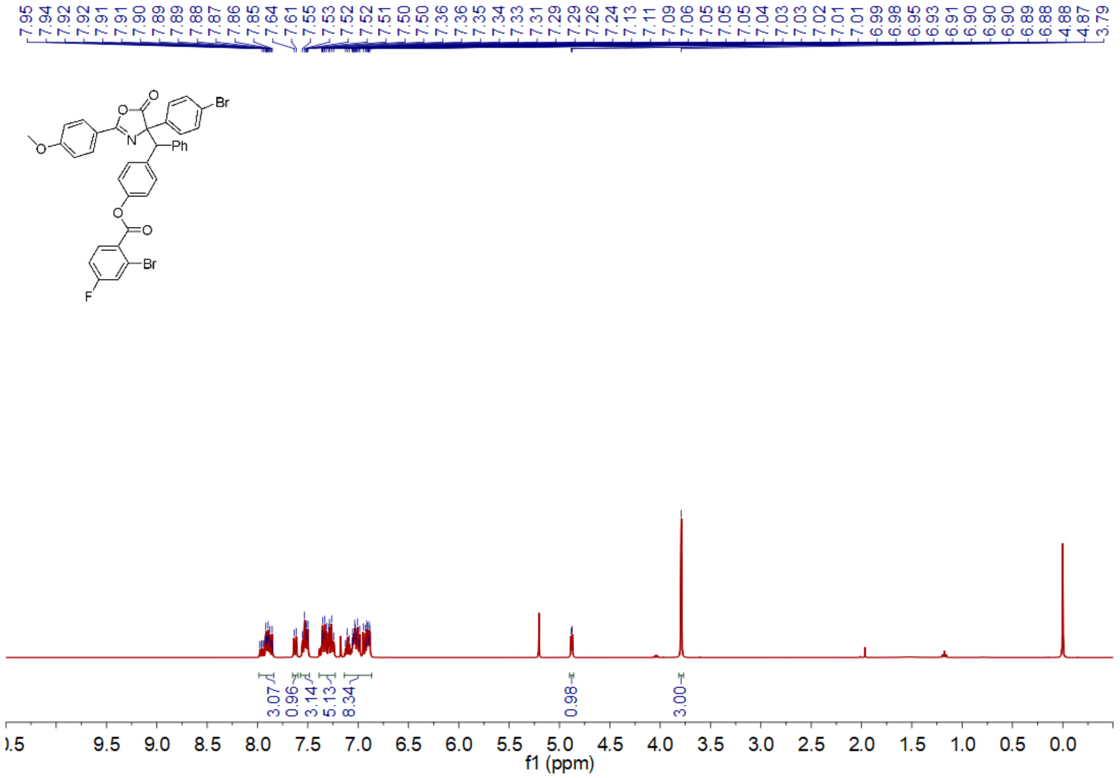

Supplementary Figure 163 ^{1}H NMR (400 MHz, $CDCl_3$, 25 °C) of compound **53**

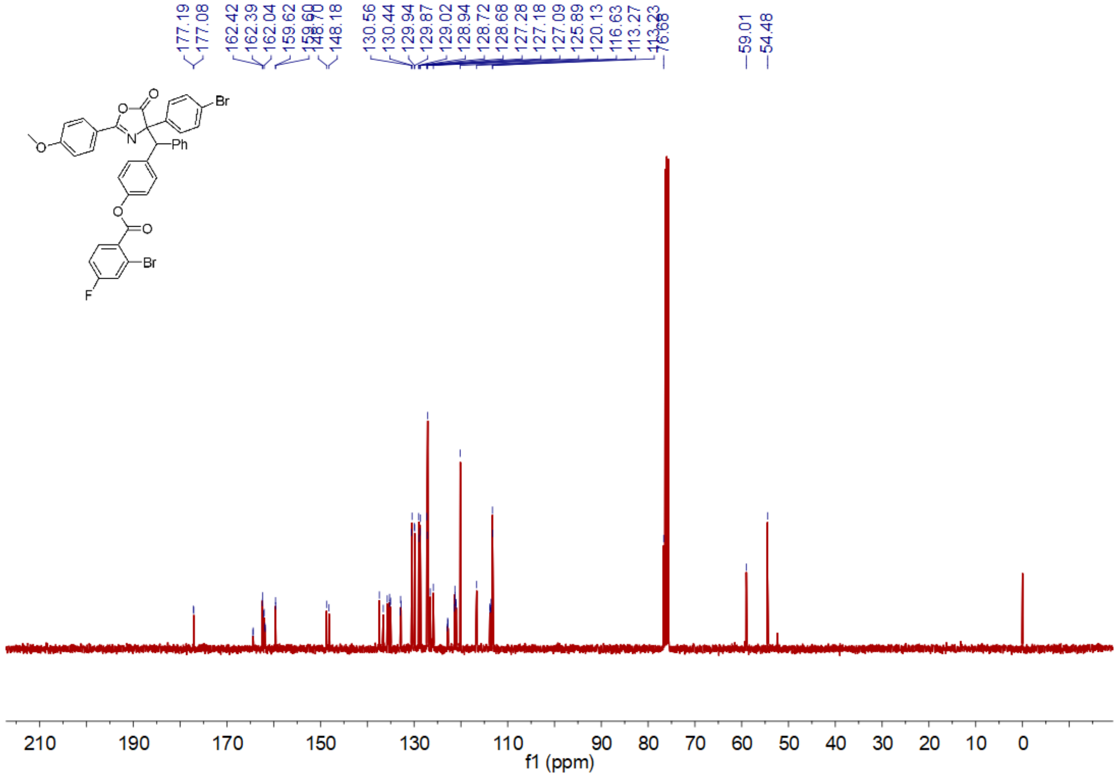

Supplementary Figure 164 ^{13}C NMR (101 MHz, $CDCl_3$, 25 °C) of compound **53**

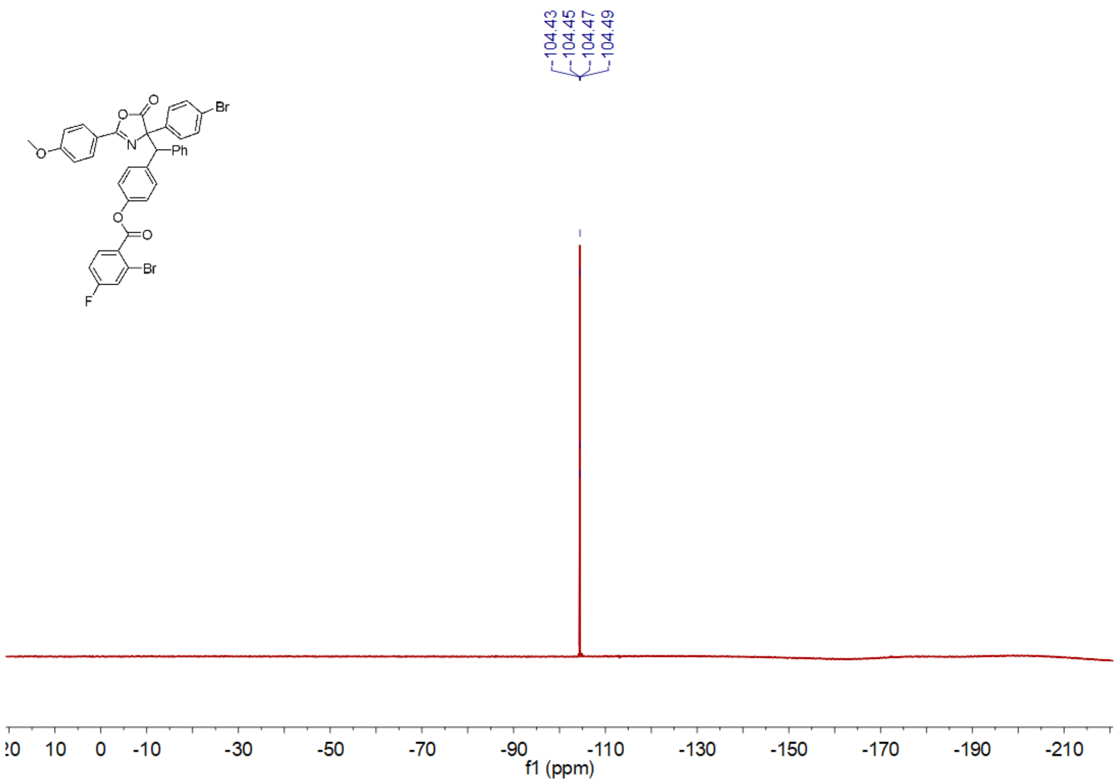

Supplementary Figure 165 ^{19}F NMR (377 MHz, $CDCl_3$, 25 °C) of compound **53**

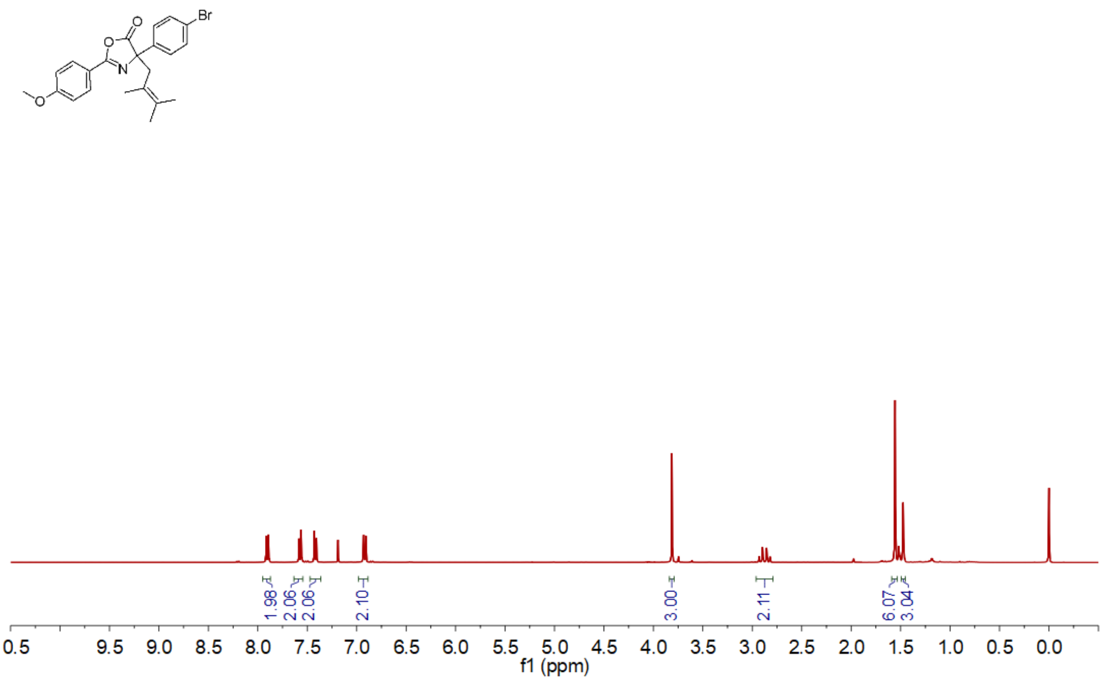

Supplementary Figure 166 ^{1}H NMR (400 MHz, $CDCl_3$, 25 °C) of compound **54**

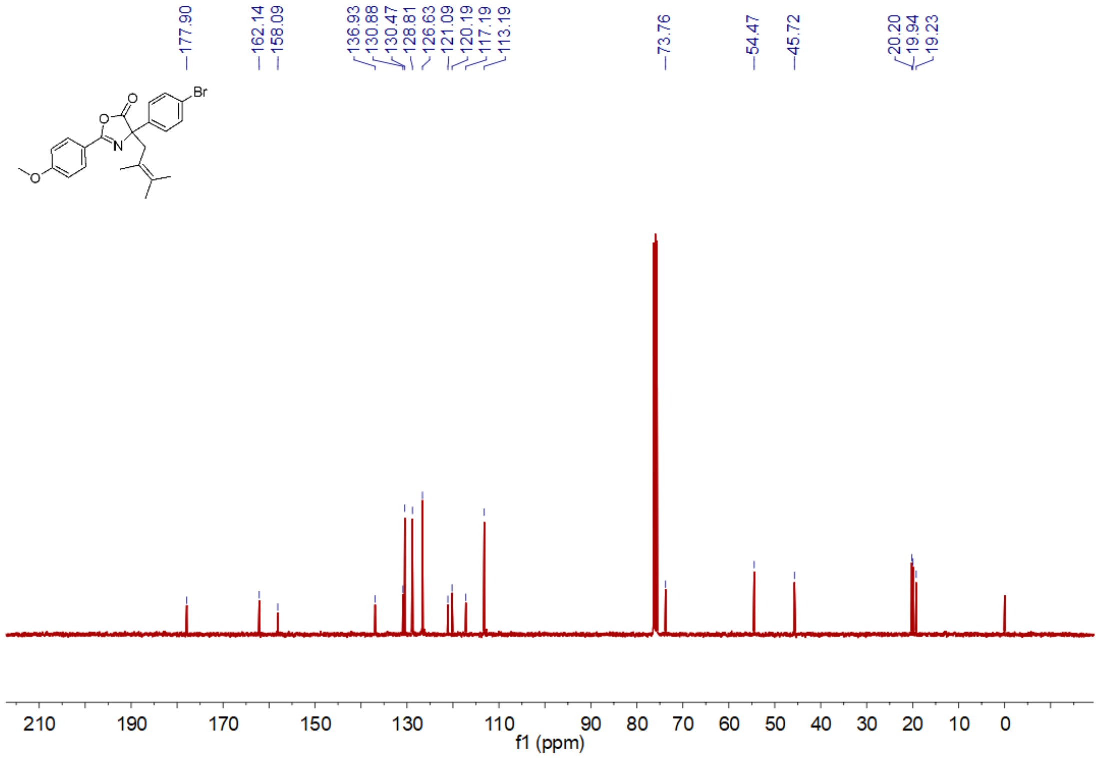

Supplementary Figure 167 ^{13}C NMR (101 MHz, $CDCl_3$, 25 °C) of compound **54**

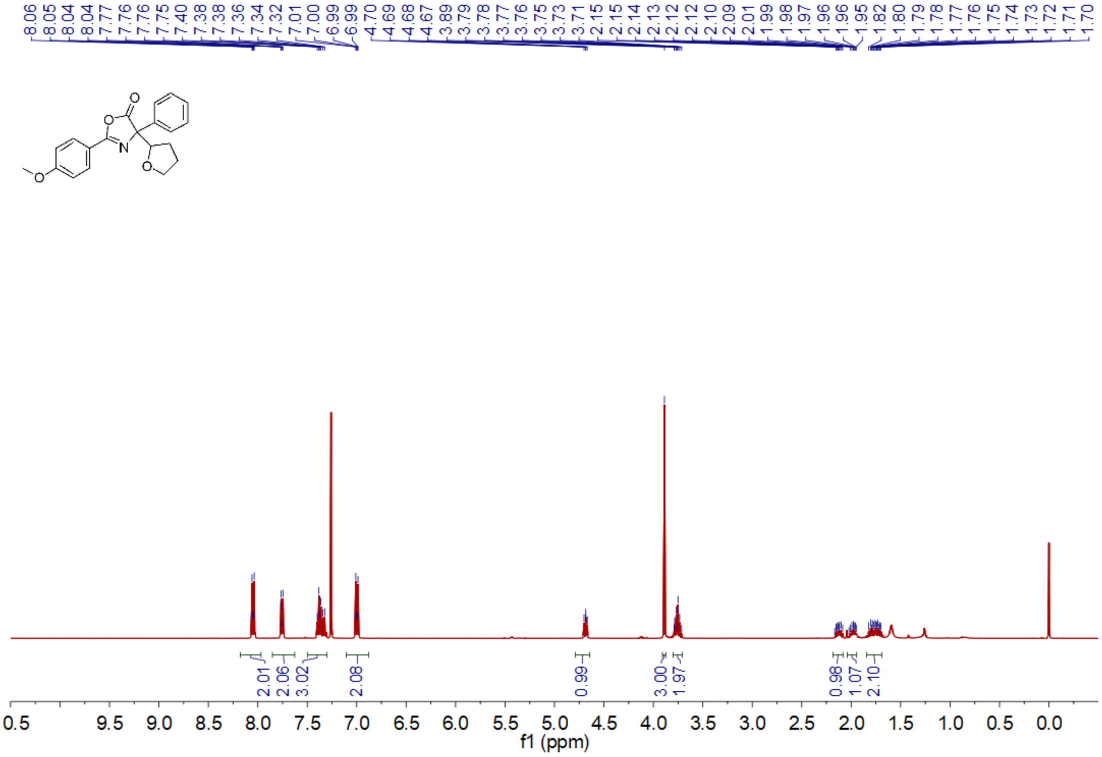

Supplementary Figure 168 ^{1}H NMR (400 MHz, $CDCl_3$, 25 °C) of compound **55**

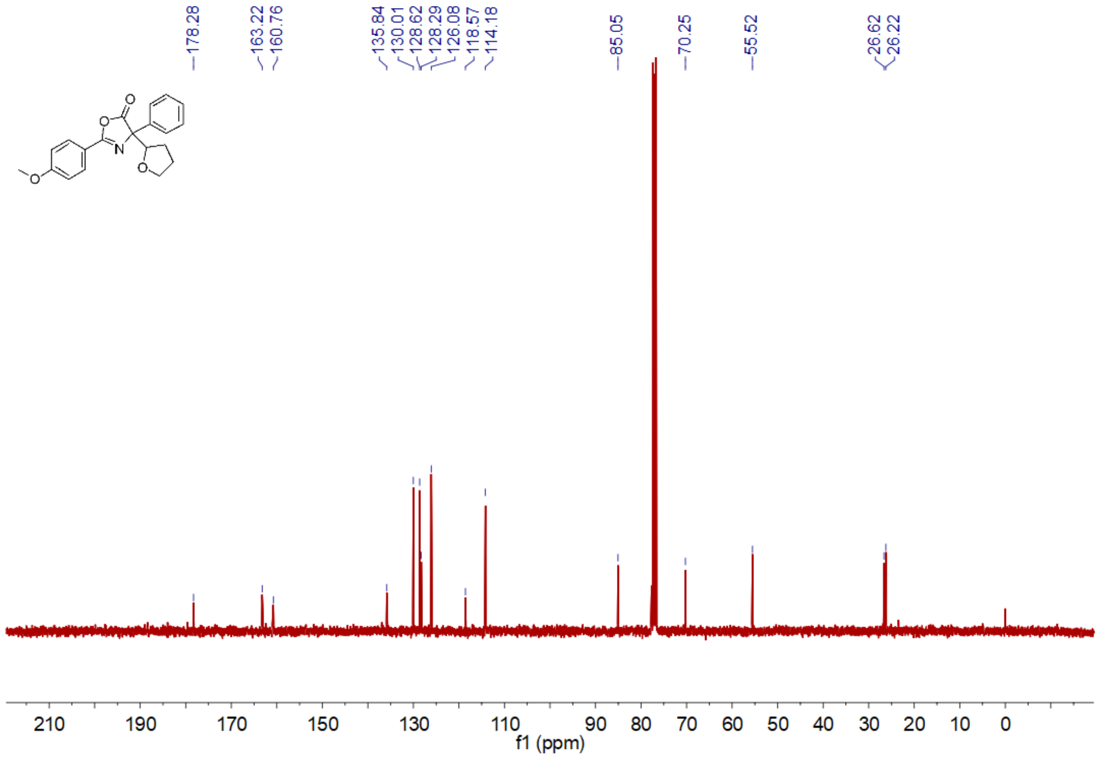

Supplementary Figure 169 ^{13}C NMR (101 MHz, $CDCl_3$, 25 °C) of compound **55**

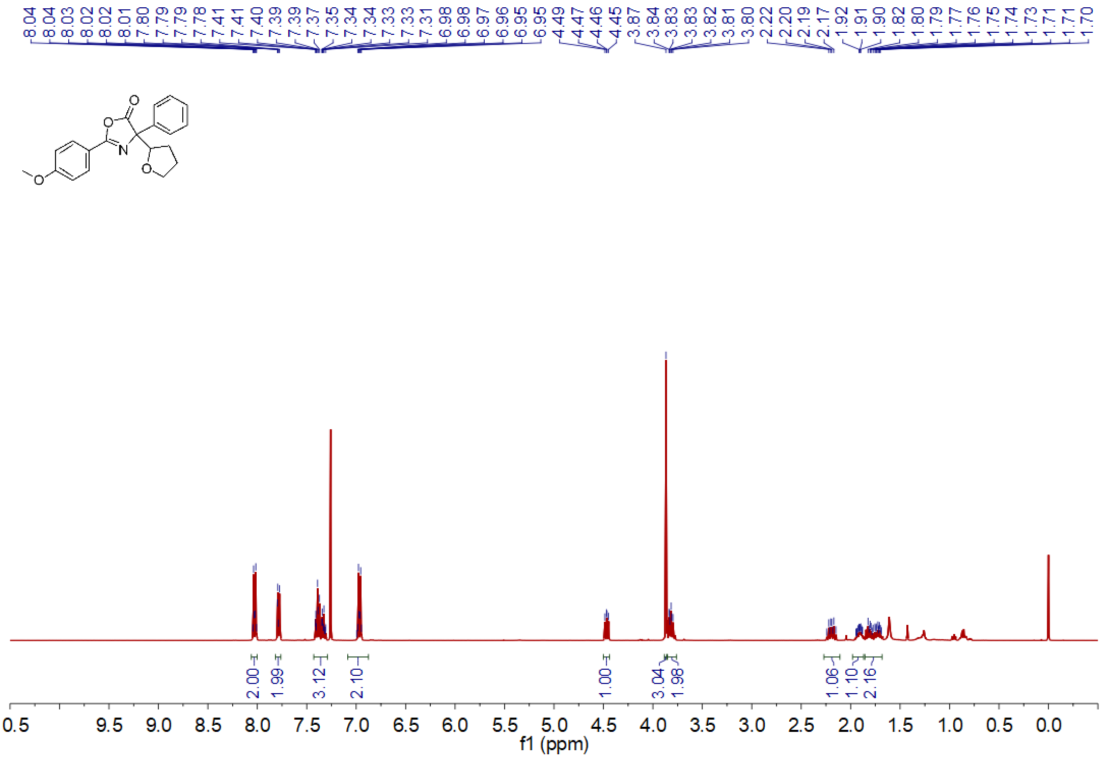

Supplementary Figure 170 ^{1}H NMR (400 MHz, $CDCl_3$, 25 °C) of compound **55**

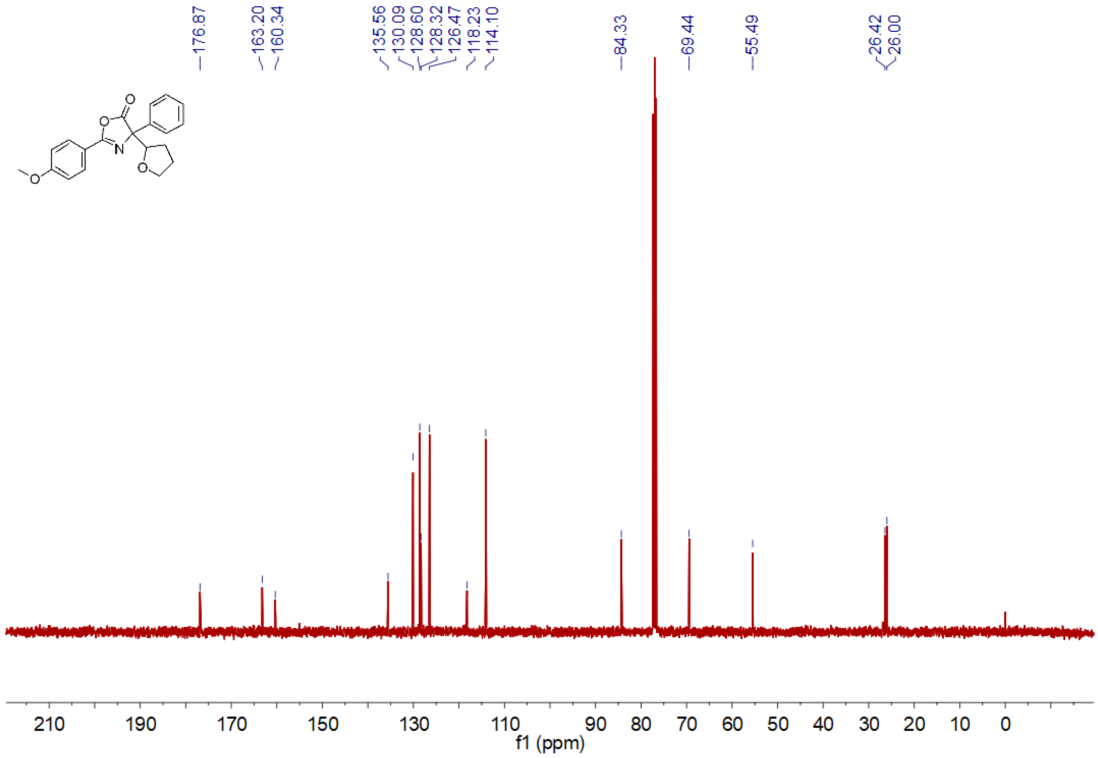

Supplementary Figure 171 ^{13}C NMR (101 MHz, $CDCl_3$, 25 °C) of compound **55**

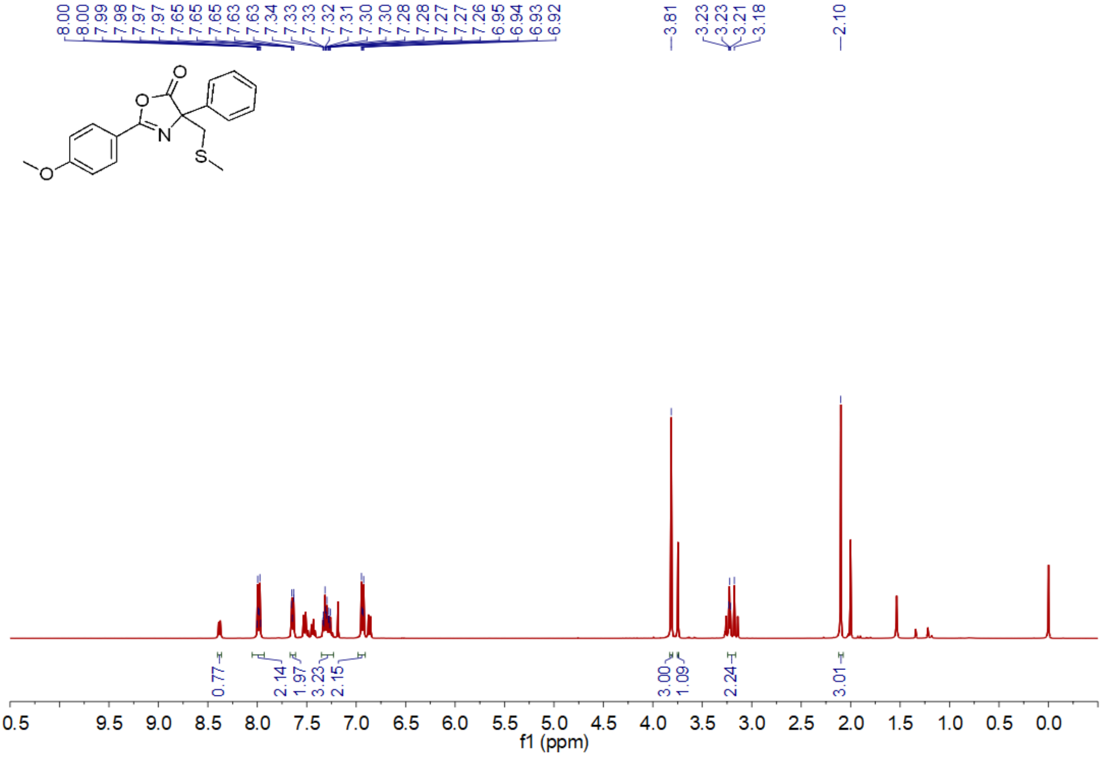

Supplementary Figure 172 ^{1}H NMR (400 MHz, $CDCl_3$, 25 °C) of compound **56**

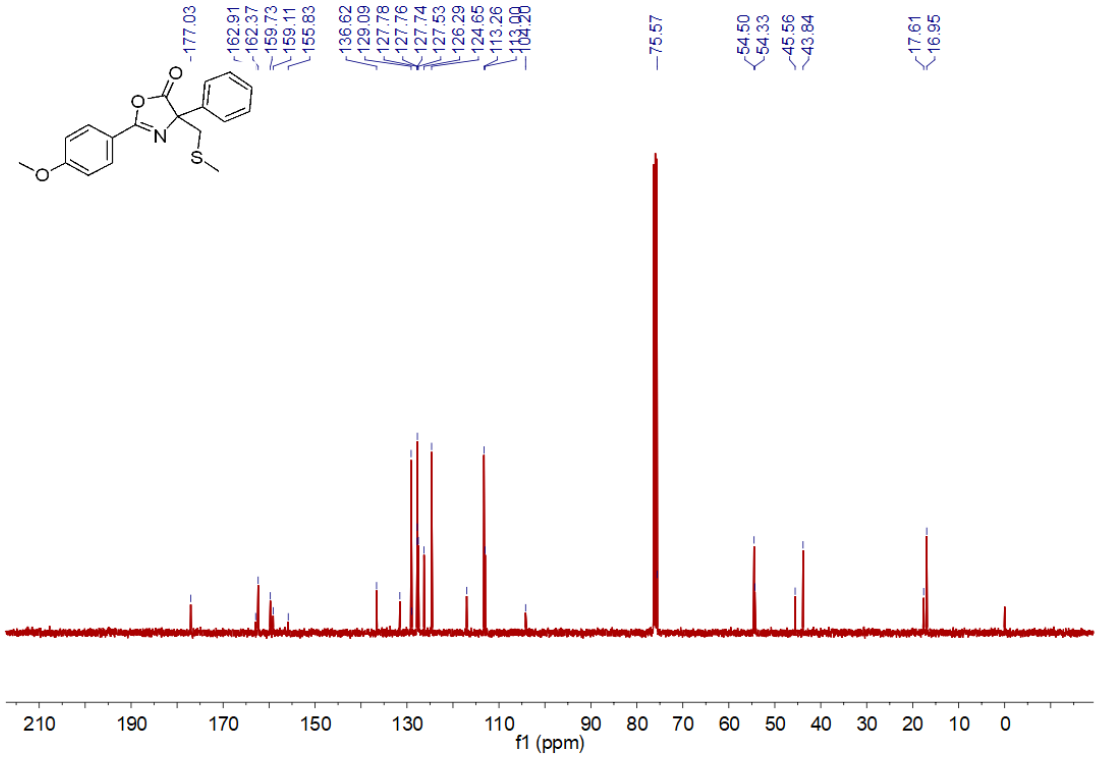

Supplementary Figure 173 ^{13}C NMR (101 MHz, $CDCl_3$, 25 °C) of compound **56**

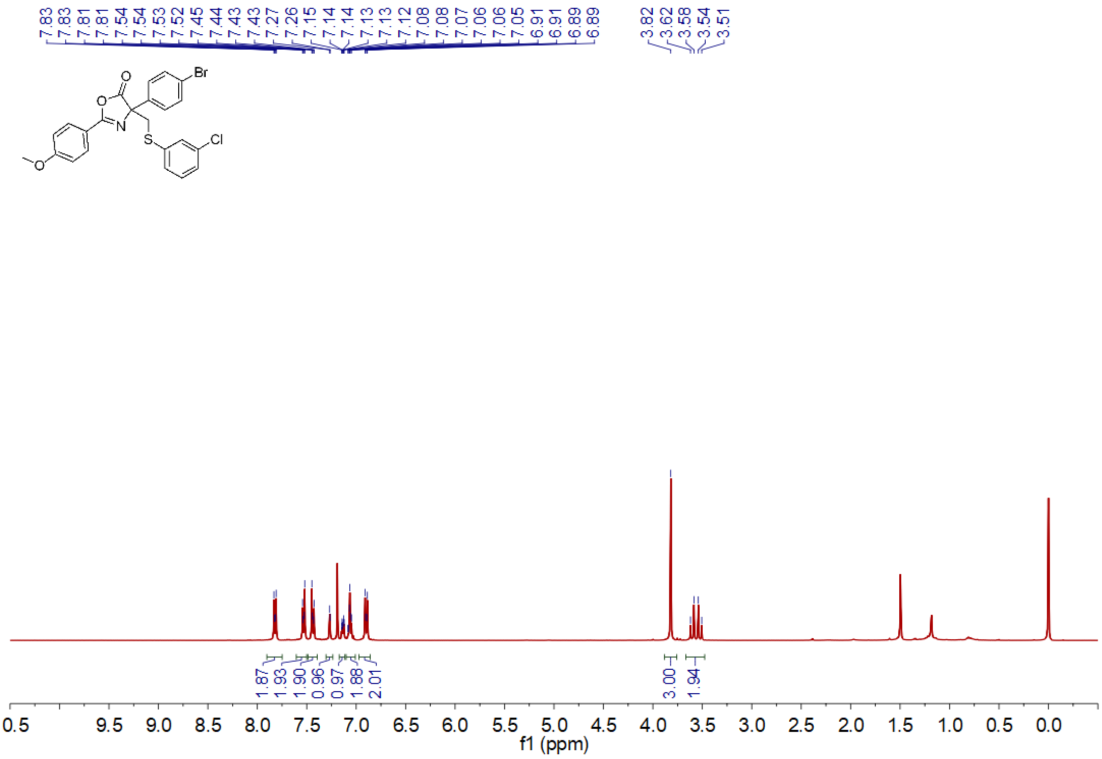

Supplementary Figure 174 ^{1}H NMR (400 MHz, $CDCl_3$, 25 °C) of compound **57**

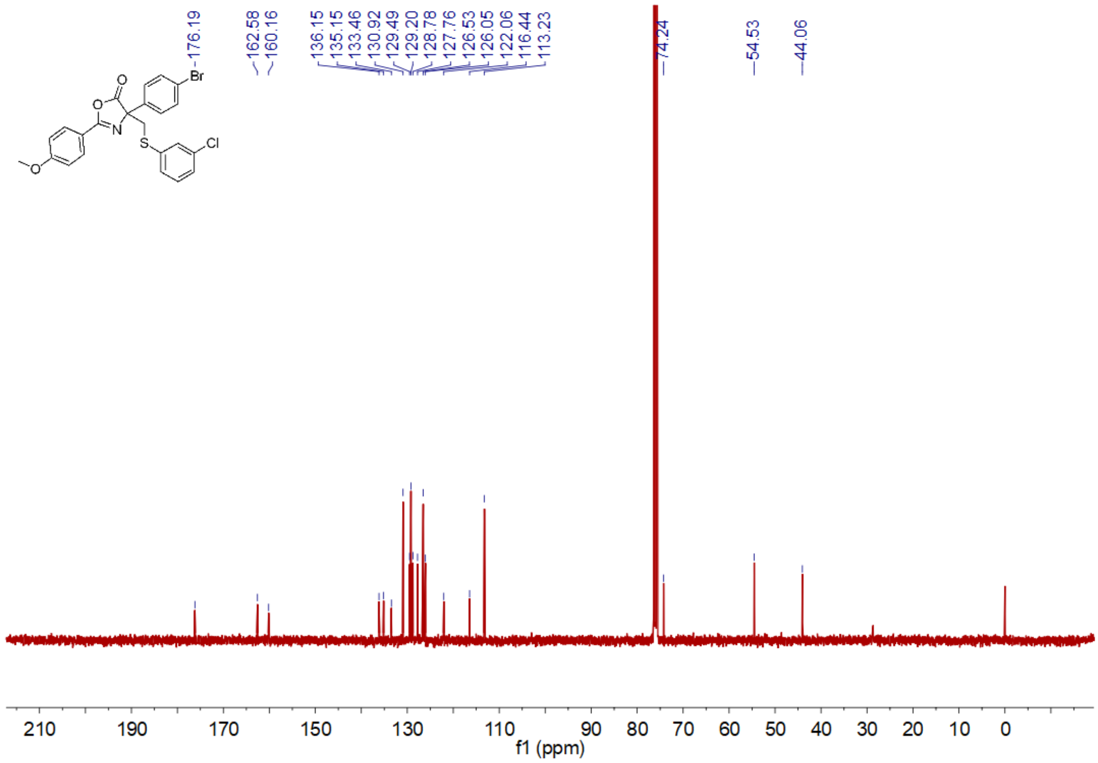

Supplementary Figure 175 ^{13}C NMR (101 MHz, $CDCl_3$, 25 °C) of compound **57**

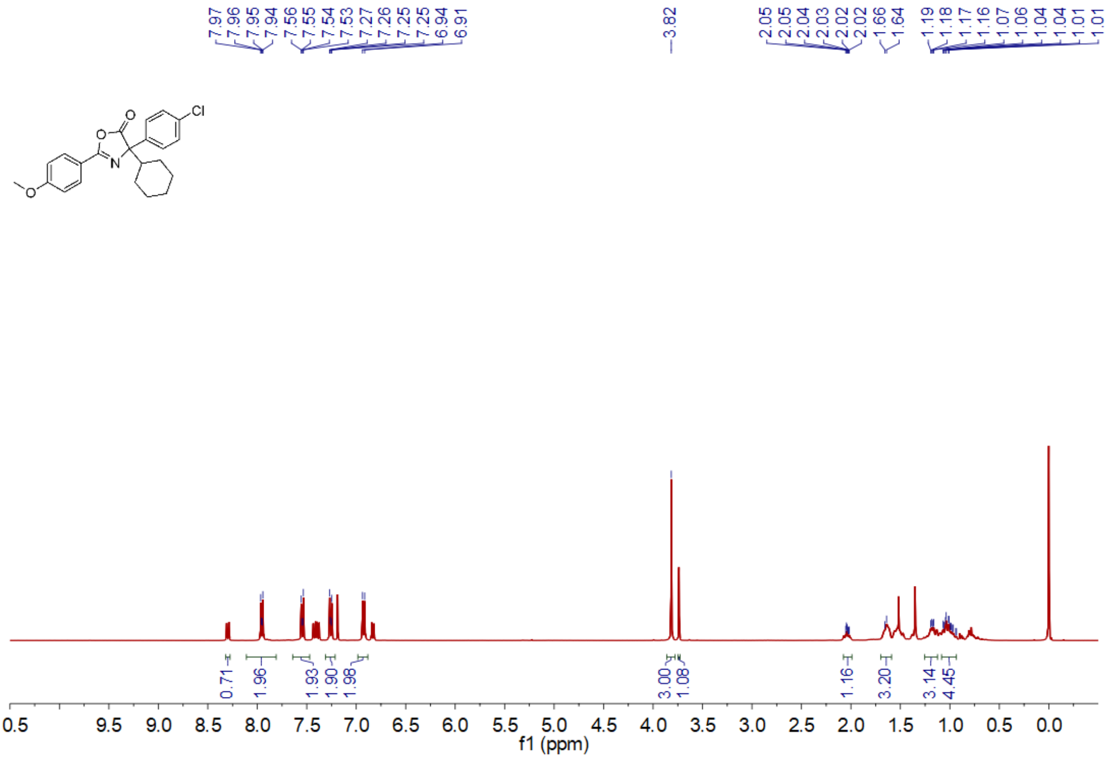

Supplementary Figure 176 ^{1}H NMR (400 MHz, $CDCl_3$, 25 °C) of compound **58**

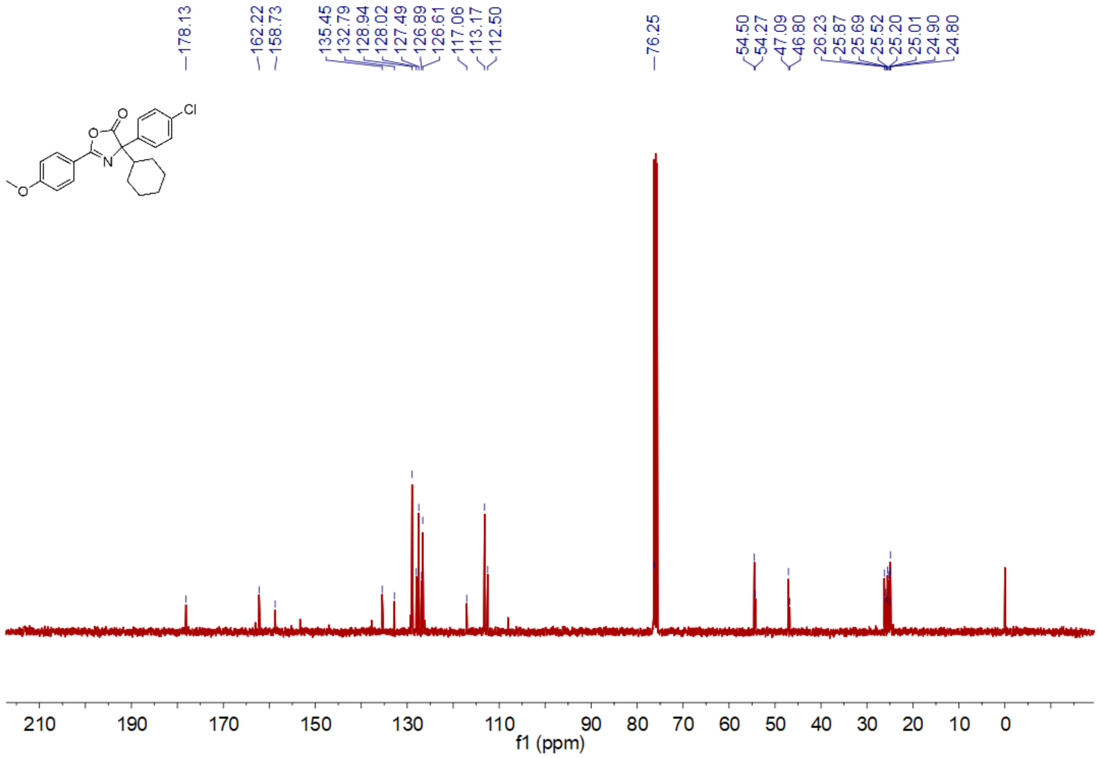

Supplementary Figure 177 ^{13}C NMR (101 MHz, $CDCl_3$, 25 °C) of compound **58**

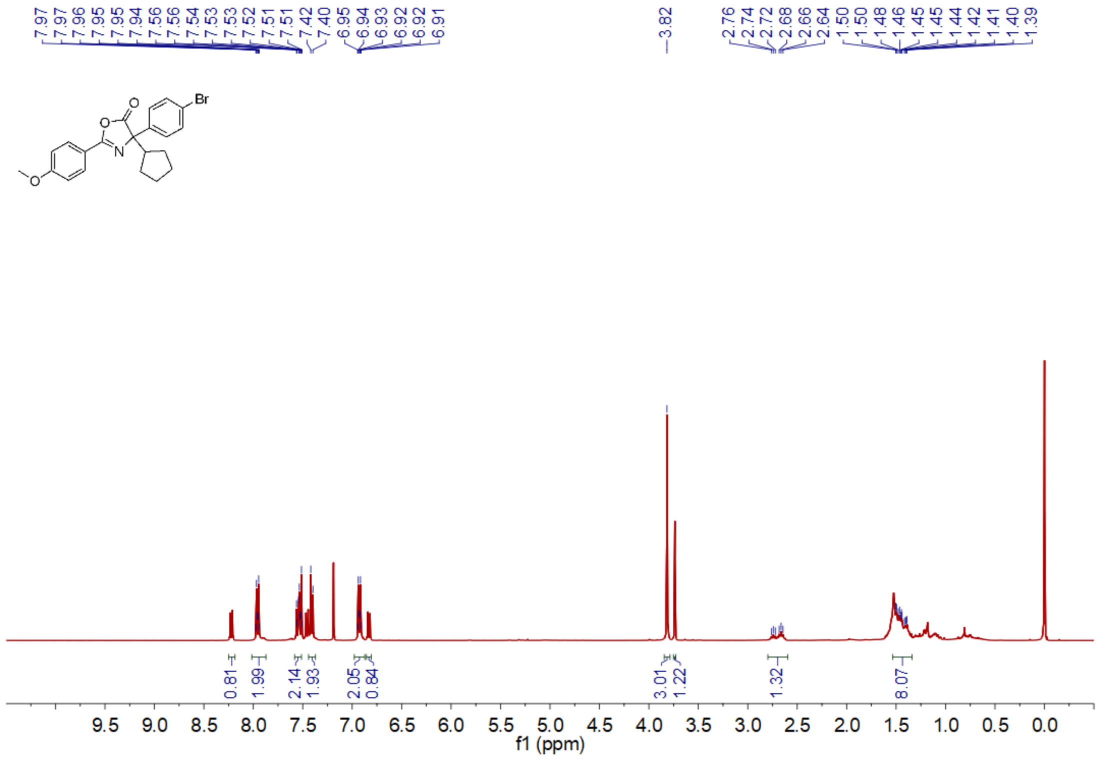

Supplementary Figure 178 ^{1}H NMR (400 MHz, $CDCl_3$, 25 °C) of compound **59**

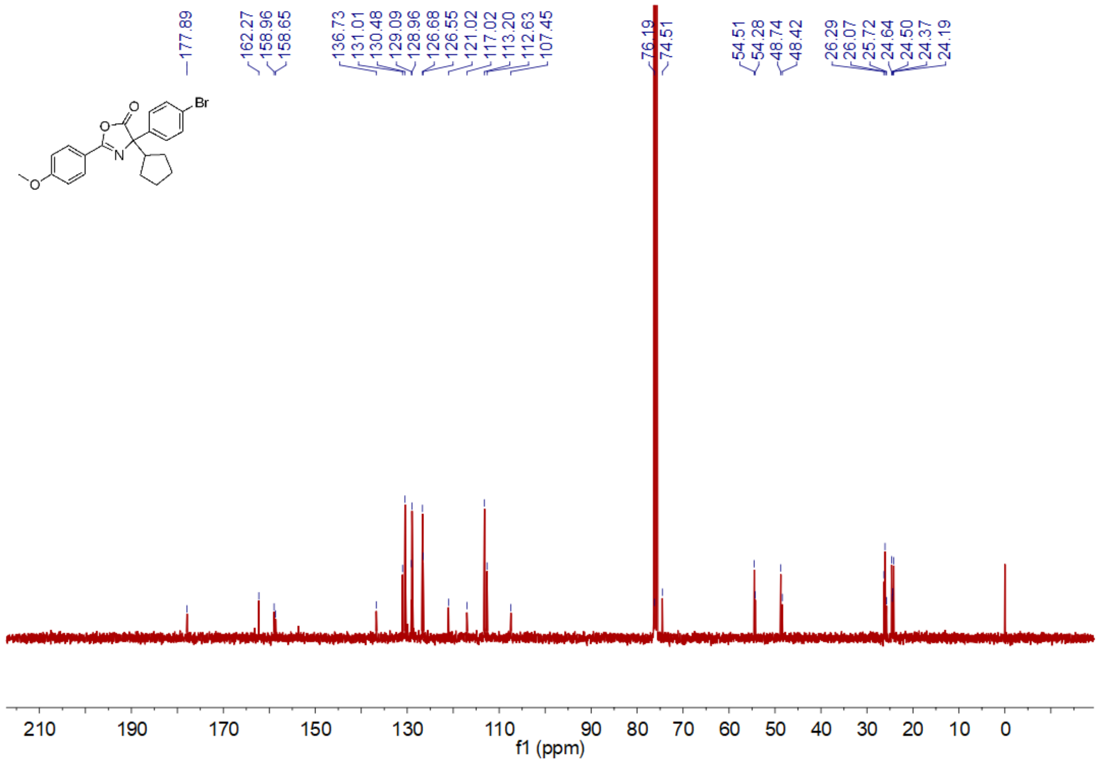

Supplementary Figure 179 ^{13}C NMR (101 MHz, $CDCl_3$, 25 °C) of compound **59**

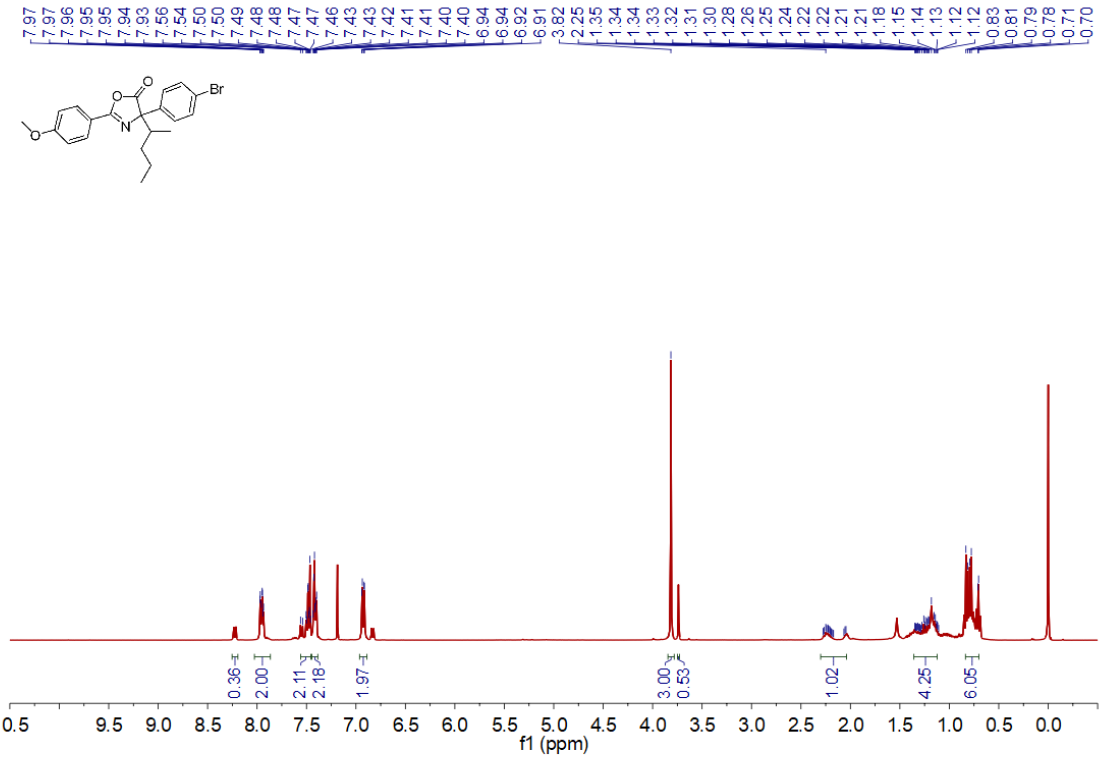

Supplementary Figure 180 ^{1}H NMR (400 MHz, $CDCl_3$, 25 °C) of compound **60**

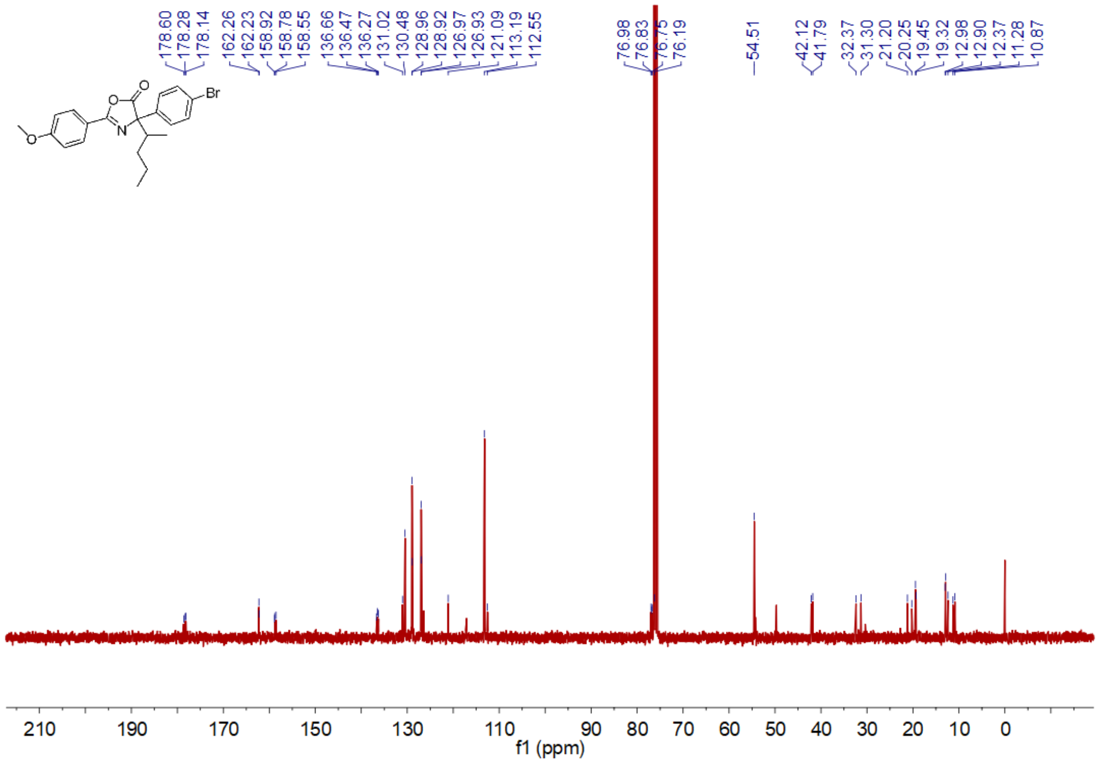

Supplementary Figure 181 ^{13}C NMR (101 MHz, $CDCl_3$, 25 °C) of compound **60**

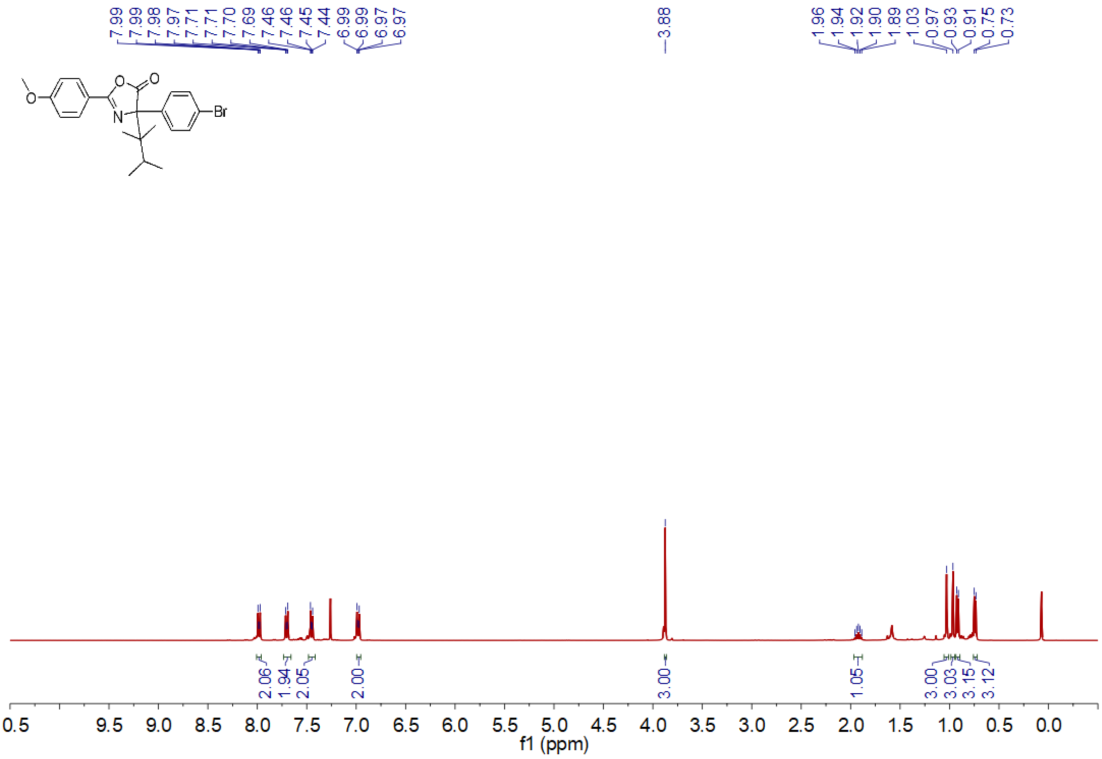

Supplementary Figure 182 ^{1}H NMR (400 MHz, $CDCl_3$, 25 °C) of compound **61**

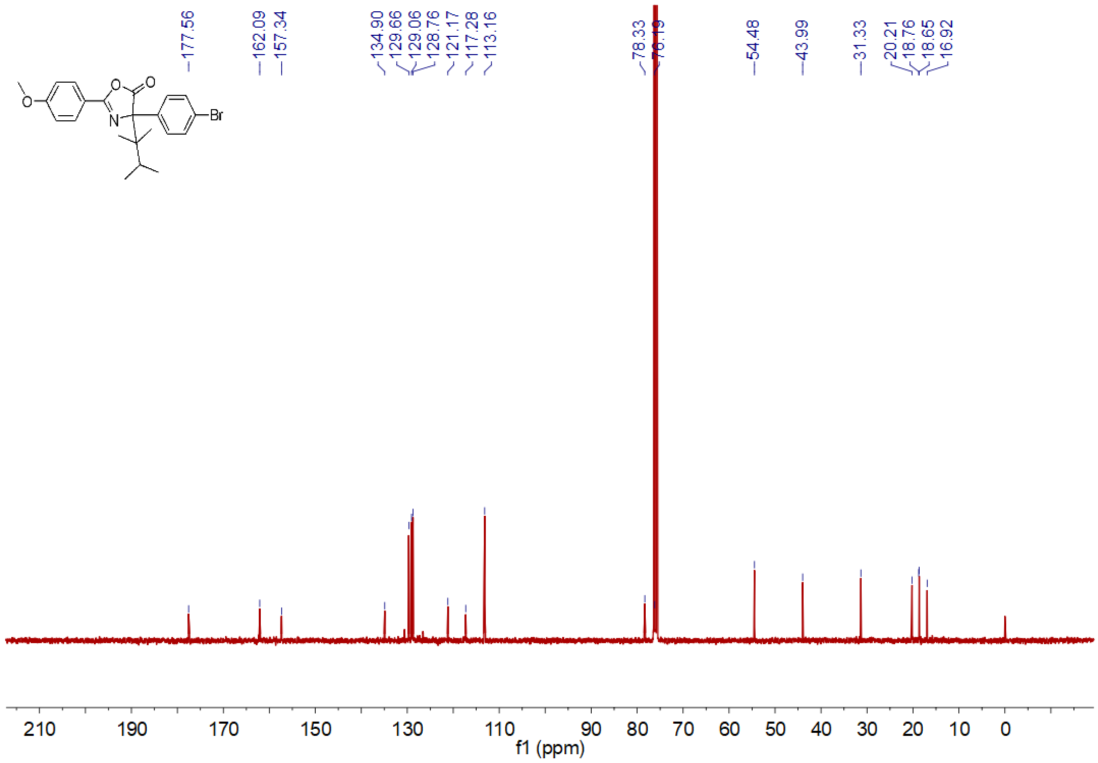

Supplementary Figure 183 ^{13}C NMR (101 MHz, $CDCl_3$, 25 °C) of compound **61**

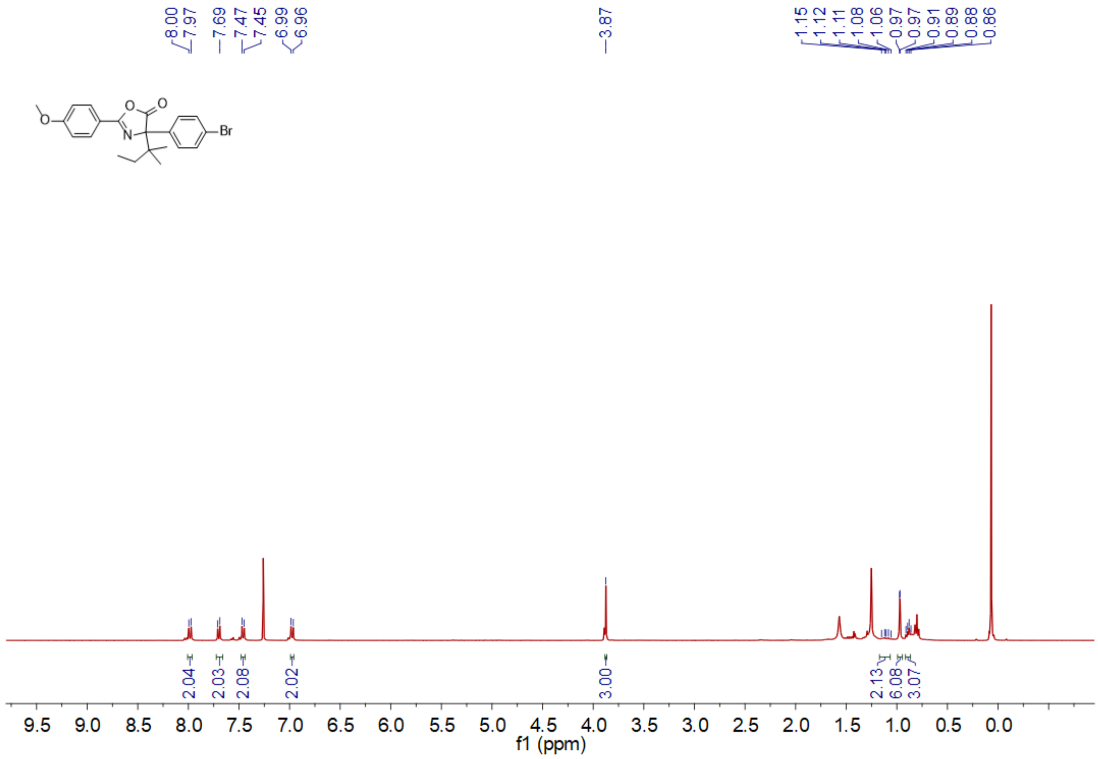

Supplementary Figure 184 ^{1}H NMR (400 MHz, $CDCl_3$, 25 °C) of compound **62**

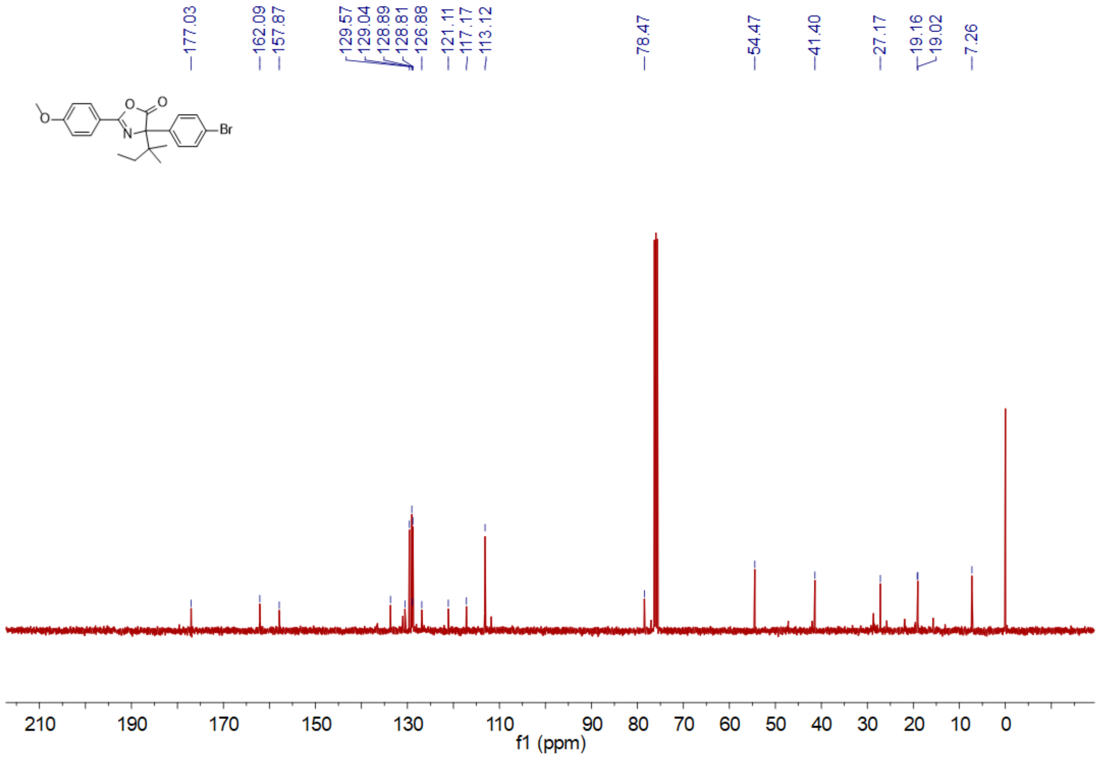

Supplementary Figure 185 ^{13}C NMR (101 MHz, $CDCl_3$, 25 °C) of compound **62**

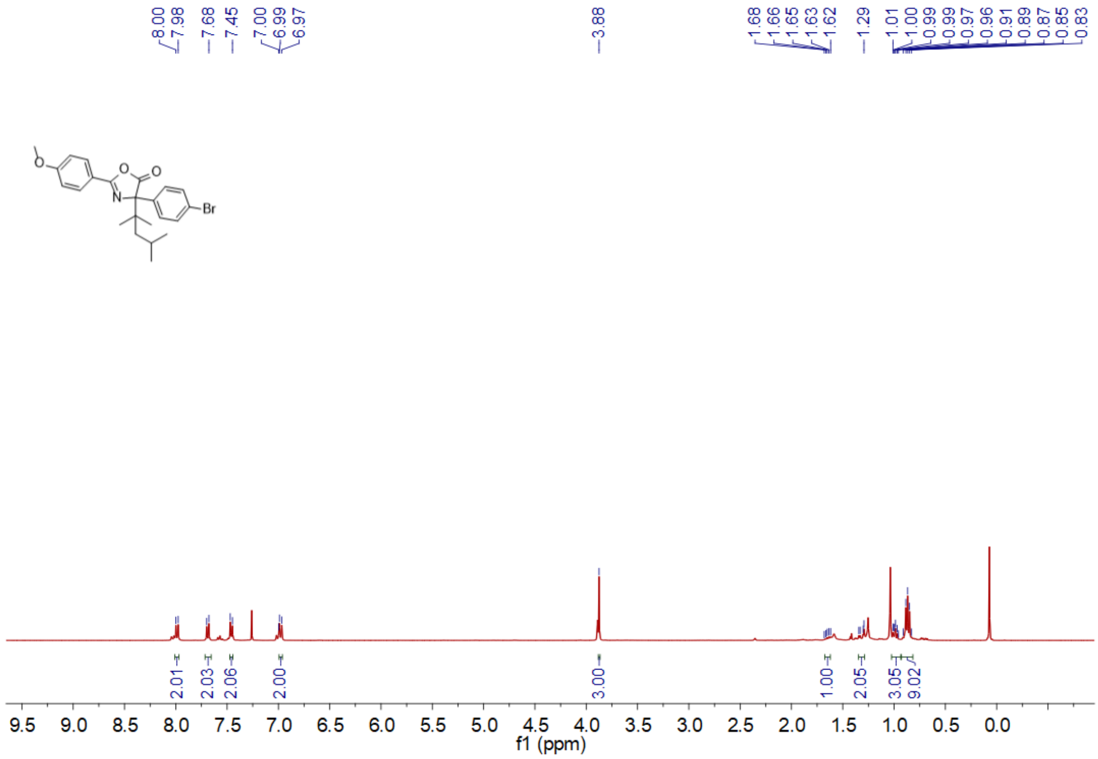

Supplementary Figure 186 ^{1}H NMR (400 MHz, $CDCl_3$, 25 °C) of compound **63**

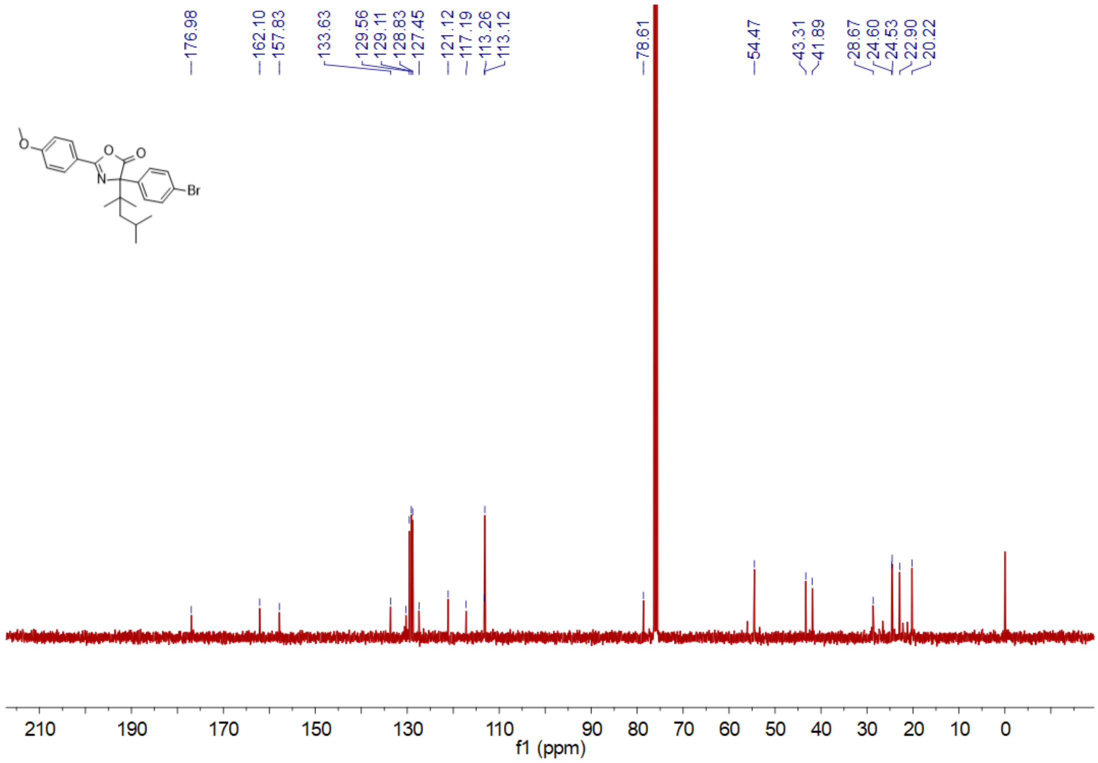

Supplementary Figure 187 ^{13}C NMR (101 MHz, $CDCl_3$, 25 °C) of compound **63**

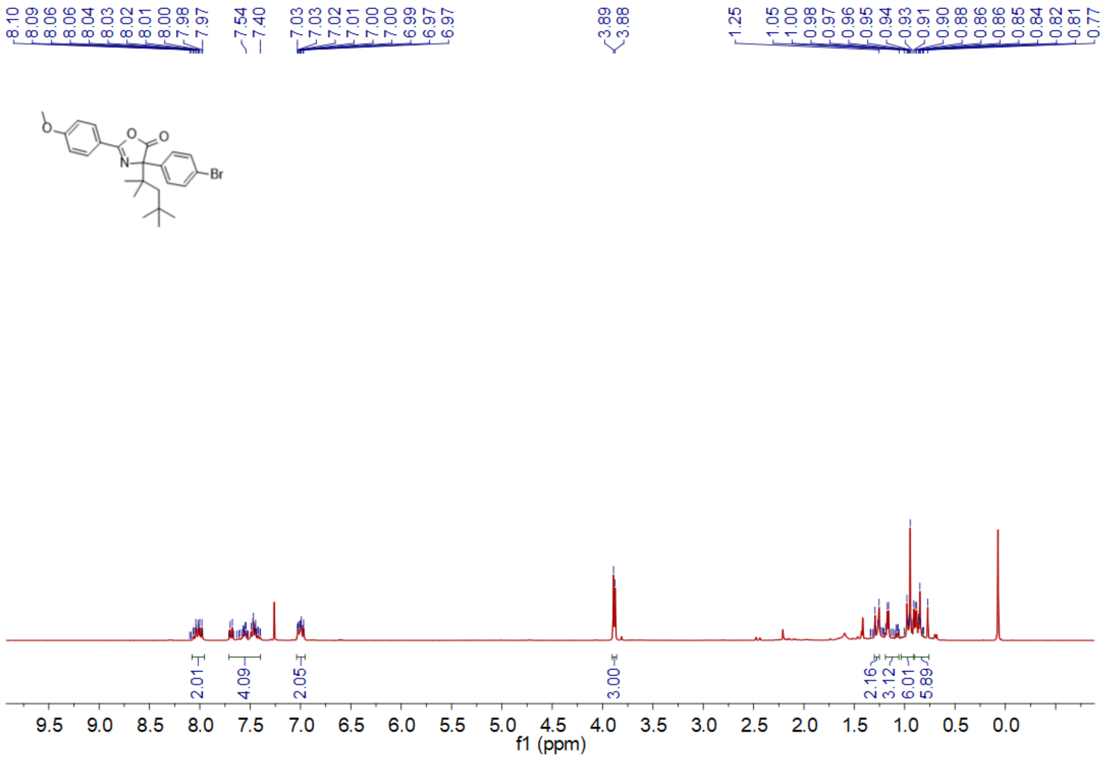

Supplementary Figure 188 ^{1}H NMR (400 MHz, $CDCl_3$, 25 °C) of compound **64**

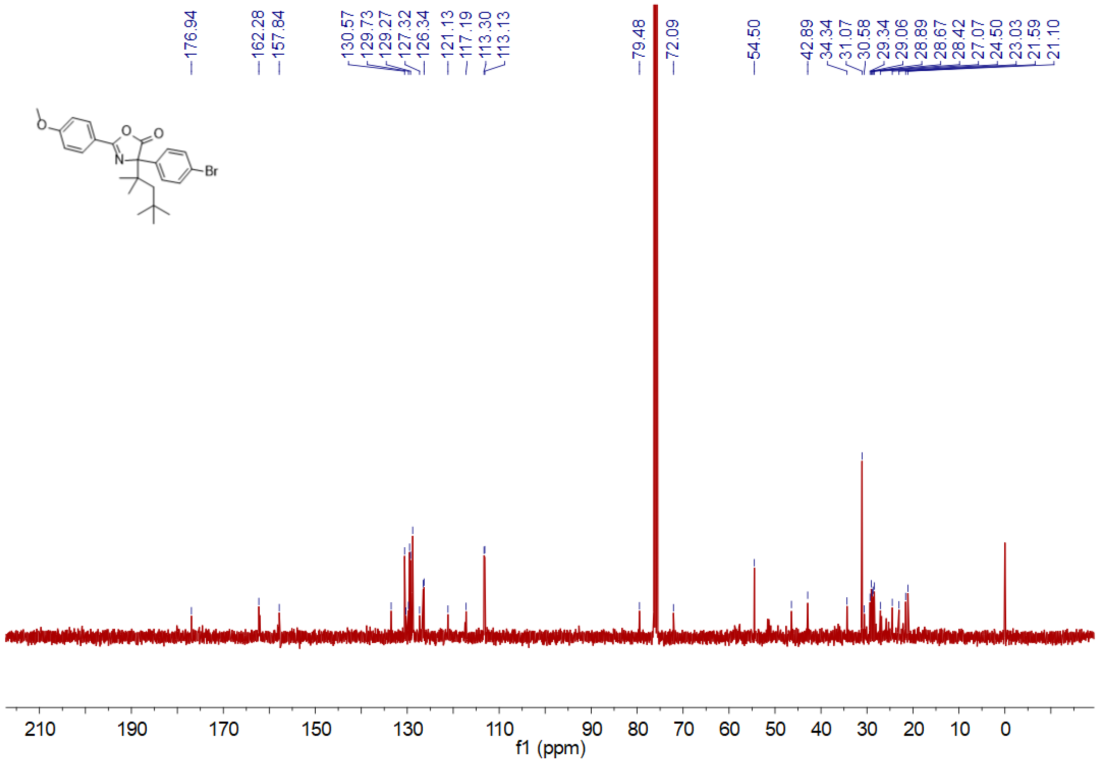

Supplementary Figure 189 ^{13}C NMR (101 MHz, $CDCl_3$, 25 °C) of compound **64**

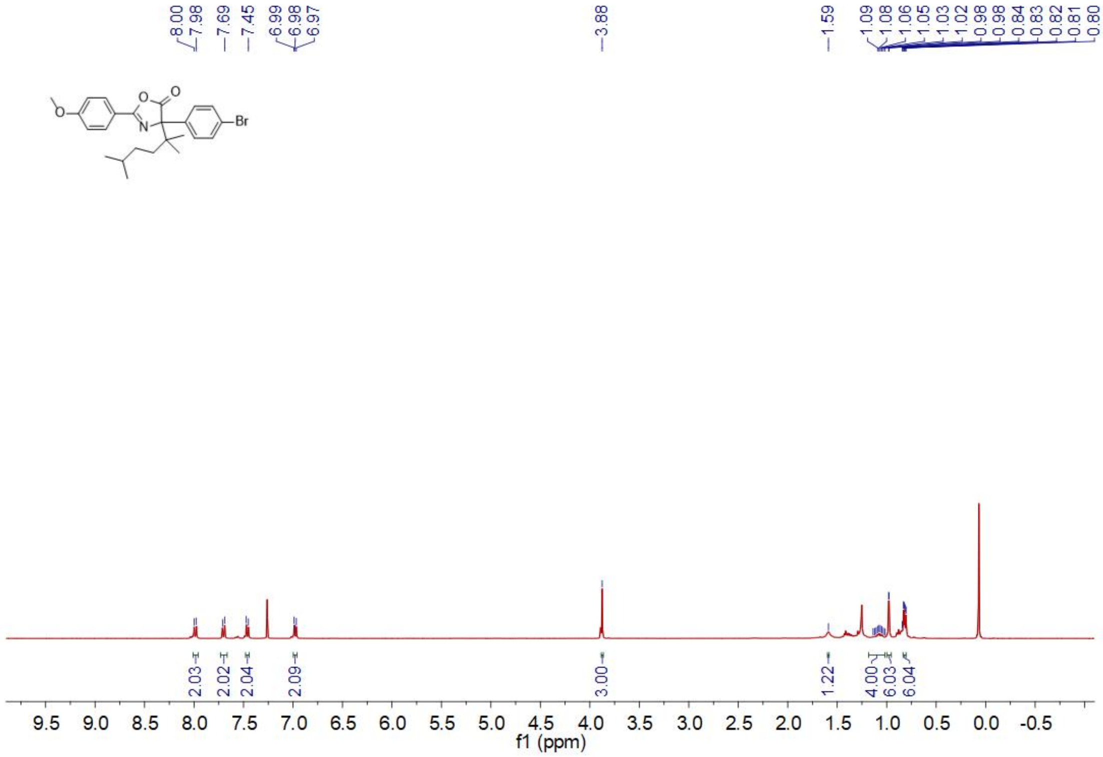

Supplementary Figure 190 ^{1}H NMR (400 MHz, $CDCl_3$, 25 °C) of compound **65**

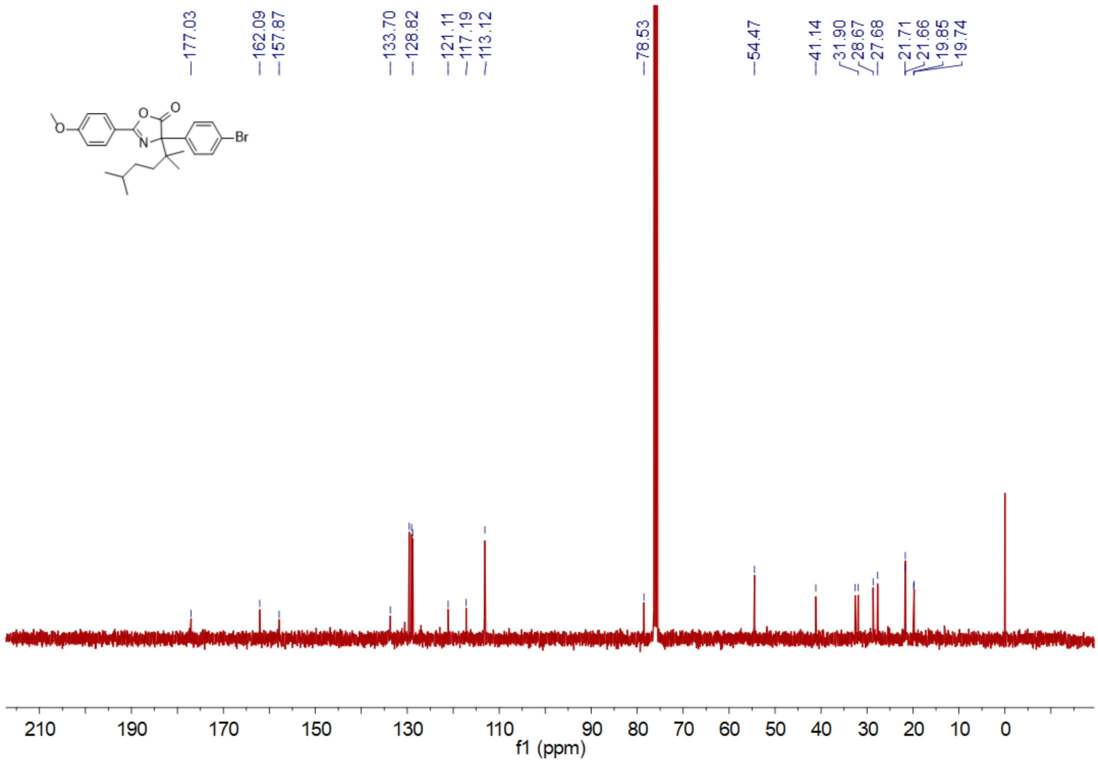

Supplementary Figure 191 ^{13}C NMR (101 MHz, $CDCl_3$, 25 °C) of compound **65**

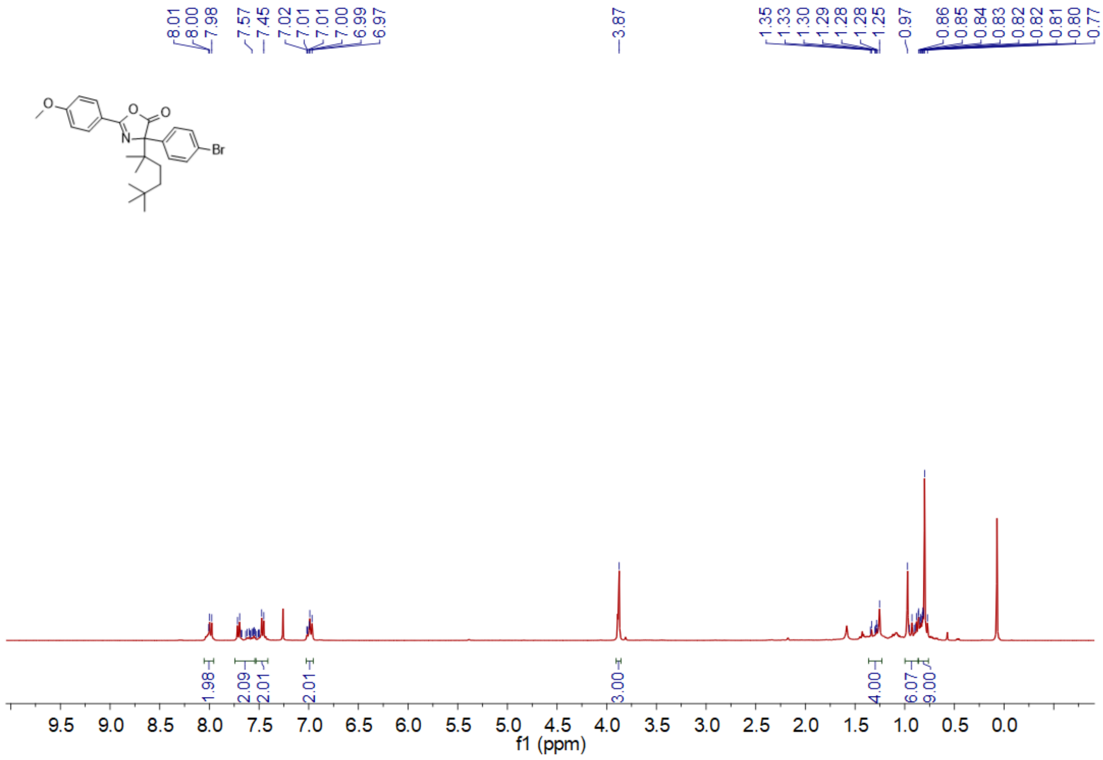

Supplementary Figure 192 ^{1}H NMR (400 MHz, $CDCl_3$, 25 °C) of compound **66**

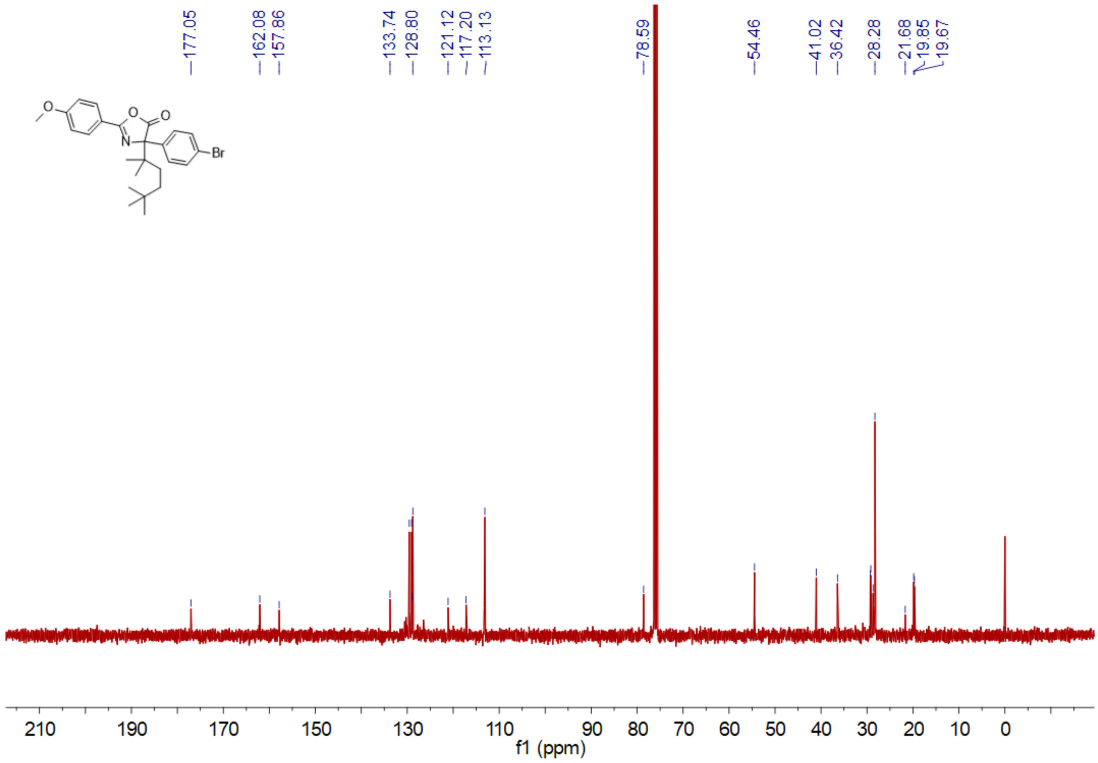

Supplementary Figure 193 ^{13}C NMR (101 MHz, $CDCl_3$, 25 °C) of compound **66**

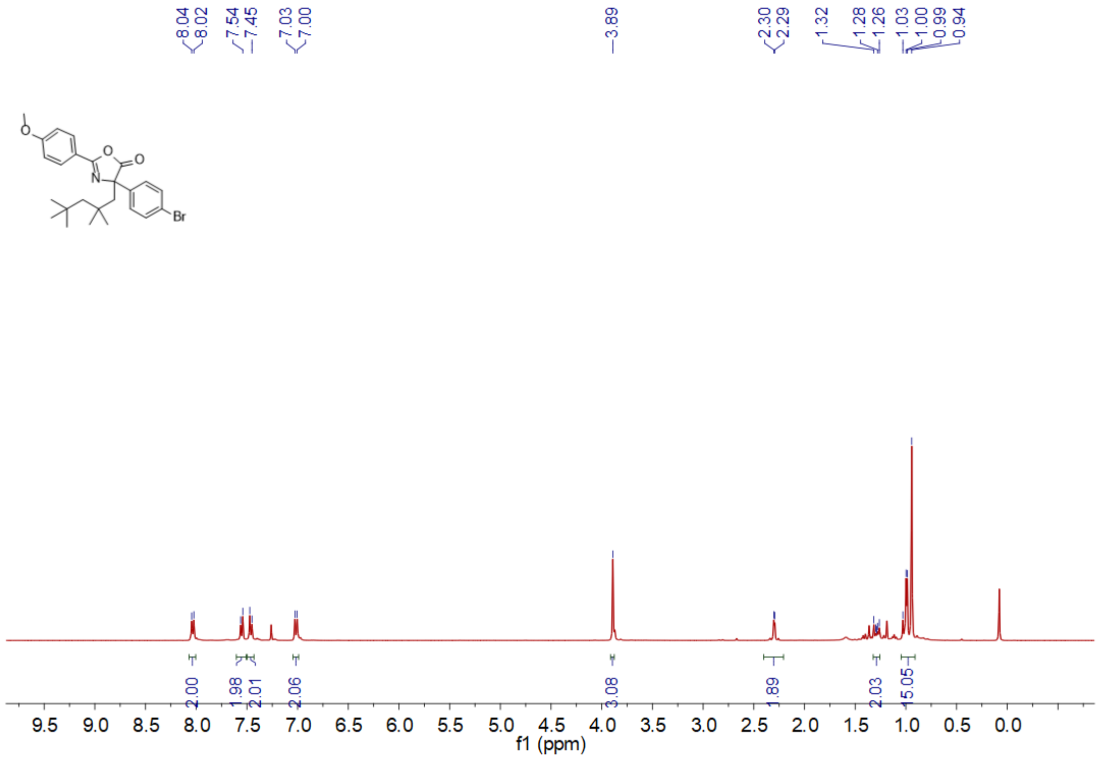

Supplementary Figure 194 ^{1}H NMR (400 MHz, $CDCl_3$, 25 °C) of compound **67**

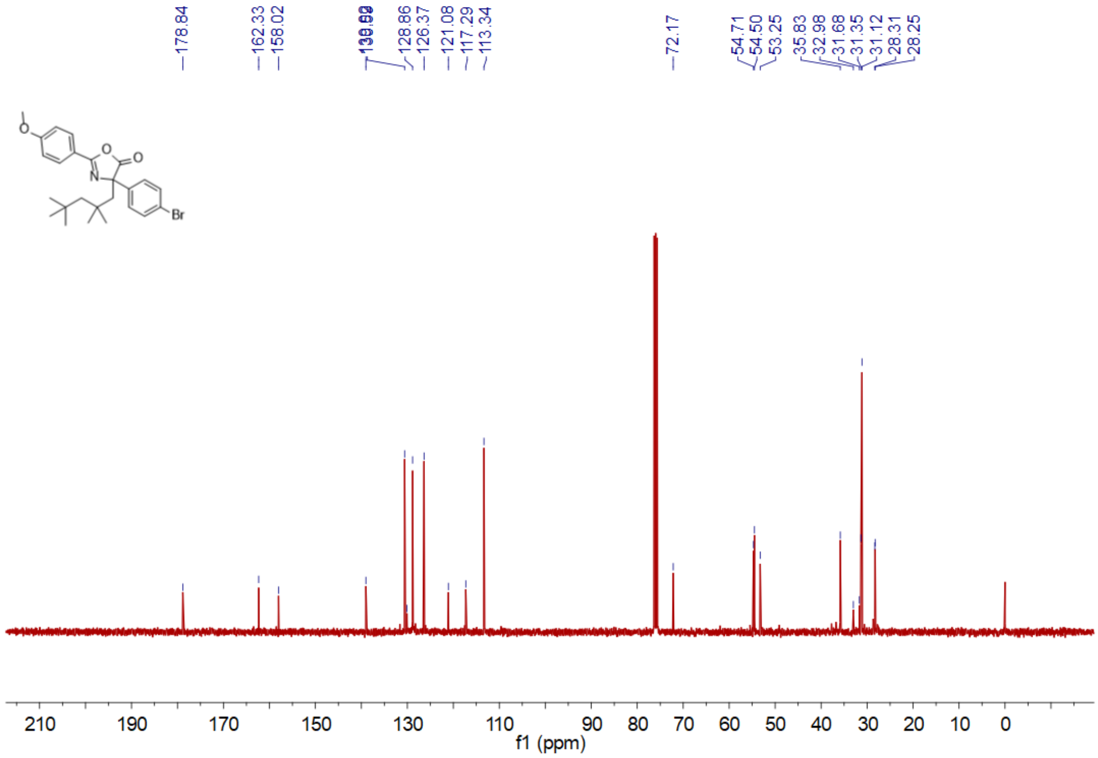

Supplementary Figure 195 ^{13}C NMR (101 MHz, $CDCl_3$, 25 °C) of compound **67**

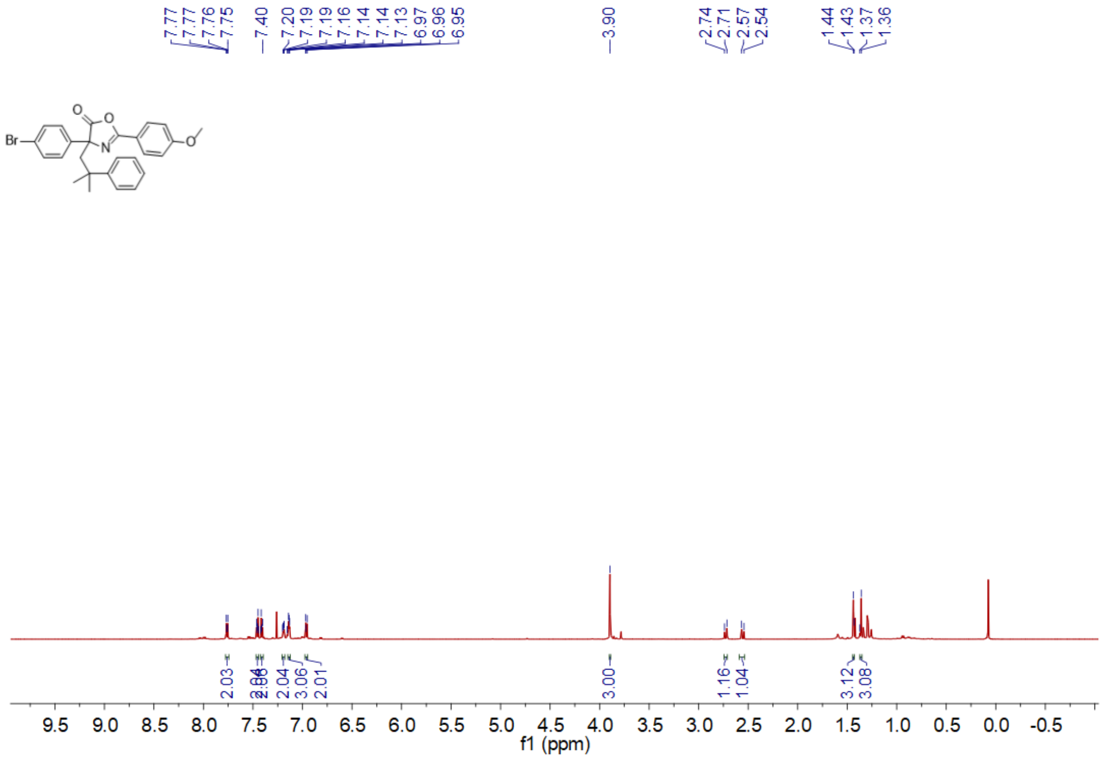

Supplementary Figure 196 ^{1}H NMR (600 MHz, $CDCl_3$, 25 °C) of compound **68**

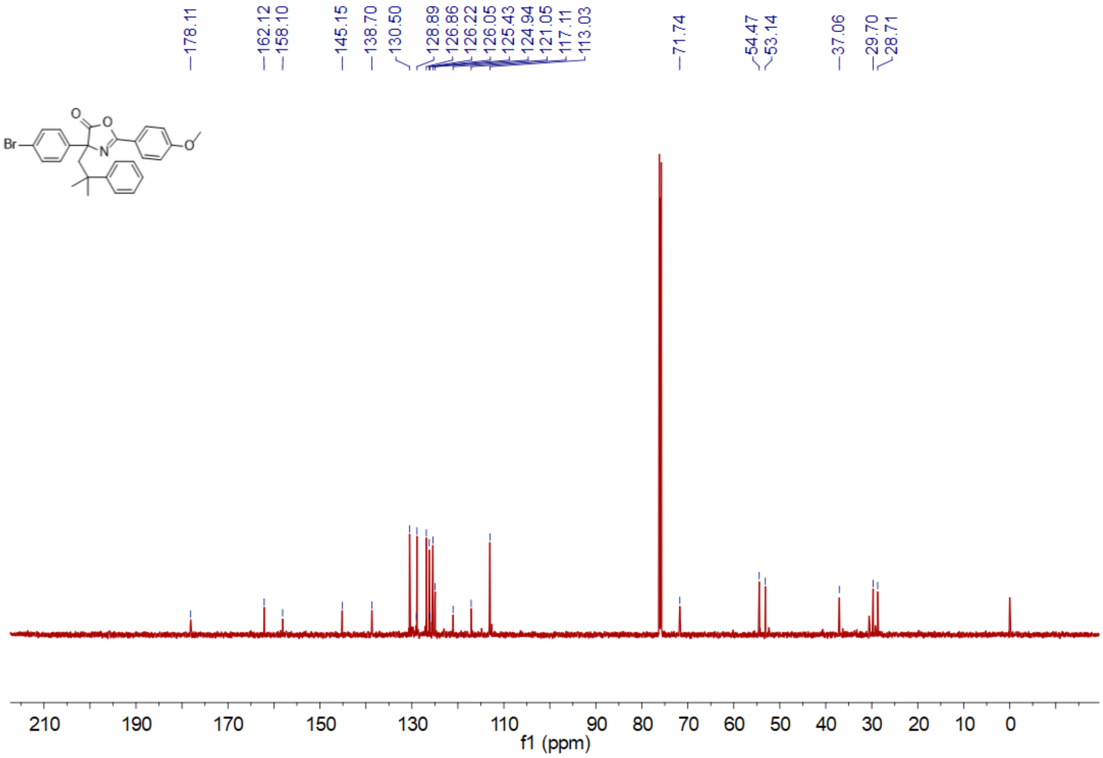

Supplementary Figure 197 ^{13}C NMR (151 MHz, $CDCl_3$, 25 °C) of compound **68**

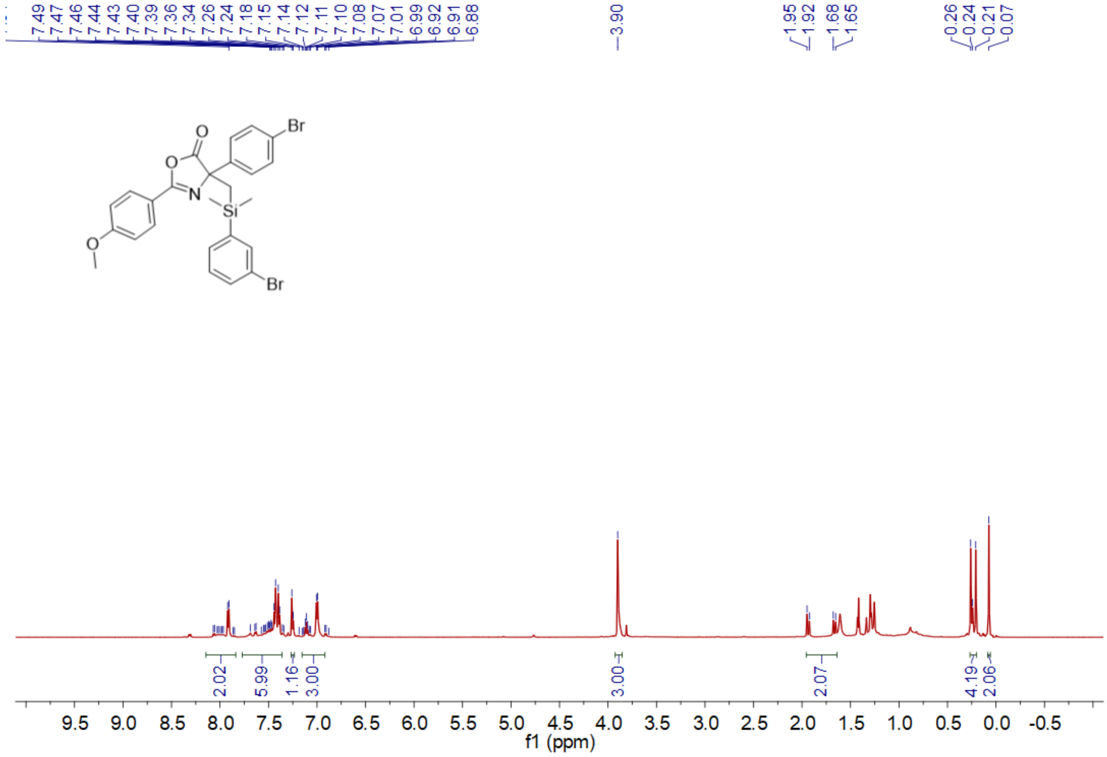

Supplementary Figure 198 ^{1}H NMR (600 MHz, $CDCl_3$, 25 °C) of compound **69**

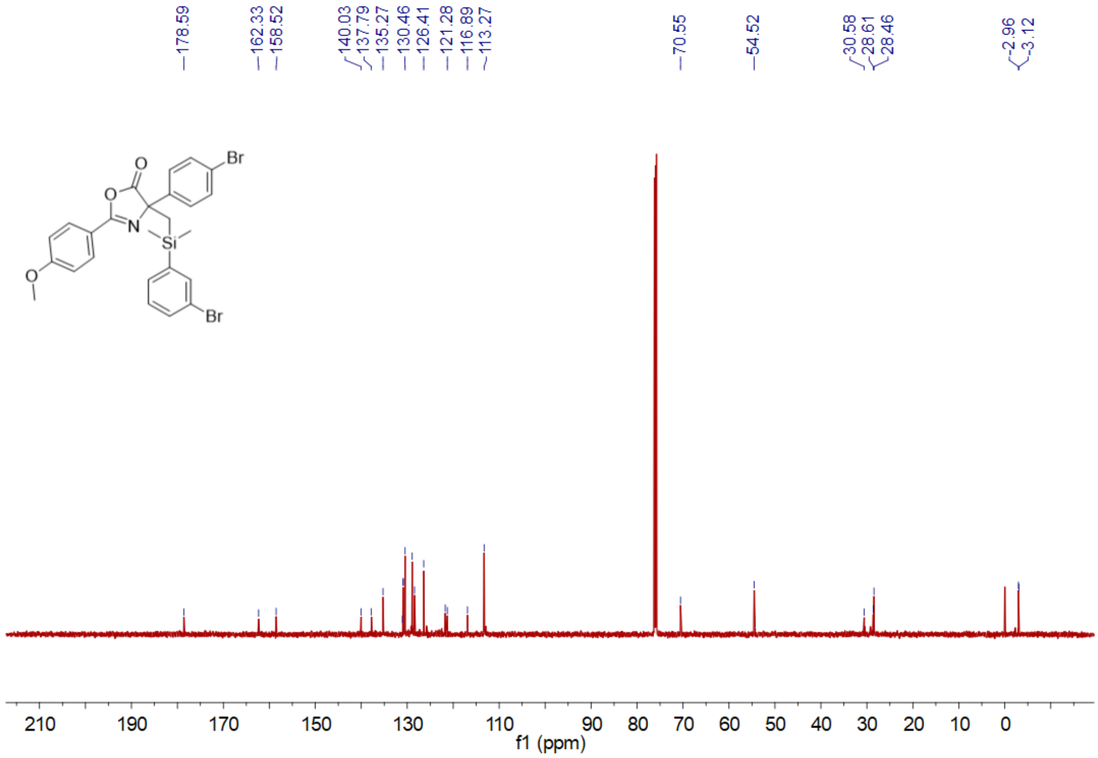

Supplementary Figure 199 ^{13}C NMR (151 MHz, $CDCl_3$, 25 °C) of compound **69**

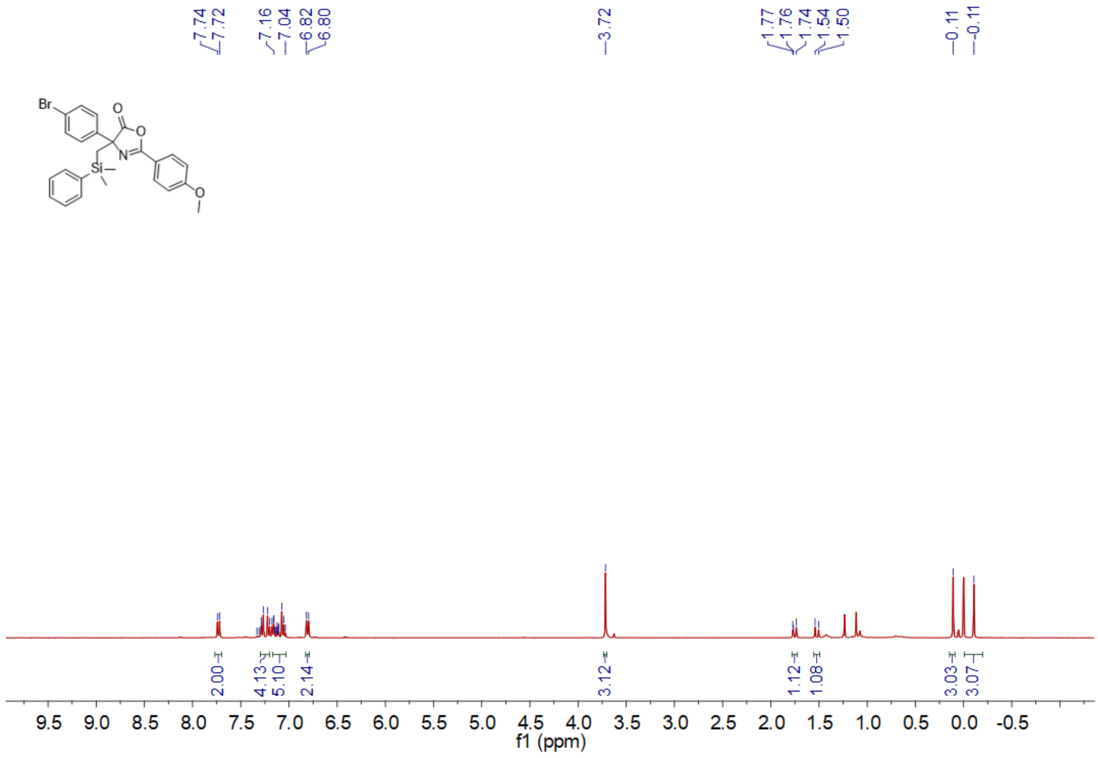

Supplementary Figure 200 ^{1}H NMR (400 MHz, $CDCl_3$, 25 °C) of compound **70**

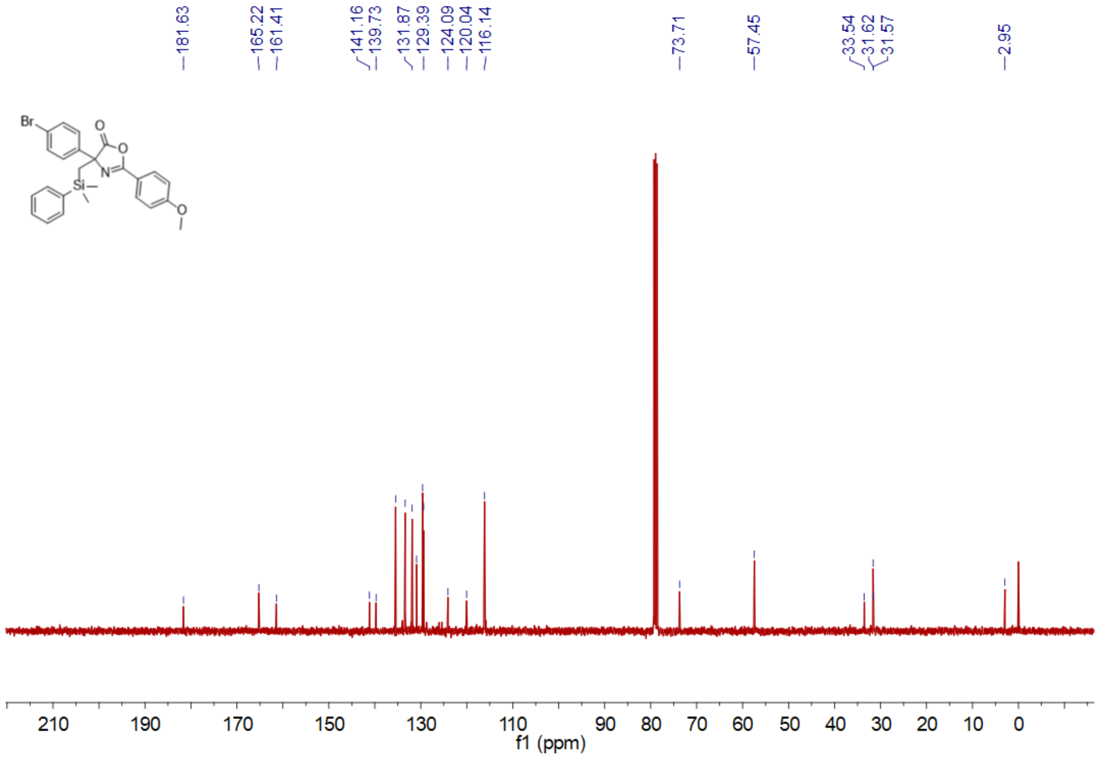

Supplementary Figure 201 ^{13}C NMR (101 MHz, $CDCl_3$, 25 °C) of compound **70**

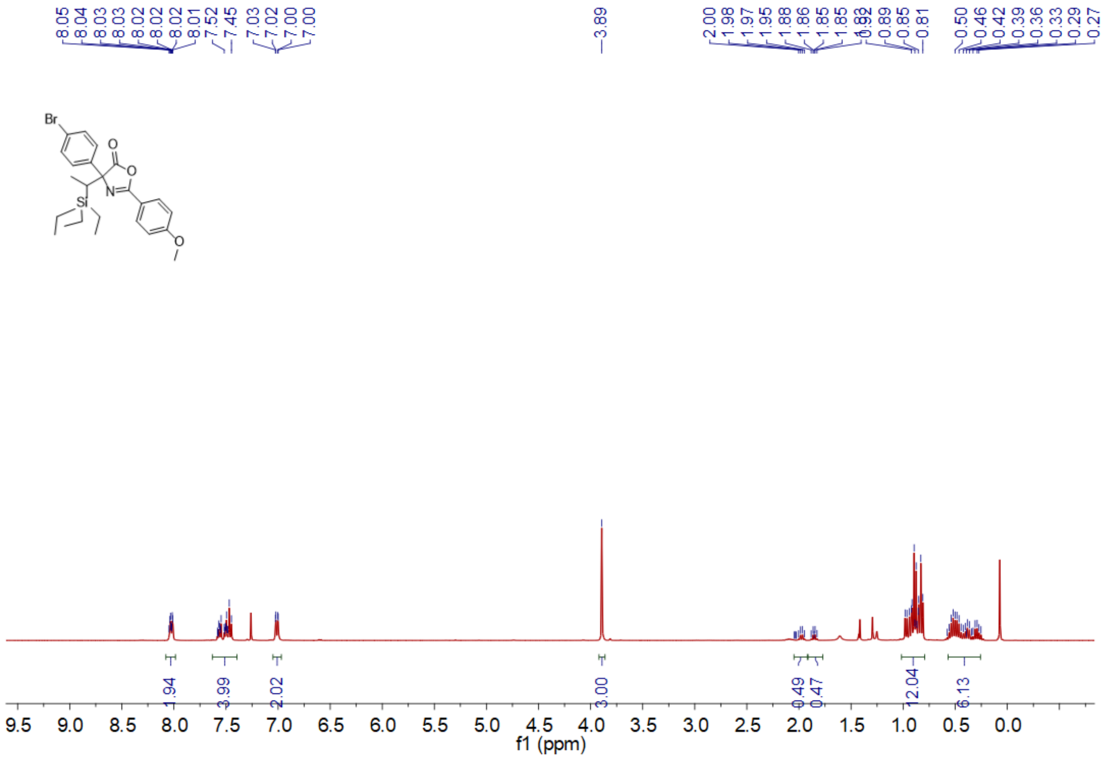

Supplementary Figure 202 ^{1}H NMR (400 MHz, $CDCl_3$, 25 °C) of compound **71**

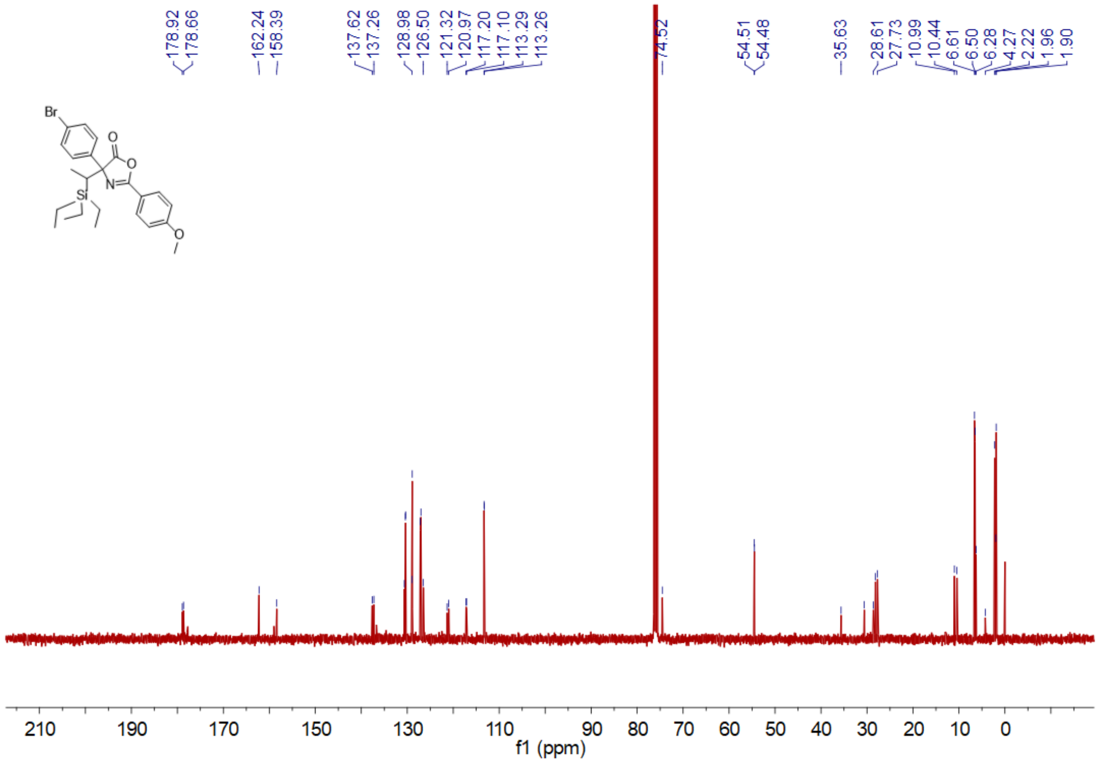

Supplementary Figure 203 ^{13}C NMR (101 MHz, $CDCl_3$, 25 °C) of compound **71**

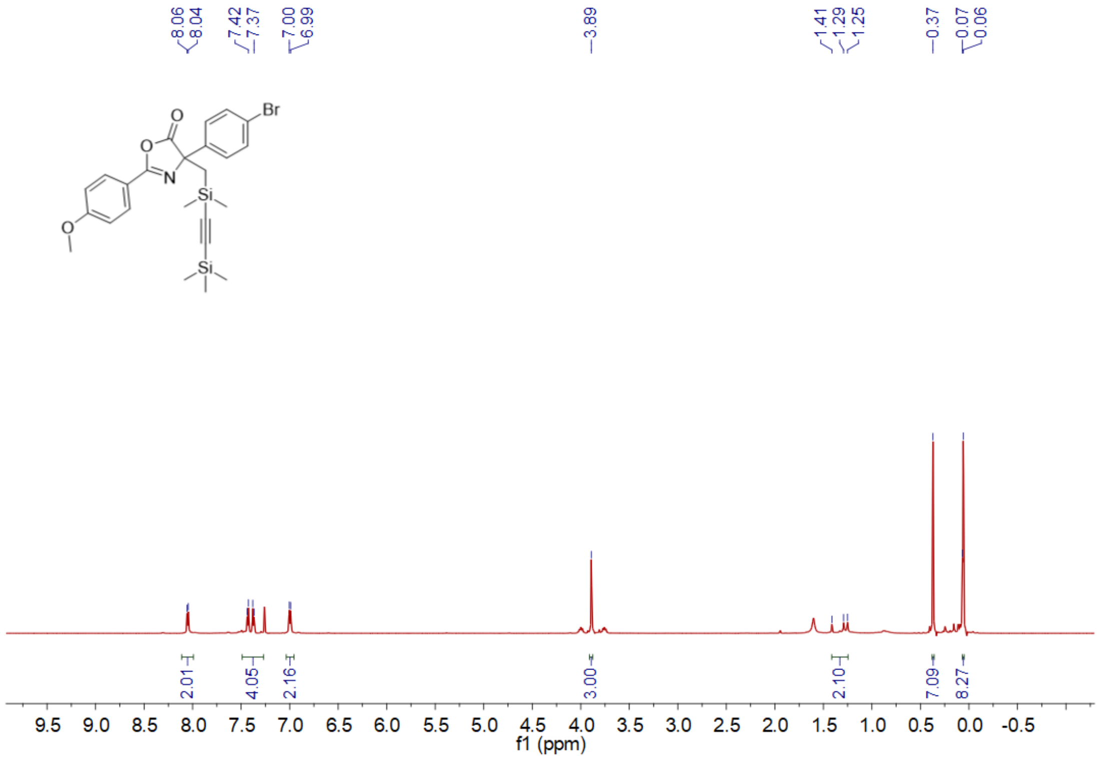

Supplementary Figure 204 ^{1}H NMR (600 MHz, $CDCl_3$, 25 °C) of compound **72**

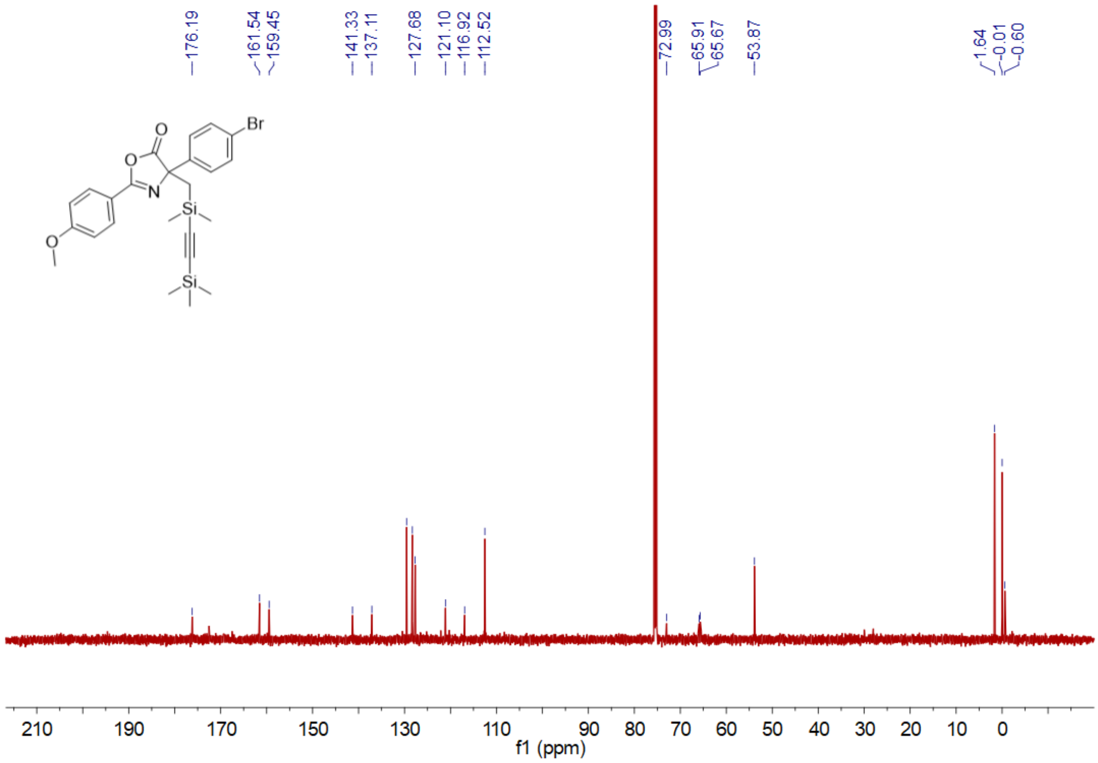

Supplementary Figure 205 ^{13}C NMR (151 MHz, $CDCl_3$, 25 °C) of compound **72**

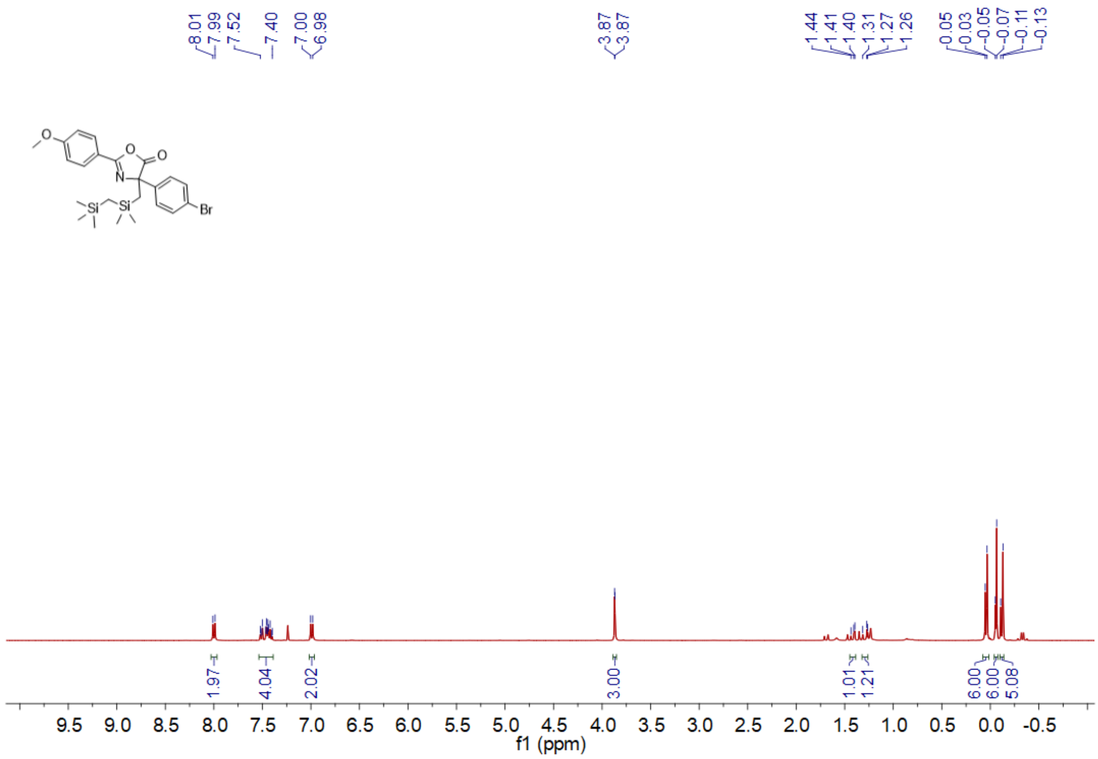

Supplementary Figure 206 ^{1}H NMR (400 MHz, $CDCl_3$, 25 °C) of compound **73**

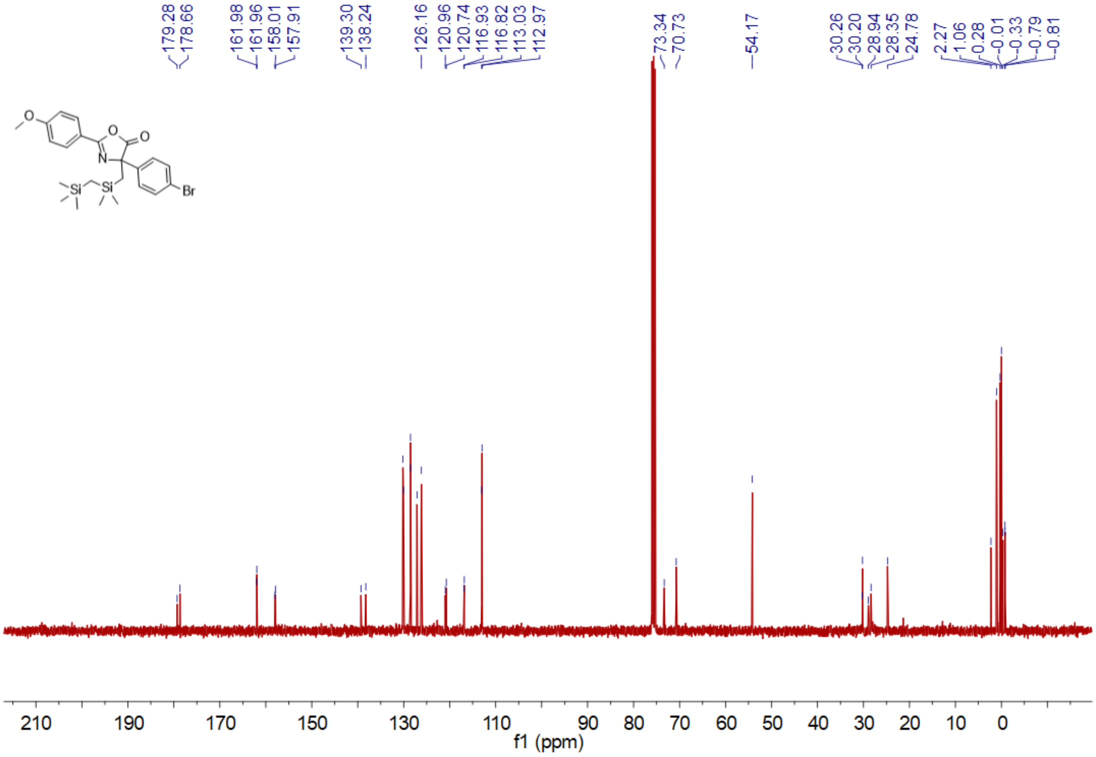

Supplementary Figure 207 ^{13}C NMR (101 MHz, $CDCl_3$, 25 °C) of compound **73**

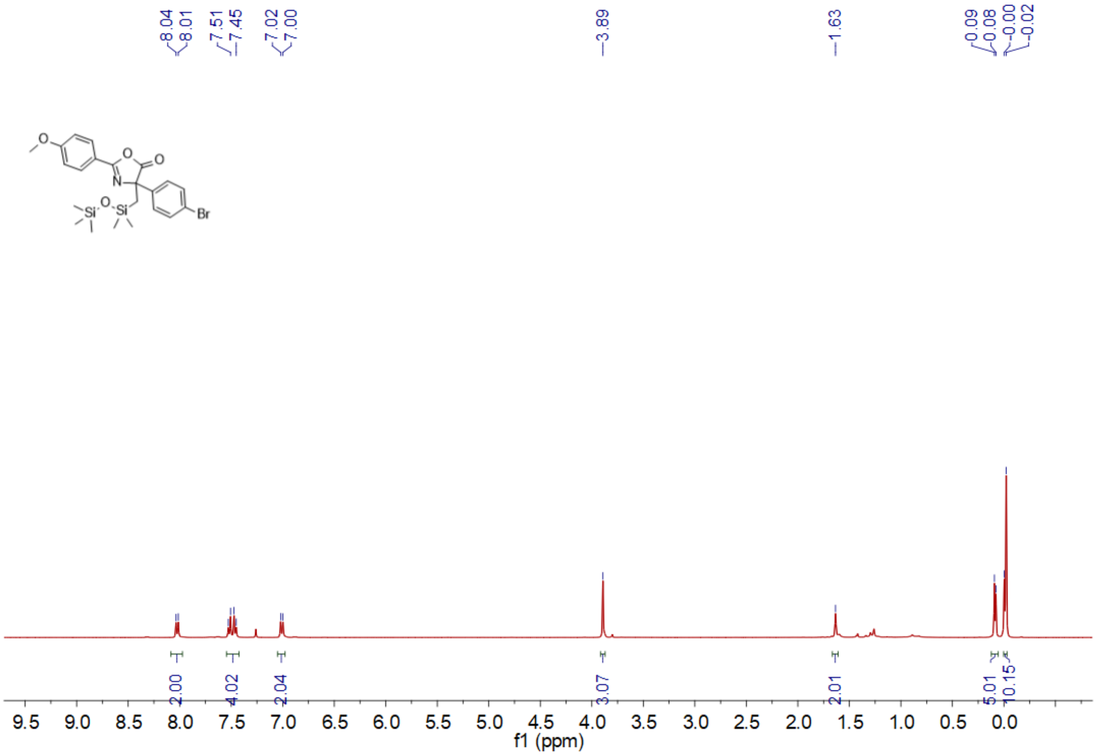

Supplementary Figure 208 ^{1}H NMR (400 MHz, $CDCl_3$, 25 °C) of compound **74**

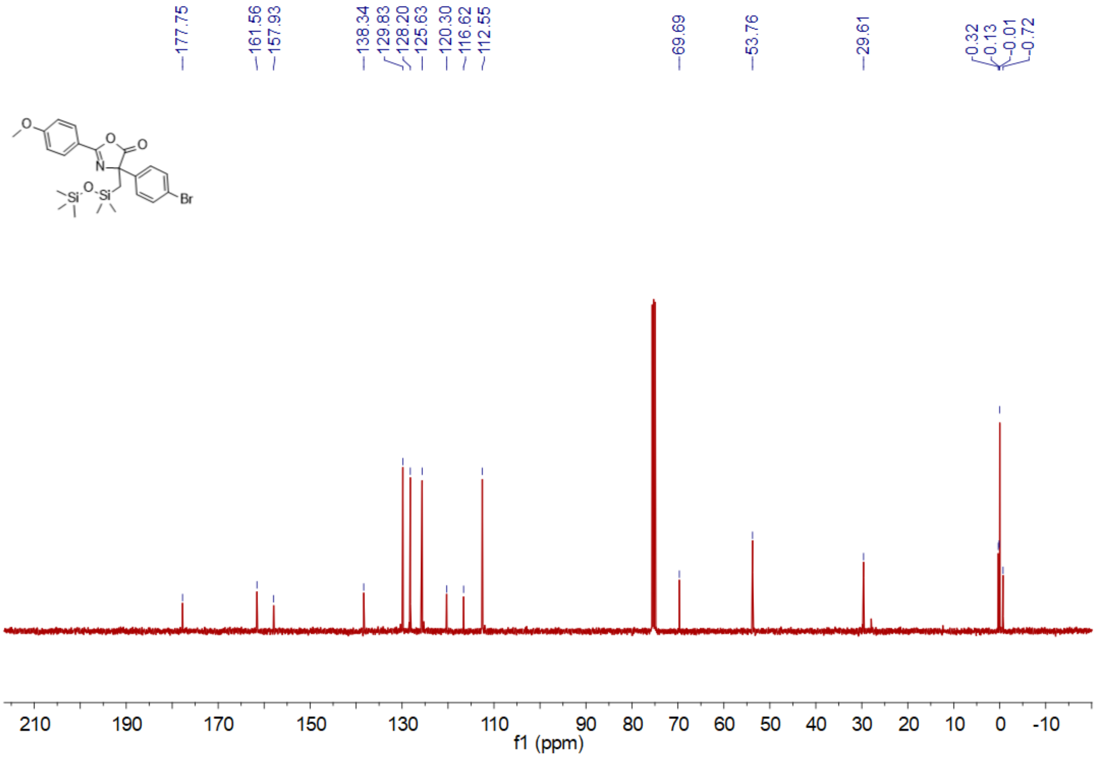

Supplementary Figure 209 ^{13}C NMR (101 MHz, $CDCl_3$, 25 °C) of compound **74**

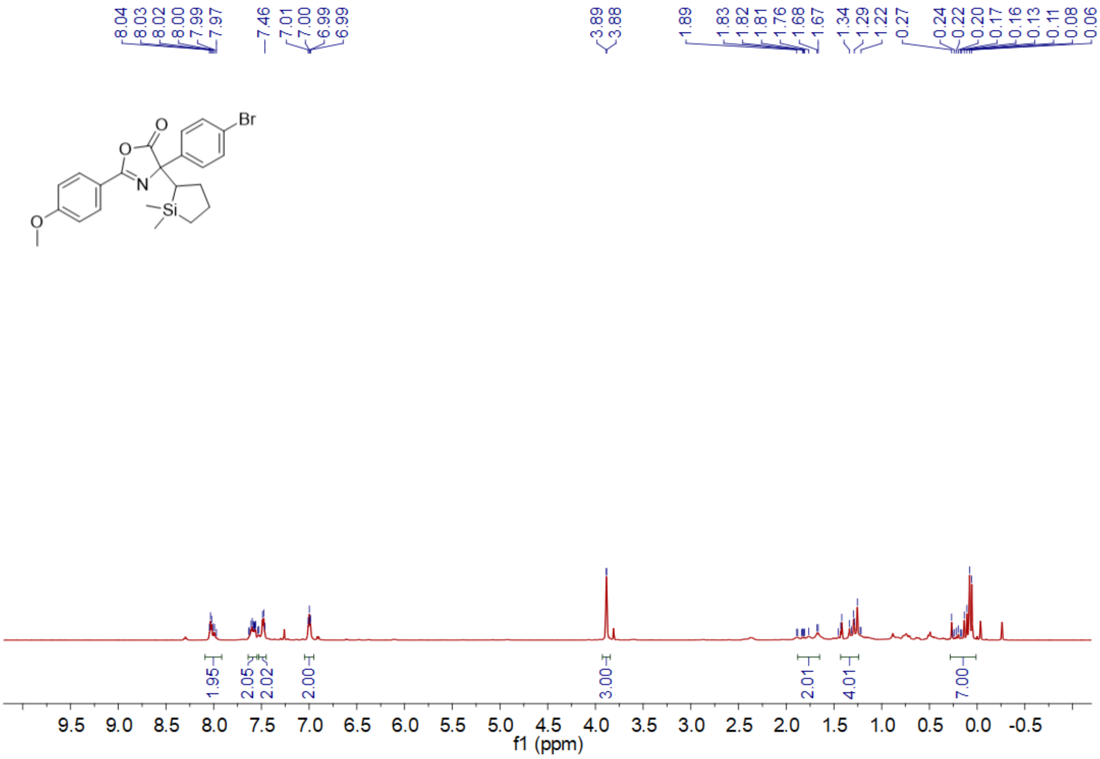

Supplementary Figure 210 ^{1}H NMR (600 MHz, $CDCl_3$, 25 °C) of compound **75**

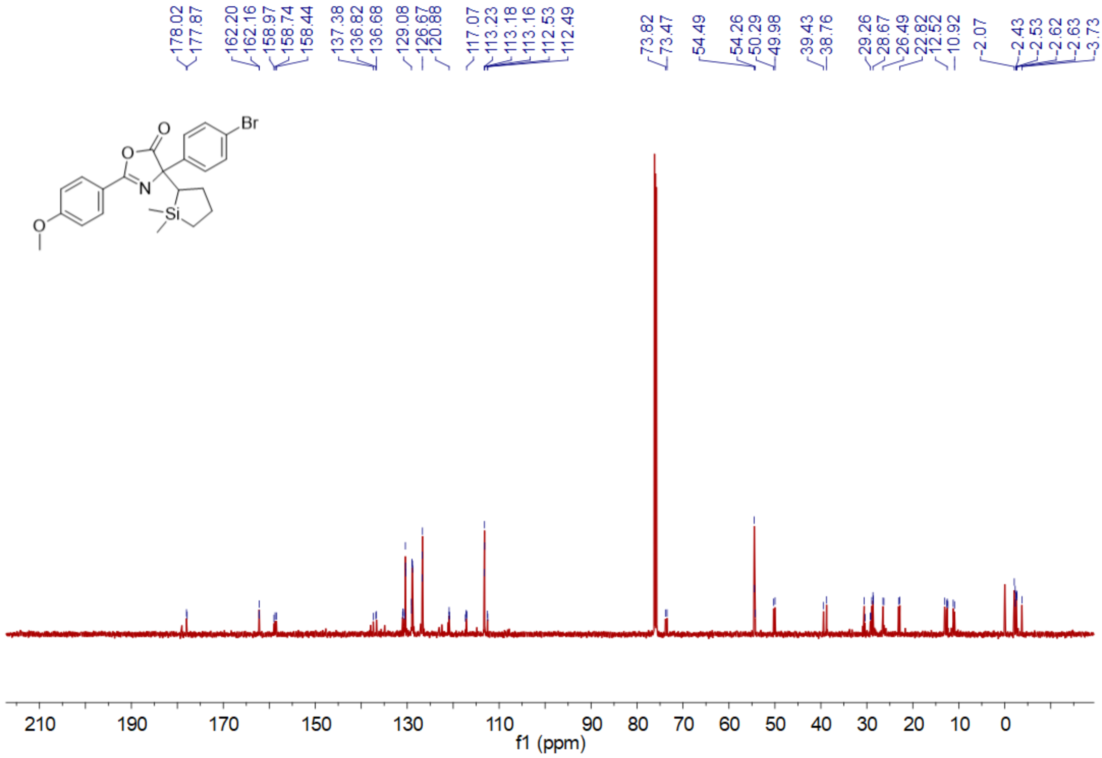

Supplementary Figure 211 ^{13}C NMR (151 MHz, $CDCl_3$, 25 °C) of compound **75**

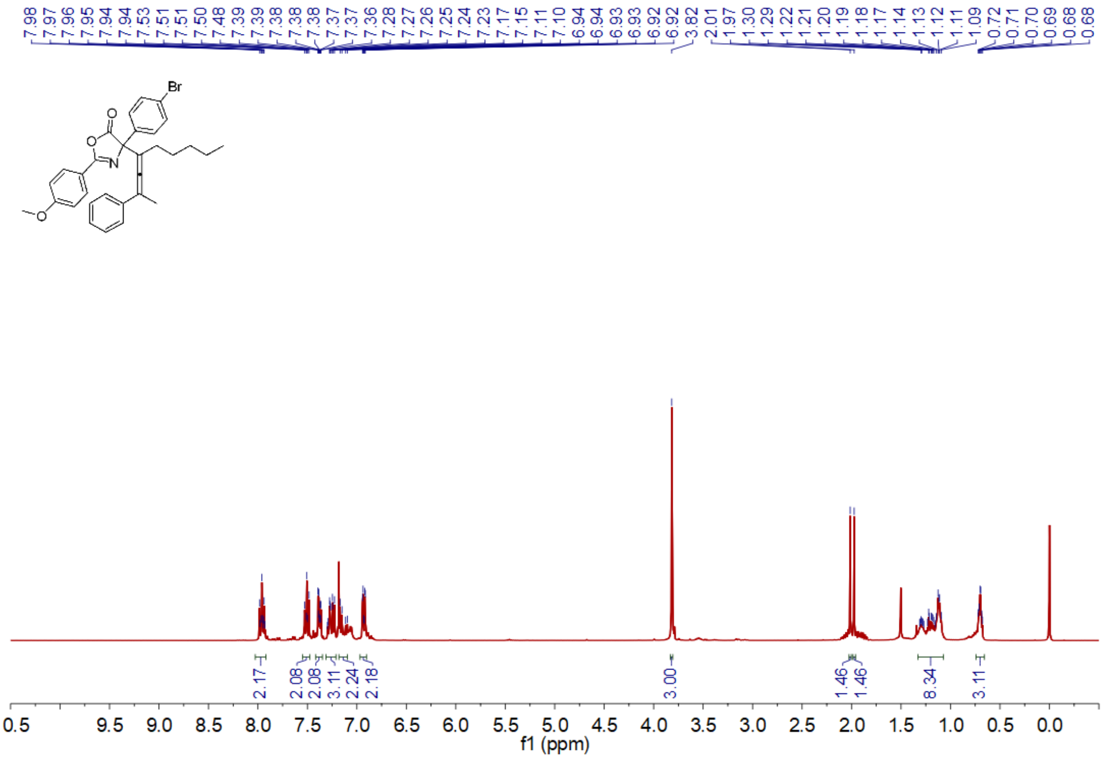

Supplementary Figure 212 ^{1}H NMR (400 MHz, $CDCl_3$, 25 °C) of compound **76**

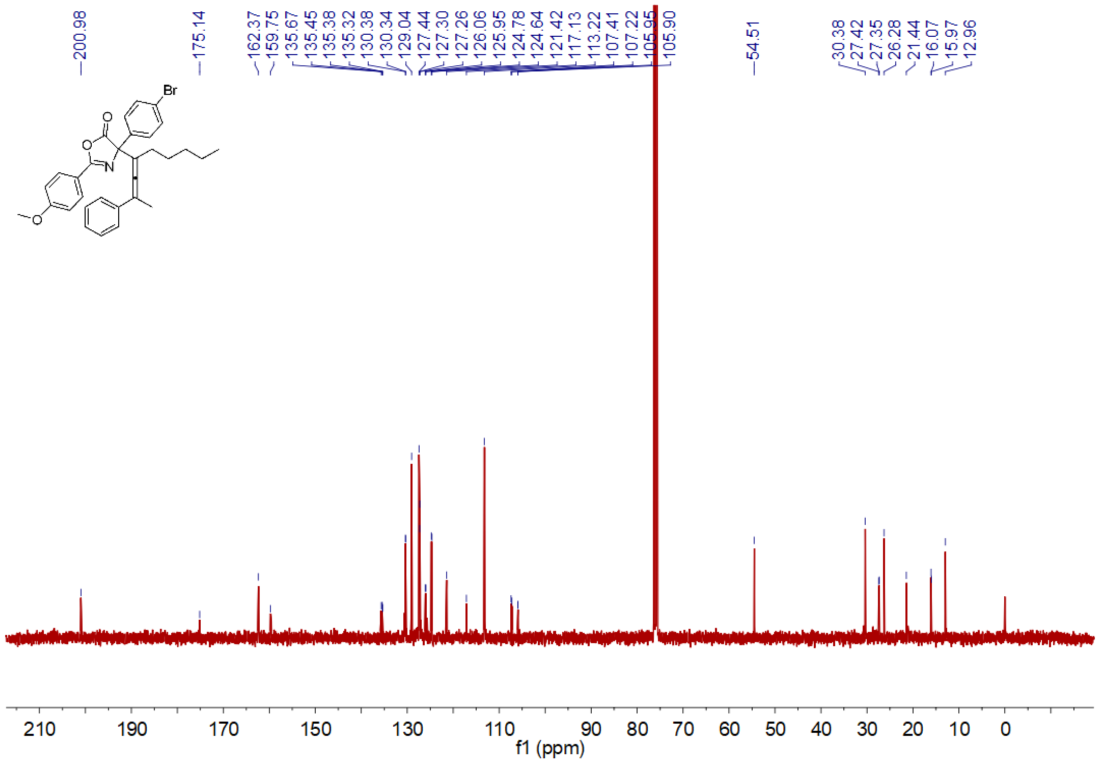

Supplementary Figure 213 ^{13}C NMR (101 MHz, $CDCl_3$, 25 °C) of compound **76**

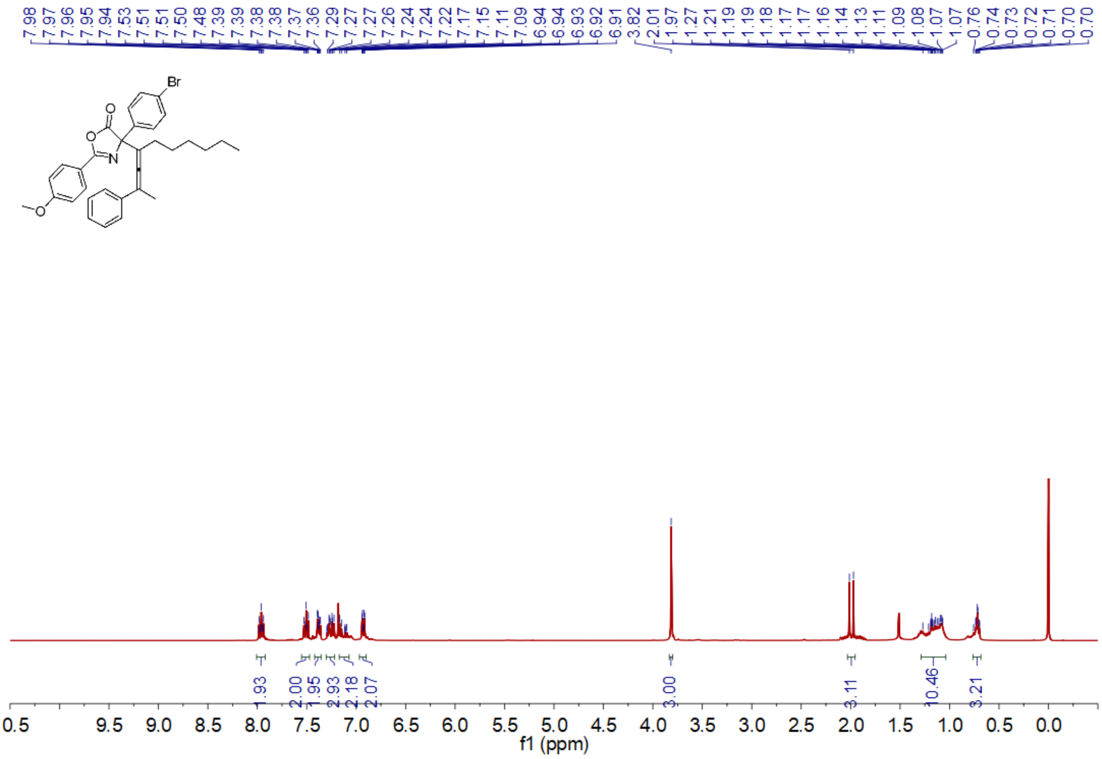

Supplementary Figure 214 ^{1}H NMR (400 MHz, $CDCl_3$, 25 °C) of compound **77**

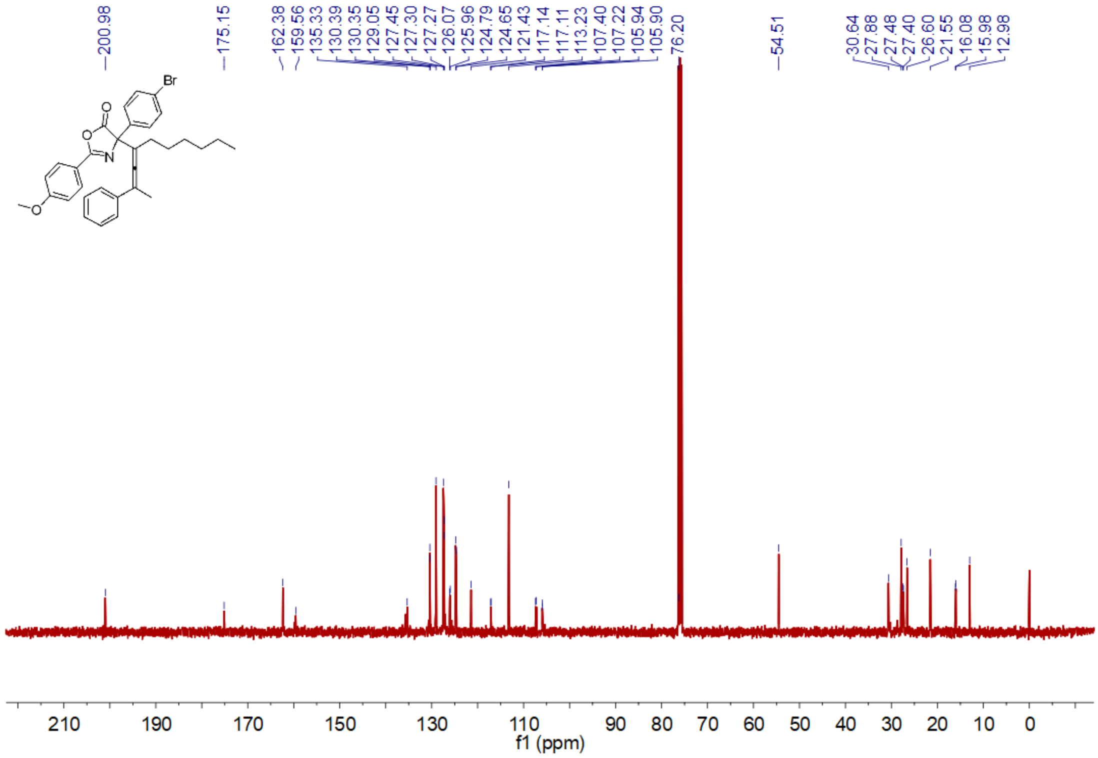

Supplementary Figure 215 ^{13}C NMR (101 MHz, $CDCl_3$, 25 °C) of compound **77**

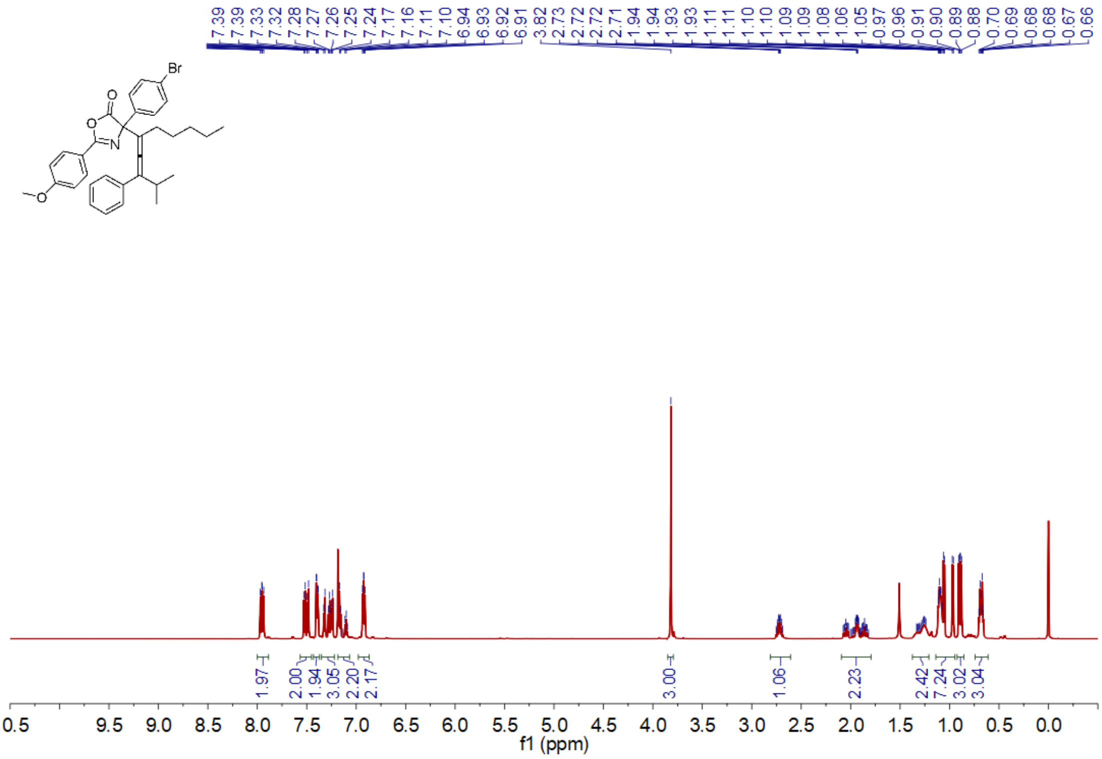

Supplementary Figure 216 ^{1}H NMR (600 MHz, $CDCl_3$, 25 °C) of compound **78**

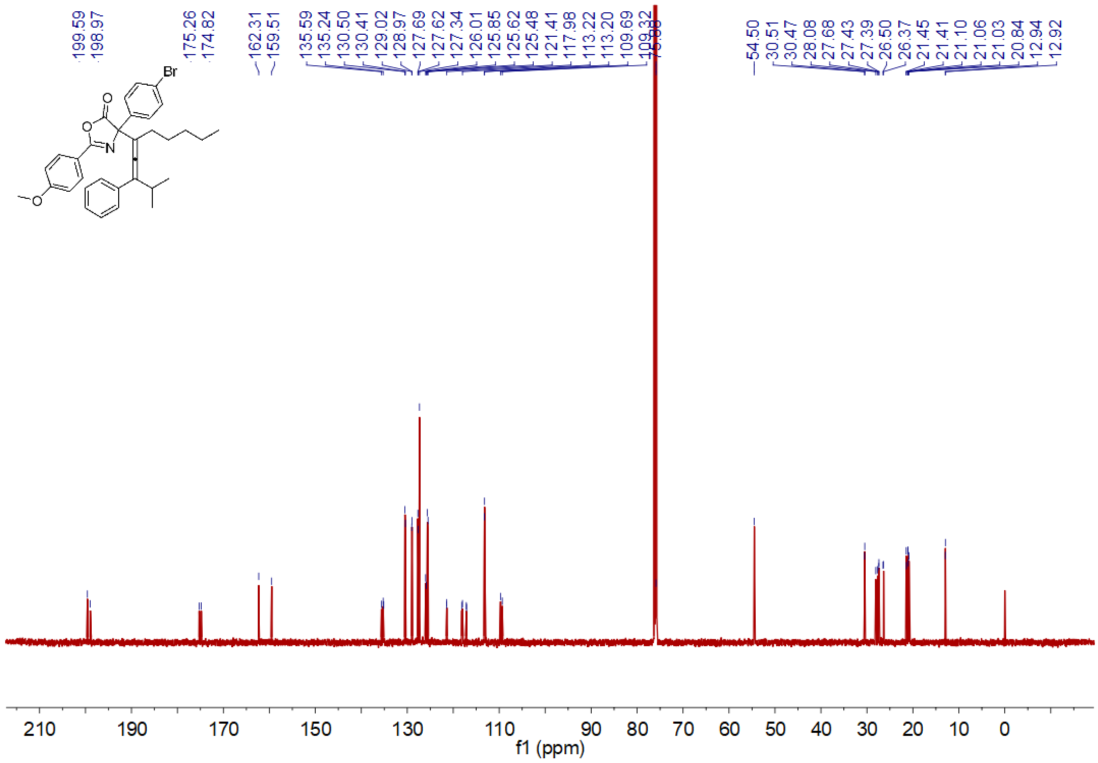

Supplementary Figure 217 ^{13}C NMR (151 MHz, $CDCl_3$, 25 °C) of compound **78**

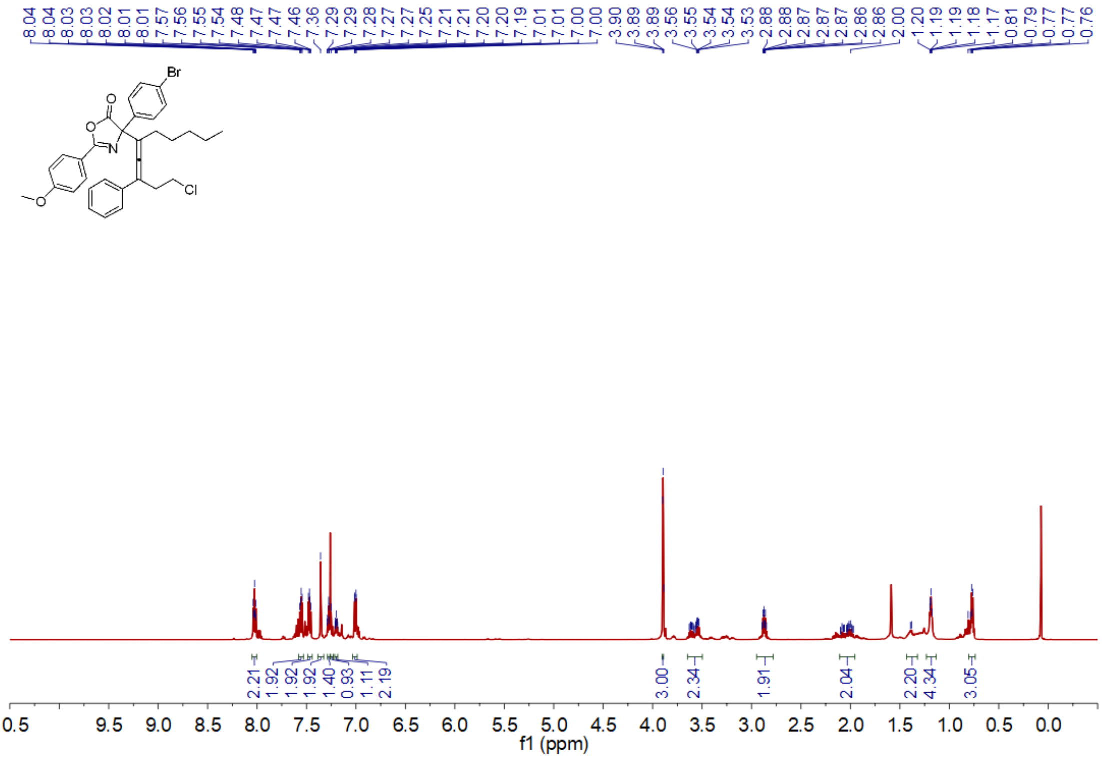

Supplementary Figure 218 ^{1}H NMR (600 MHz, $CDCl_3$, 25 °C) of compound **79**

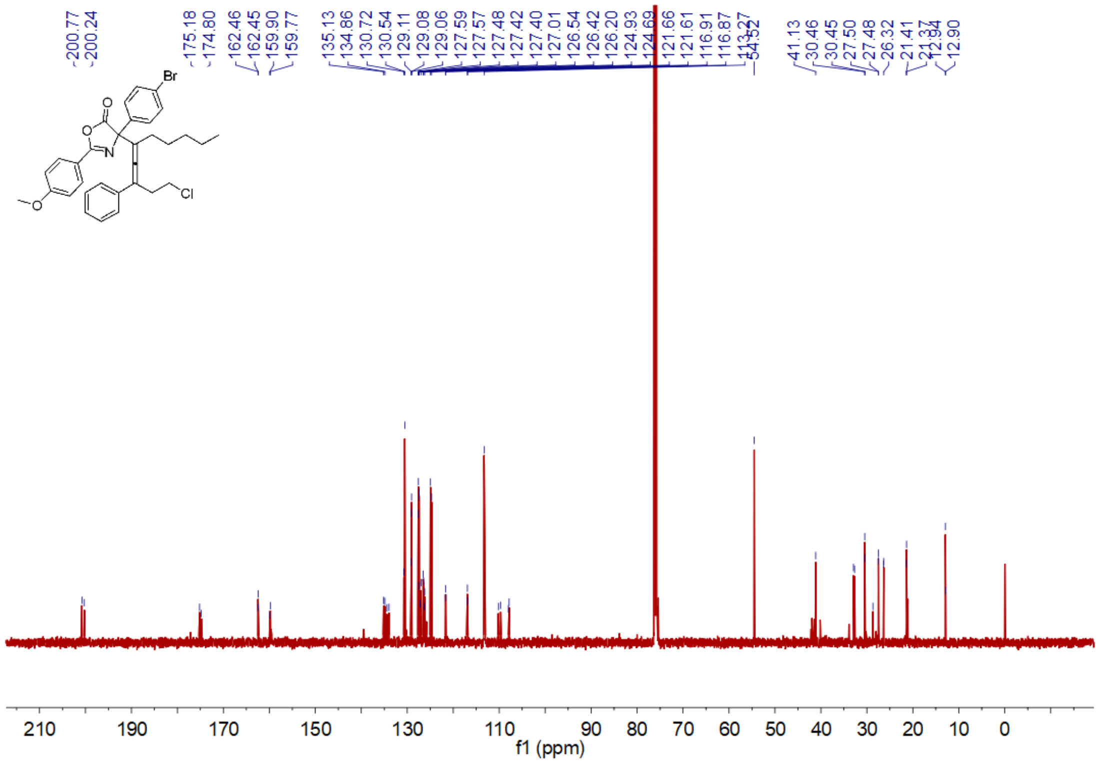

Supplementary Figure 219 ^{13}C NMR (151 MHz, $CDCl_3$, 25 °C) of compound **79**

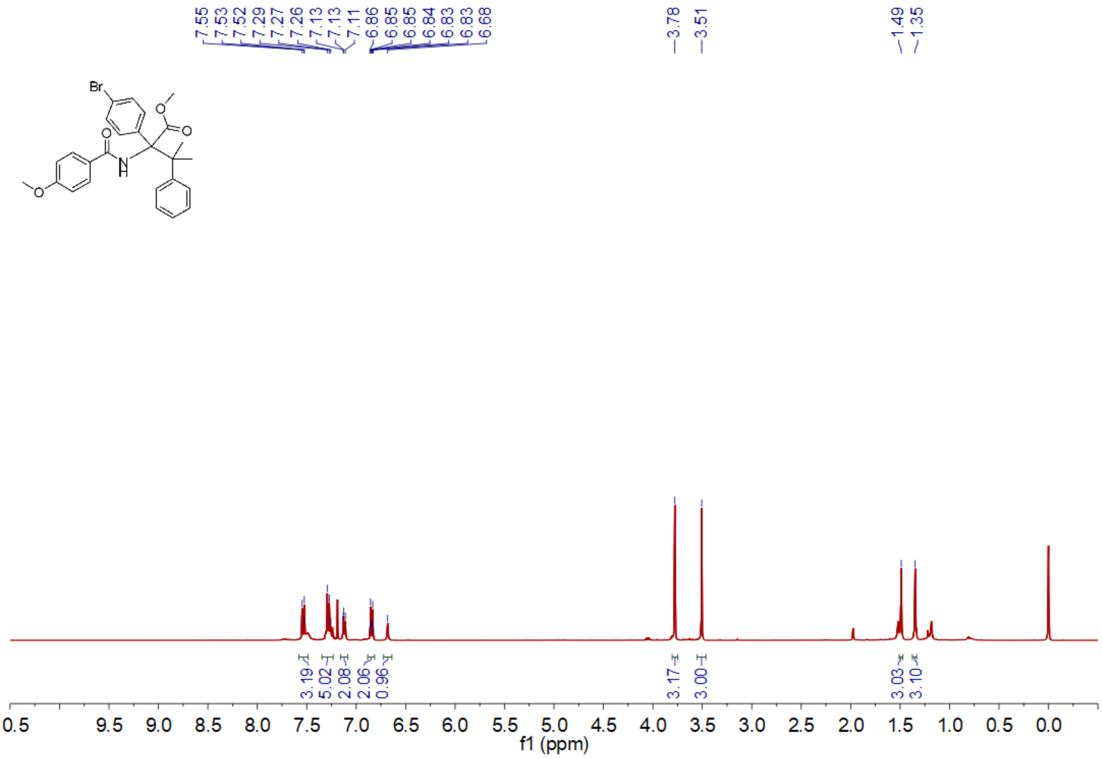

Supplementary Figure 220 ^{1}H NMR (400 MHz, $CDCl_3$, 25 °C) of compound **82**

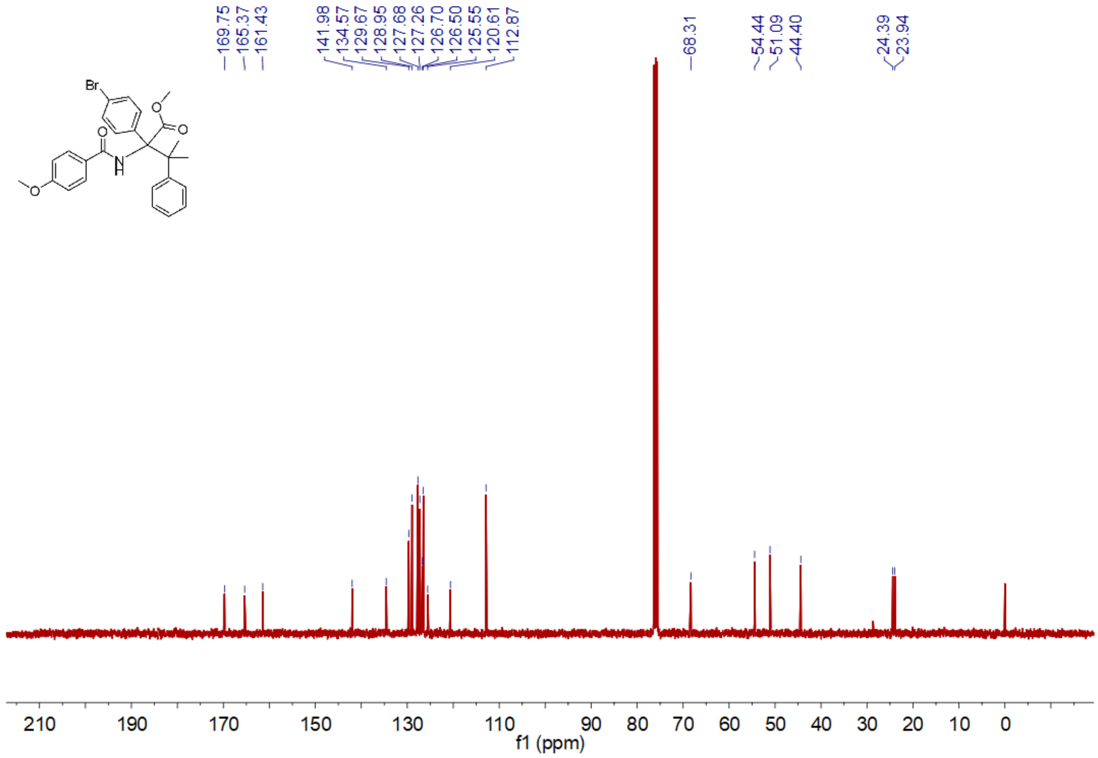

Supplementary Figure 221 ^{13}C NMR (101 MHz, $CDCl_3$, 25 °C) of compound **82**

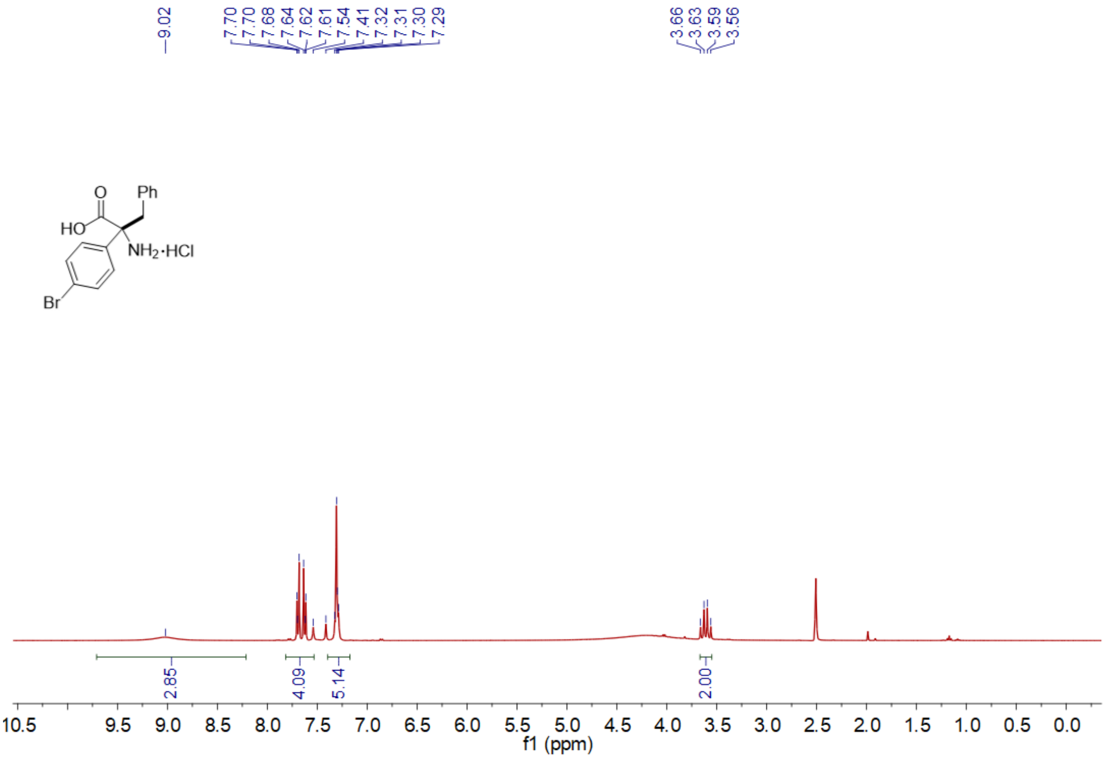

Supplementary Figure 222 ^{1}H NMR (400 MHz, DMSO, 25 °C) of compound **83**

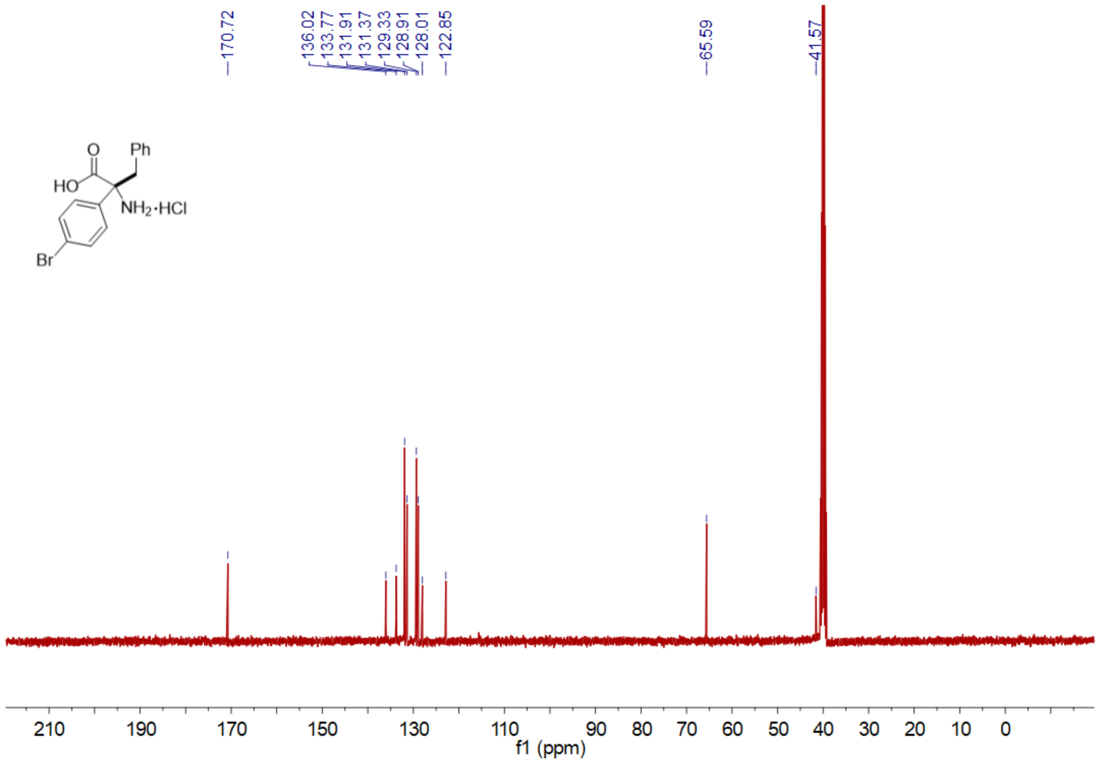

Supplementary Figure 223 ^{13}C NMR (101 MHz, DMSO, 25 °C) of compound **83**

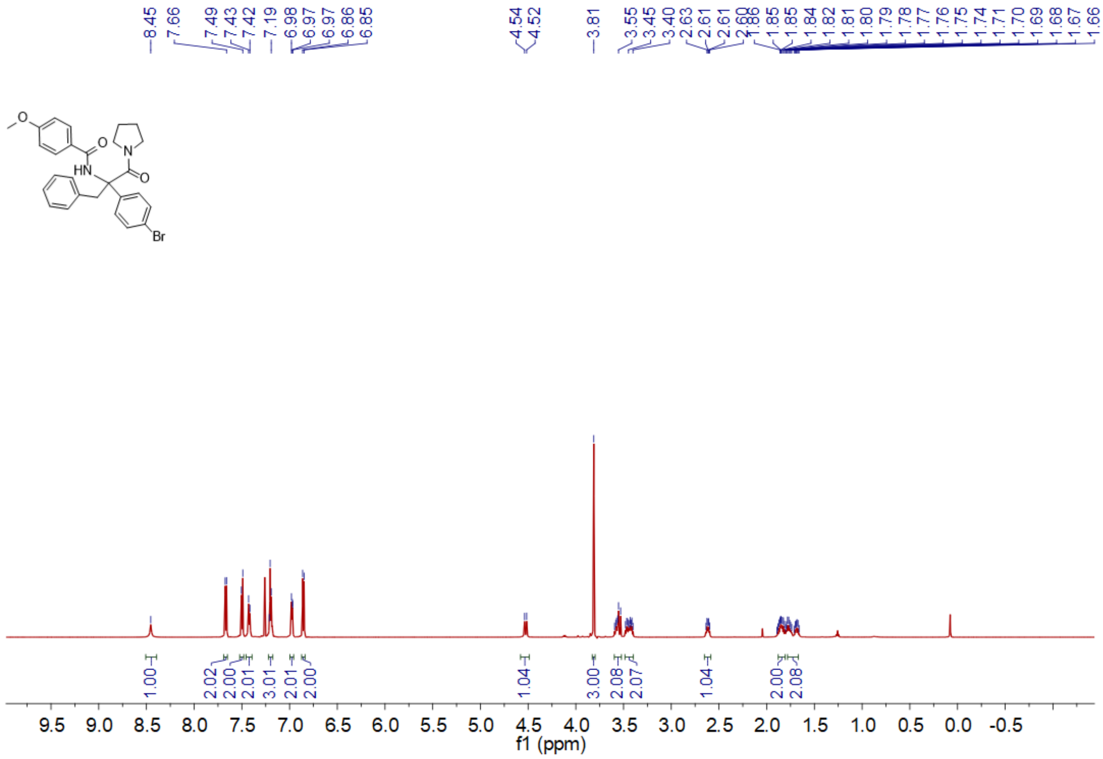

Supplementary Figure 224 ^{1}H NMR (600 MHz, $CDCl_3$, 25 °C) of compound **84**

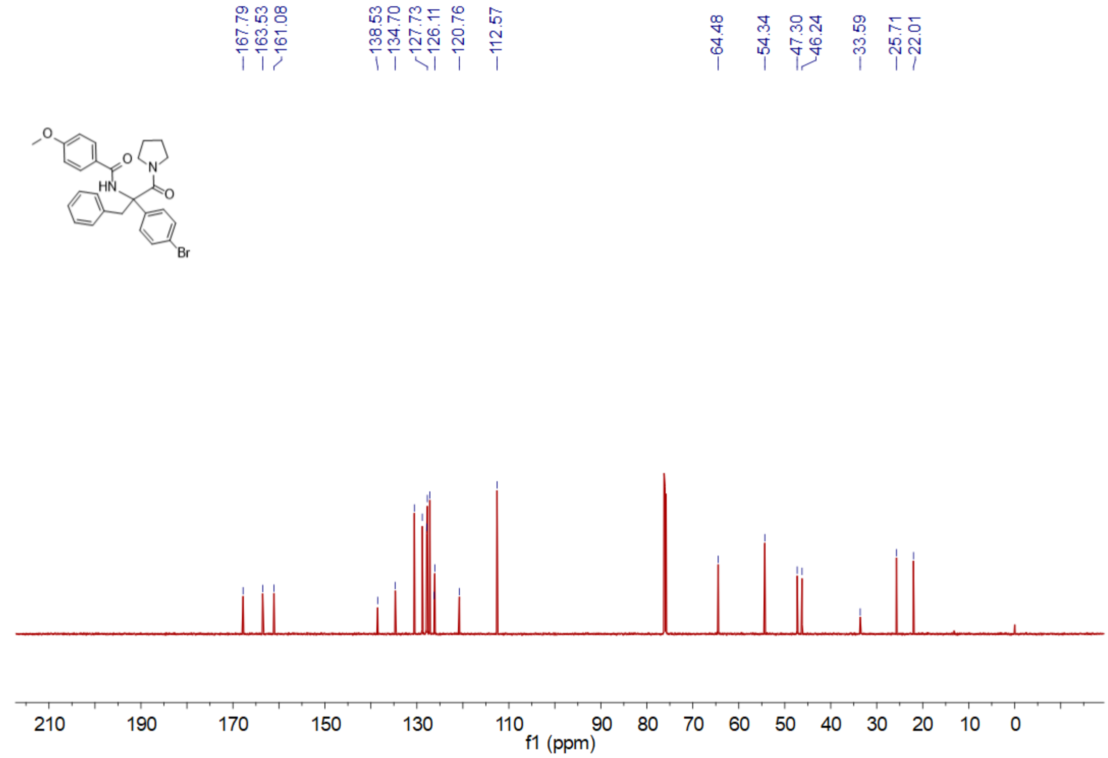

Supplementary Figure 225 ^{13}C NMR (151 MHz, $CDCl_3$, 25 °C) of compound **84**

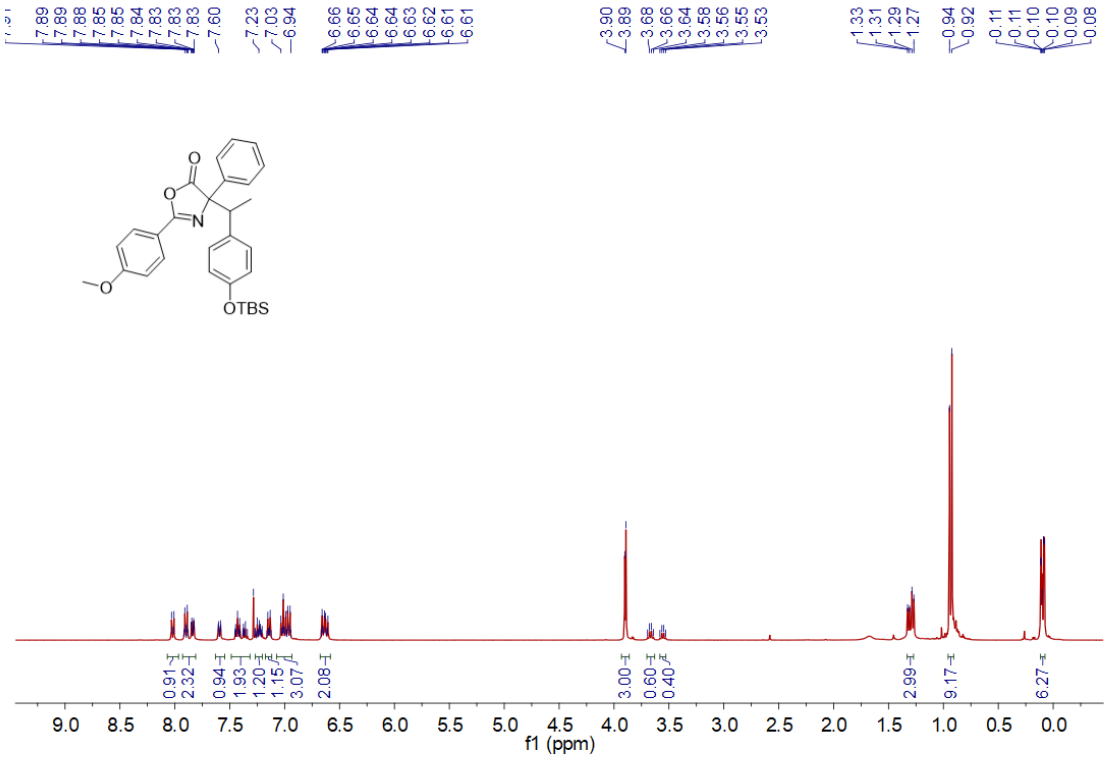

Supplementary Figure 226 ^{1}H NMR (400 MHz, $CDCl_3$, 25 °C) of compound **86**

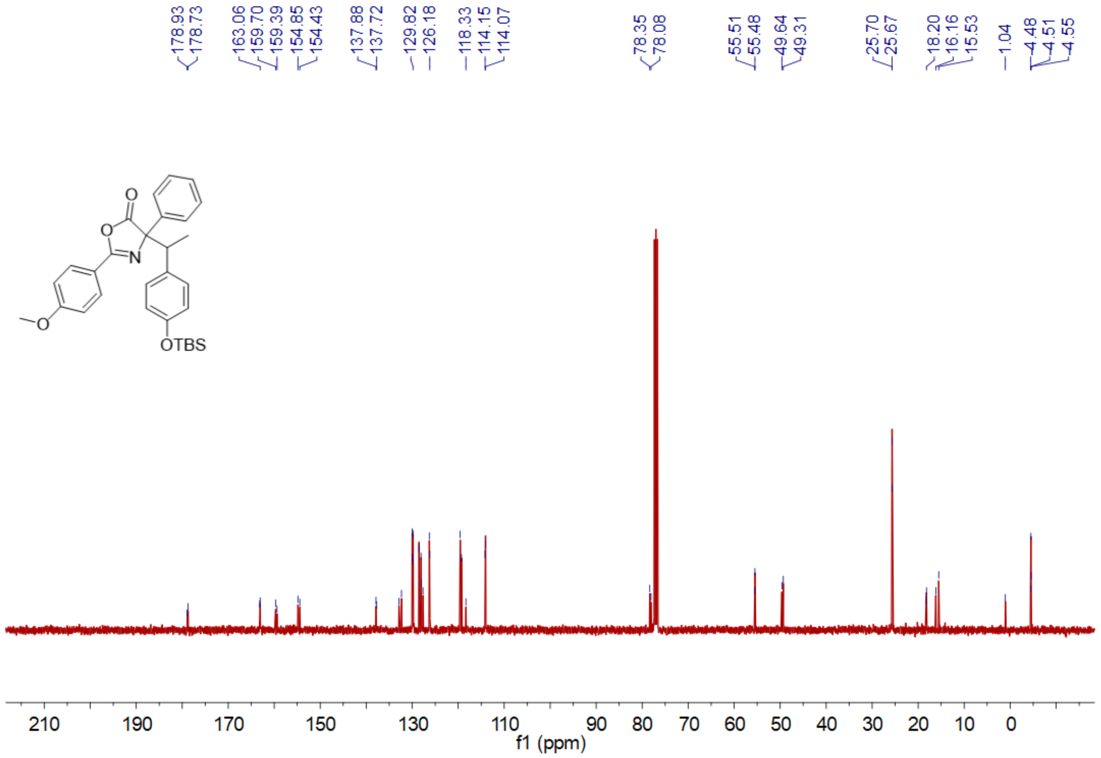

Supplementary Figure 227 ^{13}C NMR (101 MHz, $CDCl_3$, 25 °C) of compound **86**

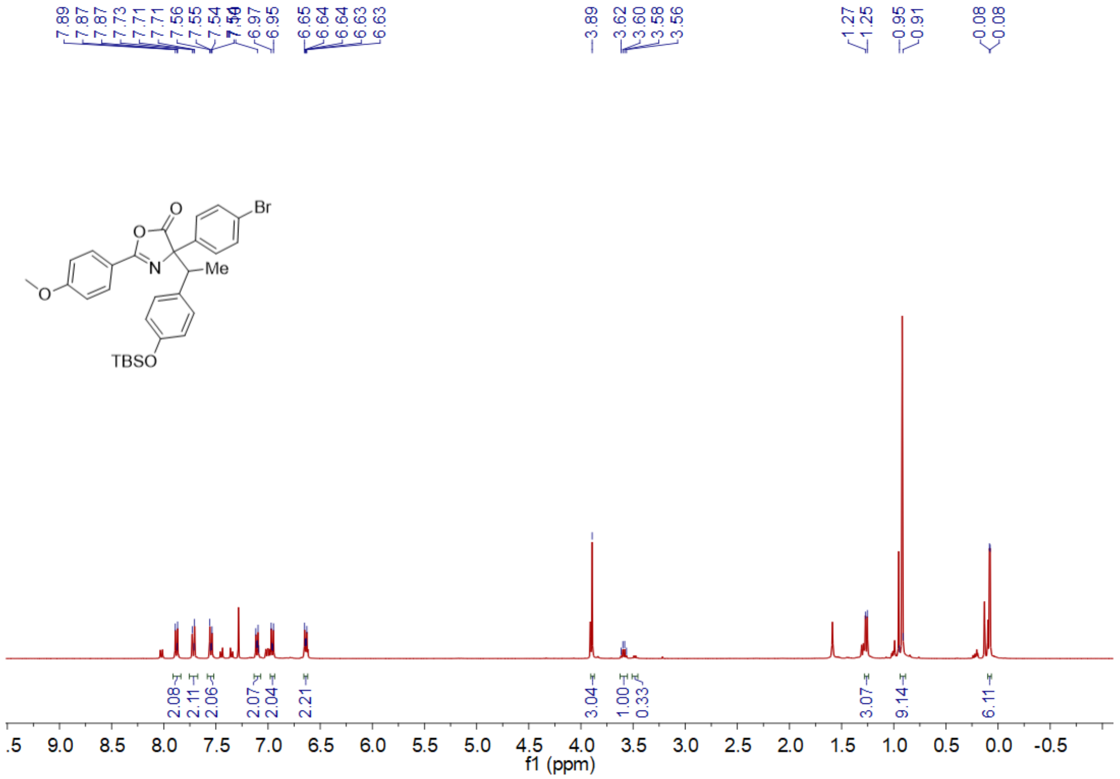

Supplementary Figure 228 ^{1}H NMR (400 MHz, $CDCl_3$, 25 °C) of compound **87**

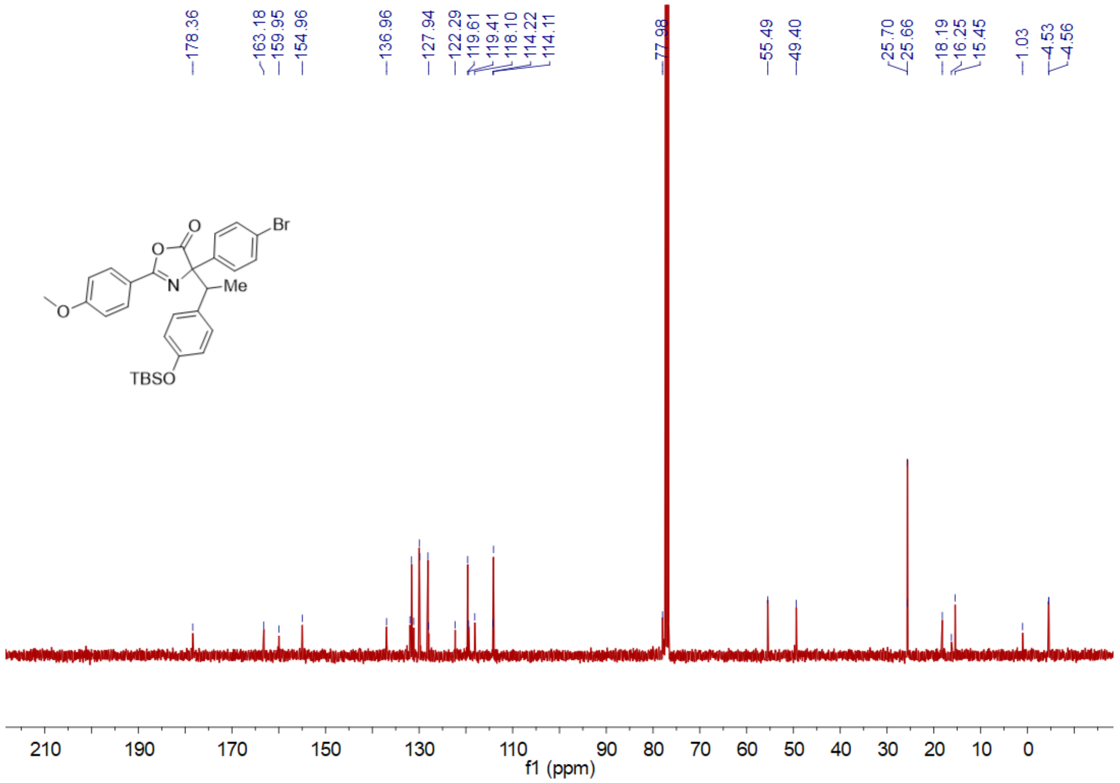

Supplementary Figure 229 ^{13}C NMR (101 MHz, $CDCl_3$, 25 °C) of compound **87**

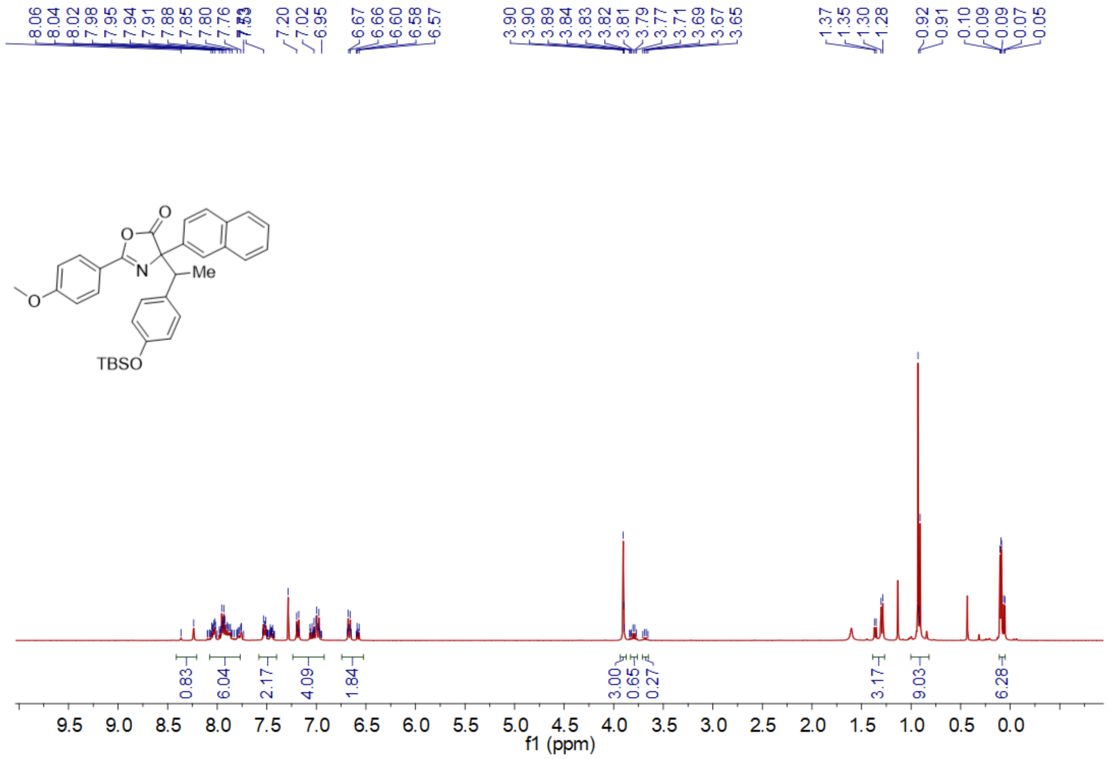

Supplementary Figure 230 ^{1}H NMR (400 MHz, $CDCl_3$, 25 °C) of compound **88**

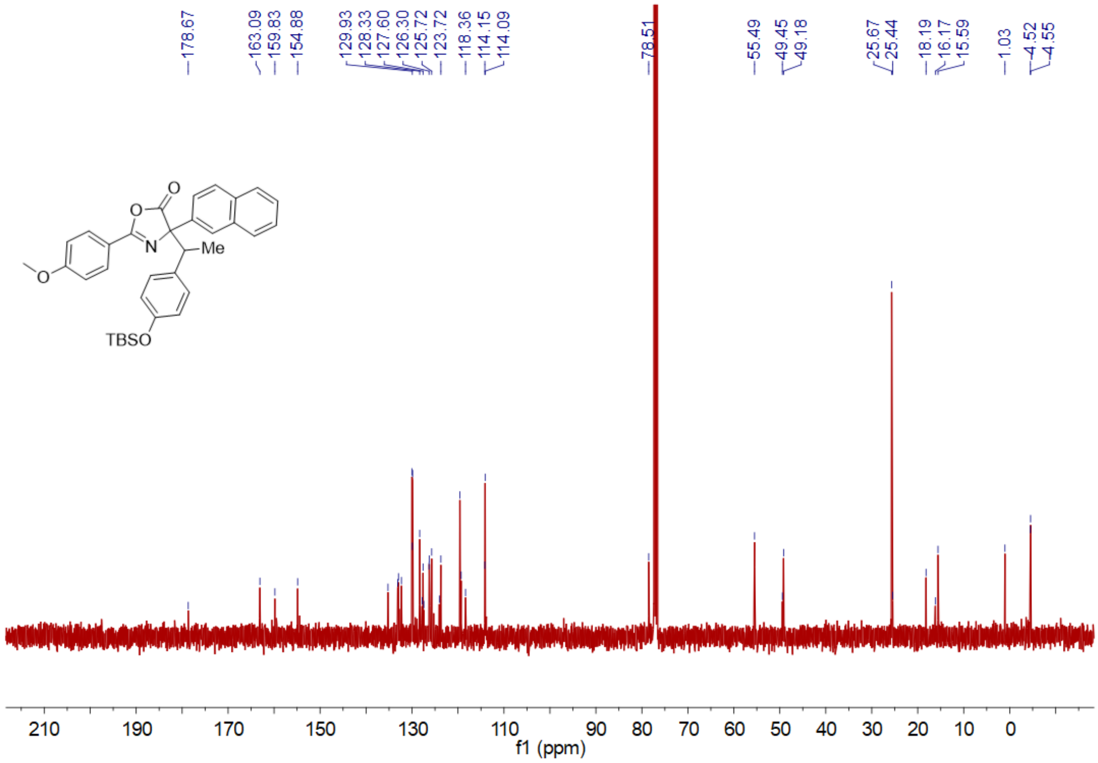

Supplementary Figure 231 ^{13}C NMR (101 MHz, $CDCl_3$, 25 °C) of compound **88**

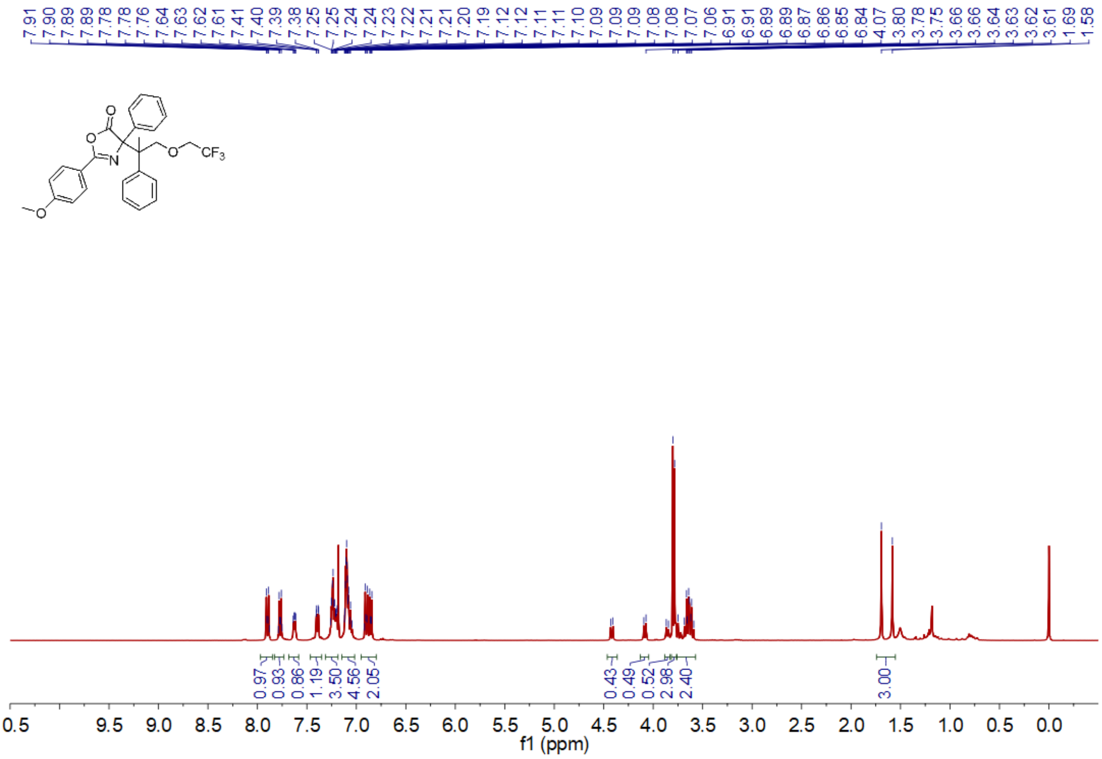

Supplementary Figure 232 ^{1}H NMR (400 MHz, $CDCl_3$, 25 °C) of compound **91**

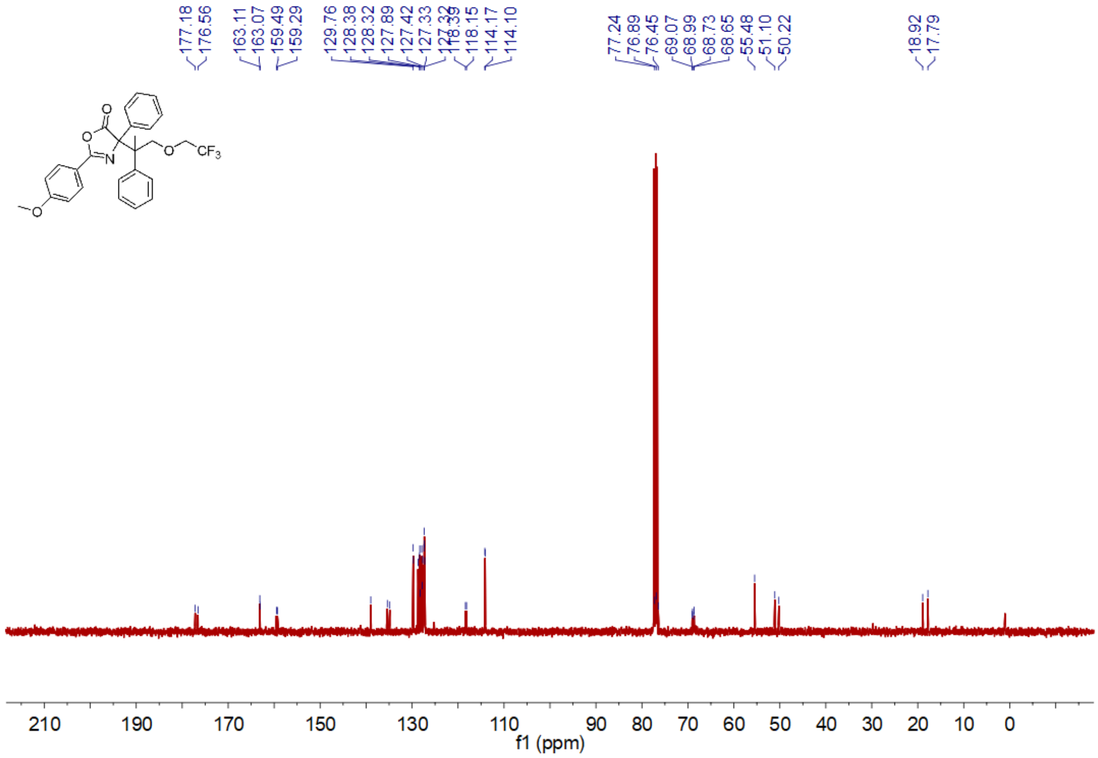

Supplementary Figure 233 ^{13}C NMR (101 MHz, $CDCl_3$, 25 °C) of compound **91**

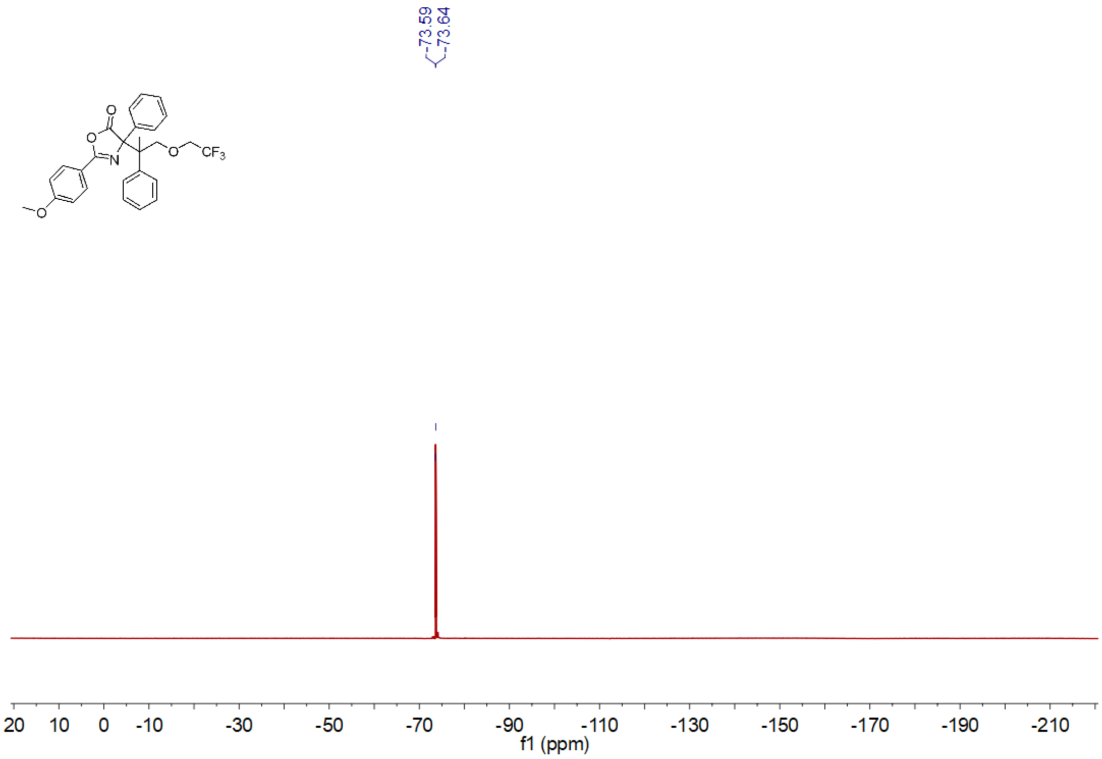

Supplementary Figure 234 ^{19}F NMR (377 MHz, $CDCl_3$, 25 °C) of compound **91**

HPLC Chromatographs

<Chromatogram>

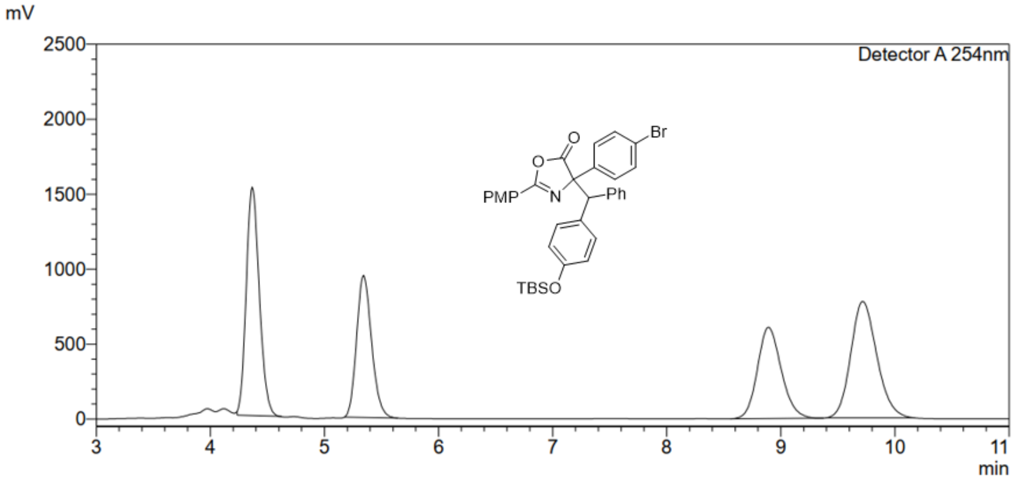

<Peak Table>

Detector A 254nm

Peak#	Ret. Time	Height	Height%	Area	Area%
1	4.367	1521372	39.459	12142583	28.882
2	5.342	947340	24.570	8675701	20.636
3	8.891	608841	15.791	8854166	21.060
4	9.717	778066	20.180	12369697	29.422
Total		3855618	100.000	42042147	100.000

Supplementary Figure 235 Racemic **85**

<Chromatogram>

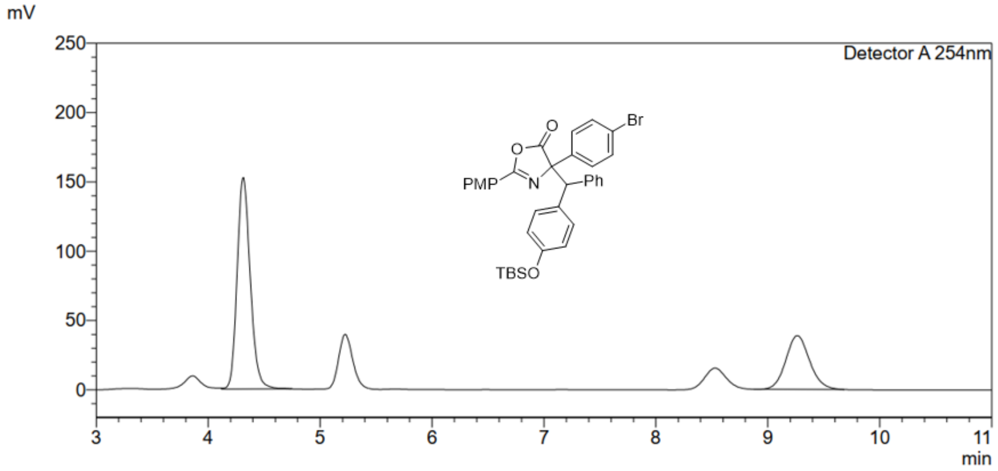

<Peak Table>

Detector A 254nm

Peak#	Ret. Time	Height	Height%	Area	Area%
1	4.313	152490	79.713	1247329	68.475
2	9.263	38808	20.287	574258	31.525
Total		191298	100.000	1821587	100.000

Supplementary Figure 236 Enantiomerically enriched **85**

<Chromatogram>

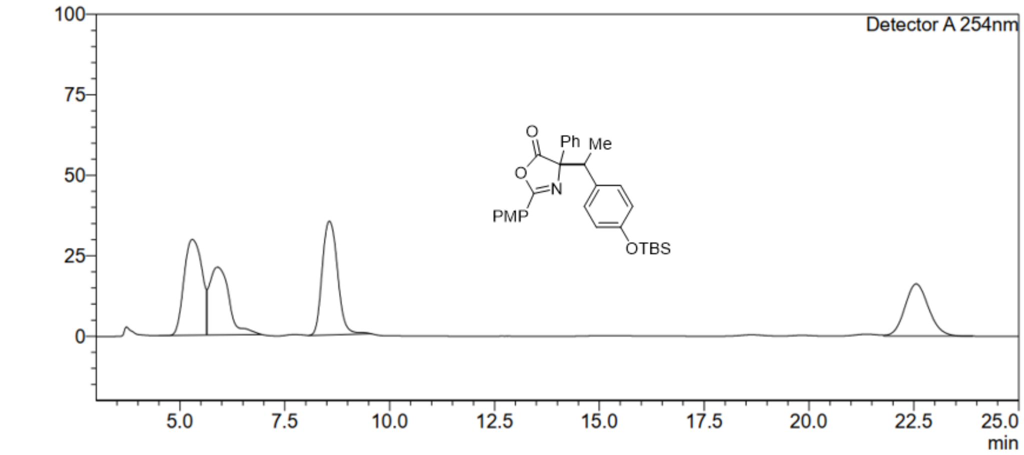

<Peak Table>

Detector A 254nm

Peak#	Ret. Time	Height	Height%	Area	Area%
1	5.294	29754	29.053	894790	28.644
2	5.892	21074	20.578	684606	21.915
3	8.563	35357	34.524	902533	28.891
4	22.556	16227	15.845	641951	20.550
Total		102412	100.000	3123879	100.000

Supplementary Figure 237 Racemic **86**

<Chromatogram>

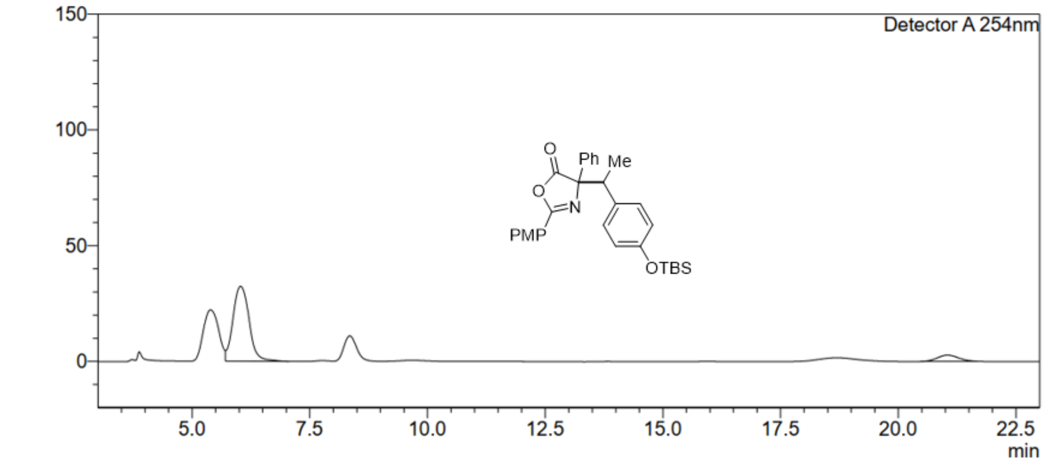

<Peak Table>

Detector A 254nm

Peak#	Ret. Time	Height	Height%	Area	Area%
1	6.031	32393	92.292	774481	90.080
2	21.047	2705	7.708	85292	9.920
Total		35099	100.000	859773	100.000

Supplementary Figure 238 Enantiomerically enriched **86**

<Chromatogram>

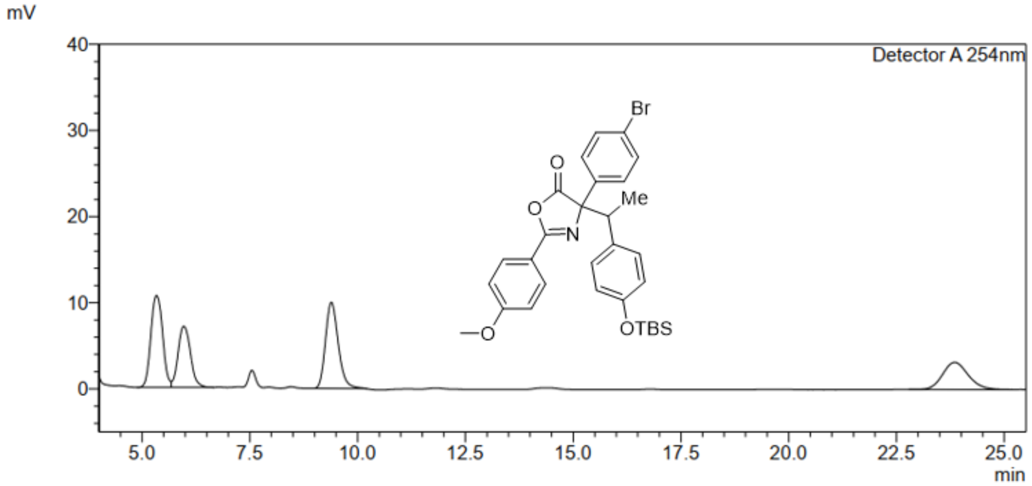

<Peak Table>

Detector A 254nm

Peak#	Ret. Time	Height	Height%	Area	Area%
1	5.334	10670	34.479	202585	29.616
2	5.966	7097	22.933	142489	20.831
3	9.390	10017	32.368	207885	30.391
4	23.850	3163	10.220	131074	19.162
Total		30947	100.000	684033	100.000

Supplementary Figure 239 Racemic **87**

<Chromatogram>

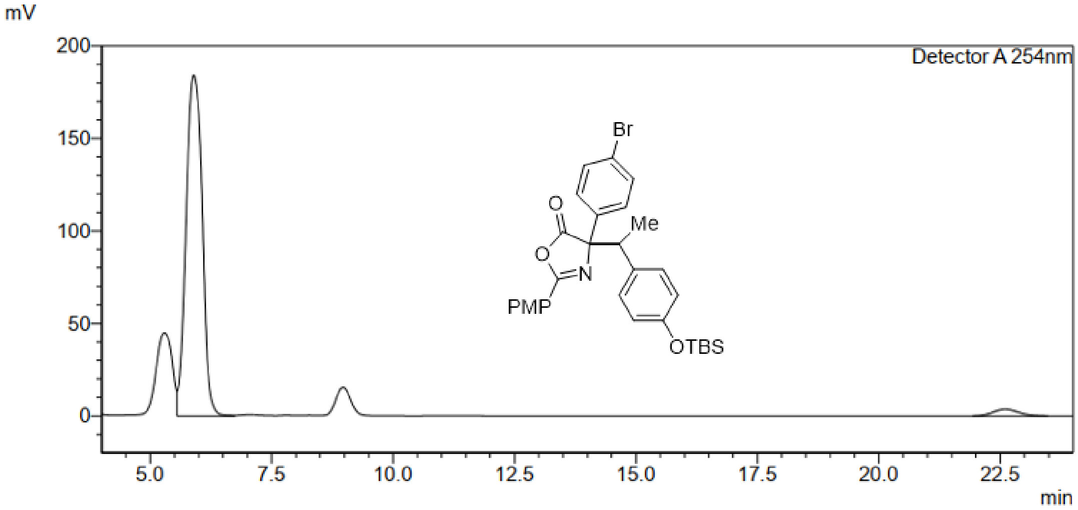

<Peak Table>

Detector A 254nm

Peak#	Ret. Time	Height	Height%	Area	Area%
1	5.897	184138	98.094	4265962	96.997
2	22.605	3578	1.906	132079	3.003
Total		187716	100.000	4398041	100.000

Supplementary Figure 240 Enantiomerically enriched **87**

<Chromatogram>

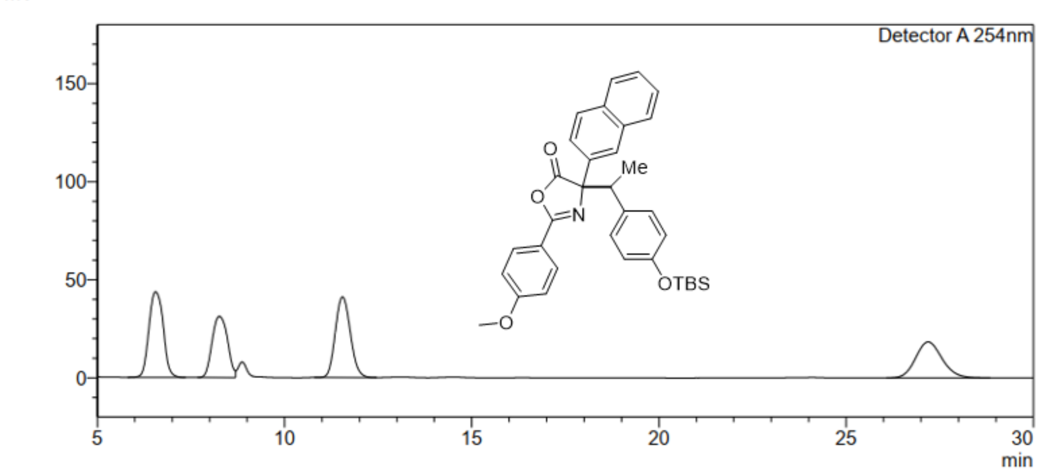

<Peak Table>

Detector A 254nm

Peak#	Ret. Time	Height	Height%	Area	Area%
1	6.555	43595	32.486	1163335	28.329
2	8.258	31210	23.257	893637	21.761
3	11.547	41049	30.589	1151203	28.033
4	27.190	18341	13.668	898414	21.877
Total		134195	100.000	4106588	100.000

Supplementary Figure 241 Racemic **88**

<Chromatogram>

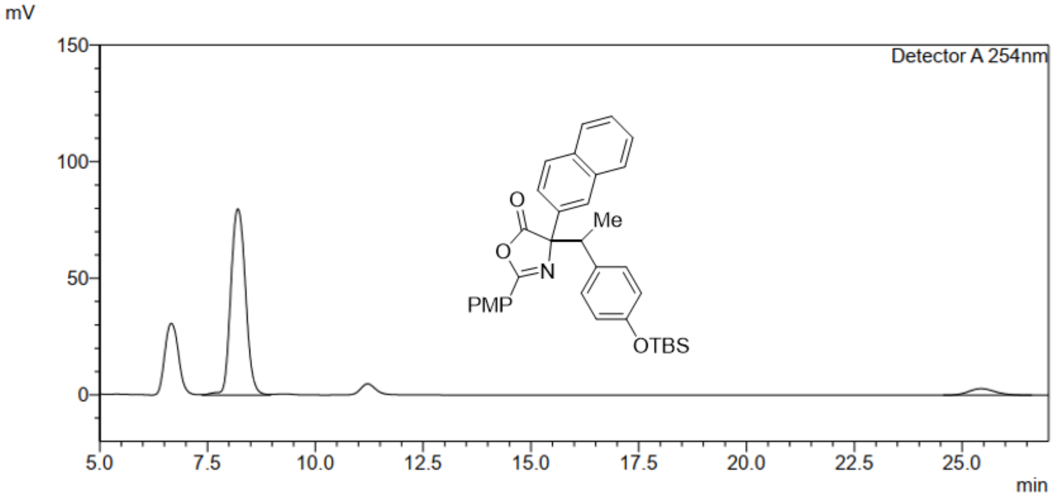

<Peak Table>

Detector A 254nm

Peak#	Ret. Time	Height	Height%	Area	Area%
1	8.203	79820	96.623	1896388	94.044
2	25.439	2790	3.377	120112	5.956
Total		82610	100.000	2016501	100.000

Supplementary Figure 242 Enantiomerically enriched **88**

Supplementary References

1. Cismesia, M. A., & Yoon, T. P. Characterizing Chain Processes in Visible Light Photoredox Catalysis. *Chem. Sci*.**6**, 5426-5434 (2015).
2. Li, Y. et al. Modular Construction of Unnatural α-Tertiary Amino Acid Derivatives by Multicomponent Radical Cross-Couplings. *Angew. Chem. Int. Ed*. **61**, e202210755 (2022).
3. Wegner, E. E. & Adamson, A. W. Photochemistry of Complex Ions. III. Absolute Quantum Yields for the Photolysis of Some Aqueous Chromium(III) Complexes. Chemical Actinometry in the Long Wavelength Visible Region. *J. Am. Chem. Soc*. **88**, 394–404 (1966).
4. Tsuji, T., Tanaka, T., Tanaka, T., Yazaki, R. & Ohshima, T. Catalytic aerobic cross-dehydrogenative coupling of azlactones en route to α,α-disubstituted α-amino acids. *Org. Lett*. **22**, 4164-4170 (2020).
5. Shu, C., Noble, A. & Aggarwal, V. K. Metal-free photoinduced C(sp^3)–H borylation of alkanes. *Nature* **586**, 714-719 (2020).
6. Candish, L., Teders, M. & Glorius, F. Transition-metal-free, visible-light-enabled decarboxylative borylation of aryl N-hydroxyphthalimide esters. *J. Am. Chem. Soc*. **139**, 7440-7443 (2017).
